# Identification of Susceptibility Genes in *Castanea sativa* and Their Transcription Dynamics following Pathogen Infection

**DOI:** 10.3390/plants10050913

**Published:** 2021-05-02

**Authors:** Vera Pavese, Andrea Moglia, Paolo Gonthier, Daniela Torello Marinoni, Emile Cavalet-Giorsa, Roberto Botta

**Affiliations:** Dipartimento di Scienze Agrarie, Forestali e Alimentari-DISAFA, Università degli Studi di Torino, Largo Paolo Braccini 2, Grugliasco, 10095 Torino, Italy; vera.pavese@unito.it (V.P.); paolo.gonthier@unito.it (P.G.); daniela.marinoni@unito.it (D.T.M.); emile.cavaletgiorsa@unito.it (E.C.-G.); roberto.botta@unito.it (R.B.)

**Keywords:** chestnut, susceptibility genes, *Phytophthora cinnamomi*, *Cryphonectria parasitica*

## Abstract

*Castanea sativa* is one of the main multipurpose tree species valued for its timber and nuts. This species is susceptible to two major diseases, ink disease and chestnut blight, caused by *Phytophthora* spp. and *Cryphonectria parasitica*, respectively. The loss-of-function mutations of genes required for the onset of pathogenesis, referred to as plant susceptibility (S) genes, are one mechanism of plant resistance against pathogens. On the basis of sequence homology, functional domain identification, and phylogenetic analyses, we report for the first time on the identification of S-genes (*mlo1*, *dmr6*, *dnd1*, and *pmr4*) in the *Castanea* genus. The expression dynamics of S-genes were assessed in *C. sativa* and *C. crenata* plants inoculated with *P. cinnamomi* and *C. parasitica*. Our results highlighted the upregulation of *pmr4* and *dmr6* in response to pathogen infection. *Pmr4* was strongly expressed at early infection phases of both pathogens in *C. sativa*, whereas in *C. crenata*, no significant upregulation was observed. The infection of *P. cinnamomi* led to a higher increase in the transcript level of *dmr6* in *C. sativa* compared to *C. crenata*-infected samples. For a better understanding of plant responses, the transcript levels of defense genes *gluB* and *chi3* were also analyzed.

## 1. Introduction

The *Castanea* genus belongs to the Fagaceae family and includes four major species of commercial and ecosystemic interest: *Castanea sativa* Mill. (European chestnut), *Castanea crenata* Sieb. et Zucc. (Japanese chestnut), *Castanea mollissima* Bl. (Chinese chestnut), and *Castanea dentata* Borkh (American chestnut). *C. sativa* is a woody species common in all Mediterranean countries and Asia Minor. It has been widely used since ancient times, not only for the consumption of its edible nuts, but also for wood and the products of its ecosystem, such as mushrooms and honey. It is a forest tree, relevant for landscape ecology and biodiversity of mountain and rural areas [1].

Over the last century, the number of chestnut trees decreased in growing areas in Europe due to the depopulation of mountains, climate change, and the spread of two severe diseases: ink disease and chestnut blight [2,3]. Ink disease is caused by the Oomycete *Phytophthora cinnamomi* and *Phytophthora cambivora*. Both species are pathogenic to *C. sativa*, although *P. cinnamomi* generally displays greater virulence than *P. cambivora* [4,5]. Among *Castanea* species, only *C. crenata* exhibits high tolerance to *P. cinnamomi* [6]. The disease, which affects both young and old trees, leads to subcortical necrosis of the root system and the basal part of the stem; this is followed by the appearance of wasting symptoms in the foliage until the total desiccation and death of the plant occur [7,8,9,10]. These pathogens spread mainly through the movement of soil harboring inoculum and the dissemination of asexual flagellated spores (i.e., zoospores) that can actively travel short distances or passively travel long distances in flowing water [10,11]. The use of resistant rootstocks represents one possible solution to protect against these pathogens, although, at present, only tolerant selections obtained from hybridization between *C. sativa* and *C. crenata* are available [12].

Chestnut blight stands among the most destructive fungal tree diseases ever [10,13]. The causal agent, *Cryphonectria parasitica*, infects trees through dead plant tissue and wounds, including those caused by pruning, graft, and hail [13,14]. The symptoms involve bark cankers that can develop on suckers, young branches, and adult branches and trunks [15]. Chestnut blight was one of the causes of the abandonment of chestnut orchards in Europe until the end of the 1970s, when the natural spread of the hypovirulent form of the fungus favored a slow but progressive recovery of chestnut orchards and coppices. However, the fungus still represents a relevant problem in many areas of Europe. It is very harmful to young grafted trees in particular, hampering the establishment of new orchards in many areas [10,13]. 

*C. dentata* forests in Eastern North America were wiped out by *C. parasitica* in the early 20th century [16]. Extensive studies and breeding activities have been carried out to restore the American chestnut species introgressing resistance genes of *C. mollissima* [17,18]. More recently, researchers discovered that the onset of the disease is associated with the release of oxalic acid by the pathogen during infection. Blight-resistant *C. dentata* trees were obtained by transferring a wheat gene that encodes oxalate oxidase [19].

Recently, a new interest and sensitivity towards the preservation of the local landscape generated a growing interest in silviculture and chestnut trees [20]. Moreover, the market demand for chestnuts in European countries has been strong in the last two decades and has often been supplied by importations. This has been due in part to the gall wasp (*Dryocosmus kuriphilus* Yasumatsu) infestation, which only recently has been controlled effectively [1,21], and to the general difficulty of developing a modern chestnut industry based on quality cultivars of *C. sativa* that are more tolerant to pathogens. The elucidation of the genetic mechanism behind host–pathogen interaction could thus be useful for the development of novel breeding strategies aimed at achieving resistance or higher tolerance to these pathogens. 

Plants take advantage of different defense mechanisms during pathogen attack, and pathogens trigger counter-defense mechanisms. Plants carry pattern recognition receptors (PRRs) able to perceive pathogen-associated molecular patterns (PAMPs); this perception leads to intracellular signal transduction culminating in PAMP-triggered immunity (PTI). PTI is characterized by the production of reactive oxygen species (ROS), the secretion of antimicrobial compounds, and hydrolytic enzymes targeting the pathogen cell wall (chitinase and glucanase) and local cell wall fortifications (through callose deposition) [22].

To suppress PTI, pathogens developed effector molecules able to facilitate pathogen infection by manipulating the host response to support compatibility. Plant resistance (R) genes can detect effectors and trigger effector-triggered immunity (ETI) [23]. The recognition between R genes and effectors causes a cascade of responses involving jasmonic acid (JA) and salicylic acid (SA), culminating in a hypersensitive response (HR) [24].

Most pathogens require the cooperation of the host to establish a compatible interaction. Plant genes supporting compatibility and facilitating infection are called susceptibility (S) genes. S-genes can be divided into three main classes: (a) genes required for the early pathogen infection step (basic compatibility); (b) genes encoding negative regulators of plant immunity; (c) genes necessary for pathogen proliferation (sustained compatibility) [22].

The mutation or loss of an S-gene can thus limit the ability of the pathogen to infect the host and the spread of the disease. The resistance mediated by the S-gene mutation can be pathogen-specific or broad-spectrum. In the former case, the pathway can be implicated in the penetration phase; in the latter, one of the target genes can be involved in constitutive defense responses [22]. Resistance due to the loss of S-genes is generally recessive, differing from the generally dominant resistance mediated by R genes.

Among the S-genes, *Mildew resistance locus O* (*mlo1*), *Powdery mildew resistance 4* (*pmr4*), *Downy Mildew Resistance 6* (*dmr6*), and *Defense no death* (*dnd1*) have been characterized in many plant species. The *Mlo* gene family, encoding seven transmembrane domain proteins, has been characterized in many plant species [25]. Some *mlo* homologs act as PM susceptibility factors, as their loss of function results in a distinguished type of resistance known as *mlo* resistance. Originally discovered in barley (*Hordeum vulgare* L.), *mlo* resistance was later shown to occur in several monocot and eudicot species, namely Arabidopsis, tomato (*Solanum lycopersicum* L.), pea (*Pisum sativum* L.), pepper (*Capsicum annum* L.), tobacco (*Nicotiana tabacum* L.), and wheat (*Triticum aestivum* L.) [26,27] plants. The callose synthase encoded by *pmr4* is responsible for the production of callose in response to biotic and abiotic stresses. In tomato and potato plants, the knockout and silencing of *pmr4* led to *Oidium neolycopersici* and *Phytophthora infestans* tolerance [28,29]. *Dmr6* is involved in the conversion of salicylic acid (SA) to 2,3-dihydroxybenzoic acid (2,3-DHBA) and negatively regulates defense gene expression [30]. Its silencing caused resistance to hemi-biotrophic *Phythophthora capsici*, *Hyaloperonospora arabidopsidis*, and *Pseudomonas syringae* [31]. Mutants of *dnd1*, encoding for a cyclic nucleotide-gated cation channel, showed *P. infestans* resistance [29].

At the moment, studies on S-genes in woody plant species have been carried out only for *mlo* genes in rubber trees [32], poplar trees [33], apple trees, and grapevines [34]. In our work, we report on the S-genes identification and characterization in *C. sativa* on the basis of sequence homology, functional domain detection and phylogenetic relationships. In addition, the expression dynamics of S-genes were assessed in *C. sativa* and *C. crenata* plants inoculated with the two pathogens, *P. cinnamomi* and *C. parasitica*, belonging to different kingdoms. Using the same plant material, the transcription levels of key genes involved in pathogen resistance, *chi3* (*acidic 26 kDa endochitinase*) and *gluB* (*glucan endo-1,3-beta- glucosidase)*, were also determined (S1 File). Our analysis revealed the strong activation of *pmr4* and *dmr6 genes* in response to infection by both *P. cinnamomi* and *C. parasitica*.

## 2. Results

### 2.1. Genes Identification and Structure

**Figure 1:**
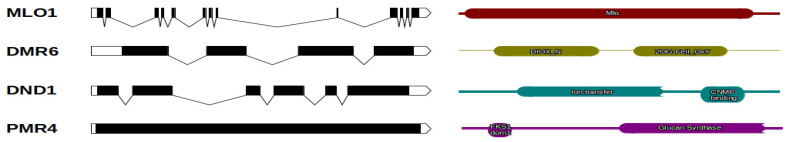
Chestnut S-genes and their protein structures. The graphical representations of gene exon/intron structures were generated using the http://wormweb.org/exonintron tool (accessed on 31 March 2021) and are shown in the left panel. The exons are indicated with black boxes, whereas introns are shown with lines. In the right panel, the protein structural domains are displayed.

Chestnut susceptible (S) genes were identified in the *C. mollissima* v1.1 reference genome using available coding sequences of gene orthologs as a query (S2 File). Based on the blastn survey, four loci with high similarity were identified and attributed to different subclasses of S-genes due to the presence of specific domains: *mlo1*, *dmr6*, *dnd1*, and *pmr4* (Figure 1). The coding sequence length of *mlo1* is 1425 bp (composed of 13 exons); the protein size is 474 amino acids (aa) (Table 1). A single *Mlo* domain (PF03094) is present within the protein sequence. The *Dmr6* gene, whose coding sequence is 1128 bp, contains four exons and is translated into a 375 aa protein (Table 1). Two specific domains are characterized: 2OG-FeII_Oxy and DIOX_N (PF03171; PF14226). *Dnd1* is 1407 bp in length, codes for 468 aa proteins, and is composed of six exons. Two structural domains, cNMP_binding and Ion_trans, were highlighted (PF00027; PF00520) (Figure 1; Table 1). The *Pmr4* gene is characterized by one single 5346 bp exon. The protein size is 1781 aa, and the structural domains are FKS1 dom1 and Glucan_synthase (PF14288; PF02364) (Table 1).

### 2.2. Phylogenetic and Modeling Analysis

**Figure 2:**
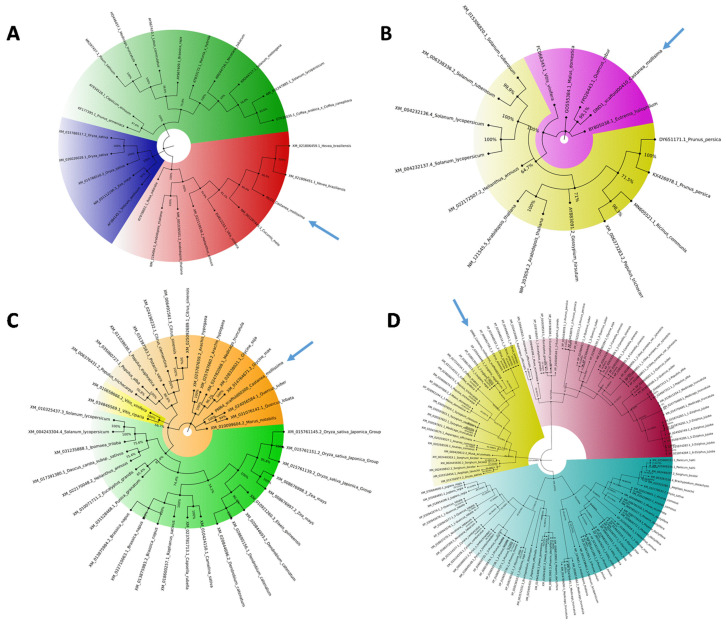
Phylogenetic analysis of the S-genes. The 4 phylogenetic trees of mlo1 (**A**), dnd1 (**B**), pmr4 (**C**), and dmr6 (**D**) were constructed using MEGAX software by aligning chestnut S-gene coding sequences with NCBI S-gene ortholog coding sequences (available in S3 File). The colors indicate the main clades detected, and the arrows underline the location of *C. mollissima*. To visualize details, all the phylogenetic trees are available in S4 File.

Available full-length NCBI S-gene coding sequence orthologues (S3 File) were used for phylogenetic tree construction. The resulting unrooted maximum-likelihood trees are shown in Figure 2, and all the phylogenetic trees are available separately in the S4 File.

The *mlo1* tree was grouped into three clades (blue, green, red) with 100% bootstrap value. The monocot proteins formed a separate clade (blue) with respect to those of the dicotyledonous species. *CmMlo1* is located in the red clade with *Vitis vinifera* and *Hevea Brasiliensis* orthologs (Figure 2A). The 17 *dnd1* coding sequences were divided into two subclades; no monocotyledon genes were included. *CmDnd1* is in the violet subclade, phylogenetically close to *Quercus robur* ortholog (Figure 2B). For the construction of *pmr4* and *dmr6* trees, a greater number of coding sequences were available: 40 and 115, respectively. The *Pmr4* phylogenetic tree showed the division in three main clades, with monocots in the green clade intermixed with dicots. *CmPmr4* is located in the orange clade and clusters together with *Quercus lobata* and *Quercus* spp. orthologs (Figure 2C). The *Dmr6* tree is divided into three clades intermixed with monocot/dicot proteins. *CmDmr6* is in the yellow clade, phylogenetically close to the *Juglans regia* ortholog gene (99% bootstrap value) (Figure 2D).

**Figure 3:**
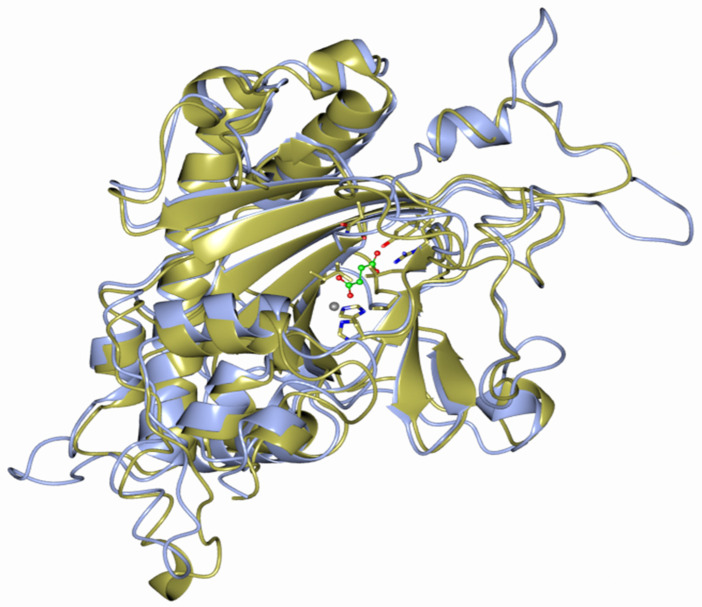
The DMR6 3D protein model created using Modeller software and visualized using Ccp4mg software. The *C. mollissima* (Cm) DMR6 (blue) protein and A. thaliana (At) DMR6 (yellow) protein are shown.

By comparing the 3D protein structure model of *C. mollissima DMR6* with *Arabidopsis thaliana DMR6* via Modeller software, a high degree of structural conservation was observed (Figure 3). DMR6 is a putative oxygenase involved in the conversion of salicylic acid (SA) to 2,3-dihydroxybenzoic acid (2,3-DHBA), and its catalytic activity is probably necessary to suppress plant immunity. The catalytic triad that binds the iron atom (grey sphere) is made by two histidines (H212 and H269), and an aspartic acid residue (D214). Zeilmaker et al. [31] demonstrated, by removing the histidines, that these residues were fundamental for the catalytic activity of DMR6, as well as for its role in immune suppression.

### 2.3. Transcriptional Profiling in Response to P. cinnamomi Infection

S-genes are genes related to plant–pathogen interaction and are supposed to be activated during the early stages of infection before symptoms emerge. The susceptible species *C. sativa* was used as a reference to define the onset of symptoms due to *P. cinnamomi* inoculation on the stem. Five days after inoculation, lesions [35] were observed, followed by total leaf desiccation. Based on the evidence from the preliminary inoculation tests, *C. sativa* and *C. crenata* species inoculated with *P. cinnamomi* and samples were collected at 0, 3, 6, 12, 24, 48, and 72 h post-inoculation (hpi). The wound area left a lesion of 0.5 cm^2^. *C. sativa* plants inoculated with *P. cinnamomi* showed an enlargement of the lesion of 0.6 cm^2^ at 24, 48, and 72 hpi compared to the initial area of the lesion at time 0 (control). No visible enlargement of lesions was recorded in the case of *C. crenata*.

**Figure 4:**
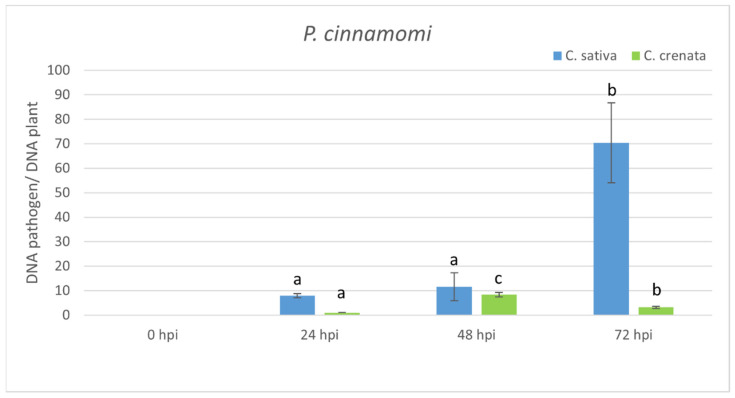
qRT-PCR pathogen DNA quantification after *P. cinnamomi* inoculation. Data were quantified using the 2^−ΔΔCt^ method based on the Ct values of pathogen genes (ypt and mf1) and actin-7 as a housekeeping gene. Data are the means of three biological replicates ± SE. *C. sativa* data are normalized with *C. sativa* 0 hpi control; *C. crenata* data are normalized with *C. crenata* 0 hpi control. Different letters associated with the set of means indicate a significant difference based on Tukey’s HSD test (*p* ≤ 0.05).

**Figure 5:**
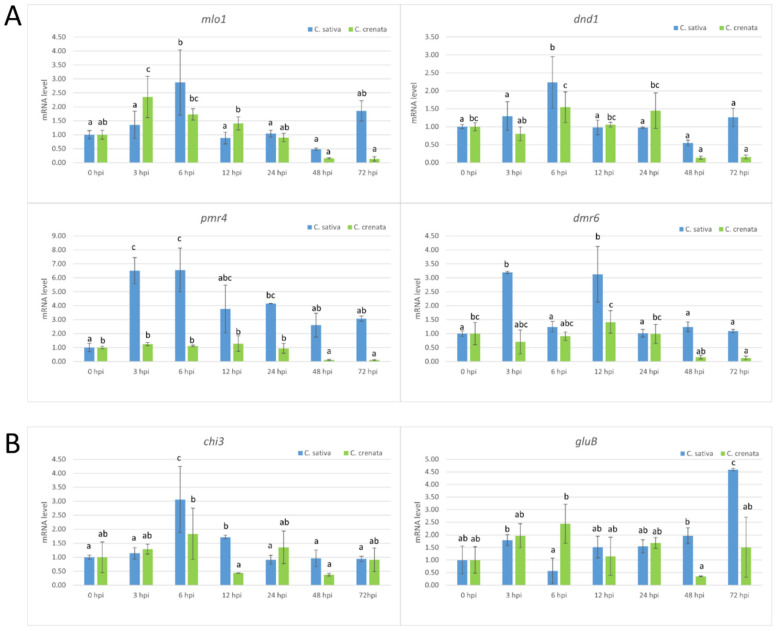
qRT-PCR-based transcription profiling after *P. cinnamomi* inoculation. (**A**) The S-gene transcription profiles in *C. sativa* (blue) and *C. crenata* (green) chestnut species. (**B**) The expression analysis of genes coding for several pathogenesis-related (PR) proteins in *C. sativa* (blue) and *C. crenata* (green) species. In all analyses, Cm7-actin was used as a housekeeping gene. Data are the means of three biological replicates ± SE. *C. sativa* data are normalized with *C. sativa* 0 hpi control; *C. crenata* data are normalized with *C. crenata* 0 hpi control. Different letters associated with the set of means indicate a significant difference based on Tukey’s HSD test (*p* ≤ 0.05).

Quantitative PCR from infected stem tissues was applied to quantify *P. cinnamomi* and the assay confirmed a higher amount of the pathogen in plant tissues at 72 hpi (Figure 4). *C. crenata* showed a lower abundance of the pathogen compared to *C. sativa* at all tested experimental time points. S-gene expression was analyzed using the same time points (Figure 5A). *Mlo1* was mainly expressed at early infection phases, peaking at 6 and 3 hpi in *C. sativa* and *C. crenata*, respectively. *Dnd1* showed an analogous trend in both plant species, and its transcription was strongest at 6 hpi. Regarding *pmr4*, a differential transcript regulation in response to *P. cinnamomi* infection was highlighted in *C. sativa* and *C. crenata.* In *C. sativa*, *pmr4* was strongly expressed at 3 and 6 hpi, with transcript level around 6-fold higher compared to 0 hpi. On the contrary, the expression of p*mr4* remained very limited in *C. crenata* infected tissues, and a significant downregulation at 48 and 72 hpi was observed. Significant increases in the transcript level of *dmr6* were observed only at 12 hpi (for *C. crenata*) and at 3 and 12 hpi (for *C. sativa*). For a better understanding of the plant response against *P. cinnamomi*, genes coding for pathogenesis-related (PR) proteins, *chi3* and *gluB*, were analyzed (Figure 5B). *Chi3* peaked at 6 hpi both in *C. sativa* and in *C. crenata*, but the increment was higher in the former species (~3 fold vs. ~2 fold). *GluB* increased with the progression of infection in *C. sativa*, whereas a higher upregulation was observed during the early infection phases (at 3 and 6 hpi) in *C. crenata*.

### 2.4. Transcriptional Profiling in Response to C. parasitica Infection

As described for *P. cinnamomi*, a preliminary stem inoculation assay on *C. sativa* plants was done with *C. parasitica*. Seven days after inoculation, a necrotic lesion around the inoculation point and orange fruiting bodies were observed in *C. sativa*. Based on the results of the preliminary inoculation test, *C. sativa* and *C. crenata* plants were inoculated and sampled at 0, 12, 24, 48, 72, 96, and 120 hpi. The size of the wound area was 0.5 cm^2^. *C. sativa* plants inoculated with *C. parasitica* showed enlargements of the lesion of 0.5 cm^2^ (at 48 and 120 hpi) and of 1 cm^2^ (at 96 hpi) compared to the initial wound area at 0 hpi. No visible enlargement of the inoculation lesions was recorded in *C. crenata* at 72 and 120 hpi, and a limited enlargement of 0.5 cm^2^ was observed at 96 hpi. 

**Figure 6:**
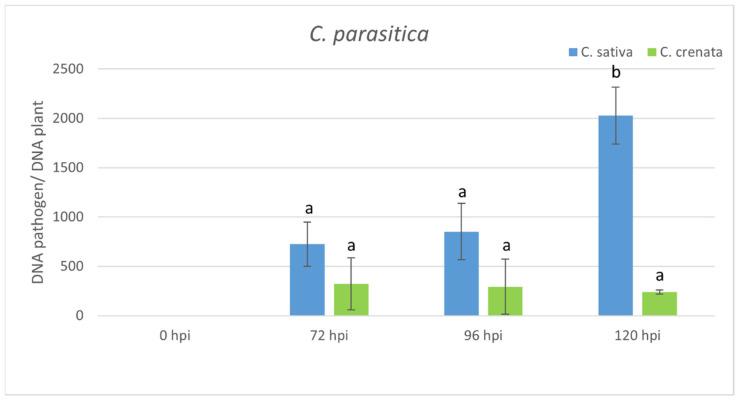
qRT-PCR pathogen DNA quantification after *C. parasitica* inoculation. Data were quantified using the 2^−ΔΔCt^ method based on the Ct values of fungal genes (ypt and mf1) with actin-7 as a housekeeping gene. Data are the means of three biological replicates ± SE. *C. sativa* data are normalized with the *C. sativa* 0 hpi control; *C. crenata* data are normalized with *C. crenata* 0 hpi control. Different letters associated with the set of means indicate a significant difference based on Tukey’s HSD test (*p* ≤ 0.05).

**Figure 7:**
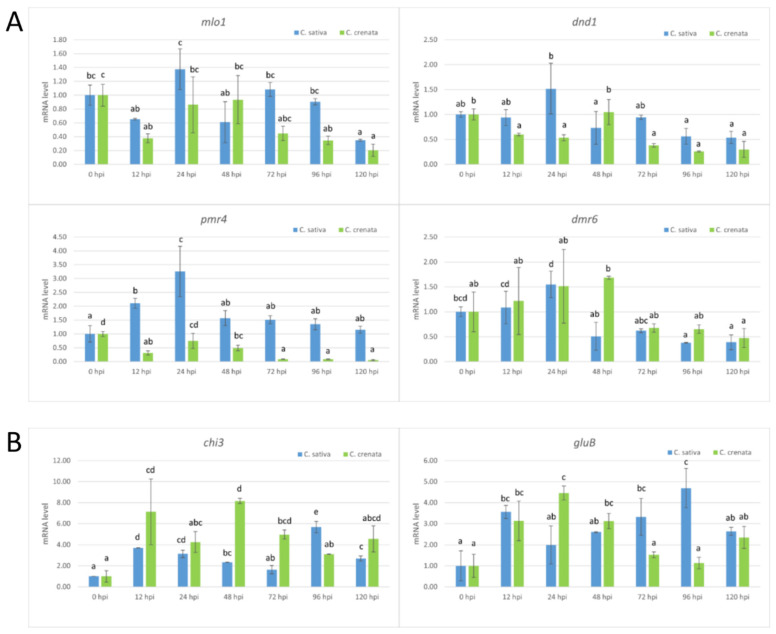
qRT-PCR-based transcription profiling after *C. parasitica* inoculation. (**A**) The S-gene transcription profile in *C. sativa* (blue) and *C. crenata* (green) chestnut species. (**B**) The expression analysis of genes coding for several pathogenesis-related (PR) proteins of *C. sativa* (blue) and *C. crenata* (green) species. In all the analyses, Cm7-actin was used as the housekeeping gene. The data are the means of three biological replicates ± SE. *C. sativa* data are normalized with *C. sativa* 0 hpi control; *C. crenata* data are normalized with *C. crenata* 0 hpi control. Different letters associated with the set of means indicate a significant difference based on Tukey’s HSD test (*p* ≤ 0.05).

In *C. sativa*, qPCR analysis showed an increase in the abundance of pathogen inoculum with time elapsing from infection, peaking at 72 hpi. In *C. crenata*, no statistical differences were observed among the different experimental times (from 72 to 120 hpi) (Figure 6). The transcript levels of S-genes were analyzed using the same time points (Figure 7A). *Mlo1* was mainly expressed at 24 hpi in *C. sativa*. No significant upregulation of *mlo1* was observed in *C. crenata-*infected tissues. An upregulation of *dnd1* was detected at 24 hpi in *C. sativa* infected tissues. In *C. sativa*, *pmr4* was strongly expressed at the early infection phases (12 and 24 hpi), with transcript levels around 3-fold higher as compared with the level at 0 hpi. On the contrary, *pmr4* was downregulated at all experimental times in inoculated *C. crenata* plants. A significant upregulation of *dmr6* was observed only at late infection phases, 48 hpi in *C. crenata* and 24 hpi in *C. sativa*. The transcription of *chi3* was strongest at 96 hpi in *C. sativa* and at 48 hpi in *C. crenata.* The transcript level of *gluB* increased until reaching 96 hpi in *C. sativa* and 24 hpi in *C. crenata* (Figure 7B).

## 3. Discussion

*C. sativa* is a European woody tree species commonly used across the globe in the food and timber industries. This chestnut species is susceptible to the two major pathogens, *P. cinnamomi* and *C. parasitica* [10,36]. In contrast, the Asian chestnut species *C. crenata* and *C. mollissima* exhibit higher tolerance to *P. cinnamomi* and *C. parasitica* [6]. Achieving tolerance or resistance to pathogens is the major aim of rootstock breeding. Blight-resistant trees were obtained through backcross breeding of introgression genes from Asian species into American chestnut trees. [37]. However, this approach, although successful in developing blight-resistant American chestnut selections has been slowed by a lack of genetic tools. In Europe, ink disease tolerant hybrids were obtained through interspecific crosses between *C. sativa* and *C. crenata*, although the nut quality produced by these hybrids is below current market standards [38,39].

It has long been recognized that a deep understanding of a pathogen’s biology, host–pathogen interactions, and the resistance mechanisms are fundamental to improving breeding programs. Genomic and transcriptomic analyses have provided the first genetic insights into mechanisms underlying susceptible and resistant chestnut species responses to *P. cinnamomi* and *C. parasitica* [37,38,40,41,42]. Santos et al. [40] reported the upregulation of a set of candidate genes (e.g., *Cast_Gnk2-like* and *Calcium-dependent protein kinase*) after *P. cinnamomi* infection, which may trigger HR-like cell death in *C. crenata* cells. A significant number of genes involved in the defense against chestnut blight were identified [37].

Traditionally, the introduction of resistance gene analogues into plants was the most promising approach to facilitate the acquisition of resistance. However, it did not prove to be durable enough because the widespread use of R genes caused the selection of pathogens capable of overcoming them [24]. Susceptibility (S) genes can be interesting candidates to be used in target breeding programs [22,23,24]. Furthermore, on the basis of previous studies, it was highlighted that the disabling of susceptibility genes may facilitate durable resistance since the pathogen needs to gain a new function to replace the lost host factor it was exploiting [43].

In woody species, the investigation of S-genes has been performed only for MLO genes in rubber trees [32], poplar trees [33], apple trees, and grapevines [34,44]. Due to the absence of a *C. sativa* genome, highly similar S-genes were selected using the *C. mollissima* v 1.1 genome. Based on the blastn survey, four loci with high similarity were identified in the *C. mollissima* genome and attributed to different subclasses of S-genes [31,45,46,47] due to the presence of specific domains: *mlo1*, *dmr6*, *dnd1*, and *pmr4* (Figure 1, Table 1). As previously observed [31], in the phylogenetic trees, monocot proteins formed a separate clade with respect to those of dicotyledonous species, supporting the hypothesis that an independent evolution occurred for these genes (Figure 2). Quantitative PCR analysis has been carried out to identify the differential expression of candidate S-genes in response to *P. cinnamomi* and *C. parasitica* in the stems of a susceptible species, *C. sativa*, and of a tolerant one, *C. crenata*. Lesion analysis and DNA quantification of the pathogen (Figure 4 and Figure 6) confirmed the higher tolerance level of *C. crenata* in response to both *P. cinnamomi* and *C. parasitica* infection. Our qPCR results highlighted the main upregulation of *pmr4* and *dmr6* in response to infection by both *P. cinnamomi* and *C. parasitica*. As expected, a greater increase in the transcription of these susceptibility genes was observed in the susceptible species *C. sativa*. Remarkably, *pmr4* was strongly expressed at early infection phases of both pathogens in *C. sativa*; in the tolerant *C. crenata*, significant upregulation was observed (Figure 5 and Figure 7). *Pmr4* codifies for a callose synthase, which is necessary to create a physical barrier to avoid pathogen penetration and is also implicated in plant-triggered immunity suppression. *Pmr4* is thus not only involved in the synthesis of callose, but it also acts as a negative regulator of the salicylic acid pathway [28].

Huibers et al. [48] demonstrated that resistance due to the silencing of *Pmr4* is associated with salicylic acid (SA) accumulation rather than with the callose deposition absence. Salicylic acid signaling plays a key role protecting against biotrophic pathogens through the establishment of a hypersensitive response (HR). Saiz-Fernandez et al. [49] revealed the increment of SA levels in *P. cinnamomi* inoculated stems, indicating that *P. cinnamomi* activates a defense response similar to that triggered by biotrophic pathogens. Inoculation with both virulent and hypovirulent strains of *C. parasitica* led to SA accumulation in European chestnut plantlets that were grown in vitro [50]. Transcriptome analyses carried out in both *C. dentata* and *C. mollissima* highlighted activation of salicylic-acid-related genes in canker tissues [37]. 

In chestnut trees, callose deposition around *P. cinnamomi* hyphae was detected early in the infection process; however, it does not seem to play a key role in the associated interactions since the pathogen can reach the vascular cylinder in both susceptible and resistant plant genotypes [51]. This result was validated by transcriptomes analyses of *C. sativa* and *C. crenata*, in which no overexpression of *Callose synthases* after *P. cinnamomi* infection was observed [38]. 

Based on our results and the literature, we can hypothesize that callose accumulation due to the *pmr4* upregulation in inoculated *C. sativa* lines may not be responsible for controlling *P. cinnamomi* colonization. We suggest that the upregulation of *pmr4* could lead to a negative regulation of the SA pathway that in turn provokes the susceptibility of *C. sativa* to both *P. cinnamomi* and *C. parasitica*. A clear link with SA pathway has emerged even with the other chestnut gene candidate *dmr6* (downy mildew resistance 6). The mutation of *Arabidopsis dmr6* gene, associated with salicylic acid (SA) homeostasis [31], results in the generation of plants that are resistant to bacteria and oomycetes. *Dmr6* is involved in the conversion of salicylic acid (SA) to 2,3-dihydroxybenzoic acid (2,3-DHBA) and negatively regulates the expression of defense genes (PR-1, PR-2, and PR-5) [30].

The expression trend of the *Dmr6* gene in response to *P. cinnamomi* infection turned out to be similar to the profile of *pmr4*. Indeed, *dmr6* was strongly expressed at early infection phases of *P. cinnamomi* in *C. sativa*; in *C. crenata* no significant upregulation was detected (Figure 5). No upregulation of *dmr6* in response to *C. parasitica* was highlighted in both plant species (Figure 7). We can thus hypothesize that *dmr6* upregulation observed in *C. sativa* could negatively regulate defense gene expression, leading to susceptibility to *P. cinnamomi*.

Plants produce a variety of hydrolytic defense enzymes against pathogens, including chitinases, proteases, and also glucanases [52]. The genes coding for several pathogenesis-related (PR) proteins, *Acidic 26 kDa endochitinase* gene (*chi3*) and *Glucan endo-1,3-beta-glucosidase B* gene (*gluB*), were selected in our analysis because they are considered as responsive to SA-dependent signaling [53,54]. *Chi3* and *gluB* are enzymes that cause the lysis of hyphae of various pathogens, resulting in growth inhibition [55,56,57].

In both *C. sativa* and *C. crenata* plants inoculated with *C. parasitica*, *chi3* and *gluB* were significantly upregulated. The transcription of *chi3* was higher in *C. crenata* than in *C. sativa*, presumably as a consequence of the improved defense mechanism against *C. parasitica*. Our results are in agreement with Shain et al. [58], who demonstrated the involvement of *b-1,3-glucanase* and *chitinase* in chestnut species affected by *C. parasitica*. Studies on the role of *chitinase* in blight infection mostly involved *C. sativa* as a model system [50,59]. In both *C. dentata* and *C. mollissima*, transcripts of several compounds expressing *chitinase* accumulated more in canker tissues than healthy stem tissues [37]. In order to obtain chestnut plants with potentially increased resistance/tolerance to chestnut blight, the endogenous Ch3gene encoding a chitinase-like protein was over-expressed in the European chestnut through Agrobacterium-mediated transformation [60].

The emergent CRISPR/Cas9 technology is expected to play a key role in future crop breeding as it allows highly efficient gene editing and generates genetic changes indistinguishable from those arising spontaneously in nature or through conventional breeding [61]. Several examples of edited plants resistant to fungal pathogens have been described [62,63]. For example, genome editing was successfully applied to knock out *mlo* S-genes, leading to Powdery mildew (PM) resistance [44,64,65,66]. *Pmr4* and *dmr6* loss-of-function through CRISPR/Cas reduced the susceptibility to PM in tomato plants [28,67]. In our laboratory, we are setting up a CRISPR/Cas9 transformation protocol in *Castanea sativa*. Our future goal will be to perform the functional characterization using the CRISPR/Cas9 approach of the two candidate genes (*dmr6* and *pmr4*), while checking if the two genes may also play a role in the interaction between *C. sativa* and the emergent nut rot and canker agent *Gnomoniopsis castaneae* [68].

## 4. Materials and Methods

### 4.1. Identification of Chestnut S-genes Orthologues 

S-gene sequences (S2 File), available in the NCBI database (https://www.ncbi.nlm.nih.gov/, accessed on 31 March 2021), were used as a query in the BLAST+ program (blastn task) against *Castanea mollissima* v1.1 reference genome (https://www.hardwoodgenomics.org/Genome-assembly/1962958, accessed on 31 March 2021) to find chestnut S-gene orthologs. Hits were filtered using the e-value cut-off of 1 × 10^−5^. 

The domain structures of chestnut S-genes were predicted using Pfam (pfam.xfam.org/, accessed date 31 March 2021) and Uniprot (https://www.uniprot.org/, accessed on 31 March 2021) databases. The graphical gene structure with exons/introns representation was realized using the script accessible at http://wormweb.org/exonintron (accessed on 31 March 2021).

### 4.2. Phylogenetic Analysis

The alignment of chestnut S-gene coding sequences (*mlo*, *pmr4*, *dmr6*, and *dnd1)* and of known related S-genes in other plant species were generated via multiple sequence alignment using the ClustalW algorithm (http://www.clustal.org/, accessed on 31 March 2021). All the sequences used for tree construction are accessible in S3 File. MEGAX software (https://www.megasoftware.net/, accessed on 31 March 2021) was used for phylogenetic tree construction, applying maximum likelihood algorithms. To obtain a confidence level for each branch, bootstrap analysis was performed with 1000 iterations. All the phylogenetic trees are available in S4 File.

### 4.3. Protein Modeling

The Modeller (https://salilab.org/modeller/, accessed on 31 March 2021) software was applied to generate 3D protein structure models. The Modeller software generates the 3D structure of a given target protein sequence based on its alignment with a known protein structure (templates) [69].

The alignment file of *Arabidopsis thaliana* (Q9FL0) and *C. mollissima* protein sequences was obtained using the Emboss Needle online tool (https://www.ebi.ac.uk/Tools/psa/emboss_needle/, accessed on 31 March 2021). The 3D model was developed with an automodel class using the 3D *A. thaliana* model and the alignment file. Ccp4mg software (www.ccp4.ac.uk/, accessed on 31 March 2021) was used for protein 3D structure visualization, which is useful for studies of catalytic and regulatory domain conservation/divergence. 

### 4.4. Pathogens Inoculation and Samples Collection

*P. cinnamomi* (ID_C4) and *C. parasitica* (ID_5183 L2d) isolates used in the experiment were originally isolated from symptomatic *C. sativa* trees in Piedmont and Aosta Valley, northwestern Italy, respectively, and preserved in the plant pathogen culture collection at DISAFA (University of Turin). Isolates were subcultured in potato dextrose agar (PDA) before inoculations. The inoculation trial was carried out on *Castanea sativa* and *Castanea crenata* plants (1-year-old) grafted on *C. sativa* and *C. sativa* x *C. crenata* rootstocks, respectively. Plants were grown in pots under greenhouse conditions. The identity of the plant material was preliminary checked through marker analysis using 10 SSR loci from Marinoni et al. [70]. Plants were 80–100 cm high and 0.9–1.5 cm in diameter at the collar. 

Plants were inoculated 20 cm above the collar by placing a colonized plug of PDA (0.5 cm diameter) in a slit, superficially cleaned with 70% ethanol, obtained by excising a small portion of the bark with a sterile scalpel according to the methods described by Zampieri et al. [71]. After the inoculation process, the inoculation point was wrapped with parafilm to prevent tissue dryness and external contamination [71,72,73]. As negative controls (0 hpi), plants were wounded in the same way but inoculated with a sterile PDA plug. Plants were incubated in greenhouse conditions at 28 ± 2 °C with a 16-h photoperiod. 

S-genes are genes related to plant–pathogen interaction and are expressed during the first step of inoculation, before symptoms manifest. The time points used in our analysis were selected using the susceptible *C. sativa* as a reference on the basis of the onset of evident symptoms, i.e., bark necrosis and leaf dryness/browning, on *C. sativa* reference plants inoculated with each pathogens. The selected time points were 5 days after inoculation for *P. cinnamomi* and 7 days after inoculation for *C. parasitica*. Three biological replicates for seven experimental time points, including 0 hpi (control), were tested both for *Castanea sativa* and *Castanea crenata* and for the two pathogens (84 plants in total).

Plant material was harvested at 0, 3, 6, 12, 24, 48, 72 h after *P. cinnamomi* inoculation. For the *C. parasitica* experiment, material was collected at 0, 12, 24, 48, 72, 96, and 120 h after inoculation. For each time point, two disks of the stem were cut 0.5 cm above and below the wound. Bark was then removed to reduce the polyphenol contamination of RNA, and samples were frozen in liquid nitrogen to preserve RNA integrity. All samples were stored at −80 °C before RNA extraction.

### 4.5. RNA Extraction and Real-Time qPCR Quantification

RNA was extracted from both inoculated and control (0 hpi) samples. A total of 0.1 g of frozen tissue was manually ground into a fine powder and liquid nitrogen was added. RNA was extracted using Spectrum Plant Total RNA Kit (Sigma-Aldrich) following the manufacturer’s protocol. Extracted RNA was treated with DNase I (Thermo Fisher Scientific) following the manufacturer’s instructions. 

RNA was quantified by a NanoDrop spectrophotometer (Thermo Scientific, Hudson, NH, USA). cDNA was synthesized from 2 µg RNA using the High-Capacity cDNA Reverse Transcription Kit (Thermo Fisher Scientific, Hudson, NH, USA). Primer sequences for candidate S-genes were designed using the Primer3 online tool (https://primer3.ut.ee/, accessed on 31 March 2021) and are available in S5 File. All primers were in silico tested through Primer-BLAST program (https://www.ncbi.nlm.nih.gov/tools/primer-blast/, accessed on 31 March 2021).

*Chi3* and *gluB* genes, coding for several pathogenesis-related (PR) proteins, were also analyzed in order to observe their role in the defense response of chestnut trees [74]. Transcript abundance was quantified in three biological replicates by the StepOnePlus Real-Time PCR System (Applied Biosystems). Real-Time qPCR was performed using the Power SYBR^®®^ Green Master Mix added with bovine serum albumin (BSA) to reduce the action of PCR inhibitors. The amplification protocol included an initial denaturation step at 95 °C for 5 min, followed by 40 cycles of 95 °C/5 s and 60 °C/1 min. Data were quantified using the 2^−ΔΔCt^ method based on Ct values of candidate genes and actin (as a housekeeping gene) [38]. IBM SPSS statistical software was used to carry out a one-way analysis of variance test (ANOVA). Each value represented the mean of three biological replicates, which were compared using Tukey’s HSD test (*p* ≤ 0.05). 

### 4.6. Pathogen Quantification 

Samples inoculated with *P. cinnamomi* (24, 48, 72 h) and *C. parasitica* (72, 96, 120 h) were used for DNA extraction and pathogen quantification. Plants were debarked and the necrotic area in the cambium layer was measured using ImageJ v. 1.8.0 software. 

The DNA extraction was performed using an E.Z.N.A.^®®^ Stool DNA Kit following the manufacturer’s protocol. Standard curves were prepared for the quantification of DNAs by qPCR using primers designed as follows: *7-actin* for chestnut DNA, the *ypt* gene for *P. cinnamomi* DNA, and the *mf1* species-specific gene for *C. parasitica* DNA [75] (S5 File). All the inoculated and control (0 hpi) samples were analyzed through real-time qPCR both with pathogens genes (*ypt* and *mf1*) and with *7-actin*. The results, normalized by standard curves, were used for the calculation of the ratio of DNA fungus/plant DNA. Real-time qPCR was performed using the experimental conditions previously described: initial denaturation step at 95 °C for 5 min, followed by 40 cycles at 95 °C/5 s and 60 °C/1 min. Data were quantified through the 2^−ΔΔCt^ method based on Ct values of pathogen genes and *actin-7* as a housekeeping gene [38].

IBM SPSS statistical software was applied to perform a one-way analysis of variance test (ANOVA). Each value represented the mean of three biological replicates compared using Tukey’s HSD Test (*p* ≤ 0.05).

## Figures and Tables

**Table 1:** S-genes detected in the *C. mollissima* v1.1 genome and protein domain annotations.

Gene Name	Scaffold	ORF Length (bp)	N° Exons	Size (aa)	Domains	PFAM DOMAINS
*MLO1*	scaffold00101	1425	13	474	Mlo	PF03094
*DMR6*	scaffold02358	1128	4	375	2OG-FeII_Oxy; DIOX_N	PF03171;PF14226
*DND1*	scaffold00410	1407	6	468	cNMP_binding; Ion_trans	PF00027;PF00520
*PMR4*	scaffold00300	5346	1	1781	FKS1_dom1; Glucan_synthase	PF14288;PF02364

## Supplementary Materials

The following are available online at https://www.mdpi.com/article/10.3390/plants10050913/s1, S1 File: experiments pipeline, S2 File: S-gene coding sequences available in the NCBI database (https://www.ncbi.nlm.nih.gov/, accessed on 31 March 2021) used for S-gene detection in the *C. mollissima* genome, S3 File: S-gene coding sequences used for tree construction, S4 File: phylogenetic trees, S5 File: primer sequences.
