# Supplementary material for: Identification of Susceptibility Genes in Castanea sativa and Their Transcription Dynamics following Pathogen Infection
Source: Plants (Basel). 2021 May 2;10(5):913. doi: 10.3390/plants10050913 (PMC8147476; doi:10.3390/plants10050913)
Supplement: Supplementary file 1 [file plants-10-00913-s001.zip › S2 File.pdf]

#S genes coding sequences available in NCBI database (https://www.ncbi.nlm.nih.gov/) used for S genes detection in *C. mollissima* genome.

DMR6;

>NC_003076.8:8378759-8383401 *Arabidopsis thaliana* chromosome 5 sequence
TAAAAATCATTCGAATAATATGCATACTTATATAACAAAAACAATTCACTTGAAAACATAATCAATTGAG
AGTAGGACCGAGTAACACTGCATTGTTTTATATATATCATCGATGCACATCGCATACATAATATACTCAA
AGTCGAGCCTTCCTTCCTTTATCTCTTATACCCTTTTTGATTCTTCTTCAATTTTCTGACATCAAATGGC
GGCAAAGCTGATATCCACCGGTTTCCGTCATACTACTTTGCCGGAAAACTATGTCCGGCCAATCTCCGAC
CGTCCACGTCTCTCTGAAGTCTCTCAACTCGAAGATTTCCCTCTCATCGATCTCTCTTCCACTGATCGAT
CTTTTCTCATCCAACAAATCCACCAAGCTTGTGCCCGATTCGGATTTTTTCAGGTACCTAATTATAATTA
TATATACGTTTAGATCACGCATGAGCTTTTTGCTTCTATTAAAAAGATATGTGTGTTTTCTTTTGTTGTA
TTTGGTTAAAAAGTGGTACATAATTAGCATGAACCTTCTCTAATCAAATAGATTATATATAAAATTCTAC
AAAAAAAACAACAACTTTATGATGGTTGACTAGTTCTATGCTGACTATTATCATTCGGAACTTATATACA
AAGTGGTTAATTTTTTTTATTTTCGTGGTTAATCATTCGAGGTCATAAATCACGGAGTTAACAAACAAAT
AATAGATGAGATGGTGAGTGTTGCGCGTGAGTTCTTTAGCATGTCTATGGAAGAAAAAATGAAGCTATAT
TCAGACGATCCAACGAAGACAACAAGATTATCGACGAGCTTCAATGTGAAGAAAGAAGAAGTCAACAATT
GGAGAGACTATCTAAGACTCCATTGTTATCCTATCCACAAGTATGTCAATGAGTGGCCGTCAAACCCTCC
TTCTTTCAAGTAAGCACTTAAACTTACCTCTAATTTTCTTTTGATAAATTCTAATTTTACTTTACATTTT
CTTTCTCTAATACTTTCTAGTAACTATTCTATTGATTTTATAACGTTCCATAGTGAATAAAAACATATAC
TTGCATGTGCTTAAATAGGTAAAACGATCTATCAGGTTTATGGCATATCTCACGTCTTTAATATAATTGT
TGATTTTTGATGTGTCAGAATTGCCAAAAATAGGATATTATTAGCTATTTGTAGACTATCTTTACCGGAA
TAGATTTAGATTCTATTGTAAACCCATGTATATATATAATCATATTATACCCTAAATTATTAATAATACC
AAATGCTTTTAAAGCATCTAGGCTACCTATCAAAAATATATAAGGGTGAGGTATGTCTTCTTAACATTTT
CTGTTTGTCATGATTCCTATAAAGTTTTTGTTGAAATGTTTAATTGATTGATCAGAACTGAAAAAAATAT
GTACAAGAGTGGAGTGTTTCCTACGATGCTTCATTATAAAAACTAGAGCAAACATTCACAACATTATAAA
ATTCACAACAAAGCAAAACAAAACAAAACAAAACTGGAGCAAGAATTGCCTTTTCTCTTCAGATGTATAA
GCATCTATTGTCAACATTATAAAACATACGAGCATATGATCTTCGCACCAGCATATTTTCTATTTCTGTC
TTTTATCAACAACATATCTATGAAACTTTTAATTTCGATACAATATACCATATCACTATAACTATTTATT
GTGTTCTGTTTTTTTAATAACTAGAATATTGGAATATTCATATGAATATGTTCGATTCGATTTGTTTGAA
GGAAAATTCGACCTTCATTTGAGTCAACTGTTTTTGTTCTTTTTTGAAACCGACTTACAAAGAAAATAAC
ACAGATTGTGGTAAAGATAAGTTAACTTGTGGTCCCATTTGGTTTATATTTTCTGATTTGGCTCGCCATG
TGATCACATGGTTGCTAAGATTTATAAAATCTTTACATGCATACATCAATTAGTATACATGTACTTGTGG
CTATTGGACTTGGACATATATTTTTTATAAAGGTGTTATTGGGATAAAGAAGTGTGCACCATATTTTTTT
GATCTTTTATAAAACCGTGTGAACTACTACAACCGACTTGTTCGGTAGACAACAAACTGATTATAAACCA
GCAACGTGTTTTTGTTATATAGTACATGTATCATCTATTAGTAAATAAGTGAAGATAAAATATATAAACA
GAATTAATCATGAACAGTAAAAGATTCGATAAAGATGTGTTGGTGAAAAAAGACATGCAGAGATTAAAAC
AGAACCACACGATGAAAAAAGTATCATGAAAACTTCCAAACTCTTTAAACTGTGTCATTCCGACAGATCT
CCAACTGAACTGTTACAGAATTAAGAGTCAACACCGGCCAAAACCCGGCGACATCGTGCTTATCGAAAGG
TTAGTTAGGTAGAGGACAATTACCATAAATTAGGACAATAATAATGATGTGACAACTATTATTATATATG
ATTTTTGTCTCTTCAAGTTATTTAATTTTTGTCGTTTTATTGTTGCATAACGCAAAAAAAATTCGTATAC
CATTATTTTGTAGTGTTGAGCCGATTAATTCCTTAATATTTGAAGAACTTAGACATGGTTAAAATATCAC
AACGCGTCAAAAATATGATTTATAAGTAAAATGGTTAAATCATAACTTTATAAAATAGTATATGATATAT
GTGTGGTTATGGGGAAGAGACGTCACTGTACACCGCGTTGGCGTCAGCGTCGTGATATCATGCCAATCTC
TTAATTACATTTTTAAAAATTGTAAAAAAATTCATTTTCATTGACTCGGTCTTCAATTTTACAGATCGAT
GCTCTTATCTTATTTTATAATTTGTAATATATTCGCCACAAATTCTTCGGGTAAGTTCGAAGCTGACAAG
TGACAACGTGAGTTACGTGCTCCACTATTCCTTTAAAACTAACATAGCTAAGTATAATAATGCAGATTTC
TCGAGATATATGTGTTACATTATTAAATCTTAATTATATGATATACACTATTAGAATGACGTCTAAATAT
CATAAAAATTACAATAATCATAAGCTCTTAGAAAATAACTTTTCATTTTTAATGAAATATTTACATTCTT
AGCAAAAAAAAAGAAAAAAAAACTATATTAGTTATTGACAATCTAAACAATTTGAGTAGCTAATAAGTCT
TTGTCATGTTGTTGTTGCAGGGAAATAGTAAGTAAATACAGTAGAGAAGTAAGAGAAGTGGATTTAAAA

TAGAGGAATTAATATCAGAGAGCTTAGGTTTAGAAAAAGATTACATGAAGAAAGTGCTTGGTGAACAAGG
TCAACACATGGCAGTCAACTATTATCCTCCATGTCCTGAACCTGAGCTCACTTACGGTTTACCTGCTCAT
ACCGACCCAAACGCCCTAACCATTCTTCTTCAAGACACTACTGTTTGCGGTCTCCAGATCTTGATCGACG
GTCAGTGGTTCGCCGTTAATCCACATCCTGATGCTTTTGTCATCAACATAGGTGACCAGTTACAGGTACC
TTGATTACCTTTAATTCCTCCATTTGACTTGTGATATTAAAATTTCTAGTTTAAACCAAAAAAATACCAA
CCATTACTAAAAGAGTGGTGGGTTGGTTTCGACTTCAACTTTAGAAATAGATGGTTTATGTTGTATCAAC
CTGCCTAGTTTCAAACCATTTAAATAAATGTGATCCGGCCCATGAACTTCGGACTTAAAAAGAAAAGAAT
TCTAGTCTTTACGTAATAGTTTCTCTCTACTCAACTACCCCAATAAATTGAGATCTTTAAAACAATTACA
GAGAATTAGTTATAGGATTTCAAAAAGTACATCTAAAAATCTTAGGCAGGAAGAGTGTAAAATATTTTTA
AAATATTTATTTTTAAAAATGTATTCTGGACGTTTATTTATTTTAAAAAGTTCGGGTACTGTTAGAAGTT
AAAAGTTTTAACATTGATTAAAAATATGATTTTTTTCTTTTTCTTATAAGACATAAAGTGTTGTAAGAAA
TATCATCATTTAAACATTACTACAGTTATGAACATATTATAGATTTGTTTAGTTAATAAAGGGTTAGAGA
ATTTTCCGAGAATAATAGCCAATCATCAAGATCGAATGTTTGTCTAGAATTAAGATCTTAATTAACTGTG
TGTATTTTCAGGCATTAAGTAATGGAGTATACAAAAGTGTTTGGCATCGCGCTGTAACAAACACAGAAAA
TCCGAGACTATCGGTCGCATCGTTTCTGTGCCCAGCTGACTGTGCTGTCATGAGCCCGGCCAAGCCCTTG
TGGGAAGCTGAGGACGATGAAACGAAACCAGTCTACAAAGATTTCACTTATGCAGAGTATTACAAGAAGT
TTTGGAGTAGGAATCTGGACCAAGAACATTGCCTCGAGAATTTTCTAAACAACTAAGATACATATATCTT
TGGCCTTTGTGTTTGTCTAGTAGGCATATATATACAAGTCAATAACAGCATTGATGTTCGATTCTACATT
CCTACCAACATTTTGTTCTAGACGTATGATAATAGTAGGAATCATGATCATATGTCTTGATAATAACTAT
CATGGGCATATTTGTTTGTGTGTTAAATAATTTCTTACCTTTTATTTTTCTATATGCTTCAAAACTTTTA
ACTTTAGAAAATGTTTCGTATTT

>NW_017440543.1:26980-33841 *Juglans regia* cultivar Chandler unplaced genomic scaffold, wgs.5d, whole genome shotgun sequence
ATTTTGCCATGTTCGTGTATAAATAGAGTGGACACCAACTCATATTCCTCATAATACAAGCAGTTCGCGG
GTTTTGCTAGCTCGCAATTGCATTAATGGACACCACTAAGCTCCTGCTGACCGACCTCGCACCAACTGTG
AGCCATGTTCCCTCAAATTACATTCGACCCATTTCCGACCGGCCAAATCTCTCCGAGGTTCAGATATCTG
ATCAGGGCTTCATTCCTCTCATCGATCTCCGAGGCTTGGAGGGCCCCAATCGTAAGGATATTATCAAACA
GATTGGCCAGGCATGTCAACATGATGGTTTCTTTCAGGTACCCAGAATTATGTTTGTATAATCCATTGTT
TGATCATAAAAATTTATATTTTTCGTGATTTATGAACTTTTTTTCTTACGTTTTATTGCATGCAATGCGT
TGACCTCAGGTTAAAAATCATGGAATACCAGTGACAACTATTAGTAAAATCCTGGGCATAGCAAGAGAGT
TTTTCCGATTGCCAGAGAGCGAGAGGTTGAAGAATTACTCCGACGACCCTTCCAAAACCACCAGGCTTTC
CACTAGTTTCAACGTTAAGACAGAAAAAGTTTCCAACTGGAGAGATTTCTTAAGACTCCATTGCTATCCT
CTCGAGGACTATGTGCACGAATGGCCTTCATATCCTCCATCCTTTAGGTGGGTTAGAGCTAGGATTCATA
TCCTCCATTGCTATCCTCTGGAGTTTTCTGCTTAAATTGAAAGTTTTAAAGAGCAATTGACATAAAAAGT
GAAAAGTTAATCGATTATTTTGATCAAAAGTGGTTAATTGAGTTGTTTTTTGTGTAGGGAGGACGTGGCT
GAGTACTGCACCAGTATCAGAGGGTTAGTGCTAAGACTTCTCGAGGCCATATCCGAGAGCTTAGGCCTGC
AAAGAGACTACATTGAGAAGGCATTGGGGAAGCAAGCCCAACACATGGCATTGAATTACTATCCACCCTG
TCCGCAGCCAGAGCTTACTTATGGTTTGCCTGGCCATACTGATCCTAATTTAATAACAATTCTTCTTCAG
GATGATGTTCCTGGATTGCAGGTTTTAAGAAATGGCAAATGGATTGCTGTCAATCCTATTCCAAATACCT
TCATTATCAACATTGGAGACCAAATGCAGGTAGAAGAATTAGTATGTTTTCTATGAGAAATAAAGTCATG
ATTCCTAAAGTTGCATAAGATTCTAAGGTTTCCTTTTGTAATCAGGTGATTAGCAATGATCAACACAAGA
GCGTGCTGCATCGAGCCGTTGTGAACTGCAACAAGGAGAGAATATCCATTCCAACATTCTACTGTCCATC
TCCAGCTGCTGTGATTGGACCTGCTAAGGAGCTGATCAACGATGATCAGCCAGCAGTGTATAAAAACTTT
ACCTATGGGGAGTACTATGAGAAGTTTTGGAACAGAGGCCTTGCAACTGAATGCTGTTTGGACATGTTCA
GAGTGTCTAGCTAGTGCTTGAGCTATTATAATGCCTCTGAGATGCTGCTAAGCAACGTGTTATGAAGCCC
ACGAAGATGGGCCAAGAAGGAAGGAGTGGGCTGAAATTGGAAGAGAACATGGGCCAACGAATCTGAAGAA
ATGAAGAAATAAAACACAGTGTTTTGCGTAGTAGTCGATTGTTATGCATTTTATCATTCACTAGTTTACC
ATTTTATTTACAGTTGTAGATGGACACGTGTAAGGCATCTGTTAGTAAAGTTAGTTACTGGCAGAGAATA
TAGGGGAGGTAAAAAACGGGTGTGAGTTTTAACGAATATTTTGAACAATTTTCCTGAGGGAATTTATCTG
GAGGAATGTGTGCCTCGAACAGAACTGTGATTCTACTTTTATCAACTTTCCATTTCTATCATCTTCTCCA
ATGCTCATCTTTTCAAATATCCATTAATATACAGTCCAATCCATTATCTCCTACTACTACAAGCTACATT
ATAGTGGTATCAAGTCTCCGATTCTGTACCAAAAATTAGCCACACATTACCATCGCCCATTCAAATTACC
ATCATTTCTGCATACAACCATTTATGACTGACAATACTAGATTCAAACATCAAGATGTGCTGCAACGGCA

GGAAGCTCAAGAAACTCGAATGCAAGCCCAAGAGGAGAGGATGCATGTAATGAGTGCATACATAGCTCAA
CTAACTCAAATGGTCAAGATATTAGTCACAAACCAGACTGCACAAGTGGTTCACCGTGACACACATAACC
AGCATGACAGAGAGTTCTAGGACTTCAGGTGCGAAGGACAGAGGGGGGGTCAAATCCAGAAACTGGATTT
TCCTTACTTTGATGGCCCCAATCCAACAGGTTGGATTTTCAAAACTTCACACTATTTTGAATACCATCAA
ACACCTCCTGCACGAAGATTGTTAATGACTTCATATCACATGGAAGTGGATGCATTAATATGGTATCAAA
ATGCAGCAGAAACAACATAGTTTAATAATTGGGAAACTTTTTCTATAGCCTTATTGCTTAAGTTTGGACC
CACAGCCTATGACGACCCCATGGAAGCCCTCACCCTCTTTAAACAAGTGTCTACTGTTGTCACCTATAAG
GATCAATTTGAAGCCCTCTCAAATAGACTTAAAGGACTCATTGAACATACAAACTGATTATTTTCTCAAT
GGGTTGAAGGATGAAATTCGTCTTCTCGTTCGAATGTTCAACCCCTTAAGCCTAAATGTTGCCTTTGGGC
TTGCCAAAATACAAGAGGAGTACATGGCTAGTTCAAAAAAATTAGTGAGAAGTTGGATGGACAAAAACAC
CACTCCAGCCAACTCGGGGTCAGGCTTTAGCCACTTTCCTAATCGATTTCAAGGAGCTGGACAGAGACCT
TTCATGCCAATGAGAGAAATCCCCTAATCTCAAATGGATGAAAAAATGAAGAAGGGCCTATGCTACCATT
GTGAAGAAAAATGGAACCCTAATCACTTGTGCAAAGCCCCAAAGGTGTATGTATTGCAAGGATGTGATGA
AGAAAGGGAAGATAAAGGTGAGGAAGCTTATTTTGATTCACATGAGATGCAAGAAGGCCAACAAGGAGAG
AATAAAGTAGAAATATCCCTCAATGCAATCACAGGAACTCCCAATCCTAATACCATGCGAATTTGTGGCT
CTATTGGTGAGGAGATGGTGCTGTTGTTGGTGGATTCAAGAACCACACACAACTTCCTGAATCCTTCTAT
TGCAAGGAAGGCAAAGCTGCAAATGGATACCACTCAAAAGCTCAAGGTGCACGTGGCCAATGACGAGTTA
GTCACAAGTGAAGAGCCATGTACATCTACATCACTCAGATTACAAGGTAACCAGTTTACTACTTCCTTTT
GCTTGTTAACTTTGGGTGGTTGTGATGTTGTGTTAGGCATACAATGGTTGGAAATTTTGGGAAATATAAC
TTGGAACTTTTCCAAGTTAGTTATGCAATTTCTGTGGCAAAACAGATTGGTGGAGTTGAAGGGTCTTCAT
TTGGGTAGACCATCATTTGGGGAAGGAAAGGGAGTAATGTTAAATAGTATGAATGGGCCCAAATGAGTCT
TTATCCAATGTTCTACTGTACACCCCACAACCACCACCAACCTAGACCCAAACACAACTCCAAACAGCCA
GCTAATCATGTTATTACAGCGGTTTAAAAATGTTTTTGCAGAACCTATGGGCTTACCACCCAAGAGAACC
CATGACTACAAAATCGCACTAAAAGATGGAACCCAACCTATCTCCACAAGACCATACCGATACCCATTCT
ACCAAAAAACTAAGATAGAAAAAATTGTCACTGACTTACTCAAGTCAGGGATGATTAGACCCACTTCTAG
CCCTTTTTCCTCACCAGTTTTACTAGTCCGAAAGGCTTATAGAAGTTGATGCCTATGTGTAGACTATAGG
GCCCTAAATCAAGAGACGGTGAAGGATAAGTTTCCTATCCCCGTCATTGATGAGTTGCTGGATGAGTTTT
ATGGCTCTGTGGTTTTTTCTAAAATGGATCTAAGGTCTAGGTACCATCAAATCAAAGTGGTTCCTGAGGA
TGTTCACAAAACTGCCTTTGTCACCCATGAAGGGCACCACGAGTTTTTGGTGATGCCCTTTGGGCTCACC
AACGCCCTCTCCACCTTCCAAGGATTAATGAATGAACTTTTTAGACCATTTTTGAGGAGGTTTGTGCTGT
TTTTTTTTTACGACATCTTGGTGCATAGCCAAAGTGTGGAGGATCATGTGGGGCATGTGAGACAAGTGTA
GATTGGACGTTCTAGCTAAAAATAAGCTCTATGCTAAACTGTCCAAGTGCAGTTTTGGGATTCAGGAAGT
GGAATACTTAGGACACATGGTGTCAAGTGAAGGGGTTAAAGCTGACCCAAACAAGGTAGCTTCAATGATA
GAGTGGCCTATTCCTACCAACTTAAAGGCCTTGAGGGGCTTTTTGGGCCTAACGGGGTATTACCGTAAGT
TTATAAGGCACTATGGGCTTATAACTGACCCTTTGACAACCCTTTTAAAAAAGAATGCATTCACTTGGAA
TGCCACAGCTCAGCAAGCTTTTCAGCAATTGAAGGGGGCTGTATAGGAGCAGTATTGAGTGCAATGCGAG
TGGGTGTGGTATAGGAGCAGTACTCATACAGGCTGGACAGCCCAACTCCTATTTCAGTAAGGTCTTGAAA
GGCAAGGCTTTGGCCCTATCCACTTACGAGAGAGAGTTATTGGCTCTTGTCATGGCTGTACAAAGATGGA
GGCCCTACTTATTAGGTCAATCATTCATTGTAAAAACTGACCAGCAGGCTTTAAAATACTTACTAGAATA
GCCCGCCTTTAACTAAGAGTTATTGGCAGTCCAAGATAGAGCTACCTAAGAGTTATACAGTCTAGCAGGG
GCTGGTCTTAAAGAAGGGGAGGTTTGTGATCAACCCTAGCTCCCTTTTTAAAGCCAAAGTATTGCAGTTT
ATACACAGGAACCCAGAGGCAGGCCATTCGGGGTTTCTAAAAATGTATCAAAGAGCAAAGAAGAATTTTT
TTATGGCATGAGATGAAGAAGGATATTAAGGAGCTTATAAGAAACTGTGAAGTATGCCAAGTCAACAAAC
ATGAAAACCTCTACCCAGTGGGACTTTTGCAACCTTTTCCCATTCCAAACCAAGCTTGGGAAGAGATATC
TATAGACTTTGTGGAGGGTCTTCCTACTTCTCAAGGGGTTAATTTGATCCTACTTGTTGTGGATAGGCTG
ACTAAGTATGGCCATTTCATGGCTGTGGCACACCCCTATACAGATGTCGGAGTAATTCAGATTTTCTTAA
GAGAAGTATTTAAATTACATGGATTTCCTAAGATAATTCTCTCAGACAAGGATCCCATTTTTCTTAGTAC
CTTCTGGAAAACCTTGTTATCACTGCAAGGTACTTCTCTAAGCTATAGCTCAGCTTATCACCTGCAGAGT
GACGGGCAAACAAAGGTCCTTAACAAGGCATTAGAAGGTTACCTAAGGTGTTTTGTGGGCTTTAAACCAA
GGCAGTGGTCTCAATGGCTTCCCCTTGCCGAATGGTGGTAAAACACATCGTACCACACATCGGCAAAGAT
GCCACCTTTTGACGCCCTCTATAGCTACCCTTCAGCACGGCTCGTTACATACATCCTAGGAACATCACAA
AACGACGCAACAGATAAAATGTTGACTGACAGAGAATAGATCAAGCGCCTACTCAAAGAAAATCTCAACT
TTTCACAACAGAGAATGAATAAATTTGCATATCAGAGTAGAACAGAGAGGGAGTTTCAAATAGGAGATTG
GGTATACCTCCGTTTACAATCATATCGGCAGAAGATAGTGGTTGCAAGGCAAAACTTGAAACTTTCTCCT

AAGTTTTACGGACCTTTCAAGGTGGTGCAGCGCCTTGGTGCAGTGGCCTACCGCCTGAAGTTGACACTGG
AGTCCAAAATCCACTCAGTCTTCCACATTTCGTTTCTTAAGCAAAAACTGGGCACTCAGATCCAAGCTAT
GCCGTCGCTCCCTCCAATCAACAACGCCGGTGAGGCACAGCCCAAACCCGAGATGGTGGTAGAACATCGT
ATGCGTCGACACGGAGACAAAGCCCAGACTATGGTCTTGGTGAAATGGGTGGGACTTTCGGAGGAAGAGA
ACTCTTGGGAGTCTCTAAGGAGGCTTCAAGAATGTTATCCCCACCTGGTGGGCCAGGTTCTCTAAAGGGG
ATGGATTTGTTATGAAGCCCACAAAGATGGGCCAAGAAGGAAGGAGTGGGCTGAAATTGGAAGAGAACAT
GGGCCAACGAATCTGAAGAAATGAAGAAATAAAACACAGTGTTTTGCATAGTAGTCGATTGTTATGCATT
TTATCATTCACTAGTTTACCATTTTATTTACATCTGTAGATGGACACGTGTAAGGCATCTGTTAGTAAAG
TTAGTTACTGGCAGAGAATATAGGGGAGGTAAAAAACGGGTGTGAGTTTTAACGAATATTTTGAACAATT
TTCCTGAGGGAATTTATCTGGAGGAAGGTGTGCCTCGAACAGAACTGTGATTCTACTTTTATCAACTTTC
CATTTCTATCATCTTCTCCAATGCTCATCTTTTCCAATATCCATTAATATACAGTCCAATTCATTATCTC
CTACTACTACAAGCTACGTCACAACATGTGTTGTTCTGGGCGCAGACATGTTTCTTGACATGTTCAATCA
TTGTCGATCGAGCAATAAAAATCGATGTAAAGTTGATCTTCGAACAATTCTGAACTGCCTTTTTTATGAT
GTAATCACAGTATTCCTCCATATTGCTCCTTTATAACTAATGAATGGTATTAATAAAATTAGGTATTTTT
TT

>NW_019814625.1:747958-748693 *Quercus suber* isolate HL8 unplaced genomic scaffold, CorkOak1.0 scaffold_9122, whole genome shotgun sequence
CAAAACTAGTAAGGAATACTCAAAAATCAAGCCACAAGAACCGTGCTAGCTGTTGCCTGTTGCAATATGG
ATTCCAAACTGGAACAGCCACAGCCACAGCCACTATTATCCGACCTTGTCTCCAGCATGAGCTCTGTTCC
CTCTAACTACATCCGATCCGCCAATGATCGTCCAAATTTTCATGAGGTTGTGTCATTTGATGGTGCAATT
CCTCTCATTGATCTTCAAGACCTCAATGGTCCTAAGCGCTTAGATATAATCAAAGAGATAGGCCTTGCCT
GCCTAAATTATGGCTTCTTTCAGGTATTTTCTCTCTCACACATAAACATATTGCTACTGAACTAGAATTA
GTGTATTTTCTTTCAATGAATATATATCACATCATTTTTTCTCTTTTCAACATCGTTCCAAGGTTAGCAA
CCACGGAATTTCAGAAGCAGTGATCGATAACATGTTAGATATATCAAGGGAATTTTTCCACCTGCCGGAA
AGTGAAAGGTTAAAGAATTACTCTGATGATCCAATGAAGACAATGAGACTCTCGACTAGTTTCAATGTAA
GAACTGAAAACGTATCCAGCTGGAGGGATTACTTAAGACTCCATTGCCACCCTCTAGAGGACTACATCCA
GGAATGGCCTACCAACCCTCCATCCTTCAGGTATTACCAAAAAAAAAAAACCAACTCCTCTGATAAAGAC
ACGACTGAGGAGTGGTTCAATATGTTTGTAACATGA

>NW_017442669.1:c26778-24333 *Juglans regia* cultivar Chandler unplaced genomic scaffold, wgs.5d, whole genome shotgun sequence
TGAAGTCTCTGGAGTCTGGACTGGAGCATGCATGCCATGGTGGTGGGTTTTCGTTGTCCCTACCACCAGC
TCGTGGTCTGACTGATTTTTTGTCAACGGGTCGTTAAATGGAAGGAGTCCTTGAAATTCACGACCGATGG
AACCCGAAGCTGGTTTCTCTGAAACTTGATACTATTCTTCCCCGCTCGCTCCTCTGCTATATATACTCCA
AAACCTTATCAGTATACCCGCACTTCATTTCATTCTTAGTCTCCGCATATCCCTAGCTATTATCTTTTCA
AGGCCTCACGTTTGGTATATATATTTATCCAATCAATACTCTCTCTCTCTAGATCTCTCTCTCTCTCTCT
CTCTCTCTCATACACACACACAGCGTTTCTGGCCAAAAAGAAAATACATGTATTCAAAGCTTATATAATT
AATAGAACACAATTTTCCTTAACCCTGCATGAATTCCAGGTTATTGTTCCATGTCTCCTACGATGGCTGT
AACAACTGAAACAAAGGAGGAGAATGATACGCCAGATAGCGAGTACCAAAAAGGAATAAAGCACCTCTGG
GAAAATGGCATAAACAGAGTTCCCAAGAAGTACATATTACCCCCCTGCGATCGACCCATTACTGAAGACG
GGGTGCTAAATCATGTATCCGAGCAAAATCTTAAGCTGCCCATCATTGATTTCGCAGAACTAATGCAAGG
TGCCAACCGGCCTCAAGTCCTCGAGTCCCTCGCTAATGCTTGCGAACAATACGGTTTTTTTCAGGTATGG
TTTTGAATTGTTTATAAATTACCATGTTGAAACCAAATTGTAATTCGATCTAGTCATCTCAGTCGACCAC
ACTGATCCATGTCAATGTTCTATTAAGACAAAAATCGAAGAAAATATAGGAATTTTCAAGATTAATGCTT
ACTTCAAATTAAATTTAAATTATAAATTATAAATATTGCGCGCAGCTGGTAAACCATGGCATTCCAAGCG
ATGTTATAAGCAGCATGATTGACGTTTGTACAAGGTTTTTCGAGCTGCCATTTGAGGAAAGATCAAAGTA
CATGTCTTCGGATATGCAAGCGCCGGTCCGATATGGAACCAGCTTTAACCAGAAGAAAGATAATGTGTTT
TGTTGGAGAGACTTCTTGAAGCTAATGTGCCATCCCCTATCAGATGTCCTCCCACATTGGCCTTCTTCTC
CTATGGACCTGAGGTGAGAGTTTACCTACCTTAGCTTCAGCCTTCAATTATTTTTATTTTATTTTATTTT
ATTTTTTGCAGTCCTTTCTCTCTTATATATATATATTTATATAAATATATATATATATATATATATCTCT
TTCTTTCATTTGTGTGGCTCATAATCGCTAGCTAACCTGTACACGTACGGCTTTGCGGGAAACGAATAGG
GGTTGACTTTTGCAAATGAATTACTACGACTCCTATTACTCATGAAGTTCTTAGATATAAAGAATAATTA
ACTTGCTCGTAGTTGTCAAGCAATGATATCTATTACATGCATGTATATGTAAATATTAATAATTTGAGGA

CCAATCAAGTTGATGGTTCATGCTTTAAACAGGAAATTGGCGGCTACCTACGCAACAGAAACCAAATACT
TGTTTCTAATGCTAATGGAGGCCATCGTGGAGAGCTTAGGACTTGTGGGAATCGCGGATGAGAAGAAGAA
GACAGAGGAAGAAGAAGAAGAAGATGACATATTAAAAGACTTACAAGAAGGAAGCCAGTTAATGGTTGCC
AATTGCTACCCGCCATGTCCAGAACCCAATTTAACCCTGGGAATGCCACCGCATTCCGACTATGGATTCC
TCACACTTCTTCTCCAAGATGAGGTTGAGGGCCTACAAATACAATTCCAAGAAAAATGGATTACTGTCCA
ACCAATTGCTAATTCATTTGTTGTCAACATTGGTGATCATCTAGAGGTGGGTCGTAAGTACATCAAAGTG
CACGAAATCCAAAAAATATATATAATTAATTCAAGTAGTTTGTAATTAACTGTTGATTCAATTATATTTT
AATTTGTCTCAGATATTTAGCAACGGAAAATACAAGAGCGTTTTACATAGAGTATCTGTGAACCCTATGA
AACCTCGAATATCGGTGGCTTCTTTGCATAGCCTTCCTTTCCAGAGCATGGTTAGGCCGTCGCCTAAACT
GATCAACGAAGCGAATCCAAGGCGTTTCAAGGACACCGACTTTGCTAGTTTTCTCGACTACATTTCATCT
TCTGAGCCCAAGAGGAAGAATTTCTTGGATTCTAGGAAATTATTGGATTAAGGCTATATATATATCTAGC
TAGCTCGGAGAAGTACCGATGAATTAATCCAGATCTATATATCAAAACGGATTAGACGCTTGTATGTTGA
GTACTTTCGTTGCAATCAATTATGTATCATTGATTACATGCATGGAATTAATGTATTTTTATTCTC

>NW_017443584.1:c399769-397391 *Juglans regia* cultivar Chandler unplaced genomic scaffold, wgs.5d, whole genome shotgun sequence
CAGCACTCATTTTTCCACATCTGATCTCAATCACGTCTAACCATTTCCTACTCAATTTTCCAATCATCCT
CTCAACCATTTTCGATCAGTTTCCCATGACGACATGCAAGAAACTAGCCGAATCACCTGGCCTCACCTCT
ATTCCTTCCCTTTACACTTACAACACAAATCCCAAAGATGAAGCAATTTCAGAAGACCCGGAAGACCCAA
TCCCCGTCATTGATTTATCTTTACTCACCTCAGGCACTCCTCATCAACAGTCTCAAGCCATCCAAGAGCT
TGGCAAGGCCTGCAAGGACTGGGGCTTCTTCCTGGTAAAATATCAATCAATTTTTAACTATTTCTTTTCT
TTACCAGTACTGTAGATACATGTGTAGTTGGCGCATGCGTGCTCATAAGTTTGAAAACTTGTCTCAGTTG
ATCAACCATGGCGTACCGGAGAGGCAAATGGAGGCAGTGATAGAGGGTATTAGAGGTTTCTTCGATCTGA
CAGAGGAGGAAAAGAGAGAATTCGAGGGAAAAAACTTCTTGGACCCGATCAGGTGCGGAACCAGCTTCAA
TACATCATTGGATAATGTTTTCTTCTGGAGGGATGTTCTCAAAATCCTTTTAAATCCTGATCATGAGTTT
CAGTTCCCCTACAAACCTGCTGGATTCAGGTATTTAATTTATATATATTGGTTTAGCAATTAGCTACTTC
ATGTGCTTGATTCTTCATTATAGCTGATAATACAATGCTAGTGATCTGTCTAGATCTTCAAAGGTGGATC
ATAAAGTTTCTGTGATCACCTTGACCTAAACAAATAAATTATTTATGCTTATCATCTATACACCACAAAT
TTAATAAAGAAAAAAAATCATGCAGAGTGTGTGGTGTAAAGACCATGAAATCGTCTTGTTTCTTTCCTGA
CTTGTCGGTTTCGAAAGTTAGAAACAGTTGTCTACACGTCCAAGTACTGCTGAGCATGCAATAATAGACT
ACGTACTCATGTTGAGTGAGTATGTCCTACGTACTAGGTATCATTAATATCGGCCGATGCGATACAATGA
TGTTCCTATATATTAGTTGACATATTAATCCTGAATGATAAAATTAGATAAAGAATAATTAATATTAATT
TCTGTAAAATGAATTCTAATATTAGTTAATTTGTTGCTGTTGTTGCAGGGAGGTTTCAATGGATTACTGC
AAAAGAATCCGGGAAGTGGCAAGGGAATTACTCAAAGCAATATCAATGAGCTTGGGATTGGAACCCTACT
ACATCGAAAAGGCCACGAATCTGGAATCGGGTTTACAACTCTTCGTTGCAAACCTTTACCCTCCCTGTCC
ACAGCCAGAACTTGCGATGGGTTTGCCACCACATTCGGACCACGGCCTCTTGACTCTTCTCATGGAGAAT
GGAATTGGTGGCCTCCAAACACAGCACAATGGGAATTGGGTCAATGTCAATGCCATTCCCAACTCCTTTC
TAGTCAATATTGCCGATCAGCTTGAGGTTTAAAGCTTCTTCATCTTAATTTCATCATTCATAATCAGCAA
GTACTGATACCTCATAATCACTTAAATGGAAAAGATAATAAAATTTAAGATGAAAAATACCATTTTTTCT
TAATCGAATATATATATATATACTGCCGTATATTCTAATTTCTACACAATGATGTTTTATTTTTGTATTA
GATTTTGAGTAATGGGAAGTACAAGAGTAACGTGCATCGAGCAGTGGTGAACAGCAAAGCTACGAGGATA
TCACTCGGCATTGGGAACGGGCCTTCATTGGAGACGATGATCAGGCCAGCACCTGAGTTGGAAAGTAAGG
AGCAGGCAGCAGCATACATTGGGATGAAATACAAAGACTACATGGAACTTCAACAAAGCAACCAACTTGA
TGGGAAATCCTGCTTGGATCGTGTTCGAGCTTTCAATTAATTAATGAATGTTGTCGTATTGTAATTAGTT
ATTAAAATATAAAATAAATAATAAAAACTATTGTCGAGCTCAGATGCAGGCATGTAATCTAATTGAAAAA
AAAATTGGACAATGATATGAAATAACGAGATGAGATGAAAATTGAATAAAATATTAAAATAATAATAATA
ATAAAATTTATATTTTTTTGTTAGTTTTAATTTTTGAGATAAATGATGATTTCACATAGTATTAGAGTAG
AAGTCTTGAGTTCGAATCCTGACTCTACACTATATCTCATTTAATTAAATATTTCATATGTTGGGCCCAC
CCATTAATAGAGAGTCTGACCTACAAGTAAATGAAGTATTAAGATATAAAATAAATAATAAAATTTATAT
ATTTCCATCAATTTAAGCTTTTGAAACAAATGAAGATTTTACGTTAGTTAATCAAGACACTAGTTGTTC

>NW_017443584.1:c423227-422189 *Juglans regia* cultivar Chandler unplaced genomic scaffold, wgs.5d, whole genome shotgun sequence
ATGTGGAAGGCGATAAACAATGCACTAAGTGTGGATAATAAGTTGAAATCTGTTGGGATCCCGATAGTGT

CAAAATGTGATTATTGTTTGCAGGATGGGTACGAGGACTTGAATCACGTGCTTGCAGCATGCGAGTTTGC
AAAAAAAGGTCTGGCGGCTGTTCAGATGAGTTTAGGTGATGTTGAAGGATCCTCATGGTATCAATTGATA
AATTCCTGGTTTACTAATGCGTCGAGGAACTTCAAAATGGGGCAGGTATTAGGACTTTTGCCGAGCCCTG
CTACATCGAAAAGGCCACGAATCTGGAATCGGGTTTATAAGTGCAAACCTTTACCCTCCCTGTCCACAGC
CAGAACTTGCGATGGGCTTGCCACCACATTCGGACCACGGCCTCTTGACTATTCTCATGCAGAATGGAAT
TGGTGGCCTCCAAACACGGCACAATGGGAATTGGGTCAATGTCAATGCCATTCCCAACTCCTATCTAGTC
AATACTCAATACTGCCGATCAGCTCCAGGTTTAAAGCTTCTTTGTTCTTAATTTCATCATTCATAATCAG
CAAGTACCGATACCTCATAATCACTTGAATGGAAAAGATAATAAAATTTAAGATGAAAAATACCATTTTT
TCTTAATCGAATACTGCCGTATATTCTAATTTCTACACAATGATGTTTTATTTTTGTTTTAGATTTTGAG
TAATGGGAAGTACAAGAGTAACGTGCATCGAGCGGTGGTGAACAGCAAAGCTACGAGGATATCACTCGGC
ATTGCGAACGGGCCTTCGTTGGAGACGATAATCAGGCCAGCACCTGAGTTGGAAAGCAAGGAGCAGGCAG
CAACATACATTGGGATGAAATGCAAAGACTACATGGAACTTCAACAAGGCAACCAACTTGATGGGAAATC
CTGCTTGGATCGTGTTCGAGCTTTCAATTAACTAAAGAATGTTGTCGTACTGTAATTAGTTAATCAAGAC
GCTTGCTGTTCATGATGATCGATACTCAAGTATTTATCTTTTCAAGTGCTTTCGAATAA

>NC_044911.1:53017492-53019424 *Quercus lobata* isolate SW786 chromosome 8, ValleyOak3.0 Primary Assembly, whole genome shotgun sequence
CTCTCTCTCTCTCTCTCATAGTAGTTACTTTGTGATCCCAAGCTTTATATTTTGTGGTTTCCAAATTAGT
ACATTCTAGTAAGTCATTTATTTCTTGAAATTATTTAAACTCTACAAGCACCTAGATATTCTTAAAATTT
TGATCATTACAAATTTTCTCTTTAAAGATATATAGTAGCTTTAATATCACCAGGAATGAGTTTGGCAGGT
GAAAATGAGTCATTAGAAAGAGAATTCCAAAAAGGAGTGAAACATTTATGTGAGAGAGGAATAAAAAGTG
TTCCAAGCAAGTACATATTGCCAGTCCTTGACCAACCCAATTCAGGGAAAGGAATTTCTAGTGCTAGTAA
CTCCAATCTAAAGTTACCCGTTATTGATTTTGCAAAGTTGCAAGGCTCAAATAGATCCCATACTGTCAAC
TCCCTCAGAAAAGCTTGCGAAGAATTTGGTTTCTTCCAGGTATAGTCTTTAAGTTTTCCTCAAATATATC
TTCAAGGCCTTAAAGACATGCATGCATGCCACAATGAAATATGCTTTTCTTTAACTTCTTATTCGATTTG
ATGCAGTTGGTAAACCATGACATTCCAAGGGATGTGATTGAAAACATGGTTGATGTAAGTAAAAGGTTCT
TTGAGCTCCCATTTGAGGAGAGATCAAAGTACATGTTAACTGATATGAATTCAGCAGTCAGGTATGGGAC
AAGCTTCAACCAGAATAAAGATGCTGTCTTTTGTTGGAGAGACTTCTTAAAGCTAAGCTGCCATCACTTG
TCCGATGTTCTTCCTCTTTGGCCCTCTTCTCCTATGGACCTAAGGTGATCATCATAATTGTACTCTTATT
TGCTTTCCTCATCCACAAACACATGCACCAGTTGGTTACTAGCTTAGGTACTTATTTTATTTGCTAATTG
CAAAAGTCAATTGTGTTTATTATTATTAACATATATACCTTTTCAATATTTGAATAGGCAAGCTGTGATT
AACTATTCGAACAATACTAAATTCTTGTATCAAATGCTAACGGAGGCCATCCTAGAGAGCCTAGGATTGG
TGGATACTAATAACAATGAGAAAGAAAATGATGAATTAGAAGAATTCAAAGATGGAAGCCATCTCATTGT
GATAAATTGCTATCCAGCGTGCCCTGAACCTGATTTGACACTAGGTATGCCACCCCATTCGGACTATGGC
CTACTCACACTTCTTCTCCAAGATGAAGTTGAGGGTCTTCAAATACAACATGAAGGAAGATGGGCGACAG
TCGAACCACTTCCTGATTCATTGGTTGTCAACATCGGTGACCATCTTGAGGTTGGTAAATATTAATCAAA
TTATTCAAATCCGTGTAGGAATTTTTTTAAAAAAAATTATGAAATTCAGTAAGTAAGGAATTTCATGCT
CTATATATCTTTTGCAGGAACTTTAAAAATTAATAATAACTCCTTTATTTACTAATAATCATTCACCATA
TAAGTAGTTTGTAAAAAAATTTATAAAATAATTTTCAACTCTAGTATTTTTCTTTAGATAATGATAATAG
CTAATCAATCTTCATCTTTTTTCTTTTTTTTTTACATTGTTTGACAGATATTTAGCAATGGAATATACAA
GAGTGTACTTCATAGGGTTCTTGTCAATCCTTCAAAGTCACGAATTTCCATTGCTTCATTGCATAGCCTG
CCATTCAACAACGTAGTTCAGCCGTCACCGAAACTCATCGATGAAGACAATCCAAGGCATTACAAGGATA
CAGACTTTGCCACTTTTCTTGACTATAATTCTTCCTGTGAACCCAAGAAAAAGAGTTTCCTGGAGTCCAG
GAAACTGACTTGAGGGGGGACGTGTCTACGCAGTTTGATTTTTCCAGCATCGACCGGTGTGTAGCAAAGA
TTATCAGGATGCGAGAAATTTCTAGATAACTGACTTTTGAATA

>NC_044914.1:23670361-23685892 *Quercus lobata* isolate SW786 chromosome 11, ValleyOak3.0 Primary Assembly, whole genome shotgun sequence
AACCCATATTTTCTCCAGTCAATTAGCTTTTCACTTTTCAGCTTTATCATTTTGTATATTCTCTCTTTCC
GATTCCCATTTCTCATTAGGATCTTTTCAGCAAAAGCAAAAATTCTTCATCAGGATCTCTCTCTATTTTT
TACTTCCTTCTTCACCACTTATTAAAGCCCCCAGCCCAATTATTCCACGAACAACCCGCCACTGCCTTGA
TTCATTTTATCTCTTTCTTGCATTGAAGAAAAAAAGAAGAAGAAAGGGAAACGAATTCAGAGCAGCTGCA
CAGCGAGATTTCTCAAGTTTTTTGCGAAGAAAGGTGCGACTGTGAAGCGTAAGTGTATACTGTTTGTACT
TTGTACCGTGTAAGAGTTATTATCTCTTTTTCTTTCTTTTTTTTTCTTTTTTTTATTACCAATACAAATT

TAGAAGAAGTAATGTTAGTAGTGAGTTCCGTTAAGAATCAATAAGAAGTTCTAGTTAGAACACTTTTAGC
GGTGGTAAAAAGCTAAAAACTTATTTTTAGCACAACTAAAAAGCTATTTGGAATTGCAGCAGGGGCGGAG
CCACCTTATACTGTGGGGGGGGGCAAAAATGTTCAAAATTTTTTTTATAGTTGGTAAAAATAGGCCCCAA
AGAATTTTTGTCAGATTTGGCCCCTTCAAATGAAACCAATTGGCCCCCTCAAGTCTCCTTGATTCATTCA
AATTTATTCAATTATGTTGACCTTTTGTTAGTCTTTCTCCATCTCTCCTCCCCCAACACGTCATCAGACA
AGCCCAACTTGTATACTTTTTACTATTAGGCTGTAACTTCCTTTATAGAAGGTTATTACTTCACCTTTTC
AATTTGTATATTCCATCTACCTCATCACTCACAGTGAAAGTGTTCCTTAATCTCTCTAGTCCTCCATTTC
TTCTTCTCCTCATCATTTCTATTTTACATCAAATTCTATTCATTTTTATCTAAGTTCAATTTCTATTTCT
ATTTATTTATATTATGGTTGATATTTTTTTTTTCTCCTTCTTATTTATTAATCCAATTGATAGTCTAATT
CATTATTTATATTTAGTATTTCATACTAACATACTAAGACAACCATATTTAAGTAAGCTTTGGTATGACA
AACACATCTACTACTAGATTAGATGCATAAATCTTCATAGTCCAATATATGGTACCACTAATTTTATTTG
AAAAATCTCATACGCATGCCAAAACTAACAATTTTGTCTCTGTTTGACAAATTTGTCCAGTAATGAGTGG
GTCATACACAAATACATAGATACACACACATATCTCTTGTTAATCAATATGATCATTTAGTCTTGGCTGG
CCCCCCCGAAGGAAAATTTCTAGCTCCGCCCCTGGCAGTGGAGTTATAGTCAAAAAATAATTTGGCTTCT
CAGCTATAGTGTGAGTCCAAATCTTCTGTTTTACTTTTCTTACTCTACTCTATACTCTACAAATAAAAAT
TTGTCACATGTTTACCTAATTAATTAAATACTACTATTAATTAATAACGATAGTATTAATAAGTCAATAA
TGATAGTATTTAATTAATTAGCTGAATACGTAGCATATTTTTATTAGTGGAGTATAGAGTAGAGCAAGAA
AAGTGAGACAGAAAATTTGGACTCTTAAATATAGCTGTGCTCGAAAGTAAAAAAAAAAAAATATATATATA
TATATATATATTATTTTGTGTTCACACTGCTAGTTAGAATATTTGTGGAGTTTTTTTTTTTTTTTTTTAA
ATGGGATGTATTATTTTATTGTAGTAGATATATTATTGTATTGTGATATTTATATTATTTTATTGTAGTA
AATATATTATTGTATTGTGATGTTTATATTATTTTATTATGTTGAAAACTAAAATAGATCCATTATTACA
GTATAAGAGTAGGTAAAATAGATAAAATAACTTTTTATGATATTAAATAGCTAAAAATATAGCTTCACTG
ACTAGGATACTCTAAGAAGTTGCGTAAAAAGGTTGTTCACAACAATAATTTATAAATATATTACAAAATA
GGTTGTGAGAGAAATACTTAAAATGACAAAATTTGTGTTACAACTTGCCTACTCTCGTGCCGAATTGGGA
GTACAAATTGTGCCGGCATTACTCCTAGTATATATATAGACATGATTATAGCATATAAGGAACACAACAA
TATTGCCACGATTACTCAGCTATCTCTCTCTGTTTCTAGTGTTCTCCTCGTTTCTCCTCAATCCTCATCA
ATGGCTTCAGCAGTGTCCACGAAGGCATCACCTGTTCATCCACCAAAGATAACAACCGTGAAAGCACTCG
TTGAATCAACTGGCACCTCCTCCATCCCTTCGTTTTACAACTTCACCCCTTATCTCCGTGATGAACCAAT
AGCCAATGATCCAGAAGATTCAATCCCCATTATCGACCTCTCTCTTCTTGTTTCTGGTACTCCTGAAGAA
CAGTCCCAAGTCATCCATCAACTCAGCAATGCCTGCTCAGACTGGGGCTGCTTCATGGTACATACTGTTT
ATCTTATCGGTCTTGAGCTTGCTTCTTTTTTCTTTTTTTTTTTTTTTTTTTTTTTTTTTTTTTTTTTTTT
TACGTGTCTATAGTTTTTGCATGTTACTGAGATTTGATGAGGGTCTTATTAGGTGATTAATCATGATGTG
ACGGAGAGTCTGACGAAGGCAATGATAGGTTCGTTTCAAGAATTTTTCGACTTGCCAGAAGAGGAGAAGA
GAGAGTATCAAGGAAACCATGTCATGGATCCAATCAGGTGTGGTACAGGCTTTAATCCTTCAATGGATAA
AGCAAACTATTGGAGAGATTTTCTCAAGTGCTTCGCGCATCCTGAATTTCATACACCCAACAAACCTGCT
GGGTTTAGGTACATTTCTTTTCATTTCAGATTTAACCTCCTAAATCATATGATTCTAGTGTCGTTTAAAC
ATTTGTATATAAACTTTTGGCCACACTTCCTTTGCAAGTCGATTTTCAAAAGTAAGTTCTAATCATGAGT
TTGTAACATTTGATATTAAAGCCAACCACTAGGGTCGACGGGTCAACCTAAAGGCTGAAGTTAGGGATGG
CAATTCGTGTTTGCGTGTTGGGTTTGTGACGGGTTAAGTTATGCGTATTATGTTATATGAGTCAACACAA
ACCTAACCCGTTTACTAAACGGGTTAAGATTCCTCAGCCTTAACATGGCCTGTTTATTAAACGGGTAAAC
CGACATGATATGTTTAACCCATTTAATTAGCAGGTCATGTTAGGGTCGACACAAATGACCTGTTTAATTA
ATAGGTCATAACTAATCTGAATAACCTGTTTGATTAATAGGTCATATCTAACTCGAATAATCTATTATCA
ATTTCCATATATTTAAAATTATAATACAACCTTAAATCAAGTAATAAACATTCAGTTACAACTTACAATC
ATAAATCTAGTAATAATAACAAAAGCAAATAATTCTTTCATTCCTTCAAAATTAAATTACTAGTCCAAAA
GGTTCCAATCCATACTATTAAATTCAATAATATGTCAAAGTTCCATAATTAAAAAATAAATAAATAAAAA
AAAAACAAAATTATTACATGGCATTCAAGTGCCATATCAAAAACTAAAAGACAACACAATTTATGATTAA
AAAACTATGATTACATGGCATATCAAAATAGCCATGATGGCAAAATTTAAGTCCAACCATCCAAGTGCCA
TATCAAAAACTAAAAGACATCCAGATTAAGAGACTATATTAGAAGAGATTGAGGGTTCCGTTGAAGAGGT
GTCTTTCTTGTGCTCATCCTTGTTGATATTCATGCTCATTATATTTTTAGCATACATTCAAATGGCGCTT
CAAATTGCCAGTACCATTTGCTCCAGCAGTAATATATTCCTTTCCACACTTCTTGCATTTGCAAGTAGGC
TTTTCTCCATCTTTTTTAGGAAGCATCTAAAAGAAATGTCAAATACTTGAAGTCCTTCTTTTGTAGTCTT
TGTTATTTGTCTTACTCTTAGACTTAGCCTTCTTTGGCGGAGGAAGTTCAGCTAGTTCATCAAATTCATC
TTGGAAGTCAATGTCTTCACCTTCATACTTAGAGTCAACATCACTTTCCATCTAGAAAAAATTTAAAGAG
AAATTATTAATCAGAAAATTAGTAATTCTAAGATGATAGAAACAAATAAACTTGAAACAAAATCGTAAAG

CACGACTAAAAATTTTGAAATCAACAAAAAAAAAAAAATATATATATATATATATATGTACTATTAGTACG
AAATAGTTAACAATATAGTGATTAACACAAAAAACAAATAGATTTTCAATTATTTTATTTTAAGATGCTA
GAAAACAAATACAAAAAAAACTTGAAAAGGAATCGTAGAGCAAGACCCAATTATTTTAAAACCAACATTT
AAAAAAAAAAAAAAATCCACTATTAGTACTAAACAATCAACATGATAGTGATACACAATAAACAAAAGAAT
CTTCAATTATTTTATTTTAAGATGATAGAAACAAATAAACTTGAAAATAAATCATAGAGCAAGACCTAAT
TATTTCAAAAACAATAAAAAAAAAAAAAAAAAAAAAAAAAAAAAAAAGAGATCTACTATTAGTATTTAACA
ATCAAAATGATAGTGATTAACACAATAAACAAACAGATCTACAATTATTCTATTTTGAGATGACAAACAA
AGAAACCTGAAAAAAAAAAATTCATAGAGCAAGACCCAATTATTAAAAAAAGAAAAAGAAAAAAGAATCTA
CTATTAGTAGTAAACAATCCACATGATAATGATTAGCACAATAAACAAAATTTACAATTATTTTATTTTT
CAGTGCTAAGAGAAAAAAAAAAATAACAAAATAAAACACAATAAAATAAAACCTACAATTTGTCTCTGCTA
CTACTAGTATTGTTAAAGTCATTGTGAATTAAAAATATATCTTTTGAACAAATATACAGGATCTATTAGT
TTGTACCTCAATGACATAGATACTAGAGAGAGTGAGTCTATCTATGCTTCATCATCTTAAACACGTTGTG
TTGATCAGTGCTATAACAAAATTAGATTCCCCTATATATTTAGTGAAATTTCTATCAATTACACACACTT
TGTTTTTTTGAACTAAAATAATTCATGATTTTCAACTTGGAAAAGAAACAATTTTTTTTTGCAAAAGTGG
TGAGTGTGTGTAAAACTATAAATGAGTGTGAGAAATAGACATTGTTCATATAACTTGCTAGTGGATTCAG
CGGCTGAAAGCTGAAGAAAGGGGTGAATTGGTGATCAAAGGGGTAGCTACGGTTTTGAGGAGAAGAGACA
ATAAAGGATGTGGCTAGGAATTTGAGAGGAAGAAAGAGTAGAGGGTTGAGAGACATGGGGACGGCTAGGA
TTTTGATTTTAAATAGTGCAGAATCTGTAGAGATGAAAGAGGAGCAGCTGGAGTTTTGAGGAGAAGTAGA
AGAGTTAGAGAGTTAAGGCTGATGCTAAAAATGAAGAGACAGAGAGTGAAAGAGGCGTGACTGAGATCTG
GTAGTGCGGGAAATAAGTGAATAGGTAAGCTTATGTACCTAGGTTTATAAGGGTGAACTAGTAAAATTAC
CTTTATAAGCTAATCGGGTCAAACAGGTTCACATGTTGACCACAAACATGACCCATTTAATAAACGAGTC
AATCGTATCAACCCAAATATGATCGAAACCCATTTAGTCTTAACCCATGACCTGTTTACCAATGGTTTAG
TTGTGTCGGGTTTGCAAGTCGTGTCGGATTTTGCCACTCCTAACTGGAGTGTCCCACATTGTTTAAGTGT
AAACCTAAACATGTGTATATAAGTTTTTGGACACCCTCTCTTTGCAAGGGTGATTTCTACTCATGAGTTT
GTATACAATGTAATTTGTTCAATCTACAATGTAAATTTTATATTGCTAGTTCCTTATTTTGATTATGATA
AATTTATTTTTCAATTAAAATTTCAAAAATAAAAATCAGGTCATGGCCAATGCAATTCATAATGAATGGA
TTATTTATGTTTTTGTTCACACAGTGAGATTGCAAGGGAGTTCAGCAAAAGAAACCGGGAAGTAATAATA
ATATTACTGAAAGCTATATCAAAGAGCTTGGGGTTAGAAGAGAGCTACGTAGAAAAGGCTGCCAATTTCG
AGTTGGGTTTACAATTGCTCGCTGCTAACTATTATCCAGCTTGTCCAGAGCCAGAGAAAGCAATTGGCAT
CCCCGCTCACTATGACCATGGTTTGTTAACCACTTTGGTAAATAATGGTATCTCTGGCCTTCAAGTAAAG
CATAATGGAAAATGGTTTAATGTCAACATCCCTCCCAATGGACTCTTTGTTCAAGTTGCTGATCACTTGG
AGGTACATTCTGTCATGCTTCTTGAACTCCATTTTTACTTGTTATCACGTGTTATTCATCTTCTCATTAT
CTTTCTTAATTACCTTTTCTTGTTAAGCAACGATGCCAGCTAAAGTTAACTAATAAGATCGGTCTGATAC
AATTTTAGGTTTGATAGGCCAAAAACTAATTGACTCCTTGTGATAAATTAACCAATTAATTTAGACAAGT
GAATTAATTAGGTTAATTAACATGCAAACGCATGGTAGCACAAACAAATCACTAATAAACTAAGTATAAA
ACGAAAAATAAATTGACACGGTGATTTGTTTATGAATAGGGAAAACCAATACAGCAAAAACCCCACCAGG
TGATTTTAAGGTTACCACTCCTGAAATTCCACTATTATCACAACAAGCGGTTACAAGTAAAAGAATCTCA
ATACCTTATACCAACTTACAGTTGAATCCTTACCCCAATACCTAATTGGACTTGTTCTGTAGTGACAATT
TCTCCTTTTGATGCACGACTCCCAGTACGTGACTAACCAATTGTGCGGATCCCAATACATGACTTCAATC
ACCAACTGGAGAAGTTTGTTGGCTGCAAAATTCTTCAGTTCATCCCTACGATGAAGATCAAGAAAATGCT
TGGTCACACAACCCTACGGTGCACAAATATAACAACTTCTTCACAAGAATGATGAACTAGGGCAAAACTG
TGTCTCTGGTCATAAATTGCTTGAACAAACTTTACTCAACACTTGTGCAACTTGTGAACACTTTGACAGC
CTTTAAAATAATCATTTTATATGTCTAGGGTTGTGAGAAAAGAAAGCTAAAACACATAAACACGGATTGA
AGTCAAAACAGAACTAAAAATCTATTTTTCATAAATCTCGACAGATACCCTATCTGTCAAGCTGCTGCCG
AGACACGGGACTGAACATCTCTTTAAGCTCAATAGATGGCTAGCTGTCGAGGTTTAATGACAGACATTTT
TTAGCTTGAATCTTAAACAGACTTGCATGACTTCAACACTTGATCTTGAAACAAAATTTCTTGAAGTATT
AAACATATTCTAGATCTACCCAAATACAAGTAAAGTGTGCTATGTCAAAAGATTAGCCAATTACATAAAA
TCGTGACATATGTTCTTAACATGTGAATCACATATGTCCTAACAATCTCCCCCTTTGGCAATTCGTGACA
AAATCACAACAAACAAATGAGATATGAGAGAAGTCATAAATCACTTAACTCACATTCACTTGTTGAATAC
AATAAAATCTATCCGAATACAATAAAATCTATCCTAATACAAACTCTTAAAAAACTTTGCAAGAAGAGAG
TTTATGGCAAGTAGACTTTGACAACCTGTATTTCTGAAACACTTTAAACAAAACTTATCAAGGCATCTTT
GTGTGAAATAGAAATAATAGATTGCATACAAGTAATAAGAACCATGTTTGTAAAGAAAGAAATGAAACAA
CACATGCAAGGATAGGTGAAAGAAACATACATCAACATATATAGAAAATAAGTAAAATGTATGTCAATAA
TGATCACAAGACCTAAGGTACAAGAATAAATGTATCTAAAGTAGAGAAAAGAAAAAGATACAAGTAATCC

TCACTACATCCCTCAAAATGCACTCTCCCCCTAACAAAAAACTCCTATGCTAACCCTCCCCCTAAGTATG
ACTACTCTCATAACCAAAACTAATCCTCCTTTTTGTCACAAGTGACAAAGGTTAAAAATGTCAAGTAGAC
ATCTCATCATTGCTGGGTGAGTTAGCATCGCCATCATCCTCATCATCAGCATCACTCTCATCTGTAGGGG
CCACAAGAGTGGAAATCATAGTATAACCACCCATCTCAGCCTGACATCAAGCAACGCGTCCAACACGGGT
GTTCACTTGACACAACTCATCACTGAGAGTGTCGAGGTGAGCATCCATGCCCTGAGGCTAGGCCATGATG
TCCTCTAGAGTCACTCCGCTCATAGAAGAAGAGGGAACGGATGTGGATGGAGCAAAAGGAGTGGGAGGAG
CTGCCGTACCGAATCGCCTCGATCAAAACTACACCTCGCTCCGTTTAACGGTAGTGTAGTCAATGGCACA
CATAATGAGGAAGTGGTTGGAAGAGGGAAAAGAAACAGAAAAATGGCATAAAATTCTTGTGATAGCTGAA
GAGAAAATGAGCTTATCACGGGTAGCTGTATCCCTATACACATCTATAAGAGAAAGAATAAAATGTGAAG
GAAAATCTATAGTGAGGTGCTCTGACAAAAAAAGCAAAAATCGAGCACGAGGCTTTGTGATAGAGTTATA
GTGAAAGAATTGGTGCAAAACAAAAGTCATCACCATGTTCATGAATCTAGGACCTTTAGCAAAGGCCTTA
CATGATGTAAACTGACGATCACCCCAATCAAAAGGGCGCTCGTAGAAAGCAGAGATCATCTCGTCTTTGG
ACACAGTCCTCAGACACTCACAACCGGGGTAGTTAGGATGCTCTACCCTCAGGACACAAAGCACATCAGA
TACCAACTCCAGTGTGACAACATTGCGCGTATCTTGACCGTGAGTATAAAAGAAAGGTACCGAAGAATCG
ATTCCATGCACGTTGGAGTAAAAATCCCAGATCAGTGCAAAAGGACATGTGATCGGGACGTCACACAATG
ACTGCCAACCTCGACTGTGAATGACATCGGGTAGGTCAATGTTGGTGAAGTCTGCCAAAATGACTCAGCG
TTCCGAATGAACACCTCATTGGGAAAAGTTCTTCGAGAAGTCTTTTCGGGCATCCTCATCACGAAACCAA
ATAAAAAAAGGGGTAGGATCAGAAGATGAAGTGGATGCCCTAGAATGCAAAGGGTTTTGGGATGGAGCAG
ACTTATGTTTTGGTGCCATTGACGCACTAACATAAACTAGAGAGTGAGGGGGAAGAAAGAAACACTCAGA
AAAGCTCCAAACAATTCAAATATATAGAAATATACTTAATGTAAGGTACGTATGCATGAAAATGCATGAG
CATATGACGTGCAAAGTGAAAAACATTATAGGCTTAGCCCAATCCAAACCTACCAGCACACAGACAGTTT
AACACATATAACAAACATCTAAATGCATGAAAATATAGTTATAATGTATGTGTGATGCAATGCATGAGGT
TAAAAGATCATTTAAACAAAAACCCATCCCAAAATTTCAATAAACACTCAACAATTTTGGAAAACCCCAA
AATTTTCAAAAACCCCAAAACCAAGGTCTAAATGCATGAAAAATGAGAGAAAAAGAGATCATACCAAGCG
ATTTGAAGCAAGGAAAGGCCAAAAATCACATGGGTTGAAGGTTTTGAGAGAGAAGAGAGTGTTTGGGAGG
TGAATAGGTATGGATAGATCGAGAGAGATAGAGAAAATGAGGATCGAATCGCGCTAAAACCTATTTATAG
GTGTTTAGTAATTCTCGATAGATAGAGGTGTCAAGAGGTATCGAGACATGTGTCGAGGAAAAAAGGCGTC
GATAGATGAAGCAATCGAGGAGCTGTTGAGGGACAATTCAACAAAAGCTAAAAAGAAGCTCAGTCGATCC
ATCAACTGTCAATAAGCTACTGAGAGTCTAGGAACTTTCTCGATCGATCCACCAGTTATCGAGATTGCGA
TAAGAAATAGCTTAAGATCCTAACAGAAAGGCTAGGTGTTGAGGTTGCTTAAAAACAATTTTTCAAGAAG
GGAAAAACACAGATATGAATGCAATTAAGCATGCAATTCAACCAATGATCTAATCAACATTTTAAACTTT
CAAAATCATCTCTCAACAACAATTTTTAATCCCAAAAATATACACACACACTAAACAAGTCTAACTTATT
TTATATTTCAAAAATAAGTCAAGACAGTTTAGTGAGTATACATTAACACATGTAAAACCTTGTGATGGCC
AAATCACATTGTACCTGCACATGTATCAAGAGTAGCAAAGAATACTACGTGTTGTGTGTGAAAAACATTG
TAAGATTGCATAAGTATATACATGTTATGACAATTTGAGATATAAGAAAATCACTTTAACTCACACACAA
TTATAACTGTTTGATGGGGACTATCACCTTCGAGGTACATCCTATAACTCTCACATCTCCTAGAATATAT
GCTTGCAATCATATTTAAAGCATTTTGATATTTTTGTTTTTTATTTTTCTTTGCATATTTTTCTTTTTAA
GCAAATCATGCATAGGCATATAAGAGAGAGAAAAGAAATACCCAATTATGTTAAGCATTTGACATTCCAA
TTTTGCTATGCTGAAGCACACAAATGTTATTGATATTGCACTTTTGCTATGCTAAAGCATACAAATGTCA
TTTCATGATTGACAAGCAACAGTGGTGAGATGGTTATTTATGCCTTTCTCTTAGGATTTTCTAGTCCTTC
CCGTCAAATAGAGTGATACGAGTGTTAAGTACAAGAGATTACTTAATCTTACTCATCACAAACAAAAGCC
ACAAAACTCACTTGCTTAGTTGTGTATAGAGATGCTCATCTAAGCTACAAGAAATACAAAGTTTATAAAA
CTTTGTTTCAATGGTCATCCATGGTACACAAGTACCAATGTACACAAAACACATACTGTTTTTGTATTTT
TCCGATTTTTCAATTTTTTTAATTTATTTTCATATGAAAAACAAAATAAACAAAATTGAAAACAAACAAA
AACATGTTAAACAAAGCAAAACATAAAACTAGACTGACTCAAAACTTGAAAGTAATAACACAAGTAATGC
ACACACAAAAACAAGAAGAGAGAAAGTGAAAAGTGATAGAATCACTTGAAGCCATTTTCCTTCCATGCCT
TGGAAGAACCTTTCCTTTGAGTGTACTCTTGAACCGGCGGTGAGGGAGAACAATTGAAACCGTTCAAGTT
TGAAGGAACATGAGGGTTTTGAGAAGATCTCCAAGGGGAGTAAGAGAGGATTAAAGATGATTCTGGCTTC
CAGATGCTATCATGCCGTTGCTCTGTTGAGTGGTTAACCACTTATAACAATTTGGTCGAGTATGACTGTC
AACTTCACAATGATGACAGAAATGCTACTTCTTCTGTTTAGGCTTTTGAGAGTTAGCCTTCTTAACCTTA
AGGTTTTTAGCTTCCTTCTTATCATGCTTAGGGGGTACTCCTAAGATAAATTTACTTTTATCTATGTTCT
CATTAGCCAAATCAGTTTTAACATCATTATTCTCAATTTCAACATTATTACTAAGAGGAACAAAAACAGT
AGTACTAGTAGAAACAGTATTAGAAGAAGAGAAACCATACCCTAAGCCCGTTCGATCAGAAGTAGATTTC
TGAAGACTGAGCATTTCATCAAGCTTTGCACTTGAAGTCCTCTCCAATTGAGCTCTAACTTGAAATAGCT

CTGCTTCAAGCTTCTTTGTCTTCTTAGCCAAGAAATTGTTCTCGAACCTTTGTACTCCAATAGTCTGATT
AGCTTCATCAAAATTTATGGAAAGCTCTTCACGATCAAGTTCCACATCACTGAGCTTCTTGGTGGTCAAC
CTATAAAGTTTCTCATGTTTCTCAGAAAACTTGTATAATTTCTCATAAGCTTTATGGATATCATCTTGAT
CATCCATCTTCTCAAACTTGGACTCCACTAATTCCTCTTCTTCATCCACATCTTCAACAATCCTCTCAGT
AGGATTGACAATGGTAGTGAAGGCATTCAGGATTCCGTCATCCTCATTGTCGGAATCATCCTCAGGCTCA
GTGTCGCTCAAAGTAGCAGCAAGTGCCTTGCTCTTCCCAATACTCTTGAGATATGTAGGACACTCCTATT
TCATATGATCGAAGTCTTGACACCTAAAGCACTTAGGTCCTGCGGGAACAGTGTACTGACCGCCATCCTT
AGCATCCTTCTTCCTTTTGTCTTAGCTCTTAAACTGAGAAGAACTGGATTGCCTACGGTTCTTGTCAAAG
CCCTTCCCATTGGCATTCTTCATGAACTTCTTGAATTGCCTGGTGATGTAGGATTTCATCTTAGAATCTT
CATCGTTTGAAGACTCATCCGTGTCACTGCTCTTGGCCTTAAGTGCCATGCTCTTGCCTTTGCTCGATTT
GCCAATTCTAGTTAACCCTAGCTCATAGGTCTATAGATTGCCAACCGACTTTGTCAAAGGAATCTTGTCG
ATATCCTTTGATTCCTCGATCGCCGTGATCTTTGCATGGAATCTCTTGGGTAGAGATCTGAGCACTTTCC
TCACAATCTTGGGTTCAGGAATGGTTTCACCAAGATTGAAGGCTAAGTTCACTATATCCTTGAGCTTGGC
AAAGAACTCATCGAACAACTCATCCTCCTCCATCTTGATTTCTTCAAAGCTTGTAGTGAGCCTCTGAAAC
TTTGAATCTTTGACAGCCTTGGTTCCCTCATAGGTTGTCTGGAGAATGGACCATGCCTCCTTAGCAGTTT
CAGTAGACGATATCTTCTTGAACTCCTCATTAGTGATTGCATTGAATAAAGCATTCAATGCCCTGCTGTT
GAAATTTGCCGCCTTGATCTTGGCATCATTCCAATCGGTCGGCGCTTTCTTTGGCTTGATCCAGCCAATC
TCTATAGCTTGCCATACCTTCTCATTTAAAGACTATAAGAAAGCTCTCATGCGTACTTTTCAGTATGCAT
AGTTAGTACCATCAAATAAAGGAGGTATAATTAATGACTGTCCTCTATCCATGACAAACAGGGGTCAATG
GATCAACATAGCAAAGATTAACTCTAATCAGAGTTTGCCTGCTCTGATACCACTTGATAGGCCAAAAATA
AATTGACCTCTTGTGATAAATTAACCAATTAATTTAGCCATGTGAATTAATTAAGTGAATTAACATGCAA
ATGCATAGTAGCACAAACAAATCACCAATAAACTAAGTATGCAGAGGAAAATAAATTGACACAGTGATTT
GTTTACGAATAGGGAAAACCAACACGGAAAAAACCCAATCGGGTGATTTTAAGGTCACCACTCTTGAAAT
TCCACTATTATCACAATAAGCGGTTACAAGTAAAAAAATCCTAGTACCTTATACCAACCTATAGTTGAAC
TCTTACCCCAATACCCAATTGGAGTTGTTCTGTAGTGACAATTTCTCCTTTTGATGCACAGCTCCAAGTA
CGTGATTAACCAATTGTACGGGTCCCAATACGCGACTTCAATTACCAACTAGAGAAGTTTGTTGGCTGCA
AAGTTATTCAGTTCATCCCAACGATGAAAATCAAGAAGATGCTTGGTCACAAAACCCTGCGGTGCACAAA
CACAACAACTTTTTCACAAGAATGATGAATTAGGGCAAAACTGTGTCTCCGGTCACAAATTACTTGAACA
AACTTTACTCAACACTTGTGCAACTTGTGAACACTTTGACAACTCTTAAAATAATCATTTTATATGTCTA
GGGTTGTGAAAAAAAAAAAAAATCCACGGATTAGAGTCAAAACAGAACTGAAAATCTGTTTTTCATAAACC
TCGACAGATCCCCTATCGAGCTGCTGTCGAGCCAAGGGGCTGAACAGCTCTTTAAGCTCGATAGATGGCT
AGCTGTTGGGCTTTAATGACAGGCACTTTTCAGCTTAAATCTTGGACAGATTTGCATAGTTTCAACACTT
GAATCTTGGACAGACTTGCATGTTGAAACAAAGTTTCTTGAAGTATTAAACACATTCTAAATCTACCCAA
ATACAAGTAAAATGTGTTTTGTCAAAGGATTAGCCAATTACATAAAATAGTGACATACGTTCCTAACTAG
CGAATCACATATGTTCTAACAAAGTTTAAGGTGATAAAACATTTGCCATTCACATCAATAAATATATATA
TATATATATATATATTATCCTTGAATTCTTTAAAAGAGAGGGGTGGGGGTGGGGGGGGGAGTAATTT
TGTTCTTTGTTTATGATGAGGTCGGTTCAATTTTGTTACTTAAATTTGTGTACTCTTAAATATGTGGTTG
GTTTGATTTTAGTCTCATAATTATGGGTTGCTTCAACATTCTGTTAGTCACGTAAGGGAAAAAAAAAAAG
AAGACAAAATTGTTCACTTTATATAATTTTAATGACTAAATAAAAACCCTTAAAATTTGAAGGGTTACAT
GATCAAACTTATGCCAAATTTTATAATGGAAATTAGAAATTACCCAAAATTTATTTATGTTAAATGTGAC
AAGTGACATGTGTGAATAAGTATAAAGGCAAGACAGAGAAATAGTGCTTTGATGGTGACTTGTTAACAAA
AGTATTTTTTACTTTTTGCCAAATCTGAACCACTTTCTGAAAAACTTTTTTTTTTTTTTTTACAGACGA
CTAAGAACACACAGAATTAAAATTATATGGTTTTCTTCCATATGGGATACAAAGCAATTGTCTAAATACC
CAAAAGGAACTGTTAACTAGCCTAAATGGCCAAAATGAAGAAAAAAAAAGGTAGTTGGTGTTGGGAATGGG
GTAAGTACCAACATACATATTAGGGTTGACCTTTTTCATAAAACATAAATTATGAAAATTTCTTTGATGT
GAGGAATTACATAATTGCCTAACTGGCCAAAATGAAGAAATAACCCTATAAATATAATATAAAAGAAAAT
TATAAAAAGGAGAAATGAAGCGAATACACAAATAAGAGTTAATGGTTGATCGTTTCTTTTTCTTTAGATG
TGTGCGTAATTATATATATTTTTATTTAAATATCATTGACTCTATGTTTCTCACTTATCATGTTCTTGAC
ATATGTATATTTAACTAAATGAACAATTTTTTTGAATTGATCAAAATTTTCTGAATATAATGTTTCCTAC
AAGATTTTGAGCAATGGCAAGTACAAGAGTATTGCACACCGGGCACTTGTGAATAACAAGGCTACAAGGA
TGTCCATAGCTCTAGCACATGGACCAGCACTAGACACAGTTATGAGGCCAGCTCCAAAGTTAGTTGACAA
TGAAAGTGATCAACCTGCATATGTTGAAATGACGTATGGAAAATTTTTGGAATTGCAGCAAACTGGGAGG
CTGAGTTCTAAATTCCGGTCAGATACTGAACAAAACAAAGCAGTCTGAGCTAGTTTATTGGTTTACAATG
CCAATTTCTCAATTTAGTGTTTGATAATAATAATAATGAACAAATGTTGGCAAATTTGGTTT

>NC_044914.1:23436789-23443191 *Quercus lobata* isolate SW786 chromosome 11, ValleyOak3.0 Primary Assembly, whole genome shotgun sequence
CTCTCTCTCTCTCTCTCTCTCTCTCTCTCAGAGTCATCCATGGCTGCTATTCCCCAACCCTCTCAGCCAT
CTCCTACTCAAATTTCCAAGATGACGACATATATTAAGAAAGTAGCTGAATCACCTGGGCTCACCTCTAT
TCCTTCCAGTTACGCTTTTACCCCTAGTCCCAATGTTCAAGCAGTTTCTGAAGACCCAGAAGACTCAATT
CCAATCATTGACTTCTCTCTACTCACTTCTGGTACTCCTGATCAACGGTCCCAACTTATCCAAGAGCTTG
GCCAAGCCTGCAAGTACTGGGGCTTCTTCAAGGTAAAACATATATTTACATTACATAGTAGTAATCTTCT
GCAATATTGTATATGCATGTGTACCTGATTTTGATGAGTCTCAGTTGATCAACCATGGTGTTCCGGAGAG
CTTAATGGAGGCAATGATAGAAGGCATTAGAGGATTTTTCAATCTGACAGACGAAGAAAAGAGAGAGTTC
GAAGGGAAAAACCTTTTCGACCCAATCAGCTGCGGAACCAGCGTCAATACATCAATGGAAAAGGTTTTTT
ACTGGAGGGATTATCTCAAGGTCTTTGTACATCCTGAGTTTCACTTCCCCAACAAACCTGCAGGATTCAG
GTACTCGGGTTTCAATAGTTAGAATCATTGTCTAAATCAAATAAAAAACCTATGTCCCATTTCCATTTGT
TGTGTTGCAATTTAAGCTCCGTTTATTTTTGCCGTAATAGCATTTTATCGTGTTTTGCTTTTGTAAAATT
TGCTCAAATTCATAATTTTAAAAATCGGACTAATCAAAGGACAAAAAAGAGGAGTTGTTCTCGGTGTTTT
GTTAAACGAGGATCAGACTGATAAGGCTTTTTTTTTTTTTTTTAATAGATATTTTTTAATTTTTAACAT
GATAAAAAAAAAAAAAAAAAAAAAAAAAAAAAAAAAAAAAAAACACACACAATATAGAAATTTTATTCTAA
CATAATTTAAATATATATGTGTGTAAATGTTTCTCTTGAAAACTTGAACCCCAATTCTTATCCCTCACAT
CTCACAAGTACTTATATTTGTAGAGTGACTATCGTGCAATGAGTGTGCAGTGATATTAATGAATCAGCCT
GATAGTTGAATAGGTGAGGTCATAATTAATTAATTATTAATTTTAAATTATAAAAATATTTAATAAATTT
AAAACTAAGAATATTTAGTATTAACTACATAAAAATTTTCTTTTAAGAAAAGTGTGAGTTTAGTTATCAC
ATCTTATATTTAGAGAATTAAAAATATGATAAGTATGGCGTGTAACATGTTATGTAGCCTTTTCTTCTTT
ATATCAAAGTTCAAGAAATACAAAGATAAGCCAATGGTTGAACAAAAGCACAAAAACAAAAAACACACAA
CTAGAAGAACGACACTCATTCATGGTCCAACCTACCCCACTTCCCCCGGCCCTTGTTGTTTATCTTCTTC
TTATTATCGCCGCCACTTCCCACTGACCCCACTTGTTTATCCAAATACCTCGTTGCTCATCTACAACTTG
TATAAGTTTGAAAACAGCTAATTTTTAGTTTTAGTTTTTTTATTTTTTATTTTTTTTATTTTTTATTTTT
TTATTTTTTAAATATACTTGAACTTATTTTAAAAGGTTAGATTTTAAAAATCTATGCATAAAAACTAAAA
AAATAATAAAATAAAATTAAAAAAACTTATAAGTTAGTCTCAAATACACAGAGTTATGCCTTGGTTGCCA
GGACAAACATAGGATTTTAAGCTAGCGGAGGCTAGAGTAAAAAATAATAATAATAATTAAAAAAAAATCA
TGCATATAATATTAAAAAATTATAAATACTCAATATTGTTGTTTCTAAATATATTAAGATGTAATCATTT
ATCAACAAAGATAAAAACAAAAACAAAATAATGTTTTTTTTTTTTAATCCTAGGCTAGCTAGGATTTTTT
TTTTTGGTTGAAATTAGAGCAGTCATAGTGGTGATGCTAAAAATTTTAGCTTTTAGCATCTCAAAAAGCT
GATTTATTTATTTTACTACCACACTTTACAATACATCCAATATCAAATATATATATATATATATATATAT
TTGCTAAAATACAATCACCATTGTGAATGTTTTTATTGTTTTTGTTAAGTTTTTGGGTTGGATAGTTACT
ATTTGGGTGAGATGAGGGCTTATTGGGGAGAAGGGGAGCCCAACTACAAAAATGAAATGTATTATAACTA
TAAAAAATGAAGAAAAAAAAAATTTGGACCCAAAGGGGGCCCGGGCCCCCACTTGGCTCTGTCCCTGCTTG
GTTGACATGCTTGTTGCCTACCCAGATGGTGCCACCATTAAATTCCTCTCTCCAACTCCTTTTTTTTTCT
TTTTTTTTTCTTTTTTTAATTTTATTATTTATTTATTTATTTACACTCCCATCATTCATATTAGTGGCAA
ACAATTGTCATAGCTGCTGCATTTTGGTGTTTTAAGCAAACCACTCCATAACCGAACAAACCGTCTCGAT
CCACATTTCCTCCATTCAAACTATCCGGTTCTAGTTGAATTTAATTTTTGTGGCATTTCTAGTTTTTTAA
AGCAGCCGTATCGGATTAATATCTGGTTCCCAATTGAATCAGTCAAATCGGCCGGTCTACTTCGATTTTT
AAAACCATGGTCAAACATGAAAATGTTTTCTTTTAATTGTAAAACCCTTTGTAAAATGTTTTTACCTTAA
AAAGCTTGGTTAATCATTTTGCAAAAACACACAACGGATCTTCCAACCCATCTAGTGTCTACTTCACCAA
AGTTGGAGGTTAGGTTGAATTGGTAACCGAGCTGCTGATTTTGGTGGTTTGTGCTTGAAAATTTTTACAT
GTCACAAAACATTTAAAAGCATTTTATAGCAAAACAAATGGAGTGTTATGATTCACCAATCATAACTTGT
TACATATACTTTTTTTCTTTTGTTAAATCACTAAAGTTTAAAATGAAAAAAAAAATAAAAAAAAAATAAAAA
AATAACCTAACACATAGAAAAGTTTAATTGGTGGAGTATAAAATACAAAAAGTCGAACTAGAAATTCCTA
ATGGTCTAAAACCAATTTTGAAATATGCATAAATTGCTTATGCTGAGTTGATTGCATTTCCATATCCAAT
TATCAAAATGGCTAACAAGTTTATTCACTGGCTTTGTTGCAATTGCTGCAGTGAGCTTTCATTAGAGTAT
TGCGAAAGAACACGAGGACTAGCAAGGGAATTACTTAAAGCAATATCAGAGAGTTTAGGGTTGGAACCCT
TCTACATTGAGAAGGTCATGAATTTAGATAAAGGTTTACAAATGTTATATGCAAACTTTTACCCACCCTG
TCCACAGCCAGAACTTGCAATGGGCTTGCCACCACATTCGGACCATGGCTTCTTGTCTCTTGTCACACAC
AATGGAATTGGTGGCTTCCAACTACAACACAATGGGATGTGGGTCAATGTTAATGCCATTCCCAACTCCT
TTCTAGTTAACATTGCTGATCAACTTGAGGTGCAACCTTTTTTCTTAATTACGCATTCTTAATTCCTATC

TTACTCTTTTAATGTACAATTTTATTTGTTTTATGATTTCTTTTTTCTCTGAAGTTTTTTGTGCTATTGC
TAATTCCCCTTTTAAGTCAATGGTTCTTGTATGATTTTGGAGTCACTGGCATTTGGGCTCATGTTTCAAA
TTTTCCAATTTACAATAAGCTAAGACAAATTTCTATATGCCCTCTTAGGTAAGGTTTAGTGTTATGATAC
AATGTTATTGCATTGTGTTGAGATTACATATGTTATTTAGCACTAGAAAGAAATATTAGGTAGAAGCACG
AATTATTTTGGTGATTTTGGTGGTTTTTTTTTTTTTTTTTTCCTGGTGGAGGGGGGGGGGGTTATTTAGT
CATTTTGGAGAATCTAGGAATATTCTGGTCATTTTAGATTATCTAAGGTATTTTGATCATTTAGGAGATT
CTATGAATGTTTTGGTCATTTTGAACATTCAAGGAAATTTTGGTCTTTATTTAGGTTTTTAGGGCATTTT
ACATAATTTGGGGGTTTTAGGGGGTTATTGTTCTAATTTTCAACATTCTAGGGGTTTTTTTGGTCATTTT
GTTGATTCATGATATTATTAGTCATTTTGGGGATTTTGAGAATATTTTGGACATTTGAGGATTTGGATGG
TTTTTTGGTTAATTTAGAGGTACCATGGGTGTTTTAGTTATGTTGAAGTTTCCAATGGTATTTTGGGCAT
TTCAAGGATATTTTGGTTCAAGGGGTATTCTGGTAAATTAAAAAGCTTTGTGGAGGTATTCTAATCATTT
TAAAGGTTTTAAGGGAATTTTAGTTGTTTTTGAGTTTCCATGGGGTATTTTGCTCATTTTGGGCGTTTAA
AGAGTATTTTGGTCATTTTGGAGATTCTATAGGTATGTTGCTATTTTTTAGAGGTTTAAAGGGTATTTTG
GTTATTTTGGAGGCTTTGGGTATGTACATTGGGAAGTGGATGAAGGTGGAAAACATTACTTTTATTTTTT
TGGTAAAAAATATTTTGTGCCCATCATAATTGCAATTTCTTAGTCAACAAAAGATATTTTTAGTTTCTAG
GTGAAATTCTTGGTTTCTTATAATTATTGCAATGTCAATTTGTTTTGTTTTAGATTTTAAGTAATGGGAA
ATACAAGAGTATTCTACATCGAGCAGTAGTAAACAAAAAAGTCACAAGGATATCACTTGTCATGACCAAT
GGACCACCACTCAATAAGATTGTCAACCCAGCACTAGAGTTAGTAAACAATGGAATTGATCAACCTGCAT
ACCATGGGATAACGTATAAAGAATACTTGCAATGTCGACAAAGCAACAGTCTTGATGGGAAAGGCAACTT
GGATCACATACGCATCAAAATTGTGTGAAGTAAGGGCTTTTGTCCCGAGGACACTATAAATGGCTATAGC
GCGTGTGTACTTAATTGGACAAAGTGTCCATCAAATGCTCCACTCCATTTGATGAGATACAATACAAACA
TTTATGCAATTAATTTTAAACATGTATCCATATCATATTAGATCTAACTAGTACATTCAAGCACTAAACC
CAAGACAAACCCTAGCTATTTAACTTCTTGTATAAGGTTTTATCGTCTATTATTAGTGAGTGTCTTTGTG
CATAGCAAACTTATTACTGATAATGTTCTTATGGCTTATGGTTTTTCTGCTTCTCCTGCATGTAGTCATT
TACAAGCTTCCTTAGTGTGAAAAGGGTATCATCACTAAGTTGTGTCAAGAAATCTACAATATATTCAGGC
AATTCTATCAACAAATTCTGCAATTTTGCACCCAGATTCTACTTCTACTCAAGTAACTCAACAGTCATTA
TCTGCCTAGAGGCTCTGACTTAACACTAACATCATTCTATGCTAGGGTTTTCTTCTTTTTAGTGGGTGGA
ATTTGAGTAGTAGTTTCACTTTCTATTGATAAGGCAATGATTTCTCATCAGTTCTAACTGTAAACTTCTT
CTTAATAGCTGTGGGCACAAGCGCCCCCCATCGCGGATGCTGCAGATGTGGTTTCCTCATAAGAAGTACA
CGTCGGCCTAGGATGGACCGAGTGAAGGGTGAAAATGGAGTCACTACCTAGATTAGGTCTAGGAATCATA
TGTATGACGCCCTTATATGAAGGACTAATCTTAACAGAGCATGTATCTGGAGTTTTGGTATGGGGATAAG
AAGGTGTTAGGCACCCAACCCCACCTGATCCGTAGGTTGACCTTAACTCATTGTGTTCCAAATCCTAATC
TCATCAAAGGGACTTCTAATAAGTTATTCTAAACTCACACACACACACACACTAACATGCATCTAACATC
CAAACATGGCATATCACAACAAGTGCATAAGAAGCCTACCATTTATATAACATGGCATTCACATTCATTT
ACCTAAGGGCAGCAAGTATATCACAAGGCAGTAGCATTAAATTATCATCAAGCACAAACCAAATATCACA
GCGTCATGGCATCAATCAAGCATGTTCATCATATTCAAGCACAGCATCATGGTATTAATCAAATCAAATG
CATGTTCATGTAATTATGCATGACTCACCTTACTTATCAATGCATGTTCATAGTTTGTTGAACAAGATGT
TCATAGCCATGCCACATTGACAATGACTAGACACAATTATTAGGCCAGCACCAGAGTTTGTTGACAATGA
AGGTCATCCACTTGCATGTGTTGAAATAACGTACAAGGATTTTTTGGAATTGCAACAAAGTGGCAAACTT
AGTTATAAATTCCAGTTGGATTTTGAACAAATTAGCAAAGGAGCCTGTGCTCGTTGATTGCTTTAACGTA
ACTGATCTCCGAATATAGTGTTTGATAATAAAA

DND1;

>XM_003552501.3 PREDICTED: *Glycine max* cyclic nucleotide-gated ion channel 2 (LOC100798823), mRNA
AAGAAGAAGAAGAAAAAAAAGAAAGAAAAGAGTAGTGTGAGGAGGAGGAGGCAAACAAAAGAAGGTGGTC
CCAAGTTATACAGAAAACGGCAAAGAGCAAACAGCAGGGTTGAGTAGAGAGTAGCAACCCCACCACCCTT
CCCTCAACGTTGCGTAACCGTAAGCTCCAATCCCCACCCTCTTCCATTTCACATGCAAATTCATCCCTCT
TTATTCTCATCTCATTTCCATGCTCTCTTCTTATATAGTACCCTTCTTCCACTCCATGTGCCTTCCTCTG
ACACACTCTAATCCCACTCCTCCCAATCCCTCCATGTAACTCACCTTCTTCCCCATGCTAATGGTTTCTC
TCCGATATCTCCAACCATGCACAACACCTTCTCCTCTCTCCTTCGTTGGATTAGCAAAAAGTTGCGACGA
AGGAATTCAATTAGCAATGGTGACAGTGGCAGCGACAGCTTCCAGAACGGTGCTGCCACAGTTGTGGATG
ACAATCCATTTAGCAGCGGGGTGGAGTGTTACGCCTGCACGCAAGTAGGTGTGCCGGTGTTTCACTCCAC

CAGCTGCGACAGTGCCTTCCACCAGTTGCAGTGGGAGGCTTCGGCTGGGTCGTCTCTGGTTCCCATCCAG
AGCCGACCCAACAAGGTCCTCGGCTTTCGGACCGTGTCCGGATCAAGTCGGGGGCCGTTCGGGCGGGTTC
TGGATCCGAGAAGCAAGCGCGTGCAGAGGTGGAACCGCGCGCTGCTCCTGGCGCGTGGGGTGGCGCTGGC
GATAGACCCGCTGTTCTTCTACTCGCTGTCGATAGGAAGGGAGGGGTCGCCGTGCTTGTACATGGACGGA
GGGCTGGCGGCGATGGTGACGGTGGCGCGCACGTGCGTGGACGCCGTGCACCTCTTGCACGTGTGGCTGC
AGTTCAGGCTGGCGTACGTATCGCGGGAGTCGCTGGTGGTGGGGTGCGGGAAACTCGTGTGGGACGCGCG
TGAGATCGCGTCGCATTACCTGCGATCGTTGAAGGGATTCTGGTTCGACGCATTCGTGATCCTCCCAGTC
CCCCAGGTTGTGTTTTGGTTGTTAGTGCCAAAATTGCTAAGAGAAGAGAAAATTAAAATCATTATGACAA
TAATGCTATTGATTTTTTTGTTCCAATTTCTCCCTAAGGTTTACCATAGCATCTGCATGATGAGAAGAAT
GCAAAAAGTCACAGGCTACATCTTCGGCACCATTTGGTGGGGTTTTGGTCTCAATCTCATAGCTTATTTT
ATTGCTTCTCATGTTGCTGGAGGGTGCTGGTATGTCCTTGCAATTCAACGTGTTGCGTCGTGCCTCCGGC
AGCAGTGTGAGAGAACTAATGGATGCAATCTCTCTGTGTCATGCTCAGAGGAGATATGCTACCAGTCTTT
GTTACCAGCTAGCGCGATAGGAGATTCATGTGGTGGAAACTCAACAGTGGTAAGAAAGCCTCTGTGCTTA
GATGTTGAAGGACCTTTCAAATATGGGATCTACCAATGGGCACTTCCTGTCATATCCAGCAACTCTTTGG
CTGTAAAGATTCTTTATCCCATTTTTTGGGGTTTGATGACCCTCAGCACTTTCGGAAATGATCTTGAACC
CACAAGCCACTGGCTAGAAGTGATTTTCAGTATATGCATAGTACTCAGTGGACTATTGCTTTTCACATTA
TTGATTGGTAACATTCAGGTATTCTTACATGCAGTCATGGCAAAGAAGAGAAAGATGCAGCTGAGATGTC
GTGACATGGAATGGTGGATGAGGAGGAGGCAGTTGCCATCGCGATTAAGACAGAGAGTTCGCCATTTTGA
ACGTCAGAGATGGGCAGCAATGGGAGGAGAAGATGAGATGGAAATGATCAAAGACTTGCCAGAGGGGCTG
AGGAGGGACATCAAGCGCCATCTTTGCCTCGATCTCATTAGAAAGGTTCCTCTATTCCACAACTTGGATG
ATCTTATTCTTGACAACATCTGTGACAGGGTGAAACCCCTAGTCTTCTCTAAAGATGAAAAGATAATCAG
AGAAGGTGATCCTGTACCAAGGATGGTGTTCATCGTCCGAGGGCGCATAAAACGCAACCAAAGCCTTAGC
AAAGGCATGGTAGCCTCAAGCATCCTTGAGCCAGGAGGGTTTTTGGGTGACGAGCTGCTTTCATGGTGCC
TTCGCAGGCCGTTTATCGATAGACTTCCGGCCTCCTCGGCTACATTTGTGTGTCTTGAATCATCAGAAGC
CTTTGGCCTTGATGCCAATCACTTGAGGTACATCACTGATCACTTCCGGTACAAATTTGCGAACGAGAGG
CTGAAGAGAACAGCAAGATATTATTCATCCAATTGGAGAACCTGGGCTGCTGTCAACATTCAATTTGCTT
GGAGACGTTACAGGCAGAGGACTAAAGGTCCAGTGACCCCTGTAAGGGACACTAATGGAGGCACTGAACG
CAGGCTCTTGCAATATGCTGCAATGTTCATGTCAATAAGGCCACATGACCACCTTGAATGAAAACTGGTG
TGTCCATGATTTTTCTTATTGTTGTTGGTTATTGATCCTCAATAAAATTGTATTTCAAATCCATTAGACC
TGTTGAAGGTGAAATGTATTAAAAAAAAAACAACAAAGTGAGTACGAGTAATTGAAAAAATTATTGAGAC
CCCTTCTTTCATTTGATTGTAAGTTTACCATAATCATTTTTCATGGAAATTGTGTCTTGAGCAATCCCCA
TCCTGCCGAGGGGCTGTTAGGTGCTTAATGGACAATCAGTTGAGAAGAAATAGTAGAGTCAGTTACTTCA
CAGTTT

>XM_002284128.3 PREDICTED: *Vitis vinifera* cyclic nucleotide-gated ion channel 2 (LOC100245706), transcript variant X1, mRNA
GAGGGGTTTGGAACGTGTGAAAATCCTCTGGGTGGGTGTGTGGGCCGTGGGGTGAAGGGAAGGGAGTGAG
GTGGGTGAGCGTCAGACTGGTTTCCATGGAGGGACAGAGGGGGAGAGGTGAGGCAAGTGGACCCAAGCTC
TCACGGTCACAGCGGCAGTAGCCCCACAGACGCCATGTTCCGTAACTAGTTCTTCATCCTTACAACCAGG
ACAAACCTTCATGTGCTACGTGACAGACTCTCCCATTAATCATACCACTATGTCTATATCCCCCCACCCC
ATAAACCAATACCCATTACCCCCAACTCTTCATCCCCTTCTCTCCTAATCTTTTTCACTTCCGCCCTCTT
CTCTATCTCTCTCTCCCATGGTTTCTCTTTTCCGACAATGGACGCTCCACCCAACTTCCAAATCTCCCTT
CCAAGGTGGATTGGAATATTCCGGCGCCGGAAAGCAAACTCCGATGAAAACTACGAAGAAGACGACGGCC
CCATCTCCAATTCCATTGAATGCTACGCCTGTACTCAGGTGGGAGTGCCGGTGTTCCACTCCACCAGTTG
TGACCAGGCCCACCAGCCGGAGTGGGAGGCCTCAGCCGGTTCTTCCTTGATTCCAATTCAGGACAGGAGG
ACCGGGAAGGCCCGGTCCAACGGCCGCCCCTCGGGGCCTTTCGGGCCGGTCCTGGACCCGAGGACCAAGC
CCGTGCAGAGGTGGAACCGCGCGTTCCTACTCGTCCGTGGGATGTCCCTGGCAGTGGATCCCCTCTTCTT
TTACGTCATATCCATTGGCGGCGAGGGGGGACCGTGCCTGTACATGGACGGTGGGCTGGCGGCCATCGTG
ACGGTGCTGCGGACGTGTGTGGACGCTGTGCACTTGTTTCACTTGTGGCTGCAGTTCAGGCTGGCGTACG
TGTCTAGGGAGTCTCTGGTGGTGGGTTGTGGTAAACTCGTGTGGGACGCACGTGCCATTGCCTCCCACTA
CGTCAGGTCTCTCAAAGGCTTCTGGTTCGATCTCTTCGTCATCCTCCCTGTTCCTCAGGTTGTGTTTTGG
TTGGTTGTACCGAAATTAATCAAACAAGAGGAGATAAAGCTGATGATGACAATACTTCTGCTGATGTTCT
TGTTCCAGTTCCTTCCCAAGGTCTACCATAGTGTTTGCTTGATGAGAAGAATGCAGAAGGTCATGGGTTA
TATATTTGGCACTATCTGGTGGCGTTTTGTTCTTAATCTCATTGCCTACTTGATTGCTTCTCATGTTGCT

GGAGGATGCTGGTATGTTCTTGCAATACAACGGGTAGCATCATGTCTCCGTCAACAGTGTGACAGAAATA
GGTGCAATTTCTCTTTGTCTTGCTCAAGGGAGGTTTGTTACCAGTTTCTGTTACCGGCAGGCACAGTAGG
AAATCCATGTGTAGATAACTCTACAATATTGACCAGGAAGCCGCTGTGCCTGGATGTCAATGGGCCGTTT
GAATATGGGATCTATGACATTGCTCTTCCTGTTATTTCTAGTCAATCCATTGCTGTTAAGATCCTTTATC
CCATATTCTGGGGTCTGCTGAACCTCAGCACTTTTGCTAATGAACTTGCACCCACAAGTAACTGGCTAGA
AGTGATATTCAGTATATGCATTGTGCTCAGTGGCTTACTGCTCTTTACTTTATTGATCGGAAATATTCAG
GTATTTTTACAGTCCGTCATGGCAAAGAAGAGAAAAATGCAGCTGAGATACCGAGAAATAGAATGGTGGA
TGAGGCGGAGACAGTTGCCATCTCATCTGAGGCAAAGGGTTCGCCGTTTTGAACGCCATAGTTGGGCAGC
CATGGGGGGTGAAGATGAGATGGAATTGATCCAGGACTTGCCTGAAGGGCTTAGACGGGACATTAAGCGC
CATCTTTGCCTAGATCTTATTAAAAAGGTACCTCTGTTCCAGAGTTTGGATGATCTTATCCTTGACAACA
TCTGTGACAGGGTTAAGCCCCTGGTCTTCTGCAAAGATGAAAAGATTATTAGAGAAGGAGACCCAGTGCA
TCGCATGGTGTTCATCACCCGTGGCCATATAAAGAGTAGCCAAAACCTCAGCAAAGGCATGGTGGCCACA
AGCCTGCTTGAACCTGGAGACTTTTTAGGCGATGAGCTCCTGTCTTGGTGCATTCGCCGCCCATTCATAG
ACAGGCATCCAGCCTCATCTGCCACATTTGTTTGTGTGGAATCAACAGAAGCATTCGGTCTAGATGCAAA
CCATCTGCGGTTCATCACAGAGCACTTCCGCTACAAGTTTGCGAATGAAAGGCTTAAGCGAACAGCAAGA
TACTACTCATCCAACTGGCGAACATGGGCAGCAGTGAATATACAATTGGCTTGGCGTCGCTACATAATCA
GGACCAGGGGAGCTCCAGCCATTCCTGTGATTGAAAATGGAGGTAGTGATCGTCGGCTCAGACAGTGTGC
TGCAATGTTCATGTCACTTAGGCCTCATGACCACCTGGAATAAAGGCAAGTGCTGCAATTTTTCGCCGGT
CGGCCCCATTATTCAATAAGATCCTCTTTGTCAAAGTGTTGTAACTTGCATTGAATATGAATAAGAGACC
TGCAAAATCTTCCTTCATTTGATAATAAAAATTCATTTTCTTGATC

>XM_004232136.3 PREDICTED: *Solanum lycopersicum* cyclic nucleotide-gated ion channel 2 (LOC101266887), transcript variant X1, mRNA
CTAGTTTATAATTTTATTTCGAAATTAGTAGTACATACATTTGCCCCCACTTTCCCTCCTATCCAAAAAC
CTTCACTTCTTCTCCCACATGCATATATCTTGCCTCATTCTTCATGCTTCTACATAATTCTCTTGTTTGA
TTTCTTCCTCTTAAATTGAGTATATTATCAATTACTCAATTTTTTGCCTATAGTCAGTCCTTCTTTCTTT
CCCATGGTTCCGCCCTCCTCTCAACATCATGTCTTCTCACCAAGACGTCCGCTTCTTCCTCTCAAAGTGG
TTTGGCATATTCCGACGAAGATCAGTTCAACCTGATAACAGCGACGACAACGATGACGACATCAATCCAA
TCTCAAATTCCATTGAATGTTATGCATGTACTCAAGTTGGCGTCCCTGTTTTCCACTCCACCAGTTGCGA
TGGAGCTAACCAACCGGAGTGGGAAGCTTCAGCCGGTTCTTCTCTAGTTCCAATTCAAAACCGGACGGAT
TCAAAAACCGGAAAATCCCGGTCCAGTCGCAGCCGGCACACATCGGGGCCGTTCGGGCGTGTATTAGACC
CTCGAAGCAAGCGCGTGCAGAGATGGAACCGAATGATTTTATTGGCACGTGGCATGGCTTTAGCCGTTGA
TCCTCTATTCTTTTACGCCTTATCCATCGGCCGCGGTGGATCGCCGTGTTTGTACATGGACGGCAGCCTG
GCGGCTATCGTCACCGTGATTCGGACTAGCGTCGACGCCGTGCACCTCTTCCATTTGTGGTTGCAGTTTC
GTTTGGCTTACGTGTCGAGAGAATCGCTGGTGGTTGGTTGTGGGAAACTCGTGTGGGATGCGCGTGCGAT
TGCTTCTCACTATGTTAGGTCCCTTAAAGGATTTTGGTTCGATGCTTTTGTCATCCTTCCCGTTCCACAG
GCTGTATTCTGGCTGGTGGTTCCAAAACTAATAAGAGAAGAGCAGATAAAGCTTATAATGACGATCCTTT
TATTAATGTTCTTGTTCCAGTTCCTTCCCAAAGTTTATCACTGTATAAGCTTAATGAGAAGGATGCAAAA
GGTTACAGGATATATTTTTGGTACCATCTGGTGGGGATTTGGACTTAATCTCATTGCTTATTTTATTGCT
TCTCATGTTGCTGGGGGATGCTGGTATGTTCTTGCAATACAAAGAGTGGCTTCATGTCTAAGGCAGCAGT
GTGAGCGCAACCCTTCGTGTAATCTATCTTTGTCTTGCTCAGAGGAGGTGTGTTATCAGTTTCTGTTGCC
AACAGGAACTGTGGGAAATCCATGTGCTGGGAACTCAACAACAGTGACCAGGAAGCCAATGTGTTTGGAT
GTCAATGGACCATTTCCATATGGGATATACCAATGGGCACTTCCTGTTGTTTCTAGCAGATCCGTCACTG
TTAAGATTCTTTACCCCATCTTTTGGGGATTGATGACCCTTAGCACATTTGGCAATGACTTAGAACCAAC
AAGTCACTGGCTGGAAGTTATTTTCAGTATATGCCTTGTGCTTAGTGGATTGATGCTCTTCACTTTGCTG
ATTGGTAACATTCAGGTGTTTTTACACGCGGTCATGGCAAAGAAGCGAAAAATGCAATTAAGATGTAGGG
ATATGGAATGGTGGATGAGGAGGAGACAATTACCATCACAATTAAGACAAAGAGTTCGCCACTTTGAACA
CCAGAGATGGGCTATGATGGGTGGCGAAGATGAGATGGAACTTGTAAAAGACCTGCCAGAAGGACTACGA
AGGGACATCAAACGCTTTCTTTGCCTTGATCTTATTAAGAAGGTTCCTCTGTTCGAAAGTTTGGATGATC
TGATTCTAGATAACATTTGTGATCGCGTTAAGCCACTTGTGTTCTCTAAAGATGAGAAGATCATAAGAGA
AGGAGATCCAGTGCACAGGGTTGTGTTCATTGTTCGTGGACGTGTAAAAAGTAGCCAAAACCTCAGTAAA
GGAGTGATTGCCACAAGCATACTTGAGCCTGGAGGCTTCTTTGGAGATGAACTTCTTTCCTGGTGCTTAC
GCCGTCCCTTTATTGACAGACTTCCAGCTTCTTCCGCAACCTTCACTTGCATTGAATCTACAGAAGCATT
TGGCTTAGATGCAAACCACCTTCGATTTATCACGGATCACTTCAGATACAAATTTGCAAACGAGAGGCTG

AAGAGAACAGCAAGGTATTATTCATCCAATTGGAGAACCTGGGCTGCTGTGAATATACAGTTAGCTTGGC
GACGTTACATGATGAGGACTAGCCGTCCCACTATACATGTGATCGAAAATGGGGATAATGATCATCGTCT
TCGCAAGTATGCTGCAATGTTCTTGTCAATCAGACCACATGATCATCTTGAATAGTGATAATACCTTTGA
TACTATATCCTCGTTTGGCATATTTTTCGACAATAAGCCAGATTTGTGTACAATGACATTGATTCATTGA
ACTTTAATATCTGATATGAAGGGCCCGGGAATTATTTGCTCAAATAAATTTTATATGATTTTCCCCGTCT
TTGTAATGAATCTCATTGGATTTTGCCACCCATTTTCACCTCAGAATCAAATCTTGTTGTTGTAAA

>XM_002323847.2 *Populus trichocarpa* hypothetical protein
(POPTR_0017s12570g) mRNA, complete cds
AACAGATAAAACAAAAAAACTTTGACTGCAAAATGCTCCAACCACACAGCAACATCACTTTCGTGTAATG
TTCCGGACAGCGCACCCTCTTCGTACAGTACCAGACATATTAGAACTGCTTCCGATACTTCCAGCTGCCT
CTAAGCCATTTCTCTCCCTTTCTCTCACCATTTCATTTTATGTCGTTCTCTCTCTCTAGCTGGTTTCGAC
AAAGTAGAGGTAGTGTCAAATATTGCAATGAAAGCAAACACAGCGGCAGCGGCAGCTCCAGCAACAATGC
TGTTTTCGATTCCACGGAATGTTATGCTTGCACTCAGGTTGGTGTCCCTGTGTTTCACTCCACAAGCTGT
GACAGTGTCCACCAACCCGAATGGCAAGCCTTAGCTGGATCCTCCCTCGTACCTATCCATGCCAAATCCG
ATCCGATATCCAAACCTGCCCGCTGTCAAACCCCGAACACAAAGGGCCCGTTTGGGTCCATCCTGGATCC
ACGTAGCAAGTGGGTCAAGAAATGGAATAGGGTGCTGCTGCTGACCCGTGGCATTGCGCTGGCCATTGAT
CCTCTTTTCTTCTACGCATTGTCACTGTCGATTGGAAAAGGTGGGGCCCCTTGTTTGTATGTGAATATTG
GGTTTGCAGCAATCGTGACAGTTGCTCGTTCGTGCGTGGATGCGGTGCATCTTTGGCATCTATGGCTGCA
ATTCAGGCTGGCGTACGTGTCGAGGGAGTCCCTTGTTTTTGGGTGTGGGAAACTCGTGTGGAACGCACGT
GCCATTGCTTATCATTATGTGAGGTCACTGAAAGGATTTTGGTTCGATGTTTTTGTTATCCTGCCGATCC
CCCAGGCAATATTTTGGTTGCTGGTACCAAAACTAATAAGGGAAGAAAAGATTAAGCAGGTCTTGACGAT
GTTATTAGTGACTTTCTCATTCCAATTCCTTCCCAAGGTCTACCACAGCTTTTGCTTGGCTAGAAGAATG
CGGAAAGTCACAGGTTATATATTTGGCACAATTTGGTGGGGTTTTGGACTCAATCTCGTTGCCTACCTAA
TTGCCTCTCATGTCACAGGAGGATGCTGGTATGTTCTTGCAACAGAACGAGTTGCGACATGTCTCAAGAA
ACAGTGTGAAAGAAATGGAAACTGCGATCTCACTTTGCAATGCTCGATGAATGTTTGCTATCAGTTCATG
TATCCAGCAGATAACTATGGAAACCCTTGTGGCAGAAACTCAACATGGATTGCTAAGCCGTTGTGCCTGG
ATGATAATGGACCATTCAATTATGGGATTTATTCGCCAGCTCTTCTAGTTGTATCCAGCAATTCTCTAGC
AGTCAAGATCCTTTATCCCATATTCTGGGGCTTGTTAAACCTTAGCTCTTTCGGCAACGAACTCGCTCCA
ACAAGTAACTTGGTAGAAGTGATGTTCAGTATATACATTGTGCTGTGTGGTTTCACTCTTTTCACTTTAC
TGATAGGAAACATCCAGGTATTTTTGCATGTGGTCATGGCAAAGAATAAAAAGATGCAGCTAAGACGCCA
AGATGTGGAATGGTGGATGAGGAGAAGACAGTTGCCAACTGGTTTAAGACAAAGATTTCGACATTTCGAA
CGCCAAAAAATGGAGAGTCATGGGAGGAGAGGACGAGATGAGTTGGATTGAAGAACTGCCCGAAGGACTCC
GGAGAGACATTAAACGCTATCTTTGCCTAGACCTCATAAAAAAGGTGCCTTTGTTTCACAACTTGGATGA
TCTTATCCTTGACAACATCTGTGATCGTGTCAAGCTCCTTGTCTACTCAAAAGATGAAAAGATTTTAAGA
GAAGGAGACCCTGTGCTAAGGATGGTTTTTATTGTGCACGGACGTGTGAAATACAGCCAATGCCTTAGCA
AAGGCATGGTAGCCACAAGTGTGCTTGAGCCAGGAGGCTTTTTAGGTGATGAGCTGCTCTCGTGGTGCCT
TCGCCGCCCATTTATAGACCGCCTCCCAGCCTCATCCGCAACTTTTGTCTGTATGGAACCAACAGAAGCA
TTTGTTCTGGATGCATATGATCTGAGATATATCTCAGAACACTTCAGGTACAGATTTGCCAGCAAGAGAC
TCAAGCGAACAATGAGATATTACTCGTCCAATTGGCGAACATGGGCAGCCGTGAACATTCAATTTGCTTG
GCGACGATACAGGATTAGGAAGAGGGGACTAGCGACTCCTGACATGGTAAATGTAAGCATGGAGAACCGT
CTTCGGCTGTGTGCTGCAATGTTCATGTCGCTGAGGCCACATGACCACTTCCAATAAATTAAATGCAATT
GATGCTGCTATTTTGGCCGGTCTTTTCCCTTCTTTAATAAGATTTAGACACTAAATCTTCCATGAAAATT
GTAAGATCAGGACTATACACAGCTTAAGAGCTAGCTTGTGCTATTCTAATGTTCTTTCTTCAAGAGCTCG
AAGTTTATGGATTCATCAGTGGGTAATT

>XM_006373283.1 *Populus trichocarpa* hypothetical protein
(POPTR_0017s12550g) mRNA, complete cds
CACCCAAAACCCCACACCTCCCCTCTCATATCACCAGCTCTACTACTAGTAACAACAACTAAGCCTCTCT
CTCTCTATAAATATTCCTCTCTCCTCTTTTACTCTTTTGATTCTTTACCGTCTATCTCTGTCTTCCCATG
ATTCCAATCTTCCGATCATGCCTTCCCAGCCCAACTCCCACTTCTCCCTTCCAAGGTGGATTGGATTACT
GTGTCACAAGAACAGCCAAAGCGAAACTGGTGACAATAGTGACAACAGCAACCTTAACAGTACCAACAAC
ATTGACGACAGCAACCCAATCTCTAACTCCATAGAATGCTACGCTTGCACACAAGTAGGAGTCCCTGTCT
TCCACTCCACAAGCTGTGACCAGGCCCACCAGCCTGAATGGGAGGCCTCCGCGGGCTCATCATTAGTCCC

CATCAAAAACCGGCTCGGTTCAAGAAAGAGCCCAGCTAACCGAGCCCAATCTCGCCGGCCGGCCGGTCCA
TTAGGAACCGTTCTCGATCCACGTAGCAAGCGCGTACAGAAATGGAACCGGGCTTTCCTGCTAGCACGTG
GCATGGCTTTGGCGGTCGATCCCTTGTTTTTCTACGCTTTGTCGATAGGGAGAAACGGGGCCCCTTGCTT
GTACATGGACGGTGGGTTGGCCGCAATCGTGACCGTCCTTCGCACGAGTGTGGATGCTATACACTTGTGC
CATCTGTGGTTACAGTTCAGGTTGGCTTATGTGTCAAGAGAGTCTCTTGTTGTGGGGTGCGGGAATCTGG
TGTGGGACGCACGTGCCATTGCATCTCATTACGTGAGGTCTCTTAAGGGCTTTTGGTTTGATGCTTTCGT
CATTCTTCCCGTTCCTCAGGCTGTATTTTGGTTACTTGTACCGAAATTAATCAGAGAAGAGCAGATTAAG
CTCATAATGACAATACTTTTGTTGATCTTCTTGTTCCAATTCCTCCCTAAGGTGTACCACTGCATATGCT
TGATGAAAAGAATGCAAAAGGTCACAGGTTATATATTTGGCACCATTTGGTGGGGCTTCGGCCTTAATCT
CATTGCCTACTTCATTGCCTCTCATGTTGCAGGTGGATGTTGGTATGTCCTCGCAATACAACGCGTTGCT
TCATGCCTCAAGCAGAGTTGTGAGAGTAGGCCTAATTGTGATCTCTCTTTGGCTTGCTCGGAGGAGGTTT
GCTATCAGTTCCTGTTACGAGCAGGCACAATTGGAAATCCATGCGTTGGTAACACAACACATACTGTTAG
AAAGCCTATGTGCCTGGATGTTAATGGAGCATTCAATTATGGGATTTACAAGTGGGCTCTTCCAGTCATT
TCTAGCAATTCTTTGTCCGTGAAAATTCTTTATCCCATTTTCTGGGGTTTAATGACCCTCAGCACCTTTG
GCAATGATCTTGAACCAACAAGTCACTGGCTAGAAGTAATCTTCAGTATATGTATTGTTCTCAGTGGTTT
AATGCTCTTCACTCTACTGATTGGGAACATCCAGGTGTTCTTGCACGCGGTCATGGCAAAGAAGAGAAAA
ATGCAGCTGAGATGCAGAGATATGGAATGGTGGATGAGGAGGAGACAGTTGCCTTCTCGTTTGAGACAGA
GAGTTCGCCATTATGAACGGCAGAGATGGGCAACCATGGGAGGTGAAGATGAGATAGAACTAATCAAAGA
CTTACCTGAAGGACTCCGGAGAGATATCAAGCGCTATCTTTGCCTAGATCTTATCAAGAAGGTACCTTTG
TTCCACAACCTGGATGATCTTATTCTTGACAACATCTGTGATCGGGTTAAGCCCCTTGTTTTTTCTAAAG
ATGAGAAGATAATCAGAGAAGGAGACCCAGTGCAAAGGATGGTGTTCATTGTTCGTGGGCGTATAAGGAG
TAGCCAGACTCTTAGCAAAGGCATGGTGGCAACTAGTGTGCTTGAGCCAGGAGGCTTCCTGGGTGACGAG
CTGCTATCCTGGTGTCTTCGCCGTCCATTCATAGACCGACTTCCAGCCTCATCAGCGACATTTGCTTGTA
TTGAATCGACAGAAGCATTTGGTCTTGATGCAAACCATCTTCGGTACATCACAGATCACTTCCGCTACAA
ATTTGCCAATGAAAGACTTAAGAGAACTGCAAGATATTACTCATCAAACTGGAGAACATGGGCAGCAGTC
AATATACAATTCGCTTGGCGACGTTACAGAATGAGGACTAGGGGACCGGTGATTCCTGTAACAGAAAGTG
GAGGTACTGACCGCCGGCTCCTGCAGTATGCTGCAATGTTCATGTCAATCAGGCCACACGACCACCTTGA
ATAAACCATTGCTAGCAGCTCTTCAGTCATTGTTTCAATAAAGTCTTGTTTCTGTAGGCGGAAAATCCTT
CACCGGAAAACACCTACACGATGTTGATGTTGGGTGGTTTAACTGTTTATATAAACAGTTAAACATTTTA
ATTTATATCTTTGCCCCTTGGCTCACCTGGTTTGCACGTGTAAACTAATTGGAAGGTCGCAGGTCAAAGT
TACACTGGGAACATTACTGGGTTTTACGTTATAAATGACATCATGTTTCTACTTTTTTAT

>NM_121545.5 *Arabidopsis thaliana* Cyclic nucleotide-regulated ion channel family protein (DND1), mRNA
ACGTATCACACACAAATCTTGCGGCTTTGCCTCTCTATACAAATCACTCTTCTCTCTCCCAATCACTCCC
TGCAAATTTTCTTCTCTCCCTCTCCCATGGTGGTTCCTCTATTTCAATCATGCCCTCTCACCCCAACTTC
ATCTTCAGGTGGATTGGACTGTTTTCCGATAAGTTCCGTCGACAAACGACTGGGATCGATGAAAACAGTA
ACCTCCAAATCAACGGTGGAGATTCGAGCAGCAGCGGCAGCGATGAGACGCCGGTGCTAAGCTCCGTCGA
GTGTTACGCTTGCACACAAGTAGGCGTCCCAGCTTTCCATTCAACTAGCTGCGATCAAGCTCACGCGCCG
GAGTGGCGTGCCTCCGCCGGCTCTTCTCTAGTTCCGATCCAGGAAGGATCTGTCCCTAACCCAGCCCGAA
CCAGATTCCGACGTCTCAAAGGTCCGTTTGGTGAAGTTCTCGATCCTAGGAGCAAGCGCGTGCAGAGATG
GAACCGCGCGTTGCTTTTAGCTCGTGGGATGGCTTTAGCGGTGGATCCGCTCTTCTTCTACGCGCTTTCC
ATCGGCCGAACTACCGGACCGGCGTGTCTTTACATGGATGGTGCGTTCGCCGCGGTGGTCACGGTGCTCC
GCACGTGTCTCGATGCTGTTCATCTTTGGCACGTGTGGCTTCAATTCAGACTGGCCTACGTCTCGAGAGA
GTCGCTTGTCGTTGGTTGTGGGAAGCTCGTTTGGGATCCACGCGCCATCGCGTCTCACTACGCACGCTCT
CTCACTGGCTTCTGGTTTGATGTTATCGTCATCCTCCCTGTCCCTCAGGCAGTGTTTTGGTTAGTTGTGC
CGAAACTGATAAGAGAAGAGAAGGTTAAGCTGATAATGACGATTCTGCTGCTAATATTCTTGTTCCAGTT
CCTCCCCAAGATTTATCACTGCATCTGTTTGATGAGAAGGATGCAGAAGGTCACTGGTTACATTTTTGGA
ACTATTTGGTGGGGTTTTGCTCTTAATCTCATCGCATATTTCATCGCTTCTCATGTTGCTGGGGGATGTT
GGTATGTTCTCGCAATACAGCGTGTTGCTTCTTGCATAAGACAACAATGTATGAGAACCGGGAACTGCAA
TCTGAGTCTGGCTTGCAAAGAAGAGGTCTGTTACCAATTTGTGTCACCGACAAGCACAGTTGGATATCCA
TGCTTATCTGGAAACCTTACCAGTGTGGTCAATAAGCCTATGTGCTTAGACTCTAACGGACCATTCCGAT
ATGGTATCTACCGTTGGGCACTTCCAGTCATCTCCAGCAACTCTCTTGCGGTTAAGATCCTTTACCCCAT
CTTCTGGGGCCTAATGACTCTCAGCACATTTGCGAATGATCTTGAGCCCACAAGCAACTGGCTCGAGGTT

ATTTTCAGTATAGTTATGGTTCTAAGTGGCTTGTTACTTTTCACGCTGTTGATAGGAAACATTCAGGTGT
TTTTGCATGCGGTAATGGCGAAAAAAAGGAAAATGCAGATACGGTGTAGGGATATGGAATGGTGGATGAA
ACGTAGGCAGTTACCTTCCCGGTTAAGACAGAGGGTTAGGCGATTTGAGCGGCAGAGATGGAATGCCTTG
GGTGGTGAAGACGAGCTAGAACTTATACATGATTTGCCTCCGGGTCTTCGAAGAGATATCAAACGATATC
TTTGCTTTGATCTCATTAACAAGGTGCCATTGTTCAGGGGCATGGACGACTTGATCCTCGACAACATTTG
CGATCGGGCTAAGCCTCGAGTCTTCTCTAAAGACGAAAAGATCATCCGTGAAGGAGATCCTGTACAGAGA
ATGATATTCATCATGCGTGGACGAGTCAAACGTATACAGAGCCTAAGCAAAGGCGTCCTAGCCACTAGTA
CACTAGAACCAGGCGGTTACTTGGGCGACGAGCTACTCTCATGGTGCCTACGTCGCCCGTTTCTGGACCG
TCTTCCCCCTTCCTCAGCAACATTTGTCTGCCTAGAAAACATCGAGGCATTCTCCCTCGGATCCGAAGAT
CTTAGGTACATTACCGATCATTTCCGTTATAAATTCGCGAACGAGCGGCTTAAGCGGACCGCAAGATACT
ATTCCTCAAACTGGAGGACGTGGGCAGCGGTAAATATTCAGATGGCGTGGCGCCGGCGTAGGAAAAGAAC
CCGTGGTGAAAACATCGGCGGTTCGATGAGTCCTGTGTCGGAGAATAGCATTGAAGGTAACAGTGAACGC
CGGTTACTTCAGTATGCAGCTATGTTCATGTCCATTCGACCGCATGATCATCTCGAATAATAATAAAAAG
TTCCAATTTTATATCGGTCCAAATAATTGTGTGTTCATTGTTCCTCCTCAATTGTTTACCTTTACTTCTT
TAAAAAATCTTTCTTGGAATGATACTTCAACCTCTTACGGTTAAGTTCAAAGTAGATTTTTTGAGCAACA
AAATCTTGGAGGAGTTTATCTTGGTAATTTTTTATTTTTTATTTTTTATTAGCAGTAAAAGTAATTGTCT
TAATAAGTATAAAC

>AF280939.1 *Arabidopsis thaliana* DND1 (DND1) gene, complete cds
GAATTCGCCTTGCGTACCCTACTGGCGGACGGGTATAAATAAGGAATAGAAAGTCTCGGGAAAAAGGGGA
CGTTTTTCGGGACCTGGAGAGAGGATAGCACAAGTCAGCTCGCCCAGATCATCCAGTTCGCCCAGAAGAG
CCAGCTCATCGATAGAAGACAGCTCGTCGAGAGAAGTCGCCTCGTCAAGATCATCCAGCTCGCCCAGAAG
AGTCAGCTCATCGATACAAGTCGGCTCGTCGAGAGAAGTCCGCTCATCGAAGATCATCCAGCTCGCCCAG
AAGAGCCAGCTCACCAGAACCACCAGCTCGAAACAGCATCGCCTCGTAGCGCCCTCAACTATGCTCTCAG
TTCGCAACAAGCCTTTCAGCCCGTCGATTGTCCGGCTTCAAGTTCAGCTCGTGGATTAGAGATTGATTCT
TCACCTTCCCCAGAGACTCACCCGTACTTGTTTAATTCGTGCATTAACAGTCCTCTACTACATGCAATAT
TAGAATGAGTTTGAACAAAGTTATTCATCTACCATACCCATTTATTCATCTGCACTGTATCCTTAGTTAT
TTATTCCAATAAACGCTGAATTTTATCCAACCCAAAAATAAATAAATAGAAATTTGTGTTCGATGTGTGT
GGTCCGCTGATATTGGTCGTCACTCGAATTATTAATGCATGATATGGATTTGTATGACTGACAAATTTTT
GGAAACTTCACTGTCAGGTACGTGTGTACTATATATGACCACAAAAGTGCATTTTATCATATATATTAGT
AAACTAACTGTAGGTAATTTTTTTATTTTAAAAAGTGTAAGCAATAATGAAAATTGAAAAAATGAGAGTA
TTATGCTGCAAAAAAACATTATAGTAACTGGAGTATCTCTGTATCTGCAATCAACAAAGGCTTAATTACG
AGATTAGGCTGGTCGTATGTCTCCGTTGTTATATCATAGGGATATGGCGTACGTCAACATGTATTCAATC
CGTAATAAAAACACATATATAACAAGTATCAATTGACGGCAAACAAAATATAGTATATAACAAACATATA
TATCAATAATTGAGTCAAATACATCTATCAGTAAATGTTAATGACAACGACAAAAATCATTATCATGAAA
ATCTGTTATTTGTATAAATAATGAATGTTTGACAAAAAAAAGTATAATTAATGAATAATATCAAATGATA
ATATGCTTAAATCGTACATTCACTAAAATATCTTCTACTGTATTTATAGAGTTAGAATTATGACATCTTC
CTATATAAAGCAAGTAAATTTGTAAACTTATCTAAAAGTTCAATGATTAAACGTAAAGAAATTACATTTA
AAATGATGGGTCCATTTTAACGTAGCAATCCTCATGATCTGTACGGAATCCAATTTGTTCAATGTCTATT
TGTGGGCCGTTTTGCTAAGCAAGCCACCACATCTCTCAGACACTTGACAGCTCATCATCTCCCCCTTGTC
AATCCCGGTTCGGTTTCTGATTTAGTTCGATCTTCGATTTTAGTTATTAAACCGGAATCTCCCCTAACAA
ATAAACCGAATCCTACTAAGAGACGTACGTATCACACACAAATCTTGCGGCTTTGCCTCTCTATACAAAT
CACTCTTCTCTCTCCCAATCACTCCCTGCAAATTTTCTTCTCTCCCTCTCCCATGGTGGTTCCTCTATTT
CAATCATGCCCTCTCACCCCAACTTCATCTTCAGGTTTTTATACCACACAATTCCCATTTTTTTTCATAT
CTATATTCGTTCATTAATGGTGCTTTCACGCTAAAACACTCCCGAATTCTGAATATGTATTTTGTGTCTT
TTGCTGTTTACCCAGAGGTTACAAGCTTTACCTGAGATTGATTATTTTTCCCCGAGAAACACTTTTCCCG
GAAAATTCACTCGTTTTTTGAACTGTTCTATAGCTAACAAGTCACCATGCAATTCCTTATAATCTCGCTT
GAGTATGCGAAATCGTTCATCTAGCAACGAAATCGATTTTCATAATTGTGTACTATATAACGAATGTGAG
ATGATTCTGAATGATTTTTGGCGATAGATTGCTAATTCCTGATTACAATTTGAATATTTCAGGTGGATTG
GACTGTTTTCCGATAAGTTCCGTCGACAAACGACTGGGATCGATGAAAACAGTAACCTCCAAATCAACGG
TGGAGATTCGAGCAGCAGCGGCAGCGATGAGACGCCGGTGCTAAGCTCCGTCGAGTGTTACGCTTGCACA
CAAGTAGGCGTCCCAGCTTTCCATTCAACTAGCTGCGATCAAGCTCACGCGCCGGAGTGGCGTGCCTCCG
CCGGCTCTTCTCTAGTTCCGATCCAGGAAGGATCTGTCCCTAACCCAGCCCGAACCAGATTCCGACGTCT
CAAAGGTCCGTTTGGTGAAGTTCTCGATCCTAGGAGCAAGCGCGTGCAGAGATGGAACCGCGCGTTGCTT

TTAGCTCGTGGGATGGCTTTAGCGGTGGATCCGCTCTTCTTCTACGCGCTTTCCATCGGCCGAACTACCG
GACCGGCGTGTCTTTACATGGATGGTGCGTTCGCCGCGGTGGTCACGGTGCTCCGCACGTGTCTCGATGC
TGTTCATCTTTGGCACGTGTGGCTTCAATTCAGACTGGCCTACGTCTCGAGAGAGTCGCTTGTCGTTGGT
TGTGGGAAGCTCGTTTGGGATCCACGCGCCATCGCGTCTCACTACGCACGCTCTCTCACTGGCTTCTGGT
TTGATGTTATCGTCATCCTCCCTGTCCCTCAGGTGAATTTTCAGAACAACCTTCCAACTATTCCAATATT
ATGAAATTAACCCTAAACTTTTTATTACTATCAATAAAGCCCATATTTACATTTGAAAGTGTTACCTGTT
GATGAATCATGATTTGAGATGCAGATAAGATGTGAACTTTGTGTTTTTGCTTGTTTGCAGGCAGTGTTTT
GGTTAGTTGTGCCGAAACTGATAAGAGAAGAGAAGGTTAAGCTGATAATGACGATTCTGCTGCTAATATT
CTTGTTCCAGTTCCTCCCCAAGATTTATCACTGCATCTGTTTGATGAGAAGGATGCAGAAGGTCACTGGT
TACATTTTTGGAACTATTTGGTGGGGTTTTGCTCTTAATCTCATCGCATATTTCATCGCTTCTCATGTAA
GTCCTCTCAAGCTAGATATTGTATTTCTGCCTGAATATAATGTCCTGCAACAATAGGTTATATTCAGGTC
CTAAAACAAATGCAATATAACAAATCCCATGTGTATATATATAGTACTACTAAGGTCTGTCCTAAGATAT
GAAATTAAAGTTTGTTGTTTATGGAAATTCTGGTGGTTTCAGGTTGCTGGGGGATGTTGGTATGTTCTCG
CAATACAGCGTGTTGCTTCTTGCATAAGACAACAATGTATGAGAACCGGGAACTGCAATCTGAGTCTGGC
TTGCAAAGAAGAGGTCTGTTACCAATTTGTGTCACCGACAAGCACAGTTGGATATCCATGCTTATCTGGA
AACCTTACCAGTGTGGTCAATAAGCCTATGTGCTTAGACTCTAACGGACCATTCCGATATGGTATCTACC
GTTGGGCACTTCCAGTCATCTCCAGCAACTCTCTTGCGGTTAAGATCCTTTACCCCATCTTCTGGGGCCT
AATGACTCTCAGGTAATTGCTTTGTTTCTGAGCTTAAGGTTTACATCTTGGATATAAAAAAATTCTCTGA
TTGACATACTGAATCTGTTTTGGGGGTCGCGTTTTACAGCACATTTGCGAATGATCTTGAGCCCACAAGC
AACTGGCTCGAGGTTATTTTCAGTATAGTTATGGTTCTAAGTGGCTTGTTACTTTTCACGCTGTTGATAG
GAAACATTCAGGTAAACTATGCAAGAATATGTCTTTTCTATGATATTGTGTTTTCATGGAATGTAAGATT
TTAAAAAGCTTTTTACTTTACAATATTTTGAAGGTGTTTTTGCATGCGGTAATGGCGAAAAAAAGGAAAA
TGCAGATACGGTGTAGGGATATGGAATGGTGGATGAAACGTAGGCAGTTACCTTCCCGGTTAAGACAGAG
GGTTAGGCGATTTGAGCGGCAGAGATGGAATGCCTTGGGTGGTGAAGACGAGCTAGAACTTATACATGAT
TTGCCTCCGGGTCTTCGAAGAGATATCAAACGATATCTTTGCTTTGATCTCATTAACAAGGTACTAGGAA
GCAGACTTTATACAATCTTGTTAAGACTTGTGAAAGCAGTAAGTGTTAAGTCTCTGACATGTTTGATCTT
TCTCAGGTGCCATTGTTCAGGGGCATGGACGACTTGATCCTCGACAACATTTGCGATCGGGCTAAGCCTC
GAGTCTTCTCTAAAGACGAAAAGGTACTCATCCCATCATTTCTAAAAATTTATATTTCCAATATTTTGCT
TCGGTTTCGGTTTAAGAGTTTACTTGAAGAGTAAAATCTGAGGTTCTTCGATTTTGTTGATTGTCACAGA
TCATCCGTGAAGGAGATCCTGTACAGAGAATGATATTCATCATGCGTGGACGAGTCAAACGTATACAGAG
CCTAAGCAAAGGCGTCCTAGCCACTAGTACACTAGAACCAGGCGGTTACTTGGGCGACGAGCTACTCTCA
TGGTGCCTACGTCGCCCGTTTCTGGACCGTCTTCCCCCTTCCTCAGCAACATTTGTCTGCCTAGAAAACA
TCGAGGCATTCTCCCTCGGATCCGAAGATCTTAGGTACATTACCGATCATTTCCGTTATAAATTCGCGAA
CGAGCGGCTTAAGCGGACCGCAAGATACTATTCCTCAAACTGGAGGACGTGGGCAGCGGTAAATATTCAG
ATGGCGTGGCGCCGGCGTAGGAAAAGAACCCGTGGTGAAAACATCGGCGGTTCGATGAGTCCTGTGTCGG
AGAATAGCATTGAAGGTAACAGTGAACGCCGGTTACTTCAGTATGCAGCTATGTTCATGTCCATTCGACC
GCATGATCATCTCGAATAATAATAAAAAGTTCCAATTTTATATCGGTCCAAATAATTGTGTGTTCATTGT
TCCTCCTCAATTGTTTACCTTTACTTCTTTAAAAAATCTTTCTTTGGAATGATACTTCAACCTCTTACGG
TTAAGTTCAAAGTAGATTTTTTGAGCAACAAAATCTTGGAGGAGTTTATCCTTGGTAATTTTTTATTTTT
TATTTTTTATTAGCAGTAAAAGTAATTGTCTTAATAAGTATAAACGACGTCGTTAGTAAGTACGACGTTT
GCATATATCCTATATAGACTTTTTGTCCAAGCTCAGGACTTGCTAAAGCTCGTTCAGAGACTCCAAAAGA
GTTCATAAACAAAAACCTACTCCCAGAAAGTTCTTCCAAATCTCTCGCCATGGTTTCTCTCTTCTTTCGT
GAATTTTAGTTTTTCTAAATTTAATTATACTTTAAATTGCTAAGTCCAAACTCTTTCGCTTTTGATTCAT
CGGTAATACAAGTTTAAGAAAAAAAATTCTTATATTTATCTCTCAAATCGAGTTTCGAGTGTTTGATTTG
ATTCGTAAAATCAATGGCGACGAGCAAACCTCAAAGATCGCCGGCTGAAATTGAGGATATAATCCTCCGG
AAGATATTTTATGTAACCCTAACGGAATCAACTGATTCCGATCCTCGTATTGTTTACTTGGAGATGACGG
CCGCTGAGATTCTCAGCGAAGGTAAAGAGCTTTTGCTTTCTCGTGATTTGATGGAGAGAGTTCTCATAGA
TCGTCTCTCCGGCGACTTTTCCGACGCCGAACCGCCTTTCCCGTATTTAATCGGTTGCCACCGTCGTGCT
TACGACGAATCGAAGAAAATCCAGTCGATGAAGGATAAGAATCTGAGATCGGAGATGGAGATTGTCACTA
AACAAGCGAAGAAGCTT

>XM_003531792.3 PREDICTED: *Glycine max* cyclic nucleotide-gated ion channel 2-like (LOC100810471), transcript variant X1, mRNA
GAAGGAGGCAAATTACAAAAAAGAAAGTGGTCCCAAGTTATACAGAAAACGGCGAAAGAGCAGGGATGAG

TAGCGAGTACCAACCCCACCACCACTCCCTCAACGTTGCGTAACCGTAAGCTCCAATTCACTTGCAAATT
CATCCCTCTCATTCCCTTTCTCTTCTTATATACATACACTACTCTTCTCTCTCTTCCATATTCCACACAC
TCTAACCCTCCATGTAATGCTTCCTCTCCAATATCATCAACCATGCCCAACACCTCCTCTCTCCTTCGGT
ACCTTATCTAATTCTCTCCTCCTCTCAAGTAATCATGTATCAGTGGATTATCGTGAGAGTTCACATGTGG
ATTAGCAAAAAGTTGCGACCAAGGAATTCAATTAGCAATGGAGATAGCAGCAGCGACACCTTTCACAACG
GTGCCAGTACTGACACCGTTGTTGTGGATGACAATCCATTTAGCAGCGGGGTGGAGTGTTACGCCTGCAC
GCAGGTAGGCGTGCCGGTGTTTCACTCCACCAGCTGCGACAGTGCCTACCATCAGCTGCAGTGGGAGGCT
TCTGCTGGATCGTCTCTGGTTCCAATCCAGAGCCGGCCCAAAAAGGTCCTCGGCTTCCGGACCGTGTCTG
GTTTGACACGGGGGCCGTTCGGGCGGGTTCTGGATCCGCGGAGCAAGCGAGTGCAGAGGTGGAACCGCGC
GCTGCTGCTGGCGCGTGGGGTGGCGCTGGCGATCGACCCGCTGTTCTTCTATTCGCTGTCGATAGGGAGG
GAGGGGTCGCCGTGTTTGTACATGGACGGAGGGCTGGCGGCGATGGTGACGGTGGCGCGGACGTGCGTGG
ACGCGGTGCACCTCTTGCACGTGTGGCTGCAGTTCAGGCTGGCGTACGTGTCGCGGGAGTCGCTGGTGGT
GGGGTGCGGGAAACTCGTGTGGGACGCGCGTGACATCGCGTCTCATTACCTGAGATCGTTGAAGGGGTTC
TGGTTCGACGCATTCGTGATTCTCCCAGTTCCCCAGGTTGTGTTTTGGCTGATAGTGCCAAAATTGCTAC
GAGAAGAGAAAATTAAAATCATTATGACAATAATGCTATTGATTTTTTTGTTCCAATTTCTCCCCAAGGT
TTACCATAGCATCTGCATGATGAGACGAATGCAAAAAGTCACAGGCTACATCTTTGGCACTATTTGGTGG
GGCTTTGGTCTCAATCTCATAGCTTATTTTATTGCTTCTCATGTTGCTGGAGGGTGCTGGTATGTCCTTG
CAATTCAACGTGTTGCATCCTGCCTCCGGCAGCAGTGTGAGAGAACTAATGGATGCAATCTTTCTGTGTC
ATGCTCAGAGGAGATATGCTACCAGTCTTTGTTACCAGCTAGCGCGATAGCAGATTCATGTGGTGGAAAC
TCAACAGTGGTAAGAAAGCCTTTGTGCTTAGATGTTCAAGGACCTTTCAAATATGGGATCTACCAATGGG
CACTTCCTGTCATATCCAGCAACTCTTTGGCTGTAAAGATTCTTTATCCCATTTTTTGGGGTTTGATGAC
CCTCAGCACTTTCGGAAATGATCTTGAACCCACAAGCAACTGGCTAGAAGTGATTTTCAGTATATGCATA
GTACTCAGTGGACTATTGCTTTTCACATTATTGATTGGTAACATTCAGGTATTCTTACATGCAGTCATGG
CAAAGAAGAGAAAGATGCAGCTGAGATGTCGTGACATGGAATGGTGGATGAGGAGGAGGCAGTTGCCATC
GCGATTAAGACAAAGAGTTCGCCATTTTGAACGTCAAAGATGGGCAGCGATGGGAGGAGAAGATGAGATG
GAAATGATCAAAGACTTGCCGGAGGGGCTGAGGAGGGACATCAAGCGCCATCTTTGCCTCGACCTCATTA
GAAAGGTTCCTCTATTCCACAACATGGATGATCTTATTCTTGACAACATCTGTGACAGGGTGAAGCCCCT
AGTCTTCTCTAAAGACGAAAAGATAATCAGAGAAGGTGATCCTGTACCAAGGATGGTGTTCGTTGTCCGA
GGGCGCATAAAACGCAACCAAAGCCTTAGCAAAGGCATGGTAGCCTCAAGCATCCTTGATCCCGGAGGGT
TTTTGGGTGACGAGCTACTTTCATGGTGCCTTCGTCGGCCGTTTATCGACAGACTTCCGGCCTCCTCCGC
CACATTTGTATGTCTTGAATCAGCAGAAGCCTTTGGCCTTGATGCCAATAACTTGAGGTACATCACTGAT
CACTTCCGGTACAAATTTGCAAATGAGAGGCTGAAAAGAACAGCAAGATATTACTCATCCAATTGGAGAA
CTTGGGCTGCTGTCAACATTCAATTTGCTTGGAGACGTTACAGGCAGAGGACTAAAGGTCCTGTGATCCC
TGTAAGGGACACTAATGGAGGCACTGAACGCAGGCTCTTGCAATATGCTGCATTGTTCATGTCATTAAGG
CCACATGACCACCTTGAATGAAAGCTGGTGTGTCCATGTTTTTTCTCGTTATTGATTCTTGATCCTCAAT
AAAACTGTGTCCATGTTGAAGTTTCCGTGGAAGTCCTGGAATCATTCATGAAATGTATAAATAGGAAAAG
CAAGAAAGTGAGTATGAGTATTTGAAAAATCACTGAGGCCCTGTTCTTTCATTTGATTTTAAGTTTAGAA
TAATGATTTTTTCATGGGATTTGTATCTTGAGCAATCCCCTGACGAGGCTGTTATGTGCTTATTGGATGA
TTGGTTGAGAAGTAGTAGAGTCAGTTTTACAGTTTTGTTCTGGTATCA

MLO1;

>NW_019814903.1:171767-183696 *Quercus suber* isolate HL8 unplaced genomic scaffold, CorkOak1.0 scaffold_9400, whole genome shotgun sequence
TAGATTTGAATATTGCAAGAGACACAGACAGGAGTCGTAGTAGGGGTTGCAGGAGACTGAAAACTAACAC
ATGTTGTTGTAGACTGGTAGTGTGGTACAGACAGACAAGACTTTACATACATATGAAATTGTTGAAATTA
AGCACAGAGAGAGAGAGAGAGAGAGATTCGAATTCTAATCTTAAGAAAAGAGAGCAGAAATTAAGAAGAA
TTAAGGAGTAAGTAACGATGAGTGAAGGAGAAGGGGTTACAACGTTAGAGTACACTCCGACATGGGTGGT
TGCAGGCGTGTGCACTGTGATTGTTGCCATTTCTCTTGCCATGGAACGTGTCCTTCATTACGCAGGCGTA
ACTTTGAAGAGGAAGAACCAGAAACCCCTTTTCGAGGCTTTGCAAAAAGTGAAAGAAGGTCTTTCTCTTT
CTTTCTATTATTTATGTATATATTCGATTTCATTCTACGTTTGTTTGTTTCTTTTCTTTTTTGATTTCTT
AATAAATTTGAAGTCTGAATTCTTTGGCAGAGTTGATGCTTTTGGGGTTCATATCCTTGCTTCTGACGGT
GTTTCAGAATGTGATAAATAAAATCTGTGTCCCCGCCGACTTGACACGTCACATGCTTCCTTGTGAACGC
GAGGGACATGAATTTGAGGAATCAACCACGTGTGGCGAGGTTCGATCCCACACTGTCTCTTTAACTCTCT

TAAATCTAAATTTCGAGTGTGGTAATGGTTTTGGTTTGATTTTGGTCACTGTATATTGAATTGGTTTTGA
AAAGTTTTTATGGAAACTAATAAACTATAATAATGGCCAAAAGTCGAGGATTATTCTACAAGATGTTCTC
ACTTTATGGTAAACTTTGAATTCATTCATTCTATGATCTTAAGAATAGTACGGCGGATGGAGCCATACTA
TAATGGAGACAATTTTCTGATACTTGGATTCTTTTATTTATTTATTTTTATTCTCTTATGGTACACTTGA
ATTCATTGGAGACAATTTTCTAATACTTTGGATTCATATTTGTCAATTTCTGTTTGTAATTTTGTATTCT
TCTATGGTGTTTTCCTCTTACGGTTCACATGAGGAAGAACCCCTACGACTGGGAAATCTTTATCCTCTAC
GAGAGTCTTTTTTTTTTTTTTTTTAATATATATTAATTATTTATCAATATGTGATCTATGATGTTTCAAT
CAGTAAATGCCTGTTTGTTTTGTAGTTTAAAATCACAGTTTATGTACAACTTTTTAAGGCCTCATTCTTC
TAAGGTACGAGTACAAGTTCTACAACTGTACTCTTATATGACTAATTTTTTAAGCTCATCCAAACACACA
TTTGGGTACTACACATGCCCAAGGAAATAGTCAGATGCTCGAAGAGGTCTGGATACTCTTGTTTGTCAAA
AATCACACATTTGGGTATAACTGCATTCAAGTCTTCAATGGTGACTTTCTCAAGGTTCATGCTCTGGATT
TCAAGAACTCAATTGATGATACAATGCACGTTCAGGAGAGAGGGTTGATTTAATTTAATATGCTCGTTCT
TGTTGCTTTGAGTAGGACTAGCTGTCGTTGTCTTATCTGTTTCAAAATCTGGTTGTAACTTTCCTCTGAA
TTGAGAGCTTCTAGTCCATGAATCTATTTTGAAGTTTGATTTAGAATGGAGAGAAGTGGGGAAGGGAAGA
ATCAGAAAGAAGAGGAGACAACACACTGAACAGGAAATAAGTCAACACTTTCGCTTGTTTTGCATTTATG
TTATTGATTATTTTTAAATCAATAACCTTCATTATGTGTATTGCGTTCATATTGATATTACTGTTGACAG
AGATATCATTGGAAATTTAAAGACTTCATCAAGATATTCTGAACTGACCTTGAGGACATATATACACTAA
AATTTCCTTCAATTTTTGGAACACTTCACATATAGTTTGGTAATAGCCACCTCTTATCCTAGCCAATTTA
CTTTTAAATCTGATGCAATGAAATCAAACTACTTCATGGGGTATTCCATGCATTGCATTCTATATTGATT
CAGGCATTTAATGATTTTGTTGGTGCTCTCTCTTTCTCTCTTTTTGGTTGTGGAAATTATTGATGTTATG
TTGTTATTTATAGCTGCACAAATATACCATAATTATGTGCTCTAATTGTTTTTCCCCCTTTTAGAATAAG
GTACCATTATTATCAACTGAAGCGTTGCATCATCTGCATATCTTTATCTTCGTCCTGGCCATTGTCCATG
TGACCTTCTGTGTTCTCACTGTTGTTTTTGGAGGATTAAGGGTGAGCATTTGATATGATTATTCAACTTC
TTTGTCTTACACAACTACTGAATTATTAATATATATTTTTTTTTTTGAATATTTTGTTTGTCATTATATA
GATTCGCCAATGGAAACGTTGGGAGGATGAAATTGTCAAAAATAATTATGATTCTCGGCAGTGTAAGGAT
CATTATCAATAAGTTATGTTCTGTCTGTCTTGGCTATTTTTCATTCACTAGTGGATCCATGCGCTATGCT
TGTGTAGACTTTTGAAATAAGAAGGTTCAAATAAGTTAACCCAACAAAGCCTTATATGGTATTACCTTTC
TCCTGATTGACTACTTAAAGTAGCAAAGAGAACTGATTGACTCAATAAAGTTATTTGCCAGTTAATCGGC
ATCCTCATTGCTTCTAATTTATGACTTGTGAAAGTGCAGTTCCGAAATCAAAAACAGTTACCGATGTCCA
TCAACATGCATTTATCAAGGATCATTTTCTGGGTCTTGGTAAAAACTCAGTTCTTTTGGGTTGGTTGGTA
AGTTACTATCTTATTTCTATTAGATTCTACTTGTATCTTAAATGGAGGCTATGAGTTATATTTTTGAATA
GGTATGTACCATCAATTCTAACTGTAAAAATAATATAGAAAAATTTTCATCAAAGAAAAATGTTCAAAGA
TTTTCCTTTTCCATGCAACACGTGAGACTTCACATAAAATTATGCTTGCCTGCATGAAAAAAACGTTTGT
ATGGATTTTTCCCTTTCAATTTAGTCTTGCTTTCACCTGTAGCATTTCTTTTATAATATAAAACACAGTA
TGAACAAATGAAAAATAAAAGGAATCGACAAATTTTGCAAACTGGTGTGCATTTGTGATGGCATTACTGA
TCTAGTTTGCAATCACTAAATTTTTCCCAGGGTTGGTTACTTGCAAAAGCAATGTTCTCTTTTCCTGGCC
TATAATATTAATTATTAAACCCTGGGAGGGAAACCATATTTGGAGTTTTTCCCTTCTTGTTTGTTCTAAA
AGGAAAAATAACAGTTTTGCGGAAGATGTATGTTTGTCATGCGAATTATAAGAGTTGAGATAGAAAATAA
AAAGTTAGGGTTCAGTTTGATATGTTCCTTCTTTCAGGAGAGATGGGTATACCCAACTTTAGTAGCAACT
AATAATAACGTCTATCTAGTGTTGCTTTCATAAAAGCAATACGATGGAGAACTGAGATAGGCCCTTTTAA
GTAATGATTCTTAATAAACTACCACTTATCCCAAAAACTTAAGCTATTGGGAAAGAGTGAGTGAATTTAA
TCATTTAACCATTATTCTAATAATCCTAAAGTATGAACTGCAAAATATTTTAGATGGAGAACAGTTATTA
TCAGCTTGCCAAGTGTGTTTAACCATGCTTCTGACACTTTCTTTTTCCCCCTTCAGCATTCTTTTGGAAA
GCAATTTTATGGATCCGTGACTAAATCAGACTACATAACGCTACGACTAGGTTTTATCATGGTAAGGTAT
TTAATACGAGGAGGCTGTTCTCATTCACCAGTATAGTATTCTTCTGTTATCTCTTACGGGGAATCCTTTA
CTATATTTAATATTTCTGTTGTAGACCCATTGCAGAAGCAATCCCAAGTTTAATTTTCACAAATACATGA
TACGGGCCCTTGAAGATGATTTTAAGAAAGTCGTTGGTATAAGGCAAGTGACATATGAGAATACCATGTG
GTGTTAATGATTCTTCTGGCTTGCAGTATATTTTTATTTGTGAGTTGTGTCTTTTACCAATTGCTTACTG
ATTATCCTTGACTCACTGTTTGATCTTCTTTGTTGCAGTTGGTATCTTTGGATATTTGTAGTCATCTTCT
TGTTGCTTAATGTAAACGGTATGCTACTGCATTGAATAATAACTTACATGCTTGAAACATCAGTGAGCAA
TGTTGGTTTGTCTTTTTGCCCGCGTCTAATAATCCTGATAGCAAAATTTTTTTGGTCTAGTCAATTTAGG
TTGCTTGCTCAAACAGGTTTAGAGTTAGCTTGGAAACTCAAAGGGTAGGACAATTCATTGTTTTATACGC
ATCAGGAGTACAACAGTCAGAAAAAGAAAAAGAAAAAGAAAAAAAAAGACTAGTATATGTCGCATTCATC
CGAAAGAGCTCAATTTAGACATCTTGAGGGTTTGGTTCAATCATATGCACATGCTTTGCTAATTCCTCTA

CATTGGGTCGTTTTCTAGTATATGCTGTCAACTAGTGGAAGAAAGGGATTTGAACCTAAGTTCTCCTCTA
TGGGAGGATCAGGCAGTGCAACAGAGCCAGAAGACTTTTGGCATGGTGTTACACAATAAAAGAATTTATA
TGTGAGATGATTATAAGATATTATCTTAACAAAGTACAATAAAAATTCAGGTGTGCAATGAAATCCAATA
AAGAATCACAATGTACATTGCCTAAACAAGTTCACATTTGGCTCTCCATATTTGAATCTCTCTCTCCAAC
CTAACCTGTCCACATAATAGAGAAGGAGGGACACCCCCAAATCCTATCTTTCTCCTAAATCTTCCCTTCC
CCTTGCAAATGCTTCAAGCATGGTCTGAGGCATCCCCCAATGCACACCAAATAAAGAAAAACCCAGTGAC
CAGAATTCTCATGCCACAACATTGTAAAAGGAGGTGGTTCAACGTCTAACACAGGAAGGAAGCTATGATG
GGCTTTCATATCAATTTGATGCACGATCTTTAACAAATTTAGCACTATATTAGGTTCCTTGATGAGTTTT
TTGAAAGATTCTTGAATGGGGTTTGATGTTTAAGGATTTTTGGATGGTTGGGTCATAGAAAATTAGATAT
TGATTGGATATTGAAGGAATTTGATGAATTGTTGGTGTTACAACAATGAATTGAATCGCGGCTCTCAGTT
AGACTATTTACTCTTCTTTTGTCTTTTTGAACAATTTGAAGAAAAAAAGAAGGATAAATCCTAAGCAATT
GCACTGAATTTCCCCAAGCAATCACACTTAGAAAGAAGAAAGCAATCACTCCCTCAAAATTTGAGAAAAG
CGGTAGAAATTTTATTTAAATTGTAGTGCAGTTTCTGTTTTGAAGGTTAGATAAAGTTATTCCTAATCCT
TTTCCAACAAATACTAGATGTTCCAACAAAACGAAATAGGAACTAAAATCTGGAAACTTAAACTAAGAGT
CATGGGCCTCTTGTACATCCCATTATAGATCATTCCAGAAAATCTGGAAATTACCAAATTACCCCTACTT
TAGTTGAACTTAATTAAAACTAAACTATTAACTTTCTAACCTAAAGAAACTTAATACTAAGAGTATGAGT
TGATAGGAACTAAATTAGCCTGTCAAAGGTGACATTAAGACTCAACATCAAAGCTAGACTATAGTTTTGA
ATTTTGCAAATGAGCTCTATCTCAACTGGCACCTCCTCCCTCGATAAGTGCTAGGTGGAGGGTGAGGTCA
TGAGTTCAAGACTCATTGGGTGTGTGTGTAACTTACCAATAAATAAAAAAAAAGAAAGAATTTTTTTTGA
TCCTTGATCATACTTTTTGGAGAGCTTTTTGTTGGCAGCATTAGTGTGTCCTTATAATTCTAACATATGC
AAGATTGTGCTGGAAACAAGACAAGAAAAAGGGCTTCAAGGTTGGTACTGTTGCAATCACCAGTGATCAT
TCTTTCCCTTGGAAAATTATATGGAGATGAAGGCCCCTTGAGAGTAGCCTTCTTTGTATGGACTGTAGCT
TTGGGTAAATTTTTTACTATTGACAACTTAAGGAAGAGAAAGGTAAGGATTACAGATTGGTGTTATATGT
GCAAATGCAATGGTGAATTTGTTGATCTCCTTTTGCTACATTGCCCTTGTTAAAGATAAGAATAAAATAA
TATAGAGAAGTGGAAGCAAGAAAGAGAAAAAAACATAAGGGATTCCATGGTTCGGCCAACAGCCTACATC
TACAGAATAAAACCCTAGTGACTACGTCTTTACTGTATACTCAAGTCACTATTACAGTGAACCCTAATAG
GGTATATATAGAGACTAGAAGTTAGGGTTAGCTCAATATGGGCTTACAACATAACTGGGTTCCTTGGGCC
ATTACATCAACATATTAAACCCAATACTTTAAGAACCCTATTGCTTCAGACTTATGGTCCATGATTTTGG
GTTTGTTTGGAGTTTATTGGGTGATGCTAAAATTTGTTGTTGAGCTTATAGTTTGCTGGCAAAGTTGGTT
TGGTCGTCATCAGAATGGTCATATTTGGATGGTTATCCCCCATTGCTTGATGTGGTGCATTCAGGGGCGT
AGCCAGGATTTTTTTCTTGGGGGGGCAAGGTGAAATTATCATTTTGATTTGCAATTCAATGAAAAACTCA
AAGTATAGTATATTTATAACACAAGATTATTGATAGAATTTTTTTGTGGGTGTAATTCTATAGCACTCAA
ATATCAAAATACTAACAAGTTAATCGACCAATTGAGCATCTTAAGTCTTTTTTTCTTGAGTTTCTCCTAT
TCTTTTGAAAAACCATAAGTCCATAATATTCATAGAAATCAAATATAAATATACAACAAAGCTGTGGAAG
TAGAAGAAAAAGAAGAAGTTGGTATTTCTCAATATTTGCTTAGCTTTAAATAAGAAAACTCTTTGTTAAG
GAAGAGGGAGAAATTAATGAAAATGTGATTATGTGAGAGCATCCCATGGAAGAGTTAATGGAGAGTAACT
AAAGAGAGAGCTCCAAGTGATGAGGAGAGTCTCTTCAGTGGTTTTTAAGTGGAAAGGGATATTGTAATAA
TTTTGAGAAAGTGCAAAATTTAAGTACAATATTCTAAGTGTAATACATTAGGTTCTTCAATTAAATTCAA
GCACATGTTTAACATAACCAATAACATACAAATAATGTTATTATTTTTAAGGACAATTAAATTCAATACA
TATAATTTTAATTAGGTAATCTGATGTACTACACCTCAGTTTTATCCCTTCAAAAAAATTGAATAACTAA
AAACTAAAAAAATATTGATGTGGCTATCATGCTTTAGAGAAGAATGGAAAAGACAAAAAGAGTTAATTTT
TATGATTGCACAGTTGGGATTTGGGGTGGGAAGAATTTTACGAGTGACGTGTTGGAGGGGGGCCAATCTT
TTAATCTTGTGGGGGCCAACTCTTAACTTAAAATGATTCTAGCCTAGTTTTACCTATAATTAGAGAGGTT
TTAAAAAATTTTGGAGGGGCTATGGCCCCCCCAATGCATAAGGTGGCTCCGCCCCTGGGTGCATCTGTAC
GGAAAGGAAAAGTAGGATTTTTTTAAGATACCAAAAGTTTTATGCCTAATTTCAAGTTGTTCTTTTTTAG
AATTTTATTGGATTGGCTGTCAGCTGTGAGGAACCTTTCTTTATTTTCTATTGTTGATTTATTAGATTTA
TGCAATTTTTGTAACTGATTGTTTACCCAGTATACTTGGGTGGCTCTTTTTTTGATTTATATAATTTTAT
TACTTATAAAAAAAAAATTCTAACATAAGCAAGATCTATCCACAATGTCCTTTCCTTTTTTTTTTTGAAAT
TCTTATTGTCGATATTTTACCAAAGTAGCTGTCCCTCCAAGGAAAACTACATTTCTTGGTGCCTTTATAC
ACCAAATATTCTTCCATGGAAATTAAACTGTTAACGGCCCTTTAAAAGCCCGTACTATGTATGAAATTAA
AACTTCCTTTCTTAGAATGCAGTCGGCACACTCTCTATCTTTCCCTCCACCTGAAGGAAGATTAGAGTAC
TAACTTCATAAAATGTTAACTGACTCAATCTCCCAATCTTGCAAAGATCTCACAAATATGAAAGTCAATA
CTGGACTCCTTGACCGGGCATTCCTAGCTGTGATGCAACTAAACAATCCTTATCTATGACAGTCAAGAAT
TAGTTCAGAAATCTATCCTTCAGCTCAACATTAGACTTTAATCATACCATAACCACACTTTATCGACATA

ACTTATCTTAAAACAAACTCTGAAAAACATTTGTACAAATCCTTAATGCTCTTCCACAAACTCACATCAC
AGGAACCTCACATCTCTTCTGATGTCCACTCAACCCAAGCTCCCCCATATTTCACAGAAATAACTTGCCT
CCAAATTGATTTCTAGAAACCATTTAACTATGATTGATTAAAAGGTTTCATTTTACGAATTTCCAATTCA
ACTGCCTTAATAGGTGAACATTTGTATTCCAAACAACTAGATGATATTTAAATTTATCCTCAATGAATGC
TCACAAGAATACTTTTTAATTTCTCCAACTTATTGGCTACTCACAGTCATCCTCCTTTGCTTTTATGCCA
TAGGGTTACTGTTTTTAGGATTTGGACATCCATGTGATGGGCATTACTTATATAAACAATGGTATTTTCT
GTTTTACTTATGCAGGTTGGCATACATATTTCTGGATAGCATTCCTTCCTTTTGTTGTGAGTAGTCTTGT
GATCCCTTGCTCTAGTTTTGCATCCATGTGCATTTGCACAGTTTCTTATCTGTTTGCCCTTGCTTTCAAA
AGTGTGAAATTCTTAATTCATGCAATTTCAGTATCTTATGGATGGATGAGATTGAATGGGGAGATCTTTG
GTAATGTGGATTCATTATTAGTTAACATATTTTTAGCTAAAGAAACAAATGGAATAATCCAATGACATTA
GAATATTTCCAAATCCATTCACCCTGTGTTATATGATAATTTCATGCTTCTCTTTAATGTGAGCATGGCA
TCTCCCGGGCTGTCCAAGTAATTTGACCTGAAAGACATCTTTCAATACAATTATGTTAGAAAAGTTTACC
TTTAGTTTGATTGGAACATGTCAATAACATAAGAGCTGATGGGCAAGGCAAGAGCTTTAGCTCAAACGGG
TACCTCCTTGCCCTTTAAGAATAGGGTGGAGAGTGAGGTCATGGGTTCAAGACCCATCAGGTGTGTCTTT
TATTTACCATTTTGTATTTTAAAAAGATATGATGCACAAAGCTTGCTTACTTAATCTGCTGGATATGTTC
AAGTCAAAAACACCCAGCAAAAAGTATTGTCACGTTAAAACAGTTGATGTTAAGTCATTCTGAAATTGGT
TACTTTAATGATATCGATTGAAATTTCTATATCTGATTCACGGGTCTCCATACCATTTTTATCTGTTAAA
TGATTTTCTTCTTAATTTTTCTTAGAAGCCAAGTATAGATGATCAACCCATTTTTACCCTCTCTAATTTA
TATAAAATTATGGTTTATGTATTGGTTCTTCTCATTGTCATCCACCAATTAGATTCTCATCTTTGTACTG
CAACTGTTTTTTCTCTCTCTTTGTACTGCAACTGTTTTTTAGAGAAAACGGGGGGAAAATGGTTCCTGTC
TTGCTTAACAATTACCACTCAATGCAGAGATCCATTTGGTTTCTTTCTTCTGTTTGAAAAATGAAACCAA
AGTTATTACTTATGTAAATAAAATTCTGTTTTAAGAAAATTTGAAGGTTACATTAGATCTTTCGTTGGTA
CAGAAAAGATGAGGTGATTCGTGAAATCTACTACGTTTAGGGTACACAGATCTGCTCTGTGTGTATTCAT
CTTCAATGAAGTTCCTTTTTGTTCTGTTAAAAGGAAAAGGAAAAGGAAAAGGAGAAAAAAAGTATCATCC
TTCAGAAGTAATCTCATCAGAAACTTTCTAAAGATTGTCAGTCTAAGATTCGTTTCTTATCAAGTAATGA
TGTAGTGGAGTTGGCAATATTTTTGCTTAATGAGTTAAATGACTGACTCATTTCTTAACCTCAATAGACA
AGAAACACATTTTGACCGTCTGTAATGTAAAGATTTGCTGCCTGCAGTTAGTCTTTCCTTAAAATGAATG
CTGTTAGTAAGCATCTGCATTATAGACTCTAGACACTTGAGGTATTTTTATGTCTCCACAATCTCCATTT
TCTTGTTTGTTTCAACCTTGTTGCTCTAATTTACTAGTTTATGAATTCTGGCTGAGTGTCCTTGCATGGG
CTATTGTCCCCTATTCTTTTCTTTTGTTTTTTGGAGGAAAAGTGAGGGAGGGAGGGGGGGGGGGGGTTTG
TTTTCTCCCTAGGGGGGGGGGGGGGGGGGGGGGGGTTTGTTTTCTCCCTATTTTCAATGATAATTGTCAGGC
TAATTCTTAATGCACATGTTATTCAATTGGCATGTCATAGATGCATAATCTATGCGTAGAAATTTGTCCT
GTTAATCAGTTGTTCATATTAAATAGCATATACAGGCATGTATACAATCATGTCCACTTGTAAAATTTGC
GGCCTAGCACAACTTTGTACTTGCATGGCCTTTTTTTCGTTCCCTTTTCTTTTCACTTCATATGTTGCTA
ATTCATATAAATTTCAGCTTCTACTTGCTGTGGGGACTAAGCTGGAGCATGTAATTATCCAGTTGGCTTC
TGAGGTTGCTGAGAGACATGTAGCCATAGAAGGTGACTTGGTAGTTCAACCATCTGATGAGCACTTTTGG
CTTGGTCGGCCCCGCATCGTCCTCTTCTTGATCCATTTTATCCTTTTCCAGAATTCTTTTGAGATTGCAT
TTTTTTTCTGGATATGGGTAAGAAATTCTTCAACCACCATGCATCAAATCTATTTGTAATAAACAAGAGA
TTAGTAATTGAGATGGAACATGATTAACCTACTTCTCTGCATATCTAGGTTCAATATGGCTTCGACTCTT
GCATAATGGGACAAGTCCGTTATATTGTCCCACGGCTCATTATTGGGTAGAAGTGAAAGACCCCTGTTTA
CTTTTTCATATTGTTAGGTTCGAAGTATGTTGGATCTTTTATTTACTTAGTACTGATGTCTTTCTATACT
TTTTCTTTTGTTGTTTTTATTTAGGGTCTTCATTCAGGTCCTCTGTAGTTCCAGCACACTGCCACTTTAT
GCAATAGTCACACAGGTGCTTTTGAGTTGCTGTTTAAAAATTTATTCATCTTTTTTTTTAAAAAAATTAA
TTGATTTGTTTTAGTAAGCTGTTGATCTTACCTGTTCACTGCATTGAAATGAAGATGGGAACTTCTTTCA
AGAAGGCAATATTTGACGAGCATGTGCAAGTGGGCCTCGTTGGTTGGGCCCAGAAGGCAAGGAAAAAGAA
GGGTTTAAAGGCAGCTGGCAACGACTCTGGCCAGGGAAGTTCTCATGAAGCTACCTCAAGGGGAATTCAG
CTAGGATCTGCTTTTCGCAAAGCATCTGCGCCGGAGGAGATTCAGCCTGCAGCTGGTTCTGAAAATTCAA
AATGACTGATTCCATTTCTTAACTAAATCAGGATGGAGGTCTACATCTTCGTCTAAGTATGGTCATTTCT
GTAGATTTATATATACAGGTGATAGCGATAGTTATGTCACCCTTTGCTATGGGGCTGGAAGAGCTTGGGG
TGAAACTTATGTCATTTTGAATTTCTCTTAGGCTTAAAATGACTAGTTAATTGATGCATACATATTTTTG
AATGTCATTGTGTATTCTTTACTTACAAAAGGGAGGACTGAGAAGGGGGGTGGGGGGGTGTTCTTGAATG
AGTGCTGTGTGATTTGTGGCGGCATTGATTATAAACTTTTTGTAGCCTACCTATGCTCTTTGGGTACCAT
TAAAAGTCCATGGTGTTTTATTACTATTTA

>NW_019822261.1:1260-2253 *Quercus suber* isolate HL8 unplaced genomic scaffold, CorkOak1.0 scaffold_16758, whole genome shotgun sequence
CTACTTGCTGTGGGCACTAAGTTGGAGCATGTAATTATCCAGTTGGCCCATGAGGTTGCTGAGAAACATG
TAGCAATTGCAGGTGATTTGGTAGTTCAATCTTTAGACAATCATTTCTGGTTCCATAGGCCTTGGATTGT
TCTCTTGCTGATTCATTTCATCCTGTTTCAGAATTCATTTGAACTAGCATTCTTCTTTTGGATATGGGTA
AAACAATTAAAATTTAATATTCATTACATACTAATGACACAGAAATCTGAAATGAAAACTTTGGATTGAA
TTTCTACGATGCTTCGATATGTTGCAGGTTCAATATGGATTTGACTCATGCATAATGGGAGAAATCGGTT
ATATCATCCCGAGACTTGTTATAGGGTAAAATAATGATATTAATTATTCTATTTTTGAAACCTTGTGTTT
CTATTGGTGAACAAAAACTGATGTTAAATGCCAAATTTTATTCATAGGGCATTCATTCAGTTCGTCTGCA
GTTATAGTACCCTACCACTATATGTAATTGTCACACAGGTGAGTTGGATCATCACTAAGAATTCACCTTC
TTTATGATCAATTCATATATGTTGGGGTTTTAGGCTGATGAAGATTGCTGAATTCCTTAAATGTAGATGG
GAAGTTCATATAAAAAGGCTATATTTGAAGAACACATACATGAAGGGCTTTTTGGTTGGGCTCGAATGGC
CAAAAAGAACAAGGGATTGAGAAAGGCTGCTAATGGCTTGACGACGACTACTACTACTACTCCGGGT
TCTACTACTCATGCGTCTAGCCAAATGAATCCAAAAGAAACTACTCCTATAGCAATTCAGATGATTGAAG
TATCTGCAATGGAAGAAGGCAATGCAGGAGAGATTGCAACTGCAACTCTGAACATGGACATAGCTGAGGG
CATCATAGTCTCAATCAAAACTCTTGGCAGTTAGCCAGTCTTTTTTGCTAGTTGCAGTTCAGGTGAAGTT
AGAAATAGTTCCTA

>NW_017389749.1:c5830-716 *Juglans regia* cultivar Chandler unplaced genomic scaffold, wgs.5d, whole genome shotgun sequence
TATTCACATGGAAGAATTTCTGGGGTTTATATTCCAAAGCTCCAAAAAGAACTTCGGTCCTTTGATTGGT
GTCCCACTGTCCCCTCTGCTTAGCAGTTTCCCTTCTTTTCTTCTTCTTCTCCTCCTTTTACCGTCTTGCT
AATTGCCTACACGCCAATGTCAGCCGCAAAAATGATATTTTTACTTTGTACTAGACGAAAAATACCATAT
ATTATATACCTTTCAGGCATACACTCGAAGTCCCATTTGTTAACCATGGTTTGACTGACACCTATTTTGA
ACGGAAATATCTTATTAGTCTCTGCTTTTCTTTACATCGTTTTTCCAGGGAAAGGTCCCAATGTTATCTC
TTAAAGCGCTTCATCACCTTCACATATTTATCTTTGTGTTAGCTGTTGTTCATGTGATTTTCTGTGCTCT
AACCATCCTTTTCGGAGGAGCAAAGGTAAGCTGTCCCTATTCTTGTTCTTGTCTCCTAGAGTTTCTATTC
TAAAACCTTATTGTCAATTGTTCTGTTTTACGTTTTCAATGGCATAGATACGTCAATGGAAACAGTGGGA
AGATTCTATCTTAGGAAAGGAATATGATCCAGAAAGAGGTAATGCTCATTANNNNNNNNNNNNNNNNNN
NTATCTCTTGAAGCGCTTCATCACCTTCACATATTTATCTTTGTGTTAGCTGTTGTTCATGTGACTTTCT
GTGCTTTAACCATCCTTTTCGGAGGAGCAAAGGTACGTTGTCCCTATTCTTGTTCTTGTCTCCTAGAGTT
TCTATTCTAAAACCTTATTGTCAGTTGTTCTGTTTTACGTTTTCAATGGCATAGATACGTCAATGGAAAC
AGTGGGAAGATTCTATCTTAGGAAAGGAATATGATCCAGAAAGAGGTAATGCTCATTAAGCTAATATCTA
CAGACTCTCTCCCCTGTTATCTTCACCTGAACTCTTTTGGGCTGAATAACACAGAAATATGCGGAAAATT
CTTCACTATAGATGGACTAAAACAAGTTTACCCTGAAATGTGTGGATTTTCTTCAATATCCAACAGCTAG
TTGCCTCTGTTTACAATTTTTTTTTTTTTTGCCTCATGAAAATACCGTGCGTGCAGTTCTGAAAACAAAA
TTTACGCATGTCCGAGAACATGACTTTATCCGGGGACACTATCGGGGTTTTGGCAAGGATTCAGCTTTGC
TAGGATGGGTGGTAAGCTTTCGAATCCTTCATCATCAAGTGAAGACTAATACCAATTGTTATCACAACTT
CTAAGAAAGCGATTGGTCGATGTAATTGCGAGGATACTCGAAGATAGTACTTATGAACTGTTCTGATCAA
GATGTAACATTGTCAATTCTCCGCCTTGCTTTAAAGTGTCTCTTTTCTTCCGGAATATTAAACTTTCGCT
GCTGAAATTTATGAGAAATTACAGAATGTTCAGACTAACCACTTCCCCGTTAAATTTGTTTGATAAGAGT
TGGCTTATTGTAGGATTTACAACTTCGGAATGTTTCTTGTTTTATCCATATTCCTAATAGAAAATTGTTG
ATATTATCACATTGTCTGTAGACAAGATTTTAGGTATGTTTATAAAGAATAAGCAATCATCTCTTGTAGA
ACCGGTTTTATGAGATGAGTTAGGCCCATGAATTTTTTCATAGTATCAGGCCTGCCACAGGACGAATGGG
GGCCCACACTACTTATTCCGTGATAAGGACCAGAAAAAATACTAGCCTGAACGCGAGGGAGGGTGTTGAG
GAATAATCACACATTACTTGTAGACAAGGTCTTGCGCATGTTTATAAGGAATGAACAATCATCTCTTATA
GAACCGGTTTTATGAGATGAGTTAGGCGTAAGAATTTCTTCAAAAATTAACTTATTTTTTCAAAAAAATG
TATCAGAAATTTTTTATATGTTCCTTCATGCCAGACTCCACCTAATCTTTTTCCCTTTTTTTTTTTCTTAA
CCCAAGTTTCACTTAAATATTAACTACTCTGAAATACATAGGGAAAGGCAAGTTTCACTGCATATCAAGC
TGAAATTAGAACATAAGTATAAATCGCTCTGAACCTCAAATGATGCAGATAATCTTTCTTGTGCTGTTTA
CATGATAGTAATGATCTAGAATACATACTTCAGTTACATAAATATAGAAATTCAAACCTTTCAGTTTGTG
CGCCTGACTTATGTGATGCATCAAAAACTTGGGCAGTAAATAAAATGCACTATTGTTTTCTTCTTTTTCC
TGCCATTACTAACAACTGATTTCCTTTTTAATGTCCTCAATAATTTCAGAACTCTTTTTTCAAGCAATTT
TATGGGTCTGTGACCAAAACAGAATATGTGACAATGCGACTAGGCTTCATTATGGTAAGTGATATAAAGC

TCATTATTTATCCCTTTTTTCCTTAACTCTAAGATACTCTTCCTGAGTTCTTGTGTGTGTGTGTGTGTGT
ACACACACTTATATATACATATATATTGTAGACTCATTGCAGGGGAAACCTACAGTTCAATTTCTACAAG
TACATGATACGTGCCCTTGAAGCTGATTTCAAAAAAGTTGTTGGAATAAGGTTAGTTGTGATAAGGAAAG
ACAGGCGAAGTGCATAGCTTCATTAGTTTCTTTTCTATGTTTATTTTTACTGTTCCTTATGTGAAGTACA
TCTCATAAGGTTGGCATGGATGGTTCTGCCCTAGACTCAACTTCCTTTTTTCAAAAAGATTCTGTGGAAG
CTTGAAACCATGTATTCTGCTTGTTTTATTGTTTTTTTTTCATGAGTCCTTTTATCCTGCATGTGCTATC
TCTCCTTGACAGTAGTAACTGCTAATATGATACCTCGTAGTGGATAGATCTAACTAATTTTCTAACTTCA
TCAATTGCAGTTGGTATCTTTGGTTGTTTGTTGTCATCTTCTTGTTGCTGAACGTTTCTGGTAAGTGCTC
TCTCTCTCCCTCTCCCTCCCTCCCTCCCTCCTAGATGACTGCAGCATTTTTGTTAGTGACAGTATAATTG
GTTTTGGGCACATCGTGCGATGTATGGCCTCAGGCCATACTAAGAAACACACTTTTCTAGCTAGAACTTG
AATTTTATGGTGAACTATATGTCAAAATTCTTATTTCATACTGAACTGACTATGGAACAGTATATGACAT
GCTTATTCTCACTCATAATATGGTCCTATCACTGGCTTGGAAAGTGGAGTGCAAAGTTTAGAGGTAGATG
TTTGTTATCATCCTTTTTTGATGTACACAGTTCTCTGGGACACATTGGTCTATCATATTATTATTTAGAA
GAATTCCTGGATTGGTGTTCTGAGTTTAAAAGATTCTAATGAACATAAGGCCATGGTCTTGCATCTCTAT
TCTAAGCTCTTCTACATCAAGATCACCACTTCCAAAGCATCACGTGCTCTGATTTTTCTTGCAGGTTGGC
ATACATATTTTTGGATTGCATTCATTCCCTTCATTGTAAGTTGTCCATCTTAGTTTTCCTTTCAAATCTC
CCTTAATTTATGCACTTTGATTAATCACAATGTGTGAAAACTAGACCATTTGGGTTTTTTTTTTTTTTTT
TTAAAAAAAAATTCTAGGCGAAAAGGAAAGTCCAGCAAAGTTTCTATTCACACTTCTCCTTCCTCGAATTT
AACCTTTTGATGTCCACTAGCTTGCATGACAGATTGTATCAGAAGCCTCACACAGTTCAAATGCTCACAT
TCTTCAATGTTGAGGGTGTCTTTAACAATCTTCCTTTCAGGGTCAAAGTGTAGACACTGATGTATCTGAT
TTTTTTCTATTCCATTGACCATGGTTTAGTTGTTACTAATGAGTGCGTGATTCATGAAAATTGCACATTT
CACTGCTAAGATCATCAGATTACATGTTCATCTAGTAGAAGTCCCAGAGAACATTTGAGGATAAGGACCT
TAACATTTTAGTGATTTCTTTTCACAGCTTCTTCTGGCTGTTGGCACCAAGTTGGAGCATGTGATTTGCC
AGTTGGCCCAGGAGGTTGCAGAGAAACATGTGGCAATTGAAGGGGAGTTGGTAGTTAAACCTTCAGATGA
CCACTTCTGGTTCCATAGGCCCAAGCTTGTTCTCCTATTGGTTCATATCATCATGTTTCAGAATGCTTTT
GAACTGGCATTTTTCTTTTGGATATGGGTAAAACATAACTCAACGCTCATTACCTACTTACCAAAATCCA
TAAAATTTGAAACGTGAACTACAGACCGATCTTATCTTATGCAATCTATTGCAGGTTCAATATGATTTTG
ACTCCTGCATAATGGGAAAAATCGGTTTTGTCATCCCGAGACTGGTGATAGGGTATGCAAAACAATGATT
TACATATGTTCTATTCTTGGAACCATATCTCTATATTGAGGCACAAAATCTTATGATTTGGTTCTATGCC
AATTTGCATTCTCAGGGCATTCATTCAGTTCGTCTGCAGCTACAGCACCCTACCACTCTACGCAATTGTC
ACACAGGTTAGTTGGATCAGCATTGAAAACTTGTCTTCTTGTTTCAACATCTTTATGTTAGGATTCTTGG
CTGATGAAAGTTGTTGTTTTTATGGTTCCCTGTAACGTAGATGGGAAGTTCATTGAAAAAGGAAATATTT
CCAGAGCACGTACAAGAAGGACTAGTTGACTGGGCTAAACATGCCAAAAAGACCACAGGTTTGAGAAAGG
CTGTCAATGGCACTAGGCAATACGGCTCTACCACTACTCATGGATCTAGCCACGTGGTTGGTAGAGAGAC
TACTGGTTTGGCAGTTGAGTTGACAGAGGCAGGCGAAACAGAAACTGCAATGGAAGAAGGCAATGCAGGA
GAGATTGAACATGCGAATGTCTCCCATGAACACAAATGAATTGAGGCCCTATAGTCGTCCGAACCTCTTG
CTGAAGTTAGGGTATACTGTTTTCAAAATTTACTGAATACATGTATAAAGCTCATTGGCTGGCAATCAAT
AAGAAGAGCAATTGAAAGAAATATCTTCCAAGAATATACAAACATGTAATATTTTTCTGGCAAATAAAAC
AAATTGCCATCCAAGTTTTGGTTTGCTGTCGATACAATATGTTGTTTTAGTCAATTATCATGATCTTTCA
GATTA

>NW_017443107.1:c327155-313553 *Juglans regia* cultivar Chandler unplaced genomic scaffold, wgs.5d, whole genome shotgun sequence
CGGGGAATATAGTAGAGGGGTAACGCAGTGGGGACCGGGGGAGACACGGACAGACACAGAGAGACAGAGA
GAGAGAGAGGTGGGGAGAGGACTCAGAAGAGGAAATTGGAAAGGATGAGCGGAGGTGAAGGAGAAGAGGC
TACGTTAGAGTACACACCAACATGGGTGGTGGCCGCTGTTTGCACTGTAATCGTCGCCATTTCTCTTGCT
GTGGAGCGCCTTATCCACTTCACCGGAAAAAAATTGCAGCAGAAGCACCAGAAACCCCTCTTCGAAGCCT
TACAAAAAGTTAAAGAAGGTCTCCCACTTCTGCATTTTCTTCTTCTTCTTCTTCTTCTTCTTCTTCTTCT
CTCTCTCTCTCTCTCTTTTTTTTTTTTTTTTTTGTGAGTTTGGTACACTGAAATAAATATCTGTGGTGTTAC
TTGAGAGCAGAATTGATGATATTGGGGTTCATATCGCTGCTTCTGACGGTGTTTCAGAACACGATCTCCA
AAATCTGCGTCCCCGAGGGCTTGACCCGTCACATGCTTCCGTGTAAACTCGAGCAAGGAGGGCAGGAAGA
ATCCATTACTAATGCCACCACTGCCCATTTCGGGAGCTTCTTTGTGTCCACCATTTCGGGCAAAGCCAGG
AGGCTTCTCTCTGAGGAAGTTTCGTCTTCCAACACCAGTTACTGCAGCCGAAAGGTAACGTTAAGAGTTC
AGTGATTATGATTTTCGTACGTGATGCTTAAAATCGATTTTTTTTTTTTTTTTTTTTTTTTATGTATAA

AAATAACAATATTCGTGTCCCTGTATTTGTTTTGATTTGATAAAAGTAATGCACTGGTTTCACAAAAGAA
CAAATGGTGGCTATCGTAACGAACCATTTGTTTATAGGGATTCTAAGGAGAGCATATGATTCTTGTTAAA
TATTTAGTCATACCCATTTTTTAACTATATCTCTCACATATGGAACACTGGAATCCGCTCTATAATATTA
AACCGTAGCATGGTGGACTGGAACATGCCGTGCGATTGAATTAGAAACAGTTGAGGACAATCTTGTAATA
TTTTGAACGTCGTCATTTTCTGTATGTAGTTTGGTACTTATCAGTGATATATCATCTATGAAGTAGCAAC
TGCTAAAAGTTAACACAGCATCTAGTCTCTTCGAAGCCACTTTCTCATGTTTCAGCTCTGAATTTCTTAT
AGATAAATAGATGATGCAGTGAAAGTATGGGAGAAAGTATAGACAGTTTAAAGAATCACAACTAAGTTTC
TTTATGTAGAACGTGAACCATATGTGCCATATTATTGCTGCTTCTTGTCGACCTTGTCTTTGTTTTATCT
GTTTCCAAATCTGGTTTAACTTTCCACTGAAGTAAGAGTTCCTAGGCAATGAGTCTATTATTAATCTTGG
TTGAGAATGGAAAGGGAGGAAAGAATTAGAGAAAGCAGGAGGATAATCCTGAGCAGGATCTAACTAAACA
CTTACACTTGTTTTGGAGTCTTGGTTTCATATTGTGTGGCTTGGCATTAATTACTTCTTTTAAAAAATCT
TATGCATGGATAACCTTCACAAAGTTTTTTGCATCCATATGATTGCCTTGTCTTACACTAGCTTATTTTT
TGTAAAGTTTGAGAAACCAAAAACCTCCAATAACACTAAGTGAAACGATGTTTTAGTTTAGACTGTACAA
TTAAGGTTGAAAAGGGCTGGGTTGGAGTATAGATTTTCGGTGTGCAATTATTTAGGATTTTATTTTTGGT
GTGTGAACACATTCATAAAAATGATCAGCACTCACTTTATTGTGGGTGGGTTTCCAGATAAATTATTATT
TTTCCCTTTTCGATCATTCCACCTTAGTTACAAACATCTTTCACGAGTCACAATGCTACCTTTGTTGCCC
TCATCCTGAAGAAATTGGGGGAGATTAAGGACTTTCTTCCAACAAGCTCAGTGGGAGGCATTTATAAAAT
TATTTCCGAAGTCCCAGCCAACAAGATGAAGAGAGTGTTGGAGAAGGTTATATCCAAAACCCGGAATACT
TTTAATCATTGGGAGCAGTTTGGAAATCATAATAAAACATAAGTATTGAGGATTGCTTCCCATTTGTAAT
GTGGTCTATCTAGAAGGCCTTGTTTGTGTACGCAAGTATTCTCAGATATTTTCTTCCCAGATATCACTCA
AAGACAAAAACACTTTTCAATTTCAAATCTTCAACTTTTTCATTCAGTTATTACCTAATCATTACAAATT
TCCCAAACTTCTAAACCAAACACAAAAAACAATACATTTGAATTTATGACCTGTTGTTCTCATTTAAGTT
TATAATATTTTTATTCAACTTTTTCTCTCTCATTTTCTGAAACCCAATAAAGCATCTTAACTCAAACAAG
TTCACTACTATTCACAAACCATTTCATTACTATTCGCAGATATTTTGAGATCTTCTAAGTGTCCAAACGA
GCTCTAAGACAATGGTGGTATAAATACTAGCCAAAATGTTCAAAACCCTCTACACCTAGATAGAAGATCA
TAACAGCTCTTGTATTTCTACATTCTTTGAATTTATGACCTGTATCTTTCCTCTAAGTGGGTCTTTCTCT
TGTATACTTCATGTATATTCAGGTTGTGCTCCTTTGCGCTTCCACTAAATTGACTTGTGAAAAGGGGGGT
GTTTATTTATTTTTATAATTTGGTTTGTAGGCATTAAGAAGAACAGGAGTAGGTATCTTCTATTCCCTCT
TAAGATTTTGTGTGCAGTTGTTCTTAGATTAATTTCTTTATTAACTGAATCAGAATGGCTAATGAAAAGC
ATGTAATATCCTGGTTCGAATATAGGAAGGTGGTGAATGAGATCCCACATTACTTGGGAGGGAGAAGCTC
ATGCCTTTTATTACGATTCCAAAGGGCTTTAGATGTAATCTTGTCTAGTCATTTTGGAGTGTAAGCCTAG
ATATGGCCTGGGGCTTTCTTTAGGTCGTTATAAATGCTTTTAAACCTCTAATGTCCACTTCTGGCCATTC
CATCACAAGTGGTACAATTGAAGTCTCACACTTCAAAGAAAGTACAGGAAGGACAAAGGAGTTTTTGTCC
TTTGTATATTCAAGCTGCGGCTGTCTTTTACTTTTCTTTCTACTTATGCATGAAAATGTATGAAGTGGTT
GATTTCATGGTTGTGCGCTCTTTCCCGATTAAGTGAGATCCGCTATGGGTAAACAGAACTGTAGGGTATT
GTTTTTTCAATATTTTTTATTTGGCTTACACTGTTGACAGATATCTTATTGTTTTTCCCCTATTTAAAGA
TGTAATCATAAGTTGGAGTTGAAATCACAGGCATTCTGTTCAATAGTCAGATTTGCAATTTTCCTTTTCC
CTTATTTCTTCCTCTTCTTGTTCTACTTGTTTTTCTTCCTCTTCTTTTTTAATGTCATGTATATTCTGGT
CCTTGCCACCTCCTATCCTAGTCAATCAATTTCTAATTTTGACAATGAAATCAAACTACTGCATGGAGTA
CTTCATGCATTACATTTTATTGTTTATTTTTAGGGGGTTAATAATATATTGGTGTGTACTTTTTTGGTGG
TTTATTGTGATTATAGCTGCACAAATGGGGCAGAATTACGGGATCTAATTGTTTTTCCCCTTCTAGAATA
AGGTACCACTGTTATCTGTAGAAGCTCTGCACCATCTGCATATCTTTATCTTTGTCCTAGCCATTGTCCA
TGTGACCTTCTGTGCTCTCACTATGGTTTTTGGAGGTGCAAGGGTGAGCATCTGGTATGCTTCTTCTACT
GCTTTCTCTTATACAATAACTGGTTTATTAATGTTTTTCTCTTTCCTCTAAATGTTTCACAAATAGATTC
GCCAATGGAAAAACTGTGAGGATGAAATTGCTAAACAGAATTATGACCCAAATCAAGGTAATTATCATGT
GCGATGCTTCTGTAAACTTTCGAAAGAAAAAGGTTCAAGTCAGTCAACCCCATGAAGCCCTATAGGGATT
ACCTTCCTCCTGATTGACTAATTAAAATAGTAAAGAGAGCTGTCTGGCTGAACTTTCTCATTTCAGTCTC
AGTAAGATTATCTGGCTGCTATAATTATTAATGTTGTTGTTGTTGTTGTTGTTATATGCCCATTGCTTCT
AATTTCTGCTTGTGATGGTGCAGTTATGAAAACAAAAACAGTTACCCATGTCCACCAACATGCTTTTATA
AAGGATCATTTTCTGGGTATTGGTAAAAACTCAGCTCTGTTGGGTTGGTTGGTAAGTTACTGTCTTATTT
ATATAATTGATCTGTATGCTGTATCTTCTGTTGGATTGGGTTAGGTAATAGAATCTACCTGCCATATGCT
CTGTGAATGCTATAAGCTTGGGAAATAAGATCATGTTCAAAGATTTTCCTTGTTCATGCAACATGTGAGA
ATTGGCATAAAATTTTGCTTGCCTACATGAATGGGACTCTTGTGCATTTGAGATGGCATTTTATGTTTTA
GCTTAGAATTATTGGATTTTGTTCCTGGGGCAACCAGTAATTTTTTATATTGGTCCTCTTTTTTTCTGGC

ACATATTACCAAGCCTAGCTAGGAGGAACCCTGTTTGGGGATTTGTCCTACTTGTTTTGAAAGTGAGATT
ACCACTGGCTTTAAGAAGAGGGAAAGATCAGTCCTTAATGCAGCATTGATCTTATATATGATGACACCAG
ATGCACCTTGGTATTTCCATCTGCCAATCAAGGCTAGTGGAGTGAAGAGGGCCAAAAAACGTGAGCTGCA
TAGCACGAGCCTTATTGAAAGAAAAGGCACCTTAATATAGATTGTCTGGAAAGGAGGAACAACAGTGATG
CAGAGGAAAAAGTATGTCATAGGAATTATAAGTTATGAGATAAAGAACAAAATGGTAGGGCTTGCTTTTA
TGTTTTTCTCAGGAGGGATAGGTAACAACTCATAATAAAGTCTGTCAAGACTATCAGGCATATCATCCCC
TATGTTGATTTTTTAAAAGCTACATAGAGACTGATATAGGACCTTTCAAGTCATGATCCTTAGGTTTGAA
CCACAAAATATTTATGATGAGCATGCTGATTATCAGTTTGTTAAGTGAAATTGACCCGTTGACCATTCTT
CCAATCTTTTTCTCTCCTCTTCAGCATTCTTTTGTCAAGCAATTTTATGCATCTGTGACAAAATCAGACT
ACAAGACACTACGGCTAGGTTTTATCATGGTAAGATATCCAATATGAGGCGGCAGTTGACTTGTCGATTT
CCCGTATAGTTTTCCATTTTCTCTCGCAGGAATTCTTTTGTATATTTAATATTTCTGTGTAGACCCATTG
CAGAGGAAACCCCAAGTTTAATTTTCACAAATACATGATGCGTGCACTTGAAGATGATTTTAAGCAAGTT
GTTGGGATAAGGCAAGTGATGCCATAGAATATCTTGTGATTTTAAAGCATGTTATGGCTAGTAATTTATT
TTCATTCTACAATGGGATTCTTATTCTTAACCGTTATTTTTAAATCAAATAGTTCAGATTTTACTGCCTA
CACATTTATCATATTTAAAAAAAATTTAATTTATTTGCGAGTAAGGCCCTATTGTTGTATCTATTGATGT
CAGCAATCTTTGAAACATTTAATTTTTTTTTTTTTGTAAATTGCTTTTTATAGTAGTAAATACTGGTGGTA
AAACCTGGACCATTTGATAAGAAAATAAGGAATATTTGGTTGATAACCCCCTTGTGCAATTTTGTTCAGG
AATAGCACTTTGTGATCCCAAGTTTTGAATCAAGCCCTCTGTTATGATTTCCGTCCACTCTAGTTAATGA
AAAACATGTGGCAGTCCCATAGACTGTTATGTGGAAAATTAGTTTTTCTTATTTACAAAATAAATCAAAA
TAGGAAAAATTGGTCAGTAACTCCCTATACTTCTCTCCGATCGGGATTTTACCCATATACATCTTTTGTT
TGATGAATAACCCCTGTGCTATGAAAAACTTGAGATTTGCCCTCTCCGTTACTAATTCTGTCAATCTGGC
TTAATAGAAAACACATGGCAGTCTTATTGACAGCCATGTGGAAACTGAGATTTAAAAAAAAATATATTCT
AAATAATTAAAAGTACAAAGAAACAAATTTAAAATAAAAGGACGGGCATCCCCTCCCTGCCAGGCCACAA
GGGGGCTGTACCCGGGCAATTCCATGGTGGCCCATGCCTCGGGACCACCATGGGGTGGCTGGATTTGGCC
CCCTGTGGTAGCCCAGGTCTTGGTCATGGGGTGCATACTGAATTTTTTTTTTTAAAAAAAAAACTTTGGT
ATATTTTGGAATATGAATTTCCAAGTGGCATTATGTAAGATTTTCATGTGTTTTCTGTTAAGTGAAATGG
ATGGAATTAGTAACAGAGGGGCCAATTCTCGAGTTTGTAATAGCAGTGCGGTTATTATTAAAACAAATTG
GCAGGGGACGAATCTCAATCAGGGAAAAGCACATGGAGTTACTGATCAAATTTTCAATATTAAATTTATA
CTTTTAAAATAAAAATAACAGGAACAAAAGGAACGATGCTAGGCAGCTGGACCACCAGCCCCTTGGTAGC
CATGGCTTTTGCCTGCACCGGTTGGCTGAGTTGGTGGCCATAAGGAAACTAATTTTCCATGTGGCAGTCC
CACAGCTGACATGTGTTTTTTGTTAATTGGGATGAACGGAAATCCTAATGGAGGGGCCTTATTCAAGACT
TGTGATAACACTCTTTGTGTTATTCCTAAAGCAAAATGCACAAGGGGCTACGCTCAAATAGGCCTAAGCA
TAGGGGGTTATTGATAAAATATTATTAAAAATAATGTTTAGGATTTTAGAAACTTCCCTGTAGAGATAAT
GTAGAGGATCCTTACCTAAGTGTTAATTTTCTGGCTTGTCTTTTTTTCTTGCAGTTGGTATCTTTGGGTG
TTTGTGGTCATCTTTCTGTTGATGAATATCAACGGTATGCTAATGCTTTTCTGTAACTTGGATACTTGAA
ATCTCAATGAGCAATGATGTGGCATGCTTTGTTATCCTGTATCCACTTATATTGACGACAGAGAATTTGT
TTGAATTATTTTAGGTTGTTTTCTGAAAGAAGTTTCAACTTACTTTGTTAACTTGAGGGATAGAAAAGTC
ACAGTGTTAAAGGCTTAAGATACAACAGGAAAAAAAACTGATAATCAAATGCCACATTCAACCAAAAGAG
TTCAACTAAGACTTGTTTTGACCGTTTGGTTCAGCTATATTGATCATGCTATAACAATTCGTCTGCATTG
GTTCATTTTCTTCTTAAGGATATGCTACCAACTCCAGCCATTTCTTAATACCTTGGTTCATTGCTGTCTC
ATGCCTTCATCTTTACAATACTTTTCCTAACTAGTAATGTCATTGGTAATCATGTCAATTGATTACCCTA
ATGAACTTGGAGTCATCATCACCAAATTTTTGTCTTTGTTTCATTTTCTTGTCTGAATAATATCCTACGC
TGCTGTTTTTCCCCCTTTTAGTAATAAAAGAAGTTTGATTGTCCAGTAATCAGTTGATGGAAGAAAGGTC
TCATATATCAGTTGAGAAAGCATGGTATTATTATAATAGATTGGTGCTTTATGTGCGAAAGAAATGGAGA
GTCGGTGGACAATCTTCTTCTTCACTGTGATGTGGTTAAGGTTTTATGGGATGAAATTTTTGCTAGGCTT
GGCATTGCTTGGGTGATGCCTAGGAGGGTGGTGAATCTTTTTGGTCGTTGGAGAGGGATTCAGGGCAATC
GTCAAATTGCGGCTGTTTGGAAGATGGTGTCATTATGTCTTATGTGGTGTACTTGGAATGAGAGGAATGG
TCGCTGTTTTGACAATAGAGAATGATTGATGGGAGGGTTTAGAGATTTTTTTCTTCCATACATTGTTTTT
TTGGGCTTCAGCTATTGTATTGGATGGGACTAGGTTTAATGACTTTCATGCTGTTTTTCACAGCTCTTAG
CTTGTAATTAGGTGTTCTTCTCTGTTATACTTTCTATGTACTCAGGCTATGCCTAATTATGTGGATTAGT
AAATTCTTCTTATATATAAAAAAAAATTAGACTTTCTTATGATGCTGAGGAAAAAGTAACACTTATGTTGT
TAATGGTATCTACCATCTTCTCTTGCTTTTATGTCATAACGAGCTATATGAGGAGAATTACTTGCAACCA
ACTATGCATTCTGGTTTCACTTTTGCAGGTTGGCATACGTATTTCTGGATAGCGTTCATTCCTTTCATTG
TGAGTAGTCTTGATATGATCCCTCAAGTTTTGCTTCTCATGCAAATTTGATTATTTTCTTAACTGTTTTG

CAATTATTTAGTTTGAACCCCTCATACTATAAATACAAGCAACCTCAGTATATTATGGAGATTAAAAAGG
CAGGTGTTTGAAGTGATGCAAAATGAGTTAAAAAAACTTGTTCGACCAATAAATGAAATAATCCCAGTAT
ATATATGTGTGTGTATGTGTATATATATATATATATATATATATAGTATGCTTCCTAGTCAACTTACCCT
GTGTGATATAATAAATTCTTGTTTTCCATTCGTGTGACCAGGCCATCTTCCAGGCAGTTCAAGTAATTTA
TCAGAAGGGCAATTATTCAATACAAATTATGCTAAAAAAGTTTAAATAAAGACAGTGACAATAAAATAAG
ACGTGATGGGAAGTCAATGAGCTCTACCTTGAATAACACATTGCCCTTTAAAAGCTGGGTAGAGGGAGAA
ACAAGATAAGATAGCAAAAGTTTTTTTTTTTTTTTTTTTTTTTTTTTTTTTTTTTATTTTTATCTACTGG
ACATTTGTTTTGATAAGTAACCTATTGGATATTTTGAAGCCAATAACTTCTGATTATACTGGATGTAAGT
TGTAATGAAATTGGTTACTTTGATACTATTGCCTACAATTTCTATTTTGATTTGTTGGTCTCCCTACAAT
TTTTATCTTTCCAATGATGATCCATTAAGTTATACAAAAACTCTATGTGCAATCACTTTTGCGTACTCCT
TGCGCACTTCACTAATGTGATTGGCTGCATCACTTTTTTTTTTAATATAAAATAACTGTTTTGGCCAATC
ACATTAGTGAAGTGCACAAAGAGTATGCAAAAGTAACTGTATGTAGCAAAACTCTAAGTTATATTAATAA
TGAAATACAGATGTTCAAAGTTCAAACTGATTTTCTCCTCCCTAATACATTTTATGTTTAGGATCTAACT
ATTGGTTTTTCTTCTCATTGTCAACCATCAATTAAATTTCACTTTTGATACTGCACTATCTTTTGGAGGG
AGGGGGGATAAATCTTGTGTCTTGCTCATAAATTACCATCACAATGCAATTCTCCATTTGGGTTCATTTT
TCTGCTATATGAAATTGTTTTTTTTTATTAGTTAAAATATTATATATATAAATATATATATATATATATA
TCAAAAGGAGTAACCAAGTATACTGATGTATACAAGAGAACACTTTGCCCATAATAGAAAACCCCTAATA
ACCCAAAAAGCCCATGAAAAACAACCTAAAATACACCAAGCCCGACCCCAACCCCTTGTTTCCCCGTCTT
CACAATGCCCTTCCTTTAGACTTCCTTTTAGTTGAGCTACCACCCTTAGCGTTATAGTTGATTGTCGAAA
AAAGACGACATAACTCCCTGCTTTTCTTGAAACTTGATTTGGTTTCAAGCGAGTGGCTTGCCTTGATCGT
AGTAATTAATTCTTTAAACTGGTCTTCACATTTTCCATGTGAAATTCTCACGAATTGCTGAATCTTGTTA
ACTTTAGGCAGAATCCAATCTGCTATTGGAGGAAGAGAGACCAAGGGAGCCACAATATCCCCAGACACAG
TATCCAGCTGCACTCCTGACTCTAGGCAAGGCACCAACGCCAAATCCATGTTCAAATCCTGTACCTCCTC
AGCAACTCCATTTGTTATCTCCAATGATATGACATCAAGTCCTCACACCTCTGGTGACCCTTTATGTGCA
ACTTCAGCGGGTCCCTTCACCAATGGTATGACACCATTTTCCTTCAACTCCGACGAGGGAGCCATAGTTT
CTTCTGGTACAGAAGTCACCTGAGGCGAATGACATTCTATTACAGATGTTTCTACGCCTATACCCGACGT
AGAACCCACTTCAAATAACCCAAACTTCCTTTTGACCCGGCATACTCTTTTGGATCTGGTTATAGGAACA
GGGCCGAATCCGATTTTCCGTTTTCCTTTATCTGAGTTTAAAGGATTCGAGCCTATCTTAGTCTTGTTCT
TTACAACTCTGTTGCCTCCAGTTGAAAGCAGGTCTGTTTTAGTAGACAAAAAAGCTATCGCTGCCTTCAA
TTCGACAAGTTGGGTTTCCATCTCCTTTAAAGAATTATGCATCGCGACAAGCTGGTTCTGAGGCGTGATC
TGCAAACCACTTACATCATTCCCCTCACTATGAAACCCCGAATCATGATCGGTCTACAGAGCTACCACAT
ATGATCGTCTCGCCACCGTGGATGTACTATGGTCTTTATGATTCACCTCCAAGTTGATATTCTCATTCTT
CTTTTGGTTTTCCTTCTTTGACCCATCAGCAAAAAGCATATCCGCAGCTCTGCGTAATTCCGTAGCAAAT
TTCTCCCAACCCAGACCTTCATACCCTTCAGGAATGGCTACAACACTTCGCCGGCCGCCTCCACCATATA
CGGTGACAGTTAAAAACCTACCATGTTTATTGGAGCATCTTTGAGCCATGAATGTTCTGCTGCCTTCGCG
CAAGTGTTTTGTGAAGTCCAAATTTTTTCCTGCTTGTAATACTGCTTCCATCGTAGTCGTCAACCATTCT
AGACCACTGCGACCCATCACTACAAATCTCCACACCCTTCTACTCCTCTCCACAATTTTTAATGACGATC
TCCATGATTCTGGCAAAAGCTCAAAAGCCTTCGATTCCACCCAAAAGCGCACCAGCTGACCCATACTGAT
CAACCCTTCGACTTTAAATGCAGGGGCATCCCATGTTGAAATGCATCCTTCTGTCGAGTAAAAGCAATGC
GCGAAGGTGGCTGGAGTTATCAGGTTGGGAAAAACAGAGCCCAAAGGCGCACCAGGTTGGTAAAAGCAGA
ACCCCTTAATTGAGATAACGGAAACTTCCAGTCGCCGGAGGATGACCGGCTTCGGAGACCCCGCAATGGG
GTCGCCGGAGTCCGTCGAACACGTTTGTAATGATGGCTAATGCAAAGGGTCAGGTCTTGTGAAGTAAAGG
AGGAAGTAACGGAAAAACAGCAAAATACTCCCAGTTGTCGGCAAAGGACCGACGTCGGATGCCTCACAAC
GGACTCACCGGAGTCCGTCGTCCAAGTCTGTGGCGGAGTTGAAAGTGCACGGCGACAGTACGACACCTTC
GACAAAGATGAAGTGAAAAAACCAAGTTCGCCGGCAAGGACCGACGTCGGATGGTCCTCAGTGAGGACGT
CGGGGTCCGCCGGCCTCGTCTGTGATGGAGGTGAGATATCACACTCCGATTGATGATGGGTGAGGGGTGG
CGGTGGGTTGGTTGAGTTAGGGTTTCTCGCTTCACTGTTCACCCACGGGAATCGCAAGGTGTACATGTAA
ATTTCCAAATGCAAACATGTTGAAGTAAAACTGAACTAGATGAAAATTTGAAGGTTACACAGTAGATGAG
ATACTTCCTGATATATTTTTATGAAAACTGAACGGATACATCCTGTTGAGATACTTCCTGAATTTCGAAT
CTGCTTTATGATATTGGCTTTTTATGTATGGGTCTTATTTACGTGGGTGCCATCCCTCTTCCCACCCTTT
ATTGAAATTTTTCTTATGTGTCAAAAAGAGAGAAAAAATAAAAATAAAAGATAAGGGGAAAAAAAAGTGC
TATCATCCTTTGAAGCTACGACTTGAGAAAGTACCAAAGACCTTTTCAGAGTATCCGTTTCTTATCAAGC
AATGATATTATGGTATAATCAGCAGCTTATTTGGTTGAAATTCTTTAAATGAAAGATACAAACATTTACT
GCTTGCATTTAGCCATTCCTTAAATGACTGATATTGCTTTTATTAGGTATCTGCATTTAGGTTGTGGAGA

CCTTTCTATCTTCATAAGCATCACTTCGTTGAGAGTTTTAACCTTGTTCCTCACATTCAATGGTTATGAT
TTCTAGCAATCAGTGACATCACATGGGCTATTATCCTTTTTTTTTGCTCTCCCTCTTCCAATTATAATTA
TCAGGTTTATTCTTAATGCATGTGCTATTCGATAGGCTTGTTATAGTATAAGGTGTATGCATGCACATTT
TGGCCTGTTAATTAGTAGTAGTTCGTTTTAAATGCATATAAATGAACGTGTTTGTGCATGTATATTGGAC
TTTCATTGCTTTTGTCCTTTTCTTTTTATCCACTCCCTATGCTGCTAATTCATTTATATCTCAGCTTCTA
CTTGCTGTGGGGACTAAGCTGGAGCATGTAATAACCCAGTTGGCTCAGGAGGTTGCCGAGAAACATGTGG
CCATAGAAGGTGAATTGGTAGTTCAGCCATCAGATGAGCACTTTTGGTTTGGTCGGCCCCGCATTGTCCT
CTTTCTGATCCATTTTATCCTTTTCCAAAATTCTTTTGAGATTGCATTTTTTTTCTGGATATGGGTAAGG
AGTTCTTCAAGCACCATGTTCCATATCTTTCAATATTAAAAAATATGTCAGTAATTGAGATGGAACGTAT
TTAACCTGTTTTTCAACACATGTAGGTGCAATATGGCTTTGACTCTTGCATAATGGGACAAGTTCGTTAT
ATTGTCCCAAGGCTCATCATTGGGTATAAATGAAAGACCCTCTTATACTTTTTCATCAGTGTGAGATTAC
TGTGTAGGATCTTCTGTGCCCGAGTGCTAAAATTTGTCTTTTTAAACCTTTTGCTGTTTTAATCTAGGAT
CTTCATTCAAGTGTTCTGTAGTTACAGCACCCTTCCACTTTATGCCATTGTCACACAGGTGCTTTGAGTT
TCTGTTAAAACTAGAATTCATTTTCTTTTTTTCCCTCGAACAGTGTAACTAACCTTATTTCTGCATTTAA
ATGAAGATGGGGAGTAATTTCAAGAAGTCTATCTTTGATGAGCATGTGCAAGCCGGCCTTGTTGGTTGGG
CTCAGAAGGTGAAGAAGAAAAAGGGATTAAAAGCAGTTAACAATGGCTCTGGCCAAGGAAGTTCACATGA
AAGTTCTACTGTGGGAATTCAGATGGGGGCTCTCTTTCGTAAGGCGTCTACACCAGAGGAGATCCAGCCT
GCGGCTGGTCCTGAAGGGTCAAAATGAGCAGTACCATTCATCTAGGACCCTGGTACGTCACTGGAAAAGG
TCAGGCGAAACATAATCATTAGGATTATCTTTCTGGCTTAAAACGAAGGATTAAACAATGGAGACATTTT
TGTACGTCAGCCTATCTGTGAATGTATTTATATTTTTCTTTTCAGCAAAAAGGTTTGAAGTGTTGCTCTC
AAAACTGAAGATTACAGCATATGTTGTCTTTTTTGTAATTTTCGTTTGTAATTTTTATGCCACCAGCCTA
TTATGCAGCATATGTTTTTTTCT

>NW_017438726.1:c158643-153134 *Juglans regia* cultivar Chandler unplaced genomic scaffold, wgs.5d, whole genome shotgun sequence
ATGGCTGCCGGTAGTACTGCTGCAAGAGATAGGACTCTGGAGGAGACACCCACATGGGCGCTAGCGGTTG
TGTGCGCTGTTTTTGTGATTATATCTGTCCTGATAGAACATGGGATTCATTCCGTTGGAAAGGTATATTA
ATGTTTTTATTAGAGCCAATTATCAATCATGCATGGGTTTTTTAATTTCTGGGTACGTACGTAGCATTTA
TATGCATGCATGCATGGTTTACTGCTACATCACCCCCATTTACCTTGCTTGTTTCTGTCCTGATCATGCA
GTGGTTTCAGAAACGCCGGAAGAAGGCCATGAGTGAAGCATTGGAGAAAATCAAAGCTGGTTAATTATCT
ATTAATTAGAGTATCGTATTTATATATAATTATTGCGCCATTAATAGATTTGAGTGTTGGTCCAACGAGT
TATATATATATATATATATATATATATATATATATATGCTATATATTACGACACTCGTCTCAATTTCATCTT
ATTATATATGCTGATCACGCGACAGTATCCATCAACCGTGCATTAGATTAAAATGATATATACATTCAAA
AGTGAAACATGATATATATTTACCAATCACATTTCCCATTAAAAGTAATTGTCTGTTTACTGTGTTGCAG
AGTTGATGCTGTTAGGTTTCATTTCCCTACTGCTCACCGTGAGTACGAGATTTATAGCAAAGATATGCAT
CCCAGCTGAACTTGAAGACACAATGCTTCCATGCAAACCTTATGATCAATATAAGAAGGACGACACGGAT
GATGATGGTGAAGGAGATGGCAATTATGACAGAAGAAAGTTACTTTCATACGCTGAAAATGTAGTATGGC
GTCGAGTCTTGGCTGGCGGCCCTGCTGGAGACGACTACTGCTCAAAATATGTAAGAAGCTGCATGTTATA
TACATATATATATATATATATATATATATATATATAGTACTTTAATTCTGGAACATGACTTTAAGACTTTTAAC
ATGCAGGTTTTTCCATCAAACTAATTTCGGATTATATTCATCCAACTTATAATTAAGTTTGTAACTTTGA
CATGAAGTGTCCGTTGAATATAGGAAAGTAAGTCTTATTTAAACGACTGTTAATGCTACGTACAATTGTA
GAGTGAGCAAGCGGGACGTAATTATTTTGAAAAAAAGTGAAGTTCATTATTAAAAGATTTGTTTTTTTTA
TTTATTTATTTGGATTCTATATTTACTCACTTTTTCAAAAGAATTGCAAGACATTTATACACTTTACGAT
TACAAATATCATTTATCTATTTAAAAAGTCTAAATTTAAAATATCATCGAGTATCTCTTGATCAACAAGT
ACATATTTTTTTAAGAGAATTAAGTAGTGCTTTCACAACCACAAATAAAAAATCTCATAAAAAATAAACTTA
CAAACTAACATGTATTCATATGATACACGTTAGATATACTTTACAATAAAAAATAATTTTATAATATAACA
TACTACATCAAATTATGTCAGTTTGCGAGAATTTTTTTATACGTGGCTAAACCATTTTTTTTTTTTAATTC
GAATTTTAAAGGATGTACATGACAAATTTAATCCATTGGGTTCCATTTTCCAATTAAGTTTGAAGAAAA
ATCATATTTATTTGTATACTGATCATGTGAAGGCAAATAACCCATTAAGGTTTTGTGGTAATTAATTAGG
ATAAAGTCCCATTGATCTCACCGTCTGGGGTGCACCAATTGCACATATTCATATTCGTCCTGGCTGTTTT
TCATGTCCTCTACAGCGTCATTACCATGGCACTGGCGCAAGCTAAAGTATGTCGATCTCCTTATTAATTA
TATAATTAATTCTTAATAAGGCTATTTATTTACTATATTTTTTGGTTGGAAAATATTTTCTCCATGCTAG
TATATATAAATTAAATTACTGCTTACAAAGGAGAAAAAAGGGTAATATTTATTTATTTATTTTTTTATGG
ATTTTTTTAGAAATATTAAGTTAATATAAACATCAAAGAAATATTTATTTATTTCTTATTTTTATGGGTT

TTTATTTTATTTCTTACTTTGCTTGTGGATACTGATCAGACGAACAAATGGAAAGCTTGGGAATCGGAAA
CATCATCCTTGGAATACCAATTCACCAATGGTGATTTTTTTTTTTAAATCTCTTGGATTACTATACATTT
TATTTTGAGAAATGAAAATTCATCATATTTTTTAAAGAGTAATGCTACATTTTATTTTGAGAAATAAAAT
TTATCATAATTTTTAAAGAGTAATGCTAGATACCGTCATGAGTTGTGAGTAAATATGAGACTCATATAAA
AAAAAAAAAAAATTTTAATGTTAGACCTCGTTTTTTTTCAAAAAGAACATGTGGCGCTTATACAACTTATA
ATTATATTTAACATTACTCTTTAACAGTCGTGAAACATCAAAATAATAATTGTTAAATTATTTTTCTCTT
ATGAATTATTTCATAAGAAAAAAAAAAAAGTGATTAAAAAGGCCTATTAATAATTTTTCTTCTATTTTCTG
CAAATTCATTGGCTTGGGCCTATATTAAATATTTTTAGGAATTGAACCCAAAACTAATAGGCAATGGTTT
TGATTCTTCAGACCCAGCAAGATTTAGGTTCACACATCAAACATCTTTTGTGAAGCGCCACTCTGCAGGC
TTCTCAACAACAACTGGAATAAGATGGATCGTAAGCTTAAGATCATATATTCCTCCTCCTTCTTCTTCTT
CTTCTTTTTTCGATTTTAATTTCTCTGAATTTGTTGTTTTGGAATGATGTTTTTGATGCATAGGTGGCAT
TTTTGAGGCAATTCTTTGGCTCGGTGTCAAAGGTGGACTATGTGACTATACGTCATGGATTTATAAATGT
GAGTCAACGACCAGGTTTTTCATGCTCTTTTTTTCTGTCTTCGTTTATGTATACCATGCAAATCAGACCA
AACTCAAAACACATATTTGAATTGAAAAATTCTTTTCATCAATTACTATTCATCATTTCACAACTCATGT
TTTATGAAAAATACATCCACACACTATAAAAAAGTTACAGATATAGAATATGAAAATGAATAATAACTGA
TGTATAGCATTCATCGTTTGAATTGCAGGCACATTTTGCCCCAAACAGCAAGTTTAATTTCCATAAATAT
ATTAAAAGATCCATGGAAGATGACTTCAAAATGGTGGTGGGTATCAGGTCAGTACTCTCCATCCATCTTT
CAGTCCAAATTACAGTAGTACTGTACCAATGTTAATGTCTAATTAATGACCTAAAGTTTCTGTTTGTTCC
TATTTTTGCAGCCTTCCACTGTGGCTTTCTGCTGTCATTTTCTTGCTTCTCAATGTTTACAGTAAATCTC
TGATCTAATTTCCTATGAATAATGTTAGATAATACTACTACATTTGAGGCATATTTGGGATCCAAAAAAC
TACTTTTTCATGGTGGGCCTCGCTTTTTTTTTTTTTTTCAAATAGATTGTGCAAGATTTACACACTTTAT
AAATGTATGCAACATTACTCAATATTAAATAATTAAAAAAATTTAATGACGATGATTTTAACACTTTTCT
CTTAAATGGTGCAGAATGGTACTCGCTCACCTGGTTGTCATTGGCACCACTAGTTGTAAGAAATTGATCA
GAAATTACCTTAATTTCTATATACTGGTTGTCTCAAAATATACATAATCTAATTATCTTATACTATAGTT
TAAAATTATTTAAATATTAGTGCCAGCTAGCTGAGCTGCATGCTGATCTGTATGATATTATTAATTTATT
GCGCAGATACTTCTACTAGTTGGCACAAAGCTTGAGCTTATTATTATTGAGATGGCTCAAGAAATACAAG
ATCGAGCAACAGTTGTTGAGGGAGCTCCAGTGGTAGAGCCAAACAATAAGTATTTCTGGTTTAACAGTCC
TCAATGGATTCTCTTTCTCATACATTTTACCATTTTCGAGGTAAAATCATGTCGCGCGACTATCTTTTAT
ATATATATATATATATATATATATTGTACTGATTTTTTCATTTATTTACTTTAAATACAACATAGAAATAAT
GAAATTTACGATTAAGAACATCATGATTTGATCTCCAATATTAATATTACATGGTTGTAACATGACTTGA
TTCATTTGTACAGTCAGCTTTCCAATTGGCATATTTTCTTGTGGATTTGTGTAAGTCATGATTTTGCACT
CAAGCTATTATTTCAACTCATCACCTTATTTTATCCATTGATTTTTTGGTAAATGAAAGTGTAAAAGTGG
AACTAATTTCTTGCATATATCATTGTGGAAATATTAGATGGCCAGTATGAGTTTGGGATGACATCTTGTT
TCCGTGGAAACTTGCCGGCAGCATTGACTAGGGTTTCACTCGGCATAGCCCTTCTCATCCTTTGCAGCTA
TATCACCTTCCCTCTCTATGCCTTAGTAATACATGTAAGTGTACTAGCTAACAACCATTGAGATTAATTT
CTTTGTTCTTTATGTAAAAGGGTGGCTTCCATTAATATTTGCGGATGGATTGAATTGGATTGTTCGATTT
TAAAAAGAGAGAAATGCATTATGTGAGACGTTCTACAACATTAAATTATCTGAACCGACATTTCTCGGTA
TCAGACCATCCAGTCTGATTGAAAAATTAGAGAATGTCTCGATTGCCCATTTTGAATATGAATTCAGTGA
ACTAATTGTATTATTGTGATACCCAATATGATATGGATAAGAGTAGGTGGTGTATGAGATTCTATATTGC
TTAGGAATGAGAAGTTTTTACTCTTTATAAGGTTCCAATAAGGCTCCAATTGTATCATTGACTAGTTCTT
TTGGAGTATAGGCCATGTGATTTGCCTAATAGGCTCAAGTCCCACATTTCTGATTGGGATAGGCTCTGAT
ACCATTTGTAATGCCCCAATGGAAGGCTCAAACCACATGACCTATACTCCAAAAGGACTAGTCAATGATA
TAATTGGAGCTCCATTGAAACCTTATATAGAGCAATAGCTTTTCCTTCCCAAGTAATGTGGGATCCTATA
CACCACATACTCTTATCCATATCATATGAGATATCACAATTATCAAACATGAACTTTTGACTCTCAATTC
ATCATGTCTACAGATGGGATCTCATATGAAGAAAGCTATATTTGAGGAGCAAACAGCAAATGCTCTTAAG
AAGTGGCACAAGGATGCAAAAGATAGAAACAAGTTGAGGAAAGCTACTGGAGTAGATACTAGTTGTTCAA
GATTAATGAGTGGGGAAAACACACCTAGCCAAGGAACATCACCCAGACACTTGCTCCACAACTACAAGTA
CAGGTCCAGTAAATTACAGCTCGAAGCTGGTGTTCTCAGTTCCCCAAGATCCTACCAATCTGATACTGAC
CTTTCAGAAATTGATCAGATCGGTTCCCCTGATCATCAATCAATAGGTCATGATCATCATGATCAACCAC
CAAGAGATGAAAACTCACATAGTATTGATTTTTCTTTTTCTAAGGCTTAA

>NW_017429870.1:495-1468 *Juglans regia* cultivar Chandler unplaced genomic scaffold, wgs.5d, whole genome shotgun sequence
ACGTTGGGTTCAAATTAACAAACCTGTCTCTCTCATTTTTTACAGTATTTCTTCTCTTCGCAACCAGCTG

CGATACTGACGTATTTCTGGGGTTATCCTCCTCCCATTTTTGTCATCGATTCTATTCTCTTCGTCCCTTT
TTTTTTTTTTTGTGTGGATATGGAGTTCTTCAAGACAATAACTTGAAACAGAAGACACCATATATATTCA
TTCTGGTTTTGGTTAAGGGGAGAGATCGGTACAGAGAAAGATGGCAGGGGGAGGAGAGGGAACAACTTTG
GAGTATACCCCAACGTGGGTTGTGGCTGTTGTCTGCAGTGTCATCGTCCTCATCTCTCTCGTTGTCGAAA
GAACCCTCCACGGCGTTGGCAAGGCATGCACATATATATTTTTTCATTTATCTTCTAGTTCTCACCTTGT
ATGTACGTGTACTAGACCGATCTTTATTCTTCAAATTGATGTTTTTATTTTTATTTTTGCAGTATCTCAA
GAAAAATAACCAGAAGCCTCTCTTTGAAGCATTACAGAAGATCAAAGAAGGTCACGCGCGGCCATATCTT
CATGTTCCTCGATCTCTTTCTCTCTACACAAATATGCACTTTTTTTTTTTCACCATATATCTGATGGTAT
TTTGAAGGTGAATTAATGCAGAGTTGATGCTGTTGGGTTTTATATCACTGCTGCTGACAGTGTTCCAAGA
TCGGATTGCCAAGATTTGTATTTCGGAGGTTTTGGCAAATAAGTGGTTACCCTGTAAGAAAGAAAAAGAT
AATGATGATGATGATTCTTCTTCTTCTTCTTCCACTGCCCATTTTCAAACCTTCTTCAAATATCCCA
GCTTTGTTGATCTTCCTGCGGGGACTGCCCGCCGTCTCCTTGCAGAGGCCTCTGCTTCCACTGAGAGCTG
TGCAAAGGTACTGTACTTAGTCTGTTACCTTCACTCATTCTTCCCACTCTCTTCTTCTTTAAAA

>NW_017400138.1:489-1266 *Juglans regia* cultivar Chandler unplaced genomic scaffold, wgs.5d, whole genome shotgun sequence
AATGCGTTCCAAATGGCTTATTTTCTGTGGATTTGGGTAAGTGCAACTATTAATTTCCAACTCCGGACGT
GCGTTAATTGCATATATATTATATACACATGATATATAATATCTTTAAAATCAACATATAAATCCGGCCT
CTTATTAATGCCATTGCAGTACGAGTTCAAGTTGTCGTCCTGCTTCCATGAAAATTTGGCCGAAGTCTTG
ATAAGGGTTTTCCTTGGTGTAGCCCTTCTGGTATTATGTAGCTATATCACCTTCCCTCTCTATGCCTTGG
TGACTCAAGTGAGTAGTTCTAACTTCTAATTAGCTGAATATGTAATTAATTACTAACAGAAATCAAGGTG
AGACGACTATATATATAAGAGTACTGAAATATTTGCAGATGGGATCTCACATGAAAAAGGCAATATTTGA
AGAGCAAACAGCGAAAGCTCTAAAGAAATGGCAGAATGCTGCAAGGGAGAGGAAGAAGTTGAGGAAGAGT
GGGGCAGATGTTTCCTCTACGTTCATGAGTGGAGAAAATACTCCAAGTCATGGCTCATCACCCATACACT
TACTCCACAAGTATAAAACCAATTCTGCGGATATTGAAAGTAGTCCTGATCATCTCGGCTCTCCTAGGGC
TTACCATTCTGAAACCGAACTCTCAGAGATTGAAGGTCCTACACGTCTTTCTAGTGACGATATTCATGAA
CCAAGAAGACATAACAATCCCGGAATAAATCATGTTGAAGCTCATAATTATGGTGATTTCTCGTTTGTTA
AGCCCTAG

>NW_019809665.1:c89739-79715 *Quercus suber* isolate HL8 unplaced genomic scaffold, CorkOak1.0 scaffold_4162, whole genome shotgun sequence
ATGGCTGCCGGTGCTACAGGAGAAAGGTCTTTCCAAGAGACACCTACATGGGCTGTTGCGGTTGTAATGT
ATGTGCTGTGTTTGTGATTATATCTTTATTAATCGAACAAGGAATTCATTCTCTTGTAAAGGTAGTGTTT
TGATTAAACTGTTTGTTGCTTGGTATGTAAGTACCAATAAGTTTAGAGATGCAACATTTTTCTTGACTAT
TGATGTAGTTTCGTGAAAGTGATGTCCGTTTAGTGATACATTCCCTTACTTAGCTTTTCTGTCAAGACAG
TGGTTTCGGAAACGCCGGAACAAGGCCTTGATTGAAGCTTTGGAGAAAATTAAAGCTGGTTAGAATTTTA
TGTTAAACTTGGAGTCATCTTTAAATAATAATCAATAGTGGTTTAGATTAGCCAAAACTTTATAGCTCAA
TTGGCAATTGAAAAATAAACTGTATTAAACGAACACTTATACTAGGACTAGAAGTTTTTTGAAATCGATA
CATATTTGTCAATCAACATCTATGCACCACCTCCATAGTCTAATCCATAGTAATTATTTTGTTTATTGAT
TACTCTTATTATATGGTTTGCAGAGTTAATGCGATTAGGTTTCATATCCCTACTGCTTACAGTGGGTACA
ACCTATATCACAAGGATATGCATACCTTCTAAAGTCTAAACTTGGCAACACTATGCTTCCATGTAGAATT
GATGCTACTAAGAAGGATCAGGGAGGTGCTGGCAGGAGAAAATTACTTTCAAATGATGAAAATGTAATGT
GGCGCAGAGTCTTGGCAGGATCTGGTGGAGATGATTTTTGCACAAAAAATGTAAGATTCTGAAGCAAGAA
CTTAACCTTAACTACCTATGGTTTATGTTATGATATATGAGTGAAGACAGCTTTGAAGAAGAATAAATTA
TGATTAGCCCAATGTCTTTGTGCGGAATAGGGTAAAGTGCCACTGATTTCACAAATAGCAGTGCACCAAT
TGCATATATTCATCTTCGTCCTTGCAGTTTTTCATATCCTCTACAGTGCCATTACCATGGCATTGGGGCA
AGCTAAAGTAAGTTTCTTTTTCTTTTTATTGAGATTTTTTTTTTTTTTTTGGTTTAGTTAGAGAGAGACT
CTAGTTTATGATTTTTTCGGCACTATACAAGTAGGATCAATTAGTGATTTATAGGCATATAAGTAGACAC
AAACCCTTATAGCTCAGCTATTAAGAATGTTATAATTCAATTTGTTGACATTTCGTGTGTTCATCTTATA
ATTCAAATATCACATCTCTCTCTCTCTCTCTCTCTCTCTCTCTCTATATATATATATATATATATATATA
TCCTCCTTGTAACTATGAAAAAAAAAAAAACATATACAAAATAGCTTTTACTAGAGTAGTGGGTCATGGC
CTCCTTGGCCACTATTTTTTTAGTATGTATTGTAATTTTTTTAAAATATAATATATAACTATATATAGTG
TGCGTTTGCGCAGGGATGAACGCGCATTTTGCCTTTTTTTGGCTGGTCTCATGACACTGTTCATGGACCA
GCCAACCCCTAAAAAACGCAACAACAATGTTTTATGAGTCCCACAGTACTATTCATACTTTTAAAAATTA

TTTTACTACAATACTTTTAGTAGTAAGTTTTCAGTTTTCAACAAATAAACAGTATCTAAACACAATCATA
GTTAAAATTAAGTTATAATAATTCCAGATACATCAAGTCAATTAAATTACTCCAAAGATACCCCAAAATG
TTAACTAATCTCCACCTATGGCATCCCTCTTAAAAATAACTCTTTAGTTAATAAATAACTAAAATCAAGT
GGTGCTAATGTATTGAAGACATCAATCACTTTAAGAAGTATTGTCAACTTATCCATGCATCACTACTGCC
CTAAAACTTCACTTTATTTCTTACAATTTTTTTTTTCATACCACCTCATTTAAAAAGATTGAAATATAAG
TTTTACCAACCAAGTCAACTTTGTTGTGTTGATCTATATTGTTGGCAAAATATTGATAAGTATTTTAATA
TATAATTGTCAAGAAATGACACTCCACTTCTTGGGATTTATATAAAGGTAACAAATAAGTCCATACACTC
ACAACCTATTGCTTTTCAAATAGAATATTCTATTTGCAAAAAAGATTCCTACTTAAGCTATATGCAAGAT
GACACCCTTCTCTTGAAGTTTGTAGATAGATGACACTCTCTCCCTTATTATTATTATTATTATTATTATT
ATTATTATTATTATTATTATATTTTTTTTTTTTTTTATGAATGACATTCTACCCTTGAAGTTTGTGTAAA
TAATAAAAAAAAAATGAAATTTTATGTCAGGAAAGTAATACTATGACTATGTGAGAGGGTATGGACTTATT
AGTTACATTTATGCAAACTTCATAACTGAGACTATCATTTTATTAAATGTTTGGGGGGGAGGTGTCATAT
TATGAAACCTAATAGGAGGCAGGTGTAATTTAACCCAAAAAGAAAAGGGAAAAAAATGCACAAGGATAA
GATGAAAATTATAAGTGAGTATTTCAAAAAATTTATAAGTGAAAGCTTTATATTTTTTTTTCTTTAATTT
TTTTAATTAAAAAATTATATAATTTTTTGACAAAGGAATCTAAGTTAAGTCCAAATGTATTAAAGTTAAT
TACCTTTTTTTTTTAGAATCATTAAAGTTAATTACTTAATAATCAAGTTCCAGGCCATTTAAACCTTATT
AAACTATGTACTATTATTCTCAAAAAAAAAAAAAAACAAAACAAAAAACAAAAAAAAAAACTATGTACTA
TTTGCCTCTTCCTTCTTTTTTTTTTTCTTTTTTTTTTTTTTTCATAGAAAGGTAGTACTAATATTTACGA
TTTACTTGTGAATGTTGATTAGATGAAGAAATGGAAAACTTGGGAATCGGAAACTGAATNNNNNNNNNNN
NNNNNNNNNNNNNNNNNNNNNNNNNNNNNNNNNNNNNNNNNNNNNNNNNNNNNNNNNNNNNNNNNNNNNN
NNNNNNNNNNNNNNNNNNNNNNNNNNNNNNNNNNNNNNNNNNNNNNNNNNNNNNNNNNNNNNNNNNNNNN
NNNNNNNNNNNNNNNNNNNNNNNNNNNNNNNNNNNNNNNNNNNNNNNNNNNNNNNNNNNNNNNNNNNNNN
NNNNNNNNNNNNNNNNNNNNNNNNNNNNNNNNNNNNNNNNNNNNNNNNNNNNNNNNNNNNNNNNNNNNNN
NNNNNNNNNNNNNNNNNNNNNNNNNNNNNNNNNNNNNNNNNNNNNNNNNNNNNNNNNNNNNNNNNNNNN
NNNNNNNNNNNNNNNNNNNNNNNNNNNNNNNNNNNNNNNNNNNNNNNNNNNNNNNNNNNNNNNNNNNNNN
NNNNNNNNNNNNNNNNNNNNNNNNNNNNNNNNNNNNNNNNNNNNNNNNNNNNNNNNNNNNNNNNNNNNNN
NAAAAAAAAAAAAAAAAAAAAAAAAAAAAAAAAAAAAAAAAAAAAAAGAGAAGAAGAAGAAGTAGATACA
CATAAATAATACAGTAATAATTATAATATTGTCTTGTAATAAGTTGAAAAAAGTGAAATTTGTAAATAGT
ATATAGGAATACTCATATTTTGTTTGGGTGTAAAATTGTTATAGTTGGCCCCTCAAAATTTTGAATTGGT
CCAAATAACCCAATAGAAATTAATCACTAACCCTTTCCTTGTGCCTGTGGCCCCTCCACAGTCCAAATAC
AACACAACAAAAATCTCCTAATCACCATTGTCACAACACAATTGAAGAACTATCTTAGCGTATGTACTAT
GTCACAGGATAGCTGCCCTCCTTCTGTTTCTGTTATTAGGGAGGAAGTTAGTCTTTGTTTGTAGTTTGTT
TCTCTATCCAATAAAGTCTTTTTTCATTTAAAAAAAGAAAAAAAGAAAAAGAAAAAAGAGCTTAGCATAT
TTAACCAATTTGTTTGAGCTTTGGGTGTCAACAGACTTAGTAGTAATTAATTTTTTTAAGTAATGCGATG
TGCATAACATTTTTATAATAAATTCAAACTGACAAACTGTTATTGGTGAATAAAAAAGTAATATAAATGG
TTGGCTCAGATTATAACCAAAAACAAGTTTTCTTTTTTTTTTTTTAATTTTTATATAAATGGAAAGAACC
ATTAACAAGTTACTTTGTAGGATAAGAACTAGTGAAATTGTTGTGAATCATTATTTAATTTTTTTTAGTT
TGAGAATAAATAATGCACATATGATGAGACAATTTATATGCCTTTTTTAATTGATTTATAGATTATGGGA
AAGTCCATTACAATATTTGTCTTTTTTCACAAGATAAAATGTAAATGCTTTCAAAGCCAATATTGATGAT
GTAGCATTCTAATCCCATAATACTAATTTCCCATTTTTTGAAAATCCTCAAACAAAGTTTTGAAAAACTT
CGTTAGTGTGATCCAAGTTGCATAAAAAAGAATAAGATTTTCATGTTGGTCATATGATAAAATCAACATA
TTCTTTCCAATTACCCAAAATTTGGAGATCCAAGTCCTTTTGTTTTTTTTTTTCTTCTTCTTTTAAAAAA
TGTTAGAATACCATGTTGAAATTTTCAATATTTTGGTTCATGTTATTTCCTTCGTGACTTAAATATTTAC
GCAATAAAGTGCAATTTTTTGTCTACCATGATTTTTCTTTAATAAACCATTTCATCTGAAATTTTTTTGA
CTCATGCTCCATTTCCTCCCCCCCAGATCTCCCCCCAAAAGTTCAAATCTTAGTTCCATCATTGGTTTGA
ATTGTATATTAAAATACACGTAGATGGTTATTAAATCATCACATCATATGTGTTAGTGGAGATTCCTTCA
AACCAATTTAAAAGAAAACTTTGTCCAATTGTTTCTTGCTTGTTTGTTTTTTTATTTCTTTTCTTTCTAT
GTCTGCATAATTTATTGTTTTTCAATGTTTACTACAGGTAGAATTCTACAGGTAATTTTTTGGCTCGCTG
TCAAAGGTGGACTATTCGACAATGCGGCGGGGTTTATCAGTGTAAGTGAAATGGACTCTTTAATATGCC
TAACATAATATCATTTGATATTAAATATTTTTTTCCTTGATCAATTTTCCTGCCCATTTATTTATGTGGC
AAATTAAACCATTTTTTAACAAATTGTACAGGCACATTTTGCCCCAGACAGCAAATTTAATTTCCACAAG
TATATTAAAAGATCAGTGGAAGATGATTTTAAAGTTGTCGTGGGTATCAGGTCCCCTTTCCTCAATCCTT
ACAATGCAATGTACATTTGTTTTTGTTTCCTTTACACTGATCTGATGGTAATGATTTTTTGCTTTCTGTT
TGTAGTATTCCATTATGGATGTTTGCAGTTATTTTTTTTGCTTCTCAATGTTTACAGTAAAATCTCTCTC

TCTCTCTCAAAAGGGCGGAGGCACGTATATTAGGGGGCCATGGCCCTCCAAAACTATTGATGCCCTTTAT
AATAGTATATAAAAATAAGATGCACTTCTACAAAAATAAGCTGGGCTTTAATAATTGTACCAATTGGCGC
CACCTCGAATTCAACAAAACCCAAATACTTTAGTCCACAGGGTGCTCAAAGCAAAGTACTTTGCTAATTC
GACGTTTAAGGAAGCTCAATTAGGTAGTAGACCTTCATATGTGTGGCGAAGTTTGATGGCTGCTAAGGAT
ATTGTGGTGAATGGTGCCAGATGGGTCGTGGGTAATGGAGAAAGTGTGAGGATATGGGAGGATAGATGGA
TCCCTATTCCGGATTCTTTCTTGGTGGTAAGCCCAAAAGCCCCTCTTGAATCTGATATGGTTGCATGTCT
CATTGACAAAGAGACAAGATCTTGGGACATAGACAAGGTGAGGAGAGTTTTCTTACCTCATGAGGCAGAT
GCGATTCTGGGTATGGTAGTAAGTTCTCGGCCTGTAGAGGATTCAGAGATATGGGCATGGACCACAAACG
GCAAATTTTCAGTAAAAAGTGCCTATAGGGTAGCTCATAAGTGGCTTACAACCCAGAACCACAAAGCAGA
TAGGGGAGGTTCCTCAGATAACACACGTATGCGTGCATTATGGAGGCTGGTGTGGAGTCTAAACTGCCCA
AACAAACTAAAACAGTTCATGTGGCGCTCGTGTAGGAATATCCTTCCAACAAAACACAGGCTAAAATCAA
GGGGTGTCGATATAGAGGTGGGGTGTGACTATTGTGGGCAATGTGAATCAGTTGTACATGTGCTGTGGGG
ATGCAAATTTGCTACTGATGTTTGGGGAGAATCAAGACTCAAGCTGCCTCTTTTACCTTACTCAACGGAG
GAGTTTTTGGGGGTTGTGTGGGAGGTTAGGAACAGGAAGCCCGTGATTGACTGGGAGCTGTTTGTGGCCA
CGGCATGGGGTTTATGGAATCAAAGGAACACGGTCAGATTTGGGGGTCAGCGCAAAACTGCTCTCAGACT
TTGCCGTGATGTGGAGGAATATGTGAAGGAGTTTCGTCATGAAAATCCCCCCCCGTGTAAGCTCCCTAGA
CCCTTTACTCCCTCTTGGAAACCGCCAAAACAGGGCTGGTATAAAGCCAATGTGGACGGGGCTGTATGTA
AGGAGAGGGGCTGCTGTGGTATAGGTGTAGTAATTAGAAATGCAGAGGGTCAGCTGATGGGGGCGATGAG
TAAAAGGCTGGCGCTTCCCCTTAAGGCCTTGGAAATAGAAGCTATGGCAATGCAGGTGGGGATTCAATTT
GCGTGGGATTTGGGTTTGATGGATGTTGTGTTTGAAAGCGACTCCTTGACAGTAATCACAGCCCTTTTAA
GCGCTACTCCCCCACCTTGGTCAATTCAAAAGGTGATTGAAGGAACCAAGCAAAGTCTCAAGTGCTTCAA
CACTTGGTCTGCAGTCCATGTTCGCCGCAGTGGGAATGTGGCTGCTCATCTCATGGCAAAAAATGCCATT
ACAGTGTTAGATAGTATTGTATGGATTGAGGATATCCCACCCGTTATAGCAATGCAGGTCTCAAAAGATG
TATCAAGCTTGAATGCTATTTAGTTTTAATGAAATCTTCCAGAGTTTCGAATNGCCCCCCCCCCCCCCCC
CCCCCCCCCCCCAAAAAAAAAAAAAAAAAAAGAGGAACCCAGGCAACCTACCCTTTGAAATCTCACAATAA
TACAATACAACACTCCACTGTTAGTTTTTATGTATATTGTACATCTCACCCAAATAAAACAAACGCTTTT
AACAAAACCTAATAAATGCCTAGTTGCTACGTGTCCATCCTTTTTAATATTTTATAATAAGAATGCAAAA
GTGCCATGTAGTAAGTAATCTGCTTCAAAAAAAAAAAAATGTAGTATGTAATCAATGTTCAATTTCGAAGT
GCACTATGCACCGTGCACTACCTGAACCTATGAGGTTTTACCCGCCCACAAATTTTTTTTTTCTTCTCG
TTATCTCTCTTTTCTTTTTCTTTTTCTTTTTCTTTTTTTTTTTCCCTCTGGCTGATATTATCTTTTACTT
TTGGATTTAGGTGCTTGATAAGGTTCTAGCATTGAATTTTTTTTTAATTTTATCTTTTTCTAGAAGAAAA
TTTAGGTTGCAATAGAGATTTATTTATTTGAATAGGAAATCCTATAGAATTGAAAATGAATAAATTTTAT
TCTATGAAAGATCAACGTTGAACATAACTTTTATAGGAGGTACTTTTTTTTTGTTCATGTTTATAGCAAA
TTACTCATTTTTGTACACTGATTTGAGTTTAGAAATATTATTTAATTTCATTGATCATTTTGTATATAAT
ATATGAATGAGTTATGACTAACATATGAATAAGTAATTCGGCCCCCAAATAAAAAATCCTGCCTACGCCC
CCTGCTCACACACACATTTCTTGGTAATCATAGTCGTGAATATTTGTTATTCACTTCATTGGTGCAGAAT
GGTACTCGCTCACCTGGTTGGCCTTAGCACCACTAATTGTAAGACTTGAACAACTAGCTAGCCTTGCTGT
TTACTACGCGATTTGTTTGAATATCCTAACATACAATTATTGACATGATTTAAGCGTCCAACTGTTCCAT
ATGATATTTTCTTGTGCAGATAATTCTATTAGTGGGCACAAAGCTTGAACTTATTATCATGGAGATGGCT
CAAGAAATTCAAGATCGAACTGCATTTGTCAAGGGTGCTCCAGTGGTAGAGCAAAGCAACAAGTATTTTT
GGTTTAATAGACCCCAATGGATTCTCTATTTGATACATTTTACCTCGTTCCAGGTGAACATTGAAGTCCA
ATATGCTTCATACGCTATCCATAATTTTTTTTTTTTTTTGGGATAATCAAAATGGAATCCATAAAGATAAT
TAGAAACACCAAATTTGAGAGTTAAATGGGAGAAATAAAGTTTGAAAGTTAAAATAACACGGCAATATGC
TAACGAGTTTGTAGCTTGATTGGCACATACCCCTATCTCCCAGTCCTATGTTGCAAAATTGACTTGCCTC
CTCTCCTCCCAAAAAGAGAAAAATACTCATACTAATACTAATAATAACATGACATGGTTTTAGAGCAACC
AATCGTGTCAAATTGATAATATAACATGGTAGTATAGATGAATTAATGTGTAAGTCTTATTGGAGAAATG
TTTGTAATTTCTTTGTGGATGAATGATACTTATCGAACTTTTAGATACAATTTACCATGGCTTGTGTTGA
ACTCTATTCAAATTACATGCTTCTAAGTTCTAAGTTCTAACTCTGGTGTAATTTGCCTTCCATACAGAAT
GCTTTCCAAATGGCATATTTTCCGTGGACATGTATAAGTTGTCTTGAAGTCTATATTCTTTTTAACTCGA
TTGATTTTGTTGAGTTTAAAAACTTTTGATGAAATCAAATTCTAATGGATACTTGATTGCAAAATTGTTG
TAACATGAGATATGCAGTATGAGTTCAAGATCACATCATGCTTCCACGAAAACTTGCCGTTATTATTGAT
AAGAGTTTCCCTTGGTGACACAAGTGAGTAGCAATATCACCCAATGAGATGAAAACTTTTTGAAATATAG
TGCTATATCTTAAATTTATCAATTAAATTCAACAATGTGGTTTTATTGGTCAATAATTAGCCTAGTGAAA
AATAGGGAGAAATTACATTTTACCACTTTAAACTATATATACCTCTGATTAAACTTTGCAATTTAAACTT

TTCGAATGTATGTTTTGCATATAAACTATGACTCTTGTTATACTTTGCACACCAATGTTAAGTTTGTTGT
TAACTTGGATGAAAAAATATGACATCACATGAAAAGACTTATTTGCCCCTATTCTTAATGCTTTAAAAAC
AAAAGATAAATTATATTTTACCACTCTAAACTATACTTCTGATTACACTTTGCACCATAAACTTTAAATA
TCTATCCAAGCTAACAGCAAACTTAACGTCAAGGTGCAAACTGTAGCAAATGTCATAGTTTAAGATGCAA
AATATGCATTCAAAAAGTTTAGGATGCATAGTGTAAAATGAGGCATAGTTTAGGGTGGTAAAGTATAATT
TCTCTGAAAAATAGCTCTATTTGAGTTGCATTAGTTAATGAAATTATATAGTTGAATTTAATTAAAATAG
GTTCTTGTTCAAATTTAATGTTAACATAAATCTTATGCTAACTCAAGGTTTGACTCCTCTTCATATTTCT
AGATGGGTTCTCACATGAAGCAAGCTATCTTTGAGGAGCAAACAGAAAAAGCACTTAAGAAATGGCATAA
GGCTGTAAAGGATAAGAAAAAAATTGAGCTAAGGTGGAGTAGATCGTTCTTCAGGATTTATGAGTGGGGAA
AACACACCAAGCCGAGGGACATCACTCATTCACTTGCTTCACAAGCACCAGTCCAATAAATCAGATTTTG
AAGCTGATCTTTATTCCCCAAAATCTTACCAATCCGATACTGACCTTTCGGAGATTGAGGGTTCCACTCC
TGATAGGCATGCATCAAGAAAAGAAGGTCAACCAACAAGAACTGAAGAATCACATAATATTGATTTTACC
TTTGTTAAGGCTTGA

>NW_019809389.1:c180050-174695 *Quercus suber* isolate HL8 unplaced genomic scaffold, CorkOak1.0 scaffold_3886, whole genome shotgun sequence
ATGGCTGCAGCTGCTGCAGGAGAATGGTCATTGAAGGAGACACCTACATGGGCTGTGGCATTTGTTTGTG
CGGTTTTCGTGATCATTTCAGTTCTCATTGAACATGGGATTCATTCTCTTGGAAAGGTAAGACTTCTATG
CTAGTGTTATATAACGTACTATGGAACTTTGTGAGTTTTGACTTCCCAGAGTTAGTGTGTAACTGCGTAT
CTGGATGCAATTTGTTTTGTTAATTTATTGCTTATTTGCTAAAAGTTATTGTATGACAGTGGTTTCAGAA
GCGCCAGAAGAAGGCTATGAATGAGGCTTTAGAGAAAATTAAAGCTGGTATCTCTCTCTGTCTCACAACA
CAAACTTGAGCACACACATTTCCAATTAAAATTTTCATGATGGACCAAATGAGTTGACTTTTATTTCTCG
TTTTTGTTGTTGTTGTTGATATAGAGTTGATGCTGCTAGGCTTCATATCTCTGATAATTACAGTGAGCCA
AATACCCATCTCCAAGATATGTATCCCTGCCAAAGCTGGAAACATCATGCTTCCTTGTAGTAAAAAGGGG
TACACAGGTAGTGGTGGTAACAGGAGAAAGCTATTGTGGTATGACGAAGATGTTACATGGCGCCTAGTCC
TTGCCGCAACTGCTGGTGATGATTACTGCTTCAAATATGTATGTACAAATAAATTATTATAGGTTAACTT
GTATATTTGGTGCTCTACATTTTATCACTAATTCAAATTCGTTATTATCTTTAAAATGTTTTCAAAAATA
TTCTCATATCATCATCTTATCATGGAGGGAAAATATTGAAGTAGTAAACCAAATTTACTCTTATGATATG
AAAATATGATGTAACACGAATTAAAGGTTAAAAAAAGATGAAAATCAAATATCAATTTGAATTAAATATT
AGTTCTACAAAAAAAAAAAAAAAAAAATGACTTTTTTGCTTGGTATGGTATGCCTTGTCTTATATATTCGT
TGTTTTTGGATGTCAGGGCAAGGTAGCTTTGGTGTCACAGTCGGGGTTGCACCAATTGCATATCTTCATA
TTTGTGCTTGCGGTCTTCCACGTTCTCTATAGTGTCATCACCCTAGTTCTGGCAAAGGCCAAAGTAAGCT
ACTGCTCTGTTTTTTTCTACAACAAAATTCAATTCCATGAGGTTAAAAAAGGGGGAGCAATTAGTGAAGA
ATTTGACGTGCTAGATTCGTAATGTTACTTTAATTGACTTGTACAGATGAAGAAGTGGAAAGCATGGGAA
GCTGAAACCACATCCCTGGAATATCAATCTACTAATGGTAAAAAACTCATTTGAATTGGTGTGTTTTTAC
TGTTTATTTATTTATTATTATAAAAGAGTGATGTTAGAAATACTACAAAATTTATTATATGATCCTTATA
AACTTATGTGTTATCAATTATAATAAAGTAATTCAAACATTTATTTATTATATGGTTGAAGTGATCATAA
TCACATTGATAACCACATTTTCTATAATAAATTGGTAGCAGCTTAGTATTTAAGTGCTCGAGTTGGTATT
ATTGTAGATCTGTCAAGGTTTAGGCTTGCCCATCAAACTTCTTTCGTTCAGCGGCATAGCGGCCTCTCAA
CAACAACACCTGGTCTCAGGTGGATTGTAAGATGTATTCTTCTAAAAGTTTCTTGATACACGTTTGAAAA
GCACACGCATACACAATGAGACACATACAAACATTTGTGTGTATTGAGAAGCTTGTAAAATAGGTTGACT
TGCATTCTTGTTAAGTAGAAATTATTCAATACATCCATTGAATTATCCTTTCTTTGATATAAAAGTGGAT
TCCATATGTTACTTCTATGCATATGACACGTATTTAATATGAGAGGAAGGTATAATACACCGTGTTTAAT
GAATAATTTTTCCTTTTTATTGGCTTAAACATATAGAATAAACTCAAACCAAAGTCACAAGATACAATTC
ATAATTATCACTAGATGTCTGTCTAAACTTATATTTATGCTTGATCCAATAGCTTGGGCAATGGTTCAAG
GTATCATATATTGATATTGAATTAAGATGTGACTGGTTTCTTTTCTTTTTTTTTTCCCAAGTAAGAATGA
CATTCAAAAAAAGAGAAACAAACAAACAAATTATAAAAGGATGGAATCAATCCCATCTTCATCCTAAAGT
CCAAGCCAAAAATTTGGCAAGGAAGAAAAGGAGGAAATGGGGATCGTTCCCTCAAAACAATTCAAAGGAG
TCCCTTTAACCACATTACGGGTCACAAAATTAGCCTCCCTTAAAGTATGATTTACAAACCAATGTAAGAA
TAAATTCAAAATTAACTTAATTTTGTCAATAATATGTTGAATTGAGCAATGAGAAGAGAGTGAATCTCTT
TGTAGCGGAATAATCATATTTAACACATCTCCTTCATAAATTACATGCTTTTTATACCCTGATTTTGCTG
CTCTTTTTGCTTATGCTTTTAAGGGGTCTTGATCTTGTGCAACTCTATCCATGCTAAGATCTCTTTTTTT
GTTCTCTCAGGTGGGCTTTTTTAGGCAATTTGTTGCTTCAGTAACAAAGGTTGACTATCTAACTATGCGT
CACGGATTTATCAATGTATGTATTTATCCAAAAAGTATCTATCTCTCTATTAAAAAAAAAAAAAAAAAAA

AAAAACAACAACAACAACAACAACAGTGAAACACACGCATCTTTTTCTTTAATATTTGTTGGGGGTACAA
CTTCGTATTTTTGTTTTTGATCTTTACGCTAGCTAGGGAAAGCCTTTATTTAATTTTTTGGTAAGAGAAA
ATCTTATATTGAATACCGAATAAAGGATGTGTTAATTAAGACTTGCCTAGGCTTTCCCAAAGAAAAAAGA
AAAGAAAAAAAGAGTTGCCTAGGGACCTCTCTATATATATATATATATACTATTTCCTTTTCCCTTCTCAA
CAAGTTACTTGATTTTAATAAGAATGATAAATTAGATAATTCCCCCTATAATTTTGGGTGTTTAATATTT
TTCAATTAAATCATAAATTTTAAAAAAGTTCTATTCAAAACTCAAAACTTTCTAAACCATTTTAATGGCC
CCCTCACTGAGTGAAAATAAACAAAGAAAAGCAAGCAAAAAAAACAAAAGAATCAACTTCACTAAGCAAAA
GTGTGAAGACCATTCCATCAAATTCAACCGAAGCAAAATTATTGGCTACCAAATGCGGACTAAATTTTTT
GAACACGATTCTCACTTTCAGATTCCTGTGTCTTTGCTTTACAACTCAATTAATCACCATCTAAGTGCCT
GTTTGTTTGTCTGAAGGGCAAAAAGTGTTTTTTAAAACCAAAAAAATAAATTATCATGACAACATCAATA
TGCATGTTCCTGTTAAATTTGCAGGCCCACCTGGCACTTAATAGCAAATTTGACTTTCATAAGTACATCA
AAAGATCAATGGAAGATGATTTTAAGGTGGGCGTTGGTATTAGGTTGATCTCTTTCACACTCTCTGTCTG
TCTCTCTCTCTATGTTTTTGTTTTGTGCTAGTGCTACCATATAACCACGAATCAATGTTGATATTACAGT
ATACCATTGTGGACGTGCTGTATCATCTTTCTGCTTTTAAATGTCTATGGTAGGACATCTCACATCTTCT
TCTCTTCCTGTACTTTGTAGTTGTTGTGACTTTGTGATTTTCATTGACAAAACCATAATGCTGTACTTTG
AACAGGATGGTACACACTCACATGGATGTCATTCATGCCACTAGCGATAACAAATTTTTATATGCTTAAA
GAAAAAGAAAAAAAACCTTGCTTAATTTTGTGAATGTCTTTTTCAAATGTTTTCATCATTACAAAATTGG
ATTACTGGAATAGTAGAAAATAACTATGATTGATGGACCTCATTATGCGAGACAGATACTTCTACTAGTT
GGAACAAAACTTGAAGTTATAATCATGGAAATGGCTCAACAAATCCAAGATCGAACAAGCTGTAGTAAAA
GGGGCTCCTGTGGTAGAGCCTAGTAACAAATATTTCTGGTTCAATCAACCTGATTGGATTCTTTTTTTGA
TACGTCTTACCTTGTTCTAGGTAATTTATAATGCCTTATTCAAAAATTCTAAACATGACATTTTTCATAA
CTGTTAAGGTAATATGTTATGATTGGTGTATGATAAAAGTAATACTAGTAGTGAACCCAATAAAAATAAT
TTGTACTAATCATAGTTTGTCGTTTCAACAAGTTATGAAATTTAATTTGGTGTATAGCATTGTTGAAGAC
TTGTTAATTTCAAAGAGGTAAAACTGTAAATACATCAGCGTATGGGGTTATGTTAACAAATAATTTAATT
TTGAGTTCACTGAACCTGTATCAAGCAAATAAAAACAAAATATAAATATGAAATGAGTAAGCCTAATGAC
ACAACTTATTCCATAACCTTTTTACTCACATCCTATGAAGGTGGCGGGTTATGAGTAGTGAAAAAGAAAG
TAACGTTAGTGATTGGTCCATGTGAAAAATGAATGTCTATCCTTCACCACTAATCATATCAATATGTCAC
AATAAAAGCACTTATATATGTTAATCCTAAGCAGCTTTGGATCATGTGTGTTGACAAATATATTTTGAGC
TAATTAGTTTCCTTTCAATTTTACAGTACGCATTCCAAATGGCTTACTTTCTATGCATATGGGTAAATGA
AACTATTTCCAACTCCAGTTGCATACTGGTGTCTATAAAATCACTTATTAATGAAATTCTTTTTGATTAA
CTAATAAGATAATCCACTGCAGTATGAGTTCGGGTTGACCTCGTGCTTCCATGAAAAGTTGCCAGAAATC
TTGATAAGTGTGTTTCTTGGTGTAGCCCTTCAGGTCTTATGTAGTTACATCACCTTCCCTCTCTATTCCT
TGGTGACACAAGTGAGTGCTTCCACATTGAGGATTTTTATTCAAGATTAATAGAAATCAGAAAAATAACA
CTACTAAGCTTTGCAAAATTTGCAGATGGGGGTCTCACATGAAGAAGGCAATCTTTGAAGAGCAAACAGC
AAAAGCTATAAAGAAATGGCAAAAAGCTGCTAGGGAGAGAAAGAAGTTGAGGAAGGCAGGGGCGACAGAT
GTTTCATCATCTGGGTTCATGAGCGGAGAGAATACACCAAGCCATAACTCATCACCTATACATTTGCTCC
ACAAGTATAAAATCAATTCTAATGACATTGAAACTGTTCTCAGCTCTCCTAAGGCTTACCATTCTGATAC
CGAACTCTCTGAGATTGAAGGTCCAGCGCGCCCTTCCTCTAGTGATGACCATGAAACAAGAAGAGCGGAA
ACTCATCACATTTATTTCTCTTTTGTTACGCCTTGA

>NW_019812324.1:43424-51768 *Quercus suber* isolate HL8 unplaced genomic scaffold, CorkOak1.0 scaffold_6821, whole genome shotgun sequence
ACCCAGTTTTTCATATATTTGGAGTTGATGTTCGCACTGATTAGCGCTCAGTCTCGGTCAGCATATTTTA
AGCTTTAGCCTTTTAACCCCATAAATTTTCACATTTAATTTTCATTTTGGCTCATAAGGTTTTTCCAAAG
TGGCAGTGGCAGCTTGATAATCCCACCAGCTGCTTTACAGCTCCTCCATCCCACTCTTCTTATTATGATT
CTATATATTCTCTTAATTCCATTTTCTTGTTTCAAAGACAAGAATTATTTGTAGGAGAGAGAAGAGTGAG
AGTACTGCACTGCATCATAATTTTTGGTTAATTAGATATTGGGCAGAGATAGAGACAGAGACAGAGAAAG
ATGGCAGCTGAAGAAGGTCCAACTTTGGAGTACACCCCAACGTGGGTTGTTGCTGTTGTGTGCAGTGTTA
TCGTCGCCATCTCTCTCGCCGTCGAACGTCTCCTTCACTACACTGGCAAGGCATGTCAATCTCTTCTCTT
TTCTGCATCCATCGTATCATTTCTATTTCCTTATTAGTCTTAGTCTTATACTACTTTGGTAAAGGTACTG
ATCATCATTAATTATTTATATAATGAATGACTCTCTCTCTCTCTTTTGCAGTATCTCAAGAAAAAGAACC
AGAAGCCTCTCTATCAAGCCTTACAGAAGATCAAAGAAGGTCACCAGTACTCTCCACTCTCTCTCTCTCT
ATTACATGCACACACACACACACACACACACACACATACATATAGAGTTGGATATATTCTGGGTGTGCCTGA
TCATATCATTTTATGGTGTGAATGAATGCAGAGTTGATGTTGTTGGGGTTCATATCACTGCTACTCACAG

TGTTCCAAGCACGGATTGCCAAAATTTGCATACCCGAGAACTTAGCCAATAAGTGGTTGCCTTGTAAAAC
AGATGCATCTTCTACTACTACCCATTTCCAAACCTTGTTCACTTCTTTTATTCCCCGTCGCCTCCTCGCC
GAGGCAACAACTACTTCCACCGGTTATTGTGCTGAGGAGGTACTTAAAAACTTTTTCTCTGTCTAAATTT
TGGAACTTAACCGCAATCAGTGCTTCTATCCATTTTACTATTTGGCTATTTGGTGATTGATTATGGGGTA
TTCTAGTTATTTTAATCAAAGTAGATACGAATATATCACAAGGAAGCAATTTTTAGTGGAGTTAGTTGAC
TTTATCAACGCACCAAACAATTTTTATTTTTTTTATTTTTATTTTTTTTATTTTAGTTTGTTGTTTGTAG
TGTATTGAGTACTGTTTATTGGGGGGACAAGGGCCCATATGAATTCTTTTTTTTTTTTTTTTTTTAAAGC
AAACTACAGATAACCATTTTAATCATGACTTGACTTGGAATAATAGTTAATTTTTGAAGTCTTAAAAAAA
CAAATATATTATTGATTGGCGGAATCTTTCAGTCAGACTTCTCAAAGCACATGCATAGGTTGCAAATGTA
TGTGCTCTTCATATCTACATACTTGTTAAATCAACGTGCAGGGTGGATTGTTTGAAATGTCACATATTTG
GAATCCTACATTTGACTTTTGACTTTCTACTTTCATGATTTCTATAAATTGTAGTTTCTATCATCTTAGT
TCAATCCGATTTTGAAACATAAAGGATCACTTTGATTGGATTGACAGTTTACTCCCTCTCTCATTTCTGC
AAGACAAAATTTACATTATCTTTTTGGTCTGGATGATTTTACAGGAAAAAAAAGTTGTTATCACATTTGC
CATCAATGATCTTCATATTAGGTGCAGAATTAGAACATGTTATAATCTCCAGTATGGTTAATCAACTTAA
CCTGGTATAAATAAGAAGATTTGAGAACCAAGGTTCTTACTGCTCATGTTTTAAATACTCCCAAGCATAT
ATGTTTGCTGTTTCAGGGCTTTCCCTGTCTTCCTGGTCACTTATTTAAGATTTCATAGTAAAATGACTTA
GAATTTGGAGATTCAGCTATTATGAAATCAGCCTCCTGGGGCAGGATGGTTAATGTGCTCATGTGAGTTC
TAGCTTTAAAACGTTCAAGTTAAGTCTGTAAAAATCAAACCCCATAAGATACAGCAATGCAATTGTTTAT
AGGTAAAGCACACATATAATCAACATGATCATTTTTATTTTTTATTATTTTTATGCTGAGAATAATCAAG
TGATCATTAAAAGAGAGTAAATTGCACTAGATAGCTTGTCCTGAATTACCTTCTTTTTCCTGTATAATAG
AAAGATACAACTGCGCATAAGTAAATTGAACTTAGCTTCTATATGTAAGAGTCTTTCTTACCTGATGAAG
CACCAAATTTCTTCTGATATGCATTGCCAATGTAACAGCAAGGATGCAACTCCTTAAATAAATCATTAAA
CTTTCACAAAATATTGAGATTCTAGTCATGGATAAGGTTCCAAGCAGATATGTTTGACTGATTATTTTGG
ATATGACCTCACAAACTCTTACTAGCCAGGGTGCATTTTCAGAGTAAATTCTAAATTACCTTAGATGAAG
CATCCCATATGTTCCTATTGAAGAGTTTTTTGGTGTTCCAAAGAGCCAAAAAGGTTTTCCTAAAAAGTTG
GTCGTTTAATCTTTGGAGTGCTTATTTTTTTCAACTAATTCCTGCAATTGCTAGTTATATCTTCAGTACCC
TTATATGATACACCTGATAGCCTTAGAGCAATATGACATGAGGTGATTGTTAATAGCAGAGTTCTCTCCA
AAGCAGTGAAAAAGAATTGCAGAGTATACATACCCCTTTGAGGCTATAGGACATTAATTACTGTTCCTTA
TTCTTCCTTCTTTTGAATTGTCTTTGTGTATTATAGACTCTTGTTCAATCTTCTGCAATAGCTAAATCTT
ACTGGTAGGAAGCATGTATTACAAAATTGTCAGCTAATGTTAAATTGTTAATACAAACAAAATATTTTTA
CTCTTCACTGTTTGAAATTGAATAGATCAACATCTTCTAGTGTGGACAGAATTATGTAATTTTTTTTTCC
CAATTGCGACCTTTGTGGGTTATAACTTATAAGCATGCATAACTGAACTCAGCATCATATAAAGTTATTA
AGATCTTCAAAGAATGCTCTAGGAATGCAATCAAAATCTAATTTGTTAAATAAGGTTGCACATGTGCTTA
TGAATTGAAATATCTTATTATTTTTTTGCTTTCTTTACATCATTTTTTCAGGGAAAGGCCCCACTGTTAT
CTCTTACTGCATTGCATCATCTTCACATATTTATCTTTGTGTTAGCTGTTGTGCATGTGACTTTCTGTGC
TCTAACCATTCTTTTCGGAGGAGCAAAGGTTAGTTGTCCCTATTCTTATAATTATCTGTCTATTTTTTTT
TTCTTTTTTTTAATTATAACCAAGAATTTTTATTCTTTTACCTTAGTGTCAATTTTTCTAAAATATATTT
TAATGGTGAAGATACGTCAATGGAAACATTGGGAGGATTCTATCCTGAAAAAGGAAAACGATCCAGAAGA
AGGTAATGCTCAATTTCTCATTAAAGTTGAAGTCTACATACTTTCCTGTGTTATGTTTCCTTCTGCTGAA
TCCCAGATCATAAAATAAATTTATTCCTTACCTCAATTCTTTTCGACTGAATGATATAGAATTGAGTTGC
TCAGAATTTTTCAATATGGACTGAAATTTCATCATTCAACCTGAAGTGTGTGCATTTTCTTTAATATCCA
ACAACTACTTGCTGTTGTTTGCATCTTTTCTCATGAAATACTGTATTTGCAGTTCTGAAAACAAACTTTA
CACAAGTCCAAGATCACGATTTTATCAGGAATCGCTTTCTGGGTATTGGCAAAGATTCAGCTTTGTTAGG
ATGGGTGGTAAGTTATAGAACCCTTTATAATCAAGTCAATCTACCTGTTTGACAACTAATACCAACTATG
ATTACAACTGCTAATAATTTGATTCATCAATGTTATTAAGAAGGATAATGTAATGATCATACTTATCAAT
AGTTTTTCATGATGGAGAGCATTCATTTCACTATGGTTTCTTGGTCTCTCTTCTTCAGAATATAAAATTC
ATTGATGAAATTTGTGAAAAATTACAAAGTGACCATACCGACCATTTCCATTTTAAATTTGTTTGATTAA
AAGTTAGCTTACTAAGATTCATAACTTAATTGTTTCCAATTGTATCCCAGGTTGATTTATAAAAATCAGG
AAGAAGAGAAACCAGTATCTTTTACCTCTTCTTTTATGCCAGACTACACAAATTTTTTTTTTTGAAACCC
ATGTACTACTCAAATGTTAACAAGCCTGAAATGCTTAGGGAAAGGTCAGTTTCAAAGGAAAAAAGAACAA
GAAAATGAAGGGGAAAAAAAAAAAGAAAAAGAGAGAGATTAACAAAAAATGATTTCACTCTGAAAAAATGT
TGCAGATCACCTTTCCTGCGCAGTTTATGTAATAATAATCTAGAATGAAATATTTCAGTTACAACATTAG
AATATCACTTCTACAGCCATAAAATATATAAAAGATAGAAATCATTTCTGGGATGGTTGCCTAACTTGTA
ATGGACATCAGGATTGGGCAGTTACAACAAAATACATGGCTAGCAGTTTTGTGAGATGGTGTGTTTTATT

ATCCATAAAAAAAAAAAACAAAATACACGAATTTGTTCTTCTCTACAATTACAAACAAATGATTTTTTTT
TTATGACATCATCATTTTCAGCAATCATTTTTCAAGCAATTTTATGGGTCTGTGACCAAAACAGATTATC
TGACAATGCGACTGGGCTTCATTATGGTAAGTTATATAAAGGTCTATATGTATTCAATTTATTATTCTTC
TTTCTTTTTTTATTTTTTTCAAAACCCCATTACTTTTTACTTCACTAGATAATCTTTTTGATTTTTAATA
TCTTTCTTTTCTATAGACTCATTGCAGAGGAAACCCACAATTTAATTTCCACAAGTACATGATTCGTGCC
CTTGAAGCTGATTTCAGGAAAGTGGTTGGAATAAGGTTAGTCACAATTATTAAAGACATTCTAAGTGCAC
TCATTATCTTATTTTCTATATTTTGAGCTCTACTATTTCTAATGTGGAGTACATCTTATAAGGTTAAACC
AATAAAAAAAGTACAGACTTAGAAGGTTTGTGCTTATGCTCAACCTCTTTTGTTTGAAAAATGTTAGTAC
TTCTATTTTCCATGTATCATATTACAATATACTAGCTCTCCGTGCACTAAGTACTTTCTAATCTGATTCC
TCTAAGTTGATAGATCCTACTAAATTCTAAGTTCATCAATTGCAGTTGGTATCTTTGGTTATTTGTGGTC
ATTTTCTTGTTGCTGAATGTGGCTGGTAAGCTCTCTCTCTCTCTCTCTCATTAACAAAGTGTAACAGTTT
TTTTGCACATGATTGAATGTATGGCCTTAAGGCCAACCTTAGCAACACATTTTTTCTATTCCAACTCACA
TTTATAATATGGTCCTAACCCAGGTTCTGAAAACAGTGTAGAAATTTTCGTTGTAGAGGTTTCGAGATCT
TATCCATTTGACTTACATTTCTATATCTAATTTCTATTGAAAGGAACGTCATGTTCCATGAATTATTCAT
CAAAATTTCTGAGTTCCAGTGACTATAATGCCATAGTCCTTGTCTCTGTTCTAGGCTCAGCTACATGAAG
ATCATTATCTCCAATCCATAATGTATTCTGATTTTTCTTGCAGGTTGGCATGCGTACTTCTGGATTGCAT
TCATACCCTTCATTGTAAGTTATATGTCATAGTTTTCCTGCTAAATTGCTTATATATTTATGCAATTTGA
TTAGTCACAATTTTGTGAAGATTATACCTTGTAAAAACATTTTGATTGCCAACCTTGTTTTTTGTTTGTG
CCGGAAGAGAAGTTCCAGTAAAGTTTGTTTGTGCACTAGGTCACCCTAGAATTTAAATCTTGATGGCCAC
TAGCTCACATGACAGATTGTGCTTATGTGTTTTTTTTTTTTTTAATGATTGGATTAAAAGGAACTAGCCAG
TCTTACATTAACTGCATTTTCTGAGGTAAATTCTTTTTGATATACAAGGTGGCTTTTGTATGTAAGATGT
TATATATATAATTGGTTTTTAACCAAATAAAGCTGTGTCATTGACAGTAGGTATCTAGTTCTTCCTTTGA
ACACTTCTAACCTATCACGAGGAGCTTGTAATATTTTAAAGCTGAATGTTACAGTTTTACAACTAGTGTA
GATCAAGGAAAAATTGTGGTTGTCAAATATAAAATGGAGTCCTAGATCATCAATTCAAAGAAACCATCAT
ATGTCCCCTCAAAAGCTTGACATGCTTATCTAGGATCAAATCATTAACATCTATTGTAGCTGCAATTAAG
CATTGCTACAATTCACCCAAGCTTAGGCTCTATAAAAGGAGTCTTATAATCTTGCAGGTGCTTCCAGAAA
TTTAAGGAACTCTCAGTGGTACGAGATAATTTTTGGGCAAGAATTTGGTATATACATGCTAATCTCCCTA
AAAAGGAACCTGTGAAAGATAATAATTATTCCTGGTTTCATTAAAATAATACTTGTGTACTAGTATTTAC
AAATATAAATCTGTTAGAGATATTGGTTAGGGATTTTGTTATGTCTAGATGAATTAAATTTCACAATTAT
GTTGTCAAGATTAAATTCTGTACCCTTCAATTTTAATCCATTTCATCAAGTCCTAACACATGTTTTTGTG
TGTGTGTGTGTGTGTGTCAAAAATATAAGGAAGATTGCATGCACTATTTGAATGGCTGAAATTGTTCAAT
TTTGAGTGTGTGCAATTTCTACACTAGAAGGAAGACTGGATATAGACTTTGTATACCACTTTATGATATG
TTCCTCATCAGTCAACCATGCCACCTTGCAGCTACATGTTATCTATACTGTTATAGTTGAATGGATGGAT
TTCAGGCTTTTCTTTCAAAGATTTTAGGCTTTTCAGCTTCTGAAGATAAATGTGTTAGCTCATTTCTAGT
GTCTTTTCCATACTTTTGTTTAACTTACCATGCTCTTTAGTTGAAAATAACAATCAAATAAATGATTCAT
AATAATTGTGCATTTGTCTTCTCAAATCATCAAACTACGTGTTCATCTTGTAAATGTCCTAGATAACATG
TAGGGTAAGGACCATAACATTGATATTGTTTTTTATATTTTACAGCTTCTACTTGCTGTGGGCACTAAGT
TAGAGCATGTCATTATCCAGTTGGCCCATGAGGTTGCAGAGAAACATGCAGCAATTGCAGGTGCTTTGGT
AATTCAACCTTCAGATGATCACTTCTGGTTCCATAGGCCCCGGATTGTTCTCTTGCTGATTCATATCATC
CTGTTTCAGAATTCATTTGAACTGGCAGTTTTCTTTTGGATATGGGTAAAACAATTAAAATTCAATATTC
ATTGCATATTAATGAGACAAAGATAAATTGGAAATGAAAACTATGGATTGAATTTCTATGATGCTTCAAT
ATGTTACAGGTTCAATATGGATTTGACTCGTGCATAATGGGAGAAATCAGTTATATCATCCCAAGACTTG
TTATAGGGTAAAACAATGATTAATTATTCTATTCTTGAAACCATATGTTTCTATTGATGAACTGAATCTG
ATGTTAAATGCTAATTTTATTCATAGGGTATTCGTTCAGTTCGTCTGCAGTTATAGTACCCTACCTCTGT
ATGCAATTGTCACACAGGTGAGTTGGATCATCACTAAGAATTCACCTTCTCTATGATCAATTAACTTATG
TTGGGATTTTATGCTGATGAAGATTGCTGAATTCCTTAAAATGTAGATGGGAAGTTCATTTAAAAAGGCT
ATATTTGAAGAACACATACAAGAAGGGCTTGTTGGTTGGGCTCGAACGGCAAAAAAGAACAAGGGTTTGA
GAAAGGCTGCTAATGGCTCAACTACTACTACTACTACTCAGGGTTCTACTACTCATGCATCTGGCCAAAT
GAGTCCAAAAGAAACTACTCCTTTGGCAATTCAGATGAATGAAGTATCTGCAATGGAAGAAGGTAATGCA
GGAGAGATTGCAACTGCAACTGTCTACGATGGACATAGATAAGGGCATCATAGTCTCACTCAAAACTCTT
GTAGGTAGCCGGGTGTTTTTTACTTCTTGTGGTTAAGGTGAAGTTAGAAATAGTTGTGTTTTGATATATG
CTACATATTTATTAAAATTGTTCAGATGCATACTATATAGTTAGTTTATTGATTTTATTTCTTTTGTAGT
AATGATGATGATGATGTGTAAAATGATTATATAATTTTGATGTAAGTAGTTAAGTACTAAGTTCAATGGA
ATTGTTTGCTAGCAA

PMR4;

>NC_003075.7:1573266-1579820 *Arabidopsis thaliana* chromosome 4 sequence
AAGAAAAGGAAATTCAAATTATATTGTTGGAAAAAAATACAAATCAAAGACAGCAAAAAAAGTAACAAAA
AAAAAAGGTAGAAGAAACTGAAACGCGGAAAGGAGGCAAAATCTTCTCGTCGTCGTTGTCGCCGTCTTCA
GAGCTACAAACGAAAAAACTCGCTTCCGTTTCGATTTCTCCATTGTTATTGTTTCTTCAGTGAAGCTTTT
TTCTTCGAGAAATTTCTAAGATCTACCACATGCTACTATGAGCCTCCGCCACCGCACCGTCCCGCCGCAA
ACCGGACGGCCGTTGGCGGCGGAAGCTGTCGGAATCGAAGAGGAGCCGTACAATATCATTCCCGTTAACA
ATCTCCTCGCCGACCATCCTTCACTCCGTTTTCCCGAGGTTCGTGCCGCCGCTGCTGCTCTTAAAACCGT
TGGAGACCTTCGTCGTCCGCCGTATGTTCAATGGCGTTCTCACTACGATCTCCTCGACTGGCTCGCCTTG
TTCTTCGGTTTCCAGAAAGATAACGTTCGTAACCAGCGTGAGCATATGGTGCTTCATCTCGCAAATGCTC
AGATGCGTCTCTCTCCGCCGCCGGATAATATTGATTCTCTCGATTCCGCGGTTGTTCGTCGGTTTCGTCG
GAAACTTCTCGCTAACTACTCTAGCTGGTGTTCGTATTTGGGGAAAAAATCAAATATCTGGATCTCAGAT
CGGAACCCTGATTCGAGACGAGAGCTTCTCTATGTTGGACTCTATCTTCTCATTTGGGGAGAGGCTGCGA
ATCTTCGGTTCATGCCTGAATGTATCTGTTACATCTTCCATAACATGGCCTCTGAGCTCAACAAAATCTT
AGAGGATTGCCTCGATGAGAACACCGGCCAACCTTACTTGCCTTCTCTCTCAGGCGAAAACGCTTTCTTA
ACCGGCGTCGTTAAACCTATTTACGATACTATCCAAGCTGAGATTGATGAGAGCAAGAACGGTACAGTTG
CGCATTGTAAGTGGAGGAACTACGACGATATCAATGAGTACTTCTGGACTGATCGGTGTTTCAGCAAATT
GAAATGGCCGCTTGATTTGGGAAGCAATTTCTTTAAGAGTAGAGGCAAAAGTGTAGGGAAAACTGGTTTC
GTGGAGCGCAGGACGTTCTTCTACCTTTACAGGAGTTTTGATCGACTTTGGGTGATGCTAGCTTTGTTCC
TTCAAGCCGCCATTATAGTAGCTTGGGAGGAAAAGCCAGATACCTCGTCGGTAACAAGGCAGCTGTGGAA
TGCTCTGAAGGCAAGAGATGTTCAGGTGAGACTATTGACCGTGTTCTTGACATGGAGTGGTATGCGACTC
TTGCAGGCTGTGCTGGACGCGGCTTCACAATATCCCCTCGTTTCCAGAGAGACCAAAAGGCATTTTTTCA
GAATGCTGATGAAGGTTATAGCTGCCGCAGTTTGGATTGTAGCTTTCACTGTCCTCTACACTAACATCTG
GAAGCAGAAGAGGCAAGACAGGCAGTGGTCCAATGCCGCGACGACTAAGATATACCAATTCCTTTACGCT
GTGGGGGCCTTCTTGGTGCCCGAAATCCTGGCTTTGGCTTTGTTTATTATCCCATGGATGAGAAACTTCC
TGGAAGAGACCAATTGGAAAATATTCTTTGCTCTAACTTGGTGGTTTCAAGGCAAAAGCTTTGTGGGTCG
AGGTTTGAGAGAGGGTTTAGTGGACAACATCAAGTACTCGACTTTCTGGATCTTTGTCCTAGCTACAAAG
TTTACATTTAGTTACTTCCTGCAGGTTAAGCCAATGATTAAACCCTCAAAGCTGCTATGGAACTTAAAGG
ATGTCGATTATGAGTGGCATCAGTTTTATGGAGACAGCAATAGGTTTTCTGTCGCATTGTTATGGTTGCC
AGTTGTGTTGATATATCTGATGGATATCCAAATTTGGTACGCAATCTATTCTTCGATTGTTGGTGCTGTT
GTTGGGCTGTTTGATCATCTGGGGGAGATCAGGGACATGGGACAGCTGAGGCTAAGGTTTCAATTCTTTG
CTAGTGCTATTCAATTCAACCTAATGCCTGAGGAACAACTCCTGAATGCTAGAGGCTTTGGTAACAAGTT
CAAGGACGGCATTCATAGGTAGTCCGTGGAAGCATGCTACTAATATTCCTAAATAATTTTCTGTACAACG
CTTGACTTGACTGTACAAGCTGTGAAATTTTACTTTTGTTAACGCAGATGCTGCATATAATTAAATTTTT
CAATATTGTAATAACTTGAGGTTGTGTACTGTATGCAGATTGAAGCTAAGGTATGGATTTGGGAGGCCGT
TTAAGAAACTTGAGTCGAATCAGGTCGAGGCCAACAAGTTTGCGTTGATCTGGAACGAAATCATCTTAGC
TTTCAGAGAAGAGGATATAGTTTCTGATCGTGAAGTAGAGCTACTGGAGCTGCCAAAGAATTCCTGGGAT
GTGACGGTTATTCGCTGGCCGTGTTTCTTGTTGTGCAATGAGCTTTTGCTTGCACTGAGCCAGGCCAGAG
AGCTGATAGACGCACCTGATAAATGGCTGTGGCACAAAATATGCAAGAATGAATACAGGCGTTGTGCTGT
AGTTGAGGCATATGACAGCATCAAACATCTATTGCTCTCAATCATCAAAGTTGACACTGAAGAACATTCG
ATAATTACGGTCTTCTTTCAGATAATTAATCAGTCCATTCAGTCAGAGCAGTTCACCAAGACCTTTAGAG
TGGACCTGCTGCCAAAAATTTATGAAACACTGCAGAAATTGGTTGGGCTGGTAAATGATGAGGAAACAGA
TAGTGGGCGGGTGGTGAATGTTCTGCAGTCTCTTTATGAGATTGCAACTCGACAGTTCTTTATAGAGAAG
AAGACAACTGAACAGCTATCTAATGAAGGTTTAACTCCTCGAGACCCAGCCTCAAAGTTGCTGTTTCAAA
ATGCTATTAGGCTTCCTGATGCAAGCAATGAAGACTTCTACCGGCAGGTTAGGCGTTTACACACGATTCT
CACCTCTAGGGACTCTATGCACAGCGTCCCTGTGAATCTAGAGGCGAGACGGCGGATTGCTTTCTTCAGT
AATTCGCTTTTCATGAACATGCCTCATGCCCCTCAGGTTGAGAAAATGATGGCGTTCAGTGTTCTGACTC
CATATTACAGTGAGGAAGTTGTATACAGCAAAGAACAGCTCCGAAATGAGACTGAGGATGGGATTTCCAC
CCTATACTACCTGCAGACAATTTATGCTGATGAATGGAAAAATTTCAAGGAACGGATGCATAGGGAAGGA
ATCAAGACAGATAGTGAGTTGTGGACAACCAAGCTGAGAGACCTCAGGCTTTGGGCTTCCTACAGAGGTC
AGACATTGGCACGTACAGTTCGTGGGATGATGTACTACTACCGGGCTCTTAAGATGCTCGCTTTTCTTGA
CTCTGCGTCTGAAATGGACATTCGGGAGGGTGCTCAGGAGCTTGGTTCAGTGAGGAATTTGCAGGGAGAA

CTGGGTGGTCAATCTGATGGGTTTGTCTCTGAAAACGACCGATCTTCCTTAAGCAGAGCAAGTAGTTCCG
TGAGTACGCTGTATAAAGGCCATGAGTATGGGACTGCATTGATGAAATTCACATATGTTGTGGCGTGTCA
GATCTACGGGTCTCAAAAAGCAAAGAAAGAGCCTCAGGCAGAGGAAATTCTGTATCTGATGAAGCAGAAC
GAAGCTCTCCGTATTGCATATGTGGATGAGGTGCCTGCGGGAAGAGGAGAGACTGATTATTACTCCGTTC
TGGTGAAATACGATCACCAGTTGGAGAAGGAAGTGGAAATATTCCGTGTGAAGCTACCTGGTCCAGTGAA
GCTGGGCGAGGGAAAGCCAGAGAACCAGAATCATGCAATGATCTTTACCCGTGGTGATGCTGTTCAGACC
ATTGATATGAACCAAGACAGTTATTTTGAGGAAGCTCTCAAGATGAGAAATTTGCTCCAGGAGTACAACC
ATTATCATGGTATCAGAAAACCAACTATTCTTGGTGTCAGGGAGCATATCTTCACGGGATCAGTCTCGTC
ACTGGCGTGGTTCATGTCTGCTCAGGAGACAAGTTTTGTCACTCTTGGTCAGCGTGTTCTTGCAAACCCA
CTGAAGGTCAGAATGCATTATGGCCACCCTGATGTATTTGACAGATTCTGGTTCTTGAGTCGAGGCGGCA
TCAGTAAGGCTTCCAGAGTTATAAATATCAGTGAGGACATCTTTGCCGGGTTTAACTGCACGTTAAGGGG
GGGAAACGTCACCCACCACGAGTACATTCAGGTTGGGAAGGGTCCACAATTTGGATTATTTCTAACTAAC
TATACTGCTACAACGTTTTTTTAACGTTTTTAACGTTTATTAATTATGCAATCTACTTTTGTTATAATTA
TGTAATTTAACGTTTTTTAATCTTCTAAATTCAAAAAATTTGAGTAACCTTTGTCTTTATGCATTTTTCA
GGTCGGGAAGGGACGGGATGTTGGATTGAATCAGATATCAATGTTTGAGGCTAAGGTAGCCAGTGGGAAC
GGAGAGCAGGTTCTCAGCCGAGATGTGTACCGGCTCGGGCACAGGCTTGATTTCTTCAGAATGTTATCAT
TTTTCTACACAACTGTAGGGTTTTTCTTCAACACAATGATGGTCATTCTTACTGTTTACGCTTTCCTCTG
GGGACGGGTTTATCTGGCTCTCAGCGGGGTTGAGAAGTCCGCTCTAGCAGACAGTACGGACACCAACGCC
GCGCTTGGGGTGATCCTGAACCAGCAGTTCATCATTCAGCTCGGTCTGTTCACTGCCCTGCCAATGATTG
TTGAATGGTCTCTCGAGGAGGGTTTCCTTCTAGCGATATGGAATTTCATTCGAATGCAGATTCAGCTTTC
AGCTGTCTTCTACACATTCTCAATGGGGACCAGAGCTCACTATTTCGGTCGAACTATTCTCCATGGTGGG
GCCAAGTATAGAGCCACTGGACGTGGATTTGTTGTCGAGCACAAGGGATTCACTGAGAACTACCGACTGT
ATGCACGCAGTCACTTTGTGAAGGCCATCGAGCTTGGGCTGATCCTCATAGTCTACGCTTCGCACAGTCC
GATTGCCAAAGACTCGTTGATTTACATAGCCATGACTATCACCAGCTGGTTTCTTGTGATTTCATGGATA
ATGGCCCCATTTGTGTTTAACCCATCAGGATTCGACTGGCTTAAGACAGTCTATGACTTTGAAGACTTCA
TGAACTGGATCTGGTACCAAGGCAGAATCTCAACGAAATCTGAACAAAGCTGGGAAAAATGGTGGTACGA
GGAACAGGACCACCTGAGAAACACCGGGAAGGCAGGATTATTTGTGGAGATCATCTTGGTCCTCCGGTTT
TTCTTCTTCCAGTATGGGATTGTATACCAGCTTAAAATTGCAAACGGATCCACCAGCCTTTTTGTCTACT
TGTTCTCATGGATATACATCTTTGCTATATTTGTGCTCTTCCTAGTCATCCAATACGCCCGTGACAAGTA
CTCGGCAAAAGCTCACATACGGTACAGGCTTGTCCAATTCCTCCTGATCGTGCTTGCTATACTGGTGATT
GTTGCTTTGCTCGAGTTCACGCATTTCAGCTTCATCGATATCTTCACAAGCCTTCTTGCATTCATCCCAA
CTGGCTGGGGAATTCTGCTGATCGCACAGACTCAAAGGAAGTGGCTGAAGAATTACACTATTTTCTGGAA
TGCTGTTGTCTCTGTTGCTCGCATGTATGACATATTGTTTGGGATACTCATAATGGTTCCAGTAGCGTTC
TTGTCATGGATGCCTGGATTCCAGTCAATGCAAACGAGGATATTATTCAATGAAGCTTTTAGCAGAGGAC
TTCGCATCATGCAGATTGTCACTGGGAAGAAATCAAAAGGCGATGTCTAAGTTTAAAAAACGGTAAAGCT
CCTTGTTCTCAACACCTTATGTTATGATCGTTTAAATCCTGGATTTCACACCAATGCGGGCTTTAAATTT
GTGTAGGTCTTAAGAAGTAAATGGTAGTTCAAATCCTATTGGTATGTGGCGAAGGAATCAGTTGGAGGTT
AGTTTTCCCGAAACAACCGAATTCGAAGTTTTGTTTCGTCTAAAGAAAAACTCAGATGCTGATGATTTAT
CTTTGTATTTTAAACAGGTTTTTGGAGAGTTTGGTTGGATGAGGAATCGGGAAGTTGGTTTGATTCGGTT
AGATGGGTTTAGGGAGATATTTGATTGTCAGTGTGTGTGGAGGGAACTCTGATTCTTGTATGGTTTTTGT
TCTAAAGGTACAGCAATTTGTGTAGTGAGGCTTTGTGTATTTGTTCTCCTTCTCTCATTATAGAGCTTTA
GAGCATTTTTAGTTTATATTCAGATTGTTATCTAATGTCATCTCGCAGAGCTTTTGTTCACATTTCACAT
CTTTTCTTCTCCTTCTTAGTAGAGATCAGTTTCAGATTAGATACTTGTCCATATTCCACTACTCTCGTCT
ATTATCGGTTTCTGCTTGTCAATTTCTGGGTCCAAAATTGAAATA

>NC_003074.8:2923518-2925889 *Arabidopsis thaliana* chromosome 3 sequence
CATATTGGCAATATTCAAAGGTCAACACCAACTTTCCTCCTCGCTTCCTCACCACAAAATATAAGTTAAC
GACGTCGCTCTAACGCTGTGTAGTAGTCTTATAAATACCCCTTCAACGACTAAATCTTCTCCCCTCATAA
ACTCTCTCACAACTTGCATTATCGTTTCAAAATTTTCTCTCAACGACGAAAAACTGAGTCCTCTAGAAAT
TTCCACGACGACTTGATGATGACGAGAAAAGCAATGATAAACATTTGTTGCGTGGCCACAACGCCGTCCA
AGTATCCACCTCCTCCTCCTCTTACGTTAACCCCTCGCCAGAGTTTGGAATCGGCGGCTATCTATGGAGT
CATTGAATCCGCGGCGGATATAATCGAGAGATGGAACACTGAGACCAACACTTTCGCTAAAGTTACTTGT
ATGTTCTACGAGAACAAACCTGAAGCCATGATGTTCATCGAACGTGTGAAGGATCTTCAGAAGACGATGG
ACGTACTCGTGAGTGAGGATCCAAACTCTGAGAGACTCTTGAGATCGCATAAGCTTATGCAAATCGCTAT

GAAGAGGTTGCAAAAAGAGTTTTACCAGATTCTTTCGATGAATCGTGCTTATCTTGATCCAGAATCAGTG
TCCACTCGATCTTCACTTACTTCCGCAAGATCTAGCTACTCAGATTTCCCCGACTATGTCGAAGATCTAG
ATACGATCATCGAACTGGAGGAGGTTTCTACCAACGTCATGACGGATTTGAAATCCATCGCTGAGTGCAT
GATCGGTTCTGGTTACGCTAAGGAGTGTCTAAGCATCTATAAAAGCATTAGGAAATCGATCATCGACGAA
GGGATTTATCGTTTAGAAGTGGAGAAGACGAGTACTGGGAAGGTGAAGAAAATGTCATGGGAAGTTATGG
AGTTGAAGATCAGAAGCTGGTTAAAAGCAGTCAAAGTTTCTATGGAGACTCTTTTCAAAGGTGAGAAGAT
TTTGTGTGATCATGTCTTTGAATCCTCTGACGCAATTAGAGAGTCTTGCTTTAGTGATATCTCTCGAGAC
GGTGCGCTTCTTCTCTTTGGATTCCCGGAGATCATTAATACTAAAACTAGCAAGAAACACTCTCCGCCGG
AGAAAGTTTTCCGGCTGCTTGACATGTACACCGCCATCGCCGGAAACTGGCAAGCGATTGAGTCTATCTT
CTCGTTTGATTCGATCTCCGTCGTCAGATCTCTTGCTCTTAAATCTCTAATCTCTCTCAGCGAATCAATT
CGATCACTACTTGTGGAATTCGAATCCGGAATCCAGAAGGACTCGTCGAAGGTGGTGGTTCCCGGCGGAG
GAGTACATCCACTGACGATCTCCGTCATGGATCACCTCTCTCTCCTCGCTGACTACAGCAACGTACTAGT
CGATATCCTCGCCGGATCTCCACCACCGGACAGGTCGCTGTTACCGGAGTCTTACTTCAACGTCTCAGAA
TCCGACGACTCTCCTTCGTCGGAGCTGACGATCCGTTTCGCTTGGATCATCCTCGTCCTCCTTTGTAAAA
TCGATCGTAAATCAATTCACTACAAAGACTTCTCTATACAGTATCTCTTCCTCACCAACAATCTCCAGCA
CGTGGTCTCACGTGCTCGTTCCTCAAACCTGAAGAACCTTCTCGGCGAGGATTGGATCACGAGACATTTC
GCTAAGATGAGACAATTCGCCGGCAGCTATAAGCGGCTAGCTTGGGGACCGGTGGTTGCTACTTTACCTG
AGAATCGTACGGTGGAGATGACGCCGGAGGAAGTGAAGGAGCGGTTTGAAAAATTCAGCGAGAGCTTCGA
AAACGCGTATAGTAAGCACAGTGTCTGCGTCGTAGCTGATCCAAATATACGAGACGAGATCAAAGTATCG
ATATCGAGAAAGCTGGTGCCGATCTATCGCGAGTTTTACAACACGAGAGGTTCTGTTATCTTGGGAGAAG
GCGATGGCGCGAGAAACCTGAACTCAGTTGTCCGATTTACCCCTGAAGATATTGAAAACTATTTGTCTGA
TTTGTTCAGAGAGAAGGGTAGTTCGGGGAATTCGTCAGCCTCTTCTCCTTCCTCTTGTCGGTCAAGGCAA
TCCATGTCCTAAAGTGAAGGAGAGTCTATAATCGTCTTAAATGTAAAAGCAAACACTAATTAATTAGTAG
TTAACCTTTCATGCCAAAATTGTTACGCAACAAACTATTTTTCATTATTACTATTTCATAAAGAGTAAAT
GGACAACGACATATAAAGTGAAGATAGCTATGTTTTATTTTTTATTTTTCCTTACAGTTTAAGGTAAAAA
TCTTCGGGTTTCTATTTCGAACATTATGTCACACATTTGTCTGTAATGTTTAATTAATGATA

>NC_015441.3:2412940-2414628 *Solanum lycopersicum* cultivar Heinz 1706 chromosome 4, SL3.0, whole genome shotgun sequence
AAATATACCTAAAAATCCTTTTTTTCTCTATTTGTTCTCTTTGTTCTTCTCCTTCTCTGAACTTTCACAT
CAATGGCTATAACCTTTCAATCTCCCATGAAACTCAGCTTCATCACTTCTAATGGCTTCTCAAATCCTCC
TTCTCTTTATCCCATCAATACCCATTTCTCATTTGGATTCAATCTCTCATCTGTCTCCTCCAAAACCCAA
ACCCATATCACCATACCCGAACCCGAACCCGTATTCACCTCCGTCAAGTCGTTTGCTCCGGCCACTGTTG
CTAATCTAGGTCCGGGTTTTGATTTCCTCGGATGCGCCGTTGATGGAGTCGGAGATTTTGTCACTCTTCG
GGTTGACCCAAATGTTAAAGCTGGGGAGGTTTCGATTTCTGATATCTCCGGTGCTGGAAATAGGCTTAGT
AAAGACCCTTTATCGAACTGTGCTGGAATAGCTGCTATTTCTGTTATGAAGATGTTGAATATACAGTCTG
TTGGTTTATCGATTTCGCTTGAAAAAGGGTTGCCGTTGGGTAGTGGACTTGGGTCTAGTGCTGCTAGTGC
TGCGGCGGCGGCGGTGGCTGTGAATGAGATTTTTGGACGGAAGTTGAGTGTTGATGATCTTGTGCTTGCT
GGGTTGGAATCGGAAACGAAGGTTTCGGGTTATCATGCTGATAATATAGCACCTTCGATTATGGGTGGTT
TTGTGTTGATAAGAAGTTATGATCCGTTGGAATTGATCCCATTGAAGTTTCCATTTGAAAAAGATTTGTT
TTTTGTGCTTGTGAATCCCGAATTCGAAGCTCCAACGAAGAAGATGAGGGCGGTATTGCCATCGGAGGTG
ACAATGTCGCATCATATATGGAATTGTAGTCAGGCTGGGGCGTTGGTGGCTGCGATATTGCAGGGGGATT
CGAGGGGTTTAGGGAAGGCGTTGTCGTCTGATAAGATTGTGGAGCCGAGGAGAGGGCCGTTGATTCCTGG
GATGGAGGGAGTGAAGAAGGCGGCGTTGAAGGCTGGGGCATTTGGTTGCACGATAAGCGGAGCTGGACCT
ACTTTGGTCGCGGTGACGGATGATGAAGAGAGAGGGAGGGAGATTGGGGAGAGAATGGTGGAGGCGTTTA
TGAAGGAAGGGAACTTGAAGGCTTTGGCTATGGTGAAGAAGCTTGATCGAGTTGGTGCCCGCCTTGTTAG
TAGCAATTCACGATGATCGATGGATCAATCAATCAATCAACTATGCCTCAATTCCAAACGAGTTTGGTAT
TAGTTGTATGAATCCTATATATATCTTTTCACTATATGAATTCATCATGCTATGTGAAAAATGATATGTA
GGAATTTGTGTGCAATGAATTGTTAGAGCTTCTTTCACTTTGTATTTTTCAAACATGTTTTGAAATGTTT
TTTTGAGCTGTCTATCGGAAATTGCCTCTCTGTCACACAAGATAGGGGTATATGGTCTGCGTACACTCTA
CCCTTCCCATATCTTACTTGTGGGATCACACTTGATGTGTCGTCGTTGTATTGATCATTGAGAAATCAGT
TGATTACCGAGCAAATAAAATAGTTAAAAAGTTGCCATTTTGAAATGGCTTACTTGTCAATTGATTAGCA
TTCTTGATTTGCCTTTCTTGAGGACATTTTGTTCAATACTTATGTTGCAATATATCGAATGTCACTTGCA
GTTTCGAAA

>NC_015444.3:c62521907-62512892 *Solanum lycopersicum* cultivar Heinz 1706 chromosome 7, SL3.0, whole genome shotgun sequence
TTGGCCTGAGGCGAAAGTACTAAAAATATTTGTAATTCAAAAAAATTTAAAGAAGCAAATCATTATAAAC
CACGCGCATTGCCACGCGCCGCTTCTCAGTTTTGCCATTCATCTTCTATGCTCTGTATCCGTTTACACAC
AAAAGGGGATTTTCCTTTTTAGATCTCTCCGAATGCTACAATGAGCCTCCGGCAACGTTCAACGCCGGCG
GCGAGACAAGTTTCTATAGATGAAGAACCATATAACATCATTCCGATTCATAATCTTCTAGCTGACCATC
CTTCTCTACGTTTCCCTGAGGTACGCGCTGCGGCGGCGGCTTTACGCTCTGTAGGTGACCTAAGGAGACC
TCCATTTGCACCGTGGAAACCTCACTATGACCTGCTTGACTGGCTGGCGCTGTTCTTCGGGTTTCAGGAT
TCTAGTGTTCGTAACCAACGGGAACATATCGTGCTTCATCTTGCTAATGCTCAGATGCGTTTATCTCCGC
CGCCGGACAATATTGACTCTCTTGACCCTGCTGTTCTCCGTCGGTTCCGACGTCAGCTTCTGAAGAATTA
CTCGTCGTGGTGCTCTTTTCTCGGTCTCAAATCTAATGTTTGGCTTTCCGACCGGCATAACTCATCTGAC
CACCGCCGTGAGTTGCTTTATGTCTCGCTTTACCTTCTTATATGGGGTGAGTCAGCGAATCTACGTTTTG
TTCCTGAATGTTTATGCTTTATTTTTCATAATATGGCTATGGAATTGAATAAGATTTTGGAGGATTACAT
TGATGAGAATACTGGTAGGCCATTTTTTGCCATCGATATCTGGTGAAAATGCTTTTCTGAATCGGATCGTA
ACGCCAATTTACCAAACAATCCGAGCTGAGGCTGATAATAGTCGGAATGGTACTGCCCCACACTCTGCGT
GGCGGAATTACGATGACATCAATGAGTATTTCTGGACTAAAAGGTGTTTTGATAAGTTGAAGTGGCCTAT
TGATATTGGGAGTACATTTTTTGTGACCACTAACAAGGGAAAGAAGGTTGGAAAGACAGGGTTTGTGGAG
CAGAGATCATTTTTGAATTTGTATAGGAGTTTTGATAAGCTATGGATCATGCTGGCGTTGTTTTTGCAGG
CTGCAATTATTGTAGCTTGGGAAGGGAAGCCTTATCCGTGGCAGGCTTTGGAGAGTAGGGAGGTTCAGGT
GAGGGTGTTAACTATCTTCTTCACCTGGAGCAGTATGAGATTTCTGCAGTCGTTACTTGATGCAGGAATG
CAATATCGTATCATCTCTAGGGAGACCCCGTGGCATGGGGTGAGAATGGTGTTGAAGAGTGTGGTTGCAG
CTGCGTGGATTGTGGTCTTTGGTGCATTCTATGGGAGGATTTGGATCCAGAGGAATAGGGATGGGAAATG
GAGCAGTGCTGCTAACAGGAGGGTAGTGAATTTTCTTGAGGTTGCTCTTGTTTTCATTGCTCCAGAACTG
TTAGCCCTGGCACTCTTTGTTCTGCCATGGGTCAGGAATTTTCTCGAGAACACGAACTGGAGGATATTTT
ACCTGTTGTCCTGGTGGTTCCAGAGTCGAACGTTTGTGGGTCGTGGACTCAGGGAAGGCCTTGTTGATAA
CATTAAGTATTCCCTCTTTTGGGTAGTAGTGCTCGCGACCAAGTTTTCCTTCAGTTACTTCCTACAGATC
AAACCTATGATCGTTCCAACAAGAGCACTGTTGCGCCTCAGGGATGTGAAGTACGAATGGCATGAATTCT
TTAACCATAGCAACAGGTTCTCAGTAGGATTGCTTTGGCTTCCTGTTGTACTGATTTATCTCATGGATAT
TCAGATATGGTACTCAATCTACTCTTCTTTTGTTGGGGCAGCGGTTGGATTATTTGATCACTTGGGAGAG
ATTCGAAACATGCCGCAGTTAAGGTTGAGATTTCAATTTTTTGCAAGTGCAATGCAGTTTAATCTGATGC
CAGAAGAGCAGTTGTTGAATGCTCAAGGAACACTAAAAAGCAAGTTCAAGGACGCCATCCTCCGTTTGAA
ACTCAGATATGGGTTTGGTCGACCATTCAAAAAGCTTGAATCAAACCAGGTAGAGGCGAACAAATTTGCC
TTGATTTGGAATGAGATAATTACAACTTTCAGAGAAGAAGATATTCTGAATGACCGTGAGGTTGAGTTGT
TGGAGCTGCCCCAGAACACATGGAATGTTAGAGTGATTCGTTGGCCATGTTTGCTCCTCTGCAACGAGGT
GCTGCTTGGTCTCAGCCAGGCGAAGGAGCTGGTGGATGCTCCTGATAAGTGGCTCTGGCATAAGATCAGC
AAGTATGAGTACAGACGATGTGCTGTTATTGAGGCTTATGACAGTACAAGGCATTTGCTGCTGGAAATTG
TGAAATTGAACAGCGAGGAGCATTCCATCATAACAACCTTTTTTCAGCAGATTGATCAGTGGATTCAGCT
GGAGAAATTCACAAAATACTATAATCTGACTGCTCTGCCCCAGATCCGTGGAAAGTTGATTGCTCTTCTG
GATCTATTACTTAAGCCAAAAAAGGATGTTGACAAGATTGTGAATGTTCTCCAGGCCTTATATGAGGTTG
CCACTCGGGATTTTCTGAAAGAGAAGATGACTGGAGATCAGCTGAGAGAGGAAGGTCTGGCTCTTCAGGC
ATCTGCAACTAGATTGCTTTTTGAGAATGTAGTTTCATTGCCTGATCCAGAGAATGAGACATTTTATCGG
CAAGCTCGCCGCTTGAACACTATTCTTACATCTCGGGACTCTATGAGTAATATCCCAAGAAATCTTGAGG
CGAGACGTCGACTTGCCTTCTTTAGCAATTCTCTATTTATGAATATGCCACATGCGCCCCAAGTTGAGAA
GATGATGGCTTTCAGTGTTTTGACACCTTACTACAATGAAGATGTACTGTACAACAAGGAACAACTCAGA
ACTGAGAATGAAGATGGGATTTCTACATTATATTACTTGCAGACTATTTATGCTGATGAGTGGGAAAATT
TCTTGCAGCGAATGCGTAGAGAAGGAATGGTTGATGAGAAGAAAGAGTTATGGACTACAAAGCTAAGGGA
TCTTCGTCTTTGGGCATCATACAGAGGGCAGACTCTTACTCGCACGGTTAGGGGGATGATGTACTACTAT
CGAGCTCTCAAAATGCTGGCCTTTCTGGATTCTGCTTGTGAGATGGATATCAGAGAAGGATCAGTGGAAC
TTGGTTCTATGAGGCATGATGATAGCATTGGTGGTTTAAGTTCAGAAAGATCTCAGTCTTCGAGGAGGTT
GAGTAGAGCTGACAGTTCAGTGAGTATGTTGTTTAAAGGCCACGAGTATGGGACTGCTTTAATGAAATTC
ACATATGTGGTAGCTTGTCAGATATATGGGGCTCAGAAGGCCAAAAAAGATCCACATGCAGAGGAAATTT
TGTATCTGATGAAAAATAATGAAGCTCTTCGTGTAGCTTATGTTGATGAGGTTCCCACAGGAAGGGATGA
GAAGGATTATTATTCTGTGCTTGTGAAGTATGATCAAAAACTTGAAAGGGAAGTTGAGATCTATCGAGTT

AAGTTGCCTGGTCCTTTGAAGCTTGGGGAGGGGAAACCAGAAAATCAAAATCATGCCTTTATCTTTACCC
GTGGTGATGCAGTTCAGACTATTGACATGAACCAAGATAATTACTTTGAGGAGGCACTGAAAATGAGGAA
CTTGTTGGAAGAATTCAAACTCTACTATGGTATTCGCAAACCTACGATTCTTGGAGTTCGAGAACATATA
TTTACTGGTTCCGTGTCATCCCTTGCTTGGTTCATGTCAGCTCAGGAAATGAGTTTTGTAACCCTAGGAC
AGCGTGTATTAGCCAACCCCCTGAAAATCCGAATGCATTATGGACATCCAGATGTATTTGACAGGTTTTG
GTTTCTAACTAGGGGAGGAATAAGCAAGGCATCTAAAGTGATCAACATCAGCGAGGACATTTTTGCTGGC
TTCAACTGTACATTACGAGGTGGCAATGTCACTCACCATGAGTATATACAAGTTGGCAAAGGAAGGGATG
TTGGGTTGAATCAGATATCTATGTTTGAAGCCAAGGTTGCCAGTGGCAATGGAGAACAAGTTCTTAGCAG
AGATGTCTATAGGTTGGGTCATAGGCTGGATTTCTTCAGAATGCTTTCTTTCTTTTATACAACTGTAGGA
TTCTTCTTCAATACAATGATGATTGTCCTCACTGTATATGCATTCTTATGGGGACGACTTTACCTGGCAC
TTAGTGGGGTTGAGGGCTCTGTTGCTGCAGATACCACCGACAACAACAGAGCACTTGGTGCCATACTGAA
CCAGCAATTTATCATCCAGCTGGGCCTTTTCACCGCATTACCAATGATTGTGGAGAACTCTCTCGAGCAT
GGTTTTCTTACATCTATCTGGGAATTTCTTACAATGATGCTCCAACTTTCATCTGTATTTTACACATTCT
CAATGGGAACTCGTGCTCATTACTTTGGTCGTACCATTCTCCATGGTGGTGCAAAATACCGGGCAACTGG
GAGAGGTTTTGTCGTGCAGCACAAGTGTTTTGCTGAGAATTATCGGTTATATGCTCGTAGCCATTTTGTC
AAGGCAATTGAACTTGGTTTGATACTTACAGTGTATGCTGCATACAGCCCTGTTGCTAAAGGAACTTTTA
CATATATAGCACTGACTATATCAAGTTGGTTCCTGGTGGTGTCATGGATCTTGGGGCCCTTTGTGTTTAA
TCCTTCTGGGTTTGATTGGCTAAAGACAGTGTATGATTTTGATGACTTCATGAACTGGATTTGGTACCGT
GGTAGTGTTTTTGCGAAGTCAGACCAGAGCTGGGAGAAATGGTGGGAGGAGGAACAGGATCATTTAAGAA
CGACAGGTCTGTGGGGAAAGATACTGGAAATTATCCTAGACCTCCGCTTCTTCTTTTTCCAGTATGGCAT
TGTATATCATCTGGGTATTGCTGCTGGAAGCAAAAGCATTGCTGTTTACTTGCTTTCATGGATTTATGTG
GTGGTGGCTCTTGGCTTTTTTAATATTACAGCTTATGCTCGGGAAAAATATGCTGCACGGGAGCACATAT
ACTTTCGCCTTGTGCAGCTCCTTGCTGTACTCTTTTTCATAGTTGTAATTGTTGCTTTACTGCAGTTCAC
AGCATTTAAATTTGGTGATCTCTTTGTCAGCCTGTTGGCTTTTGTTCCTACTGGTTGGGGCTTCATTTCA
ATCGCCCAAGTGTTACGTCCCTTTTTGCAGAAGAGTATGATATGGGGAACTGTTGTGTCTGTGGCGCGAC
TATATGAGATAATGTTTGGGATTATTGTCATGGTACCTGTTGCAGTACTGTCTTGGTTGCCTGGTTTCCA
ACCAATGCAGACAAGGATCCTATTCAATGAAGCATTTAGTAGAGGTCTGCGGATATTCCAGATTGTGACA
GGAAAAAAGCCTAAGAGTGACGTGTGATTTGAGGTAATCCTTTTCAACATTTAGGTATACAGAAATGTTG
TATATAACTTTTATATTCTGCTGCAATGCATGTCTTTTGTGTGTTTCACATGTAGATGTTGTAGTTGTAA
GATCACCTCTTTTGTTGATCATTCTTAGAGAACCTCACACATTTGCTATTAAAAGTAGATTGTTTCTACT
GCTATGAACAGTTATTTGATTAATAATTCTGTCCCTTGTAATTTCTCTTCTTGTTGTCTGACTACAGCTT
GCTTGCCCAGTTGTGGCATTTGATTAGTGCCGTTGAGTTCTTTCGTTTATTTTCCGTCTTCCTTTAATTT
TTTATTTTTCTTTTGGGGGTGGGGGGTGAGGGGGTGGGGTAGGGTAGTAAATTGTTAAGAATTGAATATT
TTACATGTATTAATTTGCAATGAAGCTAGTATATTGCCCACCTGCATCCGTTTGGTTTTACTTTCATCTC
GTTTTAAGCCAATTATAAAGGATGAAAAGAATCAAAACTTTTACAAGTCATGGTTAACTTGATTCTGCTA
GGAACTATACTCTCTTCTTGCACAAGACTCTCCCATTAATCACTGGATTCACATCTTAAAAATGATGATA
GTTTCTATTCGTAGTCTACATATTCTATTATGGACTACTTCATAAGAAAAACATATCCTAATGTGGACTA
ACATATGTAAAACTCCCAAGCTTTAGTTTGAGATCTATTTCTTGACTTTTGCTTTCTGATTTCCCTGAAA
AGTTTCAATCAGGTTGATATAATATATAAAGTAGGATTACTCATGTTTATGTACTGGAGCTGAGGTTCTT
AACGTCTGGTCTTTTCTTTGGATTGTTGAAGATTTTGGAAGATATGTAATAATATTCATAAGTTGCTATC
TACGTGACAGTCTTCCTAAATTGGCCAATACACCATTGTTTTCGACTACCATTGAATTATATTCCTCTTC
AACAAAAACTCTTCTGTCTGCATAGCTCAACTTATGACTAGTCACTACTCTCTAGTGTATGTAATTATAT
ATGAAGATACAATGTTTCCCACTTTATAGCAAGAATATTAACATCTTGGACTTGAAGGGATATCTTGTTT
TAGTAGTATGCTTCTATCAAATTAACATACCTCCTGTGGGCGTTGATCTTCTAATGCTACTTTCTTAAAA
CTTCAGAAGCCAATTTGCAGGTCCAATTTCCATGGAAACTAGTGATCTTCTAGCTACCTATAGCTCCTAA
AATAATTTTGTTCGATTAGCTAAAATGTCATCCCTTTTGTTACTTATCCTGGAATTTGTGTTTTTGATCA
TTCCTCGATTTCTGGAATTATTTATTGTATTACAACAATTCTCTTCTTTCCTCACTTTCAGGATTTACAG
GAACATTAAGTATGAGTGCTCCATGTAGTCAATGTCCAATATACATGTGGAAATTAAGGCATGGTACGAA
ACCTTCTCAACTCTTTGATTTGTCAGTTATTCAACTATTTGTTCATTTTACCAACCATCTTGTTTGGAGC
TTAATCATGCATACTGCTATTTTTGGTCTTAAATTAAATATCATGAAGTTTTAAAAAGTTTAATTAAGTT
AACTTTTTAGAAGTTACATATGAAAAATAACAGTAGAAGGTACTTTAAAGAGATAACATTAGATTTTCAC
TTTCGTATGAGCCTTCTTAACTATCCTTCATAATTCATATGGATTTTTTATTTTTTATTCTTTCAAATTT
GAGGGTAGTGAAAGGGGAAATGGGGGAGGGGTTTACAATGTGGGGAATCTAACCCTCATTAATAAGGTGG
AAGTTTAGATAGCCAACCAACTGAGCTACTAAGATTTCTCCTTTCATCATAGGGTTTAATCTGATTTTTG

GTAAGCTTGATTATGTGTAAATCTGTTTGCTAGCACTAATTATGAAGATGACTCTTAAGTAGAGAGATAA
TAGGATAACATTTAGCTGCATGTTAAATTTCGAAATATATTATTGCAAACCTTTTACTCGTTGTTCACGA
TCAATTTTGTTGAGAACTGATATGGTTACTAAAATATCCAGAATAATTTGCTACATTCAAGATATCTCTG
GAGTGTTCCCACTTCTCTTTTATTTTTTGATGACTGTAGTGTCCGGACCAACTTTCGCACACCTTGGCTA
ATTTCTTATCTCTGGAGTGTAGTAGTCAGAAGCCCAAAATTTTATTGGAAAAGCACAAGGGAAGTATATT
CGAGTAAATAGTACACCGACCAACCAACTCTTTGCTTGTTTATTCTAGAATCTTACAGTAATAGTGGGCT
GAACTAGTGTCATTGAGAGAGAATCAATACTATTAGTTGGATTTATGCTAATATCTGAACTGCAAAAGGT
ATAGTTCATAATGTAGCCATATAAGGAGACTGATGAAGCTTTAGCGTGAACCAATGTGGGATTTTCAACA
TCCCTCCTCTGTTAGAACTGGAAACTTGGAGCACTGATGACAGTATTGAGGGGGGTGGGCCTACATTGGA
TAACTCAGTATTAGGGATAGCAGTGGTTCTGACTCCATGTTGATAAAACTAGAACTTGTACCTAACTCAA
CCCCAAAGCTAGCTCATAAGTTAAGTATTGTCCAGTGTAATGTGCTCGAACGAACAAGAATCAAATGGGA
AATTTATTAACCATCTCACCTCACTCACCAGAATGCCGACTTGATAATTTGTAACAACGCCGAAGATAAG
GGAAAGATTGGGAGAATAAGAGTAAGTAGAAAGAAAGAGAGGAGGAAGAGTCAGAGGTGAATAAGAAAGA
TGGTGAGAATTTTTTTTCAGATTTTTTGTTCTTCTTTCACACTTGTGGGACTCTCCATAATTACTACACT
TGAACCAAGGGCCCTTCAAAAACAACCTCTCTAGCTCCATGAGGTAGGGGTGTAGGTCTGCGTACACTTA
AACTTTCCTTCGACCCTACTTGTGGAATTTCACTTTTACTGTTGTGACTGGGTAGTTGTAGTAAATCTCT
TTTTCAATTTAAGCTCACTAAGCTCCCCTACTTTGATTTTCTTAACTAGCTATTTCCCCTATACATGACA
ATCCAAATCATATTAGTATATCAAAACCCATAACCTTTGACCAAACGCGAGACTCTAAAGACACGTGCTG
AGTTGTTATTTGCTTCTTTGTTTATATTAGCCTCTTGTTTTTGTTGAAAGATGTTGTTTTGCTTCTGCAG
GGATGAACATTAATGTGAAGTAGTAGATACAGTACAGAGTTTGAAGAGGCTAGGAGGTTAAATATTGTAG
GAGCTTGTATTGTGAATTATACTGTGCACTTGGATTACCATTAGTTTGGTTGACGAGTTTTCATTCATCT
TTTGTAATTGTTGTTGTATTCATATGTTTTATTGTCATACTAGTGCACAGATGAGGAGCATTATCTTATA
TTATTGTAGAACCCCCACAGTTCATTTTTGTACGGTTTTTGTAGTTTCTACTGGGGAGCCACGTTCAAGT
ATAGTAGCAATTCCTTCTGGTATTAAATGATTTTATCAAATGTCTTTGAATTATTA
