# Supplementary material for: Identification of Susceptibility Genes in Castanea sativa and Their Transcription Dynamics following Pathogen Infection
Source: Plants (Basel). 2021 May 2;10(5):913. doi: 10.3390/plants10050913 (PMC8147476; doi:10.3390/plants10050913)
Supplement: Supplementary file 1 [file plants-10-00913-s001.zip › S3 File.pdf]

#S-genes coding sequences used for trees construction

DMR6;

>XP_030943578.1_1-*Quercus_lobata*
ATGGCTGCTATTCCCCAACCCTCTCAGCCATCTCCTACTCAAATTTCCAAGATGACGACATATATTAAGA
AAGTAGCTGAATCACCTGGGCTCACCTCTATTCCTTCCAGTTACGCTTTTACCCCTAGTCCCAATGTTCA
AGCAGTTTCTGAAGACCCAGAAGACTCAATTCCAATCATTGACTTCTCTCTACTCACTTCTGGTACTCCT
GATCAACGGTCCCAACTTATCCAAGAGCTTGGCCAAGCCTGCAAGTACTGGGGCTTCTTCAAGTTGATCA
ACCATGGTGTTCCGGAGAGCTTAATGGAGGCAATGATAGAAGGCATTAGAGGATTTTTCAATCTGACAGA
CGAAGAAAAGAGAGAGTTCGAAGGGAAAAACCTTTTCGACCCAATCAGCTGCGGAACCAGCGTCAATACA
TCAATGGAAAAGGTTTTTTACTGGAGGGATTATCTCAAGGTCTTTGTACATCCTGAGTTTCACTTCCCCA
ACAAACCTGCAGGATTCAGTGAGCTTTCATTAGAGTATTGCGAAAGAACACGAGGACTAGCAAGGGAATT
ACTTAAAGCAATATCAGAGAGTTTAGGGTTGGAACCCTTCTACATTGAGAAGGTCATGAATTTAGATAAA
GGTTTACAAATGTTATATGCAAACTTTTACCCACCCTGTCCACAGCCAGAACTTGCAATGGGCTTGCCAC
CACATTCGGACCATGGCTTCTTGTCTCTTGTCACACACAATGGAATTGGTGGCTTCCAACTACAACACAA
TGGGATGTGGGTCAATGTTAATGCCATTCCCAACTCCTTTCTAGTTAACATTGCTGATCAACTTGAGCTG
TGGGCACAAGCGCCCCCCATCGCGGATGCTGCAGATGTGGTTTCCTCATAA
>XP_030943577.1_2-*Quercus_lobata*
ATGGCTGCTATTCCCCAACCCTCTCAGCCATCTCCTACTCAAATTTCCAAGATGACGACATATATTAAGA
AAGTAGCTGAATCACCTGGGCTCACCTCTATTCCTTCCAGTTACGCTTTTACCCCTAGTCCCAATGTTCA
AGCAGTTTCTGAAGACCCAGAAGACTCAATTCCAATCATTGACTTCTCTCTACTCACTTCTGGTACTCCT
GATCAACGGTCCCAACTTATCCAAGAGCTTGGCCAAGCCTGCAAGTACTGGGGCTTCTTCAAGTTGATCA
ACCATGGTGTTCCGGAGAGCTTAATGGAGGCAATGATAGAAGGCATTAGAGGATTTTTCAATCTGACAGA
CGAAGAAAAGAGAGAGTTCGAAGGGAAAAACCTTTTCGACCCAATCAGCTGCGGAACCAGCGTCAATACA
TCAATGGAAAAGGTTTTTTACTGGAGGGATTATCTCAAGGTCTTTGTACATCCTGAGTTTCACTTCCCCA
ACAAACCTGCAGGATTCAGTGAGCTTTCATTAGAGTATTGCGAAAGAACACGAGGACTAGCAAGGGAATT
ACTTAAAGCAATATCAGAGAGTTTAGGGTTGGAACCCTTCTACATTGAGAAGGTCATGAATTTAGATAAA
GGTTTACAAATGTTATATGCAAACTTTTACCCACCCTGTCCACAGCCAGAACTTGCAATGGGCTTGCCAC
CACATTCGGACCATGGCTTCTTGTCTCTTGTCACACACAATGGAATTGGTGGCTTCCAACTACAACACAA
TGGGATGTGGGTCAATGTTAATGCCATTCCCAACTCCTTTCTAGTTAACATTGCTGATCAACTTGAGATT
TTAAGTAATGGGAAATACAAGAGTATTCTACATCGAGCAGTAGTAAACAAAAAAGTCACAAGGATATCAC
TTGTCATGACCAATGGACCACCACTCAATAAGATTGTCAACCCAGCACTAGAGTTAGTAAACAATGGAAT
TGATCAACCTGCATACCATGGGATAACGTATAAAGAATACTTGCAATGTCGACAAAGCAACAGTCTTGAT
GGGAAAGGCAACTTGGATCACATACGCATCAAAATTGTGTGA
>XP_023904502.1_1-*Quercus_suber*
ATGGGAGAGGTAGACTCAGCTTTCATACAGCCCATTGACCACAGGCCAAAGCTCAAGCCCATCGAGGTAA
CTGATGAAATCCCAGTTCTTGACCTCTCCAGTGTGCTCAACTCTGAAAACAGTGACCAACAACTCATCTC
TGAGATTGGCAGTGCATGCCAGACCTGGGGGTTCTTCCAGGTGATCAACCATGGAGTCCCAGCTGAGCTT
CTCAGAAAAGTAGAGGCTGTGGCTAAGGAATTCTTTGCTGGGTCTTTTGAGGAGAAGAGGAAGGTGCAAA
GGGACGCGTTGTATCCCATGGGGTACCATGATAGTGAGCACACTAAAAACGTTAGGGATTGGAAGGAGGT
GTTTGATTTTTTGGCCCATGATCCCACAGAGGTACCAGCCTCGCATGAACCTGATGATGAGGGAATTAGA
GTTTTGACCAATCAGTGGCCTCAGTACCCGCCCGAGTTCAGGGAAATATGTGAGGAGTATGGCCGAGAAG
TAGAAAAGCTGGGTTTCAAGTTATTGGAACTTATTTCCCTGAGTTTAGGCTTACCAGCTGATCGGCTAAG
GGTCTTCTTCAAGGACCAAACCAGCTTTCTCCGGTTCAACTACTATCCTCCATGCCCCTCCCCCGACCTA
GCTCTTGGTGTTGGCCGCCATAAAGATGGTGGTGCCTTAAGTGTCCTTGCACAAGATGATGTTGGGGGAC
TTGAAGTGAGGCGGAAATCAGATGGAGAGTGGATTCCGGTTAAGCCTATCCCAGATGCCTATATTATTAA
TGTTGGTGATGCTGTTCAGGTTTGGAGCAATGACACGTATGAGAGTGTGGAGCACAGGGTGATAGTGAAT
TCAGAGAGAGAGAGGTACTCATATCCATTCTTCTTACTACCTCCACTCTCCACCATGGTTGAGCCTTTGG
AGGAGCTGACGAATAAGAAAAACCCTGCTAAATATAGGGCATACAACTGGGGAGAGTTTATAAGTCACAG
AAGTCAGAGTAATTTCCAAAAACAAAAAGTTGAAAACCTGCAAATTAGTGATTTCAAGATATCAGAGTAG
>XP_023904508.1_2-*Quercus_suber*
ATGGGAGAGGTAGACTCAGCTTTCATACAGCCCATTGACCACAGGCCAAAGCTCAAGCCCATCGAGGTAA

CTGATGAAATCCCAGTTCTTGACCTCTCCAGTGTGCTCAACTCTGAAAACAGTGACCAACAACTCATCTC
TGAGATTGGCAGTGCATGCCAGACCTGGGGGTTCTTCCAGGTGATCAACCATGGAGTCCCAGCTGAGCTT
CTCAGAAAAGTAGAGGCTGTGGCTAAGGAATTCTTTGCTGGGTCTTTTGAGGAGAAGAGGAAGGTGCAAA
GGGACGCGTTGTATCCCATGGGGTACCATGATAGTGAGCACACTAAAAACGTTAGGGATTGGAAGGAGGT
GTTTGATTTTTTGGCCCATGATCCCACAGAGGTACCAGCCTCGCATGAACCTGATGATGAGGGAATTAGA
GTTTTGACCAATCAGTGGCCTCAGTACCCGCCCGAGTTCAGGGAAATATGTGAGGAGTATGGCCGAGAAG
TAGAAAAGCTGGGTTTCAAGTTATTGGAACTTATTTCCCTGAGTTTAGGCTTACCAGCTGATCGGCTAAG
GGTCTTCTTCAAGGACCAAACCAGCTTTCTCCGGTTCAACTACTATCCTCCATGCCCCTCCCCCGACCTA
GCTCTTGGTGTTGGCCGCCATAAAGATGGTGGTGCCTTAAGTGTCCTTGCACAAGATGATGTTGGGGGAC
TTGAAGTGAGGCGGAAATCAGATGGAGAGTGGATTCCGGTTAAGCCTATCCCAGATGCCTATATTATTAA
TGTTGGTGATGCTGTTCAGGTTTGGAGCAATGACAAGTATGAGAGTGTGGAGCACAGGGTGGTGGTGAAT
TCTACCAAGGCAAGGCTGTCTATTCCGTTCTTCTTCTTCCCAGCCCATTATACCATGGTGAAGCCTCTAG
AGGAGCTAGTCAATGAGCAGGACCCTGCCAAATACAGAGAATTCAACTGGGGAAAATTTGTTGCCAATAG
AAACCGAAGTGATTTCAAGAAGCAAGATGTGGAAAACATCCAAATTTATCATTTTAGAATATAA
>XP_023904530.1_3-*Quercus_suber*
ATGGGAGAGGTTGATCCAGCTTTCATCCAAGAACCTGAAAACAGGCCTAGAATCTCCTACATCGAAGGTG
AAGGCATCCCAGTAATCGATCTTTCTCCATTGCACTCCCCCAACGATGTTTCTGCCTTTGAAAGCCTTGT
TAAAGAGATAGGCAATGCATGCAAAGACTGGGGGTTTTTCCAGGTGATCAACCATGGGGTGCCGTTGGAG
AAGCGCCAGAAGATTGAGAAAACATCTAAGAGGTTCTTTGCGCAGAGTTTGGAGGAGAAGAGGAAGGTGA
AAAAAACTGAGAAGCTGGTGTTGGGTTACTATGACTCTGAGTATACCAAGAATGTTAGGGACTGGAAGGA
GGTGTTTGATTTTATTATAGAGGAACCCACTTTATTCCCGAATTCACATGAGTCTGCTAATGACAAAGAA
CTCGCAGGATGGACTAATCTGTGGCCTGAGTACCCACCTGAGTTTAGGGAAACATGCCAAGAATATATTC
AAGAGATGGAGAAACTAGCATTCAAGTTGATGGAACTTGTGGCTCTGAGCCTAGGCTTACCAGCAGATAG
GTTCCATGGCTTCTTCAAAGATCAAGTCAGCTACGTTCGACTCAATTACTATCCGATATGCCCTTTTCCT
CACTTAGCGCTTGGTCTAGGTCGACACAAGGATAGTGGTGCCGTGACCATCCTAGGTGAAGATGGTGTTC
CGGGATTAGAAGTGAAACGAAGAAAAAATGGAGAGTGGGTTCGGGTCAAACACATCCCAGATGCTTATAT
CATCAACATTGGTGACAATATTAAGGTTTGGAGCAATGATGAGTATGAGAGTGTAGAGCACAGAGTAATG
GTGAATTCAGAAAAAGAAAGGTACTCGTATCCATTTTTCTTACTACCGGCACTTTCCACTGTGGTTGAAC
CCTTGGAGGAGTTGACGAATGAGCAAAACCCTGCTAAATATAGGGCATACAATTGGGGAGAGTTTATAAG
TAACAGAAATCAGAGTAATTATCAAAAACAAAAAGTTGAAAACCTGCAAATTAGTGATTTCAAGATACCA
GAGTAA
>XP_023904517.1_4-*Quercus_suber*
ATGGGAGAGGTTGATCCAGCTTTCATCCAAGAACCTGAACACAGGCCAAAAATCTCCTACATCGAAGGCG
AAGGTGTCCCACTAATCGATCTTTCTCCAATACTCTCTTCCAACAATGTTTCTGCCGTTGAATTTGAAGG
CCTTGTTAAAGAGGTAGGCAATGCATGCAAGGAGTGGGGGTTCTTCCAGGTGATCAATCATGGGGTGCCT
TTGGAGAAGCGAGAGAAGATTGATGGTGCCTCGAGGAAATTCTTTGCTCAGAGTTTGGAGGAGAAGAGGA
AGGTGAGGAGGAATGAGAAGAAGGTTACTGGTTACTATGACACAGAACATACCAAGAATGTTAGGGACTG
GAAAGAAGTGTTTGATTTTGTTGTAGTGGGTCCCACTATAGTCCCTGCCTCGCATGAGTCTGAAGACAAG
GAAGTCATTGAATGGAATAATCAGTGGCCTGAGTACCCTCCAGAATTAAGGGAGACATGCCAAGACTACG
GTCAAGAGATGGTAAAACTAGCTTACAAGTTATTGGAACTTATTGCACTGTCCCTAGACTTGCCAGCAGA
CAGATTCCATGGCTTCTTCAAAGATCAGATCAGCTTCATTCGACTAAATCGCTATCCACCTTGCCCTACT
CCTGAGTTAGCGCTCGGTGTCGGTCGGCACAAGGATCCTGGTGCCTTAACCATCCTCGCTCAAGATGATG
TTGGAGGATTGGAAGTGAAGCAGAAAATTGATGGAGAGTGGGTTCGGGTCAAACCCACCCCAGATGCTTA
TATCATCAATGTTGGTGACATTATTCAGGTTTGGAGCAATGACATATATGAGAGTGTAGAGCACAGGGTG
ATAGTGAATTTAGAGAGAGAAAGGTACTCATATCCATTCTTCTTACAACCTCCACACTCCACCGTGGTTG
AGCCTTTGGAAGAGCTGACGAATGAGAAAAACCCTGCTAAATATAGGGCATACAACTGGGGAGAGTTTGT
GAGTAACAGAAGTCAGAGTAATTTCCAAAAAAAAAAAGTTGAAAATCTGCAAATTAGTGATTTCAAGATA
TCAGAGTAG
>XP_023904495.1_5-*Quercus_suber*
ATGGGAGAGGTTGATCCAGCTTTCATCCAAGAACCTGAACACAGGCCAAAAGTCTCCTACATCGAAGGCA
AAGGTGTCCCACTAATCGATCTTTCTCCAATACTCTCCTCCAACAATGTTTCTGCCATTGAATTTGAAGG
CCTTGTTAAAGAGGTAGGCGATGCATGCAAATGTTGGGGGTTCTTCCAGGTGATCAATCATGGGGTGCCT
TTGGAGAAGCGAGAGAAGATTGATGATGCCTCGAGGAAATTCTTTGCTCAGAGTTTGGAGGAGAAGAGGA
AGGTGAGGAGGAATGAGAAGAAGGTGACTGGTTACTATGACACAGAACATACCAAGAATGTTAGGGACTG

GAAAGAAGTGTTTGATTTTGCTGTAGAGGAACCCACTATAGTCCCTGCCTCGCATAAGCCTGATGACAAG
GAAGTTATTGAATGGAATAATCAGTGGCCTGAGTACCCTCCAGAATTAAGGGAGACATGCCAAGACTATG
GTCAAGAGATGGTAAAACTAGCTTACAAGTTATTGGAACTTATTGCACTGTCCCTAGACTTGCCAGCAGA
TAGGTTCCATGGCTTCTTCAAAGATCAGACCAGTTTCATCCGACTAAATCACTATCCACCTTGCCCAACC
CCTGAGTTAGCGCTCGGTGTCGGTCGGCACAAGGATGCTGGTGCCTTAACCATCCTTGCTCAAGATGATG
TTGGAGGATTGGAAGTGAAGCAGAAAACAGATGGAGAGTGGGTTCGGGTCAAACCCACCCCAGACGCTTA
TATCATCAATGTTGGTGACATTATTCAGGTTTGGAGCAATGACACGTATGAGAGTGTGGAGCACAGGGTG
ATAGTGAATTCAGAGAGAGAGAGGTACTCATATCCATTCTTCTTACTACCTCCACTCTCCACCATGGTTG
AGCCTTTGGAGGAGCTGACGAATAAGAAAAACCCTGCTAAATATAGGGCATACAACTGGGGAGAGTTTAT
AAGTCACAGAAGTCAGAGTAATTTCCAAAAACAAAAAGTTGAAAACCTGCAAATTAGTGATTTCAAGATA
TCAGAGTAG
>XP_022860951.1_1-*Olea_europea_var._sylvestris*
ATGGGAGAAGTGGATCCTGCTTTCATCCAAGCCCTTGAACATCAGCCTAAACTTGACATAACTGAAGCAG
AAGGCATCCCGTTAATCGATCTTTCCCCGCTGAATTCCTCTAATACAGATGCTGACTTGTCAAATCTAGT
AGCTGAGATAGGTGATGCTTGCAAGAACTGGGGGTTTTTCCAGGTGATCAACCATGGCGTGCCGTCGAAG
TGTCGTGAAAAGATTGAATCAGTGGCGAGGAAATTCTTTGCTTTATCGAAAGAGGAGAAGAAGAAAGTCA
GTAAAGATCAAGACAACCCTTTGGGATATTACGATACTGAGCTTACTAAAAATGTCAGGGACTGGAGAGA
AGTGTTCGATTTTACTTTAATGAATCAGGTCATTATACCGGCTTCTCCAGAGCCTGAGGATAAGGAACTT
AAACAGTTGATTAATCAGTGGCCTGAATACCCCCCTGAATTAAGGGACGCATGTGAAGAGTATGCTGCAG
AGATGGAAAAACAGGCTTACAAGTTACTCGAACTCATTGCCCTGAGCTTAGGCTTGCCAAAAACTCGATT
GAATGGCTTCTTTAAGGATAACAATAGCTATGCCAGGCTCAATCACTATCAGCCATGCCACGTTCCTCAT
TTAGTGCTCGGTCTTGGTCGACACAAAGATGGTGGAGCCGTGACCATCCTTGCTCAAGATGATGTTGGAG
GACTTGAAGTGAAGAGGAAAACAGATGGAGAGTGGGTTCTTGTCAAACCTACCCCTAATGCTTTTATCGT
CAATGTTGGTGACGTTATCCAGGTTTGGAGTAATGATAAGTACGAAAGTGTAGAGCACCGAGTGATGGTC
AATTCTGAAAGGGAAAGATTCTCATTTCCATTCTTCTTCTGTCCTGCACATTCTACTTGGGTGGAACCCG
TGGAGGAGCTGATAAACAAAGATAATCCTGCCAAATACAAGGCTTATAACTGGGGAAAGTTTTATGCGAA
TAGAAGGCGTAGTAATATCAAGAAACTTGGTGTCGAAAACATTCAAATTTATCATTTCAGGATTTAG
>XP_022860956.1_2-*Olea_europea_var._sylvestris*
ATGGGAGAAGTGGATCCTGCTTTCATCCAAGCCCTTGAACATCAGCCTAAACTTGACATAACTGAAGCAG
AAGGCATCCCGTTAATCGATCTTTCCCCGCTGAATTCCTCTAATACAGATGCTGACTTGTCAAATCTAGT
AGCTGAGATAGGTGATGCTTGCAAGAACTGGGGGTTTTTCCAGGTGATCAACCATGGCGTGCCGTCGAAG
TGTCGTGAAAAGATTGAATCAGTGGCGAGGAAATTCTTTGCTTTATCGAAAGAGGAGAAGAAGAAAGTCA
GTAAAGATCAAGACAACCCTTTGGGATATTACGATACTGAGCTTACTAAAAATGTCAGGGACTGGAGAGA
AGTGTTCGATTTTACTTTAATGAATCAGGTCATTATACCGGCTTCTCCAGAGCCTGAGGATAAGGAACTT
AAACAGTTGATTAATCAGTGGCCTGAATACCCCCCTGAATTAAGGGACGCATGTGAAGAGTATGCTGCAG
AGATGGAAAAACAGGCTTACAAGTTACTCGAACTCATTGCCCTGAGCTTAGGCTTGCCAAAAACTCGATT
GAATGGCTTCTTTAAGGATAACAATAGCTATGCCAGGCTCAATCACTATCAGCCATGCCACGTTCCTCAT
TTAGTGCTCGGTCTTGGTCGACACAAAGATGGTGGAGCCGTGACCATCCTTGCTCAAGATGATGTTGGAG
GACTTGAAGTGAAGAGGAAAACAGATGGAGAGTGGGTTCTTGTCAAACCTACCCCTAATGCTTTTATCGT
CAATGTTGGTGACGTTATCCAGGTTTGGAGTAATGATAAGTACGAAAGTGTAGAGCACCGAGTGATGGTC
AATTCTGAAAGGGAAAGATTCTCATTTCCATTCTTCTTCTGTCCTGCACATTCTACTTGGGTGGAACCCG
TGGAGGAGCTGATAAACAAAGATAATCCTGCCAAATACAAGGCTTATAACTGGGGAAAGTTTTATGCGAA
TAGAAGGCGTAGTAATATCAAGAAACTTGGTGTCGAAAACATTCAAATTTATCATTTCAGGATTTAG
>XP_022860963.1_3-*Olea_europea_var._sylvestris*
ATGGGAGAAGTGGATCCTGCTTTCATCCAAGCCCTTGAACATCAGCCTAAACTTGACATAACTGAAGCAG
AAGGCATCCCGTTAATCGATCTTTCCCCGCTGAATTCCTCTAATACAGATGCTGACTTGTCAAATCTAGT
AGCTGAGATAGGTGATGCTTGCAAGAACTGGGGGTTTTTCCAGGTGATCAACCATGGCGTGCCGTCGAAG
TGTCGTGAAAAGATTGAATCAGTGGCGAGGAAATTCTTTGCTTTATCGAAAGAGGAGAAGAAGAAAGTCA
GTAAAGATCAAGACAACCCTTTGGGATATTACGATACTGAGCTTACTAAAAATGTCAGGGACTGGAGAGA
AGTGTTCGATTTTACTTTAATGAATCAGGTCATTATACCGGCTTCTCCAGAGCCTGAGGATAAGGAACTT
AAACAGTTGATTAATCAGTGGCCTGAATACCCCCCTGAATTAAGGGACGCATGTGAAGAGTATGCTGCAG
AGATGGAAAAACAGGCTTACAAGTTACTCGAACTCATTGCCCTGAGCTTAGGCTTGCCAAAAACTCGATT
GAATGGCTTCTTTAAGGATAACAATAGCTATGCCAGGCTCAATCACTATCAGCCATGCCACGTTCCTCAT
TTAGTGCTCGGTCTTGGTCGACACAAAGATGGTGGAGCCGTGACCATCCTTGCTCAAGATGATGTTGGAG

GACTTGAAGTGAAGAGGAAAACAGATGGAGAGTGGGTTCTTGTCAAACCTACCCCTAATGCTTTTATCGT
CAATGTTGGTGACGTTATCCAGGTTTGGAGTAATGATAAGTACGAAAGTGTAGAGCACCGAGTGATGGTC
AATTCTGAAAGGGAAAGATTCTCATTTCCATTCTTCTTCTGTCCTGCACATTCTACTTGGGTGGAACCCG
TGGAGGAGCTGATAAACAAAGATAATCCTGCCAAATACAAGGCTTATAACTGGGGAAAGTTTTATGCGAA
TAGAAGGCGTAGTAATATCAAGAAACTTGGTGTCGAAAACATTCAAATTTATCATTTCAGGATTTAG
>XP_021811284.1_1-*Prunus_avium*
ATGCTTGAATTCACTGGGATATCAGATGCCTCCTCCATTCCTCTCATTGATCTCAAGGATCTCCATGGCC
ACAACCACTCTAATATTACCAAACAGATTGGCCTAGCTTGCCAAACTGATGGTTTCTTTCAGGTGAAAAA
TCATGGGGTGCCAGAGGAAATGATCAAAGACATGTTAAGTATAGCAAGTGAGTTTTTCAATTTGCCAGAA
AGTGAGAGGTTGAAGATGTACTCAGATGACCCTTCAAAGACCACAAGGCTTTCCACTAGTTTCAATGTCA
GGACTGAAAAACTTTCCAACTGGAGGGACTTTCTGAGACTCCATTGCTACCCTCTTGAAGACTATGTCCA
AGAATGGCCTAACAATCCCCCATCATTCAGGGAGCAAGTGGGTGAGTATTGCACAAGTGTGAGAGGGCTC
GTGCTGATATTACTTGGGGCCATATCAGAAAGCTTGGGCTTAGAAAAGAACTATATTGTTGAGGCATTAG
GAAAGCAAGGCCAGCATATGGCTTTGAATTACTATCCACCCTGTCCAGAACCAGAGCTTACATATGGATT
GCCTGGACATACAGATTGTAACCTAATCACCATCCTTCTCCAAGATGATGTGCCTGGATTGCAGGTCCTT
AGAAATGGCAAGTGGGTTGCTGTCAACCCCATTCCCAACACTTTTATTGTCAATATTGGTGACATGATGC
AGGTAATTGGCAATGGTAAGTACAGAAGTGTGTTGCACAGAGCTGTGGTTAACTGCAACTCAGAGAGAAT
ATCAATTCCAACCTTCTACTGTCCATCACCTGATGCAGTGATTGGGCCAGCCAAGGACCTGATCAGCCAT
GATCAACCAGCCATGTACAGGAACTTCACATATGCAGAATACTTTGAGAAATTCTGGAACAGAGGACTTG
CAACTTCGTGCTGCTTAGACTTGTTCAAACCCAATTTATGA
>XP_021811285.1_2-*Prunus_avium*
ATGCTTGAATTCACTGGGGTGAAAAATCATGGGGTGCCAGAGGAAATGATCAAAGACATGTTAAGTATAG
CAAGTGAGTTTTTCAATTTGCCAGAAAGTGAGAGGTTGAAGATGTACTCAGATGACCCTTCAAAGACCAC
AAGGCTTTCCACTAGTTTCAATGTCAGGACTGAAAAACTTTCCAACTGGAGGGACTTTCTGAGACTCCAT
TGCTACCCTCTTGAAGACTATGTCCAAGAATGGCCTAACAATCCCCCATCATTCAGGGAGCAAGTGGGTG
AGTATTGCACAAGTGTGAGAGGGCTCGTGCTGATATTACTTGGGGCCATATCAGAAAGCTTGGGCTTAGA
AAAGAACTATATTGTTGAGGCATTAGGAAAGCAAGGCCAGCATATGGCTTTGAATTACTATCCACCCTGT
CCAGAACCAGAGCTTACATATGGATTGCCTGGACATACAGATTGTAACCTAATCACCATCCTTCTCCAAG
ATGATGTGCCTGGATTGCAGGTCCTTAGAAATGGCAAGTGGGTTGCTGTCAACCCCATTCCCAACACTTT
TATTGTCAATATTGGTGACATGATGCAGGTAATTGGCAATGGTAAGTACAGAAGTGTGTTGCACAGAGCT
GTGGTTAACTGCAACTCAGAGAGAATATCAATTCCAACCTTCTACTGTCCATCACCTGATGCAGTGATTG
GGCCAGCCAAGGACCTGATCAGCCATGATCAACCAGCCATGTACAGGAACTTCACATATGCAGAATACTT
TGAGAAATTCTGGAACAGAGGACTTGCAACTTCGTGCTGCTTAGACTTGTTCAAACCCAATTTATGA
>XP_021811283.1_3-*Prunus_avium*
ATGGCCACAGCAACAAAGCTACTGCTAACAGACCTCATGTCTGTTGTGAACCATGTCCCCTCAAACTACA
TTAGGCCCATCTCTGACCGTCCAAATCTCTCTGATGTTCAGATATCAGATGCCTCCTCCATTCCTCTCAT
TGATCTCAAGGATCTCCATGGCCACAACCACTCTAATATTACCAAACAGATTGGCCTAGCTTGCCAAACT
GATGGTTTCTTTCAGGTGAAAAATCATGGGGTGCCAGAGGAAATGATCAAAGACATGTTAAGTATAGCAA
GTGAGTTTTTCAATTTGCCAGAAAGTGAGAGGTTGAAGATGTACTCAGATGACCCTTCAAAGACCACAAG
GCTTTCCACTAGTTTCAATGTCAGGACTGAAAAACTTTCCAACTGGAGGGACTTTCTGAGACTCCATTGC
TACCCTCTTGAAGACTATGTCCAAGAATGGCCTAACAATCCCCCATCATTCAGGGAGCAAGTGGGTGAGT
ATTGCACAAGTGTGAGAGGGCTCGTGCTGATATTACTTGGGGCCATATCAGAAAGCTTGGGCTTAGAAAA
GAACTATATTGTTGAGGCATTAGGAAAGCAAGGCCAGCATATGGCTTTGAATTACTATCCACCCTGTCCA
GAACCAGAGCTTACATATGGATTGCCTGGACATACAGATTGTAACCTAATCACCATCCTTCTCCAAGATG
ATGTGCCTGGATTGCAGGTCCTTAGAAATGGCAAGTGGGTTGCTGTCAACCCCATTCCCAACACTTTTAT
TGTCAATATTGGTGACATGATGCAGGTAATTGGCAATGGTAAGTACAGAAGTGTGTTGCACAGAGCTGTG
GTTAACTGCAACTCAGAGAGAATATCAATTCCAACCTTCTACTGTCCATCACCTGATGCAGTGATTGGGC
CAGCCAAGGACCTGATCAGCCATGATCAACCAGCCATGTACAGGAACTTCACATATGCAGAATACTTTGA
GAAATTCTGGAACAGAGGACTTGCAACTTCGTGCTGCTTAGACTTGTTCAAACCCAATTTATGA
>XP_018813023.1_1-*Juglans_regia*
ATGTCTCCTACGATGGCTGTAACAACTGAAACAAAGGAGGAGAATGATACGCCAGATAGCGAGTACCAAA
AAGGAATAAAGCACCTCTGGGAAAATGGCATAAACAGAGTTCCCAAGAAGTACATATTACCCCCCTGCGA
TCGACCCATTACTGAAGACGGGGTGCTAAATCATGTATCCGAGCAAAATCTTAAGCTGCCCATCATTGAT
TTCGCAGAACTAATGCAAGGTGCCAACCGGCCTCAAGTCCTCGAGTCCCTCGCTAATGCTTGCGAACAAT

ACGGTTTTTTTCAGCTGGTAAACCATGGCATTCCAAGCGATGTTATAAGCAGCATGATTGACGTTTGTAC
AAGGTTTTTCGAGCTGCCATTTGAGGAAAGATCAAAGTACATGTCTTCGGATATGCAAGCGCCGGTCCGA
TATGGAACCAGCTTTAACCAGAAGAAAGATAATGTGTTTTGTTGGAGAGACTTCTTGAAGCTAATGTGCC
ATCCCCTATCAGATGTCCTCCCACATTGGCCTTCTTCTCCTATGGACCTGAGGAAATTGGCGGCTACCTA
CGCAACAGAAACCAAATACTTGTTTCTAATGCTAATGGAGGCCATCGTGGAGAGCTTAGGACTTGTGGGA
ATCGCGGATGAGAAGAAGAAGACAGAGGAAGAAGAAGAAGAAGATGACATATTAAAAGACTTACAAGAAG
GAAGCCAGTTAATGGTTGCCAATTGCTACCCGCCATGTCCAGAACCCAATTTAACCCTGGGAATGCCACC
GCATTCCGACTATGGATTCCTCACACTTCTTCTCCAAGATGAGGTTGAGGGCCTACAAATACAATTCCAA
GAAAAATGGATTACTGTCCAACCAATTGCTAATTCATTTGTTGTCAACATTGGTGATCATCTAGAGATAT
TTAGCAACGGAAAATACAAGAGCGTTTTACATAGAGTATCTGTGAACCCTATGAAACCTCGAATATCGGT
GGCTTCTTTGCATAGCCTTCCTTTCCAGAGCATGGTTAGGCCGTCGCCTAAACTGATCAACGAAGCGAAT
CCAAGGCGTTTCAAGGACACCGACTTTGCTAGTTTTCTCGACTACATTTCATCTTCTGAGCCCAAGAGGA
AGAATTTCTTGGATTCTAGGAAATTATTGGATTAA
>XP_018813024.1_2-*Juglans_regia*
ATGTCTCCTACGATGGCTGTAACAACTGAAACAAAGGAGGAGAATGATACGCCAGATAGCGAGTACCAAA
AAGGAATAAAGCACCTCTGGGAAAATGGCATAAACAGAGTTCCCAAGAAGTACATATTACCCCCCTGCGA
TCGACCCATTACTGAAGACGGGGTGCTAAATCATGTATCCGAGCAAAATCTTAAGCTGCCCATCATTGAT
TTCGCAGAACTAATGCAAGGTGCCAACCGGCCTCAAGTCCTCGAGTCCCTCGCTAATGCTTGCGAACAAT
ACGGTTTTTTTCAGCTGGTAAACCATGGCATTCCAAGCGATGTTATAAGCAGCATGATTGACGTTTGTAC
AAGGTTTTTCGAGCTGCCATTTGAGGAAAGATCAAAGTACATGTCTTCGGATATGCAAGCGCCGGTCCGA
TATGGAACCAGCTTTAACCAGAAGAAAGATAATGTGTTTTGTTGGAGAGACTTCTTGAAGCTAATGTGCC
ATCCCCTATCAGATGTCCTCCCACATTGGCCTTCTTCTCCTATGGACCTGAGGAAATTGGCGGCTACCTA
CGCAACAGAAACCAAATACTTGTTTCTAATGCTAATGGAGGCCATCGTGGAGAGCTTAGGACTTGTGGGA
ATCGCGGATGAGAAGAAGAAGACAGAGGAAGAAGAAGAAGAAGATGACATATTAAAAGACTTACAAGAAG
GAAGCCAGTTAATGGTTGCCAATTGCTACCCGCCATGTCCAGAACCCAATTTAACCCTGGGAATGCCACC
GCATTCCGACTATGGATTCCTCACACTTCTTCTCCAAGATGAGGTTGAGGGCCTACAAATACAATTCCAA
GAAAAATGGATTACTGTCCAACCAATTGCTAATTCATTTGTTGTCAACATTGGTGATCATCTAGAGATAT
TTAGCAACGGAAAATACAAGAGCGTTTTACATAGAGTATCTGTGAACCCTATGAAACCTCGAATATCGGT
GGCTTCTTTGCATAGCCTTCCTTTCCAGAGCATGGTTAGGCCGTCGCCTAAACTGATCAACGAAGCGAAT
CCAAGGCGTTTCAAGGACACCGACTTTGCTAGTTTTCTCGACTACATTTCATCTTCTGAGCCCAAGAGGA
AGAATTTCTTGGATTCTAGGAAATTATTGGATTAA
>XP_035544446.1_3-*Juglans_regia*
ATGTCTCCTACGATGGCTGTAACAACTGAAACAAAGGAGGAGAATGATACGCCAGATAGCGAGTACCAAA
AAGGAATAAAGCACCTCTGGGAAAATGGCATAAACAGAGTTCCCAAGAAGTACATATTACCCCCCTGCGA
TCGACCCATTACTGAAGACGGGGTGCTAAATCATGTATCCGAGCAAAATCTTAAGCTGCCCATCATTGAT
TTCGCAGAACTAATGCAAGGTGCCAACCGGCCTCAAGTCCTCGAGTCCCTCGCTAATGCTTGCGAACAAT
ACGGTTTTTTTCAGCTGGTAAACCATGGCATTCCAAGCGATGTTATAAGCAGCATGATTGACGTTTGTAC
AAGGTTTTTCGAGCTGCCATTTGAGGAAAGATCAAAGTACATGTCTTCGGATATGCAAGCGCCGGTCCGA
TATGGAACCAGCTTTAACCAGAAGAAAGATAATGTGTTTTGTTGGAGAGACTTCTTGAAGCTAATGTGCC
ATCCCCTATCAGATGTCCTCCCACATTGGCCTTCTTCTCCTATGGACCTGAGGAAATTGGCGGCTACCTA
CGCAACAGAAACCAAATACTTGTTTCTAATGCTAATGGAGGCCATCGTGGAGAGCTTAGGACTTGTGGGA
ATCGCGGATGAGAAGAAGAAGACAGAGGAAGAAGAAGAAGAAGATGACATATTAAAAGACTTACAAGAAG
GAAGCCAGTTAATGGTTGCCAATTGCTACCCGCCATGTCCAGAACCCAATTTAACCCTGGGAATGCCACC
GCATTCCGACTATGGATTCCTCACACTTCTTCTCCAAGATGAGGTTGAGGGCCTACAAATACAATTCCAA
GAAAAATGGATTACTGTCCAACCAATTGCTAATTCATTTGTTGTCAACATTGGTGATCATCTAGAGATAT
TTAGCAACGGAAAATACAAGAGCGTTTTACATAGAGTATCTGTGAACCCTATGAAACCTCGAATATCGGT
GGCTTCTTTGCATAGCCTTCCTTTCCAGAGCATGGTTAGGCCGTCGCCTAAACTGATCAACGAAGCGAAT
CCAAGGCGTTTCAAGGACACCGACTTTGCTAGTTTTCTCGACTACATTTCATCTTCTGAGCCCAAGAGGA
AGAATTTCTTGGATTCTAGGAAATTATTGGATTAA
>XP_018718607.1_1-*Eucalyptus_grandis*
ATGGGAGAGGTCGATCTGGCGTTTATACAAGACGTCGAACATAGGCCCAAGCTCGCCGCTACACAAGCCG
AAGGCATACCGCTCATCGACCTCGCCGCGCTCGCCGGCTACACACCCTCCGATAGTGCAGTTGATGCTTC
TGCGGCCGCATTCAAAGGGCTCGTGGCGGAGATAGGGGAGGCTTGCAAGAAATGGGGATTCTTTGTCGTG
GTGAACCACGGGGTGGCGGCGGAGAAGAGGGAGAGGATAGAACGGAAGGCGAGGGAGTTCTTCGGGCAGA

GCTTGGAAGAGAAGAAGAAGGTGAGGAGGGACGAGAAGCGGGTGCTGGGGTACTACGAGATGGAGCACAC
CAAGAACGTGAGGGACTGGAAGGAGGTCTTCGACTGTACCGTGCAAGAGCCTATGCCCGTCGCGGCCTCG
CCCGAGGACGGCGAAGAGGCCATCACCGAGTGGATCAATCAGTGGCCTGAGTACCCTCCTGGATTAAGGA
AGGCATGCGAAGAGTACGCACAAGAATTGGAGAAACTCGCGCACAAGCTGATGGGACTGATAGCTCAGAG
CCTGGGTTTGCCCGCAAACAGGTTTGAAGAATTCTACAAGGACCACACCAGCTTCATCCGGCTCAACCAC
TACCCGCCATGCCCCGCACCGCACCTCGCTCTGGGCGTGGGCCGCCACAAGGACTCGGGGGCGCTCACCA
TCCTGGCTCAAGACGACGTTGGAGGCCTCGAGGTGAAAAGGAAGAGTGATGGGGAGTGGGTTCTAGTCAA
GCCCATCCCCGATGCCTTCATTATCAATGTTGGTGACATTATTCAGGTTTGGAGCAACGATACTTATGAG
AGCGTCGAGCATAGAGTGATGGTGAACTCGAAGAAAGAGAGGTTCTCAATTCCGTTCTTCTTCAATCCAT
CACACCATACCATGGTGCAGCCACTGAAGGAATTGACCGATGAGCACAAACCTCCAAAGTACAGGCCATA
CAGCTGGGGCAAGTTCTTGGTCACCCGAATAGGAGGCAACTTCAAGAAACTTGATGTTGAGAACCTCCAA
ATTTATCATTTCAGGATATGA
>XP_010028518.1_2-*Eucalyptus_grandis*
ATGGGAGAGGTCGATCCGGCGTTTATACAAGACGTCGAACATAGGCCCAAGCTCGCCGCCGCACAAGCCG
AAGGCATCCCGCTTATTGACCTCGCTGCCCTCGCTAGCTACTCACCCTCCGATGGCACGGTTGATGCTTC
CACCGTCGCCTTCGAAGGGCTCGTGGCAGAGATAGGGGAGGCTTGCAGGAAGTGGGGATTCTTCGCGGTG
GTGAACCATGGCGTGGCAGCAGAGAAGAGGGAGAGGATAGAGCGGGAGGCGAGGAAGTTCTTCGGGCAGA
GCTTGGAGGAGAAGAAGAAGGTGAGGAGGGACGAAAAGGGGGTGATTGGGTACTACGAGACGGAGCACAC
CAAGAATGTGAGGGACTGGAAGGAGGTTTTCGACTGCACCGTAAAAGAGCCAACGCTCGTCCCGGCCTCA
CCCAAGGATGGTGAGGAGGCGGTCACTGAGTGGATCAATAAGTGGCCTGAGTACCCTCCTGGATTAAGGA
AGGCATGCGAAGAGTACGCACAAGAATTGGAGAAACTCGCGCACAAGCTGATGGGACTGATAGCTCAGAG
CCTGGGTTTGCCCGCAAACAGGTTTGAAGAATTCTACAAGGACCACACCAGCTTCATCCGGCTCAACCAC
TACCCGCCATGCCCCGCACCGCACCTCGCTCTGGGCGTGGGCCGCCACAAGGACTCGGGGGCGCTCACCA
TCCTGGCTCAAGACGACGTTGGAGGCCTCGAGGTGAAAAGGAAGAGTGATGGGGAGTGGGTTCTAGTCAA
GCCCATCCCCGATGCCTTCATTATCAATGTTGGTGACATTATTCAGGTTTGGAGCAACGATACTTATGAG
AGCGTCGAGCATAGAGTGATGGTGAACTCGAAGAAAGAGAGGTTCTCAATTCCGTTCTTCTTCAATCCAT
CACACCATACCATGGTGCAGCCACTGAAGGAATTGACCGATGAGCACAAACCTCCAAAGTACAGGCCATA
CAGCTGGGGCAAGTTCTTGGTCACCCGAATAGGAGGCAACTTCAAGAAACTTGATGTTGAGAACCTCCAA
ATTTATCATTTCAGGATATGA
>XP_018718608.1_3-*Eucalyptus_grandis*
ATGGGAGAGGTCGATCTGGCGTTTATACAAGACGTCGAACATAGGCCCAAGCTCGCCGCTACACAAGCCG
AAGGCATACCGCTCATCGACCTCGCCGCGCTCGCCGGCTACACACCCTCCGATAGTGCAGTTGATGCTTC
TGCGGCCGCATTCAAAGGGCTCGTGGCGGAGATAGGGGAGGCTTGCAAGAAATGGGGATTCTTTGTCGTG
GTGAACCACGGGGTGGCGGCGGAGAAGAGGGAGAGGATAGAACGGAAGGCGAGGGAGTTCTTCGGGCAGA
GCTTGGAAGAGAAGAAGAAGGTGAGGAGGGACGAGAAGCGGGTGCTGGGGTACTACGAGATGGAGCACAC
CAAGAACGTGAGGGACTGGAAGGAGGTCTTCGACTGTACCGTGCAAGAGCCTATGCCCGTCGCGGCCTCG
CCCGAGGACGGCGAAGAGGCCATCACCGAGTGGATCAATCAGTGGCCTGAGTACCCTCCTGGATTAAGGA
AGGCATGCGAAGAGTACGCACAAGAATTGGAGAAACTCGCGCACAAGCTGATGGGACTGATAGCTCAGAG
CCTGGGTTTGCCCGCAAACAGGTTTGAAGAATTCTACAAGGACCACACCAGCTTCATCCGGCTCAACCAC
TACCCGCCATGCCCCGCACCGCACCTCGCTCTGGGCGTGGGCCGCCACAAGGACTCGGGGGCGCTCACCA
TCCTGGCTCAAGACGACGTTGGAGGCCTCGAGGTGAAAAGGAAGAGTGATGGGGAGTGGGTTCTAGTCAA
GCCCATCCCCGATGCCTTCATTATCAATGTTGGTGACATTATTCAGTTTTGGAGCAATGATACTTATCGC
TTGAATATGTGTATTTCAAAGATCATTTGTGCCGCAATTTTTTTTGCCTGTGTCTAA
>XP_010028519.1_4-*Eucalyptus_grandis*
ATGGGAGAGGTCGATCTGGCGTTTATACAAGACGTCGAACATAGGCCCAAGCTCGCCGCTACACAAGCCG
AAGGCATACCGCTCATCGACCTCGCCGCGCTCGCCGGCTACACACCCTCCGATAGTGCAGTTGATGCTTC
TGCGGCCGCATTCAAAGGGCTCGTGGCGGAGATAGGGGAGGCTTGCAAGAAATGGGGATTCTTTGTCGTG
GTGAACCACGGGGTGGCGGCGGAGAAGAGGGAGAGGATAGAACGGAAGGCGAGGGAGTTCTTCGGGCAGA
GCTTGGAAGAGAAGAAGAAGGTGAGGAGGGACGAGAAGCGGGTGCTGGGGTACTACGAGATGGAGCACAC
CAAGAACGTGAGGGACTGGAAGGAGGTCTTCGACTGTACCGTGCAAGAGCCTATGCCCGTCGCGGCCTCG
CCCGAGGACGGCGAAGAGGCCATCACCGAGTGGATCAATCAGTGGCCTGAGTACCCTCCTGGATTAAGGG
AGGCATGCGAAGAGTACGCACAAGAGTTGAAGAAACTCGCTTACAAGCTGATAAGACTGATAGCTCAGAG
CCTGGGCTTGCCCGCGAACAGGTTTGACGAATTCTACAAGGACCAGACCAGCTTCATCCGGCTCAACCAC
TACCCGCCATGCCCCGTGCCGCACCTCGCTCTGGGCTTGGGCCGCCACAAGGACTCAGGGGCGCTCACCA

TCCTGGCTCAAGACGACGTCGAAGGCCTCCAGGTGAAGAAGAAGAGCGATGGGGAGTGGATAGTAGTCAA
GCCCATCCCCGATGCCTTCATTATCAATGTTGGCAGCATTATGCAGGTCTGGAGCAACGACATTTACGAA
AGCGTCGAGCATAGAGTGATGGTGAACTCGAAGAAGGAGAGGTTCTCGATCCCATTCTTCTTCAACCCAT
CACACTACACCATGGTGCAGCCACTGAAGGAATTGACTGATGAACACAACCCTCCAAAGTACAGGGCATA
CAAATGGGGTAAGTTCTTGGTCAACCGAGCAAGAAGCAACTTGAAGAAACTTGATGTTGAGAACCTCCAA
ATTTATCATTTCAGGTTATGA
>XP_020418878.1_1-*Prunus_persica*
ATGGGAGAAGTCAACCCGTCATGCATCCTTTCAGAAGAACACAGGCCCAAACATGTCATCACTGAAGGTG
AAGGAATCCCATTGATCGATCTCTCTCCAATAACCAACAGGGAAGGGTTATCAGCTGATCCTAATAAGGC
AGTGGAAGACCTTGCTGCTAAGATAGGTGAGGCTTGCAGGACTTGGGGGTTCTTCAGTGTGATCAACCAT
GGCGTGCCTTTGGGCATCCGGAGAAGAATCATGGAGGTGGCACGGAAATTTTTCGCATTGCCGGTGGAGG
AGAAGAAGAAAGTGAGCAGAGAGGACCATAACACTGCAGGGTTTCATAACGATGAGCATAGCAAAGACTT
TAAGGATTGGAAAGAGGTTTATGATTTTTATGTTAATGATGGGATGCTAATGCCGGCTTCCCATGAACTT
GATGACCCTGAAATTGTTCCGTGGTTCACTCCATGGCCCGAGAACCTACCCGAGTTCAGGGAGACATGCG
AAGAATACGGTCGAGCATGTGAGAAGCTATTCTTCAATTTGTTGGAACTTGTCAGCCTCAGCTTAGGCTT
ACCCCCGGAGAGGCTGCATGGGTACTTTGAGAATCAAGCAAGCTTTGCCCGACTCAATTACTACCCTCCA
TGTCCCAAACCGGAACTTGTTCTTGGCACCGGTGGACACAAGGACCCTAGTGCTCTAACGGTCCTCGCTC
AAGAAGATGTCGAAGGCCTCGACGTGCTTCGAAAATCAGACGGAGCATGGGTTCGTGTCAAGCCTGTCCC
GGATTCCTTTGTCATCAATGTTGGCGATGTTCTTCAGGTATGGAGCAATGACCTTTACGAAAGTGTCGAG
CATCGGGCCGAGGTGAATGCAGAAACAGAAAGGTATTCCATCCCTATATTTTTTCACCCTTCTCATGATG
TAACCATGAAGCCGTTGGATGAGTTGGTTGATGAACAAAGCCCGGCCAAGTACCCCGAATACAAGGCAGG
GAAGTGGCTGAATTTGAGGATGTATAATAACTACAAGAAACATGGCTTCTATATGCGGATGACTGACTAC
AAGGTTGCTGCTTAG
>XP_020418879.1_2-*Prunus_persica*
ATGGGAGAAGTCAACCCGTCATGCATCCTTTCAGAAGAACACAGGCCCAAACATGTCATCACTGAAGGTG
AAGGAATCCCATTGATCGATCTCTCTCCAATAACCAACAGGGAAGGGTTATCAGCTGATCCTAATAAGGC
AGTGGAAGACCTTGCTGCTAAGATAGGTGAGGCTTGCAGGACTTGGGGGTTCTTCAGTGTGATCAACCAT
GGCGTGCCTTTGGGCATCCGGAGAAGAATCATGGAGGTGGCACGGAAATTTTTCGCATTGCCGGTGGAGG
AGAAGAAGAAAGTGAGCAGAGAGGACCATAACACTGCAGGGTTTCATAACGATGAGCATAGCAAAGACTT
TAAGGATTGGAAAGAGGTTTATGATTTTTATGTTAATGATGGGATGCTAATGCCGGCTTCCCATGAACTT
GATGACCCTGAAATTGTTCCGTGGTTCACTCCATGGCCCGAGAACCTACCCGAGTTCAGGGAGACATGCG
AAGAATACGGTCGAGCATGTGAGAAGCTATTCTTCAATTTGTTGGAACTTGTCAGCCTCAGCTTAGGCTT
ACCCCCGGAGAGGCTGCATGGGTACTTTGAGAATCAAGCAAGCTTTGCCCGACTCAATTACTACCCTCCA
TGTCCCAAACCGGAACTTGTTCTTGGCACCGGTGGACACAAGGACCCTAGTGCTCTAACGGTCCTCGCTC
AAGAAGATGTCGAAGGCCTCGACGTGCTTCGAAAATCAGACGGAGCATGGGTTCGTGTCAAGCCTGTCCC
GGATTCCTTTGTCATCAATGTTGGCGATGTTCTTCAGGTATGGAGCAATGACCTTTACGAAAGTGTCGAG
CATCGGGCCGAGGTGAATGCAGAAACAGAAAGGTATTCCATCCCTATATTTTTTCACCCTTCTCATGATG
TAACCATGAAGCCGTTGGATGAGTTGGTTGATGAACAAAGCCCGGCCAAGTACCCCGAATACAAGGCAGG
GAAGTGGCTGAATTTGAGGATGTATAATAACTACAAGAAACATGGCTTCTATATGCGGATGACTGACTAC
AAGGTTGCTGCTTAG
>XP_007222213.2_3-*Prunus_persica*
ATGCAACTATATATACACGTGCCATGGCACCAATGCTTAACGATCCAAAGCTATAAACCATCAACATATA
CCATGGGAGAAGTCAACCCATCATACATCCTTTCAGAAGAACACAGGCCCAAACACGTCATCACTGAAGG
TGAAGGAATCCCATTGATCGATCTCTCTCCAATAACCAACAGGGAAGGGTTATCAGCTGATCCCAATAAG
GCAGTGGAAGACCTTGCTGCTAAGATAGGTGAGGCTTGCAGGACTTGGGGGTTCTTCAGTGTGATCAACC
ATGGCGTGCCTTTGGGCATCCGGAGAAGAATCATGGAGGTGGCACGGAAATTTTTCGCATTGCCGGTGGA
GGAGAAGAAGAAAGTGAGCAGAGAGGACCATAACACTGCAGGGTTTCATAACGATGAGCATAGCAAAGAC
TTTAAGGATTGGAAAGAGGTTTATGATTTTTATGTTAATGATGGGATGCTAATGCCGGCTTCCCATGAAC
TTGATGACCCTGAAATTGTTCCGTGGTTCACTCCATGGCCCGAGAACCTACCCGAGTTCAGGGAGACATG
CGAAGAATACGGTCGAGCATGTGAGAAGCTATTCTTCAATTTGTTGGAACTTGTCAGCCTCAGCTTAGGC
TTACCCCCGGAGAGGCTGCATGGGTACTTTGAGAATCAAGCAAGCTTTGCCCGACTCAATTACTACCCTC
CATGTCCCAAACCGGAACTTGTTCTTGGCACCGGTGGACACAAGGACCCTAGTGCTCTAACGGTCCTCGC
TCAAGAAGATGTCGAAGGCCTCGACGTGCTTCGAAAATCAGACGGAGCATGGGTTCGTGTCAAGCCTGTC
CCGGATTCCTTTGTCATCAATGTTGGCGATGTTCTTCAGGTATGGAGCAATGACCTTTACGAAAGTGTCG

AGCATCGGGCCGAGGTGAATGCAGAAACAGAAAGGTATTCCATCCCTATATTTTTTCACCCTTCTCATGA
TGTAACCATGAAGCCGTTGGATGAGTTGGTTGATGAACAAAGCCCGGCCAAGTACCCCGAATACAAGGCA
GGGAAGTGGCTGAATTTGAGGATGTATAATAACTACAAGAAACATGGCTTCTATATGCGGATGACTGACT
ACAAGGTTGCTGCTTAG
>NP_001233840.2_1-*Solanum_lycopersicum*
ATGGAAACCAAAGTTATTTCTAGCGGAATCAACCACTCTACTCTTCCTCAAAGTTACATCCGACCCGAAT
CCGATAGACCACGTCTATCGGAAGTGGTCGATTGTGAAAATGTTCCAATAATTGACTTAAGTTGCGGAGA
TCAAGCTCAAATAATTCGTCAAATTGGAGAAGCTTGTCAAACTTATGGTTTCTTTCAGGTAATTAATCAT
GGTGTACCAAAGGAAGTTGTAGAGAAAATGCTAGGGGTAGCTGGGGAATTTTTCAATTTACCAGTAGAAG
AGAAACTAAAATTATATTCAGATGATCCTTCAAAGACCATGAGATTATCAACAAGTTTTAATGTTAAAAA
GGAGACAGTTCATAATTGGAGAGATTATCTCAGACTTCATTGTTATCCTCTAGAGAAGTATGCTCCTGAA
TGGCCTTCTAATCCATCATCTTTCAGGGAAATCGTGAGCAGATATTGCAGGGAAATTCGTCAACTCGGAT
TTAGATTAGAAGAAGCCATAGCAGAAAGCCTGGGGTTAGATAAAGAGTGTATAAAAGATGTATTGGGTGA
ACAAGGACAACATATGGCTATCAATTATTATCCTCCTTGTCCACAACCAGAACTTACTTATGGGCTTCCG
GCCCATACTGATCCAAATTCACTTACAATTCTTCTTCAAGACTTGCAAGTTGCGGGTCTTCAAGTTCTTA
AAGATGGCAAATGGTTAGCTGTAAAACCTCAACCTGACGCCTTTGTCATTAATCTTGGGGATCAATTGCA
GGCAGTAAGTAACGGTAAGTACAGAAGTGTATGGCATCGAGCTATTGTGAATTCAGATCAAGCTAGGATG
TCAGTGGCTTCGTTTCTATGTCCGTGTGATAGCGCGAAAATCAGTGCACCAAAGCTGCTGACAGAAGATG
GATCTCCAGTGATTTATCAAGACTTTACGTATGCTGAGTATTACAACAAGTTCTGGAGCAGGAATTTGGA
CCAGCAACATTGTTTGGAACTTTTCAAGAACTAA
>XP_019081017.1_1-*Vitis_vinifera*
ATGGCCAATGCAAAGCTTCTGTTGTCTGACCTTGCATCCAGTATAGACTGTGTTCCTTCAAGATATGTTC
GACCCGTGAACGACCGCCCAAACCTTGATGAGGTTCAGTCCTCATTGGATGGCTCCATTCCCCTCATCGA
CCTCCAAGACCTCCATGGTCCTAGCCGCTCCCATGTTATCAAGCAGATAGCTGAAGCCTGCCAGATTGAT
GGTTTTTTTCGGGTCAAGAACCATGGAATACCGGAGAGTGTGATCCATGGCATGTTGAGCATAACAAAGG
AGTTTTTCCATTTACCGGAAAGTGAAAGGTTGAAGAACTACTCCGACGACCCTTTGAAGACTATGAGACT
CTCTACTAGTTTCAATGTGAAAACCGAGCAGGTGTCCAACTGGAGAGATTTCCTGAGACTCTATTGCTAC
CCTTTAGAGGATTACATTCAGGAATGGCCCTCCAACCCTCCATCTTTCAGAGAAGTTGTGGCCGAGTATT
GCAAGGAGGCTAGAAAATTAGCATTACTACTGCTTGAAGCAATATCCGAAAGCTTAGGACTAGAGAGGAA
CCACATAGATAAGGCGCTGGGAAAGCACAGCCAGCAGATGGCCCTCAACTACTACCCACCTTGTCCTCAA
CCAGAGCTGACGTTTGGCTTACCTGGCCATGCTGATCCTAATGCCCTCACAATCCTCCTTCAAGACGATG
TGCCCGGTTTGCAGGTGCTCAAAGATGGAAAATGGGTGGCCATCCACCCTATTCCAAATACTTTCATTGT
CAACATTGGTGATCAAATCCAGGTGATGGAGACAAAAGTACTTAGCAATGATTGCTACAAGAGTGCGGTC
CATAGGGCCGTGGTGAACTGCCAGAAGGAGAGGATCTCCATTCCAACCTTTTATTGCCCATCACCAGATG
CAGTTATAGGACCAGCTCCGGGACTGGTCGACCATGGTCATCCTGCCCTTTACAGGAAATTTACATACAG
TGAGTACTTTGGGAAATTCTGGAACAGGGGCCTTGCTACTCAATCCTGCTTGGACATGTTCAAGACGTAG
>XP_002267625.1_2-*Vitis_vinifera*
ATGGCCAATGCAAAGCTTCTGTTGTCTGACCTTGCATCCAGTATAGACTGTGTTCCTTCAAGATATGTTC
GACCCGTGAACGACCGCCCAAACCTTGATGAGGTTCAGTCCTCATTGGATGGCTCCATTCCCCTCATCGA
CCTCCAAGACCTCCATGGTCCTAGCCGCTCCCATGTTATCAAGCAGATAGCTGAAGCCTGCCAGATTGAT
GGTTTTTTTCGGGTCAAGAACCATGGAATACCGGAGAGTGTGATCCATGGCATGTTGAGCATAACAAAGG
AGTTTTTCCATTTACCGGAAAGTGAAAGGTTGAAGAACTACTCCGACGACCCTTTGAAGACTATGAGACT
CTCTACTAGTTTCAATGTGAAAACCGAGCAGGTGTCCAACTGGAGAGATTTCCTGAGACTCTATTGCTAC
CCTTTAGAGGATTACATTCAGGAATGGCCCTCCAACCCTCCATCTTTCAGAGAAGTTGTGGCCGAGTATT
GCAAGGAGGCTAGAAAATTAGCATTACTACTGCTTGAAGCAATATCCGAAAGCTTAGGACTAGAGAGGAA
CCACATAGATAAGGCGCTGGGAAAGCACAGCCAGCAGATGGCCCTCAACTACTACCCACCTTGTCCTCAA
CCAGAGCTGACGTTTGGCTTACCTGGCCATGCTGATCCTAATGCCCTCACAATCCTCCTTCAAGACGATG
TGCCCGGTTTGCAGGTGCTCAAAGATGGAAAATGGGTGGCCATCCACCCTATTCCAAATACTTTCATTGT
CAACATTGGTGATCAAATCCAGGTACTTAGCAATGATTGCTACAAGAGTGCGGTCCATAGGGCCGTGGTG
AACTGCCAGAAGGAGAGGATCTCCATTCCAACCTTTTATTGCCCATCACCAGATGCAGTTATAGGACCAG
CTCCGGGACTGGTCGACCATGGTCATCCTGCCCTTTACAGGAAATTTACATACAGTGAGTACTTTGGGAA
ATTCTGGAACAGGGGCCTTGCTACTCAATCCTGCTTGGACATGTTCAAGACGTAG
>XP_034927313.1_1-*Populus_alba*
ATGGGAGATTTAGATTTGTCCTTCATTCAAGATCTTGAAAACAGGCCTCATGTCAAAGTCATCGAACCTG

AGGAAGTCCCGGTAATCGATTTCTTCACTTCAAGCCACGGAGACACCAAAGAAATTGTCTCAGAGATAGG
AAATGCATGCAGGAAGTGGGGATTCTTTCAAGTAATTAACCATGGTGTACCTCTAGAGTTATCTAAAAGA
ATGGCGAAGATGGCGAAAGAGTTCTTTGATCAACCAATCGAGGAAAAAAAGAAGGTGAAGCGAGACGAAG
GGAATTCTATGGGGTATCATGATAGTGAGCATACTAAGAATGTTAGGGACTGGAAGGAAGTGTTTGATTT
CTCACTGGAAGATCCTAGACTTGTCCCAGCCATGGATAACCTAGATGATCAGGAATTGAGGACCATAAGT
AATCGTTGGCCTCAGTACCCTAATGAATTTAGGAAAGCATGTCAGGAGTATTCTCGAGAAGTGGAGAAGC
TTGGTTATAGGCTGTTGAAACTTGTTTCTTTGAGCTTAGGCTTGCCTGCTAACAGGCTAAATGGCTTCTT
CATTGACCAAACCAGCCTCTGTAGGATAAATCACTATCCTCCTTGCCCTAACCCCAACCTAGCTCTTGGG
GTTGGTCGACACAAAGATTTCAGTGCCTTAACTGTGCTTGCTCAGGATGATGTTGGAGGATTGGAAATAA
GACGAAAATCAGACGGTGAATGGATACCAGTTTGGAGTAATGACACCTATGAGAGTGTGGAGCACAGGGT
TGTAGTGAACACTGAGAAGGAAAGGTTCTCAATTCCAGTACTACTCTTACCTGCTCACCATGTCAACATC
GAGCCACTGGAAGAGCTACTAAACGAGCAGAACCCTTCCAAATACCAACGATATAATTGGGGGAAGTTCT
ATGCTAGCAGAATCCGCGGTGATTACAAGAAACGGGATGTAGAAAATATCCAAATTCATCATTTGAAAAA
GCAACATCCATAG
>XP_034927312.1_2-*Populus_alba*
ATGGGAGATTTAGATTTGTCCTTCATTCAAGATCTTGAAAACAGGCCTCATGTCAAAGTCATCGAACCTG
AGGAAGTCCCGGTAATCGATTTCTTCACTTCAAGCCACGGAGACACCAAAGAAATTGTCTCAGAGATAGG
AAATGCATGCAGGAAGTGGGGATTCTTTCAAGTAATTAACCATGGTGTACCTCTAGAGTTATCTAAAAGA
ATGGCGAAGATGGCGAAAGAGTTCTTTGATCAACCAATCGAGGAAAAAAAGAAGGTGAAGCGAGACGAAG
GGAATTCTATGGGGTATCATGATAGTGAGCATACTAAGAATGTTAGGGACTGGAAGGAAGTGTTTGATTT
CTCACTGGAAGATCCTAGACTTGTCCCAGCCATGGATAACCTAGATGATCAGGAATTGAGGACCATAAGT
AATCGTTGGCCTCAGTACCCTAATGAATTTAGGAAAGCATGTCAGGAGTATTCTCGAGAAGTGGAGAAGC
TTGGTTATAGGCTGTTGAAACTTGTTTCTTTGAGCTTAGGCTTGCCTGCTAACAGGCTAAATGGCTTCTT
CATTGACCAAACCAGCCTCTGTAGGATAAATCACTATCCTCCTTGCCCTAACCCCAACCTAGCTCTTGGG
GTTGGTCGACACAAAGATTTCAGTGCCTTAACTGTGCTTGCTCAGGATGATGTTGGAGGATTGGAAATAA
GACGAAAATCAGACGGTGAATGGATACCAGTTAAGCCAATTCCAGATGCATTTATCGTTAATGTCGGCGA
CATTATTCAGGTTTGGAGTAATGACACCTATGAGAGTGTGGAGCACAGGGTTGTAGTGAACACTGAGAAG
GAAAGGTTCTCAATTCCAGTACTACTCTTACCTGCTCACCATGTCAACATCGAGCCACTGGAAGAGCTAC
TAAACGAGCAGAACCCTTCCAAATACCAACGATATAATTGGGGGAAGTTCTATGCTAGCAGAATCCGCGG
TGATTACAAGAAACGGGATGTAGAAAATATCCAAATTCATCATTTGAAAAAGCAACATCCATAG
>NP_197841.1_1-*Arabidopsis_thaliana*
ATGGCGGCAAAGCTGATATCCACCGGTTTCCGTCATACTACTTTGCCGGAAAACTATGTCCGGCCAATCT
CCGACCGTCCACGTCTCTCTGAAGTCTCTCAACTCGAAGATTTCCCTCTCATCGATCTCTCTTCCACTGA
TCGATCTTTTCTCATCCAACAAATCCACCAAGCTTGTGCCCGATTCGGATTTTTTCAGGTCATAAATCAC
GGAGTTAACAAACAAATAATAGATGAGATGGTGAGTGTTGCGCGTGAGTTCTTTAGCATGTCTATGGAAG
AAAAAATGAAGCTATATTCAGACGATCCAACGAAGACAACAAGATTATCGACGAGCTTCAATGTGAAGAA
AGAAGAAGTCAACAATTGGAGAGACTATCTAAGACTCCATTGTTATCCTATCCACAAGTATGTCAATGAG
TGGCCGTCAAACCCTCCTTCTTTCAAGGAAATAGTAAGTAAATACAGTAGAGAAGTAAGAGAAGTGGGAT
TTAAAATAGAGGAATTAATATCAGAGAGCTTAGGTTTAGAAAAAGATTACATGAAGAAAGTGCTTGGTGA
ACAAGGTCAACACATGGCAGTCAACTATTATCCTCCATGTCCTGAACCTGAGCTCACTTACGGTTTACCT
GCTCATACCGACCCAAACGCCCTAACCATTCTTCTTCAAGACACTACTGTTTGCGGTCTCCAGATCTTGA
TCGACGGTCAGTGGTTCGCCGTTAATCCACATCCTGATGCTTTTGTCATCAACATAGGTGACCAGTTACA
GGCATTAAGTAATGGAGTATACAAAAGTGTTTGGCATCGCGCTGTAACAAACACAGAAAATCCGAGACTA
TCGGTCGCATCGTTTCTGTGCCCAGCTGACTGTGCTGTCATGAGCCCGGCCAAGCCCTTGTGGGAAGCTG
AGGACGATGAAACGAAACCAGTCTACAAAGATTTCACTTATGCAGAGTATTACAAGAAGTTTTGGAGTAG
GAATCTGGACCAAGAACATTGCCTCGAGAATTTTCTAAACAACTAA
>XP_030942678.1_1-*Quercus_lobata*
ATGGCTTCAGCAGTGTCCACGAAGGCATCACCTGTTCATCCACCAAAGATAACAACCGTGAAAGCACTCG
TTGAATCAACTGGCACCTCCTCCATCCCTTCGTTTTACAACTTCACCCCTTATCTCCGTGATGAACCAAT
AGCCAATGATCCAGAAGATTCAATCCCCATTATCGACCTCTCTCTTCTTGTTTCTGGTACTCCTGAAGAA
CAGTCCCAAGTCATCCATCAACTCAGCAATGCCTGCTCAGACTGGGGCTGCTTCATGGTGATTAATCATG
ATGTGACGGAGAGTCTGACGAAGGCAATGATAGGTTCGTTTCAAGAATTTTTCGACTTGCCAGAAGAGGA
GAAGAGAGAGTATCAAGGAAACCATGTCATGGATCCAATCAGGTGTGGTACAGGCTTTAATCCTTCAATG
GATAAAGCAAACTATTGGAGAGATTTTCTCAAGTGCTTCGCGCATCCTGAATTTCATACACCCAACAAAC

CTGCTGGGTTTAGTGAGATTGCAAGGGAGTTCAGCAAAAGAAACCGGGAAGTAATAATAATATTACTGAA
AGCTATATCAAAGAGCTTGGGGTTAGAAGAGAGCTACGTAGAAAAGGCTGCCAATTTCGAGTTGGGTTTA
CAATTGCTCGCTGCTAACTATTATCCAGCTTGTCCAGAGCCAGAGAAAGCAATTGGCATCCCCGCTCACT
ATGACCATGGTTTGTTAACCACTTTGGTAAATAATGGTATCTCTGGCCTTCAAGTAAAGCATAATGGAAA
ATGGTTTAATGTCAACATCCCTCCCAATGGACTCTTTGTTCAAGTTGCTGATCACTTGGAGATTTTGAGC
AATGGCAAGTACAAGAGTATTGCACACCGGGCACTTGTGAATAACAAGGCTACAAGGATGTCCATAGCTC
TAGCACATGGACCAGCACTAGACACAGTTATGAGGCCAGCTCCAAAGTTAGTTGACAATGAAAGTGATCA
ACCTGCATATGTTGAAATGACGTATGGAAAATTTTTGGAATTGCAGCAAACTGGGAGGCTGAGTTCTAAA
TTCCGGTCAGATACTGAACAAAACAAAGCAGTCTGA
>XP_030942679.1_2-*Quercus_lobata*
ATGGCTTCAGCAGTGTCCACGAAGGCATCACCTGTTCATCCACCAAAGATAACAACCGTGAAAGCACTCG
TTGAATCAACTGGCACCTCCTCCATCCCTTCGTTTTACAACTTCACCCCTTATCTCCGTGATGAACCAAT
AGCCAATGATCCAGAAGATTCAATCCCCATTATCGACCTCTCTCTTCTTGTTTCTGGTACTCCTGAAGAA
CAGTCCCAAGTCATCCATCAACTCAGCAATGCCTGCTCAGACTGGGGCTGCTTCATGGTGATTAATCATG
ATGTGACGGAGAGTCTGACGAAGGCAATGATAGGTTCGTTTCAAGAATTTTTCGACTTGCCAGAAGAGGA
GAAGAGAGAGTATCAAGGAAACCATGTCATGGATCCAATCAGGTGTGGTACAGGCTTTAATCCTTCAATG
GATAAAGCAAACTATTGGAGAGATTTTCTCAAGTGCTTCGCGCATCCTGAATTTCATACACCCAACAAAC
CTGCTGGGTTTAGTGAGATTGCAAGGGAGTTCAGCAAAAGAAACCGGGAAGTAATAATAATATTACTGAA
AGCTATATCAAAGAGCTTGGGGTTAGAAGAGAGCTACGTAGAAAAGGCTGCCAATTTCGAGTTGGGTTTA
CAATTGCTCGCTGCTAACTATTATCCAGCTTGTCCAGAGCCAGAGAAAGCAATTGGCATCCCCGCTCACT
ATGACCATGGTTTGTTAACCACTTTGGTAAATAATGGTATCTCTGGCCTTCAAGTAAAGCATAATGGAAA
ATGGTTTAATGTCAACATCCCTCCCAATGGACTCTTTGTTCAAGTTGCTGATCACTTGGAGATTTTGAGC
AATGGCAAGTACAAGAGTATTGCACACCGGGCACTTGTGAATAACAAGGCTACAAGGATGTCCATAGCTC
TAGCACATGGACCAGCACTAGACACAGTTATGAGGCCAGCTCCAAAGTTAGTTGACAATGAAAGTGATCA
ACCTGCATATGTTGAAATGACGTATGGAAAATTTTTGGAATTGCAGCAAACTGGGAGGCTGAGTTCTAAA
TTCCGGTCAGATACTGAACAAAACAAAGCAGTCTGA
>XP_022029426.1_1-*Helianthus_annuus*
ATGGCAACAATCACCAAACGATTACTCCTAAGCGACCTTGTATCCACCGATAAAATAGACCAAGTCCCTT
CAAACTACATCCGACCCATCACCCAACGTCCCAATCTACAATCCGTTGTTCGCGACTCCATCCCTCTAAT
AGACCTCCACAACCTCTTCGGCCCGAACCGCGGTCAAGTCATCGACCAAATCGGTCGAGCCTGCCGCGAC
TATGGCTTCTTCCAAGTCAAAAACCATAGCGTCCCCGAACTTACAATATCCAACATGATGCAAACCGCTA
GAGACTTCTTCAACCTACCCGAACAAGAACGACTCAAAAACTATTCAGACGACCCCCAAAAAACCACTAG
GCTCTCCACCAGCTTCAACATTCGAACCGAAAAGGTTGCTAACTGGAGAGATTACTTGCGTCTTCATTGC
TACCCGATTGAAAACTTTGTTCACGAATGGCCAACTAACCCGACGTCGTTTCGGGCCCATGTGGCGGAGT
ATTGTACGAGTGTAAGAGGGTTAGCGGTGCAGCTTATTGAGGCGATTTCGGAGAGTTTGGGGCTTGAGAA
GGAGTATATAGGGAGGCAATTGGGGAAGCATGCTCAACATATGGCTTTGAATTACTACCCGCCTTGCCCG
CAACCCGATTTGACTTATGGGTTGCCTGGACATACTGATCTTAATCTTATTACTATTCTTCTTCAAGATC
AGGTTCCTGGGCTTCAGGTCTTGAGAAATGGTCATTGGGTTGCGGTTGATCCGGTTCCGAATACGTTTAT
TATTAATATTGGTGATCAAATTCAGGTAATGAGCAATGATAAATACAAGAGTGTGTTGCATCGAGCTGTG
GTGAACTGTGATAAAGAACGGATATCTATACCTACTTTCTATTGCCCGTCACCTGAGGCGGTTATCGGTC
CTGCTCCGGAACTCGTGACCGACGATGAGCCTGCTGTGTACAGACAGTTTACATACGGGGAATACTATGA
GAAGTTTTGGAACAGGGGGCTTGCAACCGAGAGTTGCTTGGATATGTTCATGGCTACTTGA
>XP_008236676.1_1-*Prunus_mume*
ATGGTACCTTTCTACCAGCTCATGACCAAGAATGAAAAATTTGAGATGGCTGAAGTTGCTGCTGCTCAAA
GCACGGAGGCATTGAAAGTAACGAGCATCAAATCACTTGCTGAATCACCTGCTCTCAACTCTGTTCCTTC
TGCATATGGCTTCACCATAAACCCCAACGATGAAGCAGATCCCAACGACCCTGAATTCGCAATTCCCATC
GTCGACATGTCTCTTCTCACCTCAGGATCTCCAGAACAACGGTCAAAAATCATCCATGACCTCGTCAAAA
TTTGTCAAGAGTGGGGCTTCTTCATAGCCATTAACCATGGAGTCCCTGAGAGCCTCATGAAGGCGATGAT
CGAAACATGCCATGGATTTTTCAGTTTACCAGACGAGGAGAAGAATGAGTTCAAATCGGGGAACGATGTG
CTAGAGATGTTCAAGTATGGAACTAGCTATAATCTGGCATTGGATAAATTCCTTCTCTGGAGGGACTTCT
TCAAGGTCAGAGTGCATCCCGAATTCTACTCCCTCTACAAGCCGGCTTGCTTCAGTGAAGTCTCAATGGA
GTTCAGCAAGAGAGCCCGAGAAGTTGCGTTGGAGATAACAAGAGCAATCTCAGAAAGCTTGGGGTTGGAG
CCAAACTACATACACAACGCCATGAACATGGATCGTGGCATACAAATGCTCGCCGCCAACTACTACCCGC
CTTGCCCTCGGCCAGAACATGCAATTGGCATACCCCATCACACTGATCATGGCCTTGTCACCCTTCTCAT

CCAGAATGAGATGAAAGGCCTCCAAGTTGAGCACAACGGCAAATGGCTCACCGTTGATGGCCCTCCCAAC
GGTTTCTTTGTTAACCTCGCTGACCAGATGCAGATTCTTACTAATGGTAAATACAAGAGCGTAATGCATC
GAGCCACCGTGAACAACAAAGCTACAAGGATATCGATCGCCATACCGCATGGACCATCTGTAGATACAGT
TGTTGCACCAGCTCCAGAGTTGTTGGAAAAGGAAGGCCAAGCTCCTAAATACCTTGCCATGAACTACAAG
GAATATATACAGCTTCAACAAAGTGGCAAGAACTATATGAAGTCTACCTTTGATCACATACGAGTCTAA
>XP_016650738.1_2-*Prunus_mume*
ATGGTACCTTTCTACCAGCTCATGACCAAGAATGAAAAATTTGAGATGGCTGAAGTTGCTGCTGCTCAAA
GCACGGAGGCATTGAAAGTAACGAGCATCAAATCACTTGCTGAATCACCTGCTCTCAACTCTGTTCCTTC
TGCATATGGCTTCACCATAAACCCCAACGATGAAGCAGATCCCAACGACCCTGAATTCGCAATTCCCATC
GTCGACATGTCTCTTCTCACCTCAGGATCTCCAGAACAACGGTCAAAAATCATCCATGACCTCGTCAAAA
TTTGTCAAGAGTGGGGCTTCTTCATAGCCATTAACCATGGAGTCCCTGAGAGCCTCATGAAGGCGATGAT
CGAAACATGCCATGGATTTTTCAGTTTACCAGACGAGGAGAAGAATGAGTTCAAATCGGGGAACGATGTG
CTAGAGATGTTCAAGTATGGAACTAGCTATAATCTGGCATTGGATAAATTCCTTCTCTGGAGGGACTTCT
TCAAGGTCAGAGTGCATCCCGAATTCTACTCCCTCTACAAGCCGGCTTGCTTCAGTGAAGTCTCAATGGA
GTTCAGCAAGAGAGCCCGAGAAGTTGCGTTGGAGATAACAAGAGCAATCTCAGAAAGCTTGGGGTTGGAG
CCAAACTACATACACAACGCCATGAACATGGATCGTGGCATACAAATGCTCGCCGCCAACTACTACCCGC
CTTGCCCTCGGCCAGAACATGCAATTGGCATACCCCATCACACTGATCATGGCCTTGTCACCCTTCTCAT
CCAGAATGAGATGAAAGGCCTCCAAGTTGAGCACAACGGCAAATGGCTCACCGTTGATGGCCCTCCCAAC
GGTTTCTTTGTTAACCTCGCTGACCAGATATTCTTACTAATGGTAAATACAAGAGCGTAA
>XP_016650735.1_3-*Prunus_mume*
ATGGCTGAAGTTGCTGCTGCTCAAAGCACGGAGGCATTGAAAGTAACGAGCATCAAATCACTTGCTGAAT
CACCTGCTCTCAACTCTGTTCCTTCTGCATATGGCTTCACCATAAACCCCAACGATGAAGCAGATCCCAA
CGACCCTGAATTCGCAATTCCCATCGTCGACATGTCTCTTCTCACCTCAGGATCTCCAGAACAACGGTCA
AAAATCATCCATGACCTCGTCAAAATTTGTCAAGAGTGGGGCTTCTTCATAGCCATTAACCATGGAGTCC
CTGAGAGCCTCATGAAGGCGATGATCGAAACATGCCATGGATTTTTCAGTTTACCAGACGAGGAGAAGAA
TGAGTTCAAATCGGGGAACGATGTGCTAGAGATGTTCAAGTATGGAACTAGCTATAATCTGGCATTGGAT
AAATTCCTTCTCTGGAGGGACTTCTTCAAGGTCAGAGTGCATCCCGAATTCTACTCCCTCTACAAGCCGG
CTTGCTTCAGTGAAGTCTCAATGGAGTTCAGCAAGAGAGCCCGAGAAGTTGCGTTGGAGATAACAAGAGC
AATCTCAGAAAGCTTGGGGTTGGAGCCAAACTACATACACAACGCCATGAACATGGATCGTGGCATACAA
ATGCTCGCCGCCAACTACTACCCGCCTTGCCCTCGGCCAGAACATGCAATTGGCATACCCCATCACACTG
ATCATGGCCTTGTCACCCTTCTCATCCAGAATGAGATGAAAGGCCTCCAAGTTGAGCACAACGGCAAATG
GCTCACCGTTGATGGCCCTCCCAACGGTTTCTTTGTTAACCTCGCTGACCAGATGCAGATTCTTACTAAT
GGTAAATACAAGAGCGTAATGCATCGAGCCACCGTGAACAACAAAGCTACAAGGATATCGATCGCCATAC
CGCATGGACCATCTGTAGATACAGTTGTTGCACCAGCTCCAGAGTTGTTGGAAAAGGAAGGCCAAGCTCC
TAAATACCTTGCCATGAACTACAAGGAATATATACAGCTTCAACAAAGTGGCAAGAACTATATGAAGTCT
ACCTTTGATCACATACGAGTCTAA
>XP_016650736.1_4-*Prunus_mume*
ATGGCTGAAGTTGCTGCTGCTCAAAGCACGGAGGCATTGAAAGTAACGAGCATCAAATCACTTGCTGAAT
CACCTGCTCTCAACTCTGTTCCTTCTGCATATGGCTTCACCATAAACCCCAACGATGAAGCAGATCCCAA
CGACCCTGAATTCGCAATTCCCATCGTCGACATGTCTCTTCTCACCTCAGGATCTCCAGAACAACGGTCA
AAAATCATCCATGACCTCGTCAAAATTTGTCAAGAGTGGGGCTTCTTCATAGCCATTAACCATGGAGTCC
CTGAGAGCCTCATGAAGGCGATGATCGAAACATGCCATGGATTTTTCAGTTTACCAGACGAGGAGAAGAA
TGAGTTCAAATCGGGGAACGATGTGCTAGAGATGTTCAAGTATGGAACTAGCTATAATCTGGCATTGGAT
AAATTCCTTCTCTGGAGGGACTTCTTCAAGGTCAGAGTGCATCCCGAATTCTACTCCCTCTACAAGCCGG
CTTGCTTCAGTGAAGTCTCAATGGAGTTCAGCAAGAGAGCCCGAGAAGTTGCGTTGGAGATAACAAGAGC
AATCTCAGAAAGCTTGGGGTTGGAGCCAAACTACATACACAACGCCATGAACATGGATCGTGGCATACAA
ATGCTCGCCGCCAACTACTACCCGCCTTGCCCTCGGCCAGAACATGCAATTGGCATACCCCATCACACTG
ATCATGGCCTTGTCACCCTTCTCATCCAGAATGAGATGAAAGGCCTCCAAGTTGAGCACAACGGCAAATG
GCTCACCGTTGATGGCCCTCCCAACGGTTTCTTTGTTAACCTCGCTGACCAGATGCAGATTCTTACTAAT
GGTAAATACAAGAGCGTAATGCATCGAGCCACCGTGAACAACAAAGCTACAAGGATATCGATCGCCATAC
CGCATGGACCATCTGTAGATACAGTTGTTGCACCAGCTCCAGAGTTGTTGGAAAAGGAAGGCCAAGCTCC
TAAATACCTTGCCATGAACTACAAGGAATATATACAGCTTCAACAAAGTGGCAAGAACTATATGAAGTCT
ACCTTTGATCACATACGAGTCTAA
>XP_016650737.1_5-*Prunus_mume*

ATGGCTGAAGTTGCTGCTGCTCAAAGCACGGAGGCATTGAAAGTAACGAGCATCAAATCACTTGCTGAAT
CACCTGCTCTCAACTCTGTTCCTTCTGCATATGGCTTCACCATAAACCCCAACGATGAAGCAGATCCCAA
CGACCCTGAATTCGCAATTCCCATCGTCGACATGTCTCTTCTCACCTCAGGATCTCCAGAACAACGGTCA
AAAATCATCCATGACCTCGTCAAAATTTGTCAAGAGTGGGGCTTCTTCATAGCCATTAACCATGGAGTCC
CTGAGAGCCTCATGAAGGCGATGATCGAAACATGCCATGGATTTTTCAGTTTACCAGACGAGGAGAAGAA
TGAGTTCAAATCGGGGAACGATGTGCTAGAGATGTTCAAGTATGGAACTAGCTATAATCTGGCATTGGAT
AAATTCCTTCTCTGGAGGGACTTCTTCAAGGTCAGAGTGCATCCCGAATTCTACTCCCTCTACAAGCCGG
CTTGCTTCAGTGAAGTCTCAATGGAGTTCAGCAAGAGAGCCCGAGAAGTTGCGTTGGAGATAACAAGAGC
AATCTCAGAAAGCTTGGGGTTGGAGCCAAACTACATACACAACGCCATGAACATGGATCGTGGCATACAA
ATGCTCGCCGCCAACTACTACCCGCCTTGCCCTCGGCCAGAACATGCAATTGGCATACCCCATCACACTG
ATCATGGCCTTGTCACCCTTCTCATCCAGAATGAGATGAAAGGCCTCCAAGTTGAGCACAACGGCAAATG
GCTCACCGTTGATGGCCCTCCCAACGGTTTCTTTGTTAACCTCGCTGACCAGATGCAGATTCTTACTAAT
GGTAAATACAAGAGCGTAATGCATCGAGCCACCGTGAACAACAAAGCTACAAGGATATCGATCGCCATAC
CGCATGGACCATCTGTAGATACAGTTGTTGCACCAGCTCCAGAGTTGTTGGAAAAGGAAGGCCAAGCTCC
TAAATACCTTGCCATGAACTACAAGGAATATATACAGCTTCAACAAAGTGGCAAGAACTATATGAAGTCT
ACCTTTGATCACATACGAGTCTAA
>XP_015874283.1_1-*Ziziphus_jujuba*
ATGGAAGAGTTGGATTCAGCCTTCGTCCAAGACATAGAACATAGGCCCAACCTCAAATCCACTGAACCTG
TTGATGATGGAATACCTACCATTGATCTCTCAGCTTTGACCCTTGCTGACACCCAAAAACTTGCCTCGGA
GATTGGCCTTGCATGCCAAGAATGGGGATTTTTCCAAATAATCAATCATGGAGTACCTTTGGAATTGCAG
AGGAAAGTCGAGAAGGTGGCTAGGGAATTCTTTGAGCTACCATCCGAGGAGAAAAGGAAGGTGAAAAGGA
GCGAGGAGAACCGCTTGGGTTTCTATGACAGCGAGCACACTACAAATGTCAGAGATTGGAAGCAACTTTT
TGACTTCTTGGTTGAAGACAGGACTGTGATCCCAGCTTCACCTGAGCCTGATGATAAGGAGTTGATGACA
ATAACTAATCAGTGGCCTCTGTACCCTCCCGAATTGAGAGAGGTGTTCCAGGAGTATGCTAAAGAAATGA
AAAGCTGACTTTCAAGTTATTGGAACTCATTACGCTAAGCTTAGGCTTGCCAGAAAACAGGCTCAGCGG
CTACTTCAATGGCCAAACCAGCTTCGTCAGAATCAATTACTATCCTCCATGCCCTTTTCCTCACCTAGCT
CTAGGTGCTACTCGCCACAAGGATACTGGAGTCTTAACCATCCTTGCCCAAGATGAGGTTGGAGGATTGC
AAGCTAAGCGAAAGTCAGATGGAGAATGGATCCCAGTTAAACCCACCCCAAATGCCTATGTCGTCAATGT
CGGTGATGTTTTACAGGTTTGGAGCAATGAAAAATATCAAAGTGTGGAGCACAGGGTGGTGGTGAATACT
GTGAAGGAAAGGATGTCCATTCCATTCTTCTTCCATCCAGGTACCCATGTTATGGTGAAGCCCGTAGAGG
AGCTCTTGAACGGAGAAAATCGTCCCAAGTACAAGGAATACAACTGGGGCAAGTTCTTTGTCAACAGAAG
CCGCAGTGACTTCAAGAAACTTAAAGTGGAGAACATCCAAATTGAACATCTTAAGACTGTGAATCAAGAG
AAATAA
>XP_015874281.1_2-*Ziziphus_jujuba*
ATGGAAGAGTTGGATTCAGCCTTCGTCCAAGACATAGAACATAGGCCCAACCTCAAATCCACTGAACCTG
TTGATGATGGAATACCTACCATTGATCTCTCAGCTTTGACCCTTGCTGACACCCAAAAACTTGCCTCGGA
GATTGGCCTTGCATGCCAAGAATGGGGATTTTTCCAAGTAATCAATCATGGAGTACCTTTGGAATTGCAG
AGGAAAGTCGAGAAGGTGGCTAGGGAATTCTTTGAGCTACCATCCGAGGAGAAAAGGAAGGTGAAAAGGA
GCGAGGAGAACCGCTTGGGTTTCTATGACAGCGAGCACACTACAAATGTCAGAGATTGGAAGGAACTTTT
TGACTTCTTGGTTGAAGACAGGACTGTGATCCCAGCTTCACCTGAGCCTGATGATAAGGAGTTGATGACA
ATAACTAATCAGTGGCCTCTGTACCCTCCCGAATTGAGAGAGGTGTTCCAGGAGTATGCTAAAGAAATGA
AAAGCTGACTTTCAAGTTATTGGAACTCATTACGCTAAGCTTAGGCTTGCCAGAAAACAGGCTCAGCGG
CTACTTCAATGGCCAAACCAGCTTCGTCAGAATCAATTACTATCCTCCATGCCCTTTTCCTCACCTAGCT
CTAGGTGCTACTCGCCACAAGGATACTGGAGTCTTAACCATCCTTGCCCAAGATGAGGTTGGAGGATTGC
AAGCTAAGCGAAAGTCAGATGGAGAATGGATCCCAGTTAAACCCACCCCAAATGCCTATGTCGTCAATGT
CGGTGATGTTTTACAGGTTTGGAGCAATGAAAAATATCAAAGTGTGGAGCACAGGGTGGTGGTGAATACT
GTGAAGGAAAGGATGTCCATTCCATTCTTCTTCCATCCAGGTACCCATGTTATGGTGAAGCCCGTAGAGG
AGCTCTTGAACGGAGAAAATCGTCCCAAGTACAAGGAATACAACTGGGGCAAGTTCTTTGTCAACAGAAG
CCGCAGTGACTTCAAGAAACTTAAAGTGGAGAACATCCAAATTGAACATCTTAAGACTGTGAATCAAGAG
AAATAA
>XP_015874282.1_3-*Ziziphus_jujuba*
ATGGGAGAGTTGGATTCAGCCTTCATCCAAGACATAGAACATAGGCCCAACCTCAAATCCACTGAACCTG
TTGGTGATGGAATACCCACCATTGATCTCTCAGCTTTGACCATTGCTGACACCCAAAAACTTGCCTCGGA
GATTGGCCTTGCATGCCAAGAATGGGGATTTTTCCAAATAATCAATCATGGAGTACCTTTGAAATTGCAG

AGGAAAGTTGAGAAGGTGGCTAAGGAATTCTTTGAGCTACCATCCGAGGAGAAAAGGAAGGTGAAAAGGA
ACGAGGTGAACCCCTTGGGTTACTATGATTGCGAGCATACTAAAAATGTTAGAGATTGGAAGGAACTTTT
TGATTTTTTGGTGAAAGATGGGATTGTGATCCCAGCTTCACCTGAGCCTGATGATAAGGGGGTGAGGACA
ATAACTAATCAGTGGCCTCAGTACCCTCCGGAGTTTAGAGAGGTGTTCCAGGAGTATGCTAAACAAGTGG
AAAAGCTGGCTTTCAAGTTATTAGAACTCATTGCGCTAAGCTTAGGCTTGCCAGAAAACAGGTTCAGCGG
CTATTTCAATGGCCATACTACCTCCATCAGAATCAATTACTATCCTCCATGCCCTTTCCCTCACCTAGCT
CTAGGTGTTGGTCGCCACAAGGATCCTGGAGTCTTAACCATCCTTGCCCAGGATGATGTTGCAGGATTGC
AAGTTAAGCGAAAATCAGATGGAGAATGGATCACTGTTAAACCCACCCCAAATGCCTATATTGTCAATGT
TGGTGATGTTTTTCAGGTTTGGAGCAATGAAAAATATCAAAGTGTGGAGCACAGGGTGGTGGTGAATACT
GTGAAGGAAAGGATGTCCATTCCATTCTTCTTCCATCCAGGTACCCATGTTATGGTGAAGCCCGTAGAGG
AGCTCTTGAACGGAGAAAATCGTCCCAAGTACAAGGAATACAACTGGGGCAAGTTCTTTGTCAACAGAAG
CCGCAGTGACTTCAAGAAACTTAAAGTGGAGAACATCCAAATTGAACATCTTAAGACTGTGAATCAAGAG
AAATAA
>XP_024926749.1_4-*Ziziphus_jujuba*
ATGGAAGAGTTGGATTCAGCCTTCGTCCAAGACATAGAACATAGGCCCAACCTCAAATCCACTGAACCTG
TTGATGATGGAATACCTACCATTGATCTCTCAGCTTTGACCCTTGCTGACACCCAAAAACTTGCCTCGGA
GATTGGCCTTGCATGCCAAGAATGGGGATTTTTCCAAGTAATCAATCATGGAGTACCTTTGGAATTGCAG
AGGAAAGTCGAGAAGGTGGCTAGGGAATTCTTTGAGCTACCATCCGAGGAGAAAAGGAAGGTGAAAAGGA
GCGAGGAGAACCGCTTGGGTTTCTATGACAGCGAGCACACTACAAATGTCAGAGATTGGAAGGAACTTTT
TGACTTCTTGGTTGAAGACAGGACTGTGATCCCAGCTTCACCTGAGCCTGATGATAAGGAGTTGATGACA
ATAACTAATCAGTGGCCTCTGTACCCTCCCGAATTGAGAGAGGTGTTCCAGGAGTATGCTAAAGAAATGA
AAAAGCTGACTTTCAAGTTATTGGAACTCATTACGCTAAGCTTAGGCTTGCCAGAAAACAGGCTCAGCGG
CTACTTCAATGGCCAAACCAGCTTCGTCAGAATCAATTACTATCCTCCATGCCCTTTTCCTCACCTAGCT
CTAGGTGCTACTCGCCACAAGGATACTGGAGTCTTAACCATCCTTGCCCAAGATGAGGTTGGAGGATTGC
AAGCTAAGCGAAAGTCAGATGGAGAATGGATCCCAGTTAAACCCACCCCAAATGCCTATGTCGTCAATGT
CGGTGATGTTTTACAGGTTTGGAGCAATGAAAAATATCAAAGTGTGGAGCACAGGGTGGTGGTGAATACT
GTGAAGGAAAGGATGTCCATTCCATTCTTCTTCCATCCAGGTACCCATGTTATGGTGAAGCCCGTAGAGG
AGCTCTTGAATGGAGAAAATCGTCCCAAGTACAAGGAATACAACTGGGGCAAGTTCTTTGTCAACAGAAG
CCGCAGTGACTTCAAGAAACTTGAAGTGGAGAACGTCCAAATTGAACATCTTAGACTGTGA
>XP_015874285.1_5-*Ziziphus_jujuba*
ATGGGAGAGTTGGATTCAGCCTTCATCCAAGACATAGAACATAGGCCCAACCTCAAATCCACTGAACCTG
TTGGTGATGGAATACCCACCATTGATCTCTCAGCTTTGACCATTGCTGACACCCAAAAACTTGCCTCGGA
GATTGGCCTTGCATGCCAAGAATGGGGATTTTTCCAAATAATCAATCATGGAGTACCTTTGAAATTGCAG
AGGAAAGTTGAGAAGGTGGCTAAGGAATTCTTTGAGCTACCATCCGAGGAGAAAAGGAAGGTGAAAAGGA
ACGAGGTGAACCCCTTGGGTTACTATGATTGCGAGCATACTAAAAATGTTAGAGATTGGAAGGAACTTTT
TGATTTTTTGGTGAAAGATGGGATTGTGATCCCAGCTTCACCTGAGCCTGATGATAAGGGGGTGAGGACA
ATAACTAATCAGTGGCCTCAGTACCCTCCGGAGTTTAGAGAGGTGTTCCAGGAGTATGCTAAAGAAGTGG
AAAAGCTGGCTTTCAAGTTATTAGAACTCATTGCGCTAAGCTTAGGCTTGCCAGAAAACAGGTTCAGCGG
CTATTTCAATGGCCATACTACCTCCATCAGAATCAATTACTATCCTCCATGCCCTTTCCCTCACCTAGCT
CTAGGTGTTGGTCGCCACAAGGATCCTGGAGTCTTAACCATCCTTGCCCAGGATGATGTTGCAGGATTGC
AAGTTAAGCGAAAATCAGATGGAGAATGGATCACTGTTAAACCCACCCCAAATGCCTATATTGTCAATGT
TGGTGATGTTTTTCAGGTTTGGAGCAATGAAAAATATCAAAGTGTGGAGCACAGGGTGGTGGTGAATACT
GTGAAGGAAAGGATGTCCATTCCATTCTTCTTCCATCCAGGTACCCATGTTATGGTGAAGCCCGTAGAGG
AGCTCTTGAATGGAGAAAATCGTCCCAAGTACAAGGAATACAACTGGGGCAAGTTCTTTGTCAACAGAAG
CCGCAGTGACTTCAAGAAACTTGAAGTGGAGAACGTCCAAATTGAACATCTTAGACTGTGA
>XP_015874284.1_6-*Ziziphus_jujuba*
ATGGGAGAGTTGGATTCAGCCTTCATCCAAGACATAGAACATAGGCCCAACCTCAAATCCACTGAACCTG
TTGGTGATGGAATACCCACCATTGATCTCTCAGCTTTGACCATTGCTGACACCCAAAAACTTGCCTCGGA
GATTGGCCTTGCATGCCAAGAATGGGGATTTTTCCAAATAATCAATCATGGAGTACCTTTGAAATTGCAG
AGGAAAGTTGAGAAGGTGGCTAAGGAATTCTTTGAGCTACCATCCGAGGAGAAAAGGAAGGTGAAAAGGA
ACGAGGTGAACCCCTTGGGTTACTATGATTGCGAGCATACTAAAAATGTTAGAGATTGGAAGGAACTTTT
TGATTTTTTGGTGAAAGATGGGATTGTGATCCCAGCTTCACCTGAGCCTGATGATAAGGGGGTGAGGACA
ATAACTAATCAGTGGCCTCAGTACCCTCCGGAGTTTAGAGAGGTGTTCCAGGAGTATGCTAAACAAGTGG
AAAAGCTGGCTTTCAAGTTATTAGAACTCATTGCGCTAAGCTTAGGCTTGCCAGAAAACAGGTTCAGCGG

CTATTTCAATGGCCATACTACCTCCATCAGAATCAATTACTATCCTCCATGCCCTTTCCCTCACCTAGCT
CTAGGTGTTGGTCGCCACAAGGATCCTGGAGTCTTAACCATCCTTGCCCAGGATGATGTTGCAGGATTGC
AAGTTAAGCGAAAATCAGATGGAGAATGGATCACTGTTAAACCCACCCCAAATGCCTATATTGTCAATGT
TGGTGATGTTTTTCAGGTTTGGAGCAATGAAAAATATCAAAGTGTGGAGCACAGGGTGGTGGTGAATACT
GTGAAGGAAAGGATGTCCATTCCATTCTTCTTCCATCCAGGTACCCATGTTATGGTGAAGCCCGTAGAGG
AGCTCTTGAATGGAGAAAATCGTCCCAAGTACAAGGAATACAACTGGGGCAAGTTCTTTGTCAACAGAAG
CCGCAGTGACTTCAAGAAACTTGAAGTGGAGAACGTCCAAATTGAACATCTTAGACTGTGA
>XP_028961244.1_1-*Malus_domestica*
ATGTTACCTGAAATGGGTTCAAATAGTACTGCTGATGGTACATACAATATCGAAAATGAAGAAACCGAAA
TCCAATACCTCCAGAAGGGAGTGAGACATTTATGTGAAAGAGGGCTGACTAGGGTTCCAAGCAAGTACAT
ATTGCCCGTCCAAGAACGACCCGACTTGGGAGACACTGGGAATGCTAGCAAGCATAACCTCAAGTTGCCA
GTTATTGATTTTACTCAGTTGCAAGGGTCCAACAGATCCCAAGTAATAGAGTCCCTTGCAAACGCTTGCG
AAGAATTCGGGTTTTTTCAGTTGATAAACCATGGTATCAAAGATGATGTTATTCTTGAAATTATTGATGT
AAGCAGAAGATTTTTCGAGCTCCCTTTCAATGAGAGATCAAAGTACATGTCAAAGGATATTTCTTCCCCA
GCTAGGTATGGAACAAGCTTTAACCAGAACAATGATAAAGTATTTTGCTGGAGAGACTTTCTGAAACTTG
GCTGCCATCCTTTACAAGACACTGTGTCCTGTTGGCCTTCTTCTCCAGCGGACTTAAGGGAAGCAGTGGT
AAACTACTCGAAGAGCACCAAGTTTTTGTATCTAATGCTAATGGAGGCCATCATGGAGAGCCTAGGATTG
GTGGAAGCTACAAAAGGCGGTGAGGGTAAAGGCAGTTTGGAGGATTTTGAAGATGGAAGCCAACTCATTG
TGGCGAATTGCTACCCTGCGTGCCCTGAACCTGATTTAACACTAGGTATGCCACCCCATTCGGACTATGG
CCTCCTCACTCTTCTACTTCAAGATGAAGTTGGGGGTCTTCAAATACAGAATCAAGGAAGTTGGGTAACC
GTGGAACCACTTCCAAATTCTTTCGTTGTCAATGTTGGTGATCATCTTGAGACAGGACAAAGGCTCGAGG
GTGATTTTATATCAAGGAAGGAGTGA
>XP_008347068.2_2-*Malus_domestica*
ATGTTACCTGAAATGGGTTCAAATAGTACTGCTGATGGTACATACAATATCGAAAATGAAGAAACCGAAA
TCCAATACCTCCAGAAGGGAGTGAGACATTTATGTGAAAGAGGGCTGACTAGGGTTCCAAGCAAGTACAT
ATTGCCCGTCCAAGAACGACCCGACTTGGGAGACACTGGGAATGCTAGCAAGCATAACCTCAAGTTGCCA
GTTATTGATTTTACTCAGTTGCAAGGGTCCAACAGATCCCAAGTAATAGAGTCCCTTGCAAACGCTTGCG
AAGAATTCGGGTTTTTTCAGTTGATAAACCATGGTATCAAAGATGATGTTATTCTTGAAATTATTGATGT
AAGCAGAAGATTTTTCGAGCTCCCTTTCAATGAGAGATCAAAGTACATGTCAAAGGATATTTCTTCCCCA
GCTAGGTATGGAACAAGCTTTAACCAGAACAATGATAAAGTATTTTGCTGGAGAGACTTTCTGAAACTTG
GCTGCCATCCTTTACAAGACACTGTGTCCTGTTGGCCTTCTTCTCCAGCGGACTTAAGGGAAGCAGTGGT
AAACTACTCGAAGAGCACCAAGTTTTTGTATCTAATGCTAATGGAGGCCATCATGGAGAGCCTAGGATTG
GTGGAAGCTACAAAAGGCGGTGAGGGTAAAGGCAGTTTGGAGGATTTTGAAGATGGAAGCCAACTCATTG
TGGCGAATTGCTACCCTGCGTGCCCTGAACCTGATTTAACACTAGGTATGCCACCCCATTCGGACTATGG
CCTCCTCACTCTTCTACTTCAAGATGAAGTTGGGGGTCTTCAAATACAGAATCAAGGAAGTTGGGTAACC
GTGGAACCACTTCCAAATTCTTTCGTTGTCAATGTTGGTGATCATCTTGAGATATTTAGCAATGGGAGAT
ACAAGAGCGTGGTCCATAGGGTTCTGGTCAACTCTTGCAAGTCTCGAATATCTGTAGCTTCATTGCACAG
CCTGCCATTCAAGAGCACAGTTCGGCCATCACCTAAACTCATTGACGAAGCAAACCCTACACGTTACAAG
GATACAGACTTCGCCAGTTTCCTCCAATACATTTCTAATTCTTCCCATGACGAAATGGACGCCAAGACTT
TCCTACAGTCCAGGAAATTGACTTGA
>XP_014633209.1_1-*Glycine_max*
ATGGCCGCCACAAAACCATTGTTAACCGACCTAGCCTCCACCATTGATCGCGTTCCCTCTAACTTCATCA
GGCCCATTGGTGACCGTCCAAAACTTCATCAACTTCACTCCTCCCTTGCTTCTATTCCCATCATCGACCT
TCAAGGCCTTGGTGGCTCCAATCATTCCCAAATCATCCAAAACATTGCGCATGCTTGCCAAACTTATGGC
TTCTTTCAAATTGTGAACCATGGGATTCAGGAGGAGGTGGTGAGCAAGATGGTGAATGTGTCAAAAGAGT
TCTTTGGTTTGCCGGAGAGTGAGAGGCTGAAGAATTTCTCTGATGACCCATCCAAGACAACAAGACTCTC
CACCAGTTTCAATGTCAAGACTGAGAAAGTTTCCAACTGGAGAGACTTCTTGAGACTTCACTGTCATCCC
CTTGAGGATTACATTCAGGAATGGCCTGGCAACCCTCCATCTTTCAGGGAAGATGTGGCGGAGTATAGTA
GAAAGATGAGAGGGTTATCACTGAAATTGCTTGAGGCAATCTCAGAGAGTTTGGGTTTGGAAAGGGATTA
CATAGACAAAGCACTGGGGAAACATGGGCAGCACCTGGCCATAAACTACTACCCTCCATGTCCTGAGCCA
GAGTTAACGTATGGTTTGCCAGCTCATGCTGACCCAAATGCAATTACTATTCTGCTCCAAAATGAGGTCC
CAGGCTTGCAAGTCCTCTATGATGGCAAGTGGCTAACCGTCAATCCTGTCCCTAACACCTTCATTGTCAA
TATTGGTGACCAAATTCAGCTTACAGGCGTACATCATTCATCATCTAGACTTTTTCATACTACATTCACA
TGCCACAAATTATTTGTCACGAGTGGAAAAACATTGACTACGTTGAAGCAGAACTACTAG

>XP_003528757.1_2-*Glycine_max*
ATGGCCGCCACAAAACCATTGTTAACCGACCTAGCCTCCACCATTGATCGCGTTCCCTCTAACTTCATCA
GGCCCATTGGTGACCGTCCAAAACTTCATCAACTTCACTCCTCCCTTGCTTCTATTCCCATCATCGACCT
TCAAGGCCTTGGTGGCTCCAATCATTCCCAAATCATCCAAAACATTGCGCATGCTTGCCAAACTTATGGC
TTCTTTCAAATTGTGAACCATGGGATTCAGGAGGAGGTGGTGAGCAAGATGGTGAATGTGTCAAAAGAGT
TCTTTGGTTTGCCGGAGAGTGAGAGGCTGAAGAATTTCTCTGATGACCCATCCAAGACAACAAGACTCTC
CACCAGTTTCAATGTCAAGACTGAGAAAGTTTCCAACTGGAGAGACTTCTTGAGACTTCACTGTCATCCC
CTTGAGGATTACATTCAGGAATGGCCTGGCAACCCTCCATCTTTCAGGGAAGATGTGGCGGAGTATAGTA
GAAAGATGAGAGGGTTATCACTGAAATTGCTTGAGGCAATCTCAGAGAGTTTGGGTTTGGAAAGGGATTA
CATAGACAAAGCACTGGGGAAACATGGGCAGCACCTGGCCATAAACTACTACCCTCCATGTCCTGAGCCA
GAGTTAACGTATGGTTTGCCAGCTCATGCTGACCCAAATGCAATTACTATTCTGCTCCAAAATGAGGTCC
CAGGCTTGCAAGTCCTCTATGATGGCAAGTGGCTAACCGTCAATCCTGTCCCTAACACCTTCATTGTCAA
TATTGGTGACCAAATTCAGGTGATAAGCAACGATAGGTACAAGAGTGTGTTGCATCGAGCATTGGTGAAT
TGTGAGAAGGAGAGAATGTCCATTCCAACATTTTACTGTCCTTCACCAGATGCATTGATAAAACCCGCAC
CCAAACTCGTAGACAATGAACATCCTGCGCAGTACACAAACTTCACATACAGAGAATACTATGACAAGTT
CTGGAACAGAGGGCTTTCAAAAGAAACCTGCGTCGACATGTTCAAGGCTCAAGACTAA
>XP_006583203.1_3-*Glycine_max*
ATGGCCGCCACAAAACCATTGTTAACCGACCTAGCCTCCACCATTGATCGCGTTCCCTCTAACTTCATCA
GGCCCATTGGTGACCGTCCAAAACTTCATCAACTTCACTCCTCCCTTGCTTCTATTCCCATCATCGACCT
TCAAGGCCTTGGTGGCTCCAATCATTCCCAAATCATCCAAAACATTGCGCATGCTTGCCAAACTTATGGC
TTCTTTCAAGAGGAGGTGGTGAGCAAGATGGTGAATGTGTCAAAAGAGTTCTTTGGTTTGCCGGAGAGTG
AGAGGCTGAAGAATTTCTCTGATGACCCATCCAAGACAACAAGACTCTCCACCAGTTTCAATGTCAAGAC
TGAGAAAGTTTCCAACTGGAGAGACTTCTTGAGACTTCACTGTCATCCCCTTGAGGATTACATTCAGGAA
TGGCCTGGCAACCCTCCATCTTTCAGGGAAGATGTGGCGGAGTATAGTAGAAAGATGAGAGGGTTATCAC
TGAAATTGCTTGAGGCAATCTCAGAGAGTTTGGGTTTGGAAAGGGATTACATAGACAAAGCACTGGGGAA
ACATGGGCAGCACCTGGCCATAAACTACTACCCTCCATGTCCTGAGCCAGAGTTAACGTATGGTTTGCCA
GCTCATGCTGACCCAAATGCAATTACTATTCTGCTCCAAAATGAGGTCCCAGGCTTGCAAGTCCTCTATG
ATGGCAAGTGGCTAACCGTCAATCCTGTCCCTAACACCTTCATTGTCAATATTGGTGACCAAATTCAGGT
GATAAGCAACGATAGGTACAAGAGTGTGTTGCATCGAGCATTGGTGAATTGTGAGAAGGAGAGAATGTCC
ATTCCAACATTTTACTGTCCTTCACCAGATGCATTGATAAAACCCGCACCCAAACTCGTAGACAATGAAC
ATCCTGCGCAGTACACAAACTTCACATACAGAGAATACTATGACAAGTTCTGGAACAGAGGGCTTTCAAA
AGAAACCTGCGTCGACATGTTCAAGGCTCAAGACTAA
>XP_014633210.1_4-*Glycine_max*
ATGGCCGCCACAAAACCATTGTTAACCGACCTAGCCTCCACCATTGATCGCGTTCCCTCTAACTTCATCA
GGCCCATTGGTGACCGTCCAAAACTTCATCAACTTCACTCCTCCCTTGCTTCTATTCCCATCATCGACCT
TCAAGGCCTTGGTGGCTCCAATCATTCCCAAATCATCCAAAACATTGCGCATGCTTGCCAAACTTATGGC
TTCTTTCAAATTGTGAACCATGGGATTCAGGAGGAGGTGGTGAGCAAGATGGTGAATGTGTCAAAAGAGT
TCTTTGGTTTGCCGGAGAGTGAGAGGCTGAAGAATTTCTCTGATGACCCATCCAAGACAACAAGACTCTC
CACCAGTTTCAATGTCAAGACTGAGAAAGTTTCCAACTGGAGAGACTTCTTGAGACTTCACTGTCATCCC
CTTGAGGATTACATTCAGGAATGGCCTGGCAACCCTCCATCTTTCAGGGAAGATGTGGCGGAGTATAGTA
GAAAGATGAGAGGGTTATCACTGAAATTGCTTGAGGCAATCTCAGAGAGTTTGGGTTTGGAAAGGGATTA
CATAGACAAAGCACTGGGGAAACATGGGCAGCACCTGGCCATAAACTACTACCCTCCATGTCCTGAGCCA
GAGTTAACGTATGGTTTGCCAGCTCATGCTGACCCAAATGCAATTACTATTCTGCTCCAAAATGAGGTCC
CAGGCTTGCAAGTCCTCTATGATGGCAAGTGGCTAACCGTCAATCCTGTCCCTAACACCTTCATTGTCAA
TATTGGTGACCAAATTCAGTAG
>XP_028236103.1_1-*Glycine_soja*
ATGGCCTCTGCAGCTTCCATTTCCTCATCACAACCAGAAGCACCAAAGGTTCATGCATCTGATATTTCTA
GCATCAAAGCCTTTGCAGAATCAAAGGGAGCCTCTCTGATCCCTTACACCTACCACTCCATCACAGAGCA
CCATGATGATGATGTAGCAGATGAGCTTGCTGCTTCCATCCCAGTCATCGACTTGTCTCTCCTCACTTCG
CATGACCCTCAAATCCACGCCAAAGCCGTGCACCAACTCGGCAAGGCCTGTGCAGAGTGGAGTTTCTTCA
TGCTCACAAATCATGGAATTCCCGAGAGCCTGGTGGAGGAGTTGATGAAAAAGTCTCGCGAGTTTCATGA
TTTGCCGATGGAAGAAAAGAAGGAGTTTGGTAATAAAGGACCCTTTGAACCGATCAGGCATGGCACCAGC
TTCTGTCCAGAAGCAGAGAATGTTCATTATTGGAGAGATTATCTCAAGGCCATCACGTTTCCTGAATTCA
ACTTCCCCTACAAGCCACCAGGTTACAGGGAAGTTGCTTATGATTACAGCAAAAAAATCAGAGGTGTGAC

AAGGAAGTTGCTTGAAGGAATATCAGAGAGTTTGGGATTAGAATCCAATTCCATAATCGAGTCCACTGAT
TTTGACTCTGGCCATCAGCTGTTTGTTGTGAACCTGTATCCACCATGTCCACAACCGCACCTTGCTCTGG
GGCTGCCTTCTCATTCTGACGTAGGTTTATTGACCTTGCTCACGCAGAATGGAATTGGAGGGCTTCAGGT
TAAGCACAATGGCAAATGGGTCAATGTCAATCCCCTTCCCAACTGCCTAATTGTTTTGCTTTCAGATCAA
CTCGAGGTTGTGAGTAATGGGAGGTATGAGAGCATGTTACACCTAGTGATCCTCAACAACAAGGAAATCA
TGATGAGTCTTGTGGTGGTTGAAAAATTCATTTTCTGA
>XP_028236102.1_2-*Glycine_soja*
ATGGCCTCTGCAGCTTCCATTTCCTCATCACAACCAGAAGCACCAAAGGTTCATGCATCTGATATTTCTA
GCATCAAAGCCTTTGCAGAATCAAAGGGAGCCTCTCTGATCCCTTACACCTACCACTCCATCACAGAGCA
CCATGATGATGATGTAGCAGATGAGCTTGCTGCTTCCATCCCAGTCATCGACTTGTCTCTCCTCACTTCG
CATGACCCTCAAATCCACGCCAAAGCCGTGCACCAACTCGGCAAGGCCTGTGCAGAGTGGAGTTTCTTCA
TGCTCACAAATCATGGAATTCCCGAGAGCCTGGTGGAGGAGTTGATGAAAAAGTCTCGCGAGTTTCATGA
TTTGCCGATGGAAGAAAAGAAGGAGTTTGGTAATAAAGGACCCTTTGAACCGATCAGGCATGGCACCAGC
TTCTGTCCAGAAGCAGAGAATGTTCATTATTGGAGAGATTATCTCAAGGCCATCACGTTTCCTGAATTCA
ACTTCCCCTACAAGCCACCAGGTTACAGGGAAGTTGCTTATGATTACAGCAAAAAAATCAGAGGTGTGAC
AAGGAAGTTGCTTGAAGGAATATCAGAGAGTTTGGGATTAGAATCCAATTCCATAATCGAGTCCACTGAT
TTTGACTCTGGCCATCAGCTGTTTGTTGTGAACCTGTATCCACCATGTCCACAACCGCACCTTGCTCTGG
GGCTGCCTTCTCATTCTGACGTAGGTTTATTGACCTTGCTCACGCAGAATGGAATTGGAGGGCTTCAGGT
TAAGCACAATGGCAAATGGGTCAATGTCAATCCCCTTCCCAACTGCCTAATTGTTTTGCTTTCAGATCAA
CTCGAGGTAGTGAGTAATGGGAAGTACGCTAGGGTGATGCACCGAGCAATACTCAACAATGCAGACACTA
GGATCAGTGTTGTGCTGGCGAATGGACCTGCGCTAGATAAGGAAATTGGACCTTTGCCGGAGCTACTGCA
GAATTATAAGCCATTGTTCAGAAGCATCAAATACCGAGATTACTTCCAAATTCAGCAGAAGTCACGATTA
CAAGATAAAAGTAGCTTGGATGAGATTCGCCTTAACTAA
>XP_028101998.1_1-*Camellia_sinensis*
ATGGGAGAGGTAGATCCAGCTTTCATTCAGGCACCTGAACACAGGCCTAAACTTTCCACCATCGAAGCTC
AAGGCATCCCATTAATTGATCTCTCCATTTTGAACTCTCATGAGTCTTCTAACCTTAGTTTAATACAACT
TCTCAAGGCCGGTGCTGAGGGAAGACAGAGAGTCAATCTAGTGGAAGAGATAGGGGAAGCATGCAAGAAC
TGGGGGTTCTTCCAAGTGATCAACCATGGAGTCCCTTTGGAAACTCGTGAGAAGATTGAGTCGGCGGCGA
GAAAGTTCTTCGCACAGTCAGCAGATGAGAAGAGGAAGATTAGGAGGAGAGATGAAATGAACCCACAAGG
TTACAATGATATTGAGCTGACTAAGAATGTTAGAGACTGGAAAGAAGTGTTTGATTTGGCTGTCAATAAC
CCGACTGTCATTCCGGCGTCACACGAGCCCGATGATAAGGAGGTCAGGGAGTTGGTTAACCGGTGGCCAG
CGTATCCTCCTGAGTTAAGGGAATCATTTGAAGAATATGCCACGGAAATGGAGAGACTGTCACACAAGCT
GTTGGGACTCATCTCCCTGAGCTTAGGCTTGCCAGAAAACCGGTTGAGCGGCTTCTTCAATGACCATATC
AGCTTTATCCGACTCAATCACTACCCACCGTGCCCAATCCCTCACCTAGCTCTAGGCGTTGGTCGACACA
AGGATGGTGGTGCCTTGACTGTCCTTGCACAGGATGATGTTGGAGGACTGGAAGTGAAGCGAAAAACAGA
CGGAGAGTGGGTTCGAGTTAAGCCCACTCCAGATGCTTATATTGTGAATGTTGGTGACATTGTTCAGGTA
TGGAGCAATGAGAAGTATGAGAGTGTGGAGCATAGGGTGACGGTGAACTCTGAGAGAGAAAGATTCTCGA
TCCCATTCTTCTTCAATCCGGACCATCATGTCATGGTGGAGCCTTTAGAGGAGCTGACAACTGAGCAAAA
CCCTCCCAAGTACAGGGCTTACAACTGGGGGAAATTTTTCGCTACCAGAAGGCGCAGTAATTTCAAGAAG
CTTGATGTTGAGAACATCCAAATTTCTCATTTCAAAATATAA
>XP_028101999.1_2-*Camellia_sinensis*
ATGGGAGAGGTAGATCCAGCTTTCATTCAGGCACCTGAACACAGGCCTAAACTTTCCACCATCGAAGCTC
AAGGCATCCCATTAATTGATCTCTCCATTTTGAACTCTCATGAGTCTTCTAACCTTAGTTTAATACAACT
TCTCAAGGCCGGTGCTGAGGGAAGACAGAGAGTCAATCTAGTGGAAGAGATAGGGGAAGCATGCAAGAAC
TGGGGGTTCTTCCAAGTGATCAACCATGGAGTCCCTTTGGAAACTCGTGAGAAGATTGAGTCGGCGGCGA
GAAAGTTCTTCGCACAGTCAGCAGATGAGAAGAGGAAGATTAGGAGGAGAGATGAAATGAACCCACAAGG
TTACAATGATATTGAGCTGACTAAGAATGTTAGAGACTGGAAAGAAGTGTTTGATTTGGCTGTCAATAAC
CCGACTGTCATTCCGGCGTCACACGAGCCCGATGATAAGGAGGTCAGGGAGTTGGTTAACCGGTGGCCAG
CGTATCCTCCTGAGTTAAGGGAATCATTTGAAGAATATGCCACGGAAATGGAGAGACTGTCACACAAGCT
GTTGGGACTCATCTCCCTGAGCTTAGGCTTGCCAGAAAACCGGTTGAGCGGCTTCTTCAATGACCATATC
AGCTTTATCCGACTCAATCACTACCCACCGTGCCCAATCCCTCACCTAGCTCTAGGCGTTGGTCGACACA
AGGATGGTGGTGCCTTGACTGTCCTTGCACAGGATGATGTTGGAGGACTGGAAGTGAAGCGAAAAACAGA
CGGAGAGTGGGTTCGAGTTAAGCCCACTCCAGATGCTTATATTGTGAATGTTGGTGACATTGTTCAGGTA
TGGAGCAATGAGCAGTATGAGAGTGTGGAGCATAGGGTGATGGTGAACTCTGAGAGAGAGAGATTCTCGA

TCCCATTCTTCTTCAATCCGAACCATCGTGTCATGGTAGAGGCTCTAGAGGAGCTGACAACTGATCAAAA
CCCTGCCAAGTACAGGGCTTACAATTGGGGAAAATTTTTTGCTACCAGAAAGCGCGGTAATTTCAAGAAG
CTTGATGTTGAGAACATCCAAATTTCTCATTTCAAGATATAA
>XP_028102000.1_3-*Camellia_sinensis*
ATGGGAGAGGTAGATCCAGCTTTCATTCAAGCACTAGAGCACAGACCTAAACTTTCCACCATTGAAGCTC
AAGGCATCCCAGTAATTGACCTCTCCATTTTCGACTCTAATGAGTCCTCTAACCTTAGTTCCATACAACC
TCTCAAGGCTGGTGTCGAGGGAAGACAAAGAGTCAATCTAGTGGAAGAGATAGGAGAAGCATGCAAGAAT
TGGGGGTTCTTCCAAGTGATCAACCATGGAGTCCCTTTGGAAACTCGTGAGAAGTTTGAGTCAGCAGCAA
GAAAGTTCTTTGCACAGTCAACAGAGGAGAAGAGGAAGGTTAGGAGAGATGAGGTGAACCCGCAAGGGTA
CTATGATACTGAACTGACTAAGAATGTTAGGGACTGGAAAGAAGTGTTTGATCTGACTGTCAAGAATCCA
GCTGTGATTCCGGCATCGTACCAGCCCGGTGATAGGGAGGTCAGGGAGTCGGTTAACCGGTGGCCAGAGT
ATCCTCCCGAGTTTAGAGAGGCATTTGAAGAATATGCCACAGAAATGGAGAAACTGTCACACAAGCTGTT
GGGACTCATCTCCCTGAGCTTAGGCTTGCCAGAAAACCGGTTGAGCGGCTTCTTCAATGACCATATCAGC
TTTATCCGACTCAATCACTACCCGCCGTGCCCAATCCCTCACCAAGCTCTAGGCGTTGGTCGACACAAGG
ATGGCGGTGCCTTGACTGTCCTTGCACAGGATGAAGTTGGAGGTCTGGAAGTGAAGCGAAAAACAGACGA
AGAGTGGGTTCGAGTTAAGCCCACTCCAGATGCTTATATTGTGAATGTTGGTGACATTGTTCAGGTATGG
AGCAATGAGAAGTATGAGAGTGTGGAGCATAGGGTGACGGTGAACTCTGAGAGAGAAAGATTCTCGATCC
CATTCTTCTTCAATCCGGACCATCATGTCATGGTGGAGCCTTTAGAGGAGCTGACAACTGAGCAAAACCC
TCCCAAGTACAGGGCTTACAACTGGGGGAAATTTTTCGCTACCAGAAGGCGCAGTAATTTCAAGAAGCTT
GATGTTGAGAACATCCAAATTTCTCATTTCAAAATATAA
>XP_028102001.1_4-*Camellia_sinensis*
ATGGGAGAGGTAGATCCAGCTTTCATTCAAGCACTAGAGCACAGACCTAAACTTTCCACCATTGAAGCTC
AAGGCATCCCAGTAATTGACCTCTCCATTTTCGACTCTAATGAGTCCTCTAACCTTAGTTCCATACAACC
TCTCAAGGCTGGTGTCGAGGGAAGACAAAGAGTCAATCTAGTGGAAGAGATAGGAGAAGCATGCAAGAAT
TGGGGGTTCTTCCAAGTGATCAACCATGGAGTCCCTTTGGAAACTCGTGAGAAGTTTGAGTCAGCAGCAA
GAAAGTTCTTTGCACAGTCAACAGAGGAGAAGAGGAAGGTTAGGAGAGATGAGGTGAACCCGCAAGGGTA
CTATGATACTGAACTGACTAAGAATGTTAGGGACTGGAAAGAAGTGTTTGATCTGACTGTCAAGAATCCA
GCTGTGATTCCGGCATCGTACCAGCCCGGTGATAGGGAGGTCAGGGAGTCGGTTAACCGGTGGCCAGAAG
AGGCATTTGAAGAATATGCCACAGAAATGGAGAAACTGTCACACAAGCTGTTGGGACTCATCTCCCTGAG
CTTAGGCTTGCCAGAAAACCGGTTGAGCGGCTTCTTCAATGACCATATCAGCTTTATCCGACTCAATCAC
TACCCGCCGTGCCCAATCCCTCACCAAGCTCTAGGCGTTGGTCGACACAAGGATGGCGGTGCCTTGACTG
TCCTTGCACAGGATGAAGTTGGAGGTCTGGAAGTGAAGCGAAAAACAGACGAAGAGTGGGTTCGAGTTAA
GCCCACTCCAGATGCTTATATTGTGAATGTTGGTGACATTGTTCAGGTATGGAGCAATGAGAAGTATGAG
AGTGTGGAGCATAGGGTGACGGTGAACTCTGAGAGAGAAAGATTCTCGATCCCATTCTTCTTCAATCCGG
ACCATCATGTCATGGTGGAGCCTTTAGAGGAGCTGACAACTGAGCAAAACCCTCCCAAGTACAGGGCTTA
CAACTGGGGGAAATTTTTCGCTACCAGAAGGCGCAGTAATTTCAAGAAGCTTGATGTTGAGAACATCCAA
ATTTCTCATTTCAAAATATAA
>XP_006491758.2_1-*Citrus_sinensis*
ATGTTTGTATGCATATTTGTTATATATATACAGTCGTATTGCATGCAGGTACCCATCGTCATGTTTCCTG
CAATAATGGCTATTACAACTGATGAAACAAAGGCAAATGATGATGATCAAGTATACCAGAAAGGAGTGAA
GAATCTTTGCGAAAACGGCGTGACGAAAGTCCCTAAAAAGTACATATTGCCTGAATTCGATCGAGCAGAT
ATAGTACTACAAGTAGATTTGACCATGCCAAATCTTAAGCTGCCTGTTATTGATTTTTCCGAATTGCAAG
GCCCAAATCGTTCCGAGGTCCTAAAATCTCTAACTAGTGCTTGTGAAAAATATGGGTTTTTCCAGGTAGT
GAATCACGGTATCCCGAACAATGTTATTAGCAGCATGATTGATGTGAGTGCAAGGTTTTTTGAACTCCCA
ATTGAGGTAAGATCCAAGTACGTGTCATCAGATCTCACAGCACCAGTTCGATATGGAACTAGCTTTAATC
AGAATAAGGATAGGGTCTTCTGTTGGAGAGACTTCTTGAAGCTCGTATGCCACCCCTCACAAGATGTCCT
TGTTCATTGGCCTTCTTATCCAACGGACATGAGGAGATTGGCGGTTACCTACGCAAAAGAAATCAAAAAC
TTGTTCGTAATGCTAATGGAGGCTATCTTAGAGAGCTTGGGGCTGTGGGGAGCCACCATGAATAAGACTG
AAGAAGTTGAAAAACTAATAAAGCAATTCCAAGATGGGAGCCAGCAAATGGTGGTCAATTGCTACCCTCC
ATGTCCTGAGCCTCATCTAACCCTAGGAATGCCTCCACATTCAGATTATGGATTTCTTACTCTTCTTCTA
CAAGATGACGTTGAGGGTTTGCAAATTCAATATAAAGAAAAGTGGGTCGCAGTTCAACCTTTAGCCAGCT
CCTTTGTGGTTAATGTTGGTGATCACCTTGAGATATTCAGCAATGGCAAGTACAGGAGTGTGTTACATAG
GGTTCTTGTCAATCCAGCCAAGTCTCGCATTTCTGTTGCTTCTTTGCATTCTCTGCCTTTCAACTCAATG
GTTAGCCCATCGCCCTCACTCATCAATAAAGCAAATCCAAGGCGCTATAAGGAGACCGATTTTGCCTGTT

TCCTTGAATACATTTCATCTTTTGAGCCTAAGAGGAAGAATTTCCTTGAGTCAAGGAAATTACTATGA
>XP_024948532.1_2-*Citrus_sinensis*
ATGCAGGTACCCATCGTCATGTTTCCTGCAATAATGGCTATTACAACTGATGAAACAAAGGCAAATGATG
ATGATCAAGTATACCAGAAAGGAGTGAAGAATCTTTGCGAAAACGGCGTGACGAAAGTCCCTAAAAAGTA
CATATTGCCTGAATTCGATCGAGCAGATATAGTACTACAAGTAGATTTGACCATGCCAAATCTTAAGCTG
CCTGTTATTGATTTTTCCGAATTGCAAGGCCCAAATCGTTCCGAGGTCCTAAAATCTCTAACTAGTGCTT
GTGAAAAATATGGGTTTTTCCAGGTAGTGAATCACGGTATCCCGAACAATGTTATTAGCAGCATGATTGA
TGTGAGTGCAAGGTTTTTTGAACTCCCAATTGAGGTAAGATCCAAGTACGTGTCATCAGATCTCACAGCA
CCAGTTCGATATGGAACTAGCTTTAATCAGAATAAGGATAGGGTCTTCTGTTGGAGAGACTTCTTGAAGC
TCGTATGCCACCCCTCACAAGATGTCCTTGTTCATTGGCCTTCTTATCCAACGGACATGAGGAGATTGGC
GGTTACCTACGCAAAAGAAATCAAAAACTTGTTCGTAATGCTAATGGAGGCTATCTTAGAGAGCTTGGGG
CTGTGGGGAGCCACCATGAATAAGACTGAAGAAGTTGAAAAACTAATAAAGCAATTCCAAGATGGGAGCC
AGCAAATGGTGGTCAATTGCTACCCTCCATGTCCTGAGCCTCATCTAACCCTAGGAATGCCTCCACATTC
AGATTATGGATTTCTTACTCTTCTTCTACAAGATGACGTTGAGGGTTTGCAAATTCAATATAAAGAAAAG
TGGGTCGCAGTTCAACCTTTAGCCAGCTCCTTTGTGGTTAATGTTGGTGATCACCTTGAGATATTCAGCA
ATGGCAAGTACAGGAGTGTGTTACATAGGGTTCTTGTCAATCCAGCCAAGTCTCGCATTTCTGTTGCTTC
TTTGCATTCTCTGCCTTTCAACTCAATGGTTAGCCCATCGCCCTCACTCATCAATAAAGCAAATCCAAGG
CGCTATAAGGAGACCGATTTTGCCTGTTTCCTTGAATACATTTCATCTTTTGAGCCTAAGAGGAAGAATT
TCCTTGAGTCAAGGAAATTACTATGA
>XP_024948534.1_3-*Citrus_sinensis*
ATGTTTCCTGCAATAATGGCTATTACAACTGATGAAACAAAGGCAAATGATGATGATCAAGTATACCAGA
AAGGAGTGAAGAATCTTTGCGAAAACGGCGTGACGAAAGTCCCTAAAAAGTACATATTGCCTGAATTCGA
TCGAGCAGATATAGTACTACAAGTAGATTTGACCATGCCAAATCTTAAGCTGCCTGTTATTGATTTTTCC
GAATTGCAAGGCCCAAATCGTTCCGAGGTCCTAAAATCTCTAACTAGTGCTTGTGAAAAATATGGGTTTT
TCCAGGTAGTGAATCACGGTATCCCGAACAATGTTATTAGCAGCATGATTGATGTGAGTGCAAGGTTTTT
TGAACTCCCAATTGAGGTAAGATCCAAGTACGTGTCATCAGATCTCACAGCACCAGTTCGATATGGAACT
AGCTTTAATCAGAATAAGGATAGGGTCTTCTGTTGGAGAGACTTCTTGAAGCTCGTATGCCACCCCTCAC
AAGATGTCCTTGTTCATTGGCCTTCTTATCCAACGGACATGAGGAGATTGGCGGTTACCTACGCAAAAGA
AATCAAAAACTTGTTCGTAATGCTAATGGAGGCTATCTTAGAGAGCTTGGGGCTGTGGGGAGCCACCATG
AATAAGACTGAAGAAGTTGAAAAACTAATAAAGCAATTCCAAGATGGGAGCCAGCAAATGGTGGTCAATT
GCTACCCTCCATGTCCTGAGCCTCATCTAACCCTAGGAATGCCTCCACATTCAGATTATGGATTTCTTAC
TCTTCTTCTACAAGATGACGTTGAGGGTTTGCAAATTCAATATAAAGAAAAGTGGGTCGCAGTTCAACCT
TTAGCCAGCTCCTTTGTGGTTAATGTTGGTGATCACCTTGAGATATTCAGCAATGGCAAGTACAGGAGTG
TGTTACATAGGGTTCTTGTCAATCCAGCCAAGTCTCGCATTTCTGTTGCTTCTTTGCATTCTCTGCCTTT
CAACTCAATGGTTAGCCCATCGCCCTCACTCATCAATAAAGCAAATCCAAGGCGCTATAAGGAGACCGAT
TTTGCCTGTTTCCTTGAATACATTTCATCTTTTGAGCCTAAGAGGAAGAATTTCCTTGAGTCAAGGAAAT
TACTATGA
>XP_024948535.1_4-*Citrus_sinensis*
ATGTTTCCTGCAATAATGGCTATTACAACTGATGAAACAAAGGCAAATGATGATGATCAAGTATACCAGA
AAGGAGTGAAGAATCTTTGCGAAAACGGCGTGACGAAAGTCCCTAAAAAGTACATATTGCCTGAATTCGA
TCGAGCAGATATAGTACTACAAGTAGATTTGACCATGCCAAATCTTAAGCTGCCTGTTATTGATTTTTCC
GAATTGCAAGGCCCAAATCGTTCCGAGGTCCTAAAATCTCTAACTAGTGCTTGTGAAAAATATGGGTTTT
TCCAGGTAGTGAATCACGGTATCCCGAACAATGTTATTAGCAGCATGATTGATGTGAGTGCAAGGTTTTT
TGAACTCCCAATTGAGGTAAGATCCAAGTACGTGTCATCAGATCTCACAGCACCAGTTCGATATGGAACT
AGCTTTAATCAGAATAAGGATAGGGTCTTCTGTTGGAGAGACTTCTTGAAGCTCGTATGCCACCCCTCAC
AAGATGTCCTTGTTCATTGGCCTTCTTATCCAACGGACATGAGGAGATTGGCGGTTACCTACGCAAAAGA
AATCAAAAACTTGTTCGTAATGCTAATGGAGGCTATCTTAGAGAGCTTGGGGCTGTGGGGAGCCACCATG
AATAAGACTGAAGAAGTTGAAAAACTAATAAAGCAATTCCAAGATGGGAGCCAGCAAATGGTGGTCAATT
GCTACCCTCCATGTCCTGAGCCTCATCTAACCCTAGGAATGCCTCCACATTCAGATTATGGATTTCTTAC
TCTTCTTCTACAAGATGACGTTGAGGGTTTGCAAATTCAATATAAAGAAAAGTGGGTCGCAGTTCAACCT
TTAGCCAGCTCCTTTGTGGTTAATGTTGGTGATCACCTTGAGATATTCAGCAATGGCAAGTACAGGAGTG
TGTTACATAGGGTTCTTGTCAATCCAGCCAAGTCTCGCATTTCTGTTGCTTCTTTGCATTCTCTGCCTTT
CAACTCAATGGTTAGCCCATCGCCCTCACTCATCAATAAAGCAAATCCAAGGCGCTATAAGGAGACCGAT
TTTGCCTGTTTCCTTGAATACATTTCATCTTTTGAGCCTAAGAGGAAGAATTTCCTTGAGTCAAGGAAAT

TACTATGA
>scaffold02358_CDS_DMR6-*Castanea_mollissima*
ATGTCTCCTGCAATGGTGATGGCAGCTAAAACAAAGCAGAATGACAACCTAGAAAGCCAATACCAGAAAGGAG
TGAAGCACCTATGTGAAAATGGCATAAACAAAGTTCCCAAGAAATACATATTACCCGTTTCCGATCGACCCAA
TATGAAAGATAGAGAGCTAATTAATGTCAAGAAGCAAAATCTTAAGTTGCCCATCATTGATTTTGCAGAATTG
CAAGGTTCCAACCGGCCTCAAGTCCTTAAGTCTCTTGCTAATGCTTGCGAACAATATGGTTTTTTCCAGTTGG
TAAACCATGGAATTCCAAGCGATGTTATAAGCAGTATGATCGACGTGAGCACAAGGTTTTTCGAGCTCCCATT
TGAGGAAAGAGCCAAGTACATGTCCTCGGACATGCAATCACCAGTTAGATGCGGAACTAGCTTTAACCAAAAC
AAAGACGATGTGTTTTGTTGGAGAGACTTCTTGAAGCTTATGTGTAATCCCATATCAGATGTTGTCCCTCATT
GGCCTTCTTCTCCCGTGGACTTGCGGAGATTGATGGCTACCTACGCAAAAGAAACCAAATACTTGTTCCTAAT
GATCATGGTGTCCATCCTAGAAAGCTTAGGTCTTGTGGGAACCACAGAGCACAAGACAGAAGAAGACGAAATT
TTAAAGGACTTTGAAGATGGTAGCCAGCTAATGGTTGTTAATTGCTACCCTCCATGCCCTGAACCTAATCTAA
CCCTAGGAATACCACCTCACTCCGACTATGGATTCCTCACTCTTCTTCTCCAAGATGAGGTTGAGGGTTTACA
AATACAACACCAAGACAAATGGATCAACGTTGAACCAATCGCAAATGCCTTTGTTGTCAACGTTGGTGATCAT
CTCGAGATCTTTAGCAACGGGAAGTACAAGAGTGTTTTACATAGAGTCTCGGTGAATCCTATGAAGTCTCGGA
TATCGGTGGCTTCTTTGCATAGTCTTCCTTTCAATTACATGGTTAAGCCATCGCCTAAACTCATCAACGAAGA
GAATCCAAGGGGTTACAAGGACACTGACTTTTCTAGCTTTCTTGAGTACATTTCATCCTGTGAGCCCAAGAGG
AAGAATTTCTTGGAGTCAAGGAAACTATGCTGA
>XM_008813993.3_*Phoenix_dactylifera*
ATGGCCCCTCCAATTCTCCCACACACCGCTCCCATACCCTCCCCTCCCCCCAGCTCCGTGAAAGAGCTCA
CCAACTCCACATCCCTTGGCTCCGTTCCCCCCCAGTATGCCATCCGAAACCCCGAGACCACCATGGACAT
CGAACCCATCATCGAAGAAGAAATCCCAACCGTTGACTTCTCTCTGCTGACCGAAGGAACACCTGAGCAA
AGGTCTCAGGTGGTTCGCCACCTCGGCAAGGCTTGTGAGGACTGGGGCTTCTTCATGGTCGTGAACCATG
GTATACCGGAGAGACTAAGGGAGGCGATGCTGATCTCGATGAAGGAATTCTTTGATCAGACGGAGGAGGA
GAAGGGAGAGTACACTGGCAAGCATGTGATGGATCCCATCAGGGAGATATCAAAGGAGTACGCTGCGTCC
ACTAGAGCAATGGGAATGGAACTTCTGAGAGGAATATGGGAGAGCTTGGGATTAGAGGAGAATGACATGA
CCAAGGCCTTGGACCTCCATTCCTGTTACCAGGTTCTCGTGGGCAATATATACCCTCCGTGCCCACAACC
CGAGCTGGCCATCGGCCTCCCCGCTCACTCGGACCATGGCCTTCTCACCATCCTCCTGCAGAACGGCGTC
AACGGACTCCAAGTGAAGCGGAGAGGCAGCTGGGTCCGTGTCGAACCCCTCCCTAACTCGTTCCTCGTCA
ACACTGGCGATCAAATGGAGGTCGTCAGCAATGGAAGGTATAAGAGTGTGCTGCACCGGGCTGAGGTGAA
TAGCCAAAGCACGAGGATGTCGATAGCAACCGTGGTGGCACCATCGCTTGATACGATTGTCGAGCCAGCC
TCTCAGCTGGTGGGCGATGAGCACCCGATGATGTTTCGAGGCATGCGGTATGGAGAGTACTTGGAGTGTC
AGCAGGCCACCCGGCTCAAGGCCAAGTCTGCGCTGGATCCTGTAAGACTGCATGCCGAGTAG
>XM_003572252.3_*Brachypodium_distachyon*
ATGGCAGCACCTTCTCCAAACGGAGTGCACGGAGGCTGTACGGGACCGAAGTTCAGCGAGGAAGGCATCC
CAGTCGTCGACCTCGGCGTCCTCGTCAACGGCGACGGCGACCAACGGGCCAGGGCGGTTCGGCACCTCGG
CCGGGCGTGCCAAGAATGGGGCTTCTTCATGGTGATCAACCACGGGGTGCCGGAGGCTCTGCGAGAAGCA
GTGATGGAAGCATGTCAGGAGCTATTCAGCCTGCCGACGGAGGACAAGGCCGAGCACATGGACGGCAGCC
CGATGGACCCCGTGCGGGTCGGCACGGGCTTCGTCAACTCCGCCGTCGACGGCGCCAAGTTCTGGAGGGA
CTACGTGAAGATGTTCGTCCACCCCGATTTCCACTGCCCCGCGAAGCCTGCGAAGCTGCGGGACGTCGCG
GCGGAGTACGCGGCGAGGACGAGGCAACTGATGCTGGAGCTCACGGCGGCGATCTCCGAGAGCCTGGGCC
TCCGTGGAGACCGCATTGCTGAGGCCCTGAATCTGGCGTCTGGACTTCAGATTCTCGTGGGGAACCGTTA
CCCGCCGCACGCTGGTGGCGGCCCGGAGGACGACGATGGGCCGCCGCCGATTGGGCTACCGGCCCACTCC
GACCATGGGCTCCTCACGCTGCTCTTCCAGAACGGTGTCGACGGCCTGCAGGTCCATCATGATGGCCGCT
GGCTCCTCGCCAAGCCCATTCCTGGCTCCTTCTTCGTCATCGCCGGCGATCAGCTCGAGATTGTGAGCAA
TGGGAGGTACAAGGGCGTGCTCCACCGCGCGCGGGGCGGCGGGGAGCAGGCGAGGATGTCGTTCGTGAGC
CTGATCGGGCCGTGCCTGGACGCCGTCGTCGAGCCGGTGCCGGACCTGGCAACGGAGGGCGCACGGGGCC
TGGAGTTCAGGGGCATCAAGTACAGAGACTACATGGAGTACCAGCAGAGCAACGTGCTCCACGAGAAGGC
GGCGCTGGACATCGTCCGCGTGCACCGTGGCATCGTTGCAGGCGAGGGCTCACCGGATAATTCTTCGGTT
AATTGA
>XM_002445630.2_*Sorghum_bicolor*
ATGGCGATCGTCGACTTGGCCAACGCCCAGCTGCAGCAAGCAGGAGCAGGAGCAGCAGCAGCCACGATGA
GAGAGGACGACGACGGCCATGACCATGAGCAGGAGTCCTCCTACGACTACGGCGCCTGCCTGATGAAAGG
CGTCAGGCACCTCTCCGACAGCGGCATTACCAGGCTGCCCGACAGGTACGTCCTGCCCGCGTCCGACCGC

CCCGGCGCCGCCACCGTCCTTATTTCCCCCTCGTCGACGATCCCGGCGGCGGCAGGCGTCGCCGTCGGCA
GGGTCAAGCTCCCTGTCGTCGACCTCGCCGGCCTCCGCGACCCCTCCCACCGCTCCGCCGTCCTGGCCAC
CCTCGACGCCGCCTGCCGCGACTACGGCTTCTTTCAGGTGGTGAACCACGGGTTCGGGAGCGAGGTGAGC
GGCGGGATGCTGGACGTGGCGCGGCGGTTCTTCGAGCTGCCGCTGCCGGAGCGGGCGCGGCACATGTCGG
CGGACGTGCGTGCGCCGGTGCGGTACGGGACCAGCTTCAACCAGGCCAAGGACGCCGTGCTCTGCTGGCG
GGACTTCCTCAAGCTCGTCTGCCAGCCGCTGCGCGAGGTGGTCCCGCGGTGGCCGCAGCAGCCGCCGGAC
CTCAGGGACGTCGCCACCAGGTACGCCACGGCGAGCCACGCGCTGTTCATGGAGGTCATGGCCGCGGCGC
TGGAGGCCCTGGGCATCCCCGCCGGCGGAGGCGTGCTCGGGGAGCTGGCGGCGGCGTCGTCGCACATGAT
GACGGTGAACTGCTACCCGGCGTGCCCGCAGCCGGACCTCACGCTGGGGATGCCGCCGCACTCGGACTAC
GGCCTCTTCACCTTCGTCCTGCAGGACCACGTGGAGGGGCTTCAGGTCATGCACGACGGCCGCTGGCTCA
CCGTCGACCCCGTCCCGGGATCCTTCGTCGTCAACGTCGGCGACCACCTAGAGATCTACAGCAACGGGCG
GTACAAGAGCGTGCTGCACCGGGTGCGCGTGAACTCGACGCGGCCGCGGATCTCGGTGGCGTCGTTCCAC
AGCTTGCCGGCGGAGAGGGTGATCGGGCCGGCGCCGGAGCTGGTGGACGAACAGGCCGGCAACCCGCGGC
GGTACATGGACACCGACTTCGCCACGTTCCTCGCCTACCTCGCGTCCGCCGACGGCAAGAACAAGACCTT
CCTCCAGTCAAGGAAGCTGCCAGTGCCAGCTGCTGCTGCATGCGTCTAG
>XM_009420612.2_*Musa_acuminata*
ATGGACGTAACGGTGGAGAACAGAGCCATGAAGGGAGTGAGGTACCTCTGTGAAAATGGCATCACAAGGA
TCCCTCCCAGGTACGTCTTGCCCCTCTCCGATCGGCCCCAGCTCGCGCCGGCCGTTCGTAAACCCAATCT
CCAGCTTCCCGTCGTCGACATCGGCCAGCTGCTTTCGCCCGACCGCGCCAGCGTCCTCGAGACATTAGAC
AGAGCTTGCAAGGAGTATGGCTTCTTCCAGGTGGTGAATCATGACGTAGATGGTGAGGCTATTCGGAGAA
TGATCGACGTCGGGAAGAGATTCTTCGAGCTTCCGTTCGAGGAGAGAGAGAGGTACATGACGACCGACGT
GAGGAGCGCAGTGAGGTACGGGACCAGCTTCAACCAGACCAAGGATCGAGTGTTCTGTTGGAGGGACTTC
TTGAAGCTGAGCTGCCATCCTCACGACACTGTTCTTCCACACTGGCCTTCTTCTCCCACGGATTTGAGGG
AGGAAGCAGCTTCATATGCCAAGCACGTCAAGTCCTTGTTCTTGGTCGTCATGGCGGCCGTCCTCGAGAG
CCTCGGTGTGGGAACCTCCGCTCTCGACGAGTTCGACGCCGGATCCCAACTCATGGTGCTGAATTGCTAC
CCAGCGTGCCCTCAGCCTGATCTTACCCTCGGGATGCCGGCCCACTCCGACTACGGCTTCCTCACGTTCG
TCCTCCAGGACGAGGTCGAGGGCCTCCAGGTGCTGCACCGCGACGAATGGATCACCGTCGAGCCCGTTCC
GAACGCCTTTGTCGTCAACGTCGGCGATCACCTTGAGATATTTAGCAATGGAAGATACAGTAGCGTGCTG
CACCGGGTGTTGGTCAACTCCTCCAGGTCGCGGTTGTCCGTGGCCTCCCTCCACAGCCTCCCGTTCGACA
GAGTCGTACGGCCGTCGCCGGAGCTCGTCAACGAAGGGAACCCCAGGATGTACGTGGACACGGATTTCGC
CGCTTTCCTGGACTACATGTCCTCCTGCGAGCCCCAGCGCAAGAACTTCGTCGACTCCAGGAAGTTGACC
CGCCGCTCATAG
>XM_009409398.2_*Musa_acuminata*
ATGGTTCTGGAATTCGATCCCGCCTTCATCCAAGCCCCCGAGCACCGCCCCAAGCCCGCCGTCACCGAGG
CCGGCGGCATCCCCCTCATCGACCTCTCCCCTCTCCACCTCCTCGAACAACACGGGGGACCACTCGTGGC
GGGGTTGGAGGTGCTGGTGGCGCAGGTGGAGGCGGCTTGCCGTGACTGGGGCTTCTTCCAGGTGATCAAC
CATGGGGTGCCGCTGGCGGTGGTGGAACGAGCGTGGGCCGCGTCGAGGGGGTTCTTCGCGCTGCCGCCGG
AGGAGAGGCGGCGTGTGCGGCGGAACGAGGTGAACCCGCTGGGGTACTACGAGGCGGAGAACACCAAGAA
CGTCCGGGACTGGAAGGAGGTGTTCGACTACGTGGTCCACGAGCCGGCCGACGCTGGTGGAGGCGTCGAT
AGGCTGATCGAGCTCCGCAACCAGTGGCCACAGTTTCCCCATGGATTCAGGGAAGCACTTGTTGAGTATG
CTCAGGCCACGGAGGAGTTGGCTTTCAAGCTGCTGGAGCTGATATCACTGACCTTGAGCTTGCCGCCAAA
GCGACTGCATGGCTTCTTCAAGGACCAGACCAGCTTAATCAGGCTCAATCACTACCCGCCCTGCCCCTCC
CCTCACCTCGCCCTCGGCGTTGGCCGCCACAAGGACCCCGGAGCCCTCACCATTCTCGCTCAGGACGACG
TCGGCGGCCTCGACGTCAGGAGGCGGTCCGACGGCGAGTGGATTCGCGTCAAGCCCATCCCCAACTCCTT
TATCATCAACGTCGGTGACATCGTTCAGGTGTGGAGCAATGACAAGTACGAGAGTACAGAGCACAGGGTG
TCGGTGAACTCCGAGAGGGAGAGGTTCTCCATACCCTTCTTCTTCAACCCGGCGCACGACGTGATGGTGA
AGCCCTTGGCAGAGCTGGTAGACGAGAAGAACCCGGCCAACTACGAGGAGTACAACTGGGGGGAGTTCCT
GAAGACAAGGGCGGACGGCAACTTCAAGAAGCTGGATGTGGAGAACATCCAGATATATCATTTCAAGAAG
GCCAAGTGA
>XM_009389881.2_*Musa_acuminata*
ATGGCGACAACCACCACAAGGCTCCTCCTCGGCGACCTGGTGTCTCACGCTAAGAACGTCCCGCTTAGAT
ACGTCCGGCCACCCTCCGCCCGCCCCCACCTCTCCGCCGTCGAGAAGTCGAATGCCACGATCCCGGTCGT
AGACCTGCAGGAGCTCTCCGGGTCCGGTCGTGCCATGGTGGTCAAGGCCATTGGGTCAGCTTGCCAAAGC
GATGGGTTCTTCCAGGTCAAGAACCACGGCATTCCCGACGACGTGATCGGTGCCATGTTGCGTGTCTCGA

AGGAGTTCTTCCGGTTGCCGGAGTCGGAGAGGCTGAAGAGCTACTCCGACGACCCTTCGAGGACGACGAG
GCTGTCGACGAGCTTCAACGTGAGGACCGAGGAGGTTTGCAACTGGAGGGACTTTCTGAGGTTCCATTGC
TATCCTCTCAAGGACTACGTGCATGAATGGCCCTCCAATCCTTCCGTTTTCCGGGAGGTGGTGGGTGACT
ACTGCAAGCACGCCAGGCAACTGGCTTTGAGGTTGCTGGAGGCCATCTCGGAGAGCTTGGGACTTGAGAA
GGACTACATGGAGAAGGCACTGGCGAAGCAGGCACAACACATGGCCATAAACTACTATCCACCATGCCCA
CAGCCGGAGCTCACGTACGGGCTGCCGAGCCACAAAGACCCCAATGCCATCACCCTACTCCTCCAGGACG
GCGTCTCCGGCTTGCAAGTCTTCAGGAACGGCAAGTGGGTGGCCGTCGACCCCATCCCCAACGCCTTGGT
CATCAACATCGGCGATCAGATTCAGGTGCTCAGCAATGATCGATACAAAAGCGTGCTCCATCGCGCAGTC
GTCAACGACTCCAGCGAGAGGATTTCTATTCCCACCTTCTATTGCCCATCTCCCGATGCAGTAATTGGAC
CAGCTCAAGCACTGGTTGACGAGCAGCATCCTGCAGTCTACCGAAGCTTCACATATGGGGAGTACTATGA
CGCGTTCTGGAACCGAGGCCTCCAACGCGAGAGCTGCCTCGACATGTTCAGAGCCACCAACGATCCAATC
TAA
>XM_020405450.1_*Asparagus_officinalis*
ATGGCTTCTCCTCTTCATCTAGATGGCTCAGATGTCAATAACAACTCTTCAATCAAAGATCTTGAAAACA
TAGTCGAAGATGTGGAGATTCCAGTCATCGATTTCTCTCTGCTTACTGAAGGAACACCTGATCAACGGTA
TCTGGTTGTTCAGGAACTTGCCAAGGCCTGCGAAGACTGGGGATTCTTTGTGCTTGTGAACCATGGAGTA
CCTGATAAACTGAGGAATGCTTTAATGGACACATTCAAGGGATTCTTCAACCTACCGCCAGAAGACAAAC
AGGATTCCGCTGACACGCATGCGATGAATCCTATCCGATACGGAACCAGCTTCAATGCAAAGGTAGATGA
TAACAAATACTGGAGGGAGTATCTTAAAGTTATGACACACCCAGAGTTTCACTGTCCTACAAAGCCTCCG
CAATTTAGAGAAGAACTGAAAGAGTACAGTGCATGCATAAGAGAGCTAGGAAAGAAATTGCTTGGAGGGA
TCTGGGAGGGTCTTGGACTAGACAATAACTACATGAGAGAGGCATTAAACCTCGGCAATTGCTTCCAAAT
TGTCGTAGGAAACCACTATGCTCCATGTCCACAGCCGGAAAAGGCTATGGGCTTACCCCCTCATTCTGAC
CACGGCCTAATCAGTATCCTCTATCAGAACGACGTCGATGGTCTTGAAGTACAACATGACGGTAAATGGG
TCCGTGTCAAGCCGTTACCCAACTCGTACCTCATCAACACAGGTGACCAAATGGAGATTGTTAGCAATGG
CAAATACAAGAGCATTGTCCACCGAGCTGCGGTAAATGAGAAGAGAGCGAGGATGTCAATTGTGACTGTT
ACAGGTCCATCCTTGGACACAACTGTGGTCGCAGCTCCCCAGCTCGTCAGCGGCGAGAGCCCGGCAACAT
TCCGAGGGATGAAATATGGAGAGTTTATGGAGTACCAACAGGGTCACGGCCTCCAGGAGAAATCTGTTTT
GAAGCTGTTGCGACTATGA
>XM_020419075.1_*Asparagus_officinalis*
ATGGTGATGGACTCGGGGTTGGCTGAGACAAAAAGAGAAGAAGACGAGGGCAAGTTTGTGAAAGGGGTAA
GACATCTTTGCGAGAGTGGCATAACTAAGGTCCCTCACAAGTACATATTACCGGTCCCCGAGCGACCTGA
GCCTAGAGAAGAGATGATTTCCAATCCGAAACTCAAATTGCCTATCATTGATCTTGCTCTGTTAGATACT
CCGGACAGAAGTCAAGTTCTTCAAACAATGGCGAATTCTTGCGTGGAGTATGGCTTCTTTCAGGTAGTGA
ACCATGGCATTGATAGAGAGCTTATCTCGAGAATGGTCGACGTCGGGAAGAGATTCTTCGAACTTCCTTT
GGAGGAGACGGAGAAATATATGTCCAGTGATATAAGAGCTCCGGTGAGATATGGGACGAGCCTTAACCAA
CTAAAAGACGGTGTGTTATTTTGGAGAGATTTTCTCAAGCTCATTTGTCATCCTTTAGAGAATGTTCTTC
AACAATGGCCTACTTCTCCCATGGACTTGAGGGAGGTGGCAGCTTCATATGCAAAGCAAACCAAATCGCT
CTTCCTCATCCTGATGTCTTTAATCCTGGAGAACCTTGGAATCGACGACAACCGCAAACACGATGACGAC
TTTGATGACGGGACACACATGATGGTCCTAAACTGCTATCCTGCATGCCCGGAGCCGGACCTGACGTTCC
TGACGCTCCTCCTCCAGCAAGATGCGGTTCACGGCCTCCAAGTTCAGTGCAAGGGAGAGTGGGTCACCGT
CGAGCCTGTGCCCGGGTCAATTGTTGTTAACGTTGGTGATCACCTTGAGATATTCAGCAACGGGAAGTAC
AAGAGCGTCCTACACAGAGTCCTGGTCAACTCTTCAAGGTCAAGAATCTCCGTCGCCTCGCTCCACAGCC
TCCCTTATGATACAATGGTCAGCCCATTGCCGGAGCTGGTCAACGACAAGAACCCCAAGCTCTACAAAGA
CACGGACTTCGCCTCCTTCTTGAATTTCATTTCCTCCAGCGAGACCAAGCACAAGAATTTCCTAGAGACA
AGAAAGTTGAAGAACGAGACAAAGTAA
>XM_020419498.1_*Asparagus_officinalis*
ATGAATCCAGAATTTGATACATCTTTCATCCAGAACCCCGACAGTCGACCGAACCCAAACATTTCCGAGG
CCGAATCAATCCCTCTCATCGACCTCTCTCCATTATTCACAACCCCCGTTAACTTAACACCCCTGATTTC
AGAAATCGAGGCAGCTTGCAGTAATGTTGGGTTCTTCCAGGCCATCAACCATGGCGTACCGGCAGAACTC
CTCCAGAGAGTTCAGTCGGCAGCAAAGGAGTTCTTCGCTCTGTCGACGGAGGAGAAGAGCAGAGTGAGGA
GGAATGAGGAGAGATTTCTTGGGTATTACGATACGGAGAATACGAAGAACGTTAGGGATTGGAAGGAGGT
GTTCGATTTTTGTGTGAATGATCCTATGACTGTTCCGACGTCGAGTGAAAGTGGGGAGACGGCGGTGCAG
CAGATTAGGAACCAGTGGCCGGAGAATCCAGAGGGTATGAGAGATGCATGCATGGACTATGCAAAAGCCG
TCGAAGGCCTATCATTCAGACTGCTCGAGCTCATCGCGATGACCTTGAACGCACCCGCCAAGCGACTGAA

CGGCTACTTCGAGGACTCCATCAGCAGAATCCGCCTCAACCATTACCCTCCGTGCCCGTCCCCTGACCTT
GCCCTCGGCGTGGGACCGCACAAGGACCAAGGAGCTCTCACGGTCTTGGCGCAGGACGACGTTGGTGGCC
TCGATGTCAAGAGGAAGTACGATGGCCAGTGGGTCCGCGTCAAGCCCGTTCCTAACTCGTACATCATCAA
CATAGGGGATCTCATCCAGGTTTGGAGCAACGACAAGTACGAGAGCACGGAGCACAGGGTATCAGTGAAC
TCAGAGAAGGAGAGGTTTTCGATCCCGTTCTTCTTATATCCTGCGCACTACACAGTGATCAGGCCTGTAG
AGGAGCTTGTGAGTGAGGATAATCCTGCTAAATACAATGAGTTCAACTGGGGCCATTTCTTCCGGTCACG
AATGAGCAGCAATTTTCAAAAGCTGGGAGTAGAAAATCAGCAGATATATCATTTTAGGAAGAAAACCTAA
>XM_020420925.1_*Asparagus_officinalis*
ATGGGTTCAGAATTTGGTCCAGCCTTCATTCAGAAACCCGACCATCGACCCAAACCCAACATCTCCGATG
TAGAATCAATCCCTATCATCGACCTCTCTCCACTTTTCACAGCTCCAATCACCGCCGGTGACAATACGCT
ACCTATCAGAACGCTTGTTGCAGAAATCGAAGCAGCATGCAGAGAAGTTGGCTTCTTCCAAGTCATCAAC
CACGGCGTCCCGATCGAACTCCTCGAACGAGTTCAGTCGGCAGCGAGAGAGTTCTTCGCTCTGCCGACTG
AGGAGAAGAGGAGAGTGAGGAGGAACGAGGAGAATTTTCTTGGGTATTATGATATGGAGAATACGAAGAA
CGTTAGGGATTGGAAGGAGGTGTTTGATTTTTGTGTTAATGACGTGATGACTGTTCCAGCGTCGGGTGAA
ATTGGCGAGACAAGGATCCAGGAGATTTGGAATCGGTGGCCAGAGTATCCGGAGAACATGAGAGATGCCT
GCATGAATTACTCAAAAGCCATCGAAGACCTGTCATTCAAACTGCTCGAGCTCGTTGCGATGACTTTAAA
CTTACCGGCCAAACGACTGAACGGCTACTTCAAGGACTCCATCAGCAGAATCCGCCTCAACCATTACCCT
CCATGCCCTTCCCCTGACCTTGCCCTCGGCGTGGGACCGCACAAGGACCAAGGAGCGTTCACAGTCCTCG
CACAGGACAATGTCAGTGGCCTCGATGTCAAGAGGAAGTCCGATGGACAGTGGGTCCGTGTCAAGCCCGT
TCCCGACTCATACATCATCAATATAGGTGATCTCATCCAGGTTTGGAGTAACGACAAGTATGAGAGCACG
GAGCACAGGGTGTCGTTGAACTCGGACAAGGAGAGGTTTTCAATCCCATTCTTCTTCTATCCTGCACATT
ATACAATTATGAAGCCTATAGGGGAACTTGTGACTGAAGATAATCCAGCTAAATACAATGAATTCAGTTG
GGGACGTTTCTTCAGGTCTCGGAGGAGCAGTAATTTTCAGAAGATGAGAGTGGAAAATCTGCAGATATAT
CATTTTCGGAAGAACAATTGA
>XM_020259536.1_*Ananas_comosus*
ATGGCAATGGATTTAGAAGCGGAGGGGGAGAAGAAAGAGGAGGCGGCGGAGCGGCAGTACATGGAAGGGG
TGAAGCAGCTTTGCGAGAGCGGCATCAAGAAGGTGCCCAACAAGTACATCTTGCCAGCCTCGGAGCGTCC
TCGGGTGAACGGACGGCGCGATACCGCCGCTCAAAACCTCAGTCTCAAGCTGCCCGTCATTGATCTCTCT
CTGTTGCACTCGAAAGACCACGCATCGGTTATTCAGTCCTTGGAAAAGGCCTGCGAGGAGTACGGATTCT
TTCAGATAGTGAACCACGGCATTCCGATCGAGGTTATAAGGGATATGGTCGGCGTCGTGAGGAGATTCTT
CGAGCTTCCGTTTGAGGAGAGATCAAAGTATATGTCGTCCGACATACGAAATCCAGTTAGGTACGGGACG
AGCTTCAACCAAATGAACGACAAAGTGTTTTCTTGGAGAGACTTCCTGAAACTCAGCTGCCATCCTATCT
CCAGCGCTCTCCCGTTTTGGCCTTCTTCTCCTGCAGACTTAAGGGACAAAGCACTTTTATATGTCAAGGA
AATGAAGAGTTTGTTCATGGATCTGATGGAAGCGATTTTGGAGACTCTAGGAGTGAACCGTGGCGTGCTC
GAAGAATTTAGAACTGGATCGCATATCCTTGTAGCGAATTGTTATCCGGTGTGCCCCGAACCGGGACTTA
CCCTCGGAATGCCGCCCCATTCTGACTATGGCTTCCTCACTCTTCTCCTCCAGGACGACGTCGAGGGGCT
ACAAATCCAGCACGGCGACGAATGGGTCACCGTCGAGCCAGTCCCTGACTCTCTTGTCGTAAACATCGGC
GATCATTTTGAGATATTCAGTAACGGGAGGTACAAGAGCGTTCTCCACCGAGTGTTGGTCAACCCTGCGA
AGCTCCGAATCTCCGTCGCATCCCTCCACAGTCTCCCCATCGAGCGCGTCGTCGGGCCCTCCCCCGACCT
TGTCGACGACGACGACAACCCGCGGCGTTACATGGACACGGACTTCGCCTCGTTCCTCGATTACATCGCC
TCGTGCGAATCGAAGCACAAGAACTTCCTCGAGTCAAGGAGGTTGACTCCTGGCAAGGCCCTCCCTGATA
GTCAACCAATTTGA
>XM_010941407.3_*Elaeis_guineensis*
ATGGCCCCTCCAACTCTCCCACACTCCTCTCTCATACCCTCACCTCCCCCCAACTCCGTCAAAGAGCTCA
CCGACGCCGCATCCCTTCGCTCCGTTCCTCTCCAATATGCCACCCGAAACCCCGACAACACCATAGGCAT
CGAACCCATCATACAAGAAGAAATCCCAACCCTTGATTTCTCTCTGCTGACCGAAGGAACACCTGAGCAG
AGGTCTCAGGTGGTTCACCACCTCGGCAAGGCTTGTGAGGAGTGGGGCTTCTTCATGGTGGTGAACCATG
GTATCCCGGAGAGACTGAGGGAAGCGATGCTGGTCTCAATGAAGGAATTCTTTGATCAGACGGAGGAGGA
GAAGGGAGAGTGCGCTGGCAAGCATGTGATGGATCCCATCAGGGAGATATCAAAGGAGTACGCCAAATGC
ACTAGAGCCATGGGAATGGAACTACTGAGAGGAATATGGGAGAGCTTGGGATTGGAGGAGAATGACATGA
CCAATGCCTTGGACCTCCATTCCTGTTTCCAGGTTCTTGTTGGCAATATATACCCTCCATGCCCACAGCC
CGAGCTGGCCACCGGCCTTCCCGCCCACTCGGACCATGGCCTTCTCACCATCCTCATCCAGAATGGTGTC
AATGGACTCCAAGTAAAGCACGGAGGCAACTGGGTCCGTGTCGAACCCCTCCCTAACTCGTTCCTCGTCA
ACACTGGCGATCACATGGAGGTCGTTAGCAATGGAAGGTATAAGAGTGTGTTGCACCGGGCTGAGGTGAA

CGGCCAAAGCACGAGGATCTCCATAGCAACCGTGGTGGCACCATCACTTGATGCAATTGTGGAGCCAGCC
TCCCAGCTGGTGAGCGATGAGCACCCGATGATGTTTGGAGGTATGCGGTATGGAGAGTTCTTGGAGCGTC
AGCAGGCCAACCGGCTCAAGGAGAAGTCTGTGTTGGATCTTTTAAGACTGCATGCCGAGTAG
>XM_010941408.3_*Elaeis_guineensis*
ATGGCCCCTCCAACTCTCCCACACTCCTCTCTCATACCCTCACCTCCCCCCAACTCCGTCAAAGAGCTCA
CCGACGCCGCATCCCTTCGCTCCGTTCCTCTCCAATATGCCACCCGAAACCCCGACAACACCATAGGCAT
CGAACCCATCATACAAGAAGAAATCCCAACCCTTGATTTCTCTCTGCTGACCGAAGGAACACCTGAGCAG
AGGTCTCAGGTGGTTCACCACCTCGGCAAGGCTTGTGAGGAGTGGGGCTTCTTCATGGTGGTGAACCATG
GTATCCCGGAGAGACTGAGGGAAGCGATGCTGGTCTCAATGAAGGAATTCTTTGATCAGACGGAGGAGGA
GAAGGGAGAGTGCGCTGGCAAGCATGTGATGGATCCCATCAGGTATGGGACTAGTTTCAATTCAACAGTG
GAGGATGTCAGGTACTGGAGGGACTATATGAAGGTTTTTGCGCATCCGGTTTTCTATTCACCTGCTAAGC
CTCCAAGTTTTAGGGAGATATCAAAGGAGTACGCCAAATGCACTAGAGCCATGGGAATGGAACTACTGAG
AGGAATATGGGAGAGCTTGGGATTGGAGGAGAATGACATGACCAATGCCTTGGACCTCCATTCCTGTTTC
CAGGTTCTTGTTGGCAATATATACCCTCCATGCCCACAGCCCGAGCTGGCCACCGGCCTTCCCGCCCACT
CGGACCATGGCCTTCTCACCATCCTCATCCAGAATGGTGTCAATGGACTCCAAGTAAAGCACGGAGGCAA
CTGGGTCCGTGTCGAACCCCTCCCTAACTCGTTCCTCGTCAACACTGGCGATCACATGGAGAGATACAGG
ACGCTCATGATTGGCTATGGTTTGGATTGTGGCCAATGGAGTCTAAAATTATAA
>XM_035684691.1_*Juglans_regia*
ATGACCTCGGCAAAGCCTGCCAAGACTGGGGCTGTTTCATGGTTGATCGATCATCAGGTGATCAATCATG
GTGTGCCGGAGAGCCTGATGAAGGCGATGGTTGAGGCGTGTGGAGAATTTTTCAATCTGGCGGAGGAGGA
GAAGCAAGAATTTGCAGGGAAGCATGTCTTGGACTCGCTCAGGTGTGGCGCCAGCATCAATGGTTCAACC
GACCACGTATTTTTTTGGAGGGATTATCTCAAGTTTTTTGCACATCCCAACTTTCACTCTCCAAGCAAAC
CCACAGGATTCAGAGAGATTGCATTAGATTACAGCACAAGAACACGAAAAGTGGCTATGGAGTTGGTGAA
AGGAATATCAGCGAGCTTGGGATTGGAAGAGGGCTATATAGAGAAGGCTTTGAATATGGAATTGGGTCTT
CAGATCTTCATTTCAAACTTCTTTCCGCCTTGTCCACAACCTGAACTCGCAAGGGGATTGCCTCCTCATT
CCGACCACGCCCTCTTGACCCTTCTTATAAATAATGGCATCTCTGGCTTTCAGCTGCAGCGTCAAGGGAA
ATGGGTCAATGTTAATTCGATTCCTAATGCATTCATGGTTATCTTTGGCGATCAAATGGAGATTATGAGC
AACGGCAAGTACGAGTGTGCTGTACATCGGGCAACTGTGAATGGAAAAGCTACAAGAATGTCGATAGCCA
TGCTGTTTGGACCAGCACTAGAAACAGTTGTTGGGCCAGCACCAGAGTTGGTTGACAATGAAACCAATCC
ACCAGCATTCACCGCCATGAAGTACAGGAAATACTTGGAACTGCAGCAAACCAACAAGATAATTGGGAAA
TGCGCTCTGGATCGTGTCCGAATCTTGTCTGTCTAG
>XM_018954296.2_*Juglans_regia*
ATGATAAGCATCAAACAACTAGCTGAATCACCTGGCCTCACCTCCGTCCCTCCCACCTACACTTTCACCC
CGAATCCCAATGATCATCCAGTTTCAGATGATCAAAAAGACTCAATCCCCGTCATTGACTTCGCTCGCCT
CACCTCCGGCACTCCCGATCAACGCTCCAAGCTCGTCCATGACCTTGGCAAGGCCTGCCAAGACTGGGGC
TTCTTCATGGTGATCAATCATGGTGTGCCCGAGAGCCAGATGAAGGCGTTGATCAAGGCTTGCGGTGAGT
TCTTCAATCTGAAGGAGGAGGAGAAGCAGGAATTTGCAGGGAAGCATGTTCTGGATTCCATCAGGTGCGG
CACCAGCTTTAATGCATCTGTGGACAAAGTATTATTTTGGAGGGATTTTCTCAAGGTTTTTGTACATCCC
AAGTTTCACTCACCGACTAAACCCTCTGGATTCAGAGAGATCTTATTGGAGTACAGCAAAAGAACCCGAA
AACTAGCTCTGGACTTGCTGGAAGGAATATCAGTGAGCTTGGGATTAGAAGACGGCTATATGGAAAAGGC
CATGAATTTGGAATCGGGTTTACAGATCCTGGCTGCAAACTTTTATCCGCCATGTCCAGAGCCAGAAGTA
GCAATGGGCATGCCTTCTCATTCTGATCACGGCCTTTTGACCCTCCTCATACATAATGGAATCTCCGGCC
TACAGGTGCAACATAAAGGAAAGTGGATTAATGTAAATTCCATTCCCAATGCATTCGTGGTTAACATTGG
CGACCACTTGGAGATTGTGAGCAACGGCAGGTACAAGAGTATCTTACATCGGGCAACAGTGAATGAAAAA
GCTACCAGAATATCGATAGCTACGGTTTATGGACCATCGCTAGACACGGTTGTTAGTCCAGCACCAGAGT
TGGTTAACCTTGAAACCAATCCACCAGCATTCAAAGGAATGAAGTACGGGGAGTACTTGGAAATGCAGCA
GAGCAACAAGCTTGATGGCAAATCCTGTTTGCATCATGTGGCTGTCTAG
>XM_035684690.1_*Juglans_regia*
ATGATGTTAAGCACCAAACAACTAGCCGAAACGCCTGGCCTCACCTCCATCCCTCGCACCTACAACTTCA
CTCGGGATCCCAACGAAGAACCAATTTCGGATGAGTTTGAAGACTCCATCCCTGTCATTGACTTCTCTCT
CCTCGCCTCCGGTACTCCTCATCAACGGTCCAAGATCGTCCATGACCTCGGCAAAGCCTGCCAAGACTGG
GGCTGTTTCATGGTGATCAATCATGGTGTGCCGGAGAGCCTGATGAAGGCGATGGTTGAGGCGTGTGGAG
AATTTTTCAATCTGGCGGAGGAGGAGAAGCAAGAATTTGCAGGGAAGCATGTCTTGGACTCGCTCAGGTG
TGGCGCCAGCATCAATGGTTCAACCGACCACGTATTTTTTTGGAGGGATTATCTCAAGTTTTTTGCACAT

CCCAACTTTCACTCTCCAAGCAAACCCACAGGATTCAGAGAGATTGCATTAGATTACAGCACAAGAACAC
GAAAAGTGGCTATGGAGTTGGTGAAAGGAATATCAGCGAGCTTGGGATTGGAAGAGGGCTATATAGAGAA
GGCTTTGAATATGGAATTGGGTCTTCAGATCTTCATTTCAAACTTCTTTCCGCCTTGTCCACAACCTGAA
CTCGCAAGGGGATTGCCTCCTCATTCCGACCACGCCCTCTTGACCCTTCTTATAAATAATGGCATCTCTG
GCTTTCAGCTGCAGCGTCAAGGGAAATGGGTCAATGTTAATTCGATTCCTAATGCATTCATGGTTATCTT
TGGCGATCAAATGGAGATTATGAGCAACGGCAAGTACGAGTGTGCTGTACATCGGGCAACTGTGAATGGA
AAAGCTACAAGAATGTCGATAGCCATGCTGTTTGGACCAGCACTAGAAACAGTTGTTGGGCCAGCACCAG
AGTTGGTTGACAATGAAACCAATCCACCAGCATTCACCGCCATGAAGTACAGGAAATACTTGGAACTGCA
GCAAACCAACAAGATAATTGGGAAATGCGCTCTGGATCGTGTCCGAATCTTGTCTGTCTAG
>XM_013611254.2_*Medicago_truncatula*
ATGGGAGACATAGATTCATCTTTCATACAAGCCACAGAACACCGTCCTAAGTTATCTACCTTTGTACAAG
TTGATGAAATTCCAATCATTGACCTCTCAGAAAGTAGTCAACAAAACCTCATCTCAAAGATTGGCAAGGC
ATGTGAAGAATGGGGATTTTTTCAAGTAATCAATCATGGAGTTCCATCTGATGTTAGTACAAAGGTTGAG
ATTGAGGCCAAAAAGTTTTTTGAGCAAAGCATTGAGGAGAAGAAGAAAGTGAAAAGAGATGCAGTTAATG
CAATGGGATATCATGATGCTGAACATACTAAGAATATAAGGGATTGGAAAGAGGTTTTTGATTTTCTTGT
TGAGAATTCCCCACAAATCCCTTCTTCTCATGAGCCAGATGACTTGGAGCTTAGGACTCTCACCAACCAA
TGGCCACAATACCCTCCTCATTTTAGGGAAACAATGCAGGAGTATGCTAGAGAAGTTGAAAAGCTAGCTT
ATAAGTTATTGGAGTTGATTTTATTGAGCTTAGGCATAGGTGGTGAAAAATTTCATGACTGCTTCAAGAA
CCAACTAAGCTTGGTGAGGCTCAATCACTATCCTCCATGCCCTTCCCCTGATTTGGCACTTGGTGTTGGT
CGTCACAAGGATTCTAGTGCTTTGACTGTGCTTGCACAAGATGATATTGGTGGCTTACAAGTTAGGAGAA
AATCAGTTGGTGATTGGATTCCAGTTAAACCTACTCCTGGTGCATTTATCATCAATGTGGGTGATATTGT
TCAGGTATGGAGCAATGACAAGTATGAGAGTGTGGAGCACAGAGTGGTTGTAAACACAAAGAAAGAAAGG
TTTTCCATTCCATTCTTCTTTTACCCAGGTCACCATGTTACAGTGAAGCCTGCAGAAGAGCTAGTGAATG
AGAAAAATCCTGCAAGATATAAACCATACAATGTTGGCAAGTTCTATGCTAATAGAAACCGTAGTGATTT
CAACAAACGTGAGGTTGAGAATATCCAAATTCATCACTTCAAGATCGTGGATTAG
>XM_024776469.1_*Medicago_truncatula*
ATGGGAGACATAGATTCATCTTTCATACAAGCCACAGAACACCGTCCTAAGTTATCTACCTTTGTACAAG
TTGATGAAATTCCAATCATTGACCTCTCAGAAAGTAGTCAACAAAACCTCATCTCAAAGATTGGCAAGGC
ATGTGAAGAATGGGGATTTTTTCAAGTAATCAATCATGGAGTTCCATCTGATGTTAGTACAAAGGTTGAG
ATTGAGGCCAAAAAGTTTTTTGAGCAAAGCATTGAGGAGAAGAAGAAAGTGAAAAGAGATGCAGTTAATG
CAATGGGATATCATGATGCTGAACATACTAAGAATATAAGGGATTGGAAAGAGGTTTTTGATTTTCTTGT
TGAGAATTCCCCACAAATCCCTTCTTCTCATGAGCCAGATGACTTGGAGCTTAGGACTCTCACCAACCAA
TGGCCACAATACCCTCCTCATTTTAGGGAAACAATGCAGGAGTATGCTAGAGAAGTTGAAAAGCTAGCTT
ATAAGTTATTGGAGTTGATTTTATTGAGCTTAGGCATAGGTGGTGAAAAATTTCATGACTGCTTCAAGAA
CCAACTAAGCTTGGTGAGGCTCAATCACTATCCTCCATGCCCTTCCCCTGATTTGGCACTTGGTGTTGGT
CGTCACAAGGATTCTAGTGCTTTGACTGTGCTTGCACAAGATGATATTGGTGGCTTACAAGTTAGGAGAA
AATCAGTTGGTGATTGGATTCCAGTTAAACCTACTCCTGGTGCATTTATCATCAATGTGGGTGATATTGT
TCAGGTATGGAGCAATGACAAGTATGAGAGTGTGGAGCACAGAGTGGTTGTAAACACAAAGAAAGAAAGG
TTTTCCATTCCATTCTTCTTTTACCCAGGTCACCATGTTACAGTGAAGCCTGCAGAAGAGCTAGTGAATG
AGAAAAATCCTGCAAGATATAAACCATACAATGTTGGCAAGTTCTATGCTAATAGAAACCGTAGTGATTT
CAACAAACGTGAGGTTGAGAATATCCAAATTCATCACTTCAAGATCGTGGATTAG
>XM_024776495.1_*Medicago_truncatula*
ATGGGAGACATAGATTCATCTTTCATACAAGCCACAGAACACCGTCCTAAGTTATCTACCTTTGTACAAG
TTGATGAAATTCCAATCATTGACCTCTCAGAAAGTAGTCAACAAAACCTCATCTCAAAGATTGGCAAGGC
ATGTGAAGAATGGGGATTTTTTCAAGTAATCAATCATGGAGTTCCATCTGATGTTAGTACAAAGGTTGAG
ATTGAGGCCAAAAAGTTTTTTGAGCAAAGCATTGAGGAGAAGAAGAAAGTGAAAAGAGATGCAGTTAATG
CAATGGGATATCATGATGCTGAACATACTAAGAATATAAGGGATTGGAAAGAGGTTTTTGATTTTCTTGT
TGAGAATTCCCCACAAATCCCTTCTTCTCATGAGCCAGATGACTTGGAGCTTAGGACTCTCACCAACCAA
TGGCCACAATACCCTCCTCATTTTAGGGAAACAATGCAGGAGTATGCTAGAGAAGTTGAAAAGCTAGCTT
ATAAGTTATTGGAGTTGATTTTATTGAGCTTAGGCATAGGTGGTGAAAAATTTCATGACTGCTTCAAGAA
CCAACTAAGCTTGGTGAGGCTCAATCACTATCCTCCATGCCCTTCCCCTGATTTGGCACTTGGTGTTGGT
CGTCACAAGGATTCTAGTGCTTTGACTGTGCTTGCACAAGATGATATTGGTGGCTTACAAGTTAGGAGAA
AATCAGTTGGTGATTGGATTCCAGTTAAACCTACTCCTGGTGCATTTATCATCAATGTGGGTGATATTGT
TCAGGTATGGAGCAATGACAAGTATGAGAGTGTGGAGCACAGAGTGGTTGTAAACACAAAGAAAGAAAGG

TTTTCCATTCCATTCTTCTTTTACCCAGGTCACCATGTTACAGTGAAGCCTGCAGAAGAGCTAGTGAATG
AGAAAAATCCTGCAAGATATAAACCATACAATGTTGGCAAGTTCTATGCTAATAGAAACCGTAGTGATTT
CAACAAACGTGAGGTTGAGAATATCCAAATTCATCACTTCAAGATCGTGGATTAG
>XM_028385394.1_*Glycine_soja*
ATGGGAGAGGTTGACCCAGCTTTCATTCAAGAACCACAACACAGGCCAAATCTCTCCACCATTCAAGCAG
AAGGAATTCCCATAATTGATCTCTCTCCAATAACCAACCACACAGTTTCAGACCCTTCTGCAATTGAAAG
CTTGGTGAAGGAGATAGGACGTGCATGCCAGGAGTGGGGCTTCTTCCAAGTAACAAACCATGGAGTGCCA
CTCACTCTAAGGCAAAACATTGAGAAAGCCTCAAAACTGTTCTTTGCTCAGACTCTGGAGGAGAAGAGAA
AGGTTAGCAGAAATGAGAGCTCTCCAATGGGTTATTATGACACAGAACACACCAAGAACGTCAGGGACTG
GAAAGAAGTCTTTGATTTTCTAGCCAAAGACCCCACTTTCATTCCTCTCACTTCTGATGAACATGATGAT
CGAGTCAATCAATGGACTAATCAATCACCTCAATACCCTCCACTCTTCAGGGTTGTAACACAAGAGTATA
TTCAGGAGATGGAAAAGCTGTCCTTTAAGCTGTTGGAGCTGATAGCTTTGAGCTTAGGCCTTGAAGCAAA
GAGGTTTGAGGAATTTTTCATCAAAGATCAAACTAGCTTTATTCGACTCAACCACTATCCTCCATGCCCT
TACCCTGACCTTGCTCTTGGCGTCGGTCGACACAAGGACCCCGGTGCCTTGACCATTCTTGCACAGGACG
AAGTTGGAGGACTTGAAGTGAGACGTAAACGGGATCAAGAGTGGATTAGAGTGAAACCAACCCCAAATGC
TTATATTATCAACATTGGTGATACTGTTCAGGTTTGGAGCAATGATGCATATGAGAGTGTGGATCACAGA
GTGGTGGTCAACTCTGAGAAGGAAAGGCTTTCCATTCCGTTCTTCTTCTTCCCTGCACACGACACCAAAG
TAAAGCCTTTGGAGGAGCTGATAAATGAGCAAAACCCTTCAAAATATAGGCCATACAATTGGGGCAAGTT
TCTTGTCCACAGAGGGAACAGCAATTTCAAGAAGCAAAATGAGGAGAACATTCAAATTCATCATTACAAG
ATAGCTTAA
>XM_027214233.1_*Coffea_arabica*
ATGTCTCCTGCATTAGTAGCAACACCGGCATCACCGCAGAGCATGATGAGCATCACAAACGAACATCCTG
TTGAAAGCCAATACCAAAAAGGAGTAAAACACCTGTATGAAATTGGCATCAGGAGGGTTCCCCCCAAGTA
TATAGTGCCAGTTTCGGAACGTCCAAATCAGACACACTGGAAAACTCAGACGACCGATGGCAGTCTTCAG
CTTCCAGTCATTGATTTGTCAGGATTGCACGGACCTAAACGTTCTCAAGTCGTTAGGTCTCTCGCCGGCG
CTTGTGAAAATTACGGGTTTTTCCAGGTTGTGAATCACGGCATTCCGAACGAGGTAATCAGCAATATGAT
GGACGTGACCAAGAGGTTCTTCGAGCTTCCTTCAGCAGAAAGAGAGAAATATATGTCAGCAGATATGAGC
AAGCCGGTTCGATATGGCACCAGCTTCAATCAGCTAAAAGATAGCGTCTTCTGTTGGAGGGATTTCTTGA
AATTAGTCTGCAACCCTCTAGCAGATGTCCTTTCTCACTGGCCTTCTTCTCCCATGGACTTAAGGGGATT
GGCGGTTACCTACGCTAAAGAAACCAAATTCTTATATCTGATGATATTAGAAGCAATTTTGGAGAGCCTC
GGGCTAACAACCACGAAGAACACCACAGCTGATACGGATGACCAAATCTTGAGCGAAATAAAAGATGGAA
GCCAGCAAATGTTCCTTAATTTTTACCCTCCGTGTCCAGAACCCGATTTGACATTTGGACTAAGATCCCA
TTCTGACTTTGGAATCCTAACGCTTCTTCTCCAGGATGAAGTTGCAGGGCTTCAAATAAAGCATCAAGGG
AGATGGCTCGCCATTGAACCGATCCCGGGCTCCTTCGTTGTCAATGTTGGAGATCAACTTGAGATCTTCA
GCAACGGCAGATACAAGAGCGTACTGCATAGAGTAGTTGTAAACTCCAGCATGTCTCGAATTTCAGTGGC
TTCTCTTCACAGTCTTCCATCCCAATCCGCGGTGCGCCCGGCGGAGAAGCTTATAAATCAAACAAATCCA
AGGCGTTACAGGGACACTGATATTGCTGCTTTTCTTCATCACATAAAATCCTGCGACTACCAAGAGAAAG
GTGTCCTGGAGTCCAGGAAATTGACCTGA
>XM_020259537.1_*Ananas_comosus*
ATGGCAATGGATTTAGAAGCGGAGGGGGAGAAGAAAGAGGAGGCGGCGGAGCGGCAGTACATGGAAGGGG
TGAAGCAGCTTTGCGAGAGCGGCATCAAGAAGGTGCCCAACAAGTACATCTTGCCAGCCTCGGAGCGTCC
TCGGGTGAACGGACGGCGCGATACCGCCGCTCAAAACCTCAGTCTCAAGCTGCCCGTCATTGATCTCTCT
CTGTTGCACTCGAAAGACCACGCATCGGTTATTCAGTCCTTGGAAAAGGCCTGCGAGGAGTACGGATTCT
TTCAGATAGTGAACCACGGCATTCCGATCGAGGTTATAAGGGATATGGTCGGCGTCGTGAGGAGATTCTT
CGAGCTTCCGTTTGAGGAGAGATCAAAGTATATGTCGTCCGACATACGAAATCCAGTTAGGTACGGGACG
AGCTTCAACCAAATGAACGACAAAGTGTTTTCTTGGAGAGACTTCCTGAAACTCAGCTGCCATCCTATCT
CCAGCGCTCTCCCGTTTTGGCCTTCTTCTCCTGCAGACTTAAGGGACAAAGCACTTTTATATGTCAAGGA
AATGAAGAGTTTGTTCATGGATCTGATGGAAGCGATTTTGGAGACTCTAGGAGTGAACCGTGGCGTGCTC
GAAGAATTTAGAACTGGATCGCATATCCTTGTAGCGAATTGTTATCCGGACGACGTCGAGGGGCTACAAA
TCCAGCACGGCGACGAATGGGTCACCGTCGAGCCAGTCCCTGACTCTCTTGTCGTAAACATCGGCGATCA
TTTTGAGATATTCAGTAACGGGAGGTACAAGAGCGTTCTCCACCGAGTGTTGGTCAACCCTGCGAAGCTC
CGAATCTCCGTCGCATCCCTCCACAGTCTCCCCATCGAGCGCGTCGTCGGGCCCTCCCCCGACCTTGTCG
ACGACGACGACAACCCGCGGCGTTACATGGACACGGACTTCGCCTCGTTCCTCGATTACATCGCCTCGTG
CGAATCGAAGCACAAGAACTTCCTCGAGTCAAGGAGGTTGACTCCTGGCAAGGCCCTCCCTGATAGTCAA

CCAATTTGA
>XM_027214234.1_*Coffea_arabica*
ATGTCTCCTGCATTAGTAGCAACACCGGCATCACCGCAGAGCATGATGAGCATCACAAACGAACATCCTG
TTGAAAGCCAATACCAAAAAGGAGTAAAACACCTGTATGAAATTGGCATCAGGAGGGTTCCCCCCAAGTA
TATAGTGCCAGTTTCGGAACGTCCAAATCAGACACACTGGAAAACTCAGACGACCGATGGCAGTCTTCAG
CTTCCAGTCATTGATTTGTCAGGATTGCACGGACCTAAACGTTCTCAAGTCGTTAGGTCTCTCGCCGGCG
CTTGTGAAAATTACGGGTTTTTCCAGGTTGTGAATCACGGCATTCCGAACGAGGTAATCAGCAATATGAT
GGACGTGACCAAGAGGTTCTTCGAGCTTCCTTCAGCAGAAAGAGAGAAATATATGTCAGCAGATATGAGC
AAGCCGGTTCGATATGGCACCAGCTTCAATCAGCTAAAAGATAGCGTCTTCTGTTGGAGGGATTTCTTGA
AATTAGTCTGCAACCCTCTAGCAGATGTCCTTTCTCACTGGCCTTCTTCTCCCATGGACTTAAGGGGATT
GGCGGTTACCTACGCTAAAGAAACCAAATTCTTATATCTGATGATATTAGAAGCAATTTTGGAGAGCCTC
GGGCTAACAACCACGAAGAACACCACAGCTGATACGGATGACCAAATCTTGAGCGAAATAAAAGATGGAA
GCCAGCAAATGTTCCTTAATTTTTACCCTCCGTGTCCAGAACCCGATTTGACATTTGGACTAAGATCCCA
TTCTGACTTTGGAATCCTAACGCTTCTTCTCCAGGATGAAGTTGCAGGGCTTCAAATAAAGCATCAAGGG
AGATGGCTCGCCATTGAACCGATCCCGGGCTCCTTCGTTGTCAATGTTGGAGATCAACTTGAGATCTTCA
GCAACGGCAGATACAAGAGCGTACTGCATAGAGTAGTTGTAAACTCCAGCATGTCTCGAATTTCAGTGGC
TTCTCTTCACAGTCTTCCATCCCAATCCGCGGTGCGCCCGGCGGAGAAGCTTATAAATCAAACAAATCCA
AGGCGTTACAGGGACACTGATATTGCTGCTTTTCTTCATCACATAAAATCCTGCGACTACCAAGAGAAAG
GTGTCCTGGAGTCCAGGAAATTGACCTGA
>XM_025124378.1_*Cynara_cardunculus*
ATGAACTCAGTTAAAGCATTAGCCGAATCACCTGATCTCAACTCCATCCCTTCTCTTTACGCCTACTCCA
AAAACGCTACTGATTCCCCTGCTTCGGATCCTCAAGATCCAATCCCCACCATTGATTTTTCCTTACTCAC
CTCCGCTGATCCTGATCAACGGTCCCAAGTTATCCAGGAGCTCGATAAAGCCTGCAAAGATTGGGGCTTT
TTTCAGGTGATTAATCATGGTGTGCCCGAGAGTTTGATGAAGATGGTTATGGAAAAAGCCGGTGAATTTT
TCAATTTGACAGGGGAAGAGAAGCAAGATTTCCAGGAAAAGGATGTTCTTGATCCAATCAGGTATGGAAC
AAGCTTCAATTCTAAGAAGGATAAGGTCTTCTGTTGGAGGGACTTTCTCAAGGTTATCGTACACCCAGAG
TTTCACTCTCCGAACACGCCCTTAGGTTTCAGTGAGGTTCTATTGGAGTACTCTAATAGAACAAGAGAAG
TGGTAAGGGGTTTGCTTAGCGGGATATCGACGAGCCTTGGACTCGACCAATCGTACGTCGAAAAAGCGTT
GAAATTGGAATCCGGTTTACAAATATGTATCGCCAATCTTTACCCTCCATGCCCACAACCAGAAGTTGCA
ATCGGGCTTCCGCCTCATTCTGACCATGGCCTCTTGACGCTCGTGATCAACAACGGTGTTAGCGGACTTC
AAATCAAGCACGACGGCAAATGGATCGATGTGAAAGATACTCTTCCCAACTCGTTTCTAGTCAACACCGC
GGACCAGCTTGAGATTTTCAGCAACGGGAAGTACAAGAGTGTGGAGCATCGTGCGGTGGTGAATGATGTA
GTGACGAGGATATCGGTGGTTGTAGCGAACGGGCCGGCACTGGATGCGGTGGTGAGACCGGCGGATAAGT
TGGTGGACGAAGAAAGGCGGCCGGTGGCATATATCCCGATGAAGTACGAAGAGTATTTGAAAATGCAACA
AGGCAACCAAATCAACGGGAAAACTTGTTTGGACAGGGTTCGGGTATGA
>XM_025124377.1_*Cynara_cardunculus*
ATGAACTCAGTTAAAGCATTAGCCGAATCACCTGATCTCAACTCCATCCCTTCTCTTTACGCCTACTCCA
AAAACGCTACTGATTCCCCTGCTTCGGATCCTCAAGATCCAATCCCCACCATTGATTTTTCCTTACTCAC
CTCCGCTGATCCTGATCAACGGTCCCAAGTTATCCAGGAGCTCGATAAAGCCTGCAAAGATTGGGGCTTT
TTTCAGGTGATTAATCATGGTGTGCCCGAGAGTTTGATGAAGATGGTTATGGAAAAAGCCGGTGAATTTT
TCAATTTGACAGGGGAAGAGAAGCAAGATTTCCAGGAAAAGGATGTTCTTGATCCAATCAGGTATGGAAC
AAGCTTCAATTCTAAGAAGGATAAGGTCTTCTGTTGGAGGGACTTTCTCAAGGTTATCGTACACCCAGAG
TTTCACTCTCCGAACACGCCCTTAGGTTTCAGGTTTGACATCAAAAGTCAAAGGTTGAAGGGTTTAACAT
GTGATTGCAGTGAGGTTCTATTGGAGTACTCTAATAGAACAAGAGAAGTGGTAAGGGGTTTGCTTAGCGG
GATATCGACGAGCCTTGGACTCGACCAATCGTACGTCGAAAAAGCGTTGAAATTGGAATCCGGTTTACAA
ATATGTATCGCCAATCTTTACCCTCCATGCCCACAACCAGAAGTTGCAATCGGGCTTCCGCCTCATTCTG
ACCATGGCCTCTTGACGCTCGTGATCAACAACGGTGTTAGCGGACTTCAAATCAAGCACGACGGCAAATG
GATCGATGTGAAAGATACTCTTCCCAACTCGTTTCTAGTCAACACCGCGGACCAGCTTGAGATTTTCAGC
AACGGGAAGTACAAGAGTGTGGAGCATCGTGCGGTGGTGAATGATGTAGTGACGAGGATATCGGTGGTTG
TAGCGAACGGGCCGGCACTGGATGCGGTGGTGAGACCGGCGGATAAGTTGGTGGACGAAGAAAGGCGGCC
GGTGGCATATATCCCGATGAAGTACGAAGAGTATTTGAAAATGCAACAAGGCAACCAAATCAACGGGAAA
ACTTGTTTGGACAGGGTTCGGGTATGA
>XM_025969333.1_*Panicum_hallii*
ATGGCCCCGACCATCGCCAAGCCTCTGCTCAGCGATCTCGTGGCGCAGACGGGGCAGGTCCCATTGAGCC

ACGTCAGGCCTGTCGAAGACCGCCCGGACCTCGCGAGCGTCGACAACGAGTCCGGCGCCGGGATCCCGCT
CATCGACCTCAGGAAGCTCGACGGGCCGGAGCGCCGCACGGTGGTGGAGGCCATCGGCAGGGCCTGCGAA
TCCGACGGCTTCTTCATGCGCGAATGGCGCGTATGCGTACAGGTGACGAACCACGGCATCCCGGCGGGGG
TCGTGGAGGGGATGCTGCACGTGGCGCGGGAGTTCTTCCACCTGCCGGAGGCGGAGCGGCTCAAGTGCTA
CTCCGACGACCCCAAGAAGGCCATCCGGCTCTCCACGAGCTTCAACGTGCGCACCGAGAAGGTCAGCAAC
TGGCGAGACTTCCTGCGCCTGCATTGCTACCCGCTCCAGAGCTTCGTCGACCAGTGGCCGTCCAACCCGC
CCTCATTCAGGCAAGTGGTGGGCACCTACGCGACGGAGGCGCGGGCGCTGGCGCTGCGGCTGCTGGAGGC
CATATCGGAGAGCCTGGGCCTGGAGCGGAGCCACATGGTGACGGCCATGGGGAGGCAGGCGCAGCACATG
GCGGTGAACTACTACCCGCCGTGCCCGCAGCCGGAGCTCACCTACGGGCTGCCGGGGCACAAGGACCCCA
ATGCCATCACGCTGCTGCTCCAGGACGGCGTCTCCGGCCTGCAGGTGCAGCGCAACGGCCGCTGGGTGGC
CGTGAACCCCGTGCCCGACGCGCTGGTCATCAACATCGGCGACCAGCTGCAGGCGCTGAGCAACGACCGC
TACAAGAGCGTGCTCCACCGCGTGATCGTGAACAGCGAGAGCGAGCGGATTTCGGTGCCGACGTTCTACT
GCCCGTCGCCGGACGCGGTGATCGCGCCGGCGGACGCGCTGGTGGACGACGGCCACCCGCTGGCCTACCG
GCCGTTCACCTACCAGGAGTACTACGACGAGTTCTGGAACATGGCCTCCAATCCGCGAGCTGCCTCGAC
CGGTTCAGACCCGGCTGA
>XM_025969334.1_*Panicum_hallii*
ATGGCCCCGACCATCGCCAAGCCTCTGCTCAGCGATCTCGTGGCGCAGACGGGGCAGGTCCCATTGAGCC
ACGTCAGGCCTGTCGAAGACCGCCCGGACCTCGCGAGCGTCGACAACGAGTCCGGCGCCGGGATCCCGCT
CATCGACCTCAGGAAGCTCGACGGGCCGGAGCGCCGCACGGTGGTGGAGGCCATCGGCAGGGCCTGCGAA
TCCGACGGCTTCTTCATGGTGACGAACCACGGCATCCCGGCGGGGGTCGTGGAGGGGATGCTGCACGTGG
CGCGGGAGTTCTTCCACCTGCCGGAGGCGGAGCGGCTCAAGTGCTACTCCGACGACCCCAAGAAGGCCAT
CCGGCTCTCCACGAGCTTCAACGTGCGCACCGAGAAGGTCAGCAACTGGCGAGACTTCCTGCGCCTGCAT
TGCTACCCGCTCCAGAGCTTCGTCGACCAGTGGCCGTCCAACCCGCCCTCATTCAGGCAAGTGGTGGGCA
CCTACGCGACGGAGGCGCGGGCGCTGGCGCTGCGGCTGCTGGAGGCCATATCGGAGAGCCTGGGCCTGGA
GCGGAGCCACATGGTGACGGCCATGGGGAGGCAGGCGCAGCACATGGCGGTGAACTACTACCCGCCGTGC
CCGCAGCCGGAGCTCACCTACGGGCTGCCGGGGCACAAGGACCCCAATGCCATCACGCTGCTGCTCCAGG
ACGGCGTCTCCGGCCTGCAGGTGCAGCGCAACGGCCGCTGGGTGGCCGTGAACCCCGTGCCCGACGCGCT
GGTCATCAACATCGGCGACCAGCTGCAGGCGCTGAGCAACGACCGCTACAAGAGCGTGCTCCACCGCGTG
ATCGTGAACAGCGAGAGCGAGCGGATTTCGGTGCCGACGTTCTACTGCCCGTCGCCGGACGCGGTGATCG
CGCCGGCGGACGCGCTGGTGGACGACGGCCACCCGCTGGCCTACCGGCCGTTCACCTACCAGGAGTACTA
CGACGAGTTCTGGAACATGGCCTCCAATCCGCGAGCTGCCTCGACCGGTTCAGACCCGGCTGA
>XM_016637269.1_*Nicotiana_tabacum*
ATGGAGACCACAGCTGCTCCACTGCTTCAAACACCCAATTATATCAAACAATTAGCTGAATCGCCTGATC
TCCACTTCATACCTGCTAACTATGTTCACTCTACAAATAATCCATGTGACTCCTCTGATTCAAACTCAAT
TCCCATCATTGACTTTTCTCTACTCACTTCGGGTGATCCTCATCAATGTTCCATAGCCATCCACCACCTC
AGCAAAGCTTGTCAAGATTGGGGCTTCTTCATGGTAGTGAACCATGGCATACCAGAAAATTTGATTAAGG
CTGTGATTGATTGCACACATGAATTTTTCAACTTACCAGAGGAAGATAAGCGGGAGTATGCTGGGAAACA
TGTATTGGATCCTATAAGATGTGGGACAAGCTTCAATGCCTCTAAAGAAAATGTCTTCTTCTGGAGAGAC
TATCTTAAGGTCTTTGTGCATCCTCAGTTTCACTCCCCCACCAAACCCCAAGGCTACAGGGATATTGTGT
CGGAGTATTGTGAGAAAATACGGGAAGTGGCAAAAATGTTACTAGGAGGTATATCAGAAAGCCTGGGGCT
TGAAGAAGGTTTCTTGGACAAAGCTCTAGACTTGAAGTCAGGCCTTCAAATATTTGTGGGAAATTACTAT
CCAAATTGTCCTCAACCTGAACTTACAATGGGGATGCCACCCCATTCAGATCATGGCCTTTTAACTCTTC
TCATTCAGAACCAAGTTGGAGGTCTCCAAGTTCAGCATGAAGGCAAATGGATTAATGTCAATGCCCTACC
TAATTCTCTATTGGTAAACACCGGCGACCATCTTGAGATATTCAGTAATGGAAAGTACAAGAGCAATATG
CATAGAGCAGTGGTGAACAACAAAGTAACAAGGATATCAATAGCCACAGCTCATGGTCCATCACTGGAAA
CAATTGTGAGCCCAGCTTCTCCACTTGTATATAATGAAAACAGCGCAGCAGCTTATATTCCAATGAAGTA
CAGTGAATACTTGGAGTTGCAACAAAGCAACCAACTAGATGGAAAGTCCTGCTTGGAAAGACTCAAGTTT
CCAAGGAACTGA
>XM_016637270.1_*Nicotiana_tabacum*
ATGGAGACCACAGCTGCTCCACTGCTTCAAACACCCAATTATATCAAACAATTAGCTGAATCGCCTGATC
TCCACTTCATACCTGCTAACTATGTTCACTCTACAAATAATCCATGTGACTCCTCTGATTCAAACTCAAT
TCCCATCATTGACTTTTCTCTACTCACTTCGGGTGATCCTCATCAATGTTCCATAGCCATCCACCACCTC
AGCAAAGCTTGTCAAGATTGGGGCTTCTTCATGGTAGTGAACCATGGCATACCAGAAAATTTGATTAAGG
CTGTGATTGATTGCACACATGAATTTTTCAACTTACCAGAGGAAGATAAGCGGGAGTATGCTGGGAAACA

TGTATTGGATCCTATAAGATGTGGGACAAGCTTCAATGCCTCTAAAGAAAATGTCTTCTTCTGGAGAGAC
TATCTTAAGGTCTTTGTGCATCCTCAGTTTCACTCCCCCACCAAACCCCAAGGCTACAGGGATATTGTGT
CGGAGTATTGTGAGAAAATACGGGAAGTGGCAAAAATGTTACTAGGAGGTATATCAGAAAGCCTGGGGCT
TGAAGAAGGTTTCTTGGACAAAGCTCTAGACTTGAAGTCAGGCCTTCAAATATTTGTGGGAAATTACTAT
CCAAATTGTCCTCAACCTGAACTTACAATGGGGATGCCACCCCATTCAGATCATGGCCTTTTAACTCTTC
TCATTCAGAACCAAGTTGGAGGTCTCCAAGTTCAGCATGAAGGCAAATGGATTAATGTCAATGCCCTACC
TAATTCTCTATTGATATTCAGTAATGGAAAGTACAAGAGCAATATGCATAGAGCAGTGGTGAACAACAAA
GTAACAAGGATATCAATAGCCACAGCTCATGGTCCATCACTGGAAACAATTGTGAGCCCAGCTTCTCCAC
TTGTATATAATGAAAACAGCGCAGCAGCTTATATTCCAATGAAGTACAGTGAATACTTGGAGTTGCAACA
AAGCAACCAACTAGATGGAAAGTCCTGCTTGGAAAGACTCAAGTTTCCAAGGAACTGA
>XM_018649148.1_*Pyrus_x_bretschneideri*
ATGGCTCCAGTTGCTGCTCAAACCATGGATGTAAAGAGCATCAAATCGGTTGCGGAATCACCCGCTCTCA
GCTCTGTTCCTTCTGCTTATGCCTTCAACATAAACCCCAATGATGAAGCAGATCCAAACGATCCTGAATT
TGCAATCCCCATAGTTGATATGTCTCTTCTCACCTCGGGATCTCCTGATCAACGGTCCAAAATAATCCAT
GACCTCGTCAAAATTTGCAAAGAATGGGGCTTCTTCATTGCAATTAACCATGGAGTCCCAGAGAGTCTAA
TGAAGGGGATGATTGACGCATGCCATGGATTTTTCAGCCTCCCAGACGAAGAGAAGGAGGAGTTTAAATC
CGGAAATGATGTGCTGGAGATGTTCAAGTACGGAACAAGCTACAATCTTGCATTAGACAAAGTCCTTCTC
TGGAGGGACTTCTTCAAGGTGAGAGTACATCCCGAGTTCTACTCCCTCTACAAACCGGCTTGCTTCAGTG
AGGTTTCAATGGAGTTCAGCAAGAGAACAAGAGAAGTAGCATTGGAGATAACACGCGCAATCTCTGAAAG
TTTGGGACTGGAGCCCAACTACATACATGATGCGATGAACATGGATCGCGGCCTACAAATGCTCGCCGCC
AACTACTATCCGCCTTGCCCTCAGCCAGAACATGCAATTGGTATACCACATCACACTGATCATGGCCTTG
TCACCCTCCTCATCCAGAATGAGATGAATGGCCTCCAAGTTGAACATAACGGCAAGTGGCTCACCGTCAA
CGGCCCTGCCAACGGTTTCTTTGTCAACCTCGCTGACCAAATGCAAATTCTTACTAATGGCAAATACAAG
AGTGTGATGCATCGGGCAACTGTGAACAACAAGGCTACGAGGATATCTATTGCCATACCACATGGACCAT
CTGTTGATACACTTATCGCACCGGCACCGGAGTTATTGGAAAAAGAAGGTCAAGCTCCGAAATATATTGG
AATGAATTATAAGGAGTATATCCAGCTTCAACAGAGCGGCAAGAACTACATGAAATCCACCTTTGATCAC
ATACTAGCGTAA
>XM_009366012.2_*Pyrus_x_bretschneideri*
ATGGCTCCAGTTGCTGCTCAAACCATGGATGTAAAGAGCATCAAATCGGTTGCGGAATCACCCGCTCTCA
GCTCTGTTCCTTCTGCTTATGCCTTCAACATAAACCCCAATGATGAAGCAGATCCAAACGATCCTGAATT
TGCAATCCCCATAGTTGATATGTCTCTTCTCACCTCGGGATCTCCTGATCAACGGTCCAAAATAATCCAT
GACCTCGTCAAAATTTGCAAAGAATGGGGCTTCTTCATTGCAATTAACCATGGAGTCCCAGAGAGTCTAA
TGAAGGGGATGATTGACGCATGCCATGGATTTTTCAGCCTCCCAGACGAAGAGAAGGAGGAGTTTAAATC
CGGAAATGATGTGCTGGAGATGTTCAAGTACGGAACAAGCTACAATCTTGCATTAGACAAAGTCCTTCTC
TGGAGGGACTTCTTCAAGGTGAGAGTACATCCCGAGTTCTACTCCCTCTACAAACCGGCTTGCTTCAGTG
AGGTTTCAATGGAGTTCAGCAAGAGAACAAGAGAAGTAGCATTGGAGATAACACGCGCAATCTCTGAAAG
TTTGGGACTGGAGCCCAACTACATACATGATGCGATGAACATGGATCGCGGCCTACAAATGCTCGCCGCC
AACTACTATCCGCCTTGCCCTCAGCCAGAACATGCAATTGGTATACCACATCACACTGATCATGGCCTTG
TCACCCTCCTCATCCAGAATGAGATGAATGGCCTCCAAGTTGAACATAACGGCAAGTGGCTCACCGTCAA
CGGCCCTGCCAACGGTTTCTTTGTCAACCTCGCTGACCAAATGCAAATTCTTACTAATGGCAAATACAAG
AGTGTGATGCATCGGGCAACTGTGAACAACAAGGCTACGAGGATATCTATTGCCATACCACATGGACCAT
CTGTTGATACACTTATCGCACCGGCACCGGAGTTATTGGAAAAAGAAGGTCAAGCTCCGAAATATATTGG
AATGAATTATAAGGAGTATATCCAGCTTCAACAGAGCGGCAAGAACTACATGAAATCCACCTTTGATCAC
ATACTAGCGTAA
>XM_024606674.1_*Populus_trichocarpa*
ATGTCACCGGCAATAGTCTTGGAGGTTCAAACTCATTACAAAGAAGAGGAGCTACTAGAGAGTCACGAGT
TCCACAAAGGAGTGCAACATCTATGCGAGAGAGGGATAACAAAAGTGCCTAGAAAATACATATTGCCGGC
ATTGGACCGTCCTATTTTCCCAAAAAAAGATGGCGCTACTAATTTCAAGCTACCAATAATCGATTTTGCT
CAGCTGCAAGGTCCAGATAGAATTCATGCACTCAAATCCCTTTCAAAAGCTTGTGAGGAATATGGTTTTT
TTCAGTTGATAAATCATGGCATTGCATGTCAATCCATCCTCGACATGATTGAAGCAGGAAGGAAATTCTT
CGAGCTTTCTTTCGAGGAGAGATCGAAGTACATGTCAAAAGATATGCGTGCACCAGTTAGATATGGAACA
AGTTTTAACCAGAACAAAGATAGAGTGTTTTGCTGGAGAGACTTCTTAAAGCTTGATTGCCATCCCCTTT
CAGATGTTCTTCCTTATTGGCCCTCTTCTCCTACGGAGCTAAGGCAAGCGGCGGTTAACTACTCTAAAGA
AACAAAATTCTTGTATATAATGGTTGTGAGGGCGATACTAGAGAGCCTAGGATTGGCTGAAACAACAAAA

GATATTGATGAAAATGATGGTGATCATCATATAATTAAGGAGTTTCAAGATGGGAGCCAACTCCTTGTGG
TCAACTGCTATCCTTCATGTCCTGAACCTGACCTCACACTTGGCATACCACCCCATTCAGACTATGGCTT
CCTTACACTGCTCCTCCAAGATGAAGTTAAGGGTCTCCAAATTCAGCATGAAGGGAGATGGGTCACAGTG
GAACCAATCCCTAATTCATTTGTTATTAATGTTGGTGATCATCTCGAGATTTTCAGCAATGGGAGATACA
GGAGTGTGCTCCATAGAGTACTAGTCAATCCTTCGAAGTCTCGGATCTCCATTGCCTCGTTACACAGTTT
ACCTTTTGGCAGCATGGTTCGACCGTCGCCAAAGCTCATCGATGATGCTAATCCAAGGCGTTATAAGGAC
ACAGACTTTGCGAGTTTTATTGAATACATAGCATCTCATGAACACAGGAGCAAGAACTTCCTCGATTCTA
GGAGATTGACTTGA
>XM_020229601.1_*Ananas_comosus*
ATGGCACCAGCAGAGTCCAAGCTCCTCCTAACTGATCGAGCTATATCCAGCTCCAACCATGTCCCTTCCA
ACTACATTAGGCCCGTCGGCGACCGCCCAAACCTCCTCGATGTCGAATCCTCCACCTCCGTCCCCCTCAT
CGACCTTCATGGATTGCTCGGCCCCGCTCGCTCTGATGTAATCAAAGAAATCGGCTCCGCATGCCGAACT
GATGGATTCTTTATGGTTAAGAATCACGGCATCCCGGAGGAAGTTGTTAGAGAGATGCTGCGAGTTGCGA
GAGAGTTCTTTCACTTACCGGAGGCAGAGAGGCTGAAGTGCTACTCCGACGATCCGTCGAGAACGACGAG
GCTGTCGACGAGCTTCAACGTGCGGACGGAGAAGGTCAGCAACTGGAGGGACTTCCTGAGGCTCCACTGC
TACCCGCTCGAGGATTTCATCCGTGAATGGCCCTGCAATCCTCCAGATTTCAAGCAAGTAGTGGGCGAAT
ATTCGAAACATGCGAGAGCGCTCGCAGTGCGACTACTGGAGGGGATATCGGAGAGCTTGGGACTAGAAAG
GGATCACATGGTGAAGGCAATGGGGAAGCAGGCACAGCACATGGCGATAAACTACTACCCGCCATGCCCA
CAGCCCGAGCTGACCTACGGTCTCCCGGGCCACAAAGACCCCAACGCCATCACCGTACTGCTCCCAGACG
GCGTCTCCGGCTTGCAGGTTCTCCGCGGCCAGAAGTGGGTGGCCGTCGAGCCCGTACCGAATGCGTTAAT
CATCAACATGGGCGATCAAATACAGGTGCTCAGCAATGACCGCTACAAGAGCGTACTTCACCGCGTGATC
GTAAACAACAAAAGCGAGAGGATCTCCGTCCCGACCTTCTACTGTCCATCTCCGGACGCGGTTATAGAAC
CAGCTGAGGCTCTCGTCGACGACGATCATCCTCTCAGATATAGAAGTTTCACTTACGGAGAGTACTACGA
GAAGTTCTGGAACCGCGAGCTCCTATCTGCTAGTTGCCTCGATTTGTTCAAAGCGAGCCAGTGA
>XM_024606675.1_*Populus_trichocarpa*
ATGTCACCGGCAATAGTCTTGGAGGTTCAAACTCATTACAAAGAAGAGGAGCTACTAGAGAGTCACGAGT
TCCACAAAGGAGTGCAACATCTATGCGAGAGAGGGATAACAAAAGTGCCTAGAAAATACATATTGCCGGC
ATTGGACCGTCCTATTTTCCCAAAAAAAGATGGCGCTACTAATTTCAAGCTACCAATAATCGATTTTGCT
CAGCTGCAAGGTCCAGATAGAATTCATGCACTCAAATCCCTTTCAAAAGCTTGTGAGGAATATGGTTTTT
TTCAGTTGATAAATCATGGCATTGCATGTCAATCCATCCTCGACATGATTGAAGCAGGAAGGAAATTCTT
CGAGCTTTCTTTCGAGGAGAGATCGAAGTACATGTCAAAAGATATGCGTGCACCAGTTAGATATGGAACA
AGTTTTAACCAGAACAAAGATAGAGTGTTTTGCTGGAGAGACTTCTTAAAGCTTGATTGCCATCCCCTTT
CAGATGTTCTTCCTTATTGGCCCTCTTCTCCTACGGAGCTAAGGCAAGCGGCGGTTAACTACTCTAAAGA
AACAAAATTCTTGTATATAATGGTTGTGAGGGCGATACTAGAGAGCCTAGGATTGGCTGAAACAACAAAA
GATATTGATGAAAATGATGGTGATCATCATATAATTAAGGAGTTTCAAGATGGGAGCCAACTCCTTGTGG
TCAACTGCTATCCTTCATGTCCTGAACCTGACCTCACACTTGGCATACCACCCCATTCAGACTATGGCTT
CCTTACACTGCTCCTCCAAGATGAAGTTAAGGGTCTCCAAATTCAGCATGAAGGGAGATGGGTCACAGTG
GAACCAATCCCTAATTCATTTGTTATTAATGTTGGTGATCATCTCGAGATTTTCAGCAATGGGAGATACA
GGAGTGTGCTCCATAGAGTACTAGTCAATCCTTCGAAGTCTCGGATCTCCATTGCCTCGTTACACAGTTT
ACCTTTTGGCAGCATGGTTCGACCGTCGCCAAAGCTCATCGATGATGCTAATCCAAGGCGTTATAAGGAC
ACAGACTTTGCGAGTTTTATTGAATACATAGCATCTCATGAACACAGGAGCAAGAACTTCCTCGATTCTA
GGAGATTGACTTGA
>XM_015792977.2_*Oryza_sativa*
ATGGCGATCGTTGACTTGGTCAATGCCGGAGAGCAGCAGCAGATGGGCAGCAAGAGAGCGGCGGCGGAGG
ATGGCGACGGCGGCGTGGACGACAGCCGCGAGTACTACTGCCGGCGAGGCGTCCGGCACCTGTGCGACAG
CGGCATTACCAGGCTTCCCGGCAACTACGTCCTCCCGGCCTCCGACCGCCCTGGCCAAGCCGCCGGCGCC
GCCGCCGCCGCCGGGGGGTCAGTGAAGCTCCCCGTGGTGGACCTGTCGCGCCTCCGCGTCCCTTCGGAGC
GCGGCGCGGTGCTCCGGACGCTGGACGCGGCGTGCCGGGAGTACGGGTTCTTCCAGGTGGTGAACCACGG
CGTGGGCGGCGAGGTGGTGGGCGGCATGCTCGACGTGGCGCGCCGCTTCTTCGAGCTGCCGCAGCCGGAG
CGGGAGCGGTACATGTCCGCCGACGTGCGGGCGCCGGTGAGGTACGGCACCAGCTTCAACCAGGTGAGGG
ACGCCGTGCTGTGCTGGCGCGACTTCCTCAAGCTCGCCTGCATGCCGCTCGCCGCCGTCGTCGAGTCATG
GCCCACCTCGCCGGCCGACCTGCGGGAGGTGGCGTCGAGGTACGCGGAGGCGAACCAGCGCGTGTTCATG
GAGGTGATGGAGGCGGCGCTGGAGGCGCTGGGCGTGGGCGGCGGCGGCGTGATGGAGGACCTGGCGGCGG
GGACACAGATGATGACGGTGAACTGCTACCCGGAGTGCCCGCAGCCGGAGCTGACGTTGGGGATGCCGCC

GCACTCGGACTACGGCTTCCTCACGCTGGTGCTCCAGGACGAGGTGGCCGGACTCCAGGTGATGCACGCC
GGCGAGTGGCTCACCGTCGACCCGCTCCCGGGCTCCTTCGTCGTCAACGTCGGCGACCACTTGGAGATAT
TGAGCAATGGACGGTACAGGAGCGTGCTGCACAGGGTGAAGGTGAACTCGAGGAGGCTTCGGGTGTCGGT
GGCGTCGTTCCACAGCGTGGCGCCGGAGAGGGTGGTGTCGCCGGCGCCGGAGCTCATCGACGACCGCCAC
CCGCGCCGCTACATGGACACCGACCTCGCCACCTTCCTCGCCTACCTCGCCTCCGCCGCCGGCAACCACA
AGTCCTTCCTCCACTCCAGGAGGCTCTACTGA
>XM_015778633.2_*Oryza_sativa*
ATGGCTCCAGCCATTGCCAAGCCTCTCCTGAGCGATCTGGTGGCACAATCCGGGCAAGTCCCCTCGAGCC
ACATTCGTCCGGTTGGCGACCGCCCGGACCTCGACAACGTCGACCACGAGTCCGGCGCCGGCATTCCGGT
CATCGACCTGAAACAGCTCGACGGCCCGGATCGCCGCAAGGTTGTCGAGGCCATCGGTTCGGCGTGCGAA
ACCGACGGTTTTTTCATGGTGAAGAATCACGGGATCCCGGAGGAGGTGGTGGAAGGGATGCTGCGCGTGG
CGAGGGAGTTCTTCCACATGCCGGAGTCGGAGCGGCTCAAGTGCTATTCCGACGACCCCAAGAAGGCGAT
CCGGCTGTCGACGAGCTTCAACGTGCGCACCGAGAAGGTGAGCAACTGGCGCGACTTCCTGCGCTTGCAT
TGCTACCCTCTCGAGAGCTTCATCGACCAGTGGCCCTCCAACCCACCCTCCTTCAGGCAAGTGGTCGGCA
CCTACTCGAGGGAGGCGAGGGCGCTGGCGCTGCGGTTGCTGGAGGCGATATCTGAGAGCCTCGGGCTGGA
GAGGGGCCACATGGTGTCGGCCATGGGGCGGCAGGCGCAGCACATGGCGGTGAACTACTATCCGCCATGC
CCACAGCCGGAGCTCACCTACGGCCTGCCGGGGCACAAGGACCCCAATGCCATCACGCTGCTGCTCCAGG
ACGGCGTCTCCGGCCTGCAGGTCCAGCGCAACGGCCGCTGGGTGGCCGTCAACCCCGTGCCCGACGCCCT
GGTCATCAACATCGGAGATCAAATCCAGGCGCTGAGCAACGACCGGTATAAGAGCGTGCTCCACCGGGTG
ATCGTGAACAGCGAGAGCGAGAGGATCTCCGTGCCGACGTTCTACTGCCCGTCCCCGGACGCGGTGATCG
CGCCGGCCGGCGCGCTGGTGGACGGCGCCCTGCACCCGCTGGCGTACCGGCCCTTCAAGTACCAGGCCTA
CTACGACGAATTCTGGAACATGGGCCTCCAGTCCGCCAGCTGCTTAGACCGGTTCCGGCCTAACGATCAG
GCCGTCTGA
>XM_003581516.4_*Brachypodium_distachyon*
ATGGCTACGGCGATTGCCAAACCTCTCCTGAGTGATCTGGTGGCAGAATCAGGGACAGTCCCGTCGAGCC
ACATTAGACCGGTGGGAGACCGGCCAGACCTCGCCGATGTCGACCACGAGTCCGGCGCCGGCATTCCGCT
CATCGACCTGAAGCACCTCGACGGGCCGGAACGTCGAAGGGTCGTCGAAGCCATCGGCTCGGCGTGCGAG
ACCGACGGGTTTTTCATGGTGACGAATCATGGCATCCCGGAGGCGGTTGTCGAGGGGATGCTGCGCGTGG
CAAAGGAGTTCTTCCACCTGCCGGAGTCGGAGCGGCTCAAGTGCTACTCCGATGACCCGAAGAAGGCGAT
CCGGCTGTCCACGAGCTTCAACGTGCGCACGGAGAAGGTGAGCAACTGGCGAGATTTCCTCCGCCTGCAT
TGCTACCCTCTCCAGAGCTTCATCGACCAGTGGCCCTCCAACCCGCCCGCCTTCAGGGAAGTGGTGGGCG
CCTACTCGACGGAAGCGAGAGCGCTGGCGCTGAGGCTGCTGGAGGCGATCTCGGAGAGCCTGGGGCTGGA
GAGACGCCACATGGTGACGGCCATGGGCGGGCACGCGCAGCACATGGCGGTGAACTACTACCCGCCGTGC
CCGCAGCCGGAGCTCACCTACGGGCTGCCGGGCCACAAGGACCCCAATGCCGTCACGCTGCTCCTCCAGG
ACGGCGTCTCCGGCCTCCAGGTCCAGCGCGGCGGCCGCTGGGTCGCCGTCAACCCCGTGCCCAACGCGCT
CGTCATCAACATCGGTGACCAACTACAGGCGCTGAGCAACGACCGATACAAGAGCGTGCTCCACCGCGTG
ATTGTGAACAGCGAGAGCGAGAGGATCTCGGTGCCGACGTTCTACTGCCCGTCGCCGGACGCGGTGGTCG
CGCCGGCGGAAGCGCTGGTGGATGGCAGCCATCCTCTGGCGTACCGGCCCTTCACGTACCAGGAGTACTA
CGAGGAGTTCTGGAACATGGGCCTCGAGTCGGCTAGCTGCCTCGACAGGTTCCGACCGATGGACTAA
>XM_002459812.2_*Sorghum_bicolor*
ATGGCAATTCTCGACGTCGACCTGGCCACTCCTCCCTGTCATGCTCATGCTCGTCAGCAGCAGGCACACG
CAGACGAGCAGCAGGACGCCGGAGAGGAGGAGGACGAGCAGCTGCTGCTCCTCAAGGGCGTGCGACACCT
GTGCGAGCGCGGCACCATTACGAAGCTCCCCGCTCGCTACGTCCTGCCGCCCTCCGACCGTCCATCACCC
GGCGACGACGAGCAGCCACCACGCAGCAGCTGCGTGTCGTTCCCCGTCATCGACCTGGCCCGGCTGCGCG
TGCCGGCGGAGCGCGCCGCCGCCCTGGCCGAGCTGGACGCGGCCTGCAGGGACTACGGCATCTTCCAGGT
GGTTGGCCACGGCGTCGACGGCGGCGGCGGCGGCGCGATGCTGGACGTGGCGCGCCGCTTCTTCGACCTG
CCCTTCGAGGAGCGCGCGCGCTACATGTCCGCCGACATCCGTGCCCCCGTGCGCTACGGCACCAGCTTCA
ACCAGCTCAACGACGGCGTCCTCTGCTGGCGAGACTTCCTCAAGCTCGTCTGCTCCGACGACCTCGCCGG
CGTCGTCCCGTCGTGGCCAGACGCGCCCACTGATCTCAGGAAGGTGGTGTCGGCGTACGCGCGGTCGAGC
CGGCGGCTGTTCAAGGAGCTCATGGAGGCGTCGCTGGAGGCGCTAGGGATCCGGTCCGGCGCCGCCGCCG
AGATGCTGGCGGACTGCGACGCCGGGTCGCAGATGCTCATGGTCAACTGCTTCCCGGCGTGCCCGGAGCC
GGACCTCACGCTGGGGATGCCGCCGCACTCGGACTACGGCTTCCTCACCGTGCTCCTGCAGGACCAGGTG
AATGGCCTCGAGGTCAGGCACGCCGACAGGTGGGTCCTCGTCGACCCTCTCCCCGGCTCAGTCGTCGTCA
ACATCGGAGACCACTTCGAGATGTACAGCAACGGGCGGTACAAGAGCGTGCTGCACCGGGTCCGCGTGAA

CTCGTCTCGGCCGCGCATCTCGGTGGCGTCGCTGCACGGCCTGCCGCCGGAGAGGGTGATCGGGCCGGCG
CCGGAGCTGGTGGACGACGACAAGAACCCGAGGATGTACATGGACACGGACTTCGCCACCTTCCTCGAAT
ACCTCACCTCGGCCGAGGGCAAGCACAAGTCCTTCCTCCACACCAGGAGACTCATCACCAGCTGA
>XM_002448337.2_*Sorghum_bicolor*
ATGGCCCCAGCCATTTCCAAGCCTCTCCTCAGCGATCTCGTGGCACAGATCGGGAAAGTCCCATCGAGCC
ACATCAGGCCTGTGGGAGACCGCCCGGACCTCGCCAATGTCGACAACGAGTCCGGCGCCGGGATCCCGCT
CATCGACCTCAAGATGCTCAACGGGCCGGAGCGCCGTAAGGTGGTGGAGGCCATCGGCAGGGCCTGCGAA
TCCGACGGCTTCTTCATGGTGACGAACCACGGCATCCCGGCGGCGGTGGTGGAGGGGATGCTGCGCGTGG
CGAGGGAGTTCTTCCACCTGCCGGAGTCGGAGCGGCTCAAGTGCTACTCCGACGACCCCAAGAAGGCGAT
CCGGCTGTCCACCAGCTTCAACGTGCGCACGGAGAAGGTGAACAACTGGCGCGACTTCCTGCGCCTGCAT
TGCTACCCGCTCGAGAGCTTCGTCGACCAGTGGCCGTCAAACCCGCCATCCTTCAGGCAAGTGGTGGGCA
CCTACGCGACGGAAGCGAGGGCGCTAGCGCTGAGGCTGCTGGAGGCCATATCGGAGAGCCTGGGCCTGGA
GCGGAGCCACATGGTGCGCGCCATGGGGAGGCACGCGCAGCACATGGCGGTGAACTACTACCCGCCGTGC
CCGCAGCCGGAGCTCACCTACGGGCTGCCGGGCCACAAGGACCCCAATGCCATCACGCTGCTGCTCCAGG
ACGGCGTCTCCGGCCTGCAGGTGCAGCGCGGCGGCCGATGGGTGGCCGTGAACCCCGTGCCCGACGCGCT
GGTCATCAACATCGGAGACCAGATGCAGGCACTGAGCAACGACCGATACAAGAGCGTGCTCCACCGCGTG
ATCGTCAACAGCGAGAGCGAGCGGATCTCGGTGCCGACGTTTTACTGCCCGTCGCCGGACGGGGTGATCG
CGCCGGCCGACGCGCTGGTGGACGACGCCCACCCTCTGGCCTACCGCCCTTTCACTTACCAGGAGTACTA
CGACGAGTTCTGGAACATGGGCCTCCAGTCGGCAAGCTGCCTCGACCGGTTTAGGCCCGGAGGATCCATA
GAGTGA
>XM_002449303.1_*Sorghum_bicolor*
ATGGCAGCTATTGTTGAAGATCGTCAACTGCAGGTTGCCGCCGAGAGAGAAGCTGCCAAGCCGGTATCAG
CGTTACCGACGAAGCTGGAGTTGGAGCGCGGAGGCACAGGTGGTGCCGTCGTTAACCGCGGGACTGAAGA
GGAGGGCTCGTACTGCCTGATTAAAAACGTGAGGCACCTCAGCAACCGTGGCTTTACCAAGCTGCCAGAA
AGGTACGTCCTGCCGGACCCCGACCGCCCCGGCGACGTGCTCGGCAGGGTGAAGCTCCCCGTCGTCGACC
TGGCGCGCCTCCGCGATCCTGCCCACCGTGCTTCCGAGCTGGAGACGCTCGACGCCGCGTGCCGCCAATC
TGGGTTCTTTCAGGTGGTGAACCATGGCGTGACGCGCGAGCTGATCGACGGGTTGCTGGACGTGGCGCGG
CGGTTCTTCGAGCTTCCGTTGGCACGCCGGGCGCGGTACATGTCGCCGGACGTGCGGGCGCCGGTGAGGT
ACGGCACCAGCTTCAACCAGGCCAAAGATGCCGTGCTCTTCTGGCGCGACTTCCTCAAGCTAGGCTGCCA
GCCGCTACACGCCGTCGTCGCATTGTGGCCTGACGAACCCGCTGATCTCAGGGAAGTGGCGGCCAGGTAC
GCCATGGCGAACCATCAGTTGTTCATGGAGCTCATGGAGGCAGCACTAGAGGCTCTGGGCATCCCCTGCC
GTCACAGTCAGAGTCTGCTAGGCGAGCTGGAGGCTGGGTACTCGCAGATTATGCTCAACTGCTACCCGGC
GTGCCCGCAACCGGAGCTCACGCTTGGGCTGCCGCCGCACTCTGACTACTGTCTCCTCACGCTCCTGCTT
CAGGACCAAGTCCAGGGTCTCCAGATCATGCACCTCGGCCACTGGCTCACCGTCCATGCCGTCCCAGGGT
CCATCATCGTCAACGTCGGCGACCACCTCGAGATCTACAGCAACGGACTGTACAAGAGCAAGTTGCACCG
GGTGCGGGTGAACTCAACGCAGGCACGCATCTCGGCGGCGTCCTTCCACAGCGTCCCAGTGGAGCGGGTG
ATTGGGCCAGCGGCGGAGCTGGTCGACGAAGGAAACCCACGGCGGTACAAGGATACCGACTACGCCACCT
TCCTCAACTTCCTCGCATCCGCCGAGGGCAAGCATAAGACCTTCCTCCAGTCAAGGAAGCTGGCAGGCTA
A
>XM_020325285.1_*Aegilops_tauschii*
ATGGCTCCGGCGATCGCCGCAAAGCCTCTCCTAAGCGATCTGGTGGCGCAATCCAGGCAAGTCCCGTCGA
GCCACATCAGAGCGGTCGGAGACCGCCCGGACCTCGCCAATGTCGACCACGAGTCCGGCGCCGGCATTCC
GCTCATCGACCTGAAGCACCTCGACGGGCCGGGGCGTCGCGGGGTCGTCGAGGCCATCGGCGCGGCGTGC
GAGAACGACGGGTTTTTCATGGTGACGAATCACGGCATCCCGGAGGCGGTCGTGGAGGGGATGCTGCGCG
TGGCGAGGGAGTTCTTCCACCTACCGGAGTCGGAGCGGCTCAAGTGCTACTCCGACGACCCCAAGAAGGC
GATCCGGCTGTCCACGAGCTTCAACGTGCGCACGGAGAAGGTGAGCAACTGGCGCGACTTCCTCCGCCTG
CATTGCTACCCGCTCGAGAGCTTCGTCGACCAGTGGCCCTCCAACCCGCCGGCCTTCAGGGAAGTAGTCG
GCACCTACTCGACGGAGGCGAGAGCGCTGGCGCTGAGACTGCTGGAGGCGATATCGGAGAGCCTCGGGCT
CGAGAGAGGCCACATGGTGAAGGCCATGGGGCGGCACGCGCAGCACATGGCGGTGAACTACTACCCGCCG
TGCCCGCAGCCGGAGCTGACGTACGGACTGCCGGGGCACAAGGACCCTAATGCCGTCACGCTGCTCCTCC
AGGACGGCGTGTCCGGGCTGCAGGTCCGGCGCGACGGCCGCTGGGTGGCCGTCAGCCCCGTTCCCGGCGC
GCTGGTCATCAACATCGGTGATCAACTGCAGGCTCTGAGCAACGACCGATACAAGAGCGTGCTCCACCGG
GTGATTGTGAACAGCGAGAGCGAGAGGATCTCGGTGCCGACGTTCTACTGCCCGTCCCCGGACGCGGTGG
TCGCGCCGGCGGAGGCGCTGGTGGACGGCGGCCACCCTCTGGCCTATCGGCCCTTCACCTACCAGGAGTA

CTACGAGGAGTTCTGGAACATGGGCCTCGAGTCCGCCAGCTGCCTCGACCGCTTCCGACCGAACGCATGT
TGGACTGTCCAACCGGCTGGCTGTGGGCAACAAAATCTCACGGGCCACAAATGTTGCTTGCTACCGATGA
TGTGTCGCCATACGGGAAGGCCTCTGAAAGACGCCCGGAGCCGTCAGATATAG
>XM_020319454.1_*Aegilops_tauschii*
ATGGCGATAGTGGGCTTGTCAAATGCCGGCGACCGCCTGCCTGGCAAGAGAGCGGTCGGCGACGACGATG
AAGCTGCCGCAGCCGACTACCGCCTCAAAGGCGTGAGGCACCTCTCCGACGCCGGCATTACGAGGCTTCC
AGGCAGGTACGTCCTGCCGGCCTCGGATCGCCCTGGCCGGAGCGTCAGCGCGGGCACGAGGGTGAAGCTC
CCCGTCGTGGACCTCGGGCGCCTCCGCGTGCCCTCAGAGCGAGCCGCCGTGTTGAAAACTCTCGAGGCCG
CGTGCCGGGAGTTCGGCTTCTTCCAGGTGGTGAACCACGGCGTGGACGTCGATGGGGCGGCCGCCAGGAT
GCTTGACGTGGCGGAGCGGTTCTTCGAGCTTCCCTTCCAGGAGCGCGCGCGTTACATGTCCGCTGATGTC
CGCGCGCCCGTTCGGTACGGTACGAGCTTCAACCAGGCCAACGACGCCGTCCTCTGCTGGCGCGACTTCC
TCAAGCTCTCCTGCGCACCGCCGCTGCGGGACGTGGTGCCGTCGTGGCCCGACTCGCCGGCGGACCTCAG
GGAGGTGGCGGCTGAGTACGCCGCGGCGAACCGGCGGGTGTTCGTTGAGGTCGTGGAGGCGGCGCTGGAG
GCCATGGGTGTCGGAGGGGGTGACGGCGTGATGGAGGAGCTGGCCACGGCTGGGTCGCACATGATGACGG
TGAACTGCTATCCGGCGTGCCCGCAGCCAGAGCTGACGCTGGGGATGCCGCCACACTCCGACTACGGCTT
CCTGACGCTGGTGCTCCAGGACGAGGTGGAGGGCCTCCAGGTTATGCATGGCGGCGAATGGCTCACCGTC
GACCCCGTCCCTGGTTCCTTCGTCGTCAACGTCGGCGACCACTTCGAGATATACAGCAACGGCCGGTATA
AGAGCGTGCTGCACCGGGTGGGCGTGAACTCGACGCGCCCGCGTATCTCGGTGGCGTCGTTCCATAGCGT
GGGGGCGGAGCGGGTGGTCGGGCCGGCGGCGGAGCTCCTCGACCAGGGCCGCGGCGGGGAACCACGGCGG
TATATGGACACCGACTTCGCCACCTTCCTGGCTTACCTCGCCTCTGCCGAGGGCAAGCACAAGACCTTCC
TCCAGTCAAGGAGGCTCGCCTTCGGCTGA
>XM_024784691.1_*Medicago_truncatula*
ATGAAGTCTTTCCAATTAGCCAATGAGTCTTCTCCACTTTCCTTAACTCAAGATTTTATCCTTCCAAAAC
ACAAAAGACCACGCCTTTCAGAGGTTACTTTTTTAGACTCAATACCAATAATTGATTTAAGTCACTATGA
TGACAAAAACCCTTCATCCATGGAAGTGGTTCACAAGATTTCAAAGGCTTGTGAGGAATTTGGTTTCTTC
CAAATTGTGAACCATGGTGTCCCTAACAAAGTTTGCACTAAAATGATGAAAGCAATTTCAAGTCTTTTTG
AGTTGCCACCAGAAGAAAGAGAACATCTGTCCTCAACAGATCCTACCAAGAATGTAAGGTTGATCAATTA
TTACCTTCAAGTTGAAGGTGAAGAAAAGGTGAAGTTATGGAGTGAATGTTTTGCTCATCAATGGTATCCT
ATTGATGATATCATTCAGCTTTTACCAGAAAAAATTGGGAATCAATACAGAGAGGCTTTCACCGAATATG
CAAAGGAGGTTGGTTCATTGGTGAGAAGGCTTTTGAGTTTGATATCAATAGGGCTTGGCTTAGAGGAGGA
CTGTTTGTTGAAGAAGTTGGGTGAGCAGCCTAGACAAAGAGCACAAGCAAATTTCTATCCACCATGTCCT
GATCCAGAATTGACCATGGGACTGCTTGAACATACTGATCTCAATGCTATCACAGTCCTATTGCAATCAG
AAGTATCTGGTCTTCAAGTTAACAAAGATGGAAAATGGATTTCTGTTCCTTGTATTCCCAATGCTTTTGT
CATCAATTTAGCTGATCAAATTGAGGTTCTAAGTAATGGAAGATATAAAAGTGTACTTCACAGAGCTGTG
ACCAACAATGTGCAACCACGAATTTCGATGGCGATGTTTTATGGACCAAACCCAGAGACAATAATTGGAC
CAATTCATGAATTGATAGATGAAGAACACCCTCCCAAGTACAGAAATTATCATTTCTCTAAGTTTCTTGA
AGAATTCTTCAACCAAGAAGGAACAAGGAGGATTGTGAAAGAAGTCTTTGAGTTGCCATGTTAG
>XM_020229602.1_*Ananas_comosus*
ATGGCACCAGCAGAGTCCAAGCTCCTCCTAACTGATCGAGCTATATCCAGCTCCAACCATGTCCCTTCCA
ACTACATTAGGCCCGTCGGCGACCGCCCAAACCTCCTTGATGTCGAATCCTCCACCTCCATCCCCCTCAT
CGACCTTCATGGATTGCTCGGCCCCGCTCGCTCTCATGTAATCAAAGAAATCGGCTCCGCATGCCGAACT
GATGGATTCTTTATGGTTAAGAATCACGGCATCCCGGAGGAAGTTGTTAGAGAGATGCTGCGAGTTGCGA
GAGAGTTCTTTCACTTACCGGAGGCAGAGAGGCTGAAGTGCTACTCCGACGATCCGTCGAGAACGACGAG
GCTGTCGACGAGCTTCAACGTGCGGACGGAGAAGGTCAGCAACTGGAGGGACTTCCTGAGGCTCCACTGC
TACCCGCTCGAGGATTTCATCCGTGAATGGCCCTGCAATCCTCCAGATTTCAAGCAAGTAGTGGGCGAAT
ATTCGAAACATGCGAGAGCGCTCGCAGTGCGACTACTGGAGGGGATATCGGAGAGCTTGGGACTAGAAAG
GGATCACATGGTGAAGGCAATGGGGAAGCAGGCACAGCACATGGCGATAAACTACTACCCGCCATGCCCA
CAGCCCGAGCTGACCTACGGTCTCCCGGGCCACAAAGACCCCAACGCCATCACCGTACTGCTCCCAGACG
GCGTCTCCGGCTTGCAGGTTCTCCGCGGCCAGAAGTGGGTGGCCGTCGAGCCCGTACCGAATGCGTTAAT
CATCAACATGGGCGATCAAATACAGGTGCTCAGCAATGACCGCTACAAGAGCGTACTTCACCGCGTGATC
GTAAACAACAAAAGCGAGAGGATCTCCGTCCCGACCTTCTACTGTCCATCTCCGGACGCGGTTATAGAAC
CAGCTGAGGCTCTCGTCGACGACGATCATCCTCTCAGATATAGAAGTTTCACTTACGGAGAGTACTACGA
GAAGTTCTGGAACCGCGAGCTCCTATCTGCTAGTTGCCTCGATTTGTTCAAAGCGAGCCAGTGA
>XM_003613062.2_*Medicago_truncatula*

ATGGAGTCTTTCCAATTAGCCAATGAATCTTCTCCCCTTTCCTTAACTCCTAATTTTATCCTCCCAGAAC
ACAAAAGACCACACCTTTCAGAGGTTAAATATTTAGACTCAATTCCCATAATTGATCTAAGTTATTGTGA
TGGCAACAACCCTTCATCCTTGGAAGTGATTCACAAGATTTCAAAGGCTTGTGAAGAATTTGGTTTCTTC
CAAATTGTGAACCATGGTGTTCCTGACCAAGTTTGCACTAAAATGATGAAAGCAATTACCAACTTCTTTG
AGTTGGCACCAGAGGAAAGAGAACATCTTTCATCAACAGATAATACCAAGAATGTAAGGTTGTTCAATTA
TTACCTTCAAGTGGATGGTGGAGAAAAGGTTAAGTTGTGGAGTGAATGTTTTGCTCATCCATGGTATCCT
ATTGATGATATCATTCAGCTTTTACCAGAAAAAATTGGGACTCAATACAGAGAGGCTTTCACTGAATATG
CAAAAGAGGTTGGTTCATTGGTGAGAAGGCTTTTGAGTTTGATATCAATAGGGCTTGGCTTAGAGGAGGA
TTGTTTGTTGAAGAAGTTGGGTGAGCAGCCTAGACAAAGAGCACAATCAAATTTCTATCCACCATGTCCT
GATCCAGAATTGACCATGGGTCTCAATGAACATACTGATCTTAATGCTCTCACAGTTCTTTTGCAATCAG
AAGTATCTGGTCTTCAAGTTAACAAAGATGGGAAATGGATTTCTATTCCTTGTATTCCCAATGCTTTTGT
CATCAATTTAGCTGATCAAATTGAGGTTCTAAGTAATGGAAGATATAAAAGTGTACTTCACAGAGCTGTG
ACCAACAATGTGCAACCACGAATTTCGATGGCGATGTTTTATGGACCAAACCCAGAGACAATAATTGGAC
CAATTCATGAATTGATAGATGAAGAACACCCTCCCAAGTACAGAAATTATCATTTCTCTAAGTTTCTTGA
AGAATTCTTCAACCAAGAAGGAACAAGGAGGATTGTGAAAGAAGTCTTTGAGTTGCCATGTTAG
>XM_024784690.1_*Medicago_truncatula*
ATGAAGTCTTTCCAATTAGCCAATGAGTCTTCTCCACTTTCCTTAACTCAAGATTTTATCCTTCCAAAAC
ACAAAAGACCACGCCTTTCAGAGGTTACTTTTTTAGACTCAATACCAATAATTGATTTAAGTCACTATGA
TGACAAAAACCCTTCATCCATGGAAGTGGTTCACAAGATTTCAAAGGCTTGTGAGGAATTTGGTTTCTTC
CAAATTGTGAACCATGGTGTCCCTAACAAAGTTTGCACTAAAATGATGAAAGCAATTTCAAGTCTTTTTG
AGTTGCCACCAGAAGAAAGAGAACATCTGTCCTCAACAGATCCTACCAAGAATGTAAGGTTGATCAATTA
TTACCTTCAAGTTGAAGGTGAAGAAAAGGTGAAGTTATGGAGTGAATGTTTTGCTCATCAATGGTATCCT
ATTGATGATATCATTCAGCTTTTACCAGAAAAAATTGGGAATCAATACAGAGAGGCTTTCACCGAATATG
CAAAGGAGGTTGGTTCATTGGTGAGAAGGCTTTTGAGTTTGATATCAATAGGGCTTGGCTTAGAGGAGGA
CTGTTTGTTGAAGAAGTTGGGTGAGCAGCCTAGACAAAGAGCACAAGCAAATTTCTATCCACCATGTCCT
GATCCAGAATTGACCATGGGACTGCTTGAACATACTGATCTCAATGCTATCACAGTCCTATTGCAATCAG
AAGTATCTGGTCTTCAAGTTAACAAAGATGGAAAATGGATTTCTGTTCCTTGTATTCCCAATGCTTTTGT
CATCAATTTAGCTGATCAAATTGAGGTTCTAAGCAATGGAAGATACAAAAGTGTAATTCACAGAGCTGCC
ACAAATAATGTGCATCCACGAATGTCGATGGCAATGTTTTTCGGGCCAAACCCAGAGACAATAATTGAGC
CAATTCATGAATTAATAGATGATGAACACCCTCCCAAGTACAGAAGTTATCGTTTCTCTAAGTTTCTTGA
AGAAGTCTTCAACCATAAAGGAACAAGGAGGATCGTGAAAGAAACCTTTGAGTTGCCACGTTAG
>XM_030597820.1_*Syzygium_oleosum*
ATGGGCGCGGTCAAAGATCAGGGACCAACACCGACAGAGAGCCAATTCCAGCACGGCGTGAAGCATCTTC
ACGAGAGTGGCCTCAAAAGACTGCCGACCAAGTACATACTGCCCCTCCAAGACCGGCCGAAACCCGAGCC
CGCCGAGCCGAGCCTGAAGCTCCCCGTCATCGACCTCGCACAGCTGCAGGGCCCAGCCCGGCCGGGGGCC
ATCGCGTCCATCGCCGATGCGTGCAAGAGGTACGGCTTCTTCCAGGTGGTCAACCACAGGGTCACGAGGG
GCACGGCGGAGGGCATGGCGGACGCAGGGCGGAGGTTCTTCGGGATGCCCTTCGAGGAGAGGGCCAAGTA
CATGTCGGCGGACATGTTCGCGCCGGTTCGCTACGGGACGAGCTTCAACCAGAGGAAGGACGATGTGTTC
TGCTGGAGGGATTTCCTGAAGCTGACGTGCGAGCCCATGCCGGACGTCCTTACGCACTGGCCTGCTTCCC
CTGCTGACCTGAGGAAAGTGGCGGCTAGCTACGCGAAAGAGATCCGGGACTTGTTTCTGGTGCTGATGGA
GGCCATCTTGGAAGGCCTGCATGTGTCCAATACAGAGGAGAGGAAGAAGATGAAACCTGGGAACGAAGGA
GGAGAAGAAGATGATGGTGATGATATCATGGAGAAGTTAAAAAATGGAAGCCAACTCATGGTGGTGAACT
GTTTCCCTCCATGCCCGGAGCCTGATTTGACGCTTGGGCTGCCTCCACACTCCGACTATGGCTTCCTCAC
CCTCCTCCTCCAAGATGAAGTCCAAGGCCTGCAGATTCAGTTCCAAGAGAGGTGGGTCACTGTGGAACCC
ATCGAGAACGGGTTCGTCGTGAATGTCGGCGACCATTTGGAGATTTTCAGCAATGGGAGGTACAAGAGCG
TTCTCCACAGAGTCCTCGTGAACTCTGAGAGATCTCGAATCTCAGTGGCTTCCTTGCACAGCCTTCCTTA
CGAGTGCATGGTGAGGCCGTCCTCGAAGCTCATCGACAAAGCGAACCCAAAGCGCTACAAGGACACCAAC
TTCGGGAGCTTTCTCGAGTACATATCGTCTCGCGAGCCAAAGAAGAAGAAATTCTTGGAGACCCGGAAGT
TGAATTGA
>XM_030597821.1_*Medicago_truncatula*
ATGGGCGCGGTCAAAGATCAGGGACCAACACCGACAGAGAGCCAATTCCAGCACGGCGTGAAGCATCTTC
ACGAGAGTGGCCTCAAAAGACTGCCGACCAAGTACATACTGCCCCTCCAAGACCGGCCGAAACCCGAGCC
CGCCGAGCCGAGCCTGAAGCTCCCCGTCATCGACCTCGCACAGCTGCAGGGCCCAGCCCGGCCGGGGGCC
ATCGCGTCCATCGCCGATGCGTGCAAGAGGTACGGCTTCTTCCAGGTGGTCAACCACAGGGTCACGAGGG

GCACGGCGGAGGGCATGGCGGACGCAGGGCGGAGGTTCTTCGGGATGCCCTTCGAGGAGAGGGCCAAGTA
CATGTCGGCGGACATGTTCGCGCCGGTTCGCTACGGGACGAGCTTCAACCAGAGGAAGGACGATGTGTTC
TGCTGGAGGGATTTCCTGAAGCTGACGTGCGAGCCCATGCCGGACGTCCTTACGCACTGGCCTGCTTCCC
CTGCTGACCTGAGGAAAGTGGCGGCTAGCTACGCGAAAGAGATCCGGGACTTGTTTCTGGTGCTGATGGA
GGCCATCTTGGAAGGCCTGCATGTGTCCAATACAGAGGAGAGGAAGAAGATGAAACCTGGGAACGAAGGA
GGAGAAGAAGATGATGGTGATGATATCATGGAGAAGTTAAAAAATGGAAGCCAACTCATGGTGGTGAACT
GTTTCCCTCCATGCCCGGAGCCTGATTTGACGCTTGGGCTGCCTCCACACTCCGACTATGGCTTCCTCAC
CCTCCTCCTCCAAGATGAAGTCCAAGGCCTGCAGATTCAGTTCCAAGAGAGGTGGGTCACTGTGGAACCC
ATCGAGAACGGGTTCGTCGTGAATGTCGGCGACCATTTGGAGATTTTCAGCAATGGGAGGTACAAGAGCG
TTCTCCACAGAGTCCTCGTGAACTCTGAGAGATCTCGAATCTCAGTGGCTTCCTTGCACAGCCTTCCTTA
CGAGTGCATGGTGAGGCCGTCCTCGAAGCTCATCGACAAAGCGAACCCAAAGCGCTACAAGGACACCAAC
TTCGGGAGCTTTCTCGAGTACATATCGTCTCGCGAGCCAAAGAAGAAGAAATTCTTGGAGACCCGGAAGT
TGAATTGA
>XM_030597822.1_*Syzygium_oleosum*
ATGGGCGCGGTCAAAGATCAGGGACCAACACCGACAGAGAGCCAATTCCAGCACGGCGTGAAGCATCTTC
ACGAGAGTGGCCTCAAAAGACTGCCGACCAAGTACATACTGCCCCTCCAAGACCGGCCGAAACCCGAGCC
CGCCGAGCCGAGCCTGAAGCTCCCCGTCATCGACCTCGCACAGCTGCAGGGCCCAGCCCGGCCGGGGGCC
ATCGCGTCCATCGCCGATGCGTGCAAGAGGTACGGCTTCTTCCAGGTGGTCAACCACAGGGTCACGAGGG
GCACGGCGGAGGGCATGGCGGACGCAGGGCGGAGGTTCTTCGGGATGCCCTTCGAGGAGAGGGCCAAGTA
CATGTCGGCGGACATGTTCGCGCCGGTTCGCTACGGGACGAGCTTCAACCAGAGGAAGGACGATGTGTTC
TGCTGGAGGGATTTCCTGAAGCTGACGTGCGAGCCCATGCCGGACGTCCTTACGCACTGGCCTGCTTCCC
CTGCTGACCTGAGGAAAGTGGCGGCTAGCTACGCGAAAGAGATCCGGGACTTGTTTCTGGTGCTGATGGA
GGCCATCTTGGAAGGCCTGCATGTGTCCAATACAGAGGAGAGGAAGAAGATGAAACCTGGGAACGAAGGA
GGAGAAGAAGATGATGGTGATGATATCATGGAGAAGTTAAAAAATGGAAGCCAACTCATGGTGGTGAACT
GTTTCCCTCCATGCCCGGAGCCTGATTTGACGCTTGGGCTGCCTCCACACTCCGACTATGGCTTCCTCAC
CCTCCTCCTCCAAGATGAAGTCCAAGGCCTGCAGATTCAGTTCCAAGAGAGGTGGGTCACTGTGGAACCC
ATCGAGAACGGGTTCGTCGTGAATGTCGGCGACCATTTGGAGATTTTCAGCAATGGGAGGTACAAGAGCG
TTCTCCACAGAGTCCTCGTGAACTCTGAGAGATCTCGAATCTCAGTGGCTTCCTTGCACAGCCTTCCTTA
CGAGTGCATGGTGAGGCCGTCCTCGAAGCTCATCGACAAAGCGAACCCAAAGCGCTACAAGGACACCAAC
TTCGGGAGCTTTCTCGAGTACATATCGTCTCGCGAGCCAAAGAAGAAGAAATTCTTGGAGACCCGGAAGT
TGAATTGA
>XM_030597823.1_*Syzygium_oleosum*
ATGGGCGCGGTCAAAGATCAGGGACCAACACCGACAGAGAGCCAATTCCAGCACGGCGTGAAGCATCTTC
ACGAGAGTGGCCTCAAAAGACTGCCGACCAAGTACATACTGCCCCTCCAAGACCGGCCGAAACCCGAGCC
CGCCGAGCCGAGCCTGAAGCTCCCCGTCATCGACCTCGCACAGCTGCAGGGCCCAGCCCGGCCGGGGGCC
ATCGCGTCCATCGCCGATGCGTGCAAGAGGTACGGCTTCTTCCAGGTGGTCAACCACAGGGTCACGAGGG
GCACGGCGGAGGGCATGGCGGACGCAGGGCGGAGGTTCTTCGGGATGCCCTTCGAGGAGAGGGCCAAGTA
CATGTCGGCGGACATGTTCGCGCCGGTTCGCTACGGGACGAGCTTCAACCAGAGGAAGGACGATGTGTTC
TGCTGGAGGGATTTCCTGAAGCTGACGTGCGAGCCCATGCCGGACGTCCTTACGCACTGGCCTGCTTCCC
CTGCTGACCTGAGGAAAGTGGCGGCTAGCTACGCGAAAGAGATCCGGGACTTGTTTCTGGTGCTGATGGA
GGCCATCTTGGAAGGCCTGCATGTGTCCAATACAGAGGAGAGGAAGAAGATGAAACCTGGGAACGAAGGA
GGAGAAGAAGATGATGGTGATGATATCATGGAGAAGTTAAAAAATGGAAGCCAACTCATGGTGGTGAACT
GTTTCCCTCCATGCCCGGAGCCTGATTTGACGCTTGGGCTGCCTCCACACTCCGACTATGGCTTCCTCAC
CCTCCTCCTCCAAGATGAAGTCCAAGGCCTGCAGATTCAGTTCCAAGAGAGGTGGGTCACTGTGGAACCC
ATCGAGAACGGGTTCGTCGTGAATGTCGGCGACCATTTGGAGTTTTCACTTGGAAATCTTCCATTTACGT
AG
>XM_008785318.3_*Phoenix_dactylifera*
ATGTCTTCTCCCATGGCCACTACTGTTCCTACCAAGGTTCTCCTAAGCGATCTTGTTTCTTCCTTTGTTG
AATACGTCCCCTCCAACTACATCAGGCCAGCGTCCGACCGTCCTGACCTGCTCAATGTGGAGACATCGGA
TACGACGATTCCTATCGTAGACCTTCAAGGCCTCGCCGGCACTAATCGCTCCCGGGTAGTTGAAGAAATT
GGAATAGCATGCGAGAATGACGGATTCTTCCAGGTTACGAATCATGGCATCCCAAAGGATGTCATTGAAG
GCATGCTACGTGTTGCGAAGGAATTCTTTCATTTGCCGGAGTCTGAAAGGTTGAAGAGCTACTCCGACGA
TCCTGCAAAGACGACCAGGCTCTCGACCAGCTTCAACGTGAAGACCGAGAAAGCTAGCAGCTGGAGGGAT
TACTTAAGACTCCATTGCTACCCTCTCGAGAATTTCGTGGACGAGTGGCCCTGCAATCCTCCATGTTTA

GGCAAGTTGTCGCCGAGTACTGCAAGAACGCGAGAGAGCTGGTCTTGAGACTGTTAGAGGCCATCTCCGA
AAGCTTGGAGCTTGAGGGTGACTACATGGTGAAGGCACTGGGGAAGCAGGCACAACACATGGCTATAAAC
TACTATCCACCATGCCCCCAGCCGGAGCTGACCTACGGCCTGCCGGGCCACAAAGACCCCAACGCCATTA
CCATTCTTCTCCAAGATGGAGTCCCTGGTTTGCAAGTCCTTAGGAAGGGGAAGTGGGTGACCGTCAACCC
AGACCCAGATACCTTGGTCATCAACATCGGCGACATGATACAGGTTCTCAGTAATGACCGTTTTAAAAGT
GTGCTTCATCGTGTGGTTGTGAGTAAGACAAGCGAGAGGATCTCTGTCCCTACTTTCTATTGCCCATCTC
CAGATGCATTAATTAAACCAGCAGAAGTGGTAGTTGAAAAAGGGCATCCACCTATCTATCGAAGCTTCAC
TTATGCGGAGTATTATGAGAAATTTTGGAATCAAGGACTCCAATCTGCGAGCTGGCTCGACGTGTTTAAA
GCCACCTGA
>XM_008785317.3_*Phoenix_dactylifera*
ATGTCTTCTCCCATGGCCACTACTGTTCCTACCAAGGTTCTCCTAAGCGATCTTGTTTCTTCCTTTGTTG
AATACGTCCCCTCCAACTACATCAGGCCAGCGTCCGACCGTCCTGACCTGCTCAATGTGGAGACATCGGA
TACGACGATTCCTATCGTAGACCTTCAAGGCCTCGCCGGCACTAATCGCTCCCGGGTAGTTGAAGAAATT
GGAATAGCATGCGAGAATGACGGATTCTTCCAGGTTACGAATCATGGCATCCCAAAGGATGTCATTGAAG
GCATGCTACGTGTTGCGAAGGAATTCTTTCATTTGCCGGAGTCTGAAAGGTTGAAGAGCTACTCCGACGA
TCCTGCAAAGACGACCAGGCTCTCGACCAGCTTCAACGTGAAGACCGAGAAAGCTAGCAGCTGGAGGGAT
TACTTAAGACTCCATTGCTACCCTCTCGAGAATTTCGTGGACGAGTGGCCCTGCAATCCTCCATGTTTTA
GGCAAGTTGTCGCCGAGTACTGCAAGAACGCGAGAGAGCTGGTCTTGAGACTGTTAGAGGCCATCTCCGA
AAGCTTGGAGCTTGAGGGTGACTACATGGTGAAGGCACTGGGGAAGCAGGCACAACACATGGCTATAAAC
TACTATCCACCATGCCCCCAGCCGGAGCTGACCTACGGCCTGCCGGGCCACAAAGACCCCAACGCCATTA
CCATTCTTCTCCAAGATGGAGTCCCTGGTTTGCAAGTCCTTAGGAAGGGGAAGTGGGTGACCGTCAACCC
AGACCCAGATACCTTGGTCATCAACATCGGCGACATGATACAGGTTCTCAGTAATGACCGTTTTAAAAGT
GTGCTTCATCGTGTGGTTGTGAGTAAGACAAGCGAGAGGATCTCTGTCCCTACTTTCTATTGCCCATCTC
CAGATGCATTAATTAAACCAGCAGAAGTGGTAGTTGAAAAAGGGCATCCACCTATCTATCGAAGCTTCAC
TTATGCGGAGTATTATGAGAAATTTTGGAATCAAGGACTCCAATCTGCGAGCTGGCTCGACGTGTTTAAA
GCCACCTGA
>XM_008777619.3_*Phoenix_dactylifera*
ATGATGGCTGTCCCCTCCAAGCTCCTCCTTGCTGACATAGCCCAAAACTGTAGCTCCATCCCCTCCAACT
TCATTCGTCCAATCTCCGACCGCCCCAATCTACATGACGTGAAGACCTCAGACGCGTCAATCCCCCTCAT
AGACCTTCAAGGCCTCACCGGCCCCGGCCGCAGCAAACTTATCAAAGAAATTGGTGCTGCATGCCTGAAG
GATGGATTCTTCCAAGTCAAGAACCATGGCATCCCTGAGGCGGTGATCGACGACATGTTATGTGTGTCAA
AGGAATTCTTCCATTTGCCCGAGTCTGAAAGACTGAAGAAGTACTCCGATGACCCCATGAAGACGTGTAG
ACTCTCCACCAGCTTCAACATCAAAACAGAAAAGGTTGGCAGCTGGAGGGACTACTTGAGGATCCATTGC
TACCCTCTCGAAGATTTCGTTCATGAGTGGCCCGACAGCCCTCCATGCTTCAGACAAGTCGTCAGTGAGT
ACTGTAAAAGTGTGAGAGAGCTGGCTCTTCGAGTTCTGGAGGCCATTTCAGAGAGTTTGGATCTGGAAAG
GGACTTCATGAATAAGGTATTAAGTAGCCATGCACAGCACATGGCCATAAACTACTACCCACCATGCCCG
CAACCGGAGCTCACCTACGGATTACCAGGCCACAAAGATCCCAACGTCATCTCTCTCCTGATCCAAGATG
GAGTTTCTGGCCTGCAAGCGCTTCGTGATGGGAAGTGGGTCGCCATTAATCCTGAACCGTATAACTTGGT
AATTAACCTCGGCGATCAGATGCAGGTACTCAGCAATGGCAAGTACAAAAGCGTGCTCCACCGAGCAGTA
GTTAACAGCAATTCTGCGAGGATCTCAATTCCTACTTTCTACTGTCCATCTTCTGATGCCGTGATCAAAC
CACCAGGATCACTTGTTGATCAAGAGCACCCTTCAATCTATAGGAGCTTCACCTATGCAGAATATTATGA
GAAGTTTTGGGACCATGGAATGAATTCCGAGAGCTGTCTCGACATGTTTAAAGCCACCTAG
>XM_026800772.1_*Phoenix_dactylifera*
ATGATGGCTGTCCCCTCCAAGCTCCTCCTTGCTGACATAGCCCAAAACTGTAGCTCCATCCCCTCCAACT
TCATTCGTCCAATCTCCGACCGCCCCAATCTACATGACGTGAAGACCTCAGACGCGTCAATCCCCCTCAT
AGACCTTCAAGGCCTCACCGGCCCCGGCCGCAGCAAACTTATCAAAGAAATTGGTGCTGCATGCCTGAAG
GATGGATTCTTCCAAGTCAAGAACCATGGCATCCCTGAGGCGGTGATCGACGACATGTTATGTGTGTCAA
AGGAATTCTTCCATTTGCCCGAGTCTGAAAGACTGAAGAAGTACTCCGATGACCCCATGAAGACGTGTAG
ACTCTCCACCAGCTTCAACATCAAAACAGAAAAGGTTGGCAGCTGGAGGGACTACTTGAGGATCCATTGC
TACCCTCTCGAAGATTTCGTTCATGAGTGGCCCGACAGCCCTCCATGCTTCAGACAAGTCGTCAGTGAGT
ACTGTAAAAGTGTGAGAGAGCTGGCTCTTCGAGTTCTGGAGGCCATTTCAGAGAGTTTGGATCTGGAAAG
GGACTTCATGAATAAGGTATTAAGTAGCCATGCACAGCACATGGCCATAAACTACTACCCACCATGCCCG
CAACCGGAGCTCACCTACGGATTACCAGGCCACAAAGATCCCAACGTCATCTCTCTCCTGATCCAAGATG
GAGTTTCTGGCCTGCAAGCGCTTCGTGATGGGAAGTGGGTCGCCATTAATCCTGAACCGTATAACTTGGT

ACTCAGCAATGGCAAGTACAAAAGCGTGCTCCACCGAGCAGTAGTTAACAGCAATTCTGCGAGGATCTCA
ATTCCTACTTTCTACTGTCCATCTTCTGATGCCGTGATCAAACCACCAGGATCACTTGTTGATCAAGAGC
ACCCTTCAATCTATAGGAGCTTCACCTATGCAGAATATTATGAGAAGTTTTGGGACCATGGAATGAATTC
CGAGAGCTGTCTCGACATGTTTAAAGCCACCTAG
>XM_008813992.3_*Phoenix_dactylifera*
ATGGCCCCTCCAATTCTCCCACACACCGCTCCCATACCCTCCCCTCCCCCCAGCTCCGTGAAAGAGCTCA
CCAACTCCACATCCCTTGGCTCCGTTCCCCCCCAGTATGCCATCCGAAACCCCGAGACCACCATGGACAT
CGAACCCATCATCGAAGAAGAAATCCCAACCGTTGACTTCTCTCTGCTGACCGAAGGAACACCTGAGCAA
AGGTCTCAGGTGGTTCGCCACCTCGGCAAGGCTTGTGAGGACTGGGGCTTCTTCATGGTCGTGAACCATG
GTATACCGGAGAGACTAAGGGAGGCGATGCTGATCTCGATGAAGGAATTCTTTGATCAGACGGAGGAGGA
GAAGGGAGAGTACACTGGCAAGCATGTGATGGATCCCATCAGGTATGGGACTAGTTTCAATTTAACGGTG
GATGATGTCAGGTACTGGAGGGACTATCTGAAAGTTTTTGTGCACCCGATTTTGTATTCACCCGCTAAGC
CTCCAAGTTTCAGGGAGATATCAAAGGAGTACGCTGCGTCCACTAGAGCAATGGGAATGGAACTTCTGAG
AGGAATATGGGAGAGCTTGGGATTAGAGGAGAATGACATGACCAAGGCCTTGGACCTCCATTCCTGTTAC
CAGGTTCTCGTGGGCAATATATACCCTCCGTGCCCACAACCCGAGCTGGCCATCGGCCTCCCCGCTCACT
CGGACCATGGCCTTCTCACCATCCTCCTGCAGAACGGCGTCAACGGACTCCAAGTGAAGCGGAGAGGCAG
CTGGGTCCGTGTCGAACCCCTCCCTAACTCGTTCCTCGTCAACACTGGCGATCAAATGGAGGTCGTCAGC
AATGGAAGGTATAAGAGTGTGCTGCACCGGGCTGAGGTGAATAGCCAAAGCACGAGGATGTCGATAGCAA
CCGTGGTGGCACCATCGCTTGATACGATTGTCGAGCCAGCCTCTCAGCTGGTGGGCGATGAGCACCCGAT
GATGTTTCGAGGCATGCGGTATGGAGAGTACTTGGAGTGTCAGCAGGCCACCCGGCTCAAGGCCAAGTCT
GCGCTGGATCCTGTAAGACTGCATGCCGAGTAG

DND1;

>AY883091.2_*Gossypium_hirsutum*
ATGCCTTCTCAGTCCAACTTCTCCCTATCAAGGTGGTTTGGACTTTTTCAACTTCCAAACTCAATGCCAG
AGAGATCTGATAATGGTAGTGTTAGCGGCGAAGGCAATGAAGAAAACCCAATTTCCTACACCGTAGAATG
TTACGCTTGTACTCAAGTCGGTGTTCCAGTTTTTCACTCCACCAGCTGTGACCAAGCTCACCCACCGGAA
TGGGAAGCCTCCGCTGGTTCTTCCCTCGTTCCAATTCAAGCTCGTACGGCCTCCAAACAGAAGAAGACTC
AACAGCCTGCCGCGCCTAATACTCGGCGACCTTCTGGTCCGTTCGGTCGGGTGCTTGATCCTAGGACCAA
GCGAGTGCAAAACTGGAACCGGGCTTTCTTATTGGCTCGTGCAATGGCTTTAGCCATTGATCCTTTGTTT
TTCTATGCTTTATCTATAGGAAGAGGTGGGTCGCCGTGTTTGTACATGGATGGGGGCCTCGCTGCCATCG
TAACCGTCCTCCGCACGTGTGTGGACGCCGTGCATTTGTTCCATCTTTGGCTTCAGTTCAGACTGGCGTA
CGTGTCAAGGGAGTCGCTGGTCGTCGGTTGTGGTAAACTCGTGTGGGACGCACGTGCCATCGCTTCTCAT
TACGTTCGTTCCCTCAAAGGTTTCTGGTTTGATGTCTTTGTGATTCTGCCGGTTCCTCAGGCAGTATTTT
GGTTAGTTGTACCAAAATTAATAAGGGAAGAGCAGATCAAGATTATTATGACAATACTGTTATTAATCTT
CTTGTTCCAATTCTTGCCAAAGGTTTACCACATCATTTGCTTAATGAGAAGGCTGCAAAAGGTCACCGGT
TACATCTTTGGCACCATTTGGTGGGGTTTTGGCCTTAATCTCATTGCCTACTTCATAGCCTCTCACGTTG
CTGGAGGGTGCTGGTATGTCCTTGCAATACAACGGGTAGCCTCATGTCTGCGGCAACAATGCGCGAGAAA
CAAGCAGTGCAAGCTTTCATTGTCGTGCTCGGAGGAAGTGTGCTACCAATTCTTATTTCCAGCTGAGGCA
GTAGGAAATACTTGTGGTGGTAACTCAACCAACGTTATTGGAAAACCTTTATGTTTAGAGGTTCATGGAC
CATTCAATTATGGGATATATCAGTGGGCTCTCCCTGTTGTTTCTAGCAATTCTGTTGCTGTTAGGATCCT
TTATCCCATCTATTGGGGCTTAATGTCTCTCAGCACCTTTGGGAATGATCTTGAACCAACAAGTCACTGG
TTAGAAGTGATGTTCAGTATTTGCATTGTGCTTGCTGGATTGATGCTCTTTACTTTATTGATTGGAAACA
TTCAGGTATTCTTGCATGCTGTCATGGCGAAGAAGAGGAAAATGCAGCTGAGATGTCGAGACATGGAATG
GTGGATGAAACGCCGGCAACTACCATCTTGTTTGAGACAACGAGTCCGCCATTACGAACGCCAAAAATGG
GCGACCTTGGGCGGAGAAGACGAAATGGAACTGATCAAAGACTTACCCGAAGGCCTCCGGAGAGACATTA
AACGCTTCCTTTGCCTTGACCTCATCAAGAAGGTTCCTTTATTCCATAACTTGAATGATCTTATTCTGGA
TAACATCTGTGATCGAGTTAAGCCGCTCGTATTCTCTAAAGATGAAAAGATAATTAGAGAAGGTGATCCA
GTACAAAGAATGGTGTTTGTCGTTCGTGGACGTATAAAACGTATCCAAAGCCTTAGCAAAGGCGTGGTTG
CCACAAGTTTAATCGAGTCAGGAGGCTTCCTAGGTGACGAATTGTTGTCATGGTGTCTTCGCCGACCATT
TATCAACCGTCTTCCAGCCTCGTCCGCAACATTTGTTTGTGTAGAGCCGATTGAAGCATTCAGTCTCGAC
TCAAACCATCTCAAATACATTACAGATCACTTCAGGTATAAATTTGCCAATGAGAGACTTAAAAGAACAG
CAAGATACTATTCATCGAATTGGCGAACATGGGCAGCCGTGAATATACAACTTGGCTGGCGGCGTTACAG

AACGAGGACTCGAGGTCCAATGATTTCTGCTGCCGAAAACGGCAACAGCAGCGACCGCCGGTTGCTGCAA
TACGCTGCCATGTTTATGTCAATAAGGCCACAAGATCATCTAGAATAA
>XM_004232137.4_*Solanum_lycopersicum*
ATGGCTCATTTTCTCTTAAACTCCAGGTGGTTTGGCATATTCCGACGAAGATCAGTTCAACCTGATAACA
GCGACGACAACGATGACGACATCAATCCAATCTCAAATTCCATTGAATGTTATGCATGTACTCAAGTTGG
CGTCCCTGTTTTCCACTCCACCAGTTGCGATGGAGCTAACCAACCGGAGTGGGAAGCTTCAGCCGGTTCT
TCTCTAGTTCCAATTCAAAACCGGACGGATTCAAAAACCGGAAAATCCCGGTCCAGTCGCAGCCGGCACA
CATCGGGGCCGTTCGGGCGTGTATTAGACCCTCGAAGCAAGCGCGTGCAGAGATGGAACCGAATGATTTT
ATTGGCACGTGGCATGGCTTTAGCCGTTGATCCTCTATTCTTTTACGCCTTATCCATCGGCCGCGGTGGA
TCGCCGTGTTTGTACATGGACGGCAGCCTGGCGGCTATCGTCACCGTGATTCGGACTAGCGTCGACGCCG
TGCACCTCTTCCATTTGTGGTTGCAGTTTCGTTTGGCTTACGTGTCGAGAGAATCGCTGGTGGTTGGTTG
TGGGAAACTCGTGTGGGATGCGCGTGCGATTGCTTCTCACTATGTTAGGTCCCTTAAAGGATTTTGGTTC
GATGCTTTTGTCATCCTTCCCGTTCCACAGGCTGTATTCTGGCTGGTGGTTCCAAAACTAATAAGAGAAG
AGCAGATAAAGCTTATAATGACGATCCTTTTATTAATGTTCTTGTTCCAGTTCCTTCCCAAAGTTTATCA
CTGTATAAGCTTAATGAGAAGGATGCAAAAGGTTACAGGATATATTTTTGGTACCATCTGGTGGGGATTT
GGACTTAATCTCATTGCTTATTTTATTGCTTCTCATGTTGCTGGGGGATGCTGGTATGTTCTTGCAATAC
AAAGAGTGGCTTCATGTCTAAGGCAGCAGTGTGAGCGCAACCCTTCGTGTAATCTATCTTTGTCTTGCTC
AGAGGAGGTGTGTTATCAGTTTCTGTTGCCAACAGGAACTGTGGGAAATCCATGTGCTGGGAACTCAACA
ACAGTGACCAGGAAGCCAATGTGTTTGGATGTCAATGGACCATTTCCATATGGGATATACCAATGGGCAC
TTCCTGTTGTTTCTAGCAGATCCGTCACTGTTAAGATTCTTTACCCCATCTTTTGGGGATTGATGACCCT
TAGCACATTTGGCAATGACTTAGAACCAACAAGTCACTGGCTGGAAGTTATTTTCAGTATATGCCTTGTG
CTTAGTGGATTGATGCTCTTCACTTTGCTGATTGGTAACATTCAGGTGTTTTTACACGCGGTCATGGCAA
AGAAGCGAAAAATGCAATTAAGATGTAGGGATATGGAATGGTGGATGAGGAGGAGACAATTACCATCACA
ATTAAGACAAAGAGTTCGCCACTTTGAACACCAGAGATGGGCTATGATGGGTGGCGAAGATGAGATGGAA
CTTGTAAAAGACCTGCCAGAAGGACTACGAAGGGACATCAAACGCTTTCTTTGCCTTGATCTTATTAAGA
AGGTTCCTCTGTTCGAAAGTTTGGATGATCTGATTCTAGATAACATTTGTGATCGCGTTAAGCCACTTGT
GTTCTCTAAAGATGAGAAGATCATAAGAGAAGGAGATCCAGTGCACAGGGTTGTGTTCATTGTTCGTGGA
CGTGTAAAAAGTAGCCAAAACCTCAGTAAAGGAGTGATTGCCACAAGCATACTTGAGCCTGGAGGCTTCT
TTGGAGATGAACTTCTTTCCTGGTGCTTACGCCGTCCCTTTATTGACAGACTTCCAGCTTCTTCCGCAAC
CTTCACTTGCATTGAATCTACAGAAGCATTTGGCTTAGATGCAAACCACCTTCGATTTATCACGGATCAC
TTCAGATACAAATTTGCAAACGAGAGGCTGAAGAGAACAGCAAGGTATTATTCATCCAATTGGAGAACCT
GGGCTGCTGTGAATATACAGTTAGCTTGGCGACGTTACATGATGAGGACTAGCCGTCCCACTATACATGT
GATCGAAAATGGGGATAATGATCATCGTCTTCGCAAGTATGCTGCAATGTTCTTGTCAATCAGACCACAT
GATCATCTTGAATAG
>XM_022172507.2_*Helianthus_annuus*
ATGTCTTCATTCTTCCGACTATTCCGTCACCGGAGTAACGCTTCTGATAGTGCTGACGAGCCCGAAGACG
ATGGTCCCGTCTCGTACTCAACCGAGTGTTACGCCTGCACCCAAGTAGGCGTCCCCGTCTTCCACTCCAC
CAGCTGTGACAATGTTAATCAACCCGAATGGGAAGCCTCCGCAGGCTCTTCCCTTGTCCCCATTCGCGAC
CGACCCGGTTCCAAAATTATCCGCACCCGTTCCTCCAAAGACGGCCGGAGACGCCGGCCGTCCGGGTTCC
TGAGCCGGGTGTACGACCCGCGGAGCAAACGGGTGCAGAGATGGAACCGGTTTTTTTACTGGCTCGCGG
GATGGCGTTGGCGGTAGACCCGCTGTTCTTTTACACGCTGTCCATCGGGCGCGGGGGTACGCCGTGCCTT
TACATGGACGGCGCCCTGGCGGCCGTGGTGGCGGTGGTCAGGACGTTAGTGGACGCCATACATCTTGTGC
ACATGTGGTTACAGTTTCGGATGGCTTACGTGTCACGTGAGTCGCTTGTGGTTGGGTGTGGTAAACTCGT
GTGGGACCCACAGGCTATTGCTTCTCACTATGTGCGGTCAGTAAAAGGCTTCTGGTACGACGCATTCGTC
ATACTTCCGATCCCACAGGTTGTATTTTGGTTAGTAGTGCCACGATTGATACGCGAAGAGCGAATAAAGG
CAATCATGACGACCCTCGTACTCGTTTTCATCTTCCAATTCCTGCCCAAAGTCTACCACTCGATCTCGCT
AATGAGAAGAATGGGCAAAGTCACAGGCTACATCTTTGGCACCATTTGGTGGGGTTTCGCCCTTAATCTC
ATCGCCTACTTCATCGCTTCTCACGTTGCTGGTGGTTGTTGGTATGTTTTGGCGATACAACGGGTCGTTT
CATGTCTAAAGCATCAATGTAACAAGAAAAATGCGTGTAATCTAGCATTGTCTTGTGCCGATGAGGTTTG
CTACCAATTTTTGTTACCCGAGGGGGTGTTTGGTGATCGTTGTGCTGGAAACTCAACCACGAGTTTGGTT
AGAAAGCCGTTGTGTTTGGATGTAAATGGCCCGTATCACTATGGCATTTACCGATGGGCTCTTCCGGTTA
TTTCTAGCAACTCGCTCACCGTCAAGATTCTTTATCCCATCTTTTGGGGTCTTATGAGCCTTAGTACCTT
TGGAAATGATCTTGAGCCCACAAGTCATTGGGTTGAAGTGATCTTTAGTATATGTGTCGTGTTAAGTGGA
TTGATGCTATTCACGTTATTAATTGGTAATATTCAGGTGTTCTTGCATGCGGTTATGGCAAAAAAGAGGA

AAATGCAACTAAGATGTCGCGATATGGAGTGGTGGATGAAGAGGAGACAACTACCGTCACTTCTTAGACA
AAGAGTTCGTCATTACGAACGCCAAAATTGGGCGTTGATGGGAGGTGAGGATGAGATGGAGTTGATTAAA
GACTTTCCCGAAGGCCTTAGACGCGATATCAAGCGTTTTCTATGCCTAGAGCTCATTCGAATGGTACCAT
TCTTTCATAACTTAGATGATTTGATACTTGACAACATATGTGATCGAGTTAAACCGCTAGTATTCTCAAA
GGACGAAAAGATCATACGCGAAGGTGATCCGGTTCAAAGAATGGTGTTTATAGTTCAAGGACGCGTAAAA
AGCGGCCAAAACCTAAGTAAAGGAGTTGTTGCAACGAGTATTTTGGACCCGGGAGGCTACCTAGGAGACG
AGCTTCTATCATGGTGCCTCCGAAGGCCATTTATAAACCGGCTTCCAGCATCTTCGGCTACTTTTACATG
TATGGAACCCACAGAAGCTTTTGGGCTAGATGCTAACAATCTTCGTTACGTGACCGATCATTTTCGGTAC
AAATTTGCCAACGAAAGGTTGAAACGAACCGTGAGATATCATTCAGCCAATTGGCGGACATGGGCTGCGG
TGAATATACAAATAGGGTGGAGGCGGTACATGGCGAGGATGCGACGGTCAGTGAATCAGGTGACAGATGA
GAATGGTGGCAGCAACCGTATGCTCCGGCAATATGCTGCCATTTTCATGTCAATAAGGCCTCATGATCAT
CTTGAATAG
>FC066345.1_*Vitis_vinifera*
ATACCACTATGTCTATATCCCCCCACCCCATAAACCAATACCCTTTACCCCCAACTCTTCATCCCCTTCT
CTCCTAATCTTTTTCACTTCCGCCCTCTTCTCTATCTCTCTCTCCCATGGTTTCTCTTTTCCGACAATGG
ACGCTCCACCCAACTTCCAAATCTCCCTTCCAAGGTGGATTGGAATATTCCGGCGCCGGAAAGCAAACTC
CGATGAAAACTACGAAGAAGACGACGGCCCCATCTCCAATTCCATTGAATGCTACGCCTGTACTCAGGTG
GGAGTGCCGGTGTTCCACTCCACCAGTTGTGACCAGGCCCACCAGCCGGAGTGGGAGGCCTCAGCCGGTT
CTTCCTTGATTCCAATTCAGGACAGGAGGACCGGGAAGGCCCGGTCCAACGGCCGCCCCTCGGGGCCTTT
CGGGCCGGTCCTGGACCCGAGGACCAAGCCCGTGCAGAGGTGGAACCGCGCGTTCCTACTCGTCCGTGGG
ATGTCCCTGGCAGTGGATCCCCTCTTCTTTTACGTCATATCCATTGGCGGCGAGGGGGGACCGTGCCTGT
ACATGGACGGTGGGCTGGCGGCCATCGTGACGGTGCTGCGGACGTGTGTGGACGCTGTGCACTTGTTTCA
CTTGTGGCTGCAGTTCAGGCTGGCGTACGTGTCTAGGGAGTCTCTGGTGGTGGGTTGTGGTAAACTCGTG
TGGGACGCACGTGCCA
>NM_203054.2_*Arabidopsis_thaliana*
ATGCAATTCCTTATAATCTCGCTTGAGTGGATTGGACTGTTTTCCGATAAGTTCCGTCGACAAACGACTG
GGATCGATGAAAACAGTAACCTCCAAATCAACGGTGGAGATTCGAGCAGCAGCGGCAGCGATGAGACGCC
GGTGCTAAGCTCCGTCGAGTGTTACGCTTGCACACAAGTAGGCGTCCCAGCTTTCCATTCAACTAGCTGC
GATCAAGCTCACGCGCCGGAGTGGCGTGCCTCCGCCGGCTCTTCTCTAGTTCCGATCCAGGAAGGATCTG
TCCCTAACCCAGCCCGAACCAGATTCCGACGTCTCAAAGGTCCGTTTGGTGAAGTTCTCGATCCTAGGAG
CAAGCGCGTGCAGAGATGGAACCGCGCGTTGCTTTTAGCTCGTGGGATGGCTTTAGCGGTGGATCCGCTC
TTCTTCTACGCGCTTTCCATCGGCCGAACTACCGGACCGGCGTGTCTTTACATGGATGGTGCGTTCGCCG
CGGTGGTCACGGTGCTCCGCACGTGTCTCGATGCTGTTCATCTTTGGCACGTGTGGCTTCAATTCAGACT
GGCCTACGTCTCGAGAGAGTCGCTTGTCGTTGGTTGTGGGAAGCTCGTTTGGGATCCACGCGCCATCGCG
TCTCACTACGCACGCTCTCTCACTGGCTTCTGGTTTGATGTTATCGTCATCCTCCCTGTCCCTCAGGCAG
TGTTTTGGTTAGTTGTGCCGAAACTGATAAGAGAAGAGAAGGTTAAGCTGATAATGACGATTCTGCTGCT
AATATTCTTGTTCCAGTTCCTCCCCAAGATTTATCACTGCATCTGTTTGATGAGAAGGATGCAGAAGGTC
ACTGGTTACATTTTTGGAACTATTTGGTGGGGTTTTGCTCTTAATCTCATCGCATATTTCATCGCTTCTC
ATGTTGCTGGGGGATGTTGGTATGTTCTCGCAATACAGCGTGTTGCTTCTTGCATAAGACAACAATGTAT
GAGAACCGGGAACTGCAATCTGAGTCTGGCTTGCAAAGAAGAGGTCTGTTACCAATTTGTGTCACCGACA
AGCACAGTTGGATATCCATGCTTATCTGGAAACCTTACCAGTGTGGTCAATAAGCCTATGTGCTTAGACT
CTAACGGACCATTCCGATATGGTATCTACCGTTGGGCACTTCCAGTCATCTCCAGCAACTCTCTTGCGGT
TAAGATCCTTTACCCCATCTTCTGGGGCCTAATGACTCTCAGCACATTTGCGAATGATCTTGAGCCCACA
AGCAACTGGCTCGAGGTTATTTTCAGTATAGTTATGGTTCTAAGTGGCTTGTTACTTTTCACGCTGTTGA
TAGGAAACATTCAGGTGTTTTTGCATGCGGTAATGGCGAAAAAAAGGAAAATGCAGATACGGTGTAGGGA
TATGGAATGGTGGATGAAACGTAGGCAGTTACCTTCCCGGTTAAGACAGAGGGTTAGGCGATTTGAGCGG
CAGAGATGGAATGCCTTGGGTGGTGAAGACGAGCTAGAACTTATACATGATTTGCCTCCGGGTCTTCGAA
GAGATATCAAACGATATCTTTGCTTTGATCTCATTAACAAGGTGCCATTGTTCAGGGGCATGGACGACTT
GATCCTCGACAACATTTGCGATCGGGCTAAGCCTCGAGTCTTCTCTAAAGACGAAAAGATCATCCGTGAA
GGAGATCCTGTACAGAGAATGATATTCATCATGCGTGGACGAGTCAAACGTATACAGAGCCTAAGCAAAG
GCGTCCTAGCCACTAGTACACTAGAACCAGGCGGTTACTTGGGCGACGAGCTACTCTCATGGTGCCTACG
TCGCCCGTTTCTGGACCGTCTTCCCCCTTCCTCAGCAACATTTGTCTGCCTAGAAAACATCGAGGCATTC
TCCCTCGGATCCGAAGATCTTAGGTACATTACCGATCATTTCCGTTATAAATTCGCGAACGAGCGGCTTA
AGCGGACCGCAAGATACTATTCCTCAAACTGGAGGACGTGGGCAGCGGTAAATATTCAGATGGCGTGGCG

CCGGCGTAGGAAAAGAACCCGTGGTGAAAACATCGGCGGTTCGATGAGTCCTGTGTCGGAGAATAGCATT
GAAGGTAACAGTGAACGCCGGTTACTTCAGTATGCAGCTATGTTCATGTCCATTCGACCGCATGATCATC
TCGAATAA
>DY651171.1_*Prunus_persica*
TTTGCTACCAATTTTTGTCACCGGCGGACACGATGCAAAATCCTTGTGGAGGCAACACAACAGCAAGTGT
GGTGAGAAAGCCCCCATTGTGCTTGGATGTCAATGGAACATTCAATTATGGAATCTATCAGTGGGCTCTC
CCGGTCATATCTAGCAACTCACTGGCTATTAAGATCCTATATCCCATTTTTTGGGGTCTAATGACTCTCA
GCACCTTTGGCAATGATCTTGAACCTACAAGTCACTGGCTAGAAGTTATTTTCAGCATATGCATTGTGCT
CAGTGGCTTACTTCTCTTCACTTTGTTGATTGGGAACATTCAGGTATTTCTGCACGCGGTTATGGCGAAG
AAGAGAAAAATGCAGCTGAGATGCAGAGACATGGAATGGTGGATGAGGCGAAGGCAGTTGCCATCCGGTT
TGAGACGAAGAGTGCGCCATTATGAAAGGCATAGATGGGTTACAATGGGGGGAGAAGATGAGATGGACTT
GATCAAAGACTTGCCTGAAGGGCTCCGAAGGGAAATTAAGCGCCACTTATGCCTAGACCTCATAAAAAAG
TTCCCCTGTTCCACAATTNGGATGATCTATTCTCGACACATCTG
>NM_121545.5_*Arabidopsis_thaliana*
ATGCCCTCTCACCCCAACTTCATCTTCAGGTGGATTGGACTGTTTTCCGATAAGTTCCGTCGACAAACGA
CTGGGATCGATGAAAACAGTAACCTCCAAATCAACGGTGGAGATTCGAGCAGCAGCGGCAGCGATGAGAC
GCCGGTGCTAAGCTCCGTCGAGTGTTACGCTTGCACACAAGTAGGCGTCCCAGCTTTCCATTCAACTAGC
TGCGATCAAGCTCACGCGCCGGAGTGGCGTGCCTCCGCCGGCTCTTCTCTAGTTCCGATCCAGGAAGGAT
CTGTCCCTAACCCAGCCCGAACCAGATTCCGACGTCTCAAAGGTCCGTTTGGTGAAGTTCTCGATCCTAG
GAGCAAGCGCGTGCAGAGATGGAACCGCGCGTTGCTTTTAGCTCGTGGGATGGCTTTAGCGGTGGATCCG
CTCTTCTTCTACGCGCTTTCCATCGGCCGAACTACCGGACCGGCGTGTCTTTACATGGATGGTGCGTTCG
CCGCGGTGGTCACGGTGCTCCGCACGTGTCTCGATGCTGTTCATCTTTGGCACGTGTGGCTTCAATTCAG
ACTGGCCTACGTCTCGAGAGAGTCGCTTGTCGTTGGTTGTGGGAAGCTCGTTTGGGATCCACGCGCCATC
GCGTCTCACTACGCACGCTCTCTCACTGGCTTCTGGTTTGATGTTATCGTCATCCTCCCTGTCCCTCAGG
CAGTGTTTTGGTTAGTTGTGCCGAAACTGATAAGAGAAGAGAAGGTTAAGCTGATAATGACGATTCTGCT
GCTAATATTCTTGTTCCAGTTCCTCCCCAAGATTTATCACTGCATCTGTTTGATGAGAAGGATGCAGAAG
GTCACTGGTTACATTTTTGGAACTATTTGGTGGGGTTTTGCTCTTAATCTCATCGCATATTTCATCGCTT
CTCATGTTGCTGGGGGATGTTGGTATGTTCTCGCAATACAGCGTGTTGCTTCTTGCATAAGACAACAATG
TATGAGAACCGGGAACTGCAATCTGAGTCTGGCTTGCAAAGAAGAGGTCTGTTACCAATTTGTGTCACCG
ACAAGCACAGTTGGATATCCATGCTTATCTGGAAACCTTACCAGTGTGGTCAATAAGCCTATGTGCTTAG
ACTCTAACGGACCATTCCGATATGGTATCTACCGTTGGGCACTTCCAGTCATCTCCAGCAACTCTCTTGC
GGTTAAGATCCTTTACCCCATCTTCTGGGGCCTAATGACTCTCAGCACATTTGCGAATGATCTTGAGCCC
ACAAGCAACTGGCTCGAGGTTATTTTCAGTATAGTTATGGTTCTAAGTGGCTTGTTACTTTTCACGCTGT
TGATAGGAAACATTCAGGTGTTTTTGCATGCGGTAATGGCGAAAAAAAGGAAAATGCAGATACGGTGTAG
GGATATGGAATGGTGGATGAAACGTAGGCAGTTACCTTCCCGGTTAAGACAGAGGGTTAGGCGATTTGAG
CGGCAGAGATGGAATGCCTTGGGTGGTGAAGACGAGCTAGAACTTATACATGATTTGCCTCCGGGTCTTC
GAAGAGATATCAAACGATATCTTTGCTTTGATCTCATTAACAAGGTGCCATTGTTCAGGGGCATGGACGA
CTTGATCCTCGACAACATTTGCGATCGGGCTAAGCCTCGAGTCTTCTCTAAAGACGAAAAGATCATCCGT
GAAGGAGATCCTGTACAGAGAATGATATTCATCATGCGTGGACGAGTCAAACGTATACAGAGCCTAAGCA
AAGGCGTCCTAGCCACTAGTACACTAGAACCAGGCGGTTACTTGGGCGACGAGCTACTCTCATGGTGCCT
ACGTCGCCCGTTTCTGGACCGTCTTCCCCCTTCCTCAGCAACATTTGTCTGCCTAGAAAACATCGAGGCA
TTCTCCCTCGGATCCGAAGATCTTAGGTACATTACCGATCATTTCCGTTATAAATTCGCGAACGAGCGGC
TTAAGCGGACCGCAAGATACTATTCCTCAAACTGGAGGACGTGGGCAGCGGTAAATATTCAGATGGCGTG
GCGCCGGCGTAGGAAAAGAACCCGTGGTGAAAACATCGGCGGTTCGATGAGTCCTGTGTCGGAGAATAGC
ATTGAAGGTAACAGTGAACGCCGGTTACTTCAGTATGCAGCTATGTTCATGTCCATTCGACCGCATGATC
ATCTCGAATAA
>FP026443.1_*Quercus_robur*
GAGGAAAGATGCAATTTTCTATCTCTCATATTTTACATCCAATGCGATTTCCATGGCTTCATCGGTGACT
CTAGGGGTTGACACGAACAAGATTTTATTGACTAATGGGGTAGAAAAGAAAGATGAAGAAAAGATTTCAG
ACATGATTTTATTCTAGATGGTCATGTGGCCTTATCGACATGAACATTGCAGCATACTGCAGAAGCCTTC
TCTCACTGCTTCCATTTTCAGTGGCACAAGGAATCATTGGACCCCTGGTCCTCATCCTGTACCTGCGCCA
ACCTAATTGTATAATCACAGCAGCCCATGTTCGCCAATTAGATGAGTAATATCTCGCAGTTCGCTTGAGT
CTCTCATTGCCAAATTTGTAGCGGAAATGATCAGTGATGTATCTAAGATGGGCAGAATCAAGGCTAAATG
CTTCTGTTGATTCAATGCAACTAAATGTTGCTGATGAAGCTGGAAGGCGATCCATAAATGGGCGGCGAAG

GCACCAAGAGAGAAGTTCATCACCTAA
>XM_006338336.2_*Solanum_tuberosum*
ATGTCTTCTCACCAAGACGTCCGCTTCTTCCTCTCAAGGTGGTTCGGCATATTCCGACGAAGATCAGTTC
AACCTGATAACAGCGATGACAACGATGACGACAACAATCCAATCTCAAATTCCATTGAATGTTATGCATG
TACTCAAGTTGGCGTCCCTGTTTTCCACTCCACCAGTTGCGACGGAGCTAACCAACCGGAGT GGGAAGCT
TCAGCCGGTTCTTCGCTAATTCCAATTCAAAACCGGACTGATTTGAAAACCGGAAAATCCCGGTCCAGTC
GCAGCCGGCACACATCGGGGCCGTTCGGGCGTGTATTAGACCCTCGAAGCAAGCGCGTGCAGAGATGGAA
CCGAATGATATTATTGGCACGTGGCATGGCTTTAGCCGTTGATCCTCTATTCTTTTACGCCTTATCCATC
GGCCGTGGTGGATCGCCGTGTTTGTACATGGACGGCGGCCTGGCGGCTATCGTCACGGTGATTCGGACTA
GCGTCGACGCCGTGCACCTCTTCCATTTGTGGTTGCAGTTTCGGTTGGCTTACGTGTCGAGAGAATCGCT
GGTGGTTGGGTGTGGGAAACTCGTGTGGGATGCGCGTGCGATTGCTTCTCACTATGTTAGGTCCCTTAAA
GGATTCTGGTTCGATGCTTTTGTCATCCTTCCCGTTCCACAGGCTGTATTCTGGCTGGTGGTTCCAAAAC
TAATAAGAGAAGAGCAGATAAAGCTTATAATGACGATCCTTTTATTAATGTTCTTGTTCCAGTTCCTTCC
CAAAGTTTATCACTGTATAAGCTTAATGAGAAGGATGCAAAAGGTTACTGGATACATTTTTGGCACCATT
TGGTGGGGATTTGGACTTAATCTCATTGCTTATTTTATTGCTTCTCATGTTGCTGGGGGATGCTGGTATG
TTCTTGCAATACAAAGAGTGGCTTCATGTCTAAGGCAGCAGTGTGAGCGCAACCCTTCGTGTAATCTATC
TTTGTCATGCTCAGAGGAGGTGTGTTATCAGTTTCTATTGCCAACAGGAACTGTGGGAAATCCATGTGCT
GGGAACTCAACAACAGTGACTAGGAAGCCAATGTGTTTGGATGTCAATGGACCATTTCCATATGGGATAT
ACCAATGGGCACTTCCTGTTGTTTCTAGCAGATCCGTCACTGTTAAGATTCTTTACCCCATCTTTTGGGG
ACTGATGACCCTTAGCACATTTGGCAATGACTTAGAACCAACAAGTCACTGGCTAGAAGTTATTTTCAGT
ATATGCCTTGTGCTTAGTGGATTGATGCTCTTTACTCTGTTGATTGGTAACATCCAGGTGTTTTTACACG
CGGTCATGGCAAAGAAGCGAAAAATGCAATTAAGATGTAGGGATATGGAATGGTGGATGAGGAGAAGACA
ATTGCCATCACAATTAAGACAAAGAGTTCGCCACTTTGAACACCAGAGATGGGCTATGATGGGT GGCGAA
GATGAGATGGAACTTGTAAAAGACTTGCCAGAGGGACTTCGAAGGGACATCAAACGCTTTCTTTGCCTTG
ATCTTATTAAAAAGGTTCCTCTGTTCCAAAGTTTGGATGATCTGATTCTAGATAACATTTGTGATCGTGT
TAAGCCACTTGTGTTCTCTAAAGATGAGAAGATCATAAGAGAAGGAGATCCAGTGCACAGAGTTGTGTTC
ATTGTTCGTGGACGTGTAAAAAGTAGCCAAAACCTCAGTAAAGGAGTGATTGCCACAAGCATACTTGAGC
CTGGAGGCTTCTTTGGAGATGAACTTCTTTCCTGGTGCTTACGCCGTCCCTTTATTGACAGACTTCCAGC
TTCTTCCGCAACCTTCACTTGCATTGAATCTACAGAAGCATTTGGCTTAGATGCAAACCACCTCCGATTT
ATCACGGATCACTTCAGATACAAATTTGCAAACGAGAGGCTAAAGAGAACAGCAAGGTATTATTCATCCA
ATTGGAGAACCTGGGCTGCTGTCAACATACAGTTAGCTTGGCGACGTTACATGATGAGGACTAGCCGTCC
CACTATACATGTTATCGAAAATGGGGATAATGATCAGCGTCTTCGGAAGTATGCTGCAATGTTCTTGTCA
ATCAGGCCACATGATCATCTTGAATAG
>BY805038.1_*Eutrema_halophilum*
AAAACTCAAATGCTAGTTTTTTTTAATAAGGTACATAAACATTCACATAAAGATCTTTTCAAAATAATAA
TAATAATAACAATTGAGGACGAACAATGGAAACAAACAATTATTGAGACCAATGATTTTGGCACTTTCTG
TTTGTTTTACTCGAGATGATCATGTGGTCGAATGGACATGAACATGGCGGCATACTGAAGTAAACGGCGT
TCACTGTTACCTTCGACGCTGTTCTCCGACACAGGACTCATCGAACCGCCGTTGTTTTCACCACCACGGG
TTCTCTTCCTGTACCGCCGCCACGCCATTTGAATATTTACGGCAGCCCAAGTCCTCCAGTTTGAGGAATA
GTATCTTGCGGTCCGCTTAAGCCGCTCGTTTGCGAATTTATAACGGAAATGATCGGTGATGTACCTAAGA
TCTTCGGATCCGAGTGAGAACGCCTCTATGTTGTCTAGGCAGACAAATGTTGCAGAGGAAGGAGGAAGAC
GGTCGATGAATGGACGCCTTAAGCACCATGAGAGTAGCTCGTCGCCTAAGTAACCGCCTTGTTCGAGCGT
ACTAGTGGCTACCACACCTTTGCTTAGGCTCTGGTTACGTT
>XM_015306820.1_*Solanum_tuberosum*
ATGGCTCATTTTCTCTCAAACTCCAGGTGGTTCGGCATATTCCGACGAAGATCAGTTCAACCTGATAACA
GCGATGACAACGATGACGACAACAATCCAATCTCAAATTCCATTGAATGTTATGCATGTACTCAAGTTGG
CGTCCCTGTTTTCCACTCCACCAGTTGCGACGGAGCTAACCAACCGGAGTGGGAAGCTTCAGCCGGTTCT
TCGCTAATTCCAATTCAAAACCGGACTGATTTGAAAACCGGAAAATCCCGGTCCAGTCGCAGCCGGCACA
CATCGGGGCCGTTCGGGCGTGTATTAGACCCTCGAAGCAAGCGCGTGCAGAGATGGAACCGAATGATATT
ATTGGCACGTGGCATGGCTTTAGCCGTTGATCCTCTATTCTTTTACGCCTTATCCATCGGCCGTGGTGGA
TCGCCGTGTTTGTACATGGACGGCGGCCTGGCGGCTATCGTCACGGTGATTCGGACTAGCGTCGACGCCG
TGCACCTCTTCCATTTGTGGTTGCAGTTTCGGTTGGCTTACGTGTCGAGAGAATCGCTGGTGGTTGGGTG
TGGGAAACTCGTGTGGGATGCGCGTGCGATTGCTTCTCACTATGTTAGGTCCCTTAAAGGATTCTGGTTC
GATGCTTTTGTCATCCTTCCCGTTCCACAGGCTGTATTCTGGCTGGTGGTTCCAAAACTAATAAGAGAAG

AGCAGATAAAGCTTATAATGACGATCCTTTTATTAATGTTCTTGTTCCAGTTCCTTCCCAAAGTTTATCA
CTGTATAAGCTTAATGAGAAGGATGCAAAAGGTTACTGGATACATTTTTGGCACCATTTGGTGGGGATTT
GGACTTAATCTCATTGCTTATTTTATTGCTTCTCATGTTGCTGGGGGATGCTGGTATGTTCTTGCAATAC
AAAGAGTGGCTTCATGTCTAAGGCAGCAGTGTGAGCGCAACCCTTCGTGTAATCTATCTTTGTCATGCTC
AGAGGAGGTGTGTTATCAGTTTCTATTGCCAACAGGAACTGTGGGAAATCCATGTGCTGGGAACTCAACA
ACAGTGACTAGGAAGCCAATGTGTTTGGATGTCAATGGACCATTTCCATATGGGATATACCAATGGGCAC
TTCCTGTTGTTTCTAGCAGATCCGTCACTGTTAAGATTCTTTACCCCATCTTTTGGGGACTGATGACCCT
TAGCACATTTGGCAATGACTTAGAACCAACAAGTCACTGGCTAGAAGTTATTTTCAGTATATGCCTTGTG
CTTAGTGGATTGATGCTCTTTACTCTGTTGATTGGTAACATCCAGGTGTTTTTACACGCGGTCATGGCAA
AGAAGCGAAAAATGCAATTAAGATGTAGGGATATGGAATGGTGGATGAGGAGAAGACAATTGCCATCACA
ATTAAGACAAAGAGTTCGCCACTTTGAACACCAGAGATGGGCTATGATGGGTGGCGAAGATGAGATGGAA
CTTGTAAAAGACTTGCCAGAGGGACTTCGAAGGGACATCAAACGCTTTCTTTGCCTTGATCTTATTAAAA
AGGTTCCTCTGTTCCAAAGTTTGGATGATCTGATTCTAGATAACATTTGTGATCGTGTTAAGCCACTTGT
GTTCTCTAAAGATGAGAAGATCATAAGAGAAGGAGATCCAGTGCACAGAGTTGTGTTCATTGTTCGTGGA
CGTGTAAAAAGTAGCCAAAACCTCAGTAAAGGAGTGATTGCCACAAGCATACTTGAGCCTGGAGGCTTCT
TTGGAGATGAACTTCTTTCCTGGTGCTTACGCCGTCCCTTTATTGACAGACTTCCAGCTTCTTCCGCAAC
CTTCACTTGCATTGAATCTACAGAAGCATTTGGCTTAGATGCAAACCACCTCCGATTTATCACGGATCAC
TTCAGATACAAATTTGCAAACGAGAGGCTAAAGAGAACAGCAAGGTATTATTCATCCAATTGGAGAACCT
GGGCTGCTGTCAACATACAGTTAGCTTGGCGACGTTACATGATGAGGACTAGCCGTCCCACTATACATGT
TATCGAAAATGGGGATAATGATCAGCGTCTTCGGAAGTATGCTGCAATGTTCTTGTCAATCAGGCCACAT
GATCATCTTGAATAG
>GO555384.1_*Malus_domestica*
GCCACGATTTTTTTTTTTTTTTTTTGAGATATATCGTTGGTATTTTTTTATTCATAACTCAACGTACAGA
TATGATTGTCCACACCAATAACCTTAACGGAGAATTGATACTTAAATGAGATCAATGTATGGTTTAGACA
ATGCCCTTTTAAGTATAAAGCTAACATTACAATAGATTAAAATATCGTCTTTAATACAGTGGGTTAGGCC
AGTTCGCCATAATAATATGTCTACCCACTTGTACTTAAGTTCGAAAATTCTCTTATAAATTAAAATAGTT
TGAAATATAGTATTCTCATGAAAATAATAATATTGATAATGATACCATCATAGAACAATATTTCTACATA
CACTAATGCTACATTGAATTCAGACCTTTATTTTAATTGATGTGTTAATTAGGTAAAATGGATTAGAACA
TGATCTGAAAATGTGGCCTAAATAGTTTTATTTTCTACATGAAGAAGATTTTATTAAGCACTAATTGAGT
GCTATTTATTCAAGGTGATCGTGTGGCCTAAGTGACATGAACATTGCAGCATACTGCAACAGCTTCCGCT
CAGAGCCTCCATTTGGAATCACAGGAATCACCGGACCTCGATTAGTCCTCAACCTGTAGCGACGCCAAGT
AAATTGTATGTTCACAGCTG
>XM_004232136.4_*Solanum_lycopersicum*
ATGTCTTCTCACCAAGACGTCCGCTTCTTCCTCTCAAAGTGGTTTGGCATATTCCGACGAAGATCAGTTC
AACCTGATAACAGCGACGACAACGATGACGACATCAATCCAATCTCAAATTCCATTGAATGTTATGCATG
TACTCAAGTTGGCGTCCCTGTTTTCCACTCCACCAGTTGCGATGGAGCTAACCAACCGGAGTGGGAAGCT
TCAGCCGGTTCTTCTCTAGTTCCAATTCAAAACCGGACGGATTCAAAAACCGGAAAATCCCGGTCCAGTC
GCAGCCGGCACACATCGGGGCCGTTCGGGCGTGTATTAGACCCTCGAAGCAAGCGCGTGCAGAGATGGAA
CCGAATGATTTTATTGGCACGTGGCATGGCTTTAGCCGTTGATCCTCTATTCTTTTACGCCTTATCCATC
GGCCGCGGTGGATCGCCGTGTTTGTACATGGACGGCAGCCTGGCGGCTATCGTCACCGTGATTCGGACTA
GCGTCGACGCCGTGCACCTCTTCCATTTGTGGTTGCAGTTTCGTTTGGCTTACGTGTCGAGAGAATCGCT
GGTGGTTGGTTGTGGGAAACTCGTGTGGGATGCGCGTGCGATTGCTTCTCACTATGTTAGGTCCCTTAAA
GGATTTTGGTTCGATGCTTTTGTCATCCTTCCCGTTCCACAGGCTGTATTCTGGCTGGTGGTTCCAAAAC
TAATAAGAGAAGAGCAGATAAAGCTTATAATGACGATCCTTTTATTAATGTTCTTGTTCCAGTTCCTTCC
CAAAGTTTATCACTGTATAAGCTTAATGAGAAGGATGCAAAAGGTTACAGGATATATTTTTGGTACCATC
TGGTGGGGATTTGGACTTAATCTCATTGCTTATTTTATTGCTTCTCATGTTGCTGGGGGATGCTGGTATG
TTCTTGCAATACAAAGAGTGGCTTCATGTCTAAGGCAGCAGTGTGAGCGCAACCCTTCGTGTAATCTATC
TTTGTCTTGCTCAGAGGAGGTGTGTTATCAGTTTCTGTTGCCAACAGGAACTGTGGGAAATCCATGTGCT
GGGAACTCAACAACAGTGACCAGGAAGCCAATGTGTTTGGATGTCAATGGACCATTTCCATATGGGATAT
ACCAATGGGCACTTCCTGTTGTTTCTAGCAGATCCGTCACTGTTAAGATTCTTTACCCCATCTTTTGGGG
ATTGATGACCCTTAGCACATTTGGCAATGACTTAGAACCAACAAGTCACTGGCTGGAAGTTATTTTCAGT
ATATGCCTTGTGCTTAGTGGATTGATGCTCTTCACTTTGCTGATTGGTAACATTCAGGTGTTTTTACACG
CGGTCATGGCAAAGAAGCGAAAAATGCAATTAAGATGTAGGGATATGGAATGGTGGATGAGGAGGAGACA
ATTACCATCACAATTAAGACAAAGAGTTCGCCACTTTGAACACCAGAGATGGGCTATGATGGGTGGCGAA

GATGAGATGGAACTTGTAAAAGACCTGCCAGAAGGACTACGAAGGGACATCAAACGCTTTCTTTGCCTTG
ATCTTATTAAGAAGGTTCCTCTGTTCGAAAGTTTGGATGATCTGATTCTAGATAACATTTGTGATCGCGT
TAAGCCACTTGTGTTCTCTAAAGATGAGAAGATCATAAGAGAAGGAGATCCAGTGCACAGGGTTGTGTTC
ATTGTTCGTGGACGTGTAAAAAGTAGCCAAAACCTCAGTAAAGGAGTGATTGCCACAAGCATACTTGAGC
CTGGAGGCTTCTTTGGAGATGAACTTCTTTCCTGGTGCTTACGCCGTCCCTTTATTGACAGACTTCCAGC
TTCTTCCGCAACCTTCACTTGCATTGAATCTACAGAAGCATTTGGCTTAGATGCAAACCACCTTCGATTT
ATCACGGATCACTTCAGATACAAATTTGCAAACGAGAGGCTGAAGAGAACAGCAAGGTATTATTCATCCA
ATTGGAGAACCTGGGCTGCTGTGAATATACAGTTAGCTTGGCGACGTTACATGATGAGGACTAGCCGTCC
CACTATACATGTGATCGAAAATGGGGATAATGATCATCGTCTTCGCAAGTATGCTGCAATGTTCTTGTCA
ATCAGACCACATGATCATCTTGAATAG
>MN605521.1_*Ricinus_communis*
ATGCCTTCTCAGCCCGACTTCCACTTCTCTCTCCAAAGGTGGATTGGACTGCTTTGCGGCAAGAATGGGC
AGAATGATAACAGTGATAGCAGTGGCGGTGACAGCACTGTTAGAAACATCGACGACAATAACAACATCTC
CAGCTCCGTAGAATGTTATGCCTGCACCCAAGTAGGTGTTCCTGTCTTTCACTCAACAAGCTGTGACCAC
GCTCACCAACCCCAATGGGTCGCTTCAGCCGGTTCTTCTCTTATCCCCATTCAAAACCAGCCAGGAGCGA
AGAAACCGCCCACAATTATCTCGCGTTTCCCAGCGGGACCATTCGGAGCCGTCCTGGACCCAAGAAGTAA
GCGCGTACAGAAATGGAACCGGGCCTTCCTTCTTGCACGTGGCATGGCTTTAGCCATCGATCCGTTATTC
TTCTACTCGTTATCAATCGGCAGGGGAGGAGCTCCTTGCCTTTACATGGACGGTGGATTAGCCGCAATCG
TGACGGTCCTTCGCACGTGTGTGGACGCCATACATTTGTGCCACTTATGGTTGCAATTCAGATTAGCGTA
TGTGTCCAGGGAGTCGTTAGTTGTCGGATGTGGGAAACTCGTGTGGGACGCACGTGCCATAGCGTCACAC
TATGTTCGGTCTTTGAAAGGCTTCTGGTTTGATGCCTTTGTTATACTGCCAGTTCCTCAGGCTGTGTTCT
GGTTGATTCTTCCCAAATTGATCAGAGAAGAGCAGATTAAACTGATAATGACGATACTTCTACTGATCTT
CTTGTTCCAGTTCCTCCCTAAAGTATACCACTGCATATACTTGATGAGAAAAATGCAGAAGGTTACAGGT
TATATCTTTGGGACGATTTGGTGGGGTTTTGGCCTTAATCTCATTGCTTACTTCATTGCTTCTCATGTTG
CTGGAGGATGTTGGTATGTTCTTGCTATACAACGCGCTGCGTCGTGCCTCAGGCAGCAGTGTCAGAGAAG
ACCGAACTGTGACCTGTTTCTATCTTGTTCCGAGGAGGTGTGTTATCAGTTACTAGTACCTACAGGCACA
ATCGGGAACTCCTGCGGTGGAAACTCAACTGCAGCTGTCAGGAAGCCCATGTGCTTAGATATTGCAGGAC
CATTCAAATATGGCATTTATAAGACTGCTCTTCCTGTTATCGCTAGCAATTCGTTGGCTGTCAAGATCCT
TTATCCCATCTTCTGGGGTTTAATGACCCTCAGCACCTTTGGCAATGATCTTGAGCCGACGAGTAATTGG
CTAGAAGTGATATTCAGTATATGTATAGTGCTTAGTGGTTTAATGCTCTTCACATTGTTGATTGGTAATA
TCCAGGTGTTCCTACACGCGGTCATGGCAAAGAAGCGGAAAATGCAGCTAAGATGTCGAGACATGGAATG
GTGGATGAGGAGGAGACAGTTACCTTCTCGTTTGAGGCAAAGAGTTCGCCATTTCGAACGCCAGAAATGG
ACTGCCATGGGAGGGGAAGATGAAATGGAGTTAATCAAGGACTTACCTGAAGGACTCCGGAGAGATATCA
AGCGCTACCTATGCCTAGACCTTATCAAGGAGGTACCTCTGTTTCATAACTTGGATGATCTTATCCTTGA
CAACATCTGTGACCGAGTTAAGCCTCTTGTCTTCTCTAAAGATGAAAAGATAATAAGAGAAGGAGACCCT
GTGCAAAGGATAGTATTCATTGTTCGTGGGCGAATAAAACGAAGCCAAAGCCTTAGCAAAGGCATGGTAG
CAACAAGTGTGCTAGAACCAGGAGGCTATTTAGGCGATGAGCTGCTATCCTGGTGTCTTCGACGCCCATT
CATAGACCGGCGTCCTGCCTCATCTGCTACATTCGTATGTTTAGAATCTACAGAAGCCTTCGGTCTTGAT
GCCAACCATCTTCGTTACATAACTGATCACTTCCGATACAAATTTGCCAATGAAAGGCTTAAGCGAACTG
CAAGATATTACTCATCAAACTGGAGAACATGGGCAGCAGTAAACATACAATTCGCTTGGCGTCGATACAG
GATGAGGACTAGAGGTCCTGTGATTCCTGTAACAACAGAAAATGGAGGCACAGATCGCCGTCTGCTGCAA
TATGCTGCTATGTTCATGTCAATCAGGCCACATGACCACCTTGAATAA
>KX426978.1_*Prunus_persica*
ATGTCCTCCCACTTCTCCTTCTCCCTCTCCAGGTGGACCAGAGCTCTCCGGCGCCAGAGCTCTCGAGCCG
ACGACACCGACAACAGCCACAACAGCAACAACATCGAAGACATCATCAACGCCCCAATCTCCAACTCCGT
CGAGTGCTACGCCTGCACGCAGGTTGGGGTCCCAGTCTTCCACTCCACCAGCTGCGACGGGGCCCACCAG
CTCCAATGGGAGGCCTCCGCCGGCTCTTCCCTAGTCCCCATCCAATCCCGCTCCGACCTCAAAAAGGGCC
TCTCCCGCGGCCTCCCCGCCGGCCCCTTCGGCCATGTCCTCGACCCCCGCAGCAAGCGCGTGCAGAGGTG
GAACCGCGCCTTCCTCCTCGCGCGTGGGATGGCGCTGGCAGTCGACCCGCTCTTCTTCTACGCGCTTTCC
ATTGGCAGGGGAGGGACCCCCTGCCTCTACATGGACGGCGGGTTGGCGGCCATCGTCACGGTGCTGCGCA
CGTGCGTGGACGCCGTGCACCTCTGCCACCTGTGGCTTCAGTTCCGGCTGGCGTACGTGTCGCGGGAGTC
GCTCGTTGTCGGGTGCGGGAAGCTCGTGTGGGATCCACATGCCATCGCCTCCCACTACGTTCGGTCCCTC
AAAGGCTTCTGGTTCGACGCCTTCGTTATCCTCCCCGTTCCCCAGGCTGTATTTTGGCTGGTACTTCCAA
AATTGATCAGAGAAGAAAAGATTAAGGTGATAATGACCATTCTTCTCTTAATTTTTTTGTTCCAATTCCT

CCCAAAAGTGTACCACAGCATCTGCTTGATGAGAAGAATGCAGAAGGTCACAGGCTACATTTTTGGCACC
ATCTGGTGGGGTTTTGGCCTCAATCTCATTGCCTACTTCATTGCCTCTCACGTTGCCGGGGGGTGCTGGT
ATGTGCTCGCAATTCAGCGCGTAGCTTCATGCCTAAGGCAACATTGTGAATTGAGTGTCAACTGTAATCT
CTCTTTGTCTTGCTCAGAGGAAGTTTGCTACCAATTTTTGTCACCGGCGGACACGATGCAAAATCCTTGT
GGAGGCAACACAACAGCAAGTGTGGTGAGAAAGCCCCCATTGTGCTTGGATGTCAATGGAACATTCAATT
ATGGAATCTATCAGTGGGCTCTCCCGGTCATATCTAGCAACTCACTGGCTATTAAGATCCTATATCCCAT
TTTTTGGGGTCTAATGACTCTCAGCACCTTTGGCAATGATCTTGAACCTACAAGTCACTGGCTAGAAGTT
ATTTTCAGCATATGCATTGTGCTCAGTGGCTTACTTCTCTTCACTTTGTTGATTGGGAACATTCAGGTAT
TTCTGCACGCGGTTATGGCGAAGAAGAGAAAAATGCAGCTGAGATGCAGAGACATGGAATGGTGGATGAG
GCGAAGGCAGTTGCCATCCGGTTTGAGACGAAGAGTGCGCCATTATGAAAGGCATAGATGGGTTACAATG
GGGGGAGAAGATGAGATGGACTTGATCAAAGACTTGCCTGAAGGGCTCCGAAGGGAAATTAAGCGCCACT
TATGCCTAGACCTCATAAAAAAGGTTCCCCTGTTCCACAATTTGGATGATCTTATTCTCGACAACATCTG
CGATCGAGTTAGGCCACTGGTTTTCTCTAAAGATGAAAAGATAATTAGAGAAGGAGACCCTGTTCCGAAG
ATGCTATTTATCGTGCGTGGACACATAAAGCGTAGCCAAGGCCTAAGCAAAGGCATGGTGGGAACCAATG
TGCTTGAACCAGGAGGGTTTCTGGGGGATGAGCTGCTCTCCTGGTGCCTTCGCCGGCCATTCATAGACCG
CTTGCCAGCCTCATCTGCCACGTTTGTTTGCCTCGAATCAACAGAAGCATTTGGCCTCAATGCAGATGAT
CTGAGATACATCACAGATCATTTCAGATACAAATTTGCCAATGAGAGGCTCAAAAGAACAGCAAGATACT
ACTCTTCCAATTGGAGAACTTGGGCTGCTGTGAACATACAATTCGCTTGGCGCCGCTACAGGCTGAGGAC
TAACAGAGGTCCTGTGATCCCTGTGGTGCAAAATGGAGGCACTGAGCAGAGGCTGTTGCAGTATGCTGCA
ATGTTCATGTCACTTAAGCCACATGACCACCTTGAATAA
>XM_006373283.2_*Populus_trichocarpa*
ATGCCTTCCCAGCCCAACTCCCACTTCTCCCTTCCAAGGTGGATTGGATTACTGTGTCACAAGAACAGCC
AAAGCGAAACTGGTGACAATAGTGACAACAGCAACCTTAACAGTACCAACAACATTGACGACAGCAACCC
AATCTCTAACTCCATAGAATGCTACGCTTGCACACAAGTAGGAGTCCCTGTCTTCCACTCCACAAGCTGT
GACCAGGCCCACCAGCCTGAATGGGAGGCCTCCGCGGGCTCATCATTAGTCCCCATCAAAAACCGGCTCG
GTTCAAGAAAGAGCCCAGCTAACCGAGCCCAATCTCGCCGGCCGGCCGGTCCATTAGGAACTGTTCTCGA
TCCACGTAGCAAGCGCGTACAGAAATGGAACCGGGCTTTCCTGCTAGCACGTGGCATGGCTTTGGCGGTC
GATCCCTTGTTTTTCTACGCTTTGTCGATAGGGAGAAACGGGGCCCCTTGCTTGTACATGGACGGTGGGT
TGGCCGCAATCGTGACCGTCCTTCGCACGAGTGTGGATGCTATACACTTGTGCCATCTGTGGTTACAGTT
CAGGTTGGCTTATGTGTCAAGAGAGTCTCTTGTTGTGGGGTGCGGGAATCTGGTGTGGGACGCACGTGCC
ATTGCATCTCATTACGTGAGGTCTCTTAAGGGCTTTTGGTTTGATGCTTTCGTCATTCTTCCCGTTCCTC
AGGCTGTATTTTGGTTACTTGTACCGAAATTAATCAGAGAAGAGCAGATTAAGCTCATAATGACAATACT
TTTGTTGATCTTCTTGTTCCAATTCCTCCCTAAGGTGTACCACTGCATATGCTTGATGAAAAGAATGCAA
AAGGTCACAGGTTATATATTTGGCACCATTTGGTGGGGCTTCGGCCTTAATCTCATTGCCTACTTCATTG
CCTCTCATGTTGCAGGTGGATGTTGGTATGTCCTCGCAATACAACGCGTTGCTTCATGCCTCAAGCAGAG
TTGTGAGAGTAGGCCTAATTGTGATCTCTCTTTGGCTTGCTCGGAGGAGGTTTGCTATCAGTTCCTGTTA
CGAGCAGGCACAATTGGAAATCCATGCGTTGGTAACACAACACATACTGTTAGAAAGCCTATGTGCCTGG
ATGTTAATGGAGCATTCAATTATGGGATTTACAAGTGGGCTCTTCCAGTCATTTCTAGCAATTCTTTGTC
CGTGAAAATTCTTTATCCCATTTTCTGGGGTTTAATGACCCTCAGCACCTTTGGCAATGATCTTGAACCA
ACAAGTCACTGGCTAGAAGTAATCTTCAGTATATGTATTGTTCTCAGTGGTTTAATGCTCTTCACTCTAC
TGATTGGGAACATCCAGGTGTTCTTGCACGCGGTCATGGCAAAGAAGAGAAAAATGCAGCTGAGATGCAG
AGATATGGAATGGTGGATGAGGAGGAGACAGTTGCCTTCTCGTTTGAGACAGAGAGTTCGCCATTATGAA
CGGCAGAGATGGGCAACCATGGGAGGTGAAGATGAGATAGAACTAATCAAAGACTTACCTGAAGGACTCC
GGAGAGATATCAAGCGCTATCTTTGCCTAGATCTTATCAAGAAGGTACCTTTGTTCCACAACCTGGATGA
TCTTATTCTTGACAACATCTGTGATCGGGTTAAGCCCCTTGTTTTTCTAAAGATGAGAAGATAATCAGA
GAAGGAGACCCAGTGCAAAGGATGGTGTTCATTGTTCGTGGGCGTATAAGGAGTAGCCAGACTCTTAGCA
AAGGCATGGTGGCAACTAGTGTGCTTGAGCCAGGAGGCTTCCTGGGTGACGAGCTGCTATCCTGGTGTCT
TCGCCGTCCATTCATAGACCGACTTCCAGCCTCATCAGCGACATTTGCTTGTATTGAATCGACAGAAGCA
TTTGGTCTTGATGCAAACCATCTTCGGTACATCACAGATCACTTCCGCTACAAATTTGCCAATGAAAGAC
TTAAGAGAACTGCAAGATATTACTCATCAAACTGGAGAACATGGGCAGCAGTCAATATACAATTCGCTTG
GCGACGTTACAGAATGAGGACTAGGGGACCGGTGATTCCTGTAACAGAAAGTGGAGGTACTGACCGCCGG
CTCCTGCAGTATGCTGCAATGTTCATGTCAATCAGGCCACACGACCACCTTGAATAA
>DND1_Scaffold410_*Castanea_mollissima*

TTATTCTAGATGGTCATGTGGCCTTATCGACATGAACATTGCAGCATACTGCAGAAGCCTTCTCTCACTGCTT
CCATTTTCAGTGGCACAAGGAATCATTGGACCCCTGGTCCTCATCCTGTACCTGCGCCAAGTTAATTGTATAA
TCACAGCAGCCCATGTTCGCCAATTAGATGAATAGTATCTCGCAGTTCGCTTGAGTCTCTCATTGCCAAATTT
GTAGCGGAAATGATCAGTGATGTATCTAAGATGGGCAGAATCAAGGCCAAATGCTTCTGTTGATTCAATGCAA
CTAAATGTTGCTGATGAAGCTGGAAGGCGTTCCATAAATGGGCGGCGAAGGCACCAAGAGAGAAGTTCATCAC
CTAAAAATCCTCCAGGTTCAAGTATACTTGTGGCTACCATGCCTTTGCTTAGGCTTTGACAACGTTTTACACG
TCCACGAACAATGAACATTATCCTCGGTACAGGATCTCCTTCTCTGATTATCTTCTCATCTTTAGAGAAGACA
AGAGGCTTAACCCGATCACAGATGTTGTCAAGAATAAGATCATCCAAGTTGTGGAAGAGAGGCACCTTCTTTA
TAAGGTCTAGGCAGAGATAGCGCTTAATGTCCCTTCGAAGACCTTCAGGAAGGTCTTTGATCATTTCCATCTC
ATCTTCTCCCCCCATAATTGCCCATCTTTGGCGTTCAAAATGGCGAACTCTTTGTCTTAGTCGAGAAGGCAAC
TGTCTCCGCCTCATCCACCATTCCATGTCTCGACATCTCAGCTGCATTTTTCTCTTCTTTGCCATGACTGCAT
GCAAGAATACCTGGATATTCCCTATCAACAACGTGAAGAGCATCAGGCCACTAAGCACAATGACTATGCTGAA
AATCACTTCTAGCCAGTGACTTGTAGGCTCCAGATCATTGCCGAAGGTGCTGAGAGTCATTAAGCCCCAAAAT
ATGGGATAAAGGATCTTAACAGAAGCTGAGTTGCTAGAAATGACTGGAAGAGCCCACTGGTAGATCCCATAAT
GGAATGGCCCATTAACATCCAAACACAATGGCTTTCTGACCATGGTTGTTGAGTTACCACCACATGGATTTCC
AGCTGTGCCTGTAGGTAACAGAAACTGGTAACAAACCTCCTCGGAGCAAGACAACGAGAGATTGCACTTATCA
CTTCTCTCACACTGTTGCTGGAGGCACGATGCTACACGTTGTATAGCAAGGACATACCAGCATCCTCCAGCAA
CATGAGAGGCAATGAAGTAGGCAATAAGATTGAGGCCAAAACCCCACCAAATGGTGCCAAAGATGTAACCTGT
GACTTTTTGCATTCTTCTCATCAAATAAATGCTGTGATAGACCTTGGGGAGGTACTGGAACAAGAATATTAAT
AGAAGTATTGTCATTATCAT

MLO1;

>XM_022119536.2_*Helianthus_annuus*
ATGGCGGGTGGTGAAGGTGAAGGTACTTCTTTAGAGTTTACACCCACATGGGTTGTGGCTGCCGTTTGTA
CTGTTATTGTTGGGATCTCTCTTGCTGTTGAACGCCTGCTTCATTATACTGGAAAGAAATTGAAGAAGGC
TGGTCAAAAGCCACTCTTTGAAGCCTTGCAAAAGATTAAAGAGGAGTTGATGCTTTTGGGATTTATTTCT
CTTTTATTAACTGTTTTCCAATCAAGAATTGTCAAGATTTGTGTAAAGGAGAGTATAACGGAACACCTTC
TTCCATGCTCACTGCATGATAAAGAAGAGGCGTTAAACCCTAAACCCGAGGGTACTTCTCATGTTCGTCA
TTTACTCGCAGAAGAAGCAGTGGCTGTCGGCTATTGTGCTGCGAAGGGAAAGGTCCCACTTCTATCTCTT
GAGGCTTTGCATCATCTACACATATTTATTTTCGTTCTAGCCGTCGTGCACGTGACGTTTTCTGTCCTCA
CCGTTGTATTTGGAGGCGCTAAGATACGGCAATGGAAGCATTGGGAAGAATCTATCACAAAAGATAACTT
TGATCACTCACAAGTTTTTATTAAGTCGTCGAAATTCACAAATGTAAAAGAGCATGACTTTATTAGGAAC
CGTTTTGTTGGTATTGGCAAAGGCTCAGCGATTCGAGGCTGGATACATTCCTTCTGCAAGCAATTCTATG
GATCTGTCACAAAGACAGATTATGTAGCACTGCGTTTGGGATTTATTACGACCCATTGCAAGGCAAACCC
GAAGTTCAATTTTCACAAATACATGATACGTGCTCTGGAAGATGATTTTAAAAAAGTGGTTGGAATCAGC
TGGTATCTGTGGGTCTTCGTTGTCATATTCTTGTTGCTGAACGTTAATGGCTGGCATACATATTTTTGGA
TAGCTTTCGTTCCGTTCATCCTTCTACTTGCCGTGGGAACAAAGCTAGAGCACATAATAATTCAGTTAGC
TCATGAGGTTGCTGAGAAACATATAGCCATAGAAGGCGATTTAGTTGTGCAACCATCGGATGATCATTTT
TGGTTTCACCGCCCCAAAATTGTGCTTTTTCTCATACATTTCATCCTCTTTCAAAATGCATTTGAGATCG
CATTCTTCTTCTGGATTTGGGTTCAATATGGCTTTGACTCGTGCATAATGGGACAAGTGCGCTATATTAT
CCCTCGGCTCATTATTGGGGTGTTTATTCAGGTTCTGTGCAGTTACAGCACTCTACCACTTTACGCCCTT
GTGACACAGATGGGGACCAATTTCAAGAAAGCAATATTTGAAGACCATATACAGGCCAAAGTTGTGGGCT
GGGCTCTAAATGTCAAGAAAAAGATGGCCAATAACGGTGGGACCAATGGCTCGAGCCATGATGGTTCAAC
TGCCACCACTTCTGCCACTACTGCTGCTGCCACATCTGCTTTTAAGACCATTCAACTTGGTAAAATTAAT
CAGAGCCAGCAGTCGTGA
>NM_001297445.1_*Cucumis_melo*
ATGGGCGGCGGAGGTGAAGGAACGACGCTGGAATTCACTCCGACGTGGGTTGTAGCCGCCGTATGTACCG
TCATCGTTGCCATTTCCCTCGCCTTAGAGCGTCTTCTTCACTTTCTCGGCAGATACCTCAAAAGCAAGAA
TCAAAAGCCGCTCAATGAAGCTCTTCAGAAAGTTAAAGAAGAATTGATGCTTTTGGGGTTCATTTCACTT
CTGCTCACTGTATTTCAAGGCACCATCTCTAAATTGTGTGTTCCTGAGAGTTTGACCGAACATTTACTTC
CGTGTGATCTGAAGGATAAACCGAAAGCTGAACATGGTTCGCCCTCAGGTGAAACCGGTTCGTCAACGAC
GAAGCATTTTCAAACTTTCTTTGTTTCGAGTATTTCTGGTACGGCCAGACGGCTTCTTGCTGAGGGATCT
GCTTCACAGGCTGGTTATTGTGCCAAAAAGAATAAGGTGCCATTGCTATCACTCGAAGCATTGCATCATC

TACATATTTTTATCTTCATCCTAGCTATCGTCCACGTAACATTTTGCGTTCTCACTGTAGTTTTTGGAGG
ATTGAAGATTCGCCAGTGGAAGCATTGGGAGGATTCTATTGCAAAAGAGAATTATGATACTGAACAAGTT
CTAAAACCAAAAGTCACTCATGTCCATCAACATGCTCTTATCAAAGACCACTTTTTGGGCTTTGGTAAAG
ATTCAGCTCTTCTTGGTTGGTTGCATTCCTTTCTCAAGCAATTTTATGCTTCTGTAACAAAATCAGATTA
TGCAACGTTACGGCTTGGTTTCATTATGACGCACTGCAGGGGAAATCCGAAGTTTAATTTTCACAAGTAC
ATGATACGTGCCCTTGAAGATGACTTCAAGCATGTTGTTGGAATCAGTTGGTATCTTTGGATATTCGTGG
TTGTCTTCTTGTTCCTTAATGTCAGTGGTTGGCATACATATTTCTGGATAGCATTCATTCCTTTCGTTCT
TTTGCTTGCTGTGGGAACGAAGCTGGAACATGTGATAACCCAGCTGGCTCATGAGGTTGCAGAGAAGCAC
GTAGCAATTGAAGGTGATCTAGTAGTCCAACCGTCTGATGATCACTTTTGGTTTCAACGTCCTCGTATTG
TTCTCTTCTTGATCCACTTCATACTTTTCCAAAATGCTTTTGAGATTGGATTTTTCTTCTGGATATGGGT
TCAATATGGATTTGACTCGTGCATCATGGGACAAGTCCGCTATATCATTCCAAGGCTCATCATTGGGGTG
TTTGTCCAGGTTCTTTGCAGTTACAGCACCCTTCCGCTATACGCCATTGTCACTCAGATGGGAAGTTCTT
TCAAGAAAGCAATCTTTGATGAACATGTACAAGTAGGGCTAGTTGGGTGGGCTCAGAAGGTGAAGAAAAG
AAAGGGACTTAGAGCAGCTGCTGATGGCTCCAGTCAAGGAGTCAAGGAAGGTGGTTCAACTGTGGGGATT
CAGTTGGGAAATGTTATGCGCAAGGCTTTTGCCCCTCAAGAAATTAAGCCTGATGACTCCAAATCAAATG
ATCTTCCTTAG
>AF384145.1_*Triticum_aestivum*
ATGGCGGACGACGACGAGTACCCCCCAGCGAGGACGCTGCCGGAGACGCCGTCCTGGGCGGTGGCCCTCG
TCTTCGCCGTCATGATCATCGTGTCCGTCCTCCTGGAGCACGCGCTCCATAAGCTCGGCCATTGGTTCCA
CAAGCGGCACAAGAACGCGCTGGCGGAGGCGCTGGAGAAGATCAAGGCGGAGCTCATGCTGGTGGGCTTC
ATCTCGCTGCTGCTCGCCGTGACGCAGGACCCCATCTCCGGGATATGCATCCCCGAGAAGGCCGCCAGCA
TCATGCGGCCCTGCAAGCTGCCCCCTGGCTCCGTCAAGAGCAAGTACAAAGACTACTACTGCGCCAAACA
GGGCAAGGTGTCGCTCATGTCCACGGGCAGCTTGCACCAGCTGCACATATTCATCTTCGTGCTCGCCGTC
TTCCATGTCACCTACAGCGTCATCATCATGGCTCTAAGCCGTCTCAAAATGAGAACCTGGAAGAAATGGG
AGACAGAGACCGCCTCCCTGGAATACCAGTTCGCAAATGATCCTGCGCGGTTCCGCTTCACGCACCAGAC
GTCGTTCGTGAAGCGGCACCTGGGCCTCTCCAGCACCCCCGGCGTCAGATGGGTGGTGGCCTCCTTCAGG
CAGTTCTTCAGGTCGGTCACCAAGGTGGACTACCTCACCTTGAGGGCAGGCTTCATCAACGCGCATTTGT
CGCATAACAGCAAGTTCGACTTCCACAAGTACATCAAGAGGTCCATGGAGGACGACTTCAAAGTCGTCGT
TGGCATCAGCCTCCCGCTGTGGTGTGTGGCGATCCTCACCCTCTTCCTTGACATTGACGGGATCGGCACG
CTCACCTGGATTTCTTTCATCCCTCTCGTCATCCTCTTGTGTGTTGGAACCAAGCTGGAGATGATCATCA
TGGAGATGGCCCTGGAGATCCAGGACCGGGCGAGCGTCATCAAGGGGGGCGCCCGTGGTTGAGCCCAGCAA
CAAGTTCTTCTGGTTCCACCGCCCCGACTGGGTCCTCTTCTTCATACACCTGACGCTATTCCAGAACGCG
TTTCAGATGGCACATTTCGTGTGGACAGTGGCCACGCGCGGCTTGAAGAAATGCTTCCATATGCACATCG
GGCTGAGCATCATGAAGGTCGTGCTGGGGCTGGCTCTTCAGTTCCTCTGCAGCTATATCACCTTCCCGCT
CTACGCGCTCGTCACACAGATGGGATCAAACATGAAGAGGTCCATCTTCGACGAGCAGACGGCCAAGGCG
CTGACAAACTGGCGGAACACGGCCAAGGAGAAGAAGAAGGTCCGAGACACGGACATGCTGATGGCGCAGA
TGATCGGCGACGCGACGCCCAGCCGAGGGGCGTCGCCCATGCCTAGCCGGGGCTCGTCGCCAGTGCACCT
GCTTCACAAGGGCATGGGACGGTCCGACGATCCCCAGAGCACGCCAACCTCGCCAAGGGCCATGGAGGAG
GCTAGGGACATGTACCCGGTTGTGGTGGCGCATCCAGTCCACAGACTAAATCCTGCTGACAGGAGAAGGT
CGGTCTCGTCGTCGGCACTCGATGTCGACATTCCCAGCGCAGATTTTTCCTTCAGCCAAGGATGA
>KT833172.1_*Petunia_x_hybrida*
ATGGAGGCAACTCCCACTTGGGCAGTTGCCGTGGTTTGTTTCATATTGCTGGCTATTTCCATTTTCATTG
AACAAATTATCCATCACCTTGGAGAGTGGTTGTTGAAAAAACACAAGAAGTCTCTTTATGAAGCACTTGA
GAAAATCAAAGCAGAACTAATGTTGTTGGGATTCATATCGCTGCTGTTGACAGTGGTGCAAACGCCTGTT
TCTAACATATGCGTCCCCAAGAGTGTTGGTTATTCTTGGCATCCTTGTAAGACAAGTGAAGAAGACCAGT
CTAAATATGATGATCCTTGTCTGAAAAAGGGAAAAGTCCAATTTGCATCTTCGTATGCAATACACCAGCT
CCATATCTTCATCTTTGTATTGGCAGTTGCTCATATATCATACTGTATAGCAACTTTTGCTTTGGGCAGA
CTAAAGATGAGAAAATGGAGGGCGTGGGAGGATGAAACAAAAACAATTGAGTACCAATTCTACAACGATC
CTGAGAGGTTCAGATTTGCAAGGGAGACGTCATTTGGACGTAGGCATTTTCATTTCTGGAGCAAGTCTCC
CGTGCTACTGTGGATAGTTTGTTTCTTTAGGCAATTCATCACATCAGTAGCAAAAGTTGACTATCTAACC
CTTAGACATGGGTTCATGATGGCACATTTAACTCCACAGAATCAAGATAACTTTGATTTCCAGATATACA
TCAAAAGAGCTGTTGAAAAAGACTTCAAAGTAGTTGTAGGAATAAGTCCAGCATTATGGCTCTTCACAGT
ACTATATTTTCTAACCACTACCAATGGACTATACTCGTACCTTTGGGTGCCATTTATCCCGCTAATAATA
ATATTGCTGGTTGGGACAAAACTTCAAATGATCATAACAGAAATGGGATTAAGGATTGGAGAGAGGGGAG

AAATAGTGAAGGGTGTACCAGTGGTGGAGACAGGAGACCATCTTTTCTGGTTTAATCGCCCTGCGTTTGT
TCTTTTCTTGATTAATTTTGTGCTCTTTCAGAATGCATTTCAAGTTGCTTTCTTTGCTTGGAGTTGGTGG
AAATTTGGTTATCCATCTTGCTTCCACAAAAATGCTGCAGACCTAGCCATAAGGCTGTCCATGGGGGTGA
CCATACAGGTCCTTTGCAGTTATGTGACTCTCCCACTTTATGCCTTGGTCACACAAATGGGTTCATCAAT
GAAACCTATAATCTTTGGTGATAATGTGGCAACTGCTCTTAGAAGCTGGCACAAGACAGCGAAAAAACGG
GTAAAACATGGCGGTTTATCAGAAAACGCCACCCCCGTCTCAAGCAGACCTGCTACACCATTGCACGGTT
CCTCCCCTGTTCACTTATTACGTGGTTACCCACAATATACCGAGGATAGCCTTCAAGCATCTCCTCGGAC
ATCCAATGTCGAACATGAAGGCTGGGCTAATGAATTATCGACTTCTCCCGGTAGAAGAAACGAGATTAAA
GATCAGGAGGGAGAAATCTTTGCATCAAGCTCTATGCAACGTCCCAATTCTGATCAGCATCAAGTTGAAA
TTACATTGTCAGAATTCACATTTGGCAACAAAATCAATTGA
>MK297457.1_*Pisum_sativum*
ATGGCTGAAGAGGGAGTTAAGGAACGAACTTTGGAAGAAACACCAACTTGGGCTGTTGCAGTTGTGTGTC
TTGTGTTGCTAGCTGTTTCAATCTTAATTGAACATATTATTCATGTTATTGGAAAGTGGTTGAAGAAGAG
AAACAAAAATGCTCTTTATGAAGCTTTGGAAAAGATCAAAGGAGAGCTTATGCTACTAGGATTCATATCC
TTGCTTCTAACTGTCTTCCAAGATAATATTTCTAAAATATGCGTATCACAAAAAATTGGATCAACTTGGC
ATCCTTGTTCCACTTCAAACACAAAGGCCAAGGCTAAATCTGATGAATCATTAGACTATAAAACCAACAA
TGATAGAAAACTCTTGGAGTATTTTGATCCTATTCCTCGGAGAATTCTTGCTACAAAAGGATATGATAAA
TGTTTTGATAAGGGTCAAGTTGCATTAGTTTCTGCATATGGAATTCACCAACTCCATATATTCATTTTTG
TGCTGGCACTATTTCATATCCTTCAATGTATAATAACATTAACTTTGGGAAGAATCAAGATGAGGAAGTG
GAAGACTTGGGAAGATGAGACAAGAACAGTTGAATATCAATTTTATAATGATCCTGAGAGGTTTAGGTTT
GCAAGGGACACAACATTTGGAAGAAGGCACTTGAGCATGTGGGCTCAGTCACCTATTTTGTTATGGATTG
TTAGCTTCTTCAGACAATTCTTTGGATCTATCAGTAGAGTTGATTATATGGCTCTTAGGCATGGATTTAT
CATGGCTCATCTTCCTCCAGGACATGATGCACAATTTGATTTCCAAAAGTATATAAGTAGATCAATTGAA
GAGGATTTTAAAGTTGTTGTAGGAATAAGTCCAACTATCTGGCTCTTCACAGTGCTTTTCCTTCTTACAA
ATACTCATGGGTGGTATCTTATTATTGGCTTCCATTTCTTCCACTAA
>KM244717.1_*Solanum_melongena*
ATGGCTAAAGAACGGTCGATGGAGGGAACCCCCACTTGGGCGGTTGCCGTCGTTTGCTTCATCTTGCTGG
CTATTTCCATTTTTATTGAACAAATTATTCATCACCTTGGAGAGTGGTTATTGGAAAAGCACAAAAAGCC
ACTACACGAAGCACTTGAGAAGATCAAAGCAGAACTTATGCTGTTGGGATTCATATCACTGCTGTTGACA
GTGGTGCAAGATCCAGTTTCTAACATATGCGTGCCCAAAACTGTTGGTTATTCGTGGCATCCTTGTAAGG
CACAGGAAGACGACAAGCCTAAGTATGATGACCCTTGTCTAGAAAAGGGAAAAGTCCAATTTGCTTCTTC
ATATGCAATACACCAGCTCCATATCTTCATCTTTGTGTTGGCAGTTGCTCATGTATTGTATTGTATAGCA
ACTTTTGCTTTGGGCAGGCTAAAGATGAGAAAATGGAGGGCCTGGGAAGATGAAACTAAAACAATTGAAT
ACCAATTCTACAACGATCCTGAGAGATTCAGATTTGCAAGGGAGACCTCCTTTGGACGTAGGCATTTGCA
TTTCTGGAGCAAGTCACCGCTGTTGCTCTGGATAGTTTGTTTCTTCAGGCAATTCTTCTCCTCAGTAGCA
AAGGTTGACTATTTAACCCTTAGACATGGGTTCATGATGGCACATTTAACTCCAGAGAATCAAAAGAATT
TTGATTTTCAAATATACATTAACAGAGCAGTTGACAAAGACTTCAAAGTTGTTGTGGGAATAAGTCCAGC
ATTATGGCTCTTCACGGTACTATATTTTCTAACGACTACCGATGGACTATACTCGTACCTTTGGGTGCCA
TTTGTCCCACTCATAATAATATTGCTGGTTGGCACAAAACTTCAAATGATCATAACAGAAATGGGGGTAA
GGATTTCAGAAAGGGGAGACATAGTGAAAGGTGTGCCAGTGGTGGAGACTGGTGACCATCTTTTTTGGTT
TAATCGCCCTGGCCTTGTGCTTTTCTTGATTAATTTTGTGCTCTTTCAGAATGCGTTTCAAGTCGCTTTC
TTTGTTTGGAGTTGGTGGAAATTTGACTTTCCGTCTTGCTTTCACAAGAATGCTGCAGACCTAGCCATAA
GGCTAACCATGGGGGTGATCATACAGGTCCATTGCAGCTATGTGACTCTTCCTCTCTATGCCTTAGTCAC
CCAGATGGGTTCATCAATGAAACCTATCATCTTTGGTGATAATGTGGCAACAGCTCTTAGAAGCTGGCAC
CATATGGCGAAAAAGCGAGTGAAACATGGGCGGCTATCGGGAAACACCACCCCTGTCTCCAGCAGACCGA
CCACACCTTTGCATGGTACTTCCCCGGTTCACTTATTGCGCGGTTACCCACAATACAATGAGGACAGTGT
TCAAGCATCTCCTCGGACATCCAACGTCGAAAATGAAGGGTGGGCTAATGAAATATCTACTGACAATAAA
GATTATCAGGAGGGACATGCCTCCACATCTGTGCGACCTCCCCATGCTCACAACCAGCAAATTGAGATTA
CAATGTCAGATTTTACTTTTGGAAACAAATAA
>KM244716.1_*Nicotiana_tabacum*
ATGGAGGCAACTCCGACTTGGGCAGTTGCCGCAGTTTGCTTCATCTTGCTGGCTATTTCCATTTTCATTG
AACAAATTATTCATCATCTTGGAGAGTGGTTGTTGAAAAAACATAAAAAGCCTCTTTATGAAGCACTTGA
AAAGATCAAAGCAGAACTGATGTTGTTGGGATTCATATCACTGCTGTTGACAGTGGTGCAAAGCCCAGTG
TCTAACTTATGCGTGCCAAAGAGTGTTGGTTATTCTTGGCATCCTTGTAAGTCTGATGAAGCTGCCAAGA

ATAAATATGATGACCCTTGTCTACCAAAGGGAAAAGTCCAATTTGCATCTTCATATGCAATACACCAGCT
CCACATTTTCATCTTTGTCTTGGCAGTTGCTCATGTATTATACTCTATAGCAACTTTTGCTTTAGGCAGG
CTAAAGATGAGAAAATGGAGAGCCTGGGAGGAAGAAACAAAAACAATTGAGTACCAATTCTACAACGATC
CAGAGAGGTTCAGATTTGCAAGGGAGACGTCATTTGGACGTAGGCACTTGCATTATTGGAGCAAGTCTCC
AGTGCTGCTCTGGATAGTTTGTTTCTTCAGGCAATTCTTCTCATCAGTAGCAAAAGTTGACTATCTAACC
CTTAGACATGGGTTCATGATGGCACATTTAACTCCACAGAATCAGGAAAATTTTGATTTCCAGATATACA
TCAATAGAGCAGTTGAAAAAGACTTCAAATTTGTTGTGGAAATAAGTCCAGCATTATGGCTCTTCACAGT
ACTATATTTTCTAACCACTACCAATGGATTGTACTCGTACCTTTGGGTGCCATTTATCCCGTTAGTAATA
ATATTGCTGGTTGGCACAAAACTTGAAATGATAATAGCAGAAATGGGAGTAAGGATTTCAAAGAGGGGAG
ACATAGTGAGAGGTGTACCAGTGGTGGAGACAGGTGACCATCTTTTCTGGTTCAACCGACCTGGCTTTGT
CCTTTTCTTGATTAACTTTGTGCTCTTTCAGAATGCATTCCAAGTTGCTTTCTTCGTTTGGAGTTGGTGG
AAATTTAGTTACCCATCTTGCTTCCACCAGAATGCTGCAGATATAGCCATAAGGCTGACCATGGGGGTGA
TCATACAGGTCCATTGCAGCTATGTGACTCTCCCTCTTTATGCCTTGGTCACACAGATGGGAACATCAAT
GAAACCTATAATCTTTGGTGATAATGTGGCAACAGCTCTTAGAAGCTGGCACAACACGGCGAAAAAGCGG
GTGAAACACGGCCGGCTATCGGAAAACACCACCCCTGTCTCTAGCAGACCGGCCACACCGTTGCATGGTA
CCTCGCCGGTTCACTTATTACGCAGTTACCCACAATATAGTAATGAGGAGAGTCGGACATCCAATGCGGA
AAATGAAGGCTGGGCTAATGAAATACCAACCTCTCCTCGTAGACAAATTGAGAATATTAAAGATGATGAT
CATCAGGAGGGAGAAATCCATGCCTCCAGCTCTGTGCATCAAGTTGAGATTGCAATGTCAGAATTCACAT
TTGGCAACAAAATGAGTTGA
>KF177395.1_*Prunus_armeniaca*
ATGGCAGCCGCAACCTCAGGAAGATCACTAGAGCAAACGCCAACATGGGCTGTTGCCGTAGTCTGTTTTG
TTTTGGTCTTAGTTTCAATCATCATTGAGCACTTGATCGAACTCGTAGCAAAGTGGTTGAAGAAGAAACA
CAAAAGAGCTCTCTATGAAGCTCTAGAAAAGGTCAAATCAGAGCTTATGCTTTTGGGGTTCATATCCTTG
CTCCTAACAGTAGGACAAGGCCCCATATCAAACATTTGCATATCAGAGAAACTTGGCAACACTTGGCATC
CTTGTGGCAAGAAGCAAGAAACCAAAATGAACAAGGATACAGAGCATGACGAAGAAACCCCAAGCAGGAG
ATTACTCTCACTGTTCAATCAGTCTGATGTTGGCGTACGCCGCGTTTTGGCGGCTGCTGGCACGGACAAA
TGTGCACCAAAGGGGAAAGTTCCATTTATATCCGCGGATGGTATTCATCAACTGCATATTTTTATCTTCG
TGTTGGCTGTTTTCCATATCCTTTATTGCATCCTCACCATGGCTTTAGGAAGAGCCAAGATGAGAAGCTG
GAAGCGGTGGGAAAAGGAAACAAGAACAGCTGAGTATCAATTCTCGCATGACCCTGAGAGGTTCAGGTTT
GCAAGGGACACGTCTTTTGGGAGAAGGCATTTAAGCTTCTGGACCCAAACACCTTTCCTCATGTGGATAG
TTTGTTTTTTCAGACAATTTGTTAGGTCGGTTCCTAAAGTTGATTACTTAACCCTGCGGCATGGGTTTAT
CATGGCACATTTGGCACCCCAAAGCCATCAGAAATTCAATTTCCAAAAATACATAAACAGATCCCTTGAA
GAGGATTTCAAGGTGGTTGTGGGAATCAGCCCTCCAATTTGGTTATTTGCTGTGATATTTCTACTCTTCA
ATACTCATGGATGGTATTCTTATCTTTGGCTACCCTTTATCCCATTGATTATCATTCTCTTGGTGGGGAC
CAAGCTACAGGTGATCATAACCAAAATGGGTCTTAGAATCCAAGAAAGAGGAGAGGTTGTGAAGGGGGTT
CCGGTGGTTCAACCTGGTGATCACCTCTTCTGGTTCAACCGGCCTCGCCTCATTCTCTATCTCATCAACT
TCGTTCTCTTTCAGAATGCCTTCCAGCTTGCTTTCTTTGCATGGAGTTGGTATGAATTTAGTTTGAAATC
TTGCTTCCACGAGCACATTGAGGACATAATCATCAGAGTTTCAATGGGGATCCTTATACAGATTCTGTGC
AGTTATGTCACTCTCCCCCTCTATGCCCTTGTCACACAGATGGGTTCAACCATGAAGCCAACCATATTCA
ATGAAAGAGTAGCCGCTGCCCTGCGCAACTGGCATCACACGGCAAGGAAGCACATAAAGCATAACAAAGG
GTCCGTGACCCCAATGTCTAGTAGGCCAGCCACCCCATCCCACCACATGTCCCCTATTCACCTCCTCCGG
AATTATCGGAGTGAGGTGGACAGCTTCTACAACTCACCACGAAGATCGAACTTCGAGGGTGAACGTTGGG
ACACAGAGTCAGCCTCCCCGTCACACCACCTCCATGTGGATGGTACTTCATCCTCCCACCATCACCAAAT
CGAATTGGGAAATGTAGAGCATGAGAAGGATGTTGATGTCAACGAACCAAACTCGGTTCATGCAGCTCCT
ACCACACCAACAACCAATCAACCGGCTCGAATCCAACATGAAATTAATATGGGACAGACCAAAGACTTCT
CGTTTGACAATAGGCAAAGTATATAA
>HQ446457.1_*Medicago_truncatula*
ATGGCTGAAGATAAAGTTTATGAAAGAACTTTGGAAGAAACACCAACTTGGGCTGTTGCAGTTGTGTGTT
TTGTGTTGCTTGCTATTTCAATCGTAATTGAACATATTATTCATGCTATTGGCAAGTGGTTCAAGAAGAA
AAACAAAAAATGCTCTTTATGAAGCATTGGAAAAAGTCAAAGGAGAGCTTATGTTGATGGGATTTATATCC
TTGCTCCTAACTGTGTTCCAAGATTATATTTCCAAAATATGCATATCAGAAAAAGTTGGATCAACTTGGC
ATCCTTGTTCCACTCCAAAAACAAAAACTGCATCTAATGATGAAAATTCTGAGAGTGAAAACCATGATAG
AAAACTCTTGGAATATTTTGATCCTAACCCTAGGCGAATTCTTGCTACAAAAGGATATGATCAATGTGCT
GATAAGGGTAAAGTTGCTTTGGTTTCTGCATATGGAATTCATGAACTCCATATATTCATTTTTGTGCTGG

CAATATTTCATATTCTACAATGTATAATAACACTAGCTTTGGGAAGATTCAAGATGAGGAGGTGGAAGAA
ATGGGAGGATGAAACAAGAACCGTTGAATATCAATTCTATAATGATCCTGAGAGGTTTAGGTTTGCAAGG
GACACAACATTTGGAAGAAGACACTTGAGCATGTGGACCAAGTCACCCATTTCATTATGGATTGTTTGCT
TCTTCAGACAATTCTTTGGATCTATTAGTAGAGTTGATTATTTGGCACTAAGGCATGGATTTATCATGGC
ACATCTTGCCCCAGGAAATGACGCAGAATTTGATTTCCAAAAGTATATTAGTAGATCACTTGAAAAGGAT
TTTAAAGTTGTAGTGGGAATAAGCCCAACTATCTGGTTCTTTGCAGTGCTATTCCTTCTTACAAATACTC
ATGGGTGGTATTCTTCTTATTGGCTTCCATTTCTCCCACTAATTATAATATTATTAGTAGGAGCTAAGCT
ACAAATGATCATAACAAAGATGGGACTAAGGATTCAAGATAGAGGAGAAGTAATCAAGGGTGCACCTGTG
GTTGAGCCAGGAGATCACCTTTTTTGGTTCAATAGTCCCAACCTTCTTCTCTTTATAATTCATCTTGTTC
TCTTTCAGAATGCCTTTCAACTTGCATTTTTTTCTTGGAGTACATATGAATTCTCTATAAATTCTTGCTT
CCACAGAACAACTGCAGATAATGTCATTAGAGTCTCAGTAGGGATTTTAATACAATTTCTATGTAGCTAT
GTCACTTTGCCTCTTTATGCTCTAGTCACACAGATGGGTTCAACCATGAAACCAACCATTTTTAATGAAA
GATTGGCAACAGCACTCAAGAAATGGCACCACACAGCCAAAAAGCAAGTCAAACACAACAAACATTCGAA
CAACACAACACCGTACTCAAGCAGACAATCAACCCCAACACATGGCATGTCACCTGTTCATCTTCTACAT
AGACAAACTTTTGGCAACAGTGACAGTTTACAAACTTCTCCAAGGACTTCTAACTATGAAAATGAGCAAT
GGGATGTTGAAGGAGGAGGATCAACTTCACCAAGAAACAACCAAACAGTAGCATCTGAGATTGAGATACC
AATTGTCGAGTCATTTTCGACAACTGAATTGCCGGTTAGCGTTAGACATGAAATCGGCACAACTTCAAGT
TCAAAAGATTTTTCTTTCGAGAAGCGACACATCGGTAGCAATTAA
>AY967410.1_*Lotus_corniculatus*
ATGGATAAAGTTGCTCAGAAGAAGTTGGAGGAGACACCAACCTGGGCTGTTGCAGTTGTATGCTTTGTGA
TGCTTGCTATCTCAATCATCATTGAACATGGCATCGAAGCTATCGAAAAGTGGTTAGAAAAGAGACACAA
GAAGGCTCTTCATGAAGCAGTTGAAAAGATCAAAGGAGAGCTTATGCTAATGGGATTCATATCCTTCCTC
CTAACGGTGTTTAAGGATCCCATTTCTAATATTTGCATATCAAAACAAGTTGCATCCACTTGGCATCCTT
GTCATCCCGAAGAAAAGAAGAAAGGTCCAGAAGGATATTATGACAAATGTGCAAAAGATGGTAAAGATAA
AGTTGCTTTTATGTCTCAATATGGGATTCACCAGCTCCACATATTCATCTTTGTGCTTGCTATTTTTCAT
ATCCTTCAATGCATCACAACACTAGCTCTGGGTAGAACAAGGATGGCCATGTGGAAGAAGTGGGAAGAGG
AAACAAAGACTCTTGAACATCAATTTGACAATGATCCTGAGAGGTTCAGATTTGCAAGGGATACAACATT
TGGAAGAAGGCACTTGAATTCATGGAGTCAATCACCAATTTCCTTATGGATAGTTAGCTTCTTCAGACAA
TTCTATGGATCAGTTGATAAGGTTGACTATATGGTATTGCGGCATGGATTTATCATTGCCCATCTTGCAC
CGGGAAGTGAATCAAAATTTGATTTCCAAAAGTATATCAGTAGATCAGTTGATGAGGATTTCAAAGTTGT
AGTGGGAATAAGCCCAACCGTATGGTTCTTTGCGGTGCTAATCCTGCTGACTAATACTCATGGGTGGCAT
TCTTATTTGTGGCTTCCTTTTATCCCATTAATTATAATCTTATTGGTGGGTACTAAGCTACAAATGATCA
TAACAAACATGGGGCTAAAGATTCAAGAAAGAGGGGATGTGATCAAAGGTGCACCTTTGGTTGAGCCAGG
AGATGATTTGTTCTGGTTCAATAGACCTCGCCTTATTCTCTCTTTGGTTCATCTTGTTCTCTTTCAGAAT
GCATTTCAACTAGCATTTTTTGCTTGGAGTGCATGTGACAATGATTTCAAGATAAACTCTTGCTTCCACC
GAAGTACTGCAGATGTTGTCATCAGACTTACATTGGGGGTTGTCACCCAAGTTCTATGCAGCTATGTGAC
CCTGCCTCTCTATGCTCTAGTCACACAGATGGGTTCAACCATGAGACCCACCATTTTCCATGACAGGGTA
GCAACAGCACTAAAGAGTTGGCACCACACAGCTAAAAAGCATGTCAAGCACAACAGGGATTCTAATTCTC
ATTCTAACACACCATTCTCAAGCAGGCCAGCAACCCCAACACATGGCATGTCTCCAGTTCACCTGCTTCA
CAAGCACCACAATTATCACAACAGTGATAGTCCACTAGCTTCTCCAAGAGAGTCACCATCCAATTATGAA
ACTGAACAATGGTACCTTGAACCTAATTCCCCTAGCAACCACACAAGAGGTCATGATCAGACCCTGCAGA
TGCAAGTTCTGGGGTCAAGTGCAACTGAATTTTCTCCTGCTGAAGTCCATCATGAAATCACCCCTATTGG
TTTACCAGAGTTCTCATTTGACAAGGCACCAACTAGTAGGGAATAG
>NM_001247885.1_*Solanum_lycopersicum*
ATGGAGGCAACCCCTACGTGGGCAATTGCTGTGGTTTGCTTCATCTTGCTCGCTATTTCTATTTTTATTG
AACAAATTATTCATCACATTGGAGAGTGGTTACTGGAAAAGCGGAAAAAGTCTCTATATGAAGCACTTGA
AAAGATCAAAGCTGAACTTATGCTGTTGGGATTCTTATCACTGTTGTTGACAGTGTTGCAAGATCCAGTT
TCTAACTTATGTGTCCCCAAGAGTGTTGGTTATTCATGGCATCCTTGTATGGCAAAGGAAGATGCCAAGT
CTGAGTATGATGACCCTTGTCTACCAAAGGGAAAAGTGCAATTTGCATCTTCATATGCAATACACCAGCT
CCATATCTTCATCTTTGTATTGGCAGTTGCTCATGTATTGTACTGTATAGCAACTTTTGCTTTGGGCAGG
CTAAAGATGAGAAAATGGAGGGCATGGGAGGATGAAACAAAAACAATGGAGTACCAATTCTACAACGACC
CTGAGAGATTCAGATTTGCAAGGGAGACCTCGTTTGGACGTAGGCATTTGCATTTCTGGAGCAAGTCCCC
CGTGTTGCTCTCGATAGTTTGTTTCTTTCGGCAATTCTTCTCATCAGTTGCAAAAGTTGACTATTTAACC
CTTAGACATGGGTTCATGATGGCACATTTAACTCCACAAAATCAAAATAATTTTGATTTTCAATTATACA

TTAACAGAGCAGTTGACAAAGACTTCAAAGTTGTTGTTGGAATAAGTCCTGCATTATGGCTCTTCACGGT
GCTATATTTTCTGACTACTACCGATCGATTGTACTCGTATCTTTGGGTGCCATTTATCCCACTTGTAATA
ATATTGCTAGTTGGCACAAAACTTCAAATGATCATAACAGAAATGGGAGTAAGGATTTCAGAAAGGGGAG
ACATAGTAAAAGGTGTACCTGTGGTGGAGACTGGTGACCATCTTTTCTGGTTTAATCGCCCTGCCCTTGT
CCTATTCTTGATTAACTTTGTACTCTTTCAGAATGCGTTTCAAGTTGCTTTCTTTTTTTGGAGTTGGTGG
AAATTTGGTTTCCCATCTTGCTTTCATAAGAATGCTGCAGACCTAGCCATAAGGCTAACCATGGGGGTGA
TCATACAGGTCCATTGCAGCTATGTGACTCTCCCTCTTTATGCCTTAGTTACACAGATGGGTTCATCAAT
GAAGCCTATCATCTTTGGTGATAATGTGGCAACAGCTCTTAGAAGCTGGCACCATACAGCGAAAAAACGG
GTGAAACATGGGCTATCAGGACATACCACCCCTGCAAACAGCAGACCAACCACACCATTGCGTGGTACCT
CCCCTGTTCACTTATTACGCGGTTATCCACAATATAATGAGGACAGTGTTCAAGCATCTCCTCGGACATC
CAATGTCGAAAATGAAGGGTGGGCTAATGAAAATCAGGAGGGAGAGATCCTGCAGCATGCCTCCACTGAT
CATAACAAGCAAATTGAGATTACAATGTCAGATTTTACTTTTGGAAACAAATAA
>AY967409.1_*Brassica_rapa*
ATGGCGGATCAAGTAAAGGAGAGGACTTTAGAGGAGACCTCTACGTGGGCAGTCGCTGTGGTTTGCTTCG
TCTTGCTCTTCATTTCCATTGTCCTTGAACACTCCATTCACAAAATTGGAAGTTGGTTCAAACAGAAGCA
CAAGAAGGCTCTTTATGAAGCTCTTGAAAAGGTCAAAGCAGAGCTTATGCTGTTGGGATTCATATCACTA
CTCCTAACAATTGGACAAACACCAATCTCAAACATCTGCATCTCCCAAAACGTTGCATCATCAATGCACC
CTTGCAGCGCCGCACAAGAAGCTGAAAAATACGGCAAGAAAGACTCCGGCAAGAAAGGAGGAGACGACGA
TGAAAAACCAAGTCACAGGCTTCTCCTTGAGTTAGCTGAGTCTTTTATCCCTAGACGAAGTTTAGCCACC
AAAGGTTATGACAAATGCGCTGAGAAGGGGAAAGTGGCTTTTGTATCTGCCTATGGAATCCACCAGCTGC
ATATATTCATCTTCGTGCTCGCAGTGGTTCATGTTATCTACTGCATTGTTACTTATGCTCTTGGAAAGAC
CAAGATGAGGAGGTGGAAGCAGTGGGAGAATGAGACCAAGACTATAGAGTATCAGTACGCCAACGATCCT
GAGAGGTTCAGGTTTGCGAGGGACACATCTTTCGGGAGAAGACATCTCAATTTCTGGAGCAAGACTAGTA
TTACTCTATGGACTGTGTGTTTTTTCAGACAGTTCTTTGGATCTGTCACCAAAGTTGACTACTTAGCTCT
GAGGCATGGTTTCATCACGGCTCATTTTGCTCCAGGGAGTGAAAGAAGTTTTGATTTCCGCAAGTATATT
CAGAGATCATTAGAAGAAGACTTCAAAACTGTTGTTGAAATCAGTCCGGTTATCTGGTTTGTAGCCGTGC
TATTCCTTTTGACCAACACAAATGGACTACGTTCTTACCTCTGGTTGCCATTCATTCCACTAGTTGTGAT
TCTAATAGTTGGAACAAAGCTTCAAGTCATTATAACCAAACTGGGTCTAAGAATCCAAGAAAAAGGTGAT
GTGGTGAGAGGAGCCCCAGTGGTTCAGCCTGGTGATGATCTCTTTTGGTTTGGCCGTCCTCGATTCATCC
TTTTCTTAATCCATTTGGTTCTCTTCACGAATGCATTTCAACTTGCCTTCTTTGCCTGGAGTACGTATGA
GTTCAATCTCAAGAACTGTTTCCATGAAAGCAATGCAGATGTGATTATTAGAATTGTAGTTGGAGTTGTT
GTACAGATACTTTGCAGCTATGTGACTCTTCCACTCTATGCTCTTGTCACTCAGATGGGTACTAAGATGA
AGCCAACGGTGTTCAATGAAAGAGTAGCCACAGCGTTGAAGAAGTGGCATCACACAGCAAAGAAGCAGAC
AAAACATGGAAGACACTCTGAGTCAACCACACCTTACTCTAGCCGACCAACCACACCAACTCATGGCTCA
TCTCCAATCCATCTCCTTCACAACTTCAACCACCGAAGCGTTGAAAGTTTCCCGAACTCTCCTTCTCCTA
GATACTCTAGTCATCATGACGACCACCAGTTTTGGGATCCTGAGTCTCAACGCCAAGAAGCTGGATCTTC
TTCACATCATTCTCTTGCGCATGAAAGCTCGGAAAAGAAACCTGTTCTTGCATCCGTGGAACTTCCTCCT
ATACGGACTAGCAAAAGCTTGAAAGATTTTTCCTTTAAGAGATAG
>AY934528.1_*Capsicum_annuum*
ATGGCGGGAGGAGGGGGAGGAAGATCGTTGGAGCAAACGCCGACGTGGGCGGTTGCCGTAGTTTGTTTTG
CGTTGGTTGCTATTTCTGTCGTAATAGAGTTCATCATCCATCTTATTGGCAAGTGGTTGAAGTCCAAACA
AAAAAGAGCATTATATGAAGCACTTGAGAAGATAAAATCAGAATTAATGTTGTTGGGATTTATATCCCTA
CTACTAACAGTAGGGCAAGATCCAATTTCAAATATTTGTGTATCTGAAAAAATTGCTAGTACATGGCATC
CATGTACTAAGCAAAAAGAAAATGAAATAAATAAAGAAAAGTCCGATGACTTAGAGGGTCATCGCCGGCG
ACTACTTACGGCTTCTGATGGCGGAGTCCGGCGAGTTTTGGCGGCTGTTGGAACCGACAAATGTGCGGAT
AAGGGAAAAGTAGCATTTGTGTCTGCAGATGGAATTCATCAATTACATATTTTTATTTTTGTGCTGGCTC
TTTTTCATATATTTTATTGTATTACTACATTGGCTTTGGGAAGAGCTAAGATGAGTAGTTGGAAGGCATG
GGAAAACGAAACAAGAACAGCTGAGTACCAATTTACAAATGATCCAGAGAGATTTCGATTTGCTAGAGAC
ACATCATTTGGAAGAAGACATTTGAGCTTTTGGACAAAAAATTCAGTGCTTCTATGGATTGTTTGTTTTT
TCAGACAATTTGTAAGATCTGTTCCAAAAGTTGATTATTTGACCCTACGTCATGGTTTTATCATGGCACA
TTTGGCACCTCAGAGCCAAATAAATTTTGATTTCCAAAAATATATTAAGAGGTCATTAGAAGAAGATTTC
AAAGTAGTAGTTAGCATAAGTCCTCCAATTTGGTTCCTTGCTGTATTATTCCTACTCTTCAATACTCATG
GCTGGTATTCTTATCTGTGGCTACCATTCATTCCACTATTTGTGATATTGTTAGTAGGGACCAAATTACA
AGTGATTATAACAAAAATGGGATTAAGAATTCAAGAAAGGGGAGAAGTAGTGAAAGGTGTACCTGTGGTT

CAGCCTGGAGATGATTTATTTTGGTTTAATCGTCCTCGTCTTCTTCTTTATCTAATTAATTTTGTGCTTT
TTCAGAATGCTTTTCAATTGGCTTTCTTTGCTTGGACTTGGTATGAATTTGGGCTGAAATCTTGTTTCCA
TGACAAAACTGAGGATATCGTCATTAGAATGACAATGGGGGTTCTTATTCAAATTCTTTGCAGCTATGTA
ACTCTTCCATTATATGCCCTTGTGACACAGATGGGATCATCAATGAAACCAACAATTTTCAATGAAAGAG
TAGCAACAGCATTGAGGAAGTGGCATCATGGTGCCAAGAAGCACATCAAAGAGATCAACAAACATCATTC
AAATCCAGCAACACCAATGTCAAGTAGGCCAACAACGCCCACTCATGGCATGTCACCTGTCCATCTCCTA
CGCGGGATCCGGACGAGTGACATGGATGTGAGTCCACAAAGATCGAATTATAATGTGGACCATTGGGACA
TCGAGGGGTCGTCATCTCCCACCCGATTCTACCAAGGTGGTGGTGGAGATGGCTCGTCTTCGCCGTCCCA
TATGCATCAAATTATTCAAAGTGGTCATGACTTACGTCATGACGACTCAGAAGGTCACGAGCCTAGTCTG
CCACAAACGGCTCGTGACCAACACGAAGTCAACATTGCCCGTCCAAGGGAATTCTCTTTTGATAAAAGAA
CAACTAGTGTATAA

>KY978601.1_*Rosa_odorata*

ATGGCCGAAGATGCACAAGAAATGCGCTCTTTGGCCTTGACACCAACATGGTCTGTTGCTACTGTGTTGA
CAATTTTTGTGGCTGTTTCTCTGCTTGTGGAGCGCAGCATTCATCGGTTAAGCCATTGGTTGCGGAAAAC
TAATCGTAAGCCCTTGCTTGAAGCTGTGGAGAAAATGAAGGAAGAGTTGATGCTGCTTGGATTTATCTCT
CTTCTTTTGACGGCTACCTCAAGCACGATAGCCAATATTTGCATTCCATCAAACTTCTATGATAGCAAGT
TTTCTCCATGTACAAGAAAAGAGATTGATGAAGAAAATGAAACCAAGACGTCTGAGGAACGCAAACTATT
GATGTTTACTGTTCTGCCTCATACGTTTAGGAGAATGTTAAATGGCTTGAATGCTAATACCTGCAAAGAG
GGTTATGAGCCATTCGTTTCATATCAAGGTCTTGAGCAGTTGCACCGCTTCATCTTTGTCATGGCAATAA
CACATGTATCCTACAGCTGCTTAACGATGTTGCTGGCAATAGTGAAGATTCACGGTTGGAGAGCATGGGA
GGATGTGGCTCGCATGGACAGGCATGATTCATTGACTGAAATCACACGGCAGATGACAATGCGAAGGCAG
TCTACCTTTGGAAATACATCAAATCCACTGGCTAGGAATAGTTTTTTAATCTGGGTGATATGCTTCTTCA
GGCAATTCGGGCATTCAGTGGTTCGTGCTGACTACTTAACACTCCGCAAGGGTTTCATCATGAATCACAA
CCTTCCACCAAAGTATGATTTTCACAGCTATATGATTCGATCTATGGAAGAGGAATTCCAAAGAATAGTT
GGTGTGAGCGCTCCACTCTGGGGATTTGTCGTTGCTTTCATGCTGTTTAATGTACAAGGGTCTAATCTTT
ATTTCTGGATTGCAATCATTCCAATTACTCTAGTCCTTCTTGTGGGAATGAAGTTGCAGCATGTTATTGC
AACCCTTGCTTTGGAGAATGCTGGTATATCTGGATCCTATCCAGGAGCAAAGCTGAGGCCTCGAGATGAT
CTTTTCTGGTTCAAGAAGCCAGAACTTCTGTTGTCCTTGATACATTTTTGTTTGTTCCAGAATGCGTTTG
AGTTGGCTTCATTCTTTTGGTTTTGGTGGCAGTTTGGGTATAGTTCTTGCTTCATTCGGAATCATACACT
TGTGTATTTGAGGCTGATTTTAGGGTTTGCTGGGCAATTCCTGTGCAGCTACAGCACCTTGCCACTGTAT
GCCTTGGTTACTCAGATGGGAACAAATTATAAGGCCGCACTAATCCCACAAAGGATAAGAGAAACCATAC
ATGGGTGGAAAAAAGCAGCTAGGAGGAAAAGAAGGCTAGGCACATACAATGACGATTCAACCATACACAC
ATGTACAGACACAAGCACAGTAGTGTCGGTTGAGGACGATGATCGTGATTTTTTCGATGGCCCAGCAACT
GATGCCAATACACATAATGGAGTTGAGTTGCAACCAGTTTCTAGTGTGATGAACAGTCCTTTCCCAGTTG
CTAACGAAACATCAAGTAGGGCAGGTACACCCCTTCTTCGTCCATCGGCATCTGTTTCTGCATCAGTAGT
ATTGAATTTTCAACCAGAAACTGTTCCAAGATCTGCATCAATGAAGTAG

>EU812233.1_*Vitis_vinifera*

ATGTCTGGCGGTGGAGCCGAAGAAGAGACGACGTTGGAGTACACTCCCACATGGGTGGTGGCGGTTGTCT
GCACCGTCATTGTCGCCATTTCTCTGGCCGTCGAGCGCTTTCTGCACTTTCTCGGCAAGTATTTGAAGAA
GAAGAACCAGAATCCTCTGTTCCAGGCTTTGCAGAAGGTTAAGGAGGAGTTGATGCTTTTGGGGTTCATT
TCCCTGCTGCTGACTGTGTTCCAGAACTTGATCACCAAATTTTGTGTGCCAAAGCATGTCGTGAGTCACC
TGCTGCCTTGCAAGCTTCCGGAAGAAAAGCATGCCACTCAGAGTCTCTCGCACTGGGCACTTGGGAGGCA
TCTGCTCTCCTCTGCACCATCATCTTGCAGCAATGAGAAAGTCCCATTTCTGTCTCTTGAAGCGTTGCAT
CATCTGCATATCTTCATCTTCGTGCTTGCCATTGTCCACGTGACCTTCTGTGTTCTCACTATTATCTTTG
GAGGGGCAAAGATACATCAATGGAAACTTTGGGAGGATTCTATTGCAAAGGATAATTATGATACAGAAGA
GGTTCTGAAGAAAAAGATCACCCATGTTCATGACCATGCATTCATCAAGGATCGTTTTCTTGGTATCGGT
AAAAATTCAGCTGTCATGAGTTGGGTGCATTCATTTTTCAAGCAATTTTATGCATCTGTGACGAAATCAG
ATTACTTGACCTTACGACTAGGATTCATTATGATGCATTGCAGGCAAAACCGCAAGTTTAATTTTCACAA
ATACATGGTACGTGCCCTTGAAGACGATTTTAAGAAAGTTGTTGGTATAAGTTGGTATCTTTGGATCTTT
GTGGTTGTCTTCTTGTTGCTTAATGTTGATGGTTGGCATACTTATTTCTGGATAGCATTCATTCCTTTCA
TTCTTCTACTTGCAGTGGGCGCTAAGCTGGAGCATGTCATTTCCCAGTTGGCCCATGAGGTTGCTGAGAA
ACATGCAGCCATAGAAGGAGAATTGGTTGTTCAACCTTCAGATGACCACTTCTGGTTCCATCGACCCCGA
ATTGTTCTTTTCCTGATCCATATTATTCTCTTCCAAAATTCATTTGAGATGGCATTTTTCTTCTGGATCT
TGTTTACATATGGCTTTGATTCCTGTATAATGGGTAAAGTCCAATACATCATCCCAAGACTCGTTATAGG

GGTGATCATTCAGGTACTCTGCAGCTATAGCACCCTCCCCCTTTATGCCATTGTCACCCAGATGGGGACT
CATTTCAAAAAGGCGATATTTGATGAGCATGTGCAAGCAGGCCTTGTTGGTTGGGCTCAAAAGGCCAGGA
AGAAAACAACGACTGGTAACGCTAACAGAGGACCCACGATTCAGCTTGGAAGAGTTGTGCGTAGTGAAAA
TGCGAAAGAGGAGATAACGCTTACAGGTGCCGCTGAGGAAAACAAGTAA
>NM_001112190.3_*Zea_mays*
ATGGCGGGGGGCGGGGGCGGCCGGGACCTGCCGTCGACGCCGACGTGGGCGGTGGCCCTGGTGTGCGCCG
TCATCGTGCTCGTCTCCGTCGCCATGGAGCATGGCCTCCACAAGCTCGGCCACTGGTTCCATACGCGGCA
GAAGAAGGCCATGCGGGAGGCCCTGGAGAAGATCAAAGCAGAGTTGATGCTGATGGGCTTCATCTCGCTG
CTCCTCGCCGTGGGGCAGACGCCCATCTCCAAGATATGCATCCCGGCCAAGGCTGGCAGCATCATGCTGC
CGTGCAAGCCGCCGAAAGGCGCCGCCGCCGCCGCCGACGACGACAAGAGCGACGGCCGCCGGAGACTCCT
CTGGTACCCGCCGTACCCTGGATACGATGAGCCCGGGCACCACCGCCGTTTCCTCGCCGGCGCGGCTCCG
GACGACAACTACTGCAGTGACCAAGGCAAGGTGTCCCTCATCTCCTCGGCCGGCGTCCACCAGCTGCACA
TCTTCATCTTCGTGCTCGCGGTGTTCCATATCGTCTACAGCGTCGCCACCATGGCGCTGGGGCGTCTCAA
AATGAGGAAATGGAAGAAATGGGAATCGGAGACCAACTCCCTGGAATACCAGTACGCAAACGACCCTTCA
CGGTTCCGGTTCACGCACCAGACGTCGTTCGTGAAGCGGCACCTGGGCCTCTCGAGCACCCCTGGAGTGA
GATGGGTCGTGGCGTTCTTCAGGCAGTTCTTCGCGTCCGTGACCAAGGTGGATTACCTGACCATGCGGCA
GGGGTTCATCAACTACCATCTGTCGCCCAGCACCAAGTTCAACTTCCAGCAGTACATCAAGCGGTCCTTG
GAGGACGACTTCAAAGTCGTCGTTGGCATCAGTCTCCCGCTGTGGTTCGTCGCCATCTTCACTCTCTTGA
TCGATATCAAGGGATTCGGCACGCTTGTCTGGATCTCTTTTGTCCCGCTCGTTATACTCCTGCTAGTTGG
GGCCAAGCTGGAGGTTGTCATCATGGAGATGGCCAAGGAGATACAGGACAAGGCGACGGTCATCAAGGGG
GCGCCTGTGGTGGAGCCAAGTGACAGGTTCTTCTGGTTTAACCGCCCTGGCTGGGTCCTCTTCCTCATCC
ACCTCACGCTCTTCCAGAACGCCTTCCAGATGGCGCATTTCGTTTGGACACTGCTCACCCCAGACCTGAA
GAAATGCTACCACGAGAGGCTGGGCCTGAGCATCATGAAAGTTGCGGTGGGGCTGGTTCTCCAGGTCCTC
TGCAGCTACATCACCTTCCCGCTCTACGCGCTCGTCACGCAGATGGGGTCGCACATGAAGAAGACCATCT
TCGAGGAGCAGACGGCCAAGGCGGTGATGAAGTGGCGCAAGACGGCCAAGGATAAGGTGCGGCAGCGGGA
GGCGGCAGGCTTCCTCGACGTGCTGACGAGCGCCGACACCACGCCGAGCCACAGCCGCGCGACGTCGCCG
AGCCGGGGCAACTCGCCGGTGCACCTGCTCCACAAGTACAGGGGCAGGTCGGAGGAACCGCAGAGCGGGC
CGGCGTCGCCGGGGCGGGAGCTCGGGGACATGTACCCGGTGGCTGACCAGCATCGCCTGCACAGGCTGGA
CCCCGAGAGGATGAGGCCCGCCTCGTCCACCGCCGTCAACATTGACATCGCTGATGCCGATTTTTCTTTT
AGCATGCGGTGA
>NM_001036501.2_*Arabidopsis_thaliana*
ATGGGTCACGGAGGAGAAGGGATGTCGCTTGAATTCACTCCGACGTGGGTCGTCGCCGGAGTTTGTACGG
TCATCGTCGCGATTTCACTGGCGGTGGAGCGTTTGCTTCACTATTTCGGTACTGTTCTTAAGAAGAAGAA
GCAAAAACCCCTTTACGAAGCCCTTCAAAAGGTTAAAGAAGAGCTGATGTTGTTAGGGTTTATATCGCTG
TTACTGACGGTATTCCAAGGGCTCATTTCCAAATTCTGTGTGAAAGAAAATGTGCTTATGCATATGCTTC
CATGTTCTCTCGATTCAAGACGAGAAGCTGGGGCAAGTGAACATAAAAACGTTACAGCAAAAGAACATTT
TCAGACTTTTTTACCTATTGTTGGAACCACTAGGCGTCTACTTGCTGAACATGCTGCTGTGCAAGTTGGT
TACTGTAGCGAAAAGGGTAAAGTACCATTGCTTTCGCTTGAGGCATTGCACCATCTACATATTTTCATCT
TCGTCCTCGCCATATCCCATGTGACATTCTGTGTCCTTACCGTGATTTTTGGAAGCACAAGGATTCACCA
ATGGAAGAAATGGGAGGATTCGATCGCAGATGAGAAGTTTGACCCCGAAACAGCTCTCAGGAAAAGAAGG
GTCACTCATGTACACAACCATGCTTTTATTAAAGAGCATTTTCTTGGTATTGGCAAAGATTCAGTCATCC
TCGGATGGACGCAATCCTTTCTCAAGCAATTCTATGATTCTGTGACGAAATCAGATTACGTGACTTTACG
TCTTGGTTTCATTATGACACATTGTAAGGGAAACCCCAAGCTTAATTTCCACAAGTATATGATGCGCGCT
CTAGAGGATGATTTCAAACAAGTTGTTGGTATTAGTTGGTATCTTTGGATCTTTGTCGTCATCTTTTTGC
TGCTAAATGTTAACGGATGGCACACATATTTCTGGATAGCATTTATTCCCTTTGCTTTGCTTCTTGCTGT
GGGAACAAAGTTGGAGCATGTGATTGCACAGTTAGCTCATGAAGTTGCAGAGAAACATGTAGCCATTGAA
GGAGACTTAGTGGTGAAACCCTCAGATGAGCATTTCTGGTTCAGCAAACCTCAAATTGTTCTCTACTTGA
TCCATTTTATCCTCTTCCAGAATGCTTTTGAGATTGCGTTTTTCTTTTGGATTTGGGTTACATACGGCTT
CGACTCGTGCATTATGGGACAGGTGAGATACATTGTTCCAAGATTGGTTATCGGGGTCTTCATTCAAGTG
CTTTGCAGTTACAGTACACTGCCTCTTTACGCCATCGTCTCACAGATGGGAAGTAGCTTCAAGAAAGCTA
TATTCGAGGAGAATGTGCAGGTTGGTCTTGTTGGTTGGGCACAGAAAGTGAAACAAAAGAGAGACCTAAA
AGCTGCAGCTAGTAATGGAGACGAAGGAAGCTCTCAGGCTGGTCCTGGTCCTGATTCTGGTTCTGGTTCT
GCTCCTGCTGCTGGTCCTGGTGCAGGTTTTGCAGGAATTCAGCTCAGCAGAGTAACAAGAAACAACGCAG
GGGACACAAACAATGAGATTACACCTGATCATAACAACTGA

>GT030035.1_*Coffea_arabica_x_Coffea_canephora*
TGCAAGTAGTCGCATGACCTCAGATCAGTACGATACGGGCTAGTACAGTACAGTCCATGACAAGTACCAC
TTTATCACATCCGTTCGCGCCAGCCCTCGGCTTAACCTATCGTTATTGCAACATTCATCAGAGAGTGGTT
ACTGGAAGGATACAGTCTCTATATGGCACTTGAAGATCAAAGCTGAACTTATGCTTTTGCTACAAGGGAT
TTATCCATTCATTGCCGTTTCCTTACTGTAGTGCAACTGCCAGAACCGGCATTTCTGCCTGACAACTTAT
GTGTCCGGAAGAGTGTTCTGATTATTCATGGCATCCTCCCTTGTATGGCAAAGCGCCAAGATTTGGATGG
TAATTTATCTCTAACATACCCCATGCCGCGGTAAACTTCGTGGTTCTCATCATACTGCAACTACACCAGC
TCCTTATATCTTCATCTTTGTATTGGCAGTTGCTCATGTATTGTACTGTATAGCAACTTTTGCTTTGGGC
AGGCTAAGCAGATGAGAATGCAATGGAGGGGGATGATAACACAATGGAGTACCAATTCTACACGC
>NM_116494.5_*Arabidopsis_thaliana*
ATGGGTCACGGAGGAGAAGGGATGTCGCTTGAATTCACTCCGACGTGGGTCGTCGCCGGAGTTTGTACGG
TCATCGTCGCGATTTCACTGGCGGTGGAGCGTTTGCTTCACTATTTCGGTACTGTTCTTAAGAAGAAGAA
GCAAAAACCCCTTTACGAAGCCCTTCAAAAGGTTAAAGAAGAGCTGATGTTGTTAGGGTTTATATCGCTG
TTACTGACGGTATTCCAAGGGCTCATTTCCAAATTCTGTGTGAAAGAAAATGTGCTTATGCATATGCTTC
CATGTTCTCTCGATTCAAGACGAGAAGCTGGGGCAAGTGAACATAAAAACGTTACAGCAAAAGAACATTT
TCAGACTTTTTTACCTATTGTTGGAACCACTAGGCGTCTACTTGCTGAACATGCTGCTGTGCAAGTTGGT
TACTGTAGCGAAAAGGGTAAAGTACCATTGCTTTCGCTTGAGGCATTGCACCATCTACATATTTTCATCT
TCGTCCTCGCCATATCCCATGTGACATTCTGTGTCCTTACCGTGATTTTTGGAAGCACAAGGATTCACCA
ATGGAAGAAATGGGAGGATTCGATCGCAGATGAGAAGTTTGACCCCGAAACAGCTCTCAGGAAAAGAAGG
GTCACTCATGTACACAACCATGCTTTTATTAAAGAGCATTTTCTTGGTATTGGCAAAGATTCAGTCATCC
TCGGATGGACGCAATCCTTTCTCAAGCAATTCTATGATTCTGTGACGAAATCAGATTACGTGACTTTACG
TCTTGGTTTCATTATGACACATTGTAAGGGAAACCCCAAGCTTAATTTCCACAAGTATATGATGCGCGCT
CTAGAGGATGATTTCAAACAAGTTGTTGGTATTAGTTGGTATCTTTGGATCTTTGTCGTCATCTTTTTGC
TGCTAAATGTTAACGGATGGCACACATATTTCTGGATAGCATTTATTCCCTTTGCTTTGCTTCTTGCTGT
GGGAACAAAGTTGGAGCATGTGATTGCACAGTTAGCTCATGAAGTTGCAGAGAAACATGTAGCCATTGAA
GGAGACTTAGTGGTGAAACCCTCAGATGAGCATTTCTGGTTCAGCAAACCTCAAATTGTTCTCTACTTGA
TCCATTTTATCCTCTTCCAGAATGCTTTTGAGATTGCGTTTTTCTTTTGGATTTGGGTTACATACGGCTT
CGACTCGTGCATTATGGGACAGGTGAGATACATTGTTCCAAGATTGGTTATCGGGGTCTTCATTCAAGTG
CTTTGCAGTTACAGTACACTGCCTCTTTACGCCATCGTCTCACAGATGGGAAGTAGCTTCAAGAAAGCTA
TATTCGAGGAGAATGTGCAGGTTGGTCTTGTTGGTTGGGCACAGAAAGTGAAACAAAAGAGAGACCTAAA
AGCTGCAGCTAGTAATGGAGACGAAGGAAGCTCTCAGGCTGGTCCTGGTCCTGATTCTGGTTCTGGTTCT
GCTCCTGCTGCTGGTCCTGGTGCAGGTTTTGCAGGAATTCAGCTCAGCAGAGTAACAAGAAACAACGCAG
GGGACACAAACAATGAGATTACACCTGATCATAACAACTGA
>NM_001112190.3_*Zea_mays*
ATGGCGGGGGGCGGGGGCGGCCGGGACCTGCCGTCGACGCCGACGTGGGCGGTGGCCCTGGTGTGCGCCG
TCATCGTGCTCGTCTCCGTCGCCATGGAGCATGGCCTCCACAAGCTCGGCCACTGGTTCCATACGCGGCA
GAAGAAGGCCATGCGGGAGGCCCTGGAGAAGATCAAAGCAGAGTTGATGCTGATGGGCTTCATCTCGCTG
CTCCTCGCCGTGGGGCAGACGCCCATCTCCAAGATATGCATCCCGGCCAAGGCTGGCAGCATCATGCTGC
CGTGCAAGCCGCCGAAAGGCGCCGCCGCCGCCGCCGACGACGACAAGAGCGACGGCCGCCGGAGACTCCT
CTGGTACCCGCCGTACCCTGGATACGATGAGCCCGGGCACCACCGCCGTTTCCTCGCCGGCGCGGCTCCG
GACGACAACTACTGCAGTGACCAAGGCAAGGTGTCCCTCATCTCCTCGGCCGGCGTCCACCAGCTGCACA
TCTTCATCTTCGTGCTCGCGGTGTTCCATATCGTCTACAGCGTCGCCACCATGGCGCTGGGGCGTCTCAA
AATGAGGAAATGGAAGAAATGGGAATCGGAGACCAACTCCCTGGAATACCAGTACGCAAACGACCCTTCA
CGGTTCCGGTTCACGCACCAGACGTCGTTCGTGAAGCGGCACCTGGGCCTCTCGAGCACCCCTGGAGTGA
GATGGGTCGTGGCGTTCTTCAGGCAGTTCTTCGCGTCCGTGACCAAGGTGGATTACCTGACCATGCGGCA
GGGGTTCATCAACTACCATCTGTCGCCCAGCACCAAGTTCAACTTCCAGCAGTACATCAAGCGGTCCTTG
GAGGACGACTTCAAAGTCGTCGTTGGCATCAGTCTCCCGCTGTGGTTCGTCGCCATCTTCACTCTCTTGA
TCGATATCAAGGGATTCGGCACGCTTGTCTGGATCTCTTTTGTCCCGCTCGTTATACTCCTGCTAGTTGG
GGCCAAGCTGGAGGTTGTCATCATGGAGATGGCCAAGGAGATACAGGACAAGGCGACGGTCATCAAGGGG
GCGCCTGTGGTGGAGCCAAGTGACAGGTTCTTCTGGTTTAACCGCCCTGGCTGGGTCCTCTTCCTCATCC
ACCTCACGCTCTTCCAGAACGCCTTCCAGATGGCGCATTTCGTTTGGACACTGCTCACCCCAGACCTGAA
GAAATGCTACCACGAGAGGCTGGGCCTGAGCATCATGAAAGTTGCGGTGGGGCTGGTTCTCCAGGTCCTC
TGCAGCTACATCACCTTCCCGCTCTACGCGCTCGTCACGCAGATGGGGTCGCACATGAAGAAGACCATCT
TCGAGGAGCAGACGGCCAAGGCGGTGATGAAGTGGCGCAAGACGGCCAAGGATAAGGTGCGGCAGCGGGA

GGCGGCAGGCTTCCTCGACGTGCTGACGAGCGCCGACACCACGCCGAGCCACAGCCGCGCGACGTCGCCG
AGCCGGGGCAACTCGCCGGTGCACCTGCTCCACAAGTACAGGGGCAGGTCGGAGGAACCGCAGAGCGGGC
CGGCGTCGCCGGGGCGGGAGCTCGGGGACATGTACCCGGTGGCTGACCAGCATCGCCTGCACAGGCTGGA
CCCCGAGAGGATGAGGCCCGCCTCGTCCACCGCCGTCAACATTGACATCGCTGATGCCGATTTTTCTTTT
AGCATGCGGTGA
>XM_021806459.1_*Hevea_brasiliensis*
ATGAGTGGTGGAGGGAGTGAGGAGGGAGAGTCGTTAGAGTTCACACCCACATGGGTTGTTGCTGCTGTGT
GCACTGTGATTGTTGCCATTTCTCTTGCTGCAGAGAGATTTCTCCATTATGGTGGCAAATATCTTAAGAG
CAAGAACCAGAAACCCCTGTATGAAGCTCTCCAAAAAATTAAAGAAGAGTTGATGCTTCTGGGTTTCATA
TCATTGTTATTGACAGTATCTCAGGGCACAATCTCTAAATTCTGTGTACATGAGCATGTGCTTACTAATA
TGCTTCCTTGTGATCTCTCTGAGAAAGGAGAAGAAGGACAAGGATCTAATACTACAGCTACAACCGAACA
TTTTCAGAGGTTCTTTACAACTAGCATTTCTGGCACTAACAGGCGCCTTCTGGCTGAATCAACAGAATCC
CAGATTGGTTACTGTGAGAAGAAGGGTAAGGTGCCACTGTTATCCATAGAAGCTTTACATCATCTACATA
TCTTTATCTTCGTCCTAGCCATTGTCCATGTCACTTTCAGTGTGCTCACTATTCTTTTTGGAGGGGCAAG
GATTCGTCAATGGCAACACTGGGAGAATTCAATTGCAAAAGATCGATATGATACAGATGAAGTTTTGAAA
AAGAAGTTCACCAATGTCCATCAACACACATTTATCCAGGAGCATTTTCTTGGAATTGGTAAAGATTTTG
CTCTGTTGGGGTGGGTGCATTCCTTTTTCAAGCAATTTTATGCATCTGTGACAAAATCAGATTACATAAC
TCTGCGACTAGGTTTCATCATGACACATTGCAGAGGAAGTCCAAAGTTTAACTTTCACAGATACATGGTA
CGTGCCCTTGAAGATGACTTTAAGACAGTTGTTGGTATAAGTTGGTATCTTTGGATATTTGTGGTCATCT
TCTTATTGCTGAATGTTAATGGTTGGCATACATATTTCTGGATAGCATTCCTTCCTTTCCTTCTTCTACT
TGCTGTTGGCACCAAGTTGGAGCATGTAATCACCCAATTGGCTCATGATGTTGCTGAGAAACATGTAGCC
ATAGAAGGGGACTTAGTAGTTAAACCATCAGATGAGCACTTTTGGTTCAACCGACCTGACATTGTCTTGT
TCTTGATTCATTTCATCCTCTTCCAAAATGCTTTTGAGATTGCATTTTTCTTCTGGATATGGGTTCAATA
TGGCTTTGATTCCTGCATAATGGGACAAGTCCGATATATTGTCCCCAGGCTAATTATTGGATGGGAAGTT
CATACAAGAAAGCAATATTTGATGAGCATGTCCAAGCTGGCCTTGTCGGTTGGGCTGAGAAGGTGA
>XM_021806451.1_*Hevea_brasiliensis*
ATGAGTGGTGGAGGGAGTGAGGAGGGAGAGTCGTTAGAGTTCACACCCACATGGGTTGTTGCTGCTGTGT
GCACTGTGATTGTTGCCATTTCTCTTGCTGCAGAGAGATTTCTCCATTATGGTGGCAAATATCTTAAGAG
CAAGAACCAGAAACCCCTGTATGAAGCTCTCCAAAAAATTAAAGAAGAGTTGATGCTTCTGGGTTTCATA
TCATTGTTATTGACAGTATCTCAGGGCACAATCTCTAAATTCTGTGTACATGAGCATGTGCTTACTAATA
TGCTTCCTTGTGATCTCTCTGAGAAAGGAGAAGAAGGACAAGGATCTAATACTACAGCTACAACCGAACA
TTTTCAGAGGTTCTTTACAACTAGCATTTCTGGCACTAACAGGCGCCTTCTGGCTGAATCAACAGAATCC
CAGATTGGTTACTGTGAGAAGAAGGGTAAGGTGCCACTGTTATCCATAGAAGCTTTACATCATCTACATA
TCTTTATCTTCGTCCTAGCCATTGTCCATGTCACTTTCAGTGTGCTCACTATTCTTTTTGGAGGGGCAAG
GATTCGTCAATGGCAACACTGGGAGAATTCAATTGCAAAAGATCGATATGATACAGATGAAGTTTTGAAA
AAGAAGTTCACCAATGTCCATCAACACACATTTATCCAGGAGCATTTTCTTGGAATTGGTAAAGATTTTG
CTCTGTTGGGGTGGGTGCATTCCTTTTTCAAGCAATTTTATGCATCTGTGACAAAATCAGATTACATAAC
TCTGCGACTAGGTTTCATCATGACACATTGCAGAGGAAGTCCAAAGTTTAACTTTCACAGATACATGGTA
CGTGCCCTTGAAGATGACTTTAAGACAGTTGTTGGTATAAGTTGGTATCTTTGGATATTTGTGGTCATCT
TCTTATTGCTGAATGTTAATGGTTGGCATACATATTTCTGGATAGCATTCCTTCCTTTCCTTCTTCTACT
TGCTGTTGGCACCAAGTTGGAGCATGTAATCACCCAATTGGCTCATGATGTTGCTGAGAAACATGTAGCC
ATAGAAGGGGACTTAGTAGTTAAACCATCAGATGAGCACTTTTGGTTCAACCGACCTGACATTGTCTTGT
TCTTGATTCATTTCATCCTCTTCCAAAATGCTTTTGAGATTGCATTTTTCTTCTGGATATGGGTTCAATA
TGGCTTTGATTCCTGCATAATGGGACAAGTCCGATATATTGTCCCCAGGCTAATTATTGGGGTGTTCATT
CAGATACTATGCAGTTACAGCACCCTTCCACTTTATGCCATTGTCACACAGATGGGAAGTTCATACAAGA
AAGCAATATTTGATGAGCATGTCCAAGCTGGCCTTGTCGGTTGGGCTGAGAAGGTGAAGAGAAAGAAAGG
CCTAAAAGGAGCAACAGCAGCAGCAAGAGGTGGATCTAACCAACCAAGTTCTCATGAAAGTTCTTCTCTG
GGAATTCAGCTTGGAAGGGTCGGGCACAATGGGTCAACTCAAGAGATTCAACCTTCAGCTGGTTCTGAGG
GGCAGACATAA
>XM_015786516.2_*Oryza_sativa*
ATGGCAGGTGGGAGATCGGGATCGCGGGAGTTGCCGGAGACGCCGACGTGGGCGGTGGCCGTCGTCTGCG
CCGTCCTCGTGCTCGTCTCCGTCGCCATGGAGCACGGCCTCCACAACCTCAGCCATTGGTTCCGTAGGCG
GCAGAAGAAGGCCATGGGCGACGCCCTCGACAAGATCAAAGCAGAGCTGATGCTGCTGGGCTTCATATCC
CTGCTTCTCACCGTGGCACAGGCGCCCATCTCCAAGATCTGCATCCCCAAGTCGGCTGCCAACATCTTGT

TGCCGTGCAAGGCAGGCCAAGATGCCATCGAAGAAGAAGCAGCAAGTGATCGCCGGTCCTTGGCCGGCGC
CGGCGGCGGGGACTACTGCTCGAAATTCGATGGCAAGGTGGCGCTGATGTCGGCAAAGAGCATGCACCAG
CTGCACATTTTCATCTTCGTGCTCGCCGTGTTCCATGTTACCTACTGCGTCATCACCATGGGTTTAGGGC
GCCTCAAAATGAAGAAATGGAAGAAGTGGGAGTCACAGACCAACTCATTGGAGTATCAGTTCGCAATCGA
TCCTTCACGATTCAGGTTCACGCATCAGACGTCGTTCGTGAAGCGGCATCTGGGATCATTCTCAAGCACC
CCTGGGCTCAGATGGATCGTAGCATTCTTCAGGCAGTTCTTTGGGTCCGTCACCAAGGTGGACTACCTGA
CCATGCGGCAAGGCTTCATCAATGCGCATTTGTCGCAGAATAGCAAGTTCGACTTCCACAAATACATCAA
GAGGTCTTTGGAGGACGACTTCAAAGTTGTCGTTGGCATCAGCCTCCCTCTGTGGTTCGTCGGAATCCTT
GTACTCTTCCTCGATATCCACGGTCTTGGCACACTTATTTGGATCTCTTTTGTTCCTCTCATCATCGTCT
TGTTAGTTGGGACCAAGCTAGAGATGGTGATCATGCAGATGGCCCAAGAGATACAGGACAGGGCCACTGT
GATCCAGGGAGCACCTGTGGTTGAACCAAGCAACAAGTACTTCTGGTTCAACCGCCCTGACTGGGTCTTG
TTCTTCATACACCTGACACTCTTCCATAACGCATTTCAGATGGCGCATTTCGTATGGACTATGGCAACAC
CTGGTCTGAAGAAATGCTTCCATGAAAATATTTGGCTGAGCATCGTGGAAGTCATTGTGGGGATCTCTCT
TCAGGTGCTATGCAGCTACATCACCTTCCCGCTCTACGCGCTCGTCACACAGATGGGATCGAACATGAAG
AAGACAATCTTCGAGGAGCAAACGATGAAGGCGCTGATGAACTGGAGGAAGAAGGCGATGGAGAAGAAGA
AGGTCCGGGACGCGGACGCGTTCCTGGCGCAGATGAGCGTCGACTTCGCGACGCCGGCGTCGAGCCGGTC
CGCGTCGCCGGTGCACCTGCTGCAGGATCACAGGGCGAGGTCGGACGACCCGCCGAGCCCAATCACGGTG
GCCTCACCACCGGCACCGGAGGAGGACATATACCCGGTGCCGGCGGCGGCTGCGTCTCGCCAGCTGCTAG
ACGACCCGCCGGACAGGAGGTGGATGGCATCCTCGTCGGCCGACATCGCCGATTCTGATTTTTCCTTCAG
CGCACAACGGTGA
>XM_026026028.1_*Oryza_sativa*
ATGTACCCGCGCGCGCACGCGGTGTGCTCATCTCTCGAGTTAATTTGGTTGTTGTTGTTGTTGTGTTCTT
GTGACATCTCAATTAACATCCGATCCTGGTCGATCGATCGCCCTGTGGTGGCGCTACTGCTTGCATTGCA
GTGGTTCCGTAGGCGGCAGAAGAAGGCCATGGGCGACGCCCTCGACAAGATCAAAGCAGAGCTGATGCTG
CTGGGCTTCATATCCCTGCTTCTCACCGTGGCACAGGCGCCCATCTCCAAGATCTGCATCCCCAAGTCGG
CTGCCAACATCTTGTTGCCGTGCAAGGCAGGCCAAGATGCCATCGAAGAAGAAGCAGCAAGTGATCGCCG
GTCCTTGGCCGGCGCCGGCGGCGGGGACTACTGCTCGAAATTCGATGGCAAGGTGGCGCTGATGTCGGCA
AAGAGCATGCACCAGCTGCACATTTTCATCTTCGTGCTCGCCGTGTTCCATGTTACCTACTGCGTCATCA
CCATGGGTTTAGGGCGCCTCAAAATGAAGAAATGGAAGAAGTGGGAGTCACAGACCAACTCATTGGAGTA
TCAGTTCGCAATCGATCCTTCACGATTCAGGTTCACGCATCAGACGTCGTTCGTGAAGCGGCATCTGGGA
TCATTCTCAAGCACCCCTGGGCTCAGATGGATCGTAGCATTCTTCAGGCAGTTCTTTGGGTCCGTCACCA
AGGTGGACTACCTGACCATGCGGCAAGGCTTCATCAATGCGCATTTGTCGCAGAATAGCAAGTTCGACTT
CCACAAATACATCAAGAGGTCTTTGGAGGACGACTTCAAAGTTGTCGTTGGCATCAGCCTCCCTCTGTGG
TTCGTCGGAATCCTTGTACTCTTCCTCGATATCCACGGTCTTGGCACACTTATTTGGATCTCTTTTGTTC
CTCTCATCATCGTCTTGTTAGTTGGGACCAAGCTAGAGATGGTGATCATGCAGATGGCCCAAGAGATACA
GGACAGGGCCACTGTGATCCAGGGAGCACCTGTGGTTGAACCAAGCAACAAGTACTTCTGGTTCAACCGC
CCTGACTGGGTCTTGTTCTTCATACACCTGACACTCTTCCATAACGCATTTCAGATGGCGCATTTCGTAT
GGACTATGGCAACACCTGGTCTGAAGAAATGCTTCCATGAAAATATTTGGCTGAGCATCGTGGAAGTCAT
TGTGGGGATCTCTCTTCAGGTGCTATGCAGCTACATCACCTTCCCGCTCTACGCGCTCGTCACACAGATG
GGATCGAACATGAAGAAGACAATCTTCGAGGAGCAAACGATGAAGGCGCTGATGAACTGGAGGAAGAAGG
CGATGGAGAAGAAGAAGGTCCGGGACGCGGACGCGTTCCTGGCGCAGATGAGCGTCGACTTCGCGACGCC
GGCGTCGAGCCGGTCCGCGTCGCCGGTGCACCTGCTGCAGGATCACAGGGCGAGGTCGGACGACCCGCCG
AGCCCAATCACGGTGGCCTCACCACCGGCACCGGAGGAGGACATATACCCGGTGCCGGCGGCGGCTGCGT
CTCGCCAGCTGCTAGACGACCCGCCGGACAGGAGGTGGATGGCATCCTCGTCGGCCGACATCGCCGATTC
TGATTTTTCCTTCAGCGCACAACGGTGA
>XM_015786517.2_*Oryza_sativa*
ATGTACCCGCGCGCGCACGCGTGGTTCCGTAGGCGGCAGAAGAAGGCCATGGGCGACGCCCTCGACAAGA
TCAAAGCAGAGCTGATGCTGCTGGGCTTCATATCCCTGCTTCTCACCGTGGCACAGGCGCCCATCTCCAA
GATCTGCATCCCCAAGTCGGCTGCCAACATCTTGTTGCCGTGCAAGGCAGGCCAAGATGCCATCGAAGAA
GAAGCAGCAAGTGATCGCCGGTCCTTGGCCGGCGCCGGCGGCGGGGACTACTGCTCGAAATTCGATGGCA
AGGTGGCGCTGATGTCGGCAAAGAGCATGCACCAGCTGCACATTTTCATCTTCGTGCTCGCCGTGTTCCA
TGTTACCTACTGCGTCATCACCATGGGTTTAGGGCGCCTCAAAATGAAGAAATGGAAGAAGTGGGAGTCA
CAGACCAACTCATTGGAGTATCAGTTCGCAATCGATCCTTCACGATTCAGGTTCACGCATCAGACGTCGT
TCGTGAAGCGGCATCTGGGATCATTCTCAAGCACCCCTGGGCTCAGATGGATCGTAGCATTCTTCAGGCA

GTTCTTTGGGTCCGTCACCAAGGTGGACTACCTGACCATGCGGCAAGGCTTCATCAATGCGCATTTGTCG
CAGAATAGCAAGTTCGACTTCCACAAATACATCAAGAGGTCTTTGGAGGACGACTTCAAAGTTGTCGTTG
GCATCAGCCTCCCTCTGTGGTTCGTCGGAATCCTTGTACTCTTCCTCGATATCCACGGTCTTGGCACACT
TATTTGGATCTCTTTTGTTCCTCTCATCATCGTCTTGTTAGTTGGGACCAAGCTAGAGATGGTGATCATG
CAGATGGCCCAAGAGATACAGGACAGGGCCACTGTGATCCAGGGAGCACCTGTGGTTGAACCAAGCAACA
AGTACTTCTGGTTCAACCGCCCTGACTGGGTCTTGTTCTTCATACACCTGACACTCTTCCATAACGCATT
TCAGATGGCGCATTTCGTATGGACTATGGCAACACCTGGTCTGAAGAAATGCTTCCATGAAAATATTTGG
CTGAGCATCGTGGAAGTCATTGTGGGGATCTCTCTTCAGGTGCTATGCAGCTACATCACCTTCCCGCTCT
ACGCGCTCGTCACACAGATGGGATCGAACATGAAGAAGACAATCTTCGAGGAGCAAACGATGAAGGCGCT
GATGAACTGGAGGAAGAAGGCGATGGAGAAGAAGAAGGTCCGGGACGCGGACGCGTTCCTGGCGCAGATG
AGCGTCGACTTCGCGACGCCGGCGTCGAGCCGGTCCGCGTCGCCGGTGCACCTGCTGCAGGATCACAGGG
CGAGGTCGGACGACCCGCCGAGCCCAATCACGGTGGCCTCACCACCGGCACCGGAGGAGGACATATACCC
GGTGCCGGCGGCGGCTGCGTCTCGCCAGCTGCTAGACGACCCGCCGGACAGGAGGTGGATGGCATCCTCG
TCGGCCGACATCGCCGATTCTGATTTTTCCTTCAGCGCACAACGGTGA
>MLO1_*Castanea_mollissima*
TCATTTTGAATTTTCAGAACCAGCAGCAGACTGAATCTCCTCCGGAGTTGATGCTTTGCGAAGAGCAGATCCT
AGCTGAATTCCCCTTGAGGTAGCTTCATGGGAACTATCCTGGCCAGAGCCATTGCCAGCTGCCTTTAAACCCT
TCTTTTTCTTTGCCTTCTGAGCCCAACCAACGAGGCCAACTTGCACATGATCATCAAATATTGCCTTCTTGAA
AGAAGTTCCCATCTGTGTGACTATTGCATAAAGTGGCAGGGTGCTGGAACTACAGAGGACCTGAATGAAGACC
CCAATAATAAGCCTTGGGACAATATAACGGACGTGTCCCATTATGCAAGAGTCAAAGCCATATTGAACCCATA
TCCAGAAAAAAAATGCAATCTCAAAAGAATTCTGGAAAAGGATAAAATGGATCAAGAAGAGGACGATGCGGGG
CCGACCAAGCCAAAAGTGCCTATCAGATGGTTTAACTACCAATTCACCTTCTATGGCTACATGTTTCTCAGCA
ACCTCATGAGCCAACTGGGTAATTACATGCTCCAACTTAGTCCCCACAGCAAGTAGAAGAACAAAAGGAAGGA
ATGCTATCCAGAAATACGTATGCCAACCGTTTACATTAAGCAACAAGAAGATGACTACAAATATCCAAAGATA
CCAACTTATACCAACAACTTTCTTAAAATCATCTTCAAGGGCCCGTATCATGTATTTGTGAAAATTAAACTTG
GGGTTGGTTCTGCAATGGGTCACGATAAAACCTAGCCGTAGTGTTGCGTAGTCTGATTTAGTCACAGATCCAT
AAAATTGCTTTACAAAAGAATGCAACCAACCCAAAATAACTGAGTTTTTACCAATACCCAGAAAATGATCCTT
GATAAATGCATGTTGATGGACATCGGTAACTGTTTTTGATTTCAGAACCTGCTGAGTATCATAATTCTTGTTG
GCAAATTCATCCTCCCAACGTTTCCATTGGCGAATCCTTAATCCTCCAAAAACAACAGTGAGAACACAGAAGG
TCACATGGACAATGGCCAGGACGAAGATAAAGATATGCAGATGATGCAACGCTTCAGTTGATAATAATGGTAC
CTTATTCTCGCCACACATGGTTGATTCCTCAAATTCATGTCCTTCTCGTTTACAAGGAAGCATGTGACGTGTC
ACGTCGGTGGGTACACAGATTTTATTTATCACATTCTGGAACACCGTCAGAAGCAACGATATGAACCCCAAAA
GCATCAACTCTTCTTTAACTTTTTGCAAAGCCTCGAAGAGGGGTTTCTGGTTCTTCCTTTTCAAAGTCACGCC
TGCGTAATGAAGGACACGTTCCACGGCAAGAGAAATGGCTACAATCACAGTGCACACACCTGCAACAACCCAG
GTTGGTGTGTACTCTAACGTTGTACCCTCTTCTGCCAT

PMR4;

>XM_034846569.1_*Vitis_riparia*
ATGAGCCTCAGACAGCGCCAGCCGGCGGCTGCCGGTTCGCAGTACGGGGCGAACCGGTCGTCTCAGCCAC
CGAACCCGGAAGAAGAAGCGTACAATATCATTCCCATTCACAATCTCATCGCCGACCACCCCTCACTCCG
CTACCCGGAGGTTCGAGCCGCCGCGTATGCCCTACGCGCTGTCGGGAGTCTCCGAAAGCCGCCGTTCGGA
GCGTGGCACGAGCACATGGACCTCCTCGACTGGCTCGGCCTCTTCTTCGGCTTCCAGAGCGACAACGTTC
GGAACCAGCGAGAGCACCTAGTGCTCCACCTCGCTAACGCTCAGATGCGGCTCCAGCCACCGCCGGACAA
TATCGACACTCTCGACCCCGGCGTGCTCCGGCGATTCCGACGGAAGCTGCTAAGCAACTACTCGGCATGG
TGCTCCTTCCTCGGCCGGAAATCGAACGTTTGGATCCGAGATTCTGCGCCCGATCCGCGTCGTGAATTGC
TCTACACCGGCCTCTATCTGCTCATCTGGGGTGAGTCTGCTAATCTTAGGTTCATGCCTGAATGTATTTC
CTACATATTTCATCACATGGCTATGGAACTTAATCGAATTCTTGAGGATTACATTGATGAAAACACGGGT
CAGCCTGTCCTGCCGTCAATTTCGGGTGAGAATGCGTACCTGGCCCGTGTGGTGAAGCCCATTTACGAGA
CTGTGCGTAATGAGGTTGAGCGAAGCAAAAATGGGACTGCCCCACATAGTGCTTGGAGGAATTACGATGA
TATAAATGAGTATTTTTGGAGCCCTCGGTGTTTTCAGAAGCTCAAGTGGCCAATGGATTTGGGGAGTAAT
TTTTTTATTCTGTCCAGTAAGAGTAAGCATGTGGGTAAGACTGGTTTTGTGGAGCAAAGGTCATTTTGGA
ACTTGTTTAGGAGCTTTGATCGGCTTTGGGTCATGTTGATTTTGTTTCTTCAAGCTGCCATTATTGTTGC
TTGGGAGGGTAAGGAGTATCCATGGCAGGCATTGGAGAGTCGTTATGTTCAGGTCCGGGTTCTCACTGTG

TTTTTCACTTGGAGTGCTTTGCGGCTTCTCCAGTCTCTACTTGATGCTGGGATGCAATATAGCTTGGTTT
CTAGGGAGACTTTGTGGCTTGGGGTGAGGATGGTGATGAAGATTGTAGTTGCTGCAGGGTGGATTATTGT
GTTTGCGGTATTTTATGCGAGAATTTGGACGCAGGAAAACAATGATGGTGGGTGGACAAGCAAAGGGAAT
GCACGGGTGGTGAATTTTCTTGAGGTTGCATTGGTTTTCATCCTCCCGGAGCTTTTGGCATTGGCTCTGT
TTATTGTTCCATGGATAAGGAATTTTTTGGAGGAGAAAAATTGGAGGATCTTTTACTTGTTATCATGGTG
GTTTCAGAGTAGAATTTTTGTGGGCCGTGGGTTGAGGGAAGGTCTTGTGGATAATATAAAGTATTCTTCA
TTCTGGATTTTGGTGCTTGCTACAAAATTTTCATTCAGCTACTTCCTGCAGATTAAACCTATGGTTGCCC
CTTCCAAAGCCCTTTTGAGAATTAAGAATTTAGAATACGAGTGGCATGAGTTCTTTGATAACAGCAATAG
ATTAGCAGTGGGGCTATTGTGGCTTCCTGTGGTTTTGATGTACCTCATGGATTTGAATATTTGGTACTCC
ATTTACTCCTCTTTCTATGGGGCAGTAGTTGGGCTGTTCTCACATTTGGGTGAAATTCGAAACATTCAGC
AACTGAGACTGAGATTCCAGTTCTTTGCAAGTGCAATCAAGTTCAATCTTATGCCAGAGGAGCAACTGTT
ACATGGAAGGGGCATGAGGAACCGATTCAATGATGCTATCCACAGATTGAAGCTACGGTATGGGCTGGGC
CGGCCCTACAAGAAGCTGGAATCTAATCAGGTCGAAGCGACCAAGTTTGCTTTGATATGGAATGAGATAA
TTTCAATTTTCAGGGAAGAAGATATTATCAATGACCATGAGGTCGAGCTGTTGGAACTTCCTCAAAACTC
TTGGAATGTTAGGGTTATTCGCTGGCCGTGTTTGCTCCTTTGCAATGAGCTGCTGCTAGCTCTCAGCCAG
GCAAAAGAATTGGTAGATGCTCCTGATAAGTGGCTTTGGTATAAGATTTGCAAGAATGAGTATAGACGCT
GTGTTGTCATTGAGGCCTATGATAGTATCAAGCACTTACTGCTTCAGATCATCAAGTTTGATACTGAAGA
GCACTCCATTATCAAAGTCTTGTTTCAAGAAATTGATCACTCTCTTGATATTGAGAAGTTTACTAAAACA
TTCAAAATGACTGCTCTGCCCCAGATCCATCTCAAGTTGATATCTCTTCTTAAGCTATTGAATGAGCCTA
AGAAAGATCCTAACAAGGTGGTGAACATTTTACAGGCCCTTTATGAGATTGTAATTCGGAATTTTTTCAA
GGATCAAAGGACCAGTGATCAGCTGAGGGAGGATGGTCTGGCTCCTCGTAATCTGTCTTCCAGCACGGGC
CTCCTCTTTGAAAATGCTGTGGAGTTGCCTGATGCCAATAATGGGACCTTCTATCGGCAGGTACGACGCT
TGCACACAATTCTTACTTCTCATGACTCAATGAACAATATCCCAAAAAATCTGGAGGCAAGGCGCCGGAT
TGCCTTCTTTAGCAATTCCCTGTTCATGAACATGCCCCATGCTCCTCAAGTTGAGAAGATGATGGCTTTC
AGTGTCCTGACCCCTTATTACAATGAAGAAGTACTATACAGTAAAGAACAACTTCGAACTGAGAATGAAG
ATGGGATTTCCATCCTCTATTATTTACAAACAATTTATGATGATGAGTGGAATAATTTCTTGGAACGGAT
GAAGCAAGAAGGAATGAAGGATAAAAATGACTTATGGACAACAAAGCTGAGAGATCTCCGTCTATGGGCC
TCTTTTAGAGGCCAGACGCTCACCCGCACTGTAAGGGGAATGATGTATTATTATCGAGCTCTCAAGATGC
TGGCCTATCTCGATTCTGCATCAGAAAAGGACATTGAAGAAGGATCACACGAACTTGGCTCAGTGAGGCG
AAACAACAGTATTGATGGTTTTAATTCTGAAAGATCACCTTCTTCAAGGAGCTTAAGTAGAGCAAGTAGT
TCAGTGAGTTTGTTATTCAAAGGCCATGAGTATGGGACTGCTTTAATGAAATACACATATGTAGTTGCTT
GCCAGATATATGGGTCTCAGAAGGCAAAAAAAGATCCCCATGCTGAAGAAATTTTGTATTTAATGGAACA
CAATGAAGCCCTTCGAGTTGCCTATGTTGATGAAGTTTTGAAAGGGAGGGATGAGAAAGAATATTACTCT
GTTCTTGTGAAGTATGATCAGCAGCTACAGAAGGAAGTGGAGATCTATCGGGTCAAGTTGCCTGGTCCCT
TGAAGCTAGGAGAGGGAAAACCAGAGAATCAGAACCATGCCCTTATCTTCACTCGTGGGGATGCAGTCCA
GACGATTGATATGAACCAAGACAACTATTTTGAAGAAGCACTCAAAATGCGGAATCTATTGGAAGAATAC
AGGACCTACTATGGTATTAGGAAGCCTACTATTCTAGGAGTCCGGGAACACATTTTTACCGGTTCTGTAT
CATCACTTGCTTGGTTTATGTCTGCCCAAGAATCAAGTTTTGTTACATTGGGACAGCGTGTTTTGGCGAA
TCCTTTGAAGATTCGGATGCATTATGGTCACCCAGATGTGTTTGATAGGTTTTGGTTTCTGACCAGGGGA
GGGATTAGCAAAGCTTCCAGAGTGATTAATATTAGTGAGGACATTTTTGCTGGCTTCAACTGCACATTGC
GAGGTGGAAATGTAACACACCATGAATACATCCAAGTTGGAAAGGGAAGGGATGTTGGGTTGAATCAAAT
ATCCATGTTTGAAGCAAAGGTTGCTAGCGGAAATGGTGAGCAAGTTCTCAGCAGAGATGTCTATAGGTTG
GGTCACAGGTTGGATTTTTTACGTATGCTGTCATTCTTCTACACTACAGTGGGATTCTTTTTCAACACGA
TGCTGGTGGTTCTGACTGTTTATGCTTTTTTATGGGGCCGGCTGTATCTGGCTCTTAGTGGGGTTGAGGG
TTCTGCTCTGGCGGATAAAAGCAGCAACAATAAGGCCCTTGGTACAATCTTGAATCAGCAGTTCATAATC
CAGCTTGGTCTGTTTACTGCACTTCCGATGATTGTGGAAAATTCTCTTGAGCATGGGTTCCTTGCAGCCA
TCTGGGATTTCATAACAATGCAGCTCCAGCTTTCTTCTGTCTTCTACACATTCTCCATGGGAACTCGCAC
TCACTTTTTTGGCCGGACTATTCTTCACGGTGGTGCAAAATACCGAGCAACTGGACGTGGTTTTGTCGTG
CAGCACAAGAGTTTTGCTGAGAACTATAGACTCTATGCTCGTAGCCATTTTGTAAAGGCAATTGAGCTTG
GCTTGATCCTTACAGTTTACGCTGCATACAGTGTCATAGCTACTGACACTTTTGTCTATATAGCCATGAC
CATCACGAGTTGGTTCCTGGTAGTGTCATGGATCATGGCCCCCTTTGTATTTAATCCTTCTGGGTTTGAT
TGGTTGAAGACAGTGGACGACTTCGATGATTTTATGAACTGGATATGGTACCGTGGAGGTGTGTTTGCAA
AGGCTGAGCAAAGTTGGGAAAGATGGTGGTATGAGGAGCAGGATCATCTAAGGACAACTGGTCTTTGGGG
AAAGTTACTGGAGATAATCTTAGATCTTCGTTTCTTCTTCTTTCAGTATGGAATTGTATACCAGCTAGGT

ATTGCTGCTAATAGCACCAGTATTGCTGTTTACTTGCTTTCCTGGATCTATGTGGTTGTTGCTGTGGCCA
TTTCTTTAACAATAGCATATGCTCGGGACAAGTATGCTGCAAAAGACCACATCTACTATCGGCTGGTCCA
ATTCCTTGTTATTTTGCTTGTAATAATTGTCATTGTTGCATTGCTGGAGTTCACCCACTTTAAATTTGTT
GATCTCTTCACCAGTCTGCTGGCATTCGTTCCCACTGGGTGGGGCCTGATTTTAATTGCCCAGGTATTCA
GGCCCTTTCTGCGGCGCACTTCAGCATGGGAGGCCATTATTTCTTTGGCTCGGCTATATGATATTATGTT
TGGTGTGATCGTCATGGCCCCTGTGGCACTACTATCATGGTTGCCTGGGTTCCAATCAATGCAGACAAGG
ATTCTATTCAATGAAGCATTTAGTAGGGGCCTCCACATATCTCAGATTGTGATAGGGAAAAAATCCAAGG
TCTAA
>XM_025787659.2_*Arachis_hypogaea*
ATGAATTTCCGCCAGCGTCAACCCCATGCATCGCCGGTGGCCACCGCTACACCGGTCCGGGAGGAAGAGC
CCTACAACATAATCCCCGTCTACAATCTCCTCGCTGACCACCCCTCCCTCCGGTTTCCGGAAGTCCGCGC
CGCTGCAGCAGCACTACGCGCCGTTGGAGACCTCCGGCGGCCGCCGTTCGGGCAATGGCGACCTCACATG
GACCTCCTTGACTGGCTCGCACTCTTCTTTGGCTTCCAGCGCGACAACGTCCGCAACCAGCGCGAGCACC
TCGTCCTTCACCTCGCCAACGCTCAGATGCGCCTAACGCCGCCGCCGGACAACATCGACACACTCGACGC
CGGCGTCCTCCGTCGCTTCCGCCGGAAGCTCCTCAAAAACTACAACTCTTGGTGCTCCTACCTAGGCAAG
AAGTCCAACATCTGGATCTCCGATCGACGCCGCGGCGGCTCCGCTGACTCTGAGCAGCGCCGTGAGCTCC
TCTACGTGTCCCTCTACCTCCTGATCTGGGGCGAGGCGGCGAACCTCCGCTTTGTTCCGGAGTGCATCTG
CTACATCTTCCACCACATGGCAATGGAGCTGAACAAGATCTTGGAGGATTACATTGACGAGAACACTGGC
CAGCCTGTGATGCCTTCGCTTTCAGGTGAGAACGCTTTCCTCAACCACGTTGTGAAACCTATCTATGAAA
CTATTAGGCGTGAGGTTGATAGTAGTGGGAATGGAACTGCTCCGCACAGTGCTTGGAGGAATTACGACGA
TATCAATGAGTATTTTTGGAGTAGGAGGTGTTTTGAGAAGCTTGGATGGCCCCTTGATATTGGTAGCAAC
TTCTTTGTGACGGGTAGCGGTGGGAAGCGTGTTGGGAAGACAGGGTTTGTGGAGCAGAGGTCGTTTTTAA
ACCTGCTTAGGAGTTTTGATAGACTTTGGGTGATGCTGGTTTTGTTCCTTCAGGCTGCTATTATTGTTGC
TTGGGAGGAGAAGACTTATCCGTGGCAAGCATTGGAGGACAGGAGTGTTCAGGTTAAGGTTTTGACCATT
TTCTTCACGTGGAGTGGCATGAGGTTAGTGCAGTCTCTGCTTGATATGGTGATGCAGTTTAGGTTGGTGA
CAAGGGAGACGATGGGGCAAGGTGTGAGGATGGTGATGAAGGTTATTGTTGCAGCCGGGTGGATTGTTGT
GTTTGGGGTCATGTATGAGAGGATATGGTCTCAGAGAAACCATGATAGGAGGTGGTCAGCAGAAGCGAAT
AGAAGGGTGGTGAATTTTCTGGAGGTTGTGTTTGTTTTCATCATTCCTGAGCTTCTGGCTCTGGCCCTTT
TTATAATTCCCTGGATTAGAAACTTTGTTGAGAATACAAATTGGAGGATCTTTTACATGTTGTCATGGTG
GTTTCAAAGTAGAATCTTTGTTGGTCGTGGCTTGAGGGAAGGTCTTGTGGACAACATTAAGTACACATTG
TTCTGGGTTGTGGTGCTGGCCACAAAATTTTGTTTCAGTTACTTTCTGCAGGTCAAACCCATGGTTGCTC
CAACAAAGGCACTGTTGAAGCTTAGGAATGTTGAATATGAATGGCATGAGTTTATTCATAACAGCAACCG
ATTTGCTGTTGGATTATTGTGGCTTCCGGTTGTTTTGATATATCTAATGGATATACAGATTTGGTATTCA
ATATATTCATCCTTTGTTGGGGCTGCTGTGGGGTTGTTTGCACATTTGGGTGAGATTCGAAATATGCAAC
AGCTGAAATTGAGGTTCCAGTTTTTTGCCAGTGCAATTCAGTTCAATCTCATGCCAGAGGAGCAATTGTT
GAATGCAAGAGGAACATTGAAGAGCAAGTTTAAGGATGCCGTCCACAGGTTGAAGCTCAGATATGGGCTT
GGTCGGCCATTTAAGAAGCTTGAGTCTAACCAGGTTGAGGCCAACAAGTTTGCTTTGATATGGAATGAGA
TAATTTTGTCTTTCAGGGAGGAGGATATCATCTCTGACAGGGAGGTTGAGCTTTTGGAGCTGCCACAGAA
CTCTTGGAATGTCAGGGTTATCCGCTGGCCATGTTTTCTTCTCTGCAATGAGTTGCTGCTTGCACTCAGT
GAAGCCAAAGAACTGGTTGATGAGTCTGACAACAAGCTGACAAGAAAGATGCGCAAGAATGAGTACAGAC
GCTGTGCTGTCATTGAAGCATATGATAGCATAAAGCACTTGCTTCTTGAGATTATCAAACCCAACAGTGA
AGAGCATTCTATTGTGACAGTTCTATTTCAAGAAATTGATCACTCTCTGGAGATTGAGAAATTCACAAAA
ACATTCAAAACCACTGCACTGCCTCTGCTCCATAGCAAGTTGATAAAGCTTGTTGATTTATTAAACAAAC
CCAAGAAAGATGCTAATCAAGTGGTGAATAGCCTTCAGGCCCTTTATGAGATTGCTGTCCGAGATTTTTT
CAGGGAAGAAAGGAAAACCGAACAGCTGAGGGAGGATGGTTTGGCTCAACGTAACCCAGGTTCAGGTCTC
CTTTTTGAGAACGCTATTCAGTTACCTGAGATCAACAATGAGAACTTCTATCGACAGGTTCGGCGTTTGC
ACACAATTCTCACCTCCAGGGATTCGATGCAAAACATCCCCAAAAATCTAGAAGCTAGACGGAGGATTGC
CTTCTTCAGTAATTCACTTTTTATGAACATGCCCCATGCTCCCCAAGTTGAGAAAATGTTGGCTTTCAGT
GTTTTAACACCTTATTACTCGGAAGAAGTATTATACAGCAAAGAACAGCTCAGAACTGAGAATGAAGATG
GGGTTTCAATCCTTTATTATTTGCAGACTATATATGATGATGACTGGAAGAATTTTATGGAAAGGATGCG
TCGGGAGGGGATGGTGAAAGACAGTGATATTTGGGCTGATAAGCTTAGAGAGTTGAGGCTTTGGGCTTCC
TACAGAGGCCAGACACTAACACGGACAGTTAGAGGAATGATGTATTACTACCGGGCCCTCAAGATGCTGG
CTTTCCTGGACTCTGCGTCAGAAATGGATATTCGAGAGGGATCCCGTGAACTTGTTACAATGAGGCAAGA
TAGTTTAGATGTTATGAACGCTGAAAAGTCACCATCTAGGAGTTTAAGCAGAGCAAGTAGTTCAGTAAGT

TTGTTATTCAAAGGCCATGAATATGGGACTGCTATAATGAAATTCACATATGTGGTTGCCTGCCAGATAT
ATGGATCTCAGAAAGCAAAAAAGGATCCTCATGCTGAGGAAATTTTGTATCTGATGAAAAACAACGAAGC
TCTTCGGGTTGCCTATGTTGATGAAATAACCACTAGTAGGGATGAGAAGGAGTACTACTCCGTTCTTGTT
AAGTATGACCAACAATTGCAGAGAGAGGTGGAAATTTACCGCGTAAAGTTGCCTGGTCCCTTGAAGCTTG
GGGAAGGAAAGCCAGAAAATCAAAATCATGCCATCATATTCACTCGTGGTGATGCAGTTCAAACTATTGA
TATGAACCAGGATAACTACTTTGAGGAGGCACTGAAAATGCGAAATCTCTTGGAAGAATACAGGCATTAC
TATGGCATCCGGAAACCAACTATTTTGGGAGTTAGGGAGCATGTTTTTACTGGTTCTGTTTCCTCTCTTG
CTTGGTTCATGTCAGCTCAGGAAACAAGTTTTGTCACCTTAGGACAGAGGGTTTTGGCAAATCCTTTGAA
GGTTAGGATGCATTATGGTCATCCAGATGTATTTGACAGGTTTTGGTTCTTAACACGTGGTGGTATCAGC
AAAGCTTCCAGAGTGATCAACATCAGTGAAGACATTTTTGCTGGCTTTAATTGTACTCTCCGAGGAGGTA
ATGTTACACACCATGAATACATTCAGGTTGGAAAGGGAAGGGACGTTGGATTGAATCAAGTATCAATGTT
TGAAGCAAAGGTTGCTAGTGGGAATGGGGAGCAAGTCCTTAGCAGAGACGTGTATAGATTGGGTCACAGG
CTGGACTTTTTCCGCATGCTCTCTTTCTTCTACACTACTGTGGGATTCTTCTTCAACACAATGATGGTGA
TTCTGACTGTATATGCCTTTCTATGGGGTCGACTTTATCTTGCCCTTAGTGGTGTTGAGGCTGCAATGGA
AAGTAACAGCAATAACAATAAAGCACTTGGTACCATCTTGAATCAGCAGTTCATTATTCAACTTGGACTT
TTCACTGCCCTTCCAATGATTGTAGAGAATTCCCTTGAGCATGGGTTCCTTCAGGCTATCTGGGATTTCT
TGACAATGCAGCTCCAGCTTTCATCAGTTTTTTACACATTCTCAATGGGCACTCGTAGTCATTTCTTTGG
CCGGACTATTTTGCATGGTGGGGCAAAATATCGGGCTACCGGACGTGGATTTGTTGTAGAGCACAAGAGT
TTTGCTGAGAACTATAGACTCTATGCACGAAGCCATTTTGTGAAAGCAATTGAATTGGGGTTAATTCTTG
TAATCTATGCTTCACACAGTCCTGTGGCATCTGACACATTTGTGTATATAGCCATGACCATTACTAGTTG
GTTCTTAGTGGCATCGTGGATTATTGCACCGTTTGTTTTCAATCCTTCTGGCTTTGACTGGTTAAAAACT
GTGTATGATTTCGATGACTTTATGAACTGGATTTGGTACAGAGGAAGTGTGTTTGCTAAGGCTGAACAGA
GCTGGGAAAGGTGGTGGTATGAAGAGCAGGATCATCTAAGAGTAACTGGCCTTTGGGGGAAATGTATGGA
GATAATTTTAGACCTTCGTTTCTTCTTTTTCCAGTATGGAATTGTATATCAGCTAGGAGTTGCTGCTGGA
AGTCACAGTATTGTTGTTTACTTGCTGTCTTGGATTTGTGTGGTTCTTATATTTGGGGTATATATGCTGG
TTGCATATGCCCACAACAAATATGCAGCCAAAGAGCATATATACTATCGGCTGGTCCAGTTCCTCCTCAT
AATTCTCGCAATTCTTGTGATAGTTGCTTTGCTGGAATTCACCAACTTCAAATTTGTGGACATCTTTACT
AGCCTGTTGGCATTCATTCCCACGGGCTGGGGCCTGATACTGATTGCCCAAGTATTTCGGCCATTTTTGG
AATCAACTATAGTTTGGGATGGAGTTGTTTCAGTGGCTCGTCTATATGACATATTGTTAGGAGTCATTAT
TATGGCCCCTGTGGCACTACTATCATGGTTGCCTGGGTTTCAGAACATGCAAACCAGAATGCTTTTCAAT
GATGCATTTAGCAGGGGCCTCCGGATATTCCAAATTATCACAGGGAAAAAGTCTCAAGCTTGA
>XM_025787660.2_*Arachis_hypogaea*
ATGAATTTCCGCCAGCGTCAACCCCATGCATCGCCGGTGGCCACCGCTACACCGGTCCGGGAGGAAGAGC
CCTACAACATAATCCCCGTCTACAATCTCCTCGCTGACCACCCCTCCCTCCGGTTTCCGGAAGTCCGCGC
CGCTGCAGCAGCACTACGCGCCGTTGGAGACCTCCGGCGGCCGCCGTTCGGGCAATGGCGACCTCACATG
GACCTCCTTGACTGGCTCGCACTCTTCTTTGGCTTCCAGCGCGACAACGTCCGCAACCAGCGCGAGCACC
TCGTCCTTCACCTCGCCAACGCTCAGATGCGCCTAACGCCGCCGCCGGACAACATCGACACACTCGACGC
CGGCGTCCTCCGTCGCTTCCGCCGGAAGCTCCTCAAAAACTACAACTCTTGGTGCTCCTACCTAGGCAAG
AAGTCCAACATCTGGATCTCCGATCGACGCCGCGGCGGCTCCGCTGACTCTGAGCAGCGCCGTGAGCTCC
TCTACGTGTCCCTCTACCTCCTGATCTGGGGCGAGGCGGCGAACCTCCGCTTTGTTCCGGAGTGCATCTG
CTACATCTTCCACCACATGGCAATGGAGCTGAACAAGATCTTGGAGGATTACATTGACGAGAACACTGGC
CAGCCTGTGATGCCTTCGCTTTCAGGTGAGAACGCTTTCCTCAACCACGTTGTGAAACCTATCTATGAAA
CTATTAGGCGTGAGGTTGATAGTAGTGGGAATGGAACTGCTCCGCACAGTGCTTGGAGGAATTACGACGA
TATCAATGAGTATTTTTGGAGTAGGAGGTGTTTTGAGAAGCTTGGATGGCCCCTTGATATTGGTAGCAAC
TTCTTTGTGACGGGTAGCGGTGGGAAGCGTGTTGGGAAGACAGGGTTTGTGGAGCAGAGGTCGTTTTTAA
ACCTGCTTAGGAGTTTTGATAGACTTTGGGTGATGCTGGTTTTGTTCCTTCAGGCTGCTATTATTGTTGC
TTGGGAGGAGAAGACTTATCCGTGGCAAGCATTGGAGGACAGGAGTGTTCAGGTTAAGGTTTTGACCATT
TTCTTCACGTGGAGTGGCATGAGGTTAGTGCAGTCTCTGCTTGATATGGTGATGCAGTTTAGGTTGGTGA
CAAGGGAGACGATGGGGCAAGGTGTGAGGATGGTGATGAAGGTTATTGTTGCAGCCGGGTGGATTGTTGT
GTTTGGGGTCATGTATGAGAGGATATGGTCTCAGAGAAACCATGATAGGAGGTGGTCAGCAGAAGCGAAT
AGAAGGGTGGTGAATTTTCTGGAGGTTGTGTTTGTTTTCATCATTCCTGAGCTTCTGGCTCTGGCCCTTT
TTATAATTCCCTGGATTAGAAACTTTGTTGAGAATACAAATTGGAGGATCTTTTACATGTTGTCATGGTG
GTTTCAAAGTAGAATCTTTGTTGGTCGTGGCTTGAGGGAAGGTCTTGTGGACAACATTAAGTACACATTG
TTCTGGGTTGTGGTGCTGGCCACAAAATTTTGTTTCAGTTACTTTCTGCAGGTCAAACCCATGGTTGCTC

CAACAAAGGCACTGTTGAAGCTTAGGAATGTTGAATATGAATGGCATGAGTTTATTCATAACAGCAACCG
ATTTGCTGTTGGATTATTGTGGCTTCCGGTTGTTTTGATATATCTAATGGATATACAGATTTGGTATTCA
ATATATTCATCCTTTGTTGGGGCTGCTGTGGGGTTGTTTGCACATTTGGGTGAGATTCGAAATATGCAAC
AGCTGAAATTGAGGTTCCAGTTTTTTGCCAGTGCAATTCAGTTCAATCTCATGCCAGAGGAGCAATTGTT
GAATGCAAGAGGAACATTGAAGAGCAAGTTTAAGGATGCCGTCCACAGGTTGAAGCTCAGATATGGGCTT
GGTCGGCCATTTAAGAAGCTTGAGTCTAACCAGGTTGAGGCCAACAAGTTTGCTTTGATATGGAATGAGA
TAATTTTGTCTTTCAGGGAGGAGGATATCATCTCTGACAGGGAGGTTGAGCTTTTGGAGCTGCCACAGAA
CTCTTGGAATGTCAGGGTTATCCGCTGGCCATGTTTTCTTCTCTGCAATGAGTTGCTGCTTGCACTCAGT
GAAGCCAAAGAACTGGTTGATGAGTCTGACAACAAGCTGACAAGAAAGATGCGCAAGAATGAGTACAGAC
GCTGTGCTGTCATTGAAGCATATGATAGCATAAAGCACTTGCTTCTTGAGATTATCAAACCCAACAGTGA
AGAGCATTCTATTGTGACAGTTCTATTTCAAGAAATTGATCACTCTCTGGAGATTGAGAAATTCACAAAA
ACATTCAAAACCACTGCACTGCCTCTGCTCCATAGCAAGTTGATAAAGCTTGTTGATTTATTAAACAAAC
CCAAGAAAGATGCTAATCAAGTGGTGAATAGCCTTCAGGCCCTTTATGAGATTGCTGTCCGAGATTTTTT
CAGGGAAGAAAGGAAAACCGAACAGCTGAGGGAGGATGGTTTGGCTCAACGTAACCCAGGTTCAGGTCTC
CTTTTTGAGAACGCTATTCAGTTACCTGAGATCAACAATGAGAACTTCTATCGACAGGTTCGGCGTTTGC
ACACAATTCTCACCTCCAGGGATTCGATGCAAAACATCCCCAAAAATCTAGAAGCTAGACGGAGGATTGC
CTTCTTCAGTAATTCACTTTTTATGAACATGCCCCATGCTCCCCAAGTTGAGAAAATGTTGGCTTTCAGT
GTTTTAACACCTTATTACTCGGAAGAAGTATTATACAGCAAAGAACAGCTCAGAACTGAGAATGAAGATG
GGGTTTCAATCCTTTATTATTTGCAGACTATATATGATGATGACTGGAAGAATTTTATGGAAAGGATGCG
TCGGGAGGGGATGGTGAAAGACAGTGATATTTGGGCTGATAAGCTTAGAGAGTTGAGGCTTTGGGCTTCC
TACAGAGGCCAGACACTAACACGGACAGTTAGAGGAATGATGTATTACTACCGGGCCCTCAAGATGCTGG
CTTTCCTGGACTCTGCGTCAGAAATGGATATTCGAGAGGGATCCCGTGAACTTGTTACAATGAGGCAAGA
TAGTTTAGATGTTATGAACGCTGAAAAGTCACCATCTAGGAGTTTAAGCAGAGCAAGTAGTTCAGTAAGT
TTGTTATTCAAAGGCCATGAATATGGGACTGCTATAATGAAATTCACATATGTGGTTGCCTGCCAGATAT
ATGGATCTCAGAAAGCAAAAAAGGATCCTCATGCTGAGGAAATTTTGTATCTGATGAAAAACAACGAAGC
TCTTCGGGTTGCCTATGTTGATGAAATAACCACTAGTAGGGATGAGAAGGAGTACTACTCCGTTCTTGTT
AAGTATGACCAACAATTGCAGAGAGAGGTGGAAATTTACCGCGTAAAGTTGCCTGGTCCCTTGAAGCTTG
GGGAAGGAAAGCCAGAAAATCAAAATCATGCCATCATATTCACTCGTGGTGATGCAGTTCAAACTATTGA
TATGAACCAGGATAACTACTTTGAGGAGGCACTGAAAATGCGAAATCTCTTGGAAGAATACAGGCATTAC
TATGGCATCCGGAAACCAACTATTTTGGGAGTTAGGGAGCATGTTTTTACTGGTTCTGTTTCCTCTCTTG
CTTGGTTCATGTCAGCTCAGGAAACAAGTTTTGTCACCTTAGGACAGAGGGTTTTGGCAAATCCTTTGAA
GGTTAGGATGCATTATGGTCATCCAGATGTATTTGACAGGTTTTGGTTCTTAACACGTGGTGGTATCAGC
AAAGCTTCCAGAGTGATCAACATCAGTGAAGACATTTTTGCTGGCTTTAATTGTACTCTCCGAGGAGGTA
ATGTTACACACCATGAATACATTCAGGTTGGAAAGGGAAGGGACGTTGGATTGAATCAAGTATCAATGTT
TGAAGCAAAGGTTGCTAGTGGGAATGGGGAGCAAGTCCTTAGCAGAGACGTGTATAGATTGGGTCACAGG
CTGGACTTTTTCCGCATGCTCTCTTTCTTCTACACTACTGTGGGATTCTTCTTCAACACAATGATGG TGA
TTCTGACTGTATATGCCTTTCTATGGGGTCGACTTTATCTTGCCCTTAGTGGTGTTGAGGCTGCAATGGA
AAGTAACAGCAATAACAATAAAGCACTTGGTACCATCTTGAATCAGCAGTTCATTATTCAACTTGGACTT
TTCACTGCCCTTCCAATGATTGTAGAGAATTCCCTTGAGCATGGGTTCCTTCAGGCTATCTGGGATTTCT
TGACAATGCAGCTCCAGCTTTCATCAGTTTTTTACACATTCTCAATGGGCACTCGTAGTCATTTCTTTGG
CCGGACTATTTTGCATGGTGGGGCAAAATATCGGGCTACCGGACGTGGATTTGTTGTAGAGCACAAGAGT
TTTGCTGAGAACTATAGACTCTATGCACGAAGCCATTTTGTGAAAGCAATTGAATTGGGGTTAATTCTTG
TAATCTATGCTTCACACAGTCCTGTGGCATCTGACACATTTGTGTATATAGCCATGACCATTACTAGTTG
GTTCTTAGTGGCATCGTGGATTATTGCACCGTTTGTTTTCAATCCTTCTGGCTTTGACTGGTTAAAAACT
GTGTATGATTTCGATGACTTTATGAACTGGATTTGGTACAGAGGAAGTGTGTTTGCTAAGGCTGAACAGA
GCTGGGAAAGGTGGTGGTATGAAGAGCAGGATCATCTAAGAGTAACTGGCCTTTGGGGGAAATGTATGGA
GATAATTTTAGACCTTCGTTTCTTCTTTTTCCAGTATGGAATTGTATATCAGCTAGGAGTTGCTGCTGGA
AGTCACAGTATTGTTGTTTACTTGCTGTCTTGGATTTGTGTGGTTCTTATATTTGGGGTATATATGCTGG
TTGCATATGCCCACAACAAATATGCAGCCAAAGAGCATATATACTATCGGCTGGTCCAGTTCCTCCTCAT
AATTCTCGCAATTCTTGTGATAGTTGCTTTGCTGGAATTCACCAACTTCAAATTTGTGGACATCTTTACT
AGCCTGTTGGCATTCATTCCCACGGGCTGGGGCCTGATACTGATTGCCCAAGTATTTCGGCCATTTTTGG
AATCAACTATAGTTTGGGATGGAGTTGTTTCAGTGGCTCGTCTATATGACATATTGTTAGGAGTCATTAT
TATGGCCCCTGTGGCACTACTATCATGGTTGCCTGGGTTTCAGAACATGCAAACCAGAATGCTTTTCAAT
GATGCATTTAGCAGGGGCCTCCGGATATTCCAAATTATCACAGGGAAAAAGTCTCAAGCTTGA

>XM_028695156.1_*Dendrobium_catenatum*
ATGAGCCTCCGGCAGCGCACGACTCGCCCGGCTGGGGCCGGCGGGGCTGGGGGCAGAGGATATGGTGCTA
GGGGATACGGCGCAGCGGAGGGGGAGGAAGCCTATAATATAATACCCATCCACAATCTGCTCGCGGAGCA
TCCAGCGCTTCGGTTCCCTGAGGTGCGTGCCGCCATGGCCGCCCTCCGCACCGTTGGTGACCTTCGCAAG
CCACAGTTCATCCGGTGGCAGGAAGGATACGACCTCCTTGACTGGCTTGGCTTCTTTTTCGGTTTCCAGC
CCGACAACGTCCGCAACCAGCGCGAGCACCTAGTTCTCCTCCTCGCTAACGCGCAGATGCGCCTCCAGCC
GCCACCGGACAACATCGATGCCCTTGAACCCAACGTCGCTCGACGTCTCCGCCGCAAGCTTCTCAAAAAC
TATACCGCGTGGTGCTCCTACCTTGGTCGGAAACCTAACGTCTGGATCTCAGACAGACGCCGCTCCGCCA
CAGCTGCTTCCGACTACTCTCGCCGCGACCTCCTCTACACTGCCCTTTACCTCCTCATCTGGGGCGAAGC
TGCTAACCTCCGCTTCGTCCCTGAATGCATCTCCTACATCTTCCACCACATGGCCATGGACCTCAACCGC
ATCCTTGAGGATTACATCGATGAAGCCACTGGTCGCCCCTCCACCCCGAAAATTACCGGTGAGGAGGCCT
TCCTCAACCATGTAGTCAAGCCAATCTACGCCACCATTAAAGCCGAGGTTGACTCCAGCCGCAATGGCAC
CGCACCCCACTCCGCCTGGCGCAACTATGATGACATCAACGAGTACTTCTGGTCTCGCCATTGCTTTGAG
CGCCTTAAATGGCCTCTCGAACTCTCCAAGAACTTCTTTGCCATTCCTCCTGATCGCAACCGAGTTCGAA
AGACTGGCTTCGTCGAGCAGCGCTCATTCTGGAACCTCTTCCGCAGTTTCGACCGTCTCTGGGTAATGCT
AATCCTCTATCTCCAGGCGGCCATCATCGTTGCCTGGGAAGGCAAAACCTATCCTTGGCAAAATCTGCAG
AGCCGTGACGTCCAGGTCAGGACTCTAACTATATTTATCACTTGGGCTGTTCTTCGCTTCTTCCAATCCA
TTTTGGATGCCGGGACGCAATACAGCCTTGTGCGCAGGGAGACTATGTGGCTAGGAGTGAGGATGGTGCT
CAAGAGCGTTGTGGCTGCTGCTTGGACTGTGGTGTTTGGCATTCTGTACGCCAGGGCTTGGGACCAGAAA
AACAGAGACCGCAGATGGTCTGCGGCAGCCAACCAGAGGCTGGTGAACTACTTGGAGGCCGCCGGAGTGT
TTGTGCTGCCGGAGGTGATGGCCCTTCTTCTCTTCATACTGCCCTGGATAAGGAACTTCCTTGAGAAGAC
AAACTGGAAGATCTTTTATGTGCTTACTTGGTGGTTTCAAAGCCGCACTTTTGTTGGTCGTGGCCTGAGA
GAAGGGCTTGTTGACAACGTGAAATACGCCCTGTTTTGGGTTCTTCTTCTAGCCGTGAAGTTCACCTTCA
GCTACTTCCTTCAGATCAAGCCAATGGTCTCTCCAAGCAAATCCATTTACAAGCTTCATGTTGTAGACTA
TCATTGGCATGAGCTCTTTAGTCGGACCAACAGGTTTGCCGTTTTTCTCTTGTGGCTCCCGGTTGTACTG
GTATATCTTATGGATATTTCAATCTGGTATTCAATTTTTTCATCCCTTGTTGGAGCTCTGGTGGGGCTGT
TCTCACACATTGGCGAAATCCGGAATGTTCAGCAGCTAAGGCTGAGGTTTCAGTTCTTTGCAAGTGCGAT
GCAGTTCAATCTGATGCCTGAGGAGCAACTCTTTCATGAGAGGGGTACACTAAGGAGCAAGTTCAAAGAT
GCGGTTTTACGCCTCAAGCTCCGATATGGGTTGGGGAGGCCTTACAAGAAGATTGAATCAAACCAGGTTG
AAGCAACCAGGTTTGCGTTAATATGGAATGAGATTATGGCTACGTTCAGGGAGGAGGATATCATCAGTGA
CAGAGAGTTGGAACTTCTTGAGCTGCCCGCAAATTCTTGGAACATCAGGGTGATTCGGTGGCCTTGCCTG
TTACTCTGCAATGAGCTTCTTCTGGCTCTTAACCAGGCGAAGGAACTGGTAGCGTCGGACCGTGGGCATT
GGAGAAAGATATGCAAAAATGAGTACCGGCGATGTGCGGTTGTGGAGGCTTATGATAGCGTCAAACACCT
GCTGCTTGAGATCATCAAAGAGGAAACTGAGGAGCACAAAATCATTTCACACCTGTTCTTTGGGTTTGAT
GATGCCATCGGCGTGGAGAAATTGACTGCAGAGTATAAATTGGCAGTGTTGCCAGTTATTCACACCAAGC
TGATCACCCTCCTCGATCAACTGCTCAAACCAAATAAGGATCTAAACAAAATAGTAAATACATTACAGAC
TTTATATGATGTTGCCATCCGTGATTTTCCTACTACTAAGAGGAACATGGAGCAGCTAAAGCAAGCAGGA
CTGGTGCCCACGAGACCAAATGCATCTGGTCTTCTTTTTGAGAATGCTATTGAATTGCCTGTCGCAGAAG
ACACAAATTTCTACAGGCAGGTGAGACGGTTGCACACTATTCTTACTTCCAGGGATTCCATGAATGATGT
GCCCAAGAACCTAGAGGCTCGGCGCCGAATTTCCTTCTTCAGCAACTCCTTGTTCATGAACATGCCACGG
GCTCCCCAAGTAGAGAAAATGTTTGCCTTTAGTGTTCTTACCCCATATTATAATGAGGATGTCTTGTTCA
GTAAGGAGCAGCTTCGCACAGAGAATGAAGATGGCATCTCGATCTTATTTTATCTGCAAAAGATTTATGA
TGATGAATGGGCTAACTTTTTAGAACGAATGAGAGGAGAAGGAATGGTGTATGAGTCTGACATATGGGGT
GAGAAGTTGAGGGATCTTCGTCTTTGGGCCTCTAACAGGGGCCAGACTCTCAGTCGCACAGTCAGGGGAA
TGATGTACTACTACAAGGCTCTCAAGATGCTTACTTTTCTTGATTCTGCTTCTGAGATTGACATTAGGGA
AGGATCACGAGAACTTTCTTTACTTGGTTCTTCTCGGGAGACTGTTGCAGATGGATTAGGGGATTCTTTT
AGGTCTGTGTCAGCACGGAATCTCAGCAGAGAAAGCAGTGGTGTGAGTCTATTATTCAAAGGCCATGAGT
ATGGAACAGCCCTCATGAAGTATACTTACGTTGTTGCCTGTCAGATATATGGACAGCAGAAGGCAAAGAA
AGACCCTCATGCTGAAGAAATTTTGTATCTTATGAAGAATAATGAAGCACTCAGGGTGGCCTATGTTGAT
GAAGTTTACACAGGGAGGGAGGAAGTGGAATATTATTCTGTTCTTGTTAAATATGACCAGCAGTTGCAGA
AAGAGGTAGAGATCTACCGGGTCAAGTTGCCTGGACCATTGAAGCTTGGTGAGGGCAAGCCAGAGAACCA
GAACCATGCCCTCATTTTCACAAGAGGTGATGCAGTGCAGACTATAGATATGAACCAAGACAATTACTTT
GAAGAGGCTCTCAAGATGAGAAATTTGTTGGAAGAATACACACATAACTATGGTTCAAGAAAGCCAACCA
TTTTAGGTGTCCGGGAGCATGTTTTTACTGGTTCTGTCTCTTCACTTGCTTGGTTCATGTCTGCACAGGA

AACTAGCTTCGTCACCCTTGGACAGCGGGTCCTAGCAAATCCTCTGAAAGTGAGGATGCACTATGGACAC
CCAGATGTTTTTGATCGGCTCTGGTTTTTGGGTCGTGGCGGTATTAGCAAGGCTTCCAGAGTCATCAACA
TCAGTGAGGATATATTTGCAGGCTTTAACTGCACATTGCGAGGTGGTAATGTAACTCATCATGAGTATAT
CCAGGTTGGCAAGGGACGCGATGTTGGCCTGAATCAGATTTCGATGTTCGAGGCTAAGGTCGCTAGTGGC
AATGGAGAGCAGGCTTTAAGCAGGGATGTCTATAGACTTGGCCATAGGTTGGACTTTTTTAGGATGCTTT
CCTTTTATTACACGACCGTTGGATTTTACTTCAATACCATGTTGGTCGTCCTAACCGTATATTCTTTTGT
ATGGGGACGCCTTTACCTGGCTCTTAGTGGGCTTGAGGCTGCCATAAAGAACAGCGCCGACTCGACAAGC
AATACTGCACTTGGTACAGTTCTGAATCAGCAGTTCATTATTCAGCTGGGTATTTTCACAGCCTTGCCAA
TGATTGTAGAAAACTCACTTGAGCATGGATTCCTTGGGGCGGTTTGGGATTTTCTGACCATGCAGATTCA
GCTTGCATCTGTTTTTTACACCTTCTCCATGGGAACTAAAACTCATTATTTTGGGCGGACAATCCTTCAC
GGAGGTGCAAAATATAGAGCAACTGGGCGCGGCTTTGTTGTGCAGCACAAGAGTTTTTGTGAGAACTACA
GGCTTTATGCACGGAGCCATTTTATTAAGGCTATAGAGCTTGGTGTTGTGCTAACATTGTATGCATCATA
TAGTGCCATTGCGAAGAACACTATTGTTTACATTGTGATGACAATCTCTTCATGGTTTCTGGTGGTGTCA
TGGATAATGGCTCCTTTTGCTTTCAACCCTTCTGGTTTTGACTGGTTGAAGACTGTTGATGACTTCGATG
ATTTTGTGAATTGGATATGGTACCGTGGTGGGATATTTGCGAAATCGGAGCAAAGTTGGGAAATGTGGTG
GGAGGAAGAACAAGATCATTTGCGCACAACTGGACTTTGGGGAAAATTATTGGAGATAGTTCTTGATCTA
CGGTTCTTTTTTTTCCAGTATGGCATCGTCTTTCAGCTTAATATTGCCAATAATAGCAAAAGCATTATTG
TTTACTTTCTCTCATGGATCTATGTGCTAGTTGCTTTTGGAGTTTTCTTGATAATGGGCTATGCTCGAGA
TAAATATGCAGCAAAGGAGCATGTCTATTATAGGACCGTCCAAGCTTTGATCATCACTCTTGCTATTTTG
GTGATTGTTATATTTCTCAAGTTCACCGAGTTTGGTATCGTTGATATTTTTACTAGTCTTTTAGCATTAA
TTCCAACTGGTTGGGGCATGATTTTGATAGCTCAAGTGATGAAACCGCTCATTAAGACCACTGTGGTCTG
GGATACTGTTGTTGCTGTGGCTCGGCTGTATGATATGATGTTTGGAGTCATTGTTATGGCTCCCGTTGCA
CTATTGTCGTGGTTACCTGGCTTCCAGGCAATGCAGACAAGGATTCTCTTCAATGAAGCATTCAGCAGGG
GTCTTCAAATATCTCGCATCATTACCGGAAAGAAATCTAATTTTGATTTGTAA
>XM_020844696.2_*Dendrobium_catenatum*
ATGAGCCTCCGGCAGCGCACGACTCGCCCGGCTGGGGCCGGCGGGGCTGGGGGCAGAGGATATGGTGCTA
GGGGATACGGCGCAGCGGAGGGGGAGGAAGCCTATAATATAATACCCATCCACAATCTGCTCGCGGAGCA
TCCAGCGCTTCGGTTCCCTGAGGTGCGTGCCGCCATGGCCGCCCTCCGCACCGTTGGTGACCTTCGCAAG
CCACAGTTCATCCGGTGGCAGGAAGGATACGACCTCCTTGACTGGCTTGGCTTCTTTTTCGGTTTCCAGC
CCGACAACGTCCGCAACCAGCGCGAGCACCTAGTTCTCCTCCTCGCTAACGCGCAGATGCGCCTCCAGCC
GCCACCGGACAACATCGATGCCCTTGAACCCAACGTCGCTCGACGTCTCCGCCGCAAGCTTCTCAAAAAC
TATACCGCGTGGTGCTCCTACCTTGGTCGGAAACCTAACGTCTGGATCTCAGACAGACGCCGCTCCGCCA
CAGCTGCTTCCGACTACTCTCGCCGCGACCTCCTCTACACTGCCCTTTACCTCCTCATCTGGGGCGAAGC
TGCTAACCTCCGCTTCGTCCCTGAATGCATCTCCTACATCTTCCACCACATGGCCATGGACCTCAACCGC
ATCCTTGAGGATTACATCGATGAAGCCACTGGTCGCCCCTCCACCCCGAAAATTACCGGTGAGGAGGCCT
TCCTCAACCATGTAGTCAAGCCAATCTACGCCACCATTAAAGCCGAGGTTGACTCCAGCCGCAATGGCAC
CGCACCCCACTCCGCCTGGCGCAACTATGATGACATCAACGAGTACTTCTGGTCTCGCCATTGCTTTGAG
CGCCTTAAATGGCCTCTCGAACTCTCCAAGAACTTCTTTGCCATTCCTCCTGATCGCAACCGAGTTCGAA
AGACTGGCTTCGTCGAGCAGCGCTCATTCTGGAACCTCTTCCGCAGTTTCGACCGTCTCTGGGTAATGCT
AATCCTCTATCTCCAGGCGGCCATCATCGTTGCCTGGGAAGGCAAAACCTATCCTTGGCAAAATCTGCAG
AGCCGTGACGTCCAGGTCAGGACTCTAACTATATTTATCACTTGGGCTGTTCTTCGCTTCTTCCAATCCA
TTTTGGATGCCGGGACGCAATACAGCCTTGTGCGCAGGGAGACTATGTGGCTAGGAGTGAGGATGGTGCT
CAAGAGCGTTGTGGCTGCTGCTTGGACTGTGGTGTTTGGCATTCTGTACGCCAGGGCTTGGGACCAGAAA
AACAGAGACCGCAGATGGTCTGCGGCAGCCAACCAGAGGCTGGTGAACTACTTGGAGGCCGCCGGAGTGT
TTGTGCTGCCGGAGGTGATGGCCCTTCTTCTCTTCATACTGCCCTGGATAAGGAACTTCCTTGAGAAGAC
AAACTGGAAGATCTTTTATGTGCTTACTTGGTGGTTTCAAAGCCGCACTTTTGTTGGTCGTGGCCTGAGA
GAAGGGCTTGTTGACAACGTGAAATACGCCCTGTTTTGGGTTCTTCTTCTAGCCGTGAAGTTCACCTTCA
GCTACTTCCTTCAGATCAAGCCAATGGTCTCTCCAAGCAAATCCATTTACAAGCTTCATGTTGTAGACTA
TCATTGGCATGAGCTCTTTAGTCGGACCAACAGGTTTGCCGTTTTTCTCTTGTGGCTCCCGGTTGTACTG
GTATATCTTATGGATATTTCAATCTGGTATTCAATTTTTTCATCCCTTGTTGGAGCTCTGGTGGGGCTGT
TCTCACACATTGGCGAAATCCGGAATGTTCAGCAGCTAAGGCTGAGGTTTCAGTTCTTTGCAAGTGCGAT
GCAGTTCAATCTGATGCCTGAGGAGCAACTCTTTCATGAGAGGGGTACACTAAGGAGCAAGTTCAAAGAT
GCGGTTTTACGCCTCAAGCTCCGATATGGGTTGGGGAGGCCTTACAAGAAGATTGAATCAAACCAGGTTG
AAGCAACCAGGTTTGCGTTAATATGGAATGAGATTATGGCTACGTTCAGGGAGGAGGATATCATCAGTGA

CAGAGAGTTGGAACTTCTTGAGCTGCCCGCAAATTCTTGGAACATCAGGGTGATTCGGTGGCCTTGCCTG
TTACTCTGCAATGAGCTTCTTCTGGCTCTTAACCAGGCGAAGGAACTGGTAGCGTCGGACCGTGGGCATT
GGAGAAAGATATGCAAAAATGAGTACCGGCGATGTGCGGTTGTGGAGGCTTATGATAGCGTCAAACACCT
GCTGCTTGAGATCATCAAAGAGGAAACTGAGGAGCACAAAATCATTTCACACCTGTTCTTTGGGTTTGAT
GATGCCATCGGCGTGGAGAAATTGACTGCAGAGTATAAATTGGCAGTGTTGCCAGTTATTCACACCAAGC
TGATCACCCTCCTCGATCAACTGCTCAAACCAAATAAGGATCTAAACAAAATAGTAAATACATTACAGAC
TTTATATGATGTTGCCATCCGTGATTTTCCTACTACTAAGAGGAACATGGAGCAGCTAAAGCAAGCAGGA
CTGGTGCCCACGAGACCAAATGCATCTGGTCTTCTTTTTGAGAATGCTATTGAATTGCCTGTCGCAGAAG
ACACAAATTTCTACAGGCAGGTGAGACGGTTGCACACTATTCTTACTTCCAGGGATTCCATGAATGATGT
GCCCAAGAACCTAGAGGCTCGGCGCCGAATTTCCTTCTTCAGCAACTCCTTGTTCATGAACATGCCACGG
GCTCCCCAAGTAGAGAAAATGTTTGCCTTTAGTGTTCTTACCCCATATTATAATGAGGATGTCTTGTTCA
GTAAGGAGCAGCTTCGCACAGAGAATGAAGATGGCATCTCGATCTTATTTTATCTGCAAAAGATTTATGA
TGATGAATGGGCTAACTTTTTAGAACGAATGAGAGGAGAAGGAATGGTGTATGAGTCTGACATATGGGGT
GAGAAGTTGAGGGATCTTCGTCTTTGGGCCTCTAACAGGGGCCAGACTCTCAGTCGCACAGTCAGGGGAA
TGATGTACTACTACAAGGCTCTCAAGATGCTTACTTTTCTTGATTCTGCTTCTGAGATTGACATTAGGGA
AGGATCACGAGAACTTTCTTTACTTGGTTCTTCTCGGGAGACTGTTGCAGATGGATTAGGGGATTCTTTT
AGGTCTGTGTCAGCACGGAATCTCAGCAGAGAAAGCAGTGGTGTGAGTCTATTATTCAAAGGCCATGAGT
ATGGAACAGCCCTCATGAAGTATACTTACGTTGTTGCCTGTCAGATATATGGACAGCAGAAGGCAAAGAA
AGACCCTCATGCTGAAGAAATTTTGTATCTTATGAAGAATAATGAAGCACTCAGGGTGGCCTATGTTGAT
GAAGTTTACACAGGGAGGGAGGAAGTGGAATATTATTCTGTTCTTGTTAAATATGACCAGCAGTTGCAGA
AAGAGGTAGAGATCTACCGGGTCAAGTTGCCTGGACCATTGAAGCTTGGTGAGGGCAAGCCAGAGAACCA
GAACCATGCCCTCATTTTCACAAGAGGTGATGCAGTGCAGACTATAGATATGAACCAAGACAATTACTTT
GAAGAGGCTCTCAAGATGAGAAATTTGTTGGAAGAATACACACATAACTATGGTTCAAGAAAGCCAACCA
TTTTAGGTGTCCGGGAGCATGTTTTTACTGGTTCTGTCTCTTCACTTGCTTGGTTCATGTCTGCACAGGA
AACTAGCTTCGTCACCCTTGGACAGCGGGTCCTAGCAAATCCTCTGAAAGTGAGGATGCACTATGGACAC
CCAGATGTTTTTGATCGGCTCTGGTTTTTGGGTCGTGGCGGTATTAGCAAGGCTTCCAGAGTCATCAACA
TCAGTGAGGATATATTTGCAGGCTTTAACTGCACATTGCGAGGTGGTAATGTAACTCATCATGAGTATAT
CCAGGTTGGCAAGGGACGCGATGTTGGCCTGAATCAGATTTCGATGTTCGAGGCTAAGGTCGCTAGTGGC
AATGGAGAGCAGGCTTTAAGCAGGGATGTCTATAGACTTGGCCATAGGTTGGACTTTTTTAGGATGCTTT
CCTTTTATTACACGACCGTTGGATTTTACTTCAATACCATGTTGGTCGTCCTAACCGTATATTCTTTTGT
ATGGGGACGCCTTTACCTGGCTCTTAGTGGGCTTGAGGCTGCCATAAAGAACAGCGCCGACTCGACAAGC
AATACTGCACTTGGTACAGTTCTGAATCAGCAGTTCATTATTCAGCTGGGTATTTTCACAGCCTTGCCAA
TGATTGTAGAAAACTCACTTGAGCATGGATTCCTTGGGGCGGTTTGGGATTTTCTGACCATGCAGATTCA
GCTTGCATCTGTTTTTTACACCTTCTCCATGGGAACTAAAACTCATTATTTTGGGCGGACAATCCTTCAC
GGAGGTGCAAAATATAGAGCAACTGGGCGCGGCTTTGTTGTGCAGCACAAGAGTTTTTGTGAGAACTACA
GGCTTTATGCACGGAGCCATTTTATTAAGGCTATAGAGCTTGGTGTTGTGCTAACATTGTATGCATCATA
TAGTGCCATTGCGAAGAACACTATTGTTTACATTGTGATGACAATCTCTTCATGGTTTCTGGTGGTGTCA
TGGATAATGGCTCCTTTTGCTTTCAACCCTTCTGGTTTTGACTGGTTGAAGACTGTTGATGACTTCGATG
ATTTTGTGAATTGGATATGGTACCGTGGTGGGATATTTGCGAAATCGGAGCAAAGTTGGGAAATGTGGTG
GGAGGAAGAACAAGATCATTTGCGCACAACTGGACTTTGGGGAAAATTATTGGAGATAGTTCTTGATCTA
CGGTTCTTTTTTTTCCAGTATGGCATCGTCTTTCAGCTTAATATTGCCAATAATAGCAAAAGCATTATTG
TTTACTTTCTCTCATGGATCTATGTGCTAGTTGCTTTTGGAGTTTTCTTGATAATGGGCTATGCTCGAGA
TAAATATGCAGCAAAGGAGCATGTCTATTATAGGACCGTCCAAGCTTTGATCATCACTCTTGCTATTTTG
GTGATTGTTATATTTCTCAAGTTCACCGAGTTTGGTATCGTTGATATTTTTACTAGTCTTTTAGCATTAA
TTCCAACTGGTTGGGGCATGATTTTGATAGCTCAAGTGATGAAACCGCTCATTAAGACCACTGTGGTCTG
GGATACTGTTGTTGCTGTGGCTCGGCTGTATGATATGATGTTTGGAGTCATTGTTATGGCTCCCGTTGCA
CTATTGTCGTGGTTACCTGGCTTCCAGGCAATGCAGACAAGGATTCTCTTCAATGAAGCATTCAGCAGGG
GTCTTCAAATATCTCGCATCATTACCGGAAAGAAATCTAATTTTGATTTGTAA
>XM_020844693.2_*Dendrobium_catenatum*
ATGAGCCTCCGGCAGCGCACGACTCGCCCGGCTGGGGCCGGCGGGGCTGGGGGCAGAGGATATGGTGCTA
GGGGATACGGCGCAGCGGAGGGGGAGGAAGCCTATAATATAATACCCATCCACAATCTGCTCGCGGAGCA
TCCAGCGCTTCGGTTCCCTGAGGTGCGTGCCGCCATGGCCGCCCTCCGCACCGTTGGTGACCTTCGCAAG
CCACAGTTCATCCGGTGGCAGGAAGGATACGACCTCCTTGACTGGCTTGGCTTCTTTTTCGGTTTCCAGC
CCGACAACGTCCGCAACCAGCGCGAGCACCTAGTTCTCCTCCTCGCTAACGCGCAGATGCGCCTCCAGCC

GCCACCGGACAACATCGATGCCCTTGAACCCAACGTCGCTCGACGTCTCCGCCGCAAGCTTCTCAAAAAC
TATACCGCGTGGTGCTCCTACCTTGGTCGGAAACCTAACGTCTGGATCTCAGACAGACGCCGCTCCGCCA
CAGCTGCTTCCGACTACTCTCGCCGCGACCTCCTCTACACTGCCCTTTACCTCCTCATCTGGGGCGAAGC
TGCTAACCTCCGCTTCGTCCCTGAATGCATCTCCTACATCTTCCACCACATGGCCATGGACCTCAACCGC
ATCCTTGAGGATTACATCGATGAAGCCACTGGTCGCCCCTCCACCCCGAAAATTACCGGTGAGGAGGCCT
TCCTCAACCATGTAGTCAAGCCAATCTACGCCACCATTAAAGCCGAGGTTGACTCCAGCCGCAATGGCAC
CGCACCCCACTCCGCCTGGCGCAACTATGATGACATCAACGAGTACTTCTGGTCTCGCCATTGCTTTGAG
CGCCTTAAATGGCCTCTCGAACTCTCCAAGAACTTCTTTGCCATTCCTCCTGATCGCAACCGAGTTCGAA
AGACTGGCTTCGTCGAGCAGCGCTCATTCTGGAACCTCTTCCGCAGTTTCGACCGTCTCTGGGTAATGCT
AATCCTCTATCTCCAGGCGGCCATCATCGTTGCCTGGGAAGGCAAAACCTATCCTTGGCAAAATCTGCAG
AGCCGTGACGTCCAGGTCAGGACTCTAACTATATTTATCACTTGGGCTGTTCTTCGCTTCTTCCAATCCA
TTTTGGATGCCGGGACGCAATACAGCCTTGTGCGCAGGGAGACTATGTGGCTAGGAGTGAGGATGGTGCT
CAAGAGCGTTGTGGCTGCTGCTTGGACTGTGGTGTTTGGCATTCTGTACGCCAGGGCTTGGGACCAGAAA
AACAGAGACCGCAGATGGTCTGCGGCAGCCAACCAGAGGCTGGTGAACTACTTGGAGGCCGCCGGAGTGT
TTGTGCTGCCGGAGGTGATGGCCCTTCTTCTCTTCATACTGCCCTGGATAAGGAACTTCCTTGAGAAGAC
AAACTGGAAGATCTTTTATGTGCTTACTTGGTGGTTTCAAAGCCGCACTTTTGTTGGTCGTGGCCTGAGA
GAAGGGCTTGTTGACAACGTGAAATACGCCCTGTTTTGGGTTCTTCTTCTAGCCGTGAAGTTCACCTTCA
GCTACTTCCTTCAGATCAAGCCAATGGTCTCTCCAAGCAAATCCATTTACAAGCTTCATGTTGTAGACTA
TCATTGGCATGAGCTCTTTAGTCGGACCAACAGGTTTGCCGTTTTTCTCTTGTGGCTCCCGGTTGTACTG
GTATATCTTATGGATATTTCAATCTGGTATTCAATTTTTTCATCCCTTGTTGGAGCTCTGGTGGGGCTGT
TCTCACACATTGGCGAAATCCGGAATGTTCAGCAGCTAAGGCTGAGGTTTCAGTTCTTTGCAAGTGCGAT
GCAGTTCAATCTGATGCCTGAGGAGCAACTCTTTCATGAGAGGGGTACACTAAGGAGCAAGTTCAAAGAT
GCGGTTTTACGCCTCAAGCTCCGATATGGGTTGGGGAGGCCTTACAAGAAGATTGAATCAAACCAGGTTG
AAGCAACCAGGTTTGCGTTAATATGGAATGAGATTATGGCTACGTTCAGGGAGGAGGATATCATCAGTGA
CAGAGAGTTGGAACTTCTTGAGCTGCCCGCAAATTCTTGGAACATCAGGGTGATTCGGTGGCCTTGCCTG
TTACTCTGCAATGAGCTTCTTCTGGCTCTTAACCAGGCGAAGGAACTGGTAGCGTCGGACCGTGGGCATT
GGAGAAAGATATGCAAAAATGAGTACCGGCGATGTGCGGTTGTGGAGGCTTATGATAGCGTCAAACACCT
GCTGCTTGAGATCATCAAAGAGGAAACTGAGGAGCACAAAATCATTTCACACCTGTTCTTTGGGTTTGAT
GATGCCATCGGCGTGGAGAAATTGACTGCAGAGTATAAATTGGCAGTGTTGCCAGTTATTCACACCAAGC
TGATCACCCTCCTCGATCAACTGCTCAAACCAAATAAGGATCTAAACAAAATAGTAAATACATTACAGAC
TTTATATGATGTTGCCATCCGTGATTTTCCTACTACTAAGAGGAACATGGAGCAGCTAAAGCAAGCAGGA
CTGGTGCCCACGAGACCAAATGCATCTGGTCTTCTTTTTGAGAATGCTATTGAATTGCCTGTCGCAGAAG
ACACAAATTTCTACAGGCAGGTGAGACGGTTGCACACTATTCTTACTTCCAGGGATTCCATGAATGATGT
GCCCAAGAACCTAGAGGCTCGGCGCCGAATTTCCTTCTTCAGCAACTCCTTGTTCATGAACATGCCACGG
GCTCCCCAAGTAGAGAAAATGTTTGCCTTTAGTGTTCTTACCCCATATTATAATGAGGATGTCTTGTTCA
GTAAGGAGCAGCTTCGCACAGAGAATGAAGATGGCATCTCGATCTTATTTTATCTGCAAAAGATTTATGA
TGATGAATGGGCTAACTTTTTAGAACGAATGAGAGGAGAAGGAATGGTGTATGAGTCTGACATATGGGGT
GAGAAGTTGAGGGATCTTCGTCTTTGGGCCTCTAACAGGGGCCAGACTCTCAGTCGCACAGTCAGGGGAA
TGATGTACTACTACAAGGCTCTCAAGATGCTTACTTTTCTTGATTCTGCTTCTGAGATTGACATTAGGGA
AGGATCACGAGAACTTTCTTTACTTGGTTCTTCTCGGGAGACTGTTGCAGATGGATTAGGGGATTCTTTT
AGGTCTGTGTCAGCACGGAATCTCAGCAGAGAAAGCAGTGGTGTGAGTCTATTATTCAAAGGCCATGAGT
ATGGAACAGCCCTCATGAAGTATACTTACGTTGTTGCCTGTCAGATATATGGACAGCAGAAGGCAAAGAA
AGACCCTCATGCTGAAGAAATTTTGTATCTTATGAAGAATAATGAAGCACTCAGGGTGGCCTATGTTGAT
GAAGTTTACACAGGGAGGGAGGAAGTGGAATATTATTCTGTTCTTGTTAAATATGACCAGCAGTTGCAGA
AAGAGGTAGAGATCTACCGGGTCAAGTTGCCTGGACCATTGAAGCTTGGTGAGGGCAAGCCAGAGAACCA
GAACCATGCCCTCATTTTCACAAGAGGTGATGCAGTGCAGACTATAGATATGAACCAAGACAATTACTTT
GAAGAGGCTCTCAAGATGAGAAATTTGTTGGAAGAATACACACATAACTATGGTTCAAGAAAGCCAACCA
TTTTAGGTGTCCGGGAGCATGTTTTTACTGGTTCTGTCTCTTCACTTGCTTGGTTCATGTCTGCACAGGA
AACTAGCTTCGTCACCCTTGGACAGCGGGTCCTAGCAAATCCTCTGAAAGTGAGGATGCACTATGGACAC
CCAGATGTTTTTGATCGGCTCTGGTTTTTGGGTCGTGGCGGTATTAGCAAGGCTTCCAGAGTCATCAACA
TCAGTGAGGATATATTTGCAGGCTTTAACTGCACATTGCGAGGTGGTAATGTAACTCATCATGAGTATAT
CCAGGTTGGCAAGGGACGCGATGTTGGCCTGAATCAGATTTCGATGTTCGAGGCTAAGGTCGCTAGTGGC
AATGGAGAGCAGGCTTTAAGCAGGGATGTCTATAGACTTGGCCATAGGTTGGACTTTTTTAGGATGCTTT
CCTTTTATTACACGACCGTTGGATTTTACTTCAATACCATGTTGGTCGTCCTAACCGTATATTCTTTTGT

ATGGGGACGCCTTTACCTGGCTCTTAGTGGGCTTGAGGCTGCCATAAAGAACAGCGCCGACTCGACAAGC
AATACTGCACTTGGTACAGTTCTGAATCAGCAGTTCATTATTCAGCTGGGTATTTTCACAGCCTTGCCAA
TGATTGTAGAAAACTCACTTGAGCATGGATTCCTTGGGGCGGTTTGGGATTTTCTGACCATGCAGATTCA
GCTTGCATCTGTTTTTTACACCTTCTCCATGGGAACTAAAACTCATTATTTGGGCGGACAATCCTTCAC
GGAGGTGCAAAATATAGAGCAACTGGGCGCGGCTTTGTTGTGCAGCACAAGAGTTTTTGTGAGAACTACA
GGCTTTATGCACGGAGCCATTTTATTAAGGCTATAGAGCTTGGTGTTGTGCTAACATTGTATGCATCATA
TAGTGCCATTGCGAAGAACACTATTGTTTACATTGTGATGACAATCTCTTCATGGTTTCTGGTGGTGTCA
TGGATAATGGCTCCTTTTGCTTTCAACCCTTCTGGTTTTGACTGGTTGAAGACTGTTGATGACTTCGATG
ATTTTGTGAATTGGATATGGTACCGTGGTGGGATATTTGCGAAATCGGAGCAAAGTTGGGAAATGTGGTG
GGAGGAAGAACAAGATCATTTGCGCACAACTGGACTTTGGGGAAAATTATTGGAGATAGTTCTTGATCTA
CGGTTCTTTTTTTTCCAGTATGGCATCGTCTTTCAGCTTAATATTGCCAATAATAGCAAAAGCATTATTG
TTTACTTTCTCTCATGGATCTATGTGCTAGTTGCTTTTGGAGTTTTCTTGATAATGGGCTATGCTCGAGA
TAAATATGCAGCAAAGGAGCATGTCTATTATAGGACCGTCCAAGCTTTGATCATCACTCTTGCTATTTTG
GTGATTGTTATATTTCTCAAGTTCACCGAGTTTGGTATCGTTGATATTTTTACTAGTCTTTTAGCATTAA
TTCCAACTGGTTGGGGCATGATTTTGATAGCTCAAGTGATGAAACCGCTCATTAAGACCACTGTGGTCTG
GGATACTGTTGTTGCTGTGGCTCGGCTGTATGATATGATGTTTGGAGTCATTGTTATGGCTCCCGTTGCA
CTATTGTCGTGGTTACCTGGCTTCCAGGCAATGCAGACAAGGATTCTCTTCAATGAAGCATTCAGCAGGG
GTCTTCAAATATCTCGCATCATTACCGGAAAGAAATCTAATTTTGATTTGTAA
>XM_022710063.1_*Brassica_napus*
ATGAGCCTCCGCCACCGCACCGTCCCCTCTCAACCCGGACGGCCCCCGGCGGCGGGCGCAATCGACGACG
AGCCCTACAACATCATCCCCGTCAACAACCTCCTCGCCGACCACCCCTCCCTCCGCTACCCCGAGGTCCG
CGCCGCCGCCGCCGCCCTCAAAACCGGCGGCGACCTCCGCCGCCCCACCTACGTCCAATGGCGCCCCCAC
TACGACCTCCTCGACTGGCTCGCCCTCTTCTTCGGCTTCCAGAAGGACAACGTCCGCAACCAGCGCGAGC
ACCTCGTCCTCCACCTCGCCAACGCCCAGATGCGCCTCACGCCGCCGCCGGATAACATCGATTCCCTCGA
TCCCGCCGTCGTCCGCCGTTTCCGCCGCAAGCTCCTCGGTAACTACTCGAGCTGGTGCTCGTACCTCGGG
AGGAAGTCGAACATCTGGATCTCGGATCGGAACCCCGATTCGAGGCGGGAGCTTCTCTACGTCGGCCTCT
ACCTCCTCGTGTGGGGGGAGGCGGCGAATCTTAGGTTTATGCCGGAGTGTGTCTGTTACATCTTCCACAA
TATGGCCTCGGAGCTTAACAAGATCCTCGAGGATTGCCTCGACGAGAGCACGGGGCAGCCGTACTCTCCT
AGAATCACGGGGGAGAATAGTTTCCTAAACGGCGTCGTTAAACCTATTTACGAGACGATCAAAGCTGAGA
TTAACGAGAGCAAGAACGGGACGGAGCCGCATTGTAAGTGGAGGAACTATGATGATATTAATGAATACTT
TTGGACGGATAGGTGTTTTAGTAAATTGAAATGGCCGATTGATTTGGGGAGCAGTTTCTTCAAGAGTAGT
AGAGGGAGAGGCGTTGGGAAGACAGGTTTTGTGGAGAGGAGGACGTTCTTTTACCTCTACAGGAGCTTTG
ATAGGCTTTGGGTGATGCTTGCTTTGTTTCTTCAAGCTGCTATTATAGTCGCTTGGGAGGAGAAGCCGGG
TGGAGGGTCGGTGAGGAGTCAGCTCTGGAATGCGTTGAAGTCGAGGGATGTTCGGGTGAGGCTTTTGACT
GTGTTCTTGACGTGGAGTGGGATGAGATTACTGCAGGCTGTGCTGGACGCTGCCTCGCAACGGCCGCTTA
TTTCTAGAGAGACCAAGCGGCTGTTTTTCAGAATGTTGATGAAGGTTGTAGCTGCTACGGTTTGGATAAT
TGCTTTTATTGTTCTCTACACGAACATCTGGAAGCAGAGGAAGCAAGACAGGCAGTGGTCCAGAGCCGCG
AATGACAAGATCTATCAGTTCCTTTACGCTGTGGTGGCTTTCTTGGTCCCTGAGATCCTGGCTTTGGCTC
TGTTTATAGTCCCGTGGATAAGGAACTTTCTGGAAGAGACCAATTGGAAGATATTCTTTGCTTTGACTTG
GTGGTTCCAGGGTAAAAGCTTTGTGGGTCGAGGTTTGAGAGAGGGGTTGGTGGACAACATCAAGTACTCG
ACTTTCTGGATCTTTGTCCTAGCAACGAAGTTCACGTTCAGCTACTTCCTGCAGGTTAAGCCAATGATTA
AACCCTCGAAGCTGCTATGGAATTTGAAGGAGGTGGATTATGAGTGGCATCAGTTCTTTGGCGAGAGCAA
TAGGTTTTCTGTCTTGTTATTGTGGCTGCCAGTGGTGTTGATATACCTGATGGATATCCAAATTTGGTAC
GCGATCTATTCTTCGATTGTTGGTGCTGTTGTTGGGCTGTTTGATCATCTGGGGGAGATCAGGGACATGG
GACAGCTTAGGCTGAGGTTTCAGTTCTTTGCTAGCGCTATTCAGTTCAACCTAATGCCTGAGGAACAACT
CCTGAATGCTAGAGGATTTGGTAACAAGCTTAAGGACGCCATTCATAGATTGAAGCTGAGGTATGGACTT
GGGCGGCCATTTAAGAAACTCGAGTCTAATCAGGTTGAGGCTAACAAGTTTGCGCTGATCTGGAATGAGA
TAATCTTAGCTTTCAGAGAAGAGGATATAGTCTCTGATCGAGAAGTAGAGCTACTGGAGCTGCCAAAAAA
TTCCTGGAATGTGACAGTTATCCGCTGGCCGTGTTTCCTGTTGTGCAACGAGCTTTTGCTTGCACTGAGC
CAGGCGAAAGAGCTGGTTGACGCACCTGATAAATGGCTGTGGCACAAGATATGCAAGAACGAGTACAGGC
GGTGTGCTGTGGTTGAGGCATATGAAAGCATCAAACATCTGTTGCTCTCAATCATCAAAATTGACACCGA
AGAACATAAAATTATTACAATTTTCTTTCAGATGATTGAGGTGTCTATTCAGGGTGAGCAGTTCACCAAG
ACCTTCAAAGTGGACCTATTGCCAAAGATTTATGAGACGCTACAGAAGTTGGTTGGGCTGTTGAATGATG
AGAAAGTGGATGTTGGGCGAGTGGTGAATGGTCTGCAGTCTATTTATGAGATTGCAACACGACAGTTCTT

CATAGAAAAGAAGACGACTGAACAGCTATCTACCGAGGGGTTAACTCCTCATGATCCAGCCTCAAAGTTA
CTGTTTCAGAATGCTGTTAGGCTTCCCGATGCAAGCAATGAAGACTTCTTCCGGCAGGTTAGGCGGTTAC
ACACAATTCTCACTTCTAGGGACTCTATGCACAGCGTCCCTGTGAATCTAGAGGCGAGACGGCGGATTGC
CTTCTTCAGCAATTCGCTCTTCATGAACTTGCCTCATGCACCTCAGGTGGAGAAAATGTTGGCGTTCAGT
GTTATGACTCCATACTACAGCGAGGAAGTTGTATACAGTAAAGAACAGCTCCGAAATGAGACTGAGGATG
GGATTTCAACCTTGTATTACCTGCAGACGATTTATGCCGACGAATGGAAAAATTTTAAGGAACGGATGCG
TAGGGAAGGTATAAAGACAGATGTTGAGTTGTGGACAACCAAGCTGAGAGAGCTCAGGCTTTGGGCTTCC
TACAGAGGTCAGACTTTGGCACGTACAGTTCGAGGAATGATGTACTATTACAGGGCTCTTAAGATGCTTG
CTTTTCTCGACTCTGCGTCTGAAATGGACATTCGGGAGGATGCTCAGGAGCTTGGTTCAATGAGGAGTTC
GCAGGGAAATCGATTGGATGGTGTTGACGATGTAAATGACCGATCTTCTCTAAGCAGAGCAACTAGCTCT
GTGAGCATGCTGTATAAAGGCCATGAGCATGGGACTGCATTGATGAAATTCACATATGTCGTGGCGTGCC
AAATCTATGGGTCTCAAAAAGCGAAGAAAGAGCCTCAGGCAGAGGAAATTCTGTATCTTATGAAGCAAAA
CGAAGCCCTTCGTATTGCATATGTGGATGAGGTACATGCGGGCAGGGGAGAGACTGAGTATTACTCCGTT
CTGGTGAAATACGATCACACGTTGGAGAGGGAAGTGGAGATATTCCGTGTGAAGCTACCTGGTCCGGTGA
AGCTGGGTGAGGGAAAGCCAGAGAACCAGAATCATGCAATGATCTTTACCCGTGGTGATGCTGTTCAGAC
CATAGATATGAACCAGGATAATTATTTTGAGGAGGCTCTCAAGATGAGAAATTTGCTCCAGGAGTTTAGG
CATTATCATGGGATCAGAAAACCAACTATTCTTGGTGTCAGAGAGCACATCTTCACGGGTTCTGTCTCGT
CTCTGGCTTGGTTCATGTCTGCTCAGGAGACAAGTTTCGTCACTCTGGGTCAGCGTGTTCTAGCCAACCC
GCTGAAGGTCAGAATGCATTATGGTCACCCTGATGTATTTGACAGATTCTGGTTCTTGAGTCGAGGTGGC
ATCAGCAAAGCTTCTAGAGTTATAAATATCAGTGAGGACATCTTCGCCGGGTTTAATTGCACATTGCGGG
GCGGTAACGTCACCCACCACGAGTATATTCAGGTTGGGAAGGGTCGAGATGTTGGATTGAATCAAATATC
AATGTTTGAGGCTAAGGTAGCCAGTGGGAATGGAGAGCAGGTTCTTAGCCGAGATGTGTACAGGTTGGGT
CATAGGCTCGATTTCTTCAGAATGTTATCATTTTTCTACACAACGGTGGGGTTTTTCTTCAACACGATGA
TGGTCATTCTCACTGTCTACGCTTTCCTCTGGGGCCGGGTTTATCTTGCTCTGAGCGGTGTTGAGAAGTC
CGCTCTAGCAGACAGCACAGACACCAACGCAGCGCTTGCTGTGATATTGAACCAGCAGTTCATCATTCAG
CTTGGTCTCTTCACAGCTCTGCCAATGATTGTGGAATGGTCTCTCGAGGAGGGTTTCCTTCTCGCGATAT
GGAACTTCATTCGGATGCAGATTCAGCTTTCTTCTGTCTTCTACACATTCTCAATGGGGACCAGAGCTCA
CTATTTTGGCCGAACCATTCTCCACGGTGGAGCAAAGTACAGAGCCACTGGACGTGGATTTGTTGTCGAG
CACAAGAGTTTCACTGAAAACTACCGTCTATACGCGCGCAGTCACTTTGTGAAGGCCATCGAGCTTGGGC
TGATCCTCATAGTCTACGCTACGCACAGTCCCATCGCCAAAGACTCATTGATCTATATAGCCATGACTCT
CACCAGCTGGTTCCTCGTGATTTCATGGATACTAGCCCCTTTTGTGTTCAACCCGTCAGGTTTCGACTGG
CTTAAGACGGTCTACGACTTCGAAGGCTTCATGAACTGGATCTGGTATCAAGGCAGAATCTCAACGAAGT
CCGAACAGAGCTGGGAGATATGGTGGTATGAGGAACAGGACCACCTGAGAACCACCGGTCTACCAGGAAG
AATCATGGAGATAATCTTGGACCTTCGGTTTTTCTTCTTCCAGTACGGGATTGTATACCAGCTCAAAATC
GCAAACGGATCAACCAGCATTCTCGTCTACTTACTCTCATGGATATACATCTTCGCAGTGTTTGTGTTCT
TCCTGGTAATCCAATACGCCCGTGACAAGTACTCAGCGAGAAACCACATACGGTACAGGCTCGTTCAATT
CCTCCTGATCGTGTTTGGTACACTGGTGATTGTTGCTCTCCTGGAGTTCACGCATTTCAGCTTCGTGGAT
ATCTTCACGAGTCTTCTTGCGTTCGTCCCAACCGGCTGGGGAATCTTGCTGATCGCACAGGCTTTGAGGC
CTGCGCTGCAGAAGATCGGGCTTATCTGGAACGCGGTTATCTCCCTTGCTCGGTTATATGACATACTGTT
CGGGATAGTCATCATGGTTCCCGTAGCGTTCATGTCGTGGATGCCTGGGTTTCAGTCGATGCAAACGAGG
ATCTTATTCAATGAAGCTTTTAGCAGAGGGCTTCGTATCATGCAGATTGTCACTGGGAAGAAATCTAAAG
GCGATGTCGAAGTTGAAAAAAGAAGGTCTTAA
>XM_013875984.2_*Brassica_napus*
ATGAGCCTCCGCCACCGCACCGTCCCCTCTCAACCCGGACGGCCCCCGGCGGCGGGCGCAATCGACGACG
AGCCCTACAACATCATCCCCGTCAACAACCTCCTCGCCGACCACCCCTCCCTCCGCTACCCCGAGGTCCG
CGCCGCCGCCGCCGCCCTCAAAACCGGCGGCGACCTCCGCCGCCCCACCTACGTCCAATGGCGCCCCCAC
TACGACCTCCTCGACTGGCTCGCCCTCTTCTTCGGCTTCCAGAAGGACAACGTCCGCAACCAGCGCGAGC
ACCTCGTCCTCCACCTCGCCAACGCCCAGATGCGCCTCACGCCGCCGCCGGATAACATCGATTCCCTCGA
TCCCGCCGTCGTCCGCCGTTTCCGCCGCAAGCTCCTCGGTAACTACTCGAGCTGGTGCTCGTACCTCGGG
AGGAAGTCGAACATCTGGATCTCGGATCGGAACCCCGATTCGAGGCGGGAGCTTCTCTACGTCGGCCTCT
ACCTCCTCGTGTGGGGGGAGGCGGCGAATCTTAGGTTTATGCCGGAGTGTGTCTGTTACATCTTCCACAA
TATGGCCTCGGAGCTTAACAAGATCCTCGAGGATTGCCTCGACGAGAGCACGGGGCAGCCGTACTCTCCT
AGAATCACGGGGGAGAATAGTTTCCTAAACGGCGTCGTTAAACCTATTTACGAGACGATCAAAGCTGAGA
TTAACGAGAGCAAGAACGGGACGGAGCCGCATTGTAAGTGGAGGAACTATGATGATATTAATGAATACTT

TTGGACGGATAGGTGTTTTAGTAAATTGAAATGGCCGATTGATTTGGGGAGCAGTTTCTTCAAGAGTAGT
AGAGGGAGAGGCGTTGGGAAGACAGGTTTTGTGGAGAGGAGGACGTTCTTTTACCTCTACAGGAGCTTTG
ATAGGCTTTGGGTGATGCTTGCTTTGTTTCTTCAAGCTGCTATTATAGTCGCTTGGGAGGAGAAGCCGGG
TGGAGGGTCGGTGAGGAGTCAGCTCTGGAATGCGTTGAAGTCGAGGGATGTTCGGGTGAGGCTTTTGACT
GTGTTCTTGACGTGGAGTGGGATGAGATTACTGCAGGCTGTGCTGGACGCTGCCTCGCAACGGCCGCTTA
TTTCTAGAGAGACCAAGCGGCTGTTTTTCAGAATGTTGATGAAGGTTGTAGCTGCTACGGTTTGGATAAT
TGCTTTTATTGTTCTCTACACGAACATCTGGAAGCAGAGGAAGCAAGACAGGCAGTGGTCCAGAGCCGCG
AATGACAAGATCTATCAGTTCCTTTACGCTGTGGTGGCTTTCTTGGTCCCTGAGATCCTGGCTTTGGCTC
TGTTTATAGTCCCGTGGATAAGGAACTTTCTGGAAGAGACCAATTGGAAGATATTCTTTGCTTTGACTTG
GTGGTTCCAGGGTAAAAGCTTTGTGGGTCGAGGTTTGAGAGAGGGGTTGGTGGACAACATCAAGTACTCG
ACTTTCTGGATCTTTGTCCTAGCAACGAAGTTCACGTTCAGCTACTTCCTGCAGGTTAAGCCAATGATTA
AACCCTCGAAGCTGCTATGGAATTTGAAGGAGGTGGATTATGAGTGGCATCAGTTCTTTGGCGAGAGCAA
TAGGTTTTCTGTCTTGTTATTGTGGCTGCCAGTGGTGTTGATATACCTGATGGATATCCAAATTTGGTAC
GCGATCTATTCTTCGATTGTTGGTGCTGTTGTTGGGCTGTTTGATCATCTGGGGGAGATCAGGGACATGG
GACAGCTTAGGCTGAGGTTTCAGTTCTTTGCTAGCGCTATTCAGTTCAACCTAATGCCTGAGGAACAACT
CCTGAATGCTAGAGGATTTGGTAACAAGCTTAAGGACGCCATTCATAGATTGAAGCTGAGGTATGGACTT
GGGCGGCCATTTAAGAAACTCGAGTCTAATCAGGTTGAGGCTAACAAGTTTGCGCTGATCTGGAATGAGA
TAATCTTAGCTTTCAGAGAAGAGGATATAGTCTCTGATCGAGAAGTAGAGCTACTGGAGCTGCCAAAAAA
TTCCTGGAATGTGACAGTTATCCGCTGGCCGTGTTTCCTGTTGTGCAACGAGCTTTTGCTTGCACTGAGC
CAGGCGAAAGAGCTGGTTGACGCACCTGATAAATGGCTGTGGCACAAGATATGCAAGAACGAGTACAGGC
GGTGTGCTGTGGTTGAGGCATATGAAAGCATCAAACATCTGTTGCTCTCAATCATCAAAATTGACACCGA
AGAACATAAAATTATTACAATTTTCTTTCAGATGATTGAGGTGTCTATTCAGGGTGAGCAGTTCACCAAG
ACCTTCAAAGTGGACCTATTGCCAAAGATTTATGAGACGCTACAGAAGTTGGTTGGGCTGTTGAATGATG
AGAAAGTGGATGTTGGGCGAGTGGTGAATGGTCTGCAGTCTATTTATGAGATTGCAACACGACAGTTCTT
CATAGAAAAGAAGACGACTGAACAGCTATCTACCGAGGGGTTAACTCCTCATGATCCAGCCTCAAAGTTA
CTGTTTCAGAATGCTGTTAGGCTTCCCGATGCAAGCAATGAAGACTTCTTCCGGCAGGTTAGGCGGTTAC
ACACAATTCTCACTTCTAGGGACTCTATGCACAGCGTCCCTGTGAATCTAGAGGCGAGACGGCGGATTGC
CTTCTTCAGCAATTCGCTCTTCATGAACTTGCCTCATGCACCTCAGGTGGAGAAAATGTTGGCGTTCAGT
GTTATGACTCCATACTACAGCGAGGAAGTTGTATACAGTAAAGAACAGCTCCGAAATGAGACTGAGGATG
GGATTTCAACCTTGTATTACCTGCAGACGATTTATGCCGACGAATGGAAAAATTTTAAGGAACGGATGCG
TAGGGAAGGTATAAAGACAGATGTTGAGTTGTGGACAACCAAGCTGAGAGAGCTCAGGCTTTGGGCTTCC
TACAGAGGTCAGACTTTGGCACGTACAGTTCGAGGAATGATGTACTATTACAGGGCTCTTAAGATGCTTG
CTTTTCTCGACTCTGCGTCTGAAATGGACATTCGGGAGGATGCTCAGGAGCTTGGTTCAATGAGGAGTTC
GCAGGGAAATCGATTGGATGGTGTTGACGATGTAAATGACCGATCTTCTCTAAGCAGAGCAACTAGCTCT
GTGAGCATGCTGTATAAAGGCCATGAGCATGGGACTGCATTGATGAAATTCACATATGTCGTGGCGTGCC
AAATCTATGGGTCTCAAAAAGCGAAGAAAGAGCCTCAGGCAGAGGAAATTCTGTATCTTATGAAGCAAAA
CGAAGCCCTTCGTATTGCATATGTGGATGAGGTACATGCGGGCAGGGGAGAGACTGAGTATTACTCCGTT
CTGGTGAAATACGATCACACGTTGGAGAGGGAAGTGGAGATATTCCGTGTGAAGCTACCTGGTCCGGTGA
AGCTGGGTGAGGGAAAGCCAGAGAACCAGAATCATGCAATGATCTTTACCCGTGGTGATGCTGTTCAGAC
CATAGATATGAACCAGGATAATTATTTTGAGGAGGCTCTCAAGATGAGAAATTTGCTCCAGGAGTTTAGG
CATTATCATGGGATCAGAAAACCAACTATTCTTGGTGTCAGAGAGCACATCTTCACGGGTTCTGTCTCGT
CTCTGGCTTGGTTCATGTCTGCTCAGGAGACAAGTTTCGTCACTCTGGGTCAGCGTGTTCTAGCCAACCC
GCTGAAGGTCAGAATGCATTATGGTCACCCTGATGTATTTGACAGATTCTGGTTCTTGAGTCGAGGTGGC
ATCAGCAAAGCTTCTAGAGTTATAAATATCAGTGAGGACATCTTCGCCGGGTTTAATTGCACATTGCGGG
GCGGTAACGTCACCCACCACGAGTATATTCAGGTTGGGAAGGGTCGAGATGTTGGATTGAATCAAATATC
AATGTTTGAGGCTAAGGTAGCCAGTGGGAATGGAGAGCAGGTTCTTAGCCGAGATGTGTACAGGTTGGGT
CATAGGCTCGATTTCTTCAGAATGTTATCATTTTTCTACACAACGGTGGGGTTTTTCTTCAACACGATGA
TGGTCATTCTCACTGTCTACGCTTTCCTCTGGGGCCGGGTTTATCTTGCTCTGAGCGGTGTTGAGAAGTC
CGCTCTAGCAGACAGCACAGACACCAACGCAGCGCTTGCTGTGATATTGAACCAGCAGTTCATCATTCAG
CTTGGTCTCTTCACAGCTCTGCCAATGATTGTGGAATGGTCTCTCGAGGAGGGTTTCCTTCTCGCGATAT
GGAACTTCATTCGGATGCAGATTCAGCTTTCTTCTGTCTTCTACACATTCTCAATGGGGACCAGAGCTCA
CTATTTTGGCCGAACCATTCTCCACGGTGGAGCAAAGTACAGAGCCACTGGACGTGGATTTGTTGTCGAG
CACAAGAGTTTCACTGAAAACTACCGTCTATACGCGCGCAGTCACTTTGTGAAGGCCATCGAGCTTGGGC
TGATCCTCATAGTCTACGCTACGCACAGTCCCATCGCCAAAGACTCATTGATCTATATAGCCATGACTCT

CACCAGCTGGTTCCTCGTGATTTCATGGATACTAGCCCCTTTTGTGTTCAACCCGTCAGGTTTCGACTGG
CTTAAGACGGTCTACGACTTCGAAGGCTTCATGAACTGGATCTGGTATCAAGGCAGAATCTCAACGAAGT
CCGAACAGAGCTGGGAGATATGGTGGTATGAGGAACAGGACCACCTGAGAACCACCGGTCTACCAGGAAG
AATCATGGAGATAATCTTGGACCTTCGGTTTTTCTTCTTCCAGTACGGGATTGTATACCAGCTCAAAATC
GCAAACGGATCAACCAGCATTCTCGTCTACTTACTCTCATGGATATACATCTTCGCAGTGTTTGTGTTCT
TCCTGGTAATCCAATACGCCCGTGACAAGTACTCAGCGAGAAACCACATACGGTACAGGCTCGTTCAATT
CCTCCTGATCGTGTTTGGTACACTGGTGATTGTTGCTCTCCTGGAGTTCACGCATTTCAGCTTCGTGGAT
ATCTTCACGAGTCTTCTTGCGTTCGTCCCAACCGGCTGGGGAATCTTGCTGATCGCACAGGCTTTGAGGC
CTGCGCTGCAGAAGATCGGGCTTATCTGGAACGCGGTTATCTCCCTTGCTCGGTTATATGACATACTGTT
CGGGATAGTCATCATGGTTCCCGTAGCGTTCATGTCGTGGATGCCTGGGTTTCAGTCGATGCAAACGAGG
ATCTTATTCAATGAAGCTTTTAGCAGAGGGCTTCGTATCATGCAGATTGTCACTGGGAAGAAATCTAAAG
GCGATGTCGAAGTTGAAAAAAGAAGGTCTTAA
>XM_013875983.2_*Brassica_napus*
ATGAGCCTCCGCCACCGCACCGTCCCCTCTCAACCCGGACGGCCCCCGGCGGCGGGCGCAATCGACGACG
AGCCCTACAACATCATCCCCGTCAACAACCTCCTCGCCGACCACCCCTCCCTCCGCTACCCCGAGGTCCG
CGCCGCCGCCGCCGCCCTCAAAACCGGCGGCGACCTCCGCCGCCCCACCTACGTCCAATGGCGCCCCCAC
TACGACCTCCTCGACTGGCTCGCCCTCTTCTTCGGCTTCCAGAAGGACAACGTCCGCAACCAGCGCGAGC
ACCTCGTCCTCCACCTCGCCAACGCCCAGATGCGCCTCACGCCGCCGCCGGATAACATCGATTCCCTCGA
TCCCGCCGTCGTCCGCCGTTTCCGCCGCAAGCTCCTCGGTAACTACTCGAGCTGGTGCTCGTACCTCGGG
AGGAAGTCGAACATCTGGATCTCGGATCGGAACCCCGATTCGAGGCGGGAGCTTCTCTACGTCGGCCTCT
ACCTCCTCGTGTGGGGGGAGGCGGCGAATCTTAGGTTTATGCCGGAGTGTGTCTGTTACATCTTCCACAA
TATGGCCTCGGAGCTTAACAAGATCCTCGAGGATTGCCTCGACGAGAGCACGGGGCAGCCGTACTCTCCT
AGAATCACGGGGGAGAATAGTTTCCTAAACGGCGTCGTTAAACCTATTTACGAGACGATCAAAGCTGAGA
TTAACGAGAGCAAGAACGGGACGGAGCCGCATTGTAAGTGGAGGAACTATGATGATATTAATGAATACTT
TTGGACGGATAGGTGTTTTAGTAAATTGAAATGGCCGATTGATTTGGGGAGCAGTTTCTTCAAGAGTAGT
AGAGGGAGAGGCGTTGGGAAGACAGGTTTTGTGGAGAGGAGGACGTTCTTTTACCTCTACAGGAGCTTTG
ATAGGCTTTGGGTGATGCTTGCTTTGTTTCTTCAAGCTGCTATTATAGTCGCTTGGGAGGAGAAGCCGGG
TGGAGGGTCGGTGAGGAGTCAGCTCTGGAATGCGTTGAAGTCGAGGGATGTTCGGGTGAGGCTTTTGACT
GTGTTCTTGACGTGGAGTGGGATGAGATTACTGCAGGCTGTGCTGGACGCTGCCTCGCAACGGCCGCTTA
TTTCTAGAGAGACCAAGCGGCTGTTTTTCAGAATGTTGATGAAGGTTGTAGCTGCTACGGTTTGGATAAT
TGCTTTTATTGTTCTCTACACGAACATCTGGAAGCAGAGGAAGCAAGACAGGCAGTGGTCCAGAGCCGCG
AATGACAAGATCTATCAGTTCCTTTACGCTGTGGTGGCTTTCTTGGTCCCTGAGATCCTGGCTTTGGCTC
TGTTTATAGTCCCGTGGATAAGGAACTTTCTGGAAGAGACCAATTGGAAGATATTCTTTGCTTTGACTTG
GTGGTTCCAGGGTAAAAGCTTTGTGGGTCGAGGTTTGAGAGAGGGGTTGGTGGACAACATCAAGTACTCG
ACTTTCTGGATCTTTGTCCTAGCAACGAAGTTCACGTTCAGCTACTTCCTGCAGGTTAAGCCAATGATTA
AACCCTCGAAGCTGCTATGGAATTTGAAGGAGGTGGATTATGAGTGGCATCAGTTCTTTGGCGAGAGCAA
TAGGTTTTCTGTCTTGTTATTGTGGCTGCCAGTGGTGTTGATATACCTGATGGATATCCAAATTTGGTAC
GCGATCTATTCTTCGATTGTTGGTGCTGTTGTTGGGCTGTTTGATCATCTGGGGGAGATCAGGGACATGG
GACAGCTTAGGCTGAGGTTTCAGTTCTTTGCTAGCGCTATTCAGTTCAACCTAATGCCTGAGGAACAACT
CCTGAATGCTAGAGGATTTGGTAACAAGCTTAAGGACGCCATTCATAGATTGAAGCTGAGGTATGGACTT
GGGCGGCCATTTAAGAAACTCGAGTCTAATCAGGTTGAGGCTAACAAGTTTGCGCTGATCTGGAATGAGA
TAATCTTAGCTTTCAGAGAAGAGGATATAGTCTCTGATCGAGAAGTAGAGCTACTGGAGCTGCCAAAAAA
TTCCTGGAATGTGACAGTTATCCGCTGGCCGTGTTTCCTGTTGTGCAACGAGCTTTTGCTTGCACTGAGC
CAGGCGAAAGAGCTGGTTGACGCACCTGATAAATGGCTGTGGCACAAGATATGCAAGAACGAGTACAGGC
GGTGTGCTGTGGTTGAGGCATATGAAAGCATCAAACATCTGTTGCTCTCAATCATCAAAATTGACACCGA
AGAACATAAAATTATTACAATTTTCTTTCAGATGATTGAGGTGTCTATTCAGGGTGAGCAGTTCACCAAG
ACCTTCAAAGTGGACCTATTGCCAAAGATTTATGAGACGCTACAGAAGTTGGTTGGGCTGTTGAATGATG
AGAAAGTGGATGTTGGGCGAGTGGTGAATGGTCTGCAGTCTATTTATGAGATTGCAACACGACAGTTCTT
CATAGAAAAGAAGACGACTGAACAGCTATCTACCGAGGGGTTAACTCCTCATGATCCAGCCTCAAAGTTA
CTGTTTCAGAATGCTGTTAGGCTTCCCGATGCAAGCAATGAAGACTTCTTCCGGCAGGTTAGGCGGTTAC
ACACAATTCTCACTTCTAGGGACTCTATGCACAGCGTCCCTGTGAATCTAGAGGCGAGACGGCGGATTGC
CTTCTTCAGCAATTCGCTCTTCATGAACTTGCCTCATGCACCTCAGGTGGAGAAAATGTTGGCGTTCAGT
GTTATGACTCCATACTACAGCGAGGAAGTTGTATACAGTAAAGAACAGCTCCGAAATGAGACTGAGGATG
GGATTTCAACCTTGTATTACCTGCAGACGATTTATGCCGACGAATGGAAAAATTTAAGGAACGGATGCG

TAGGGAAGGTATAAAGACAGATGTTGAGTTGTGGACAACCAAGCTGAGAGAGCTCAGGCTTTGGGCTTCC
TACAGAGGTCAGACTTTGGCACGTACAGTTCGAGGAATGATGTACTATTACAGGGCTCTTAAGATGCTTG
CTTTTCTCGACTCTGCGTCTGAAATGGACATTCGGGAGGATGCTCAGGAGCTTGGTTCAATGAGGAGTTC
GCAGGGAAATCGATTGGATGGTGTTGACGATGTAAATGACCGATCTTCTCTAAGCAGAGCAACTAGCTCT
GTGAGCATGCTGTATAAAGGCCATGAGCATGGGACTGCATTGATGAAATTCACATATGTCGTGGCGTGCC
AAATCTATGGGTCTCAAAAAGCGAAGAAAGAGCCTCAGGCAGAGGAAATTCTGTATCTTATGAAGCAAAA
CGAAGCCCTTCGTATTGCATATGTGGATGAGGTACATGCGGGCAGGGGAGAGACTGAGTATTACTCCGTT
CTGGTGAAATACGATCACACGTTGGAGAGGGAAGTGGAGATATTCCGTGTGAAGCTACCTGGTCCGGTGA
AGCTGGGTGAGGGAAAGCCAGAGAACCAGAATCATGCAATGATCTTTACCCGTGGTGATGCTGTTCAGAC
CATAGATATGAACCAGGATAATTATTTTGAGGAGGCTCTCAAGATGAGAAATTTGCTCCAGGAGTTTAGG
CATTATCATGGGATCAGAAAACCAACTATTCTTGGTGTCAGAGAGCACATCTTCACGGGTTCTGTCTCGT
CTCTGGCTTGGTTCATGTCTGCTCAGGAGACAAGTTTCGTCACTCTGGGTCAGCGTGTTCTAGCCAACCC
GCTGAAGGTCAGAATGCATTATGGTCACCCTGATGTATTTGACAGATTCTGGTTCTTGAGTCGAGGTGGC
ATCAGCAAAGCTTCTAGAGTTATAAATATCAGTGAGGACATCTTCGCCGGGTTTAATTGCACATTGCGGG
GCGGTAACGTCACCCACCACGAGTATATTCAGGTTGGGAAGGGTCGAGATGTTGGATTGAATCAAATATC
AATGTTTGAGGCTAAGGTAGCCAGTGGGAATGGAGAGCAGGTTCTTAGCCGAGATGTGTACAGGTTGGGT
CATAGGCTCGATTTCTTCAGAATGTTATCATTTTTCTACACAACGGTGGGGTTTTTCTTCAACACGATGA
TGGTCATTCTCACTGTCTACGCTTTCCTCTGGGGCCGGGTTTATCTTGCTCTGAGCGGTGTTGAGAAGTC
CGCTCTAGCAGACAGCACAGACACCAACGCAGCGCTTGCTGTGATATTGAACCAGCAGTTCATCATTCAG
CTTGGTCTCTTCACAGCTCTGCCAATGATTGTGGAATGGTCTCTCGAGGAGGGTTTCCTTCTCGCGATAT
GGAACTTCATTCGGATGCAGATTCAGCTTTCTTCTGTCTTCTACACATTCTCAATGGGGACCAGAGCTCA
CTATTTTGGCCGAACCATTCTCCACGGTGGAGCAAAGTACAGAGCCACTGGACGTGGATTTGTTGTCGAG
CACAAGAGTTTCACTGAAAACTACCGTCTATACGCGCGCAGTCACTTTGTGAAGGCCATCGAGCTTGGGC
TGATCCTCATAGTCTACGCTACGCACAGTCCCATCGCCAAAGACTCATTGATCTATATAGCCATGACTCT
CACCAGCTGGTTCCTCGTGATTTCATGGATACTAGCCCCTTTTGTGTTCAACCCGTCAGGTTTCGACTGG
CTTAAGACGGTCTACGACTTCGAAGGCTTCATGAACTGGATCTGGTATCAAGGCAGAATCTCAACGAAGT
CCGAACAGAGCTGGGAGATATGGTGGTATGAGGAACAGGACCACCTGAGAACCACCGGTCTACCAGGAAG
AATCATGGAGATAATCTTGGACCTTCGGTTTTTCTTCTTCCAGTACGGGATTGTATACCAGCTCAAAATC
GCAAACGGATCAACCAGCATTCTCGTCTACTTACTCTCATGGATATACATCTTCGCAGTGTTTGTGTTCT
TCCTGGTAATCCAATACGCCCGTGACAAGTACTCAGCGAGAAACCACATACGGTACAGGCTCGTTCAATT
CCTCCTGATCGTGTTTGGTACACTGGTGATTGTTGCTCTCCTGGAGTTCACGCATTTCAGCTTCGTGGAT
ATCTTCACGAGTCTTCTTGCGTTCGTCCCAACCGGCTGGGGAATCTTGCTGATCGCACAGGCTTTGAGGC
CTGCGCTGCAGAAGATCGGGCTTATCTGGAACGCGGTTATCTCCCTTGCTCGGTTATATGACATACTGTT
CGGGATAGTCATCATGGTCCCCGTAGCGTTCATGTCGTGGATGCCTGGGTTTCAGTCGATGCAAACGAGG
ATCTTATTCAATGAAGCTTTTAGCAGAGGGCTTCGTATCATGCAGATTGTCACTGGGAAGAAATCTAAAG
GCGATGTCGAAGTTGAAAAAAGAAGGTCTTAA
>XM_008676998.3_*Zea_mays*
ATGACCACGCCGCGCGGCACCCAGCGACGCGCGGCCGCCCCTGCGGCGGCCGCGGGCGGCGAGCCCTACA
ACATCCTGCCCATCCACGACCTCCTCGCCGAGCACCCGTCGCTCCGGTTCCCCGAGGTGCGGGCTGCCGC
GGCGGCGCTCCGGGCGGTGGGCGGCCTCCGCCCGCCGCCCTTCTCGCAGTGGCGCGCCGACCAGGACCTC
ATGGATTGGCTCGGCGCCTTCTTCGGCTTCCAGCGCGACAACGTCAGGAACCAGCGGGAGCACCTCGTGC
TCCTCCTCGCCAACGCGCAGATGCGCCTCTCCTCCGCCGACTTCTCCGACACGCTCGAGCCCCGCATCGC
GCGCCAAATCCGCAAGAAGCTGCTCCGCAACTACACCTCCTGGTGCGGCTTCCTCGGCCGCCGCCCCTCC
GTCTACGTCCCCGAGGGCGACCCGCGCGCCGATCTGCTCTACACGGGCCTGCACCTGCTCGTCTGGGGCG
AGGCTGCCAACCTGCGTTTCATGCCCGAGTGCCTCTGCTACATCTACCACCACATGGCGACCGAGCTGCA
CCGCATCCTCGAGGGCTTCATCGACACCGCCACGGGCCGCCCCGCCAACCCCGCCGTGCACGGCGAGAAC
GCCTTCCTCGTGCGCGTCGTCACGCCCATCTGCGACGTCATCCGCGCCGAGGCCGAATCCAGCCGCGACG
GCAAGGCGCCGCACGCCGCCTGGAGGAACTACGACGACATCAACGAGTACTTCTGGCGCCGTGACGTGTT
CGACCGCCTCGGCTGGCCCATGGAGCAGTCACGCCAGTTCTTCCGCACCCCGCCGGACCGCAGCCGCGTA
CGCAAGACGGGCTTCGTCGAGGTCCGCTCGTTCTGGAACATTTATAGGAGCTTCGACAGGCTGTGGGTCA
TGCTGCTTCTCTACCTGCAAGCTGCAGCAATCGTGGCGTGGGAGGGCGCCAAGTGGCCGTGGGATGATCT
GCTCTCTTCCCGTGGCTCAGAGTCCAAGGACACACAGGTGCGCGTGCTCACCGTTTTCATCACCTGGGCT
GCGCTCCGGTTCCTCCAGTCTCTGCTGGACATCGGCACACAGTTCCGCCGCGCATTTAGGGATGGCCGAA
TGCTTGCTGTGCGCATGGTGCTCAAGGCCGTTGTTGCAGCCGCTTGGGTTGTTGCGTTCGCTGTCCTATA

CATGAGGGTCTGGAACCAGAGGACAGACAATGGGCAGTGGTCGTCAGCAGCTGATTCACGGATGAGGAGC
TTCCTCTATGCAGCTGCAGCGTTTGTTATCCCTGAGGTCCTTGCCATCGTGCTCTTCATTGTGCCTTGGG
TGCGAAACGCATTGGAGAAGACAAATTGGAAGATCTGTTACGCCCTGACCTGGTGGTTCCAGAGCCGCAG
CTTTGTTGGCCGAGGACTACGTGAGGGCACATTTGACAATGTCAAGTACTCCATCTTTTGGGTGCTTTTG
CTCGCCGTGAAGTTTGCCTTCAGCTATTTTCTCCAAATCAGGCCACTTGTAAAACCCACAAAAGAGATAT
ACAATCTGAATGGAATCCAGTACACTTGGCATGAGTTCTTTGGCCAAAGCAATCGTTTTGCGGTGTTTGT
GCTCTGGTTACCAGTGGTGTTGATCTACCTCATGGATATCCAGATTTGGTATGCTATATTTTCTTCTCTG
TCGGGTGCATTTGTGGGGCTTTTTGCACATTTGGGAGAGATCAGGGACATGAAACAGCTGCGACTTAGGT
TCCAGTTCTTTGCAAGTGCCATGTCATTCAACATCATGCCAGAGGAGCAGCAGGTGAATGAGAGCTTCTT
GCCCAGCCGTTTTCGCAATTTCTGGCAGCGATTACAGCTAAGGTATGGTTTCAGCAGATCCTTTCGGAAG
ATTGAGTCAAATCAGGTGGAGGCACGGCGGTTTGCACTTGTTTGGAATGAGATAATAAGCAAGTTCCGGG
AGGAAGACATTGTTAGTGATCGTGAGGTTGAGCTTCTTGAGCTGCCACCTGAGCTGTGGAATGTGCGTGT
AATCCGTTGGCCTTGTTTCTTGCTCTGTAATGAGCTGTCACTTGCACTTGGTCAGGCAAAAGAAGTTACA
GGATCTGATCGTAGGCTATGGAGGAAGATATGTAAGAATGATTATCGTCGTTGTGCAGTGATTGAGGTCT
ATGATAGTGCAAAACACATGCTGCTTGAGATCATAAAGGAGGGGACTGAGGAGCATGGTATTGTTACACA
ACTGTTCAGCGATTTTGATGGATCCATGAAGATGGAGAAGTTCACTGTGGAGTATAAGATGACTGAGCTG
CACAATATCCATACAAGGCTTGTAGCTCTATTGGTCCTCATCCTCAAACCCACCAAAGATGTTACCAAGA
TAGTTAATGCTTTGCAGACTCTCTATGATGTTGTTGTTCGTGATTTCCAAGCTGAGAAGAGGAGCATGGA
ACAACTGAGAAGTGAAGGTCTTGCACAGTCAAGGCCCACCAGGCTTCTCTTTGTGGATGCAGTTGTGCTT
CCTGAAGAGGACAATGCTACCTTCTATAAGCAAGTGAGGCGCATGCACACCATCCTGACCTCCAGAGACT
CTATGATTAATGTCCCACAGAACCTTGAAGCTCGTCGGAGGATTGCCTTTTTCAGCAATTCATTGTTTAT
GAACATACCAAGGGCAACCCAGGTTGAGAAGATGATGTCTTTCAGTGTCTTGACGCCTTATTATAAGGAG
GAGGTGTTGTACAGCAAGGACCAACTCTATAAAGAGAATGAAGATGGCATCTCAATATTATACTATCTAA
AACAGATTTACCCAGATGAGTGGGAGTACTTCGTAGAGCGAATGAAACGTGAGGGGATGTCTGATATTAA
TGAGCTGTACAGTGAGAAGGAAAGGCTGAGGGATCTTCGGCACTGGGTCTCATATAGGGGACAGACATTA
TCACGTACTGTGAGAGGAATGATGTACTATTATGAAGCTCTCAAGATGCTGACCTTTTTGGATTCTGCCT
CTGAACATGATTTAAAGACTGGATCAAGGGAGTTAGCTACTATGGGTTCCTCAAGAATTGGATCCTCAAG
GCATGACGGAGTTGCTGGTGGGTCAGGGTATTACAGCAGGGCATCTTCCTCTCATGCATTGAGTAGAGCA
AGCAGTAGTGTGAGCTCCTTGTTTAAAGGAAGCGAATATGGGACTGTCCTCATGAAATACACATATGTAG
TTGCATGCCAGGTATATGGACAGCAGAAAGCTAAGAATGACCCCAATGCTTTTGAAATATTGGAGCTAAT
GAAAAATTATGAAGCATTACGTGTTGCTTATGTTGATGAAAGGCAGATCAATGGCAACGAAAAAGAGTTC
TTCTCTGTTCTCGTGAAATATGACCAACAATTGCAGCGGGAGGTTGAAATCTATCGGGTTAAGTTGCCTG
GAGAGTTGAAGATTGGCGAAGGAAAGCCAGAAAATCAGAATCATGCACTCATCTTCACAAGGGGCGATGC
AGTTCAAACTATTGATATGAACCAAGACAACTACTTTGAAGAAGCTCTAAAGATGAGAAACCTACTAGAA
GAGTTCAATCGTTATTATGGAATTCGCAAGCCTAAAATTCTTGGGGTTCGGGAACACGTCTTCACGGGTT
CAGTTTCTTCTCTTGCTTGGTTCATGTCTGCCCAGGAGACAAGTTTTGTCACTCTGGGGCAGCGTGTTCT
GGCTGATCCACTTAAAGTCAGAATGCATTATGGCCACCCAGATGTTTTTGATCGTCTTTGGTTTTTGGGC
CGAGGTGGTATCAGTAAAGCATCAAGAATAATCAACATTAGTGAGGACATTTTTGCTGGTTTCAACTGTA
CTCTCCGTGGTGGTAATGTTACACACCACGAGTATATCCAGGTTGGTAAAGGTAGAGATGTGGGCCTCAA
CCAGGTCTCTATGTTTGAAGCCAAGGTTGCTAGTGGCAATGGTGAACAAACTTTGAGCCGAGATGTTTAC
AGGCTGGGCCACCGATTGGATTTCTTCCGGATGCTCTCTTTCTTCTATACAACTGTTGGATTCTATTTCA
ATACAATGATGGTTGTGCTAACTGTCTATGCATTTGTATGGGGGCGCTTTTACCTTGCACTCAGTGGGCT
TGAGGACTACATCAACAAGAACACTTCCTCTTCCAATAATGCAGCCCTGGGAGCTGTTCTCAATCAGCAG
TTCGTCATACAGCTAGGCCTGTTCACAGCTTTACCCATGATTATTGAAAATTCACTTGAGCACGGGTTCC
TCACTGCTGTGTGGGATTTCATGAAAATGCAGCTACAGTTTGCATCTGTTTTCTACACATTCTCCATGGG
AACAAAGACACACTATTATGGGAGGACAATCCTTCACGGAGGAGCGAAATATCGAGCCACTGGCAGAGGT
TTTGTTGTGGAGCATAAGAAGTTTGCTGAAAACTATAGGCTCTATGCTCGCAGCCATTTCATCAAAGCTA
TAGAACTTGGTGTTATATTAACTCTTTATGCATCTTATGGCAGTGCATCTGGGAATACATTGGTGTATAT
TCTGCTGACACTTTCAAGTTGGTTTCTTGTGTCATCATGGATTCTTGCTCCATTCATTTTTAATCCCTCT
GGTTTTGACTGGCTGAAGAATTTTAATGACTTTGAGGATTTTCTAAACTGGATATGGTTCCGGGGTGGGA
TCTCAGTTCAGTCAGATCAAAGCTGGGAGAAGTGGTGGGAGGATGAAACTGATCATCTCCGGACGACAGG
TCTATGGGGCTGCATCTTGGAAATCATATTAGACCTTCGATTTTTCTTTTTTCAGTATGCAATTGTATAT
CGGCTTCACATTGCTGATAATAGTAGAAGCATCCTTGTCTATCTTCTTTCGTGGACATGCATCCTCCTAG
CTTTTGTGGCTCTTGTGACAGTGGCTTACTTTCGAGACAGATATTCAGCAAAGAAGCACATACGGTATCG

CCTTGTCCAGGCAATCATTGTTGGGAGCACTGTGGCTGCTATTGTTGTGCTGTTGGAATTCACAAAGTTT
CAGTTTGTTGACACTTTTACTAGTCTTTTGGCTTTTCTGCCGACTGGCTGGGGAATCATATCTATTGCTC
TGGTATTCAAGCCATATCTGAGGAGATCTGAGATGGTCTGGAAAACTGTGGTTACCGTGGCACGTTTGTA
TGACATATTGTTTGGAGTAATTGTTATGGCACCTGTGGCTGTATTATCATGGTTGCCTGGGCTTCAGGAA
ATGCAAACAAGGATCCTGTTCAATGAAGCTTTCAGCAGGGGACTTCACATTTCTCAAATCATTACTGGCA
AAAAGGCACATGTGTTTTGA
>XM_028338021.1_*Glycine_soja*
ATGAGTCTCCGCCACCGTCAGTCTCCGGCTTCGGTGACCTCCTCCGCCCCCGGCCGCGGCGAAGAACCGT
TCAACATCATCCCCGTGCACAACCTCCTAGCGGACCACCCTTCCCTCCGCTTCCCCGAGGTGCGCGCGGC
GGCGGCGGCGCTGCGCGCCGTCGGAGACCTCCGGCGACCGCCGTTCGGCCAATGGCGGCCGAACATGGAC
CTCCTCGACTGGCTCGCGCTCTTCTTTGGCTTCCAGCGCGACAACGTTCGCAACCAGCGCGAGCACCTCG
TCCTCCACCTCGCCAACGCTCAGATGCGCCTCACGCCGCCGCCGGACAACATCGACACGCTCGACGCTGG
CGTGCTCCGCCGCTTCCGCAAGAAGCTCCTGAAAAACTACACCTCGTGGTGCTCCTACCTAGGAAAAAAG
TCCAACATATGGATCTCCGATCGCCGCGGCGGCGCCGGTGACGATCTCCGCCGCGAGCTCCTCTACGTCT
CCCTCTACCTCCTGATCTGGGGAGAGGCCGCGAATCTCCGCTTCATGCCTGAGTGCATCTGCTACATCTT
CCACAACATGGCGAACGAGTTGAACCGAATTTTGGAAGATTTCATCGACGAGAACACCGGGCAACCGGTT
ATGCCCTCGGTTTCCGGTGAGAACGCGTTTTTGAACCTGGTAGTGAAGCCTATATATGAGACTATTAAGC
GTGAGGTTGATAGTAGTAGGAATGGAACTGCTCCTCATAGTGCTTGGAGGAACTATGATGATATTAATGA
GTATTTTTGGAGTAGGAGGTGTTTTGAGAAGCTCAAGTGGCCACTTGATATTGGGAGTAACTTTTTTGTG
ACTGCTGGTGGGGGTGGGAAGCATGTGGGGAAGACTGGGTTTGTGGAGCAGAGGTCGTTTTGGAACTTGT
TTAGAAGTTTTGATAGGCTCTGGGTGATGCTGATACTGTTTCTTCAGGCTGCGATTATCGTGGCTTGGGA
GGGGAAGACCTACCCTTGGCAGGCTTTGGAGGATAGGACTGTCCAGGTTAGGGTTTTGACCATTTTTTTC
ACCTGGAGTGGCTTGAGGTTTCTGCAGAGTTTGCTTGATGTGGGGATGCAGTATAGGTTGGTGTCGAGGG
AGACAATTGGGCTTGGCATGAGGATGGTGATGAAGTGTGTTGTGGCTGCTGGATGGATTGTTGTGTTTGG
GGTGTTTTATGCTAGGATATGGACGCAGAGGAACCAGGATAGGAGGTGGTCGCCGGCAGCGAATAATAGG
GTGTGGAACTTTCTGGTGGTTGTGTTTGTGTTCATCATTCCTGAGCTTCTGGCTGTGGCCCTTTTTGTGA
TTCCTTGGATTAGGAATTTCATTGAGAACACGAATTGGAGGATTTTCTACATGTTGTCGTGGTGGTTTCA
GAGCAGGAGTTTTGTGGGGCGTGGCTTGAGGGAAGGGCTTGTGGACAATGTGTTGTATTCATTGTTCTGG
GTTGTGGTGCTGGCCACAAAATTTTGTTTCAGTTACTTTTTGCAGGTGAAACCGATGATTGCTCCGACCA
AGGCTGTGTTGGGCCTGAAAGATGTTGATTATGAATGGCATGAGTTTTTTCATAACAGTAACCGGTTTGC
CGTTGGGTTGTTGTGGCTTCCAGTTGTTTTGATATATCTGATGGATATTCAGATTTGGTATTCGATTTAC
TCGTCTTTTGCTGGAGCGATTGTGGGGTTGCTTGAACACTTGGGTGAGATTAGAAATATGCAACAGCTGA
AATTGAGGTTCCAGTTTTTTGCCAGTGCGATTCAGTTTAATCTCATGCCAGAGGAGCAGTTGTTGAATAC
AAGGGGAACATTGAAGAGCAGGTTTAAGGATGCCATCCGCAGGTTGAAGCTCAGGTATGGGCTTGGTCGG
CCCTACAGGAAGCTTGAGTCTAACCAGATTGAGGCCAACAAGTTTGCTTTGATATGGAATGAGATAATTC
TGTCTTTTAGGGAGGAGGATATTATATCTGACAAAGAGTTTGAGTTGCTGGAGCTGCCACAGAATTCTTG
GAATGTCAGGGTGATCCGCTGGCCATGTTTTCTTCTCTGCAATGAGCTACTGTTGGCACTCAGTCAGGCC
AAAGAACTAGTTGATGATAGTGATAAGAGGCTTTATAGGAAGATATGCAAGAGTGAGTACAGGCGCTGTG
CTGTCATTGAAGCTTATGATAGTTGCAAGCACTTGCTTCTTGAGATTATCAAACCCCACACTGAAGAGCA
TTCTATTGTGACTGTTCTGTTTCAGGAAATTGATCACTCTCTTGAGATTGAGAAATTCACTAAAATGTTC
AAAACAACTGCACTGCCTAAGCTCCACAACAAGTTGATAAAACTTGTTCAGTTATTAAACAAGCCTGTTA
AAGATCCTAACCAAGTGGTGAATACCCTTCAAGCTCTTTATGAGATTGCTACCAGAGACTTGTTCAAGGA
GCAAAGAAATCCGGAACAGCTAAAGGAGGATGGTTTGGCTCAACAGAATCCTGCTGCAGGTCTACTTTTT
GAGACTGCCATTCAGTTGCCTGATGCCAACAATGAGAACTTCTATCGGCAGGTTCGGCGCTTGTATACAA
TTCTTACATCCAATGATTCAATGCAAAACATCCCAGTAAATTTGGAAGCTAGACGGAGAATTGCCTTCTT
CAGTAACTCACTTTTTATGAACATGCCTCATGCTCCCCAAGTTGAGAAAATGATGGCTTTCAGTGTTCTA
ACGCCTTACTATAGTGAAGATGTATTATTCAGCAAAGAACAGCTCAGAAATGAGAATGAAGATGGTGTTT
CAATCCTGTACTATTTGCAGACTATATATGATGATGAGTGGAAAAATTTTATGGAGAGGATGCGTCGGGA
GGGGTTGGCAAAAGACCGTGATATATGGACTGACAAACTTAGAGATTTGAGGCTCTGGGCTTCCTACAGA
GGCCAGACACTATCACGGACAGTTAGAGGAATGATGTACTACTATCGTGCCCTCAAGATGTTGACTTTTC
TGGATTCTGCATCAGAAATGGATATTCGAGAAGGTGCCCGTGAACTTGTTTCAATGAGGCGTGATGATTT
AGAGAGTTCCAACTCAAAGTCACCTTCTTCCTCCAAGAGTTTAAGTAGAGCAAGCAGTTCTGTTAGTTTG
TTATTCAAGGGCCATGAGTATGGGACTGCTTTAATGAAATTCACATATGTGATTGCTTGCCAGATATATG
GAACTCAGAAGGAAAAAAAGGATCCTCATGCTGATGAAATTTTGTATCTAATGCAAAACAATGAGGCCCT

TCGGGTTGCTTATGTTGATGAGAAAACCACTGGAAGGGATGAGAAGGAGTATTACTCTGTTCTTGTTAAG
TATGACCAACAGTTGCAGAAGGAGGTGGAAATTTACCGCGTAAAGTTGCCTGGGCCCTTGAAGCTTGGGG
AAGGAAAGCCGGAAAATCAAAATCATGCCATTATCTTCACTCGCGGTGATGCAGTTCAGACTATTGATAT
GAATCAGGACAACTACTTTGAGGAGGCACTTAAAATGCGAAATCTCTTGGAAGAATACAGGAGTTACTAT
GGTATCCGGAAACCCACTATTTTGGGAGTTAGGGAACACATTTTTACTGGTTCTGTTTCCTCTCTTGCTT
GGTTCATGTCAGCTCAGGAAACAAGTTTTGTCACCTTAGGACAGAGGGTTTTGGCAAATCCTTTGAAGGT
TAGAATGCATTATGGTCACCCAGACGTGTTTGACAGGTTTTGGTTCTTGACTCGAGGTGGTATCAGTAAA
GCTTCCAGAGTTATCAATATAAGTGAGGACATCTTTGCTGGATTTAATTGTACTCTTCGTGGAGGTAATG
TCACTCACCATGAATACATTCAGGTTGGAAAGGGAAGGGATGTTGGGTTGAATCAAGTATCAATGTTCGA
AGCAAAGGTTGCCAGTGGAAATGGGGAGCAAGTCCTAAGTAGAGATGTGTACAGATTGGGTCACAGGCTG
GATTTTTTCCGGATGCTCTCATTCTTCTACACTACTGTGGGGTTCTTCTTCAACACTATGGTGGTGGTTC
TGACTGTGTATGCATTTTTATGGGGTCGGCTATATCTTGCTCTTAGTGGTGTTGAGAAGTCAATGGAAAG
TAACAGCAATGACAATAAAGCACTTGGTACCATCTTGAATCAACAGTTCATCATCCAACTTGGGCTTTTC
ACTGCCCTTCCAATGATTGTAGAGAATTCCCTTGAGCATGGGTTCCTTCAAGCTATCTGGGATTTCTTGA
CAATGCAGCTCCAGCTTTCATCAGTTTTTTACACATTCTCAATGGGAACTCGAAGTCATTTCTTTGGACG
GACTGTTCTGCATGGTGGGGCAAAATATCGAGCTACTGGTCGTGGTTTTGTTGTAGAGCATAAAAGATTT
GCTGAAATCTATAGACTCTTTGCCCGTAGCCATTTTGTGAAAGCAATTGAATTGGGACTGATACTTGTAA
TTTATGCATCACATAGTCCTGTAGCAACTGACACATTTGTTTATATAGCCTTGACCATCACTAGTTGGTT
CTTAGTTGCATCATGGATTATGGCACCATTTGTGTTCAATCCTTCTGGCTTTGATTGGTTAAAAACTGTT
TACGATTTTGATGACTTTATGAACTGGATTTGGTACAGTGGAAGCGTATTTGCTAAGGCTGAACAGAGCT
GGGAAAGGTGGTGGTTTGAAGAGCAGGATCATCTAAAGGTAACTGGCCTTTGGGGAAAGCTTTTGGAGAT
AATCTTAGATCTTCGGTTCTTCTTTTTCCAGTATGGAATTGTCTATCAGCTAGGCATTTCTGATCACAAT
ACCAGTATTGCTGTTTACTTGCTATCCTGGATTTATGTGTTTGTTGTATCTGGGATTTACGCTGTGGTAG
TTTATGCCCGAAACAAATATGCAGCCAAAGAGCATATCTATTATCGGCTGGTCCAGTTCCTTGTCATAAT
TCTTGCAATACTTGTGATAGTTGGTTTGCTGGAATTCACTAAATTCAAATTCATGGATATTTTCACTAGC
CTGTTGGCATTCATACCCACGGGCTGGGGCCTGATATCGATTGCCCAAGTATTCCGGCCGTTTTTGCAGT
CCACTATCATTTGGGATGGTGTTGTTTCAGTGGCTCGTATATATGATATAATGTTTGGAGTCATTATCAT
GGCCCCTGTGGCACTACTATCATGGTTGCCTGGATTTCAGAATATGCAAACCAGAATTCTTTTCAATGAA
GCATTCAGCAGGGGCCTTCGGATATTCCAGATTGTTACAGGGAAAAAATCACAGAGTTGA
>XM_008676997.2_*Zea_mays*
ATGACCACGCCGCGCGGCACCCAGCGACGCGCGGCCGCCCCTGCGGCGGCCGCGGGCGGCGAGCCCTACA
ACATCCTGCCCATCCACGACCTCCTCGCCGAGCACCCGTCGCTCCGGTTCCCCGAGGTGCGGGCTGCCGC
GGCGGCGCTCCGGGCGGTGGGCGGCCTCCGCCCGCCGCCCTTCTCGCAGTGGCGCGCCGACCAGGACCTC
ATGGATTGGCTCGGCGCCTTCTTCGGCTTCCAGCGCGACAACGTCAGGAACCAGCGGGAGCACCTCGTGC
TCCTCCTCGCCAACGCGCAGATGCGCCTCTCCTCCGCCGACTTCTCCGACACGCTCGAGCCCCGCATCGC
GCGCCAAATCCGCAAGAAGCTGCTCCGCAACTACACCTCCTGGTGCGGCTTCCTCGGCCGCCGCCCCTCC
GTCTACGTCCCCGAGGGCGACCCGCGCGCCGATCTGCTCTACACGGGCCTGCACCTGCTCGTCTGGGGCG
AGGCTGCCAACCTGCGTTTCATGCCCGAGTGCCTCTGCTACATCTACCACCACATGGCGACCGAGCTGCA
CCGCATCCTCGAGGGCTTCATCGACACCGCCACGGGCCGCCCCGCCAACCCCGCCGTGCACGGCGAGAAC
GCCTTCCTCGTGCGCGTCGTCACGCCCATCTGCGACGTCATCCGCGCCGAGGCCGAATCCAGCCGCGACG
GCAAGGCGCCGCACGCCGCCTGGAGGAACTACGACGACATCAACGAGTACTTCTGGCGCCGTGACGTGTT
CGACCGCCTCGGCTGGCCCATGGAGCAGTCACGCCAGTTCTTCCGCACCCCGCCGGACCGCAGCCGCGTA
CGCAAGACGGGCTTCGTCGAGGTCCGCTCGTTCTGGAACATTTATAGGAGCTTCGACAGGCTGTGGGTCA
TGCTGCTTCTCTACCTGCAAGCTGCAGCAATCGTGGCGTGGGAGGGCGCCAAGTGGCCGTGGGATGATCT
GCTCTCTTCCCGTGGCTCAGAGTCCAAGGACACACAGGTGCGCGTGCTCACCGTTTTCATCACCTGGGCT
GCGCTCCGGTTCCTCCAGTCTCTGCTGGACATCGGCACACAGTTCCGCCGCGCATTTAGGGATGGCCGAA
TGCTTGCTGTGCGCATGGTGCTCAAGGCCGTTGTTGCAGCCGCTTGGGTTGTTGCGTTCGCTGTCCTATA
CATGAGGGTCTGGAACCAGAGGACAGACAATGGGCAGTGGTCGTCAGCAGCTGATTCACGGATGAGGAGC
TTCCTCTATGCAGCTGCAGCGTTTGTTATCCCTGAGGTCCTTGCCATCGTGCTCTTCATTGTGCCTTGGG
TGCGAAACGCATTGGAGAAGACAAATTGGAAGATCTGTTACGCCCTGACCTGGTGGTTCCAGAGCCGCAG
CTTTGTTGGCCGAGGACTACGTGAGGGCACATTTGACAATGTCAAGTACTCCATCTTTTGGGTGCTTTTG
CTCGCCGTGAAGTTTGCCTTCAGCTATTTTCTCCAAATCAGGCCACTTGTAAAACCCACAAAAGAGATAT
ACAATCTGAATGGAATCCAGTACACTTGGCATGAGTTCTTTGGCCAAAGCAATCGTTTTGCGGTGTTTGT
GCTCTGGTTACCAGTGGTGTTGATCTACCTCATGGATATCCAGATTTGGTATGCTATATTTTCTTCTCTG

TCGGGTGCATTTGTGGGGCTTTTTGCACATTTGGGAGAGATCAGGGACATGAAACAGCTGCGACTTAGGT
TCCAGTTCTTTGCAAGTGCCATGTCATTCAACATCATGCCAGAGGAGCAGCAGGTGAATGAGAGCTTCTT
GCCCAGCCGTTTTCGCAATTTCTGGCAGCGATTACAGCTAAGGTATGGTTTCAGCAGATCCTTTCGGAAG
ATTGAGTCAAATCAGGTGGAGGCACGGCGGTTTGCACTTGTTTGGAATGAGATAATAAGCAAGTTCCGGG
AGGAAGACATTGTTAGTGATCGTGAGGTTGAGCTTCTTGAGCTGCCACCTGAGCTGTGGAATGTGCGTGT
AATCCGTTGGCCTTGTTTCTTGCTCTGTAATGAGCTGTCACTTGCACTTGGTCAGGCAAAAGAAGTTACA
GGATCTGATCGTAGGCTATGGAGGAAGATATGTAAGAATGATTATCGTCGTTGTGCAGTGATTGAGGTCT
ATGATAGTGCAAAACACATGCTGCTTGAGATCATAAAGGAGGGGACTGAGGAGCATGGTATTGTTACACA
ACTGTTCAGCGATTTTGATGGATCCATGAAGATGGAGAAGTTCACTGTGGAGTATAAGATGACTGAGCTG
CACAATATCCATACAAGGCTTGTAGCTCTATTGGTCCTCATCCTCAAACCCACCAAAGATGTTACCAAGA
TAGTTAATGCTTTGCAGACTCTCTATGATGTTGTTGTTCGTGATTTCCAAGCTGAGAAGAGGAGCATGGA
ACAACTGAGAAGTGAAGGTCTTGCACAGTCAAGGCCCACCAGGCTTCTCTTTGTGGATGCAGTTGTGCTT
CCTGAAGAGGACAATGCTACCTTCTATAAGCAAGTGAGGCGCATGCACACCATCCTGACCTCCAGAGACT
CTATGATTAATGTCCCACAGAACCTTGAAGCTCGTCGGAGGATTGCCTTTTTCAGCAATTCATTGTTTAT
GAACATACCAAGGGCAACCCAGGTTGAGAAGATGATGTCTTTCAGTGTCTTGACGCCTTATTATAAGGAG
GAGGTGTTGTACAGCAAGGACCAACTCTATAAAGAGAATGAAGATGGCATCTCAATATTATACTATCTAA
AACAGATTTACCCAGATGAGTGGGAGTACTTCGTAGAGCGAATGAAACGTGAGGGGATGTCTGATATTAA
TGAGCTGTACAGTGAGAAGGAAAGGCTGAGGGATCTTCGGCACTGGGTCTCATATAGGGGACAGACATTA
TCACGTACTGTGAGAGGAATGATGTACTATTATGAAGCTCTCAAGATGCTGACCTTTTTGGATTCTGCCT
CTGAACATGATTTAAAGACTGGATCAAGGGAGTTAGCTACTATGGGTTCCTCAAGAATTGGATCCTCAAG
GCATGACGGAGTTGCTGGTGGGTCAGGGTATTACAGCAGGGCATCTTCCTCTCATGCATTGAGTAGAGCA
AGCAGTAGTGTGAGCTCCTTGTTTAAAGGAAGCGAATATGGGACTGTCCTCATGAAATACACATATGTAG
TTGCATGCCAGGTATATGGACAGCAGAAAGCTAAGAATGACCCCAATGCTTTTGAAATATTGGAGCTAAT
GAAAAATTATGAAGCATTACGTGTTGCTTATGTTGATGAAAGGCAGATCAATGGCAACGAAAAAGAGTTC
TTCTCTGTTCTCGTGAAATATGACCAACAATTGCAGCGGGAGGTTGAAATCTATCGGGTTAAGTTGCCTG
GAGAGTTGAAGATTGGCGAAGGAAAGCCAGAAAATCAGAATCATGCACTCATCTTCACAAGGGGCGATGC
AGTTCAAACTATTGATATGAACCAAGACAACTACTTTGAAGAAGCTCTAAAGATGAGAAACCTACTAGAA
GAGTTCAATCGTTATTATGGAATTCGCAAGCCTAAAATTCTTGGGGTTCGGGAACACGTCTTCACGGGTT
CAGTTTCTTCTCTTGCTTGGTTCATGTCTGCCCAGGAGACAAGTTTTGTCACTCTGGGGCAGCGTGTTCT
GGCTGATCCACTTAAAGTCAGAATGCATTATGGCCACCCAGATGTTTTTGATCGTCTTTGGTTTTTGGGC
CGAGGTGGTATCAGTAAAGCATCAAGAATAATCAACATTAGTGAGGACATTTTTGCTGGTTTCAACTGTA
CTCTCCGTGGTGGTAATGTTACACACCACGAGTATATCCAGGTTGGTAAAGGTAGAGATGTGGGCCTCAA
CCAGGTCTCTATGTTTGAAGCCAAGGTTGCTAGTGGCAATGGTGAACAAACTTTGAGCCGAGATGTTTAC
AGGCTGGGCCACCGATTGGATTTCTTCCGGATGCTCTCTTTCTTCTATACAACTGTTGGATTCTATTTCA
ATACAATGATGGTTGTGCTAACTGTCTATGCATTTGTATGGGGGCGCTTTTACCTTGCACTCAGTGGGCT
TGAGGACTACATCAACAAGAACACTTCCTCTTCCAATAATGCAGCCCTGGGAGCTGTTCTCAATCAGCAG
TTCGTCATACAGCTAGGCCTGTTCACAGCTTTACCCATGATTATTGAAAATTCACTTGAGCACGGGTTCC
TCACTGCTGTGTGGGATTTCATGAAAATGCAGCTACAGTTTGCATCTGTTTTCTACACATTCTCCATGGG
AACAAAGACACACTATTATGGGAGGACAATCCTTCACGGAGGAGCGAAATATCGAGCCACTGGCAGAGGT
TTTGTTGTGGAGCATAAGAAGTTTGCTGAAAACTATAGGCTCTATGCTCGCAGCCATTTCATCAAAGCTA
TAGAACTTGGTGTTATATTAACTCTTTATGCATCTTATGGCAGTGCATCTGGGAATACATTGGTGTATAT
TCTGCTGACACTTTCAAGTTGGTTTCTTGTGTCATCATGGATTCTTGCTCCATTCATTTTTAATCCCTCT
GGTTTTGACTGGCTGAAGAATTTTAATGACTTTGAGGATTTTCTAAACTGGATATGGTTCCGGGGTGGA
TCTCAGTTCAGTCAGATCAAAGCTGGGAGAAGTGGTGGGAGGATGAAACTGATCATCTCCGGACGACAGG
TCTATGGGGCTGCATCTTGGAAATCATATTAGACCTTCGATTTTTCTTTTTTCAGTATGCAATTGTATAT
CGGCTTCACATTGCTGATAATAGTAGAAGCATCCTTGTCTATCTTCTTTCGTGGACATGCATCCTCCTAG
CTTTTGTGGCTCTTGTGACAGTGGCTTACTTTCGAGACAGATATTCAGCAAAGAAGCACATACGGTATCG
CCTTGTCCAGGCAATCATTGTTGGGAGCACTGTGGCTGCTATTGTTGTGCTGTTGGAATTCACAAAGTTT
CAGTTTGTTGACACTTTTACTAGTCTTTTGGCTTTTCTGCCGACTGGCTGGGGAATCATATCTATTGCTC
TGGTATTCAAGCCATATCTGAGGAGATCTGAGATGGTCTGGAAAACTGTGGTTACCGTGGCACGTTTGTA
TGACATATTGTTTGGAGTAATTGTTATGGCACCTGTGGCTGTATTATCATGGTTGCCTGGGCTTCAGGAA
ATGCAAACAAGGATCCTGTTCAATGAAGCTTTCAGCAGGGGACTTCACATTTCTCAAATCATTACTGGCA
AAAAGGCACATGTGTTTTGA
>XM_006491561.3_*Citrus_sinensis*

ATGAGTAATTTGCGTCACCGAGCGGGTGCGGGCCAGTCCAGGCCGGACCGCCTTCCCGAAGAAGAAGAGG
AGCCCTACAACATTATCCCAGTTCACAACCTCTTAGCCGACCACCCTTCTCTCCGCTACCCAGAAGTCCG
CGCCGCCGCCGCCGCCTTACGCACTGTTGGAAACCTCCGCAAGCCGCCGTATGTCCAATGGTTGCCCCAC
ATGGATCTCCTTGATTGGCTTCAGCTCTTTTTCGGTTTTCAACTTGACAACGTTCGGAACGAACGGGAGC
ATCTCGTTCTTCATTTGGCTAACGCCCAGATGCGGCTCACGCCGCCGCCGGATAACATTGACACCCTCGA
CGCTGGTGTTTTGAGGAGGTTTAGGCGGAAGTTGTTGAAGAATTATACACTCTGGTGTTCCTATTTGGGG
AAGAAATCGAACATTTGGCTTTCGGATCGGAGCTCCGACCAGCGCCGTGAACTGCTGTATGTTTCTCTGT
ATCTTTTAATTTGGGGCGAGGCGGCGAATCTCCGGTTCATGCCGGAATGTTTGTGCTACATTTTCCATAA
CATGGCCATGGAGTTGAATAAGATTTTAGAAGATTACATTGATGAGAACACGGGGCAGCCAGTGATGCCT
TCGATTTCGGGCGAAAACGCTTTTCTGAACTGCGTCGTGAAGCCCATTTACGAAACGGTGAAAGCTGAGG
TGGAGAGTAGTAAGAATGGATCGGCGCCGCATTATGCGTGGAGGAACTACGATGATATTAACGAGTACTT
TTGGAGTAAGAGGTGTTTTCAGAAGCTGAAATGGCCGATTGATGTTGGGAGCAATTTCTTTGTGTTGTCG
GGTAAGACTAAGCACGTGGGGAAAACTGGGTTTGTCGAGCAACGATCGTTTTGGAACTTGTTTAGGAGTT
TTGATAGGTTGTGGGTGATGTTGATATTGTTCATTCAAGCTGCAGTTATCGTGGCCTGGGAGGAGAGAGA
GTATCCGTGGCAAGCATTGGAGGAGAGAGATGTTCAGGTTCGTGCTTTGACTGTGGTTCTTACTTGGAGT
GGATTAAGGTTTTTGCAAGCTTTGTTGGATTTCGCAATGCAACGTAGACTTGTCTCTAGGGAAACAAAGT
TACTTGGTATGAGGATGGTGTTGAAGGGTGTTGTGTCGGCCATATGGATAACTGTTTTTGGAGTGCTTTA
TGCTAGGATTTGGATGCAGAGAAATAGTGATCGCAGATGGTCTAATGAGGCGAATAACCGTTTGGTGGTT
TTTCTTCGGGCAGTGTTTGTGTTTGTCTTGCCTGAATTATTGGCTATAGCTTTGTTTATAATTCCTTGGA
TTAGGAATTTTCTCGAGAATACAAATTGGAAGATCTTTTATGCATTGACATGGTGGTTTCAAAGTAGAAG
TTTTGTTGGTCGTGGATTGAGAGAAGGGCTTGTTGACAATTTGAAGTATAGTTTGTTCTGGGTGCTTGTT
TTGGCTACAAAATTTGTATTCAGTTACTTCTTGCAGATTAAACCCATGATTGCCCCAACCAAACAATTGT
TAAAACTTAAAAATGTGGAGTATGAGTGGTATCAAGTGTTCGGTCATGGAAACAGATTGGCTGTTGGGTT
ACTGTGGGTCCCCGTGGTTTTGATTTATTTGATGGATTTGCAGCTCTTTTACTCTATTTATTCATCTTTG
GTGGGTGCAGCAGTGGGGTTGTTCCAGCACTTGGGTGAGATCCGAAATATGCAGCAGCTGAGGCTTAGAT
TTCAGTTCTTTGCGAGTGCGATGCAGTTTAATCTAATGCCTGAGGAGCAGCTGTTGGATGCGAGGGGAAC
ACTTAAGAGTAAATTTAGAGATGCCATTCATCGGCTGAAACTGAGATATGGGCTAGGAAGACCCTATAAG
AAGCTTGAATCTAACCAGGTTGAGGCAAACAGGTTTGCTCTGATATGGAATGAGATAATTGCCACTTTCA
GGGAAGAGGATATCATATCTGATAAGGAGGTTGAGCTGTTGGAGCTGCCGCAGAATACTTGGAATGTAAG
GGTTATTCGTTGGCCTTGTTTCTTGCTATGCAATGAGTTGCTTCTAGCTCTCAGCCAAGCCAAGGAGCTG
GTGGATGCTCCTGATAAGTGGCTTTGGTATAAGATCTGCAAGAATGAGTATCGGCGTTGCGCTGTGATTG
AAGCTTATGATAGCATAAAGCACTTGATTCTCCATATCATCAAAGTTAACACCGAAGAGCATTCAATTAT
TACGGTCCTGTTTCAAGAAATTGATCATTCGCTTCAGATTGAGAAGTTTACCAGAACATTCAAGATGACC
GTGCTGCCCAGGATTCATACCCAGTTGATAAAACTAGTTGATCTGTTGAACAAGCCCAAGAAAGATCTTA
ACAAGGTGGTAAACACCCTACAGGCCCTTTATGAGACTGCTATTCGAGATTTTTTCTCAGAGAAGAGAAG
CTCTGAGCAGCTTGTGGAGGATGGTTTGGCCCCTCGTAATCCGGCTGCCATGGCAGGTTTGCTGTTTGAG
ACTGCTGTTGAGTTACCTGATCCCAGTAACGAGAATTTCTATCGTCAGGTCAGGCGCTTAAACACAATTC
TAACATCCAGGGATTCAATGAATAACATTCCTGTTAATCTTGAAGCAAGGCGCAGAATAGCCTTTTTTAG
TAACTCTCTCTTCATGAATATGCCCCATGCTCCCCAAGTTGAGAAGATGATGTCCTTCAGCGTTCTCACC
CCCTACTACAATGAAGAAGTTGTTTATAGTAAAGAGCAACTTCGAACTGAAAATGAGGATGGGGTATCCA
TCCTGTATTATTTGCAAACAATTTATGCTGATGAGTGGAAGAATTTCCTGGAGAGGATGCACCGAGAAGG
AATGGTGAATGATAAAGAAATTTGGACAGAAAAGTTAAAAGATCTCAGGCTTTGGGCATCCTACAGAGGC
CAGACACTTTCCCGCACTGTTAGGGGAATGATGTATTATTATCGGGCTCTTAAGATGCTTGCTTTTCTTG
ATTCTGCTTCTGAGATGGACATTAGGGAAGGGGCCAGGGAACTTGGTTCAATGAGGCAAGATGCCAGTTT
GGATCGTATCACCTCAGAAAGGTCACCATCTTCCATGAGTTTAAGTAGAAATGGTAGTTCAGTGAGCATG
CTGTTCAAGGGCCATGAATATGGGACTGCTCTGATGAAATTCACATATGTTGTTGCCTGCCAGATATATG
GCCAGCAAAAGGACAAGAAAGACCCCCATGCTGAGGAAATCCTGTATCTGATGAAGAACAATGAAGCCCT
TCGAGTTGCCTATGTTGATGAAGTCTCTACAGGGAGGGATGAGAAGGATTATTTCTCTGTTCTTGTGAAA
TATGATAAGCAGTTAGAGAAGGAAGTAGAAATCTACAGGGTCAAGTTGCCTGGTCCGTTGAAGCTTGGGG
AGGGTAAACCGGAGAACCAAAATCATGCTTTTATCTTCACCCGAGGGGATGCAGTCCAGACAATTGATAT
GAACCAAGACAACTACTTTGAGGAGGCACTTAAAATGCGGAATTTGTTGGAAGAATACAGACACTACTA T
GGCATACGGAAACCTACCATCCTTGGTGTCAGGGAGCACATTTTTACTGGTTCAGTGTCATCACTAGCTG
GCTTTATGTCGGCACAGGAAACCAGTTTTGTTACCTTGGGTCAGCGTGTTTTGGCTAACCCTTTGAAAAT
CCGAATGCATTATGGCCATCCTGATGTCTTTGACAGGTTTTGGTTCTTGACTCGTGGAGGCCTCAGTAAA

GCTTCCAGAGTGATTAACATCAGTGAAGACATTTTTGCTGGCTTTAACTGCACACTGCGAGGAGGCAATG
TCACACACCACGAATACATCCAAGTTGGCAAGGGAAGGGATGTTGGATTGAATCAAATATCCATGTTTGA
AGCCAAGGTTGCTAGTGGAAATGGTGAGCAAGTTCTCAGTAGAGATGTCTACAGGTTGGGTCATAGACTG
GACTTCTTTCGGATGTTATCATTCTTTTACACTACTGTGGGATTCTTTTTCAACACTATGGTGATCATTT
TGACTGTATATGCATTCTTATGGGGTCGATTTTATCTGGCTCTTAGTGGTATAGAGGATGCTGTGGCAAG
CAACAGTAACAACAATAAAGCACTTGGTACTATCTTAAATCAGCAGTTCATTATCCAACTTGGTTTGTTC
ACGGCCCTACCTATGATTGTGGAGAATTCTCTTGAGCATGGATTTCTTCAAGCTATATGGGATTTCCTGA
CTATGCTGCTTCAGCTTTCATCTGTTTTCTACACATTCTCCATGGGAACTCGCAGTCACTACTTTGGCCG
GACTATCCTTCACGGTGGTGCTAAGTATCGGGCAACTGGCCGTGGTTTTGTTGTGCAGCACAAGAGCTTT
GCAGAAAATTATAGGCTTTATGCACGTAGCCACTTTATAAAGGCCATTGAACTTGGATTGATTCTTACAA
TTTATGCATCGCATAGCGCTATAACTAAGGGCACATTTGTTTACATAGCCATGACCATTTCTAGTTGGTT
TCTGGTTATGTCATGGATAATGGCTCCATTTGCTTTCAATCCTTCTGGCTTTGATTGGTTGAAGACTGTA
TATGACTTTGAGGATTTCATGAACTGGATCTGGTTCCGTGGTAGTGTGTTTGCAAAAGCTGAACAGAGCT
GGGAAAAATGGTGGTATGAGGAGCAGGATCATCTAAAGACAACTGGCATTTTGGGGAAGATAATGGAAAT
AATTCTAGACCTCCGCTTCTTCATTTTTCAGTATGGGATAGTATACCAGCTAGGTATTTCAGCTGGAAGT
ACTAGTATTGTCGTTTACTTGCTTTCTTGGATCTATGTTGTTATGGCTTTTGGGATTTATGCAATTGTAT
CATATGCTCGGGACAAATATGCAGCCATAGAACACATCTACTACCGTCTGGTTCAGTTCCTCATTGTTAT
TTTTATGATACTTGTGATAGTTGCCCTTCTGGAGTTCACAAAATTCAGGTTGATGGATCTTTTAACTAGT
CTGATGGCTTTTATCCCTACAGGGTGGGGTCTGATATTGATTGCCCAAGTATTCCGGCCCTTTTTGCAGT
CCACTAGGCTTTGGCAGCCCGTGGTCTCTGTGGCTAGATTGTATGATATAATGTTTGGAGTGATTGTCTT
GACCCCCGTGGCATTCTTATCTTGGATGCCTGGATTCCAGTCTATGCAAACAAGGATACTGTTCAATGAA
GCATTCAGCAGAGGCCTCCGAATATTCCAGATTGTTACTGGGAAAAAAGCTAAGGGCGACATGTAA
>XM_025092689.1_*Citrus_sinensis*
ATGAGTAATTTGCGTCACCGAGCGGGTGCGGGCCAGTCCAGGCCGGACCGCCTTCCCGAAGAAGAAGAGG
AGCCCTACAACATTATCCCAGTTCACAACCTCTTAGCCGACCACCCTTCTCTCCGCTACCCAGAAGTCCG
CGCCGCCGCCGCCGCCTTACGCACTGTTGGAAACCTCCGCAAGCCGCCGTATGTCCAATGGTTGCCCCAC
ATGGATCTCCTTGATTGGCTTCAGCTCTTTTTCGGTTTTCAACTTGACAACGTTCGGAACGAACGGGAGC
ATCTCGTTCTTCATTTGGCTAACGCCCAGATGCGGCTCACGCCGCCGCCGGATAACATTGACACCCTCGA
CGCTGGTGTTTTGAGGAGGTTTAGGCGGAAGTTGTTGAAGAATTATACACTCTGGTGTTCCTATTTGGGG
AAGAAATCGAACATTTGGCTTTCGGATCGGAGCTCCGACCAGCGCCGTGAACTGCTGTATGTTTCTCTGT
ATCTTTTAATTTGGGGCGAGGCGGCGAATCTCCGGTTCATGCCGGAATGTTTGTGCTACATTTTCCATAA
CATGGCCATGGAGTTGAATAAGATTTTAGAAGATTACATTGATGAGAACACGGGGCAGCCAGTGATGCCT
TCGATTTCGGGCGAAAACGCTTTTCTGAACTGCGTCGTGAAGCCCATTTACGAAACGGTGAAAGCTGAGG
TGGAGAGTAGTAAGAATGGATCGGCGCCGCATTATGCGTGGAGGAACTACGATGATATTAACGAGTACTT
TTGGAGTAAGAGGTGTTTTCAGAAGCTGAAATGGCCGATTGATGTTGGGAGCAATTTCTTTGTGTTGTCG
GGTAAGACTAAGCACGTGGGGAAAACTGGGTTTGTCGAGCAACGATCGTTTTGGAACTTGTTTAGGAGTT
TTGATAGGTTGTGGGTGATGTTGATATTGTTCATTCAAGCTGCAGTTATCGTGGCCTGGGAGGAGAGAGA
GTATCCGTGGCAAGCATTGGAGGAGAGAGATGTTCAGGTTCGTGCTTTGACTGTGGTTCTTACTTGGAGT
GGATTAAGGTTTTTGCAAGCTTTGTTGGATTTCGCAATGCAACGTAGACTTGTCTCTAGGGAAACAAAGT
TACTTGGTATGAGGATGGTGTTGAAGGGTGTTGTGTCGGCCATATGGATAACTGTTTTTGGAGTGCTTTA
TGCTAGGATTTGGATGCAGAGAAATAGTGATCGCAGATGGTCTAATGAGGCGAATAACCGTTTGGTGGTT
TTTCTTCGGGCAGTGTTTGTGTTTGTCTTGCCTGAATTATTGGCTATAGCTTTGTTTATAATTCCTTGGA
TTAGGAATTTTCTCGAGAATACAAATTGGAAGATCTTTTATGCATTGACATGGTGGTTTCAAAGTAGAAG
TTTTGTTGGTCGTGGATTGAGAGAAGGGCTTGTTGACAATTTGAAGTATAGTTTGTTCTGGGTGCTTGTT
TTGGCTACAAAATTTGTATTCAGTTACTTCTTGCAGATTAAACCCATGATTGCCCCAACCAAACAATTGT
TAAAACTTAAAAATGTGGAGTATGAGTGGTATCAAGTGTTCGGTCATGGAAACAGATTGGCTGTTGGGTT
ACTGTGGGTCCCCGTGGTTTTGATTTATTTGATGGATTTGCAGCTCTTTTACTCTATTTATTCATCTTTG
GTGGGTGCAGCAGTGGGGTTGTTCCAGCACTTGGGTGAGATCCGAAATATGCAGCAGCTGAGGCTTAGAT
TTCAGTTCTTTGCGAGTGCGATGCAGTTTAATCTAATGCCTGAGGAGCAGCTGTTGGATGCGAGGGGAAC
ACTTAAGAGTAAATTTAGAGATGCCATTCATCGGCTGAAACTGAGATATGGGCTAGGAAGACCCTATAAG
AAGCTTGAATCTAACCAGGTTGAGGCAAACAGGTTTGCTCTGATATGGAATGAGATAATTGCCACTTTCA
GGGAAGAGGATATCATATCTGATAAGGAGGTTGAGCTGTTGGAGCTGCCGCAGAATACTTGGAATGTAAG
GGTTATTCGTTGGCCTTGTTTCTTGCTATGCAATGAGTTGCTTCTAGCTCTCAGCCAAGCCAAGGAGCTG
GTGGATGCTCCTGATAAGTGGCTTTGGTATAAGATCTGCAAGAATGAGTATCGGCGTTGCGCTGTGATTG

AAGCTTATGATAGCATAAAGCACTTGATTCTCCATATCATCAAAGTTAACACCGAAGAGCATTCAATTAT
TACGGTCCTGTTTCAAGAAATTGATCATTCGCTTCAGATTGAGAAGTTTACCAGAACATTCAAGATGACC
GTGCTGCCCAGGATTCATACCCAGTTGATAAAACTAGTTGATCTGTTGAACAAGCCCAAGAAAGATCTTA
ACAAGGTGGTAAACACCCTACAGGCCCTTTATGAGACTGCTATTCGAGATTTTTCTCAGAGAAGAGAAG
CTCTGAGCAGCTTGTGGAGGATGGTTTGGCCCCTCGTAATCCGGCTGCCATGGCAGGTTTGCTGTTTGAG
ACTGCTGTTGAGTTACCTGATCCCAGTAACGAGAATTTCTATCGTCAGGTCAGGCGCTTAAACACAATTC
TAACATCCAGGGATTCAATGAATAACATTCCTGTTAATCTTGAAGCAAGGCGCAGAATAGCCTTTTTTAG
TAACTCTCTCTTCATGAATATGCCCCATGCTCCCCAAGTTGAGAAGATGATGTCCTTCAGCGTTCTCACC
CCCTACTACAATGAAGAAGTTGTTTATAGTAAAGAGCAACTTCGAACTGAAAATGAGGATGGGGTATCCA
TCCTGTATTATTTGCAAACAATTTATGCTGATGAGTGGAAGAATTTCCTGGAGAGGATGCACCGAGAAGG
AATGGTGAATGATAAAGAAATTTGGACAGAAAAGTTAAAAGATCTCAGGCTTTGGGCATCCTACAGAGGC
CAGACACTTTCCCGCACTGTTAGGGGAATGATGTATTATTATCGGGCTCTTAAGATGCTTGCTTTTCTTG
ATTCTGCTTCTGAGATGGACATTAGGGAAGGGGCCAGGGAACTTGGTTCAATGAGGCAAGATGCCAGTTT
GGATCGTATCACCTCAGAAAGGTCACCATCTTCCATGAGTTTAAGTAGAAATGGTAGTTCAGTGAGCATG
CTGTTCAAGGGCCATGAATATGGGACTGCTCTGATGAAATTCACATATGTTGTTGCCTGCCAGATATATG
GCCAGCAAAAGGACAAGAAAGACCCCCATGCTGAGGAAATCCTGTATCTGATGAAGAACAATGAAGCCCT
TCGAGTTGCCTATGTTGATGAAGTCTCTACAGGGAGGGATGAGAAGGATTATTTCTCTGTTCTTGTGAAA
TATGATAAGCAGTTAGAGAAGGAAGTAGAAATCTACAGGGTCAAGTTGCCTGGTCCGTTGAAGCTTGGGG
AGGGTAAACCGGAGAACCAAAATCATGCTTTTATCTTCACCCGAGGGGATGCAGTCCAGACAATTGATAT
GAACCAAGACAACTACTTTGAGGAGGCACTTAAAATGCGGAATTTGTTGGAAGAATACAGACACTACTAT
GGCATACGGAAACCTACCATCCTTGGTGTCAGGGAGCACATTTTTACTGGTTCAGTGTCATCACTAGCTG
GCTTTATGTCGGCACAGGAAACCAGTTTTGTTACCTTGGGTCAGCGTGTTTTGGCTAACCCTTTGAAAAT
CCGAATGCATTATGGCCATCCTGATGTCTTTGACAGGTTTTGGTTCTTGACTCGTGGAGGCCTCAGTAAA
GCTTCCAGAGTGATTAACATCAGTGAAGACATTTTTGCTGGCTTTAACTGCACACTGCGAGGAGGCAATG
TCACACACCACGAATACATCCAAGTTGGCAAGGGAAGGGATGTTGGATTGAATCAAATATCCATGTTTGA
AGCCAAGGTTGCTAGTGGAAATGGTGAGCAAGTTCTCAGTAGAGATGTCTACAGGTTGGGTCATAGACTG
GACTTCTTTCGGATGTTATCATTCTTTTACACTACTGTGGGATTCTTTTTCAACACTATGGTGATCATTT
TGACTGTATATGCATTCTTATGGGGTCGATTTTATCTGGCTCTTAGTGGTATAGAGGATGCTGTGGCAAG
CAACAGTAACAACAATAAAGCACTTGGTACTATCTTAAATCAGCAGTTCATTATCCAACTTGGTTTGTTC
ACGGCCCTACCTATGATTGTGGAGAATTCTCTTGAGCATGGATTTCTTCAAGCTATATGGGATTTCCTGA
CTATGCTGCTTCAGCTTTCATCTGTTTTCTACACATTCTCCATGGGAACTCGCAGTCACTACTTTGGCCG
GACTATCCTTCACGGTGGTGCTAAGTATCGGGCAACTGGCCGTGGTTTTGTTGTGCAGCACAAGAGCTTT
GCAGAAAATTATAGGCTTTATGCACGTAGCCACTTTATAAAGGCCATTGAACTTGGATTGATTCTTACAA
TTTATGCATCGCATAGCGCTATAACTAAGGGCACATTTGTTTACATAGCCATGACCATTTCTAGTTGGTT
TCTGGTTATGTCATGGATAATGGCTCCATTTGCTTTCAATCCTTCTGGCTTTGATTGGTTGAAGACTGTA
TATGACTTTGAGGATTTCATGAACTGGATCTGGTTCCGTGGTAGTGTGTTTGCAAAAGCTGAACAGAGCT
GGGAAAAATGGTGGTATGAGGAGCAGGATCATCTAAAGACAACTGGCATTTTGGGGAAGATAATGGAAAT
AATTCTAGACCTCCGCTTCTTCATTTTTCAGTATGGGATAGTATACCAGCTAGGTATTTCAGCTGGAAGT
ACTAGTATTGTCGTTTACTTGCTTTCTTGGATCTATGTTGTTATGGCTTTTGGGATTTATGCAATTGTAT
CATATGCTCGGGACAAATATGCAGCCATAGAACACATCTACTACCGTCTGGTTCAGTTCCTCATTGTTAT
TTTTATGATACTTGTGATAGTTGCCCTTCTGGAGTTCACAAAATTCAGGTTGATGGATCTTTTAACTAGT
CTGATGGCTTTTATCCCTACAGGGTGGGGTCTGATATTGATTGCCCAAGTATTCCGGCCCTTTTTGCAGT
CCACTAGGCTTTGGCAGCCCGTGGTCTCTGTGGCTAGATTGTATGATATAATGTTTGGAGTGATTGTCTT
GACCCCCGTGGCATTCTTATCTTGGATGCCTGGATTCCAGTCTATGCAAACAAGGATACTGTTCAATGAA
GCATTCAGCAGAGGCCTCCGAATATTCCAGATTGTTACTGGGAAAAAAGCTAAGGGCGACATGTAA
>XM_010424150.1_*Camelina_sativa*
ATGAGCCTCCGCCACCGCACCGTCCCGCCGCAAACTGGACGGCCGTTGGCGGCGGATGCTGCCGGAATCG
AAGAGGAGCCTTATAATATCATCCCCGTTAACAATCTCCTCGCCGACCATCCTTCCTTACGATTTCCCGA
GGTTCGTGCCGCCGCCGCTGCTCTTAAAACGGTAGGGGACCTTCGTCGTCCGCCGTATGTTCAATGGCGT
TCTCACTACGATCTCCTCGACTGGCTCGCCTTGTTCTTCGGTTTCCAGAAAGATAACGTTCGTAACCAGC
GTGAGCATATGGTGCTTCACCTCGCGAATGCTCAGATGCGTCTCTCTCCCCCGCCGGATAATATCGACTC
TCTCGATTCCGCTGTTGTTCGTCGGTTTCGTCGGAAGCTTCTCGGTAACTACTCTAGCTGGTGTTCGTAT
TTGGGGAAGAAATCTAATATTTGGATCTCAGATCGGAACCCTGATTCGAGACGTGAGCTTCTCTATGTTG
GACTCTATCTTCTCATATGGGGAGAGGCTGCGAATCTTAGGTTTATGCCTGAATGTATTTGTTACATCTT

CCATAACATGGCCTCCGAGCTCAACAAAATCTTAGAGGACTGCCTCGATGAGAACACCGGTCAACCTTAT
TTGCCTTCTCTCTCAGGCGAAAACGCTTTCTTAAACGGCGTCGTTAAACCTATTTACGATACAATTGGAG
CTGAGATTGATGAGAGCAAGAACGGTACAGTTGCGCATTGTAAGTGGAGGAACTACGACGATATCAATGA
GTACTTCTGGACGGATCGATGTTTCAGCAAATTGAAATGGCCGCTTGATTTGGGAAGCAATTTTTTCAAG
AGTAGAGGCAAAAGTGTAGGGAAAACTGGTTTCGTGGAGCGCAGGACGTTCTTCTACCTTTACAGGAGTT
TCGATCGTCTTTGGGTAATGCTAGCATTGTTCCTTCAAGCTGCCATTATAGTAGCTTGGGAGGAAAAGCC
TGAGGATAAGTCGGTGACAGTGCAGCTGTGGAATGCTTTGAAGGCAAGAGATGTTCAGGTGAGACTTTTG
ACCGTGTTCTTGACCTGGAGTGGTATGCGACTCTTGCAGGCTGTGCTGGACGCGGCTTCACAATTTCCTC
TCATTTCGAGAGAGACCAAAAGGCACTTTTTCAGAATGTTGATGAAAGTTATAGCTGCCACAGTTTGGAT
CTTAGCTTTCACAGTCCTCTACATTAACATCTGGAAGCAGAAGAGGCAAGACAGGCAGTGGTCCAATGCA
GCGACGAATAAGATCTACCAATTCCTTTACGCTGTGGTGGCCTTCTTGGTGCCCGAAATTCTGGCTTTGG
TTTTGTTTATAATCCCATGGATGAGAAACTTGCTGGAAGAGACCAATTGGAAAATATTCTTTGCTTTAAC
TTGGTGGTTTCAGGGCAAAAGCTTTGTGGGTCGAGGTTTGAGAGAGGGTTTAGTGGACAATATCAAGTAC
TCGACTTTCTGGATCTTTGTCCTAGCTACAAAGTTCACATTTAGCTACTTCCTGCAGGTTAAGCCAATGA
TTAAACCCTCAAAGCTGCTATGGAACTTAAAGAACTTAGATTATGAGTGGCATCAGTTTTATGGTGACAG
CAATAGGTTTTCTGTCGCATTGTTATGGTTGCCCGTTGTGTTGATATATCTGATGGATATCCAAATTTGG
TACGCAATCTATTCTTCGATTGTTGGTGCTGTTGTTGGGCTGTTTGATCATCTGGGGGAGATCAGGGACA
TGGGTCAGCTTAGGCTGAGGTTTCAATTCTTTGCTAGCGCTATTCAGTTCAACCTAATGCCTGAGGAACA
ACTCCTGAATGCTAGAGGCTTTGGTAACAAGTTCAAGGACGGCATTCATAGATTGAAGCTAAGGTATGGA
TTCGGACGGCCGTTTAAGAAACTCGAGTCGAATCATGTCGAGGCCAACAAATTTGCGTTGATCTGGAACG
AAATCATCTTAGCTTTCAGAGAAGAGGATATAGTGTCTGATCGAGAAGTAGAGCTGCTGGAGCTACCAAA
GAATTCCTGGAATGTGACGGTTATTCGCTGGCCGTGTTTCTTGTTGTGCAATGAGCTTTTGCTTGCACTG
AGCCAGGCTAGAGAGCTGATAGACGCTCCTGATAAATGGCTGTGGCACAAAATATGCAAGAATGAATACA
GGCGTTGTGCTGTAGTTGAGGCATATGACAGCATCAAACATCTATTGCTCTCAATCATCAAAGTTGAGAC
TGAAGAACATTCGATAATTACGGTCTTCTTTCAGATGATTAATCTTTCCATTGAGTCAGAACAGTTCACC
AAGACCTTTAGAGTGGACCTGTTGCCAAAGATTTACGAAACGCTGCAGAAGTTGGTTGGGCTGGTGAATG
ATGATAACAAGGATAGTGGGCGGGTGGTGAATGTTCTGCAGTCTCTTTATGAGATTGCAACTCGGCAGTT
CTTTATAGAGAAGAAGACAACTGAACAGCTGTCTAATGAAGGGTTGACTACTCGTGACCCAGCCTCAAAG
TTACTGTTCCAGAATGCTATTAGGCTTCCTGATGCAAGCAGTGAAGACTTCTACCGGCAGGTTAGGCGGT
TACACACGATTCTCACCTCTAGAGACTCTATGCACAGCGTCCCCGTGAATCTGGAGGCGAGACGGCGGAT
TGCGTTCTTCAGTAATTCACTTTTCATGAACATGCCTCATGCACCTCAGGTTGAGAAGATGATGGCGTTC
AGTGTTCTGACGCCATATTACAGTGAGGAAGTTGTATACAGCAAAGAACAGCTTCGAAATGAGACCGAGG
ATGGGATTTCAACGCTTTACTACCTGCAGACAATATATGCTGATGAATGGAAAAATTTCAAGGAACGGAT
GCATAGGGAAGGAATAAAGACAGATAGCGAGTTGTGGACAACCAAACTGAGAGAGCTCAGGCTTTGGGCT
TCCTACAGAGGTCAGACATTGGCACGTACAGTTCGGGGGATGATGTACTACTACCGGGCTCTTAAGATGC
TCGCTTTTCTTGACTCTGCGTCTGAAATGGATATTCGGGAGGGTGCTCAGGAGCTTGGTTCAGTGAGGAG
TTTGCAGGGAGAACTGGGTGGTCGATCTGATGGGGTTATCTCTGAAAATGACCGATCTTCCTTAAGCAGA
GCAAGTAGTTCCGTGAGTACGTTGTATAAAGGCCATGAGTATGGGACTGCATTGATGAAATTCACATATG
TTGTGGCGTGCCAGATCTATGGGTCTCAAAAAGCAAAGAAAGAGCCTCAGGCAGAGGAGATTCTGTATCT
GATGAAGCAAAACGAAGCTCTCCGTATTGCATATGTGGATGAGGTACCTGCGGGCAGAGGAGAGACTGAG
TATTACTCTGTTCTGGTGAAATACGATCACCAGTTGGAGAAGGAAGTGGAGATATTCCGTGTGAAGCTAC
CTGGTCCGGTGAAGCTGGGCGAGGGAAAGCCAGAGAACCAGAATCATGCAATGATCTTTACCCGTGGTGA
TGCTGTTCAGACCATTGATATGAACCAAGACAGCTATTTCGAGGAAGCGCTCAAGATGAGAAATTTGCTC
CAGGAGTACAAACATAATCATGGGATCAGAAAACCAACTATTCTTGGTGTCCGGGAGCATATCTTCACGG
GCTCGGTCTCGTCGCTGGCGTGGTTTATGTCTGCTCAGGAAACCAGTTTTGTCACTCTTGGTCAGCGTGT
TCTTGCAAACCCGCTGAAGGTCAGAATGCATTATGGCCACCCTGATGTATTTGATAGATTCTGGTTCTTG
AGTCGAGGTGGCATCAGCAAGGCTTCCAGAGTTATTAATATCAGTGAGGACATCTTTGCCGGGTTTAATT
GCACATTGAGGGGCGGAAACGTCACCCACCACGAGTATATTCAGGTTGGAAAGGGTCGGGATGTTGGACT
GAATCAGATATCAATGTTTGAGGCTAAGGTAGCGAGTGGGAACGGAGAGCAGGTTCTTAGCCGAGATGTG
TACCGGCTTGGTCACAGGCTTGATTTCTTCAGGATGTTATCATTTTTCTACACAACGGTCGGGTTTTTCT
TCAACACGATGATGGTCATTCTTACTGTTTACGCTTTCCTCTGGGGACGGGTTTATTTGGCTCTCAGCGG
GGTTGAAAAGTCTGCTCTAGCAGACAGTACAGACACCAATGCCGCACTTGGGGTGATCTTGAACCAGCAG
TTCATCATTCAGCTCGGTCTGTTCACTGCCCTGCCAATGATTGTTGAATGGTCTCTCGAGGAGGGTTTCC
TTCTGGCGATATGGAATTTCATTCGAATGCAGATTCAGCTTTCATCTGTCTTCTACACATTCTCGATGGG

GACCAGAGCTCACTATTTCGGTCGAACTATTCTCCATGGTGGGGCAAAGTATAGAGCCACTGGACGTGGA
TTTGTTGTGGAGCACAAGGGATTTACTGAGAACTACCGACTGTATGCACGCAGTCACTTTGTGAAGGCCA
TCGAGCTTGGGCTGATCCTCGTAGTCTATGCTACACACAGTCCAGTAGCCAAAGACTCGTTGGTTTACAT
AGCCATGACTATCACCAGCTGGTTCCTGGTGATTTCATGGATTATGGCCCCATTTGTGTTCAACCCATCA
GGATTTGACTGGCTGAAGACAGTCTACGACTTTGAAGACTTTATGAACTGGATCTGGTACCAAGGCAGAA
TCTCAACGAAATCTGAACAAAGCTGGGAAAAATGGTGGTATGAGGAACAAGACCACCTGAGGAACACCGG
GATAGCAGGAACAATTGTGGAGATCATTTTGGACCTCCGGTTTTTCTTCTTCCAGTATGGGATTGTATAC
CAGCTCAAAATTGCAAGTGGATCCACCAGTCTTTTGGTCTACATGTTCTCATGGATATACATCTTTGCCA
TTTTTGTGCTCTTCCTGGTAATCCAATACGCCCGGGACAAGTGTTCTGCAAAAGCTCACATACGGTACAG
ACTTGTGCAGTTCCTCTTGATTGTGTTTGCCATAATGGTGATTGTTGCTTTGCTGGAGTTCACGCATTTC
AGCTTCATCGATATCTTCACGAGTCTTCTTGCATTCATCCCAACCGGCTGGGGAATTCTGCTGATCGCAC
AGACTCAAAGGGGATGGCTGAAAAATTACAGGGTTCTCTGGAACGCTGTCGTCTCTGTTGCTCGCATGTA
TGACATATTGTTTGGGATACTAATAATGGTGCCAGTAGCTTTCTTGTCGTGGATGCCTGGATTCCAATCA
ATGCAAACGAGGATATTATTCAATGAAGCTTTTAGCAGAGGGCTTCGCATCATGCAGATTGTCACTGGGA
AGAAATCAAAAGGCGATGTCTAA
>XM_024782068.1_*Medicago_truncatula*
ATGAGTCTCCGCCACCGTCAACCCTCCTCAACTCCTCCCCACGAGGAAGAGCCCTACAACATCATCCCAA
TCCACAACCTCCTTGCCGACCATCCCTCCCTCCGTTTCCCCGAAGTCCGTGCCGCTGCCGCTGCACTTCG
CTCCGTCGGTAACCTCCGCCGTCCTCCTTTCGGCCAATGGCGACCTCACTATGACTTGCTTGACTGGCTC
GCTCTCTTCTTCGGCTTTCAGAAAGACAACGTCCGTAATCAACGGGAGCATCTTGTTCTTCACCTCGCCA
ATGCTCAGATGCGTCTCACTCCGCCGCCGGATAACATCGATACTCTCGACGCCGCTGTTCTTCGTCGTTT
TCGTAAAAAGCTTCTCAAGAATTACACTTCGTGGTGTTCTTATCTCGGTAAGAAATCAAACATCTGGATC
TTTGATAATCGGCGTACCGGAGAGCCTGACCTCCGGCGTGAGCTGCTTTATGTGTCACTTTATCTTCTAA
TCTGGGGAGAATCTGCGAATCTGAGGTTTGTTCCGGAGTGTTTGTGTTATATCTTTCACAATTTGGCGAA
TGAGTTGAATAGAATATTGGAGGATTATATTGATGATAATACAGGGCAGCCGGTGATGCCTTCGATTTCC
GGTGAGAATGCTTTTTTGAATTTTGTTGTGAAGCCTATTTATGAAACTATTAAGACTGAGGTTGATAATA
GTAGAAATGGAACTGCTCCTCATAGTGCTTGGAGAAATTATGATGATATTAATGAGTATTTTTGGAGTAG
GAGGTGTTTTGAGAAGATGAAATGGCCTCCTGATGTTGGGAGTAATTTTTTTACTACTGTTGGTAAGGGG
AAACATGTTGGTAAGACCGGATTTGTTGAGCAGAGATCGTTTTGGAATCTGTTTCGGAGTTTTGACAGGC
TTTGGATTATGTTGGTGTTGTTTCTTCAAGCTGCTATAATTGTTGCTTGGGAGGAGAGGACTTATCCGTG
GCAGGCGTTGGAGGATCGGACTGTGCAGGTTAGGGCTTTGACGATTTTCTTTACATGGAGTGGTATGAGG
TTTTTGCAGTCTTTGCTTGATGTGGGCATGCAGTATAGGTTGGTATCGAGGGAGACAAAGATGCTTGGAG
TGAGGATGTTTCTGAAATGTATTGTTGCTGCTGTGTGGATTGTTGTGTTTGGCGTGTTTTATGGGAGGAT
TTGGGAGCAGAGAAACCATGATAGGCGGTGGACAAAGGCGGCAAATGATCGGGTGCTGAATTTTCTAGAG
GCTGTGGCTGTCTTCATCATTCCGGAGGTTCTGGCTTTAGCCCTGTTTATACTTCCTTGGATTAGAAACT
TTGTGGAGAATACAAACTGGAGGATCTTTTACATGCTGTCGTGGTGGTTTCAGAGCAGAAGCTTTGTTGG
TCGGGGTTTGAGAGAAGGGCTTTATGACAACATTAAGTATTCATTGTTCTGGGTTTTCGTGCTGGCAACA
AAATTTTGCTTTAGTTACTTTTTGCAGGTCAAACCCATGATTGCCCCAACAAAGGCAGTGTTGGACCTCA
AGAATGTTGAGTATGAATGGCATGAGTTTTTCCATCACAGTAACCGATTTGCTGCTGGAATATTGTGGAT
TCCAGTTGTTTTAATTTATCTGATGGATATACAGATTTGGTATTCGATATATTCGTCTCTTGCTGGTGCG
GGTGTGGGGTTGTTTGCACACCTGGGCGAGATTCGAAATATGCAGCAGCTGAAGTTGAGGTTCCAGTTTT
TTGCCAGTGCAATCCAGTTTAATCTTATGCCTGAGGAGCAGCTGTTGAATGCTAGAGGAACATTGAAGAG
CAAGTTTAAGGATGCCATCCATAGGTTGAAGCTCAGGTATGGGCTTGGTCGGCCCTACAGGAAGCTCGAG
TCTAACCAGGTTGAGGCCAACAAGTTTGCTTTGATATGGAACGAGATAATTTTGTCTTTTAGGGAGGAGG
ATATTATCTCTGACAGAGAGGTTGAGCTGCTGGAGCTGCCACAGAACTCTTGGAATGTTCGGGTTATTCG
TTGGCCATGTTTTCTTCTCTGCAACGAGTTACTGCTTGCTCTCAGTCAGGCCAAAGAACTAGTTAATGAT
ACTGATAAGAGGCTTTATAAGAAGATATGCAGCTCTGAGTACAGACGCTGTGCTGTGATCGAAGCATATG
ATAGTGTGAAGCACTTGCTTCATGAGATTATCAAACCCAACAGTGAAGAGCATTCGATTGTGACTGTTCT
GTTTCAGGAAATTGATCACTCTCTTGAGATTGAGAAATTCACTAATACATTCAAAACAACTGCACTGCCC
CAGCTTCACCACAAGTTGATAAAACTTGTCGAGTTATTGAACAAGCCTGTCAAAGATTCTAATCAAGTGG
TGAATACCCTTCAGGCGCTTTATGAGATTGCTATCAGAGACCTTTTCAAGGATCGAAGAAATCCTAAGCA
GCTAGAGGATGATGGCTTGGCTCCACGTAATCCTGCTTCAGGTCTACTTTTTGAAAACGCTGTTCAGTTG
CCTGACACTAGCAATGAGAACTTCTATCGCCAGGTTCGGCGTCTGCACACAATTCTTACCTCCAGGGATT
CAATGCAAAACATTCCTATAAATCTAGAGGCCAGACGGAGGATTGCTTTCTTCAGCAACTCACTTTTTAT

GAATATGCCCCATGCTCCCCAAGTTGAGAAAATGCTGGCTTTCAGTGTTCTAACACCTTATTACAATGAA
GAAGTATTATACAGCAAAGAACAGCTCAGGACTGAGAATGAAGACGGGGTTTCAACCCTGTACTATTTGC
AGACTATTTATGATGATGAGTGGAAGAATTTTCTGGAGAGGATGCGTCGGGAGGGGATGATGAAAGACAG
TGATCTGTGGACTGATAAACTTAGAGATTTGAGGCTGTGGGCTTCCTACCGAGGCCAAACACTATCGCGG
ACAGTTAGAGGAATGATGTATTACTATCGGGCCCTCAAGATGTTGACATTTCTGGATTCTGCATCAGAAA
TGGACATTCGAGAAGGCTCACGCGAGCTTGTTTCGGTGAGGCAAGATAATTTAGATAGTTTCAACTCTGA
AAGGCCACCTCACCCCAAGAGTTTAAGTAGAGCGAGCAGTTCAGTAAGTTTGTTATTTAAAGGCCACGAG
TATGGGACTGCTTTGATGAAATTCACATATGTGGTTGCTTGTCAGATTTATGGAACTCAGAAGGAAAAGA
AGGATCCCCATGCTGAGGAAATTTTGTATCTAATGAAAAACAATGAAGCTCTTCGAGTTGCATATGTGGA
TGAGAGAACTACTGGAAGGGACGGGAAGGAGTATTTCTCTGTCCTTGTTAAGTATGATCAGCAGTTGGAA
AAGGAGGTTGAAGTATACAGAGTCAAACTGCCGGGTCCATTGAAGCTCGGGGAGGGAAAGCCCGAGAATC
AAAATCACGCCATCATCTTCACTCGCGGGGACGCTTTGCAGACTATAGACATGAATCAGGATAACTACTT
TGAGGAAGCACTCAAGATGAGAAATCTCCTTGAAGAGTATAGACGTTACTACGGTGTCCGGAAACCAACA
ATTTTAGGTGTCAGGGAACACATTTTCACTGGCTCCGTGTCCTCTCTTGCCTGGTTCATGTCAGCCCAGG
AGACAAGTTTCGTCACCCTAGGACAGAGGGTTTTGGCAAATCCTTTGAAAGTTCGAATGCATTATGGTCA
TCCAGATGTGTTTGACAGGTTTTGGTTCTTAACTCGAGGTGGTATCAGCAAAGCTTCCAGAGTGATCAAC
ATAAGCGAGGACATATTTGCTGGGTTCAATTGTACTCTTCGTGGAGGTAATGTCACTCACCATGAATACA
TTCAGGTTGGAAAGGGAAGGGATGTTGGTCTGAATCAAGTATCAATGTTTGAAGCCAAGGTTGCTAGTGG
CAACGGGGAACAAATCCTGAGCAGAGATGTGTACAGGTTGGGTCATAGGCTTGATTTTTTCCGAATGCTT
TCATTCTTCTACACTACTGTGGGGTTCTTCTTCAACACAATGATGGTGGTTCTGACAGTATATGCCTTTC
TATGGAGTCGACTATATCTCGCCCTTAGTGGTGTTGAGAAATCAATGGAAAGTAACAGCAATAACAACAA
AGCACTTGGTGCCATCTTAAATCAGCAGTTTATCATCCAACTTGGACTTTTCACCGCCCTTCCGATGATT
GTAGAGAATTCCCTTGAGCACGGGTTCCTCCAAGCTATCTGGGACTTCTTAACGATGCAACTTCAGCTTT
CATCCGTTTTCTACACCTTCTCCATGGGCACTCGCAGTCATTTTTTTGGCCGGACTATTCTGCACGGTGG
AGCAAAATATCGAGCCACCGGCCGTGGTTTTGTTGTAGAGCACAAGAGTTTTGCCGAAATCTATAGACTC
TTTTCCCGTAGCCATTTTGTGAAAGCTATTGAATTGGGGCTAATACTTGTAATTTATGCAACACACAGTC
CTGTAGCAACGGACACATTTGTTTATATAGCCCTGACCATTACTAGTTGGTTCTTAGTTGCATCATGGGT
GGTGGCACCGTTTGTGTTCAATCCCTCTGGCTTCGATTGGTTAAAAACTGTTTATGATTTTGATGACTTT
ATGAACTGGATTTGGTACAGTGGAAGTGTGTTTGCCAAAGCTGAACAGAGCTGGGAAAGGTGGTGGTATG
AAGAGCAGGATCATCTAAAGGTAACCGGCCTTTGGGGAAAGCTTTTGGAAATAATCTTAGACCTTCGCTT
CTTCTTTTTCCAGTATGGAATTGTTTACCAACTTGGAATTTCTGCTGGAAATAATAGTATTGCTGTATAC
TTGCTATCATGGATTTATGTTGTTGTTGTATCTGGGATTTATGCCGTGGTAGTGTATGCACGGAACAAAT
ATTCGGCGAAAGAGCATATCTATTACCGGCTTGTTCAGTTCCTTGTCATAATTCTTGCAATACTTCTGAT
AGTTGCTTTGCTGGAATTCACAGAATTCAAATTCGTGGATATTTTTACTAGCTTGTTGGCATTCCTTCCC
ACAGGATGGGGCCTGCTATTGATTGCCCAAGTTTTCCGACCATTCTTGCAGTCTACGATAATTTGGAGTG
GTGTTGTTGCAGTGGCTCGTCTTTATGATATATTATTTGGAGTCATTATTATGACTCCTGTGGCATTACT
ATCATGGTTGCCTGGCTTTCAAAATATGCAAACTAGAATTCTTTTCAACGAAGCATTCAGCAGGGGCCTC
CGCATATCCCAGATTGTTACAGGGAAGAAGTCACAAAGTTGA
>XM_022170048.2_*Helianthus_annuus*
ATGGCCAGCCGGCAGCGTCCGCCGCCGTCCGGACCACCGCACCGGTCACCAGACCACCGTAACTCCACCT
ACAACATAATCCCCATCCACAACCTTCTAGCAGACCATCCATCCCTCCGTTTTCCCGAAGTACGCGCCGC
CACCGCCGCTATACGCGCCGTGGGAGACCTCCGGAGACCACCTTTCGTCCCATGGCAGCCACACTACGAC
CTCCTGGACTGGCTCGGCTTGTTCTTCGGCTTCCAAGCCGCCAATGTCTCGAACCAGCGCGAACACCTTG
TCCTTCACCTCGCCAACGCTCAGATGCGGCTCCAGCCGCCGCCGGACAACGTAGATACCCTAGATCCCAC
CGTTATCCGCCGTTTCCGACGTAAGCTTCTCAACAACTACACTCACTGGTGCTCCTTCCTCGGCCGTAAA
TCCAATATCTGGATATCCGATAACCGACGCTCAACCGATAGCCGCCGTGAGCTGCTATACGTAGCTCTGT
ATCTACTTATTTGGGGTGAGTCTGCTAATCTTCGGTTTATACCTGAATGTATTTGTTACATATTTCATCA
TATGGCTATGGAGTTGAATAAGATATTAGAGGATTATATTGATGAGAATACCGGTATGCCTGTTTTGCCC
TCCATATCGGGGGAGAATGCGTTTTTAGAACATGTTGTTAAGCCGATTTATAATACCGTCAAGGCGGAAG
TTGAGAATAGTAGGAATGGAACTGCGCCGCATTCTCACTGGCGGAATTATGATGATGTTAATGAGTATTT
TTGGAGTAGGAGGTGTTTCGATAAGCTCAAGTGGCCGATTGATACGGGGAGTAGCTTTTTTTGGGGGGCG
GTTAAAGGGAAGACGGTTGGGAAGACGGGGTTTGTTGAGCAGCGGTCGTTTTTGAACTTGTTTAGGAGCT
TTGATAAGCTGTGGATTATGTTGGTTTTGTTTTTACAAGCGGCGATTGTTGTGTCGTGGAAGGAACGGTA
TGCGTATCCGTGGCAGGCCTTAAGGATAAGGATGTGCAAGTTAAGGTTTTGTCGGTTTTTATTACATGG

AGTGTGTTGAGGTTTGTGAAGTCGGTTCTTGATGGTTTGATGCAGTATAAGTTGGTTTCTCGGGAGACAT
TGTGGCTTGGGGTGCGGATGGTGTTGAAGGTGGTTGTTTCCGCGATTTGGATAGTTATTTTTGTGGTGTT
TTATGTTAAGATTTGGGATCAAAGAAACCATGATCGCCGGTGGTCTCCTGCAGCTAACCGGAGAATCGTA
ACGTTTCTCGAGGCCGGTTTTGTGTTCTTGATCCCTGAGATTTTAGCCCTATCGCTCTTCTTTATTCCAT
GGATTCGCAACTTTCTCGAAAACACGAATTGGAGAATTCTTTACATGGTCACATGGTGGTTTCAGAGCCG
AAGTTTTGTGGGTCGAGGGCTCCGTGAAGGTCTAGTAGACAACATCAAATACTCGCTTTTCTGGATCGTG
GTTCTCGCCACCAAGTTTTGTTTCAGTTACTTCATGCAGATAAAACCGATGATCCGACCCACTAAAGATC
TTTTAGATCTCCAAGATGTGACATACGAATGGCATGAGTTCTTCGGTAACAGCAACAGGTTTGCAGTCGG
GCTTCTTTGGGCACCGGTTGTCTTGATTTACCTAATGGACTTACAGATTTGGTACTCGATTTATTCATCG
ATTGTCGGTATGGGGGTCGGTTTGTTTAACCATCTAGGCGAGATCAGAAACATGCAGCAATTACGATTAA
GGTTTCAGTTTTTCGCTAGTGCAATGCAGTTTAATCTCATGCCTGAAGAACAAATGTTGAATCAAATTGG
AACCTTCAAGAGTAAATTCAAAGATGCGATTCACAGATTGAAACTGAGGTACGGGTTTGGCAGACCGTTC
AAGAAACTGGAGTCGAATCAAATCGAGGCTCAGAAATTCGCGTTGGTATGGAATGAAATTATTTTGACTT
TTCGAGAAGAAGATATTGTTAGCGATCGGGAAGTTGAATTGCTTGAATTACCAGAAAATACTTGGAACGT
TAGAGTGGTTCGGTGGCCGTGTTTGTTATTGTGTAACGAGTTGCTTCTTGCACTTGGCCAGGCTAAAGAA
CTAGTTGATGCGCCTGACAAATGGCTTTGGTATAAAATGGCTAAAAACGAGTATAGACGTTGTGCGGTTA
TAGAGGCTTACGAGAGCGTAAAACACCTACTGCTCACGATAGTAAAGCTCAACACTCCCGAACATTCCAT
CATCACCACTTTCTTTCAAGAAATCGATAATTCGATCGAGATAGAAAAGTTTACGAAGACTTTTAATATG
CTAATGCTTCCAAAGCTTCATTCACAGCTCATCACACTTGTGGGTCTGTTAATAAAGCCCGAGAAAAACA
TTAACAAAGTGGTGAATACGTTGCAGGCGTTGTACGAGGTTGCCACACGGGATTTCTTTAAAGATAAACG
AAACATGGATCAGTTAAAAGAAGACGGTTTAGCCCCTCAAAGGCCTGTTTCCGGTGGCGGGCTGCTTTTT
GAAAACGTTGTTGAGTTACCCGGTTCGGAAAATGAGATTTTCTATAGACAAGCAAGACGGTTACACATGA
TTCTTACTTCTCATGACGCGATGAATAATGTTCCTGTGAATCTCGAGGCTAGACGAAGAATAGCTTTTTT
TAGCAACTCGTTGTTTATGAACATGCCACACGCTCCACAAGTCGAAAAGATGATGGCGTTTAGTGTTTTA
ACACCTTACTACAATGAAGAAGTTGTGTACAACAAAGAACAGCTTAGAACCGAGAATGAAGACGGTGTTT
CGACTCTTTATTATCTGCAGACGATTTATGCTGATGAATGGAAAAACTTTTTAGAACGAATGAAACGTGA
AGGAATGAAGTCAGATGATGAATTGTGGACGATCAAACTTCGGGATCTTAGACTCTGGGCTTCGTATAGA
GGTCAAACACTGGCCCGCACGGTTAGGGGAATGATGTATTATTACCGGGCTTTAAAAATGCTCGCTTTTC
TTGATTCGGCTGCTGAGGTGGACATTCGTGAAGGGTCAGTGGAACTCCGGTCTATGAGACGTGGCGGCAG
TGATGGTGGTGGTGGTACAATGGCGGCACGGGGGATAAGTAGAGCCAATAGTACGGTTAGTACTTTGTTC
AAAGGCCATGAATACGGAACTGCTTTGATGAAATATACGTACGTAGTTGCTTGTCAGATTTATGGACTC
AAAAGGTGAAAAAAGATCCGCATGCAGATGAGATTTTGTATTTAATGAAAAACAACGAAGCTCTTCGTGT
TGCGTATGTTGATGAGGTGGTGGTGTCACAGAGCGTATCGGAGTATTACTCGGTTCTCGTGAAGTACGAT
CAAGAATTGGGAAAAGAAGTAGAGATTTATCGGGTCAAGTTGCCCGGCCCGTTAAAACTCGGAGAAGGAA
AACCCGAGAATCAAAATCACGCCATGATTTTCACTCGTGGGGATGCGGTACAAACAATCGACATGAATCA
AGACAATTATTTCGAAGAAGCACTCAAAATGAGGAACTTACTGGAGGAATATCGAAGTTATTATGGTATT
CGTAAGCCAACTATTTTAGGTGTTCGGGAAAATATCTTCACGGGCTCAGTTTCTTCACTAGCTTGGTTTA
TGTCAGCCCAAGAAACAAGCTTTGTAACACTGGGTCAACGGGTTTTAGCCAACCCGTTAAAAATCCGGTT
GCATTATGGCCACCCAGACGTATTTGACCGGTTCTGGTTCTTGACCCGTGGCGGGATTAGTAAAGCTTCG
AGGTTAATCAACTTGAGTGAAGACATTTTTGCGGGTTTTAACTGCACTTTACGAGGCGGTAACGTGACTC
ACCATGAGTATATTCAAGTCGGGAAAGGAAGAGACGTGGGTTTAAATCAAGTATCGATGTTTGAAGCGAA
AGTTGCAAGCGGAAATGGGGAGCAGGTTTTAAGCAGAGACGTATACCGTTTGGGGCACAGGCTCGATTTC
TTCAGAATGCTTTCGTTTTACTACACGACTGTCGGGTTCTTTTTCAACACTCTGATGATTTCATTAACCG
TTTACGTCTTTTTGTGGGGCCGACTGTATCTGGCATTAAGTGGGGTCGAGAGTTCCGTTGCAGAAAACGC
TAATACAAACCGAGCATTGGGCACAATTCTAAACCAACAGTTCATCGTGCAACTCGGGCTTTTTACTGCG
TTACCGATGATAGTCGAAAACTCTCTGGAGCTCGGGTTTCTCGCTGCCATTTGGGATTTCATCACAATGC
AGCTACAGTTATCATCTGTTTTCTTCACTTTCTCATTGGGTACTCGAGCTCATTACTTCGGTCGAACGAT
TCTTCACGGCGGGGCAAAATACCGGGCCACAGGTCGAGGCTTTGTTGTCGAACACAAGAAGTTTGCCGAA
AATTACCGACTTTACGCCAGGTCTCATTTCGTAAAGGCGATTGAACTGGGGCTGATACTCACAATTTACG
CAGGCTACAGCCCCGTTGCTAAAGGAACATTTACTTACATCGCATTGACCATTTCAAGCTGGTTTCTCGT
GTTTTCATGGATCATGGCACCGTTTGTGTTCAATCCTTCCGGGTTCGATTGGTTGAAAACCGTTTACGAT
TTTGAGGATTTCATGGACTGGATTTGGTTTCGAGGTGGCGTTTTCGCAAAGTCCGACGAGAGTTGGGAAG
CATGGTGGTATGAAGAACAAGACCATTTACGAACAACCGGCATTTTCGGGAAAATATTTGAAATCATTTT
GGATTTGCGGTTCTTCTTTTTCCAATACGGGATCGTTTACCAATTGGGTATTGCAGCTGGTAGCAAAAGT

ATCGTTGTCTACCTACTTTCTTGGATCTACATAATCGTTGCCCTTGTAATCTATTCGATTATCGCATACG
CACGTGAAAATTACGCGGCTAAGGATCATATATACTACCGGCTCGTCCAGTTCCTCGTTATCATTCTCGG
GTCGCTTATAATCGCTGCCATGATTCAGTTCACCGAATTCGAGTTTCTTGATCTGTTCACGAGTCTGCTG
GCGTTTATTCCAACGGGCTGGGGTTTTCTGTTGATTGCACAAGTGTTCAGACCCGTTTTGAAGAATACAT
GGATCTGGGGTACGGTTGTTTCAGTGGCCCGATTGTATGACATCTTGTTTGGAGTGATTATTATGACTCC
GGTGGCGTTTTTGTCATGGATGCCTGGTTTCCAATCTATGCAAACCCGAATTTTGTTCAATGACGCGTTT
AGTAGGGGCTTGCAGATATTCAAGATCCTTTCTGGAAAGAAATCTAAAGTCGAAAAGCAGTATTGA
>XM_031528468.1_*Punica_granatum*
ATGACCACCCGTCACCGCCACCCCTCGTCGGCCCAGCACCGGCCCGACCCCGGCGGCGACGAGGAGCCCT
ACAACATTATTCCCATCCACAACCTCCTCGCTGACCACCCCTCCCTCCGCTTCCCCGAGGTCCGCGCCGC
CGCCGCCGCCCTCCGCTCCGTCGGCGACCTCCGCAAGCCTCCCTACGCCCCATGGAAGCCCCACTACGAC
CTCCTCGATTGGCTCGCCCTCTTCTTCGGCTTCCAGGCCGACAGCGTCCGCAACCAGCGCGAGCACCTCG
TCCTCCACCTCGCCAACCTCCAGATGCGCCTCACCCCTCCCCCCGACAACATCGACTCCCTCGAACCCGC
CGTCCTTCGCCGCTTCCGCAAGAAGCTCCTCAGAAACTACACCGACTGGTGCTCGTACCTGGGCAAGAAA
TCCAACGTTTGGATCTCCGACCGCCGCCAATCAACCTCAGATCCCCGACGCGAGCTCCTCTATGTGTCCC
TCTACCTCCTCATCTGGGGCGAGGCCGCCAACTTGCGATTCATTCCCGAGTGCATCTGCTACATTTTCCA
CAACATGGCGATGGAATTGAACAAGATCCTAGAGGACTATATAGATGAGAACACAGGGCAGCCTGTCCTG
CCCTCGATATCGGGTGAGAACGCATTCCTCAACCACGTCGTCAGGCCCATCTACAACACCGTTAGGGCGG
AGGTCGAGGGGAGCAAGAACGGGACTGCCCCGCACAGCGCATGGCGGAATTACGATGACCTTAACGAGTA
TTTCTGGAGCAGGAGGTGCTTTGAGAATCTGAAGTGGCCCCTCGAGCTGGGTAGCAACTTCTTTGTGACG
AGCGGGAGGGGGAAGAAGGTTGGCAAGACGGGTTTTGTAGAGCAGAGATCGTTCTGGAACTTGTTCCGGA
GCTTCGACCGCTTGTGGGTTATGTTGATCTTGTTTCTGCAGGCGGCTATCATCATTGCGTGGGAGGGGGA
TGAGTACCCATGGCAGGCATTGAAGAGCAGGGATGTCCAAGTTCGGGTATTGACTGTTTTCTTTACTTGG
AGTGGCATGCGGTTTGTCCAGTCTTTGCTGGACATAGGAATGCAGTACAGTTTGGTGTCGAGGGAGACTA
AGCGGCTGGGACTGAGGATGGTGATGAAGAGTGTAGTTGCAGCTGCCTGGATATTGGTTTTTGGGGTGTT
TTATGGGCGGATTTGGTCGCGGAAGAACGCGGATCGGAGGTGGTCGGGAGCAGCAAACACTCGGATCGTG
AACTTTCTTGAAGTTGCGTTAGTTTTCGTCATCCCGGAGCTGTTGGCACTAGCACTGTTTATCATCCCTT
GGATTCAGAACTTCCTCGAGGAGACAAACTGGAGGATCTTCCACTTGCTAACTTGGTGGTTCCAGAGCCG
AACTTTTGTCGGTCGGGGGCTGAGGGAAGGCCTGGTAGATAATGTGAAGTATAGTCTGTTCTGGATCGCG
GTGCTTGCCACTAAGTTTGCCTTCAGTTACTTCCTGCAAATTAAGCCCATGGTTAAACCCACTAAGCTGT
TGTTGAATATGGATAACGTGAAGTACGAGTGGCATGAGTTCTTCGATAACAGCAACAGGCTTGCAGTTGG
GCTTCTTTGGGCTCCTGTGGTACTCATTTACATCATGGACTTGCAGATCTGGTATTCAATCTATTCATCC
TTCGTGGGAGCGGCAGTCGGGCTGTTGAATCACTTGGGTGAGATCCGAAACCTCGAGCAGCTGAGGTTGA
GGTTCCAGTTCTTTGCGAGTGCGATTCAGTTTAATCTGATGCCCGAGGAGCAGCTCCTGAGTGCCCGGGG
TTCATTGAGGACCAAGTTCAATGATGCCATTCACCGGCTGAAGCTGAGGTATGGGCTCGGAAGGCCGTAC
AAGAAGCTCGAGTCCAATCAGATTGAGGCTCACAAGTTTGCTTTGATATGGAATGAGATAATCACAATCT
TCAGAGAGGAGGATATCATTTCTGATAGAGAGGTTGAGCTTTTGGAGCTGGCTCCTAATACCTGGAATGT
TCGGGTGATCCGCTGGCCCTGTTTTCTTCTGTGCAATGAGCTGCTTCTTGCCCTGAGCCAAGCTAAGGAA
TTGGTGGACGCCCCTGATAGATGGCTCTGGTACAAGATTTGTAAGAACGAATACCGTCGATGTGCTGTGA
TCGAAGCTTATGATAGCATAAAGCACTTGCTGCGCCAGATCATCAAGGTGAATTCGGAAGAACATTCTAT
TGTCACGGTTCTGTTTCAGGAAATTGACAATGCTATGCAGATCGAGAAGTTCACCAAGTCATTCAAGATG
ACCGCCCTGCCTTGGCTTCACGAGAAGTTGATTAAGCTTGTTCAGATACTGGCCAGGCCTAAGAAAGATG
TAGGACAGGTGGTGAATACCCTACAGGCTCTCTATGAGATTGCTATCCGGGATTTCCTAAAGGAGAAGAA
GAGTGCTGAGCAGCTGAAGGAGGACGGGTTGGCCCCACGCAATCCCTCTTCTAATGCTGGATTGCTGTTT
GAGAATGCAGTCGTGTTACCTGATTCCAATGATGAGAAGTTCTATAGGCAGGTCCGGAGATTGCTCAGAA
TTCTTATTTCTCGTGATTCGATGCACAATGTCCCGGTGAACCTCGAAGCAAGGAGGCGCATTGCCTTCTT
TAGTAATTCTCTGTTCATGAACATGCCCCATGCGCCTCAGGTAGAGAAAATGATGGCATTCAGTGTTTTG
ACTCCGTACTACAATGAGGAAGTTTGTTACAGCAAAGAACAACTGAGGACGGAGAATGAAGATGGCATCT
CTATTTTGTACTACCTTCAGACGATTTATGACGATGAGTGGAAGAATTTCATGGAGAGGATGCGTCGAGA
GGGAGTGGTGAAGGATGACGAGATTTGGACAGAGAAGCTGAGGGATCTCAGGCTGTGGGCCTCATGCAGA
GGCCAGACTTTGGGTCGGACTGTGAGGGGAATGATGTACTACTATCGGGCCTTGAATATGCTGGCATTTC
TTGATTCTGCGTCTGAAATGGACATCAGGGAAGGAGCAAGAGAACTGGGCTCAATGAGGAGAGACGGGAG
TGTGGTGGATGGTCTTAATTCTGAAAATTCTCTTTCTTCGAAGAGTTTGAGCAGGAACGCTAGTTCAGTC
AGCACTCTCTTTAAAGGCCACGAGTATGGTACTGCCCTGATGAAGTATACCTATGTTGTTGCCTGTCAGA

TATACGGGAACCAAAAGGCGAAAAAGGATCCCCATGCCGAGGAGATCTTGTATTTAATGAAGAATAATGA
AGCCATGCGTGTAGCTTACGTTGATGAAGTAACTACAGACAGGGAGGAGAAGGAGTACTACTCTGTTCTC
GTGAAGTTTGACCAAGTATTACAGAGGGAGGTGGAGATTTACCGGGTCAAGTTGCCTGGTCCGCTGAAGC
TTGGAGAGGGAAAGCCTGAGAACCAGAACCATGCTCTAATCTTCACCCGCGGAGACGCAGTTCAAACAAT
CGACATGAACCAGGACAACTACTTTGAAGAAGCACTCAAGATGCGGAATCTGCTGGAAGAGTATCGTGCC
TATTATGGTATTCGCAAGCCCACTATCCTGGGAGTCCGTGAACACATATTTACTGGATCAGTTTCTTCAT
TAGCATGGTTCATGTCTGCCCAGGAAACGAGCTTTGTCACCCTCGGGCAGCGGGTATTGGCAAACCCTTT
GAAGATTCGAATGCACTACGGTCATCCTGATGTGTTTGACCGGTTTTGGTTCCTGACCCGTGGTGGGATC
AGCAAAGCTTCCCGGGTTATCAACATCAGCGAGGATATTTTTGCTGGGTTTAATTGCACGCTCCGAGGTG
GAAATGTTACTCATCATGAGTACATTCAAGTGGGCAAGGGACGGGATGTTGGATTGAATCAGATCTCCAT
GTTCGAAGCAAAGGTTGCCAGCGGGAACGGCGAGCAGGTCCTCAGCAGAGACGTGTACAGGCTTGGGCAC
AGGCTCGACTTCTTCCGTATGCTGTCCTTCTTCTACACCACTGTCGGGTTTTTCTTTAACACGATGATGG
TAATCTTGACTGTGTATGCATTCCTGTGGGGCCGACTTTATCTGGCTCTGAGTGGGATTGAGGGTGCTGT
AAGCAGTAGCAACAATAACAAGGCACTAGGTGCGATTCTGAGCCAGCAGTTCATCATTCAGCTTGGACTA
TTCACTGCTCTTCCCATGATAGTTGAGAACTCCCTCGAGCACGGTTTCCTTCAAGCTGTGTGGGACTTCA
TAACAATGCAGCTCCAGCTTTCGTCTGTTTTCTACACGTTCTCGATGGGAACTCGAACTCACTTCTTCGG
CAGGACCGTTCTCCATGGTGGGGCTAAGTACCGTGCAACAGGTCGTGGTTTCGTCGTGCAGCACAAGAGC
TTCGCAGAGAACTACAGGCTCTATGCTCGTAGCCATTTCGTGAAGGCAATTGAGCTCGGCCTCATTCTCA
TTGTTTATGCAACCCACAGCCCTATAGCTAAAAGCACTTTTGTCTACATAGCCATGACGATATTTAGTTG
GTTTCTTGTGTTGTCATTTATAATGGCTCCATTTGTGTTCAATCCTTCCGGGTTTGATTGGCTTAAGACA
GTATATGACTTTGATGATTTCATGAACTGGATTTGGTACCGGGGTGGCGTCTTTTCAAAAGCGGAGCAGA
GCTGGGAGAGGTGGTGGTATGAGGAGCAGGATCATTTGAGAACGACTGGTCTGTGGGGAAAGGTTGCGGA
GATAATTTTGGATCTTCGATTCTTCTTCTTTCAGTACGGTATTGTATACCAGCTGAATATTGCTGGTGGA
AGCACCAATATTGCTGTCTACTTGCTATCATGGATTTACATCTTTGTAGCTCTCGGACTCTACCTGATCA
TCTCATATGCTCGGGACAAGTATGCGGCAAGGGAACACATTTACTATAGGATGGTGCAGTTTCTTGTGAT
AATTCTTGCCATCCTCGTGATAATCGCGCTGCTGGAGTTCACCTCATTTGTGTTCGTGGACCTATTCACC
AGTCTCTTGGCGTTCATTCCTACTGGGTGGGGTGCCATATTGATTGCTCAAGTGTTTGGGCCGCTTTTGC
AGAGGACTAAGCTCTGGGACCTTGTCGTCTCTGTGGCCCGGCTATATGATATCATGTTTGGAGTCATCGT
GATGATCCCGGTGGCTTTCCTTTCTTGGATGCCTGGATTCCAGTCCATGCAGACAAGAATTCTATTTAAC
GATGCTTTCAGTCGGGGCCTGCGCATATTCCAGATTGTTACCGGGAAAAAGCCTAAGAGTGAAGTATGA
>XM_024056584.1_*Quercus_suber*
ATGAGCTACCACCGGCAACGCCCACCTCCGAGCCCAAACCCGAGCCAAAGCCGGCCCAGTCCAGCCGCAC
CGCCATTAGAATCCGACCCATACAACATAATCCCAATCCACAACCTCCTCGCGGAGCACCCGTCGCTCAG
ATTCCCGGAGGTCCGAGCCATTACCACCGCGCTCCGAGCCGTCGGCGATCTCAGGAAGCCGCCGCACAAC
CAATGGGCCCCGCACATGGACCTCCTGGACTGGCTCGCCCTCTTCTTCGGCTTCCAGAACGACAACGTTC
GCAACCAGCGCGAGCACCTCGTCCTCCACCTCGCCAACGCTCAGATGCGCCTCTCTCCTCCGCCGGACAA
CATCGACGCCCTGGACGCCGCCGTTTTGCGCAAGTTTCGGAAGAAGCTCCTCAGGAACTACACCAACTGG
TGCTCCTATCTCGGCAAGAAATCCAATATCTGGATCTCCGATCGCCGCGAGGCCGCCGCCGATCACCGCC
GGGAGCTGCTCTACGTGTCGCTCTTCCTTCTGATTTGGGGTGAGTCTGCTAACCTCCGCTTTGTTCCCGA
GTGCATTTGCTTTATATTTCATAATATGGCCATGGAGTTGAACAAGATCTTAGAGGATTACATAGACGAG
AACACCGGTCAACCTGTGCAGCCCTCGGTCTCCGGCGAAAACGCGTTTTTGAACTGCGTTGTGAAGCCGA
TTTACGATACGATTAGGGCTGAGGTGGAGAGCAGTAAGAATGGCAGTGCGCCGCACAGTGTGTGGCGCAA
TTACGACGACATTAACGAGTACTTTTGGAGCAAACGGTGTTTTCAGAAGCTCAGATGGCCGATTGATTTA
GGAAGTAATTTCTTTGTGACGAGCGAGGGTGGCGCGAGAAGTAAACACGTAGGTAAGACTGGTTTTGTGG
AGCAGAGATCCTTTTGGAACTTGTTGAGGAGCTTTGACAGGCTTTGGGTGATGCTTGTGTTGTTTTTACA
GGCGGCGATTATTGTGGCGTGGGAAGAGAGGAGATATCCATGGGAAGCTCTGCAGACTAGGGATGTCCAA
GCCAAAGTTTTGACAGTGTTTTTCACTTGGAGTGGGATGAGGTTTTTGCAGTCTCTGCTGGATGCAGGGA
TGCAATACAGTTTGGTTTCGAGGGAGACGCTGGGGCTTGGCGTGAGGATGGTGATGAAGAGTGTGGTTGC
AGCCATTTGGATCTTGGTTTTTGGTGTGTTTTATGGGCGGATATGGACGCAGAGGAATCGCGATAGGAGG
TGGTCTGCAGAGGCTAATAGGAGGTTTGTTACTTTTCTTGAGGTGGCATTGGTTTTCATTTTGCCGGAGC
TTCTGGCCGTGGTGCTGTTTGTGATTCCGTGGATCAGGAATTTCCTCGAGGAGACGAATTTGAAGATCTT
TTATATGTTGACTTGGTGGTTTCAAGGGTGGACCTTTGTGGGTCGTGGGTTGAGGGAAGGTCTTGTGGAT
AATATCAAGTACACTTTGTTTTGGATTTTGGTGCTTGCTACCAAATTTTTGTTCAGTTACTTCTTGCAGG
TTAAACCGATGATTGCCCCAACGAAAGCATTGTTGGATTTGAAGGATGTGCAGTATGAATGGTATCAGCT

TTTTAACAATAGCAACAGGTTGGCAGTGGGCTTACTGTGGATTCCTGTTGTTCTGATTTACCTCATGGAT
ATTCAGATTTGGTATTCAATCTACTCGTCTTTTGTTGGGGCTGGTGTGGGATTGTTTCAGCACTTGGGTG
AGATTCGAAATATTGAACAGTTGAGGTTGAGGTTCCAATTCTTTGCAAGTGCTATTCAGTTTAATCTCAT
GCCGGAGGAGCAGCTGTTAAATGCAAGGGGGATGAGGAACAGGTTTAAGGATGCCATTCATCGATTGAAG
CTGAGATATGGGCTTGGTCGGCCTTATAGGAAGCTTGAATCTAGTCAGGTTGAGGCGAAGAGGTTTGCTT
TGATATGGAATGAGATCATTTTGACTTTCAGGGAAGAAGATATCATCTCTGACCGTGAGCAGGAGCTGTT
GGAGCTGCCCCCGAATTCTTGGAATGTCAGGGTCATTCGATGGCCTTGTTTCCTCCTCTGTAATGAGCTT
CTGCTTGCGCTCAGTCAGGCCAAAGAGTTGTTAGATGCTCCGGACAAGTGGCTCTGGTATAAGGTATGCA
AGAATGAGTACAGGCGTTGCGCTGTGATTGAAGCTTATGATTGTATCAAACACTTGCTGCTTGAGATTAT
CAAACGCAACACAGAAGAGCATTCCATTATGACCATATTTTTTCAAGAGATTGATCACTCCATTGAGATT
GAGAGGTTCACTAAAACATTTAACATGACTGTTCTGCCCCAGCTTCATACCCAGTTAATCAAACTTGTTC
AGCTATTGAACAAGCCAAAGAAAGATACTGCCCAGGTAGTGAACACTCTGCAAGCAATTTACGAGATTGC
TAAACGGGACCTTTTCAAAGACAAGAGGAGCATTGACCAGCTGAGGGAGGATGGTCTGGCTCCTCGTAGT
CCAGCTTCCACACAGGGGCTGCTTTTTGAGAATGCTGTTGAATTGCCTAATTCAGATAATGAGACATTCT
ATAGGCAGGTTCGGCGGTTGCACACAATTCTTACCTCTCGGGACTCAATGCACGACATCCCAGTAAATCT
TGAGGCAAGACGCCGAATTGCCTTCTTCAGTAATTCACTTTTCATGAACATGCCTCATGCCCCTCAAGTT
GAGAAAATGATGGCCTTCAGTGTTCTGACCCCTTATTACAGTGAAGAAGTAATCTATAGCAAAGAACAAC
TCAAATCTGAGAATGAAGATGGTATTTCGATCCTGTTCTATTTGCAGACAATTTATGCCGATGAGTGGAA
AAATTTCATGGAGAGAATGCGCCGAGAAGGGATGGTGAAGGATACCGAGATATGGAATACTAAGCTGAGA
GATCTCAGGCTTTGGGCATCATACAGAGGTCAGACACTCTCCCGAACTGTTAGGGGAATGATGTATTACT
ATCGGGCTCTTAAGATGCTGGCATTTCTTGATTCTGCATCGGAGATGGACATTCGGGAAGGATCACAAGA
ACTTGGTTCGATGAGGCGAGACAGCGGTTTTGATGGTTACTCCTCGGAAAGGTCACCATCTTCTAGGAGT
TTAAGCAGAGCAAGCAGTTCATTGGGCTTGTTACTCAAAGGCCACGAGTATGGGACTGCATTGATGAAAT
TTACATATGTGGTTGCCTGCCAGATATATGGAACTCAGAAGGCAAAGAAAGATCCACATGCCGAGGAAAT
CTTGTATCTAATGAAAAGCAATGAAGCCCTTCGAGTTGCCTACGTTGATGAAGTTTCCACTGGGAGGGGT
GAGAAGGAGTTTTATTCTGTTCTTGTGAAGTATGATCACCAATTGGAGAGGGAAGTGGAGATCTACAGGG
TAAAGTTGCCTGGTCCCTTGAAGCTTGGTGAGGGAAAACCGGAGAATCAAAATCATGCCATCATTTTCAC
TCGTGGTGATGCTGTCCAGACTATTGATATGAACCAAGACAACTATTTTGAAGAGGCACTCAAAATGCGC
AATCTGTTGGAAGAATTCAGGCGTTATTATGGTATCCGGAAGCCTACTATCTTGGGAGTTAGGGAACACA
TATTTACAGGTTCTGTTTCATCACTTGCTTGGTTTATGTCGGCTCAGGAAACGAGTTTTGTCACCTTGGG
GCAGCGTGTTTTGGCGAACCCTTTAAAAGTTAGAATGCATTATGGCCATCCAGATGTGTTTGACAGGTTT
TGGTTCATGACTCGTGGTGGGATCAGTAAAGCTTCCAGAGTGATTAATATTAGTGAGGACATTTTTGCTG
GCTTTAACTGCACATTGCGTGGAGGGAATGTCACTCACCATGAATACATCCAGGTCGGCAAGGGAAGGGA
TGTTGGGTTGAATCAAGTATCCATGTTTGAGGCCAAGGTGGCTAGTGGAAATGGTGAGCAAGTTCTTAGC
AGAGATATCTACAGGTTGGGTCATAGGTTGGACTTCTTCCGAATGCTGTCATTCTTTTACACTACTGTGG
GATTCTTTTTCAACACAATGGTGGTGATTCTGACCGTATATGCATTTCTGTGGGGCCGACTCTATCTGGC
TCTTAGTGGTGTTGAGGCTTCTTCTGAGGCAAGTAGTACTAATAACAATGCAGCACTTGGTGCAATCTTG
AATCAGCAGTTCATTATCCAGCTTGGTCTGTTCACGGCCCTTCCGATGATAGTGGAAAACTCTCTTGAGC
ATGGGTTCCTTCAAGCTATCTGGGATTTTCTGACAATGCAGCTCCAGCTTTCATCCGTATTCTACACGTT
CTCAATGGGAACTCGTGCCCACTACTTTGGCCGTACTATTCTTCATGGTGGTGCAAAATATCGGGCTACC
GGGCGTGGTTTTGTTGTGGAGCACAAGGGTTTTGCCGAGAATTATAGACTCTATGCTCGTAGCCATTTTG
TGAAGGCAATTGAACTTGGGTTAATACTTGTAGTTTATGCGTCACACAGTCCTATAGCTGGCAACACATT
TGTTTACATAGCCTTGACAATCACTAGTTGGTTCCTGGTTGTGTCATGGATTATGGCCCCCTTTGTGTTC
AATCCCTCTGGATTTGATTGGTTGAAGACAGTGTACGACTTTGATGACTTTATGAACTGGATTTGGTTCC
GTGGCAGTGTTTTTGCAAAAGCTGAACAGAGTTGGGAAAGATGGTGGTATGAGGAGCAAGATCATCTCAG
GAACACTGGCATTTGGGGTAAAATAATGGAAATAATCTTAGACCTCCGATTCTTCTTCTTCCAGTATGGG
ATAGTATATCAACTAAATATTGCAGCTGGAAGTACCAGCATTGCTGTTTACTTCTTGTCTTGGATCTATG
TATTTGTGGCATTTGCGATTTATGCGGTAATAGCATTTGCTCGGGATAAATATGCGGCAAAAGAGCACAT
ATACTATCGTCTAGTCCAATTCCTCGTGATTGTACTTGCAATACTTGTGACAATTGCTCTGCTGCAATTT
ACGGCTTTCACATTTATTGATATTTTCACTAGCATGTTAGCATTCATCCCCACTGGGTGGGGCCTGATAT
TGATTGCCCAAGTACTCCGGCCCTTTCTGCGGTCTACTCTTGTTTGGGAAAGCGTTGTTTCTGTGGCCCG
ACTATATGATATAATGTTCGGAGTAATTGTCATGGCTCCTGTGGCATTACTGTCATGGTTGCCTGGGTTT
CAGTCAATGCAGACAAGGATCCTTTTCAACGAAGCATTTAGTCGGGGCCTCCGCATTAACCAGCTTGTTG
CAGGAAAAAAATCCAATGACTTATAA

>XM_011039030.1_*Populus_euphratica*
ATGTCTCTCCGTCGCCCGCCCCCTCCTAACCCGACCCGACCCGAATCCCAACCCTACAACATAATCCCCA
TCCAGAACCTCCTTGCCGACCACCCTTCCCTCCGCTACCCCGAAGTTCGAGCAGCCGCCGCCTCCTTACG
CACCGTCGGAAACCTCCGCAAACCACCGTACGCTCAATGGCACCCTTCAATGGACCTCCTCGACTGGCTT
GCTCTGCTCTTCGGCTTCCAAAAGGACAACGTTCGCAACCAGCGGGAGCACCTTGTCCTTCACCTTGCAA
ACGCTCAGATGCGGCTGACTCCTCCGCCGGACAACATTGATACCCTGGACGCCGGTGTACTCCGTCGGTT
CAGGCGGAAACTGCTGAAGAATTATACAAATTGGTGTGATTACTTGAATAAGAAGTCTAATATCTGGATC
TCTGACCGGTCGACGGATCTGAGAAGGGAGTTACTCTATGTCTCGTTGTATTTGTTAATTTGGGGGGAAT
CGGCGAATTTACGGTTTATGCCGGAGTGTATTTGCTTTATATTTCATAATATGTGTTTTGAATTGAATAG
AGTATTAGAGGATTATATTGATGAGAATACAGGGCAGCCGGTGATGCCTTCAATTTCAGGGGAGAATGCG
TTTTTGAATGGTGTTGTGAAGCCAATTTATGAGACGGTGAGGAGGGAAGTGGATAGGAGCTTTAACGGGG
CGGCCCCACATAGTGCTTGGCGGAATTATGATGATTTGAATGAGTATTTTTGGAGTAAGAGGTGTTTTGA
GAGGCTGAAATGGCCGATTGATTTAGGGAGTAATTTTTTTGTGACTTCGGGATCGAGGAAGAAGGTGGGG
AAGACGGGTTTTGTGGAGCAACGGTCGTTTTGGAATATTGTAAGGAGTTTTGATAGGTTGTGGGTGATGT
TGATATTGTTTTTGCAAGCGGGGATTATTGTTGCGTGGGAGGAGAAGGAGTATCCGTGGAAAGCGTTGAA
GAGCAGGGATGTGCAGGTTAGGGTGTTGACTGTTTTTTTTACTTGGAGTGGGTTGAGGTTCTTGCAGTCA
TTGCTTGATGTCGGGACACAGTATAATTTGGTTTCGAGGGAGACTTTGGGGCTTGGGGTTAGGATGATTT
TGAAAAGTGTGGTTGCAGTAGGGTGGATTATTGTGTTTGGTGCATTTTATGGGAGGATTTGGAGCCAGAG
GAACAGTGATCTGAGGAGGTCGCCCAGGGATCTGAGTTGGTCACCGGAGGCTAATAGGAAGGTAGTGACT
TTTCTTGAGGTTGCTTTGGTGTTTGTGGCGCCAGAGATGTTGGCATTGGCTCTGTTTATTCTTCCTTGGA
TTAGGAATTTTCTTGAGAATACGGATTGGAGGATATTTCGGATGATGACATGGTGGTTTCAGAGTAGTAG
TTTTATTGGTAGAGGGTTGAGGGAGGGGCTTGTGGATAATATTAAATATACTTTGTTTTGGGCTATGGTT
TTAGCTACCAAATTTGCTTTCAGTTACTTTATGCAGATTAAACCCATGGTTAAACCATCAAAACAGATGC
TGAAGCTGAAGGATGTGAATTATGAATGGCACGAGTTTTTTGACCACAGCAATAGGTTTTCGGTTGGATT
GCTGTGGCTTCCTGTGGTGTTGATTTACTTGATGGACTTGCAGATTTGGTATGCCATTTATTCCTCTTTT
GTTGGAGCAGGAGTGGGGTTGTTTCAACATTTGGGTGAGATTCGAAACATCCAGCAATTAAGATTGAGAT
TTCAGTTCTTTGCAAGTGCAATTCAGTTTAATCTGATGCCAGAGGAGCAGCTGTTGAATGCAAGGGGTAC
GTTCAAGAGCAAGTTCAAAGATGCCATTCACAGGTTGAAGCTTAGGTATGGGTTTGGCCACCCTTACAAG
AAGCTGGAGTCTAACCAGGTTGAGGCAAACAAGTTTGCTTTGATATGGAATGAGATCATAATAATTTTCA
GGGAGGAGGATATTATCTCTGACAAGGAGCTTGAGTTGATGGAGTTGCCACAGAATTCTTGGAATGTGAG
GGTGATTCGCTGGCCAAGTTTTCTCCTGTGCAATGAGCTGCTGCTTGCTCTTAGCCAGGCCAAAGAGTTG
GTAGATGCTCCTGATAAGTGGCTCTGGTACAAGATATGCAAGAACGAGTATAGGCGCTGTGCGGTCATAG
AAGCTTATGATAGTGTCAAGCACCTGTTACTTGAAATCATCAAGACCAACACAGAAGAGCACTCAATTAT
CACGGTTTTGTTTCAAGAAATTGATCACTCTCTGCAGATTGAGAAATTCACCAAGACTTTCAAGATGACA
GCTCTGCCTAATTTCCATGCCAAGTTGATAAAGCTTCTTGAGCTGTTGAACAAGCCTAAGCGGGATCTGA
ACCAGGTGGTAGATACTCTACAGGCTCTATATGAGATTGCTGTACGAGAATTTTTCAGAGACAAGAAGAA
CACTGAACAGTTGATGGAGGACGGGTTGGCTCCTCGTGACCCAGCTGCCATGGCTGGGCTTCTTTTTGGG
AATGCAGTTCAGTTGCCTGATGCTAGTAATGAGACCTTTTATAGGCAGGCACGGCGTTTGCACATGATTC
TTACCTCTAGGGATTCGATGAACACTATCCCAGAAAATCTAGAGGCCAGGCGCAGAATTGCATTTTTCAG
CAATTCCCTGTTCATGAGCATGCCCCACGCTCCCCAGGTTGAGAAAATGATGGCTTTTAGTGTGCTGACC
CCTTATTACAATGAGGAGGTGCTGTACAGCAGAGAACAGCTTCGAACTGAAAATGAAGATGGGGTTTCCA
TACTGTACTACCTGCAAACTATTTATGCTGATGAGTGGAAAAACTTCATGCAGAGGATGCGCCGTGAAGG
AATGGAAAAGGATGGTGAGATATGGACAACCAAGTTGAGAGATCTTAGGCTCTGGGCATCTTATAGAGGC
CAGACACTTGGCCGTACTGTGAGGGGAATGATGTATTATTACCGTGCTCTTAAGATGCTGGCTTTTCTTG
ATTCTGCCTCGGAGATGGACATTAAAGAGGGTTCACGAGAACTGGGCTCGATGAGGCGAGACAATGGTTT
GGATAGCTTTGACTCAGAAAGTTCTCCTTCTAAGAGCTTGAGCAGAAATAGTAGTTCAGTGAATTTGTTG
TTTAAAGGTCATGAATATGGACTGCTTTGATGAAATACACATATGTGGTTGCCTGCCAGATATACGGGG
CACAAAAGGCAAAGAAGGATCCCCATGCTGAGGAAATCTTGTATCTGATGAAAAATAATGAGGCCCTTCG
AGTTGCCTATGTTGATGAAGTAAACACTGGGAGGGATGAGATGGAATATTATTCTGTACTTGTGAAGTAT
GATCAGCAGTTGGACAAGGAAGTGGAAATCTACAGGGTGAAGTTGCCGGGTCCCTTGAAACTCGGTGAGG
GAAAACCAGAGAATCAAAATCATGCCCTAATCTTCACTCGTGGGGATGCAGTGCAGACTATTGATATGAA
CCAGGATAACTATTTTGAAGAGGCTCTCAAAATGCGGAATCTTTTGGAAGAATACAGGCACTACTATGGA
GCTCGTAAACCTACTATCTTGGGAGTCAGGGAACACATTTTTACTGGTTCTGTCTCATCTCTTGCATGGT
TTATGTCTGCTCAGGAAACTAGTTTTGTCACCCTGGGTCAGCGTGTTTTGGCAAACCCTTTGAAAATTCG

AATGCATTATGGCCATCCAGATGTCTTTGACAGGTTTTGGTTCATGACTAGAGGTGGAATCAGCAAGGCT
TCCAGAGTGATTAACATTAGTGAGGACATATTTGCTGGCTTTAATTGCACCTTGAGAGGAGGCAATATTA
CTCACCACGAATACATCCAAGTTGGAAAAGGAAGGGATGTTGGGTTGAATCAAATATCCATGTTTGAAGC
AAAAGTTGCCAGTGGAAATGGCGAGCAAACTCTTAGCAGAGATGTCTATAGATTGGGCCATAGGCTGGAC
TTCTTCCGTATGCTGTCATTCTTTTATACTACGGTGGGATTTTTTTGAACACTATGATGGTCATTCTGA
CTGTGTATGCATTTCTGTGGGGCCGTCTTTATCTGGCTCTTAGCGGTGTTGAGGGTTCTGCTTTGGCCGA
CAACAGCAGTAACAATAAGGCACTTGGTGCTATTTTGAATCAGCAATTCATCATCCAACTTGGCCTCTTC
ACTGCCCTTCCGATGATAGTGGAGAACTCTCTTGAGCACGGGTTTCTCGAAGCTATCTGGGATTTCTTGA
CAATGCAGCTCCAGCTCTCATCTGTTTTCTACACATTCTCTATGGGAACTCGGACACACTACTTTGGCCG
TACCATCCTCCATGGTGGCGCAAAATATCGGGCAACTGGGCGTGGTTTTGTTGTGCAGCACAAGAGTTTT
GCAGAGAATTACAGGCTTTATGCTCGTAGCCATTTTGTGAAGGCAATTGAGCTTGGACTGATACTTGTAG
TTTATGCAGCATACAGCCCTGTAGCTAAGGACACATTTGTTTATATAGCAATGACCATCTCTAGTTGGTT
CCTGGTTGTGTCGTGGATAATGGCCCCATTTGTCTTCAATCCATCTGGCTTTGATTGGTTGAAGACAGTA
TACGACTTCGATGATTTTATGAACTGGATTTGGTACCAAGGTGGTGTGTTTGCAAAATCTGAACAGAGCT
GGGAAAGATGGTGGTATGAGGAGCAGGATCATCTGAGGACAACTGGGCTTTGGGGAAAGTTACTGGATGT
AATATTGGACCTTCGCTTCTTCTTTTTTCAATATGGGATCGTATACCAACTAGGTATTGCTGCTGGAAGT
ACTAGCATTGCTGTTTACATGCTTTCTTGGATTTATGTAGTTGTCGCCTTCGGGTTTTTTTTGATGGTAG
CATATGCCCGGAACAAGTATGCTGCAAAAGAACACATCTACTATCGGATGGTCCAGTTTCTGATCATTGT
GCTTGGCATCTTTGTGATTATAGCCCTGCTTCAGTTTACATCTTTCAAATTTACTGATGTTTTCACAAGT
TTGTTGGCTTTTATCCCCACTGGATGGGGCATTTTATTGATTGCCCAAGTACTCCGCCCCTTCCTGCCCG
CTATACTTTGGGAAGCAGTGGTTTCTGTGGCTCGGCTATATGATATATTGTTTGGGGTAATAGTTATGGT
CCCTGTGGCATTTTTGTCATGGATGCCTGGGTTCCAATCAATGCAGACTAGGATCCTCTTCAACGAGGCA
TTCAGCAGGGGCCTCCGGATCTTCCAGCTTTTCACGGAAAAAAATCGTAG
>XM_014764671.2_*Glycine_max*
ATGAGTCTCCGCCACCGTCAGTCTCCGGCTTCGGTGACCTCCTCCGCCCCCGGCCGCGGCGAAGAACCGT
TCAACATCATCCCCGTGCACAACCTCCTAGCGGACCACCCTTCCCTCCGCTTCCCCGAGGTGCGCGCGGC
GGCGGCGGCGCTGCGCGCCGTCGGAGACCTCCGGCGACCGCCGTTCGGCCAATGGCGGCCGAACATGGAC
CTCCTCGACTGGCTCGCGCTCTTCTTTGGCTTCCAGCGCGACAACGTTCGCAACCAGCGCGAGCACCTCG
TCCTCCACCTCGCCAACGCTCAGATGCGCCTCACGCCGCCGCCGGACAACATCGACACGCTCGACGCTGG
CGTGCTCCGCCGCTTCCGCAAGAAGCTCCTGAAAAACTACACCTCGTGGTGCTCCTACCTAGGAAAAAAG
TCCAACATATGGATCTCCGATCGCCGCGGCGGCGCCGGTGACGATCTCCGCCGCGAGCTCCTCTACGTCT
CCCTCTACCTCCTGATCTGGGGAGAGGCCGCGAATCTCCGCTTCATGCCTGAGTGCATCTGCTACATCTT
CCACAACATGGCGAACGAGTTGAACCGAATTTTGGAAGATTTCATCGACGAGAACACCGGGCAACCGGTT
ATGCCCTCGGTTTCCGGTGAGAACGCGTTTTTGAACCTGGTAGTGAAGCCTATATATGAGACTATTAAGC
GTGAGGTTGATAGTAGTAGGAATGGAACTGCTCCTCATAGTGCTTGGAGGAACTATGATGATATTAATGA
GTATTTTTGGAGTAGGAGGTGTTTTGAGAAGCTCAAGTGGCCACTTGATATTGGGAGTAACTTTTTTGTG
ACTGCTGGTGGGGGTGGGAAGCATGTGGGGAAGACTGGGTTTGTGGAGCAGAGGTCGTTTTGGAACTTGT
TTAGAAGTTTTGATAGGCTCTGGGTGATGCTGATACTGTTTCTTCAGGCTGCGATTATCGTGGCTTGGGA
GGGGAAGACCTACCCTTGGCAGGCTTTGGAGGATAGGACTGTCCAGGTTAGGGTTTTGACCATTTTTTTC
ACCTGGAGTGGCTTGAGGTTTCTGCAGAGTTTGCTTGATGTGGGGATGCAGTATAGGTTGGTGTCGAGGG
AGACAATTGGGCTTGGCATGAGGATGGTGATGAAGTGTGTTGTGGCTGCTGGATGGATTGTTGTGTTTGG
GGTGTTTTATGCTAGGATATGGACGCAGAGGAACCAGGATAGGAGGTGGTCGCCGGCAGCGAATAATAGG
GTGTGGAACTTTCTGGTGGTTGTGTTTGTGTTCATCATTCCTGAGCTTCTGGCTGTGGCCCTTTTTGTGA
TTCCTTGGATTAGGAATTTCATTGAGAACACGAATTGGAGGATTTTCTACATGTTGTCGTGGTGGTTTCA
GAGCAGGAGTTTTGTGGGGCGTGGCTTGAGGGAAGGGCTTGTGGACAATTTTACATTCATGAGATTTTTC
CACGAACTCAATGTAAAACCATTAAGTCGTTTCTTTGGACGGACTGTGCTGCATTGGGGGTCAAAATATC
GAGCTACTGGTCGTGGTTTTGAAGAAGAAAATAAAAGATTTGCTGAAATCTATAGACTCTTTGCCCGTAG
CCATTTTGTGAAAGCAATTGAATTGGGACTGATACTTGTAATTTATGCATCACATAGTCCTGTAGCAACT
GACACATTTGTTTATATAGCCTTGACCATCACTAGTTGGTTCTTAGTTGCATCATGGATTATGGCACCAT
TTGTGTTCAATCCTTCTGGCTTTGATTGGTTAAAAACTGTTTACGATTTTGATGACTTTATGAACTGGAT
TTGGTACAGTGGAAGCGTATTTGCTAAGGCTGAACAGAGCTGGGAAAGGTGGTGGTTTGAAGAGCAGGAT
CATCTAAAGGTAACTGGCCTTTGGGGAAAGCTTTTGGAGATAATCTTAGATCTTCGGTTCTTCTTTTTCC
AGTATGGAATTGTCTATCAGCTAGGCATTTCTGATCACAATACCAGTATTGCTGTTTACTTGCTATCCTG
GATTTATGTGTTTGTTGTATCTGGGATTTACGCTGTGGTAGTTTATGCCCGAAACAAATATGCAGCCAAA

GAGCATATCTATTATCGGCTGGTCCAGTTCCTTGTCATAATTCTTGCAATACTTGTGATAGTTGGTTTGC
TGGAATTCACTAAATTCAAATTCATGGATATTTTCACTAGCCTGTTGGCATTCATACCCACGGGCTGGGG
CCTGATATCGATTGCCCAAGTATTCCGGCCGTTTTTGCAGTCCACTATCATTTGGGATGGTGTTGTTTCA
GTGGCTCGTATATATGATATAATGTTTGGAGTCATTATCATGGCCCCTGTGGCACTACTATCATGGTTGC
CTGGATTTCAGAATATGCAAACCAGAATTCTTTTCAATGAAGCATTCAGCAGGGGCCTTCGGATATTCCA
GATTGTTACAGGGAAAAAATCACAGAGTTGA
>XM_010071711.2_*Eucalyptus_grandis*
ATGAGCCGCCTTCGCAACCTGAATATACCCGGCACCCTAAGGGGTCGATCCCGGCCCAGCCAGACCCCGC
CTGGTGGCGATGACCAAGAACCACCGTACAACATCATCCCAGTTGCTGCCGTCCGGGCTGACCACCCCTC
CCTCCGCGTCCCGGAGGTCCGTGCTGCCTGCGCCGCCCTGCGCTCCATTGGCGGCCTCCGCCAGCCGCCA
TGGTCCCCGTGGCGCCCTGATTACGATCTCCTCGACTGGCTCCGCCTCTTCTTTGGCTTCCAGGAGGATA
ATGTCCGGAACCAACGGGAGCACCTTGTCCTCCACCTCGCCAACCTCCAGATGCGGCTCACGTCCAGGCC
GGACAACATTGAGGCCCTTGACGTCACCGTCCTCTGCTGCTTCCGGAAAAAGCTTCTCTGGAACTACACC
CTCTGGTGCTCTTACTTGGGCAGGAAGTCAAACATCTGGATCTCCGACTGCCATGATGCTGTCTCGGACC
CACGCCGTGAGCTCCTCTATGTCTCGCTCTATCTCCTTGTGTGGGGCGAGTCGGCGAACCTGCGGCTTAT
GCCAGAGTGCATTTGCTACATCTTCCACAACATGGCGATGGAAATGAACAGGATCTTAGAGAATTACATT
GACCCGAACACTGGACAGCCCGTTTTGCCTTCGGTCTCAGGAGAGAAGGCCTTTTTAAAAAAAGTTGTGG
AGCCAATTTACGACACGATTAGGAAAGAGGTCGACAGGAGCAAGAATGGGACTGCGCCCCCCGGTGCATG
GAGTAACTATGATGATATCAATGAGTACTTCTGGTCTGGAAGGTGCTTTGCAAAGTTGAGTTGGCCGATC
GATCGTCGAAGTAGCTTTTTCACGAGGGATGAGAACCGATATCGAGTTGGCAAGATGGGGTTCGTAGAGA
CGAGAATGTTTTGGAACTTGTTCCGGAGCTTCGATCGATTGTGGGTGATGTTGGTTCTGTTTCTGCAAGC
TGCAATCATTGTGGCTTGGCACGATGATAAGGAGTATCCATGGCAGGCATTGAAGAGCAGGGACCGTCAA
GTCAAGGTGTTGACTATTTTCATGACTTGGAGTGGGATGAGGTTCTTGCAGTCCCTGCTTGATGCTGGAA
CGCAGTACCTCTTGGTGTCAAGAGAAACGATGTTTTTAGGAGTGAGGATGGTGATGAAGATTGTCGTGGC
AAGTCTTTGGATGATTGCTTTTTGGGTGTTCTATGGACGGATTTGGTCGCAAAGAAATGCCGATGAGAGA
TGGTCACAAGAGGCAAACAGGAGATTGGTGCATTTTCTCGAGCTGGCCTTCGTCTTTGTTCTTCCTGAGC
TGTTGGGATTAACCCTGATGATCATTCCATGGATTCAAAATTTCCTAGAGGAAAGAAACTGGAGGATCTT
CCACCTGTACACGTGGTGGTTCCAGAGCCGTATCTTCGTGGGTCGTGGCCTTAGAGAAGGTCTGGTCGAT
AGTGTGAAGTACTCTCTATTTTGGATACATGTACTAGCCACCAAATTTTTATTCAGCTACTTCCTGCAAA
TTAAGCCCATGATCCAACCCACGAAACAGCTCTTGAAGCTGGTAGATGTAAAATACGAGTGGCACCAGTT
CTTTGGCGACAGCAACAGGTTTGCTGTTGGGCTGTTGTGGCTCCCCGTGGTGCTTATTTACCTTATGGAT
CTGCAGATTTGGTACTCGATCTATTCATCCTTGGTTGGAGCAGCAGTGGGGTTGTTAGATCACTTGGGCG
AGATCCGAAATCTGGAGCAGGTGAGATTGCGGTTCCAGTTCTTTGCGAGCGCTGTTCAGTTTAATCTAAT
GCCTGAGGGGCAGCTTTTGCAAACGATAGGAAACAAGTTCAAGGATTTGCTTCACCGACTGAAGCTGAGG
TATGGACTGGGCAAGCCGTACCAGAAGTTTGAACCTAATCAGATCGAAGCGCTGAGGTTTGCTTTGCTTT
GGAATGAGATCATAGCCATATTTAGGGAAGAGGATATAGTATCTGACCAGGAAGTTGAGCTTTTGGAGCT
ACGGAGGAGTGCTTGGAGTGATAGCGTTATTTCCTGGCCATGTTTTCTCCTGTTCAATGAGCTGTTGCTT
GCTTTAAGACAGGCCAAGGAGCTAGTTGATGCTACTGACAAGAGGCTCTGGTATAAGATCTGCAAGAACG
AGTACAGGCGCTGCGCTGTGATCGAAGCATACGACAGTGTCAAGCACTTGCTGCTTGAAATAGTCAAAAT
TGACTCGAAGGAGCATTCCGTGATCACTGATTTGTTCGAAAAGATTGATCGCTCCATCAAGGATGGGAAA
TTTACGAAGCAATTCAACTTGTCCATGTTGCCCGAGATACACAAATATATATCAAAGTTGGCTAAGCTAT
TGAAAGATTCCAAGAAAGACGAGGGCAAAATTGTGCTCGCTCTGCAGGCCCTTTATCGGATTGTTATTAG
GGAGTTCTCCATAGATGGGGAGGATCAGTTGGCATCTGGTGGTCGATTGCCTTTCATAGATGCAGTTGAA
TTGCCGAGTAATAACCAGTTCCATTGGCAAGTTCGTCGCTTGCACATAATTCTGACCTCGAGGGACTCTA
CACACAATGTTCCTTCAAATGTTGAGGCGAGACGGCGAATTTGCTTTTTCAGTAATTCGCTATTTATGAA
CATGCCCCACGCACCTAAAGTGGAGAAAATGTTTGGTTTCAGTGTTCTGACTCCTTACCACAATGAAGAG
GTACTGTACAGCAAAGAACAGCTTCTCGAAAAGAACGAAGATGGAGTTTGCATATTGTTTTATTTGAAGA
CTGTATATCCTGATGAGTGGAAATATTTCATAGAGAGAATGCTTAGAGAAGGAAAGGTGGTGAAGGCCGA
TGAGGACGATGAAACGATGGCCAAGTTGAGAGATCTTAGGCTTTGGGCGTCATACAGAGGCCTAACACTT
GCTCGAGCAGTCAGAGGAATGACGTATTACCATCGAGCACTTAAGGTGATGGCTTTTCTTGATTCTGCAT
CAGAGGTGGAAATTCAGGAATTAGCAAAAAGACTGGACTCAGCGGGGCAAGATGGCAGCACAAACAGTTC
TAGCTCGGAAGAGTTGCCTTCCTCCCGGAGTTTTAATACTAGCATTACTTCAGCCGATCTTTCGTCAGAA
GACCATATATCTGCGATGAAATGCACATTCGTGGTCGCTTGCCAGTTATATGGGTCTCAAAAGGCAGAGA
AAGATACCCGTGCAGAGGAAATACTGTACCTGATGAAAAGTAATGAAGCGCTCCGTGTAGCTTATGTGGA

TGAAGTCCCCACCAGGAGGGCTGAAAAGGAATACTACTCTGTTCTGGTCAAGTACGATGGGCAACGCCAG
AAGGAAGTAGAACTATACAGGGTCCAATTGCCTGGTCCTTTGAAGCTTGGAGAGGGAAAATCGGAAAACC
AGAACCATGCGCTCATTTTCACTCGTGGCGATGCCGTCCAGACTATCGATATGAACCAAGACAACTATTT
CGAGGAGGCACTCAAGATGCGGAATCTACTGGAAGAATTCAAGACTTTCTATGGCATACGGAAGCCTACC
GTCTTGGGGGTTAGAGAACACATCTTCACTGGTTCAGTTTCGTCGCTTGCTTGGTTTATGTCAGCTCAAG
AGATGAGTTTAGTCACGTTGGGGCAACGCGTCTTGGCTAATCCTTTGAAAGTTCGAATGAATTACAGCCA
TTCAGATGTTTTCGACAGGTTTTGGTTCTTAAGCAGGGGTGGTATTAGCAGAGCATCCAGAATAATCAAC
ATTAGTGAAGATATTTCCGCCGGATTTAATTGCACATTGAGAGGAGGCAAGGTCACACACCATGAGTACA
TACAAGTGGGAAAGGGAAGGATGTTGGATTGAATCAGATATCGATGGTTGAAGCCAAGGTTGCTGCTGG
AAATGGTGAACAGGTCCTTAGCAGAGATGTCTATCGGTTGGGGCATCGGCTTGACTTCTTCCGGATGCTA
TCCTTCTTTTACACCACTGTGGGATACTTCTTTAACACAACGATGGTCATCCTGACAGCCTATGCATTTT
TATGGGGCCGGCTCTATCTTTGCCCCGCTGCAAGCAGCAGCAACGACAATAAAGCACTCGGTACAATTCT
AAATCTATTTGTCATCCAACTCTGTCTATTCACGGCAATCCCTATGATAGTGGAGAGTACCCTTCAGCAT
GGATTTCTTCAAGCTATGTGGGATTTCATGACCAAGCAGCTCCAGTTATCATCTGTTTTCTACACGTTTT
CTATGGGAACTCGTGCCCACTACTTCGGCCGGACCGTTCTTCATGGTAGTGCGAAATATCGTCCCACAGG
ACATGGGTTTGTCGTGGACCACAAAAGTTTCGCAGAGAACTACAGGCTCTATGCACGTAGTCACTTTGTG
AAGGCGATTGAACTCGGCCTATTACTCACATTCTATGCGTCACGCAGCCATGCTGACAAAAATACCTTCA
CTTACAAAGCCTTGACCATATCAAGTTGGTTCTTTGTTGTTTCATGGATAATGGCTCCTTTTCTGTTCAA
TCCTCTGGGTTTCGATTGGTTGAAGACGGTGCATGACTTCGGCGATTTCATGAACTGGATATGGTTCCGT
GCTGGTGTGTTTACTAAAGCTGAAGAGAGTTGGGAAGTCTGGTGGTATGAGGAGCAGGATCATCTAAGAA
CAACAGGTCTTTGGGGAAAACTGGTAGAGGTAATATTGGATCTCCGCTTCTTCCTTTTCCAGTATGGGGT
TGTATACCAACTCGGAATCCCAGCTGGAAGTAGAAGTATTTCTGTTTACTTGCTCTCTTTGATTTGCTTT
TTCGTAATTCTGGGGGTCCATGCGCTTATATCATATGCCTGGGACAAGTACGCGGCAAGAGAACACATGT
ACTATCGACTCGTGCAGTTCCTCATTATCATACTAGGAGCACTCGCGTTAGTTGCATTGGTGGAGTATCG
GCAGCTCAAAGTTAAAGAGATTTTCACCAGTTTGTTGGCGTTCATCCCCACCGGATGGGGCCTCATATCG
ATAGCGCAAGTTCTTCGACCGATTCTGAAGCCCACTTGGATCTGGGGCAGGGTTGTTTCTTTAGCTCGTC
TTTATGATATAATGTTCGGAGTGATTGTCATGGCTCCGATAGCTGTGTTTTCTTGGATGCCGGGGTCTAT
GCAGACACGGATTCTATTTAACCAAGCATTTAGCGAAGGACTCTGGATTTCACGGATTGTCTCCGCAAAA
AGGCCGAAGGTTCACTTATGA
>XM_010099604.2_*Morus_notabilis*
ATGAGCCTCCGCCAGCGACCGCCCCCACCTTCGCGTCCGGGCCCGGCGGCGGCGGCGGGAGATCC GGAGT
CGGAGCCGTACAACATAATCCCGGTGCACAACCTGCTCGCGGACCACCCGTCGCTGCGGTACCCGGAGGT
TCGAGCCGCGGCGGCGGCGCTGCGAGCCGTCGGGAACCTCCGTCGGCCGCCGTTCGCGCAATGGCTCCCT
CACATGGACCTCCTCGATTGGCTCGCACTATTCTTCGGCTTCCAAAACGATAACGTTCGGAACCAGAGGG
AGCACCTCGTCCTCCACCTCGCCAACGCCCAGATGCGCCTCACGCCGCCGCCGGACAACATCGACACACT
CGACGTCAGCGTCCTCCGCCGCTTCCGCAAGAAGCTGTTGAAGAACTACACCGATTGGTGCTACTATTTG
GGGAAGAAGTCCAACATTTGGATCTCCGACCGCCGCGAGGCGTCGTCGGACCAGCGCCGCGAGCTCCTCT
ACGTCTCGCTTTATCTCCTGATTTGGGGTGAGTCTGCTAATCTCCGATTTGTTCCAGAGTGTATTTGCTA
TATATTTCATAATATGGCCATGGAATTGAACAAGATTTTGGAGGATTACATTGATGAGAACACGGGGCAG
CCGGTTATGCCCTCGGTCTCCGGCGAGAACGCGTTCTTGAACTGCGTTGTAAAGCCGATTTACGAGACGA
TTAGGGCCGAGGTTGAGAGTAGTAGGAACGGGACAGCCCCGCACAGTGTGTGGAGGAATTATGACGATAT
AAACGAGTATTTTTGGAGTAAAAGGTGTTTCGATAAGTTGAAATGGCCTGTTGATGTTGGGAGCAATTTC
TTTGTGACTTCTAGTAGGAGTAGGCATGTGGGTAAGACCGGGTTTGTGGAGCAGAGGTCGTTTTGGAACT
TGTTTAGAAGCTTTGATAGGCTTTGGATCATGCTCATTTTGTTCCTTCAGGCGGCCATTATTGTTGCCTG
GGAACAGGACGAGTATCCTTGGCATTCGCTGCGGGATCGGGGCGTTCAGGTCCGGGTTCTCACCGTATTC
TTCACATGGAGTGCGTTGAGGTTCTTGCAGTCGTTGCTCGACGCGGGGATGCAGTACAGTTTGGTTTCGA
GGGAGACTTTAAGGCTCGGGGTGAGGATGGTGCTGAAGAGTGCTGTTGCTGCAGGGTGGATTGTGGTTTT
TGGGGTGTTCTATGCGAGGATTTGGACACAGAGGAATAATGATAGGAGGTGGTCGGCCGAGGCGAATCGG
AGGGTGGTGACTTTTCTTCAGGTGGCATTGGTTTTTGTTCTCCCGGAGATTCTAGCACTGGCTCTTT TTA
TTCTCCCCTGGATTCGAAATTTTATTGAGGGTACAAATTGGAGGATCTTTCGCATGATGTCGTGGTGGTT
TCAGGGCAGAATCTTTGTTGGTCGCGGGTTGAGGGAAGGTCTTGTGGACAATATAAAGTACACTTTGTTT
TGGATTGTGGTGCTGGCTACAAAGTTTTGCTTCAGTTACTTCATGCAGATCAAACCCATGATTGCCCCAT
CAAAAGCACTGTTGAGAATTAAGAACTTGGATTACGAGTGGCATGAGTTTTTTGAAAGCAGCAATAGATT
TTCTGTTGGGTTACTATGGCTTCCGGTTGTTTTGATTTACCTCATGGATTTGCAGATTTGGTATTCTATC

TATTCCTCTTTTGTGGGGGCAGCAGTAGGGTTGTTTTCACACTTGGGCGAGATCCGGAATCTGCAACAAT
TGAGATTAAGGTTCCAATTCTTTGCAAGTGCAATTCAGTTCAATCTCATGCCAGAGGAGCAGCTGTTGAA
TGCAAGGGGGACGCTAAGGAATAAGTTTAAAGATGCTATTCACCGGTTGAAGCTGAGATATGGGTTTGGT
CAGCCATACAGGAAGCTCGAATCTAATCAGGTAGAGGCAAACAAGTTTGCTTTGATATGGAATGAGATAA
TAATGACTTTCAGGGAAGAAGATATTATCTCTGACCGAGAACTTGAGTTGTTGGAGCTGCCCCAGAACTC
TTGGAATGTCAGGGTAATTCGCTGGCCTTGTTTCCTTCTCTGCAATGAGTTGCTGCTTGCGCTCAGCCAG
GGCAAGGAGTTGGTGGATGCTTCTGATAAGTGGCTTTGGTATAAGATTTGCAAGAATGAATATAGGCGTT
GTGCTGTGATAGAAGCTTATGATTGTACTAAGCACTTGATTCTTCAGATTATCAAACGTAACAGCGAAGA
GCATTCCATTGTCACGGTTCTATTTCAAGAGATTGATCACTCTCTACAGATTGAGAGGTTCACGAAAACT
TTTAAAACGACTGCACTTCCCACACTCCACTCGAAGTTGATCAAACTTGTTGAACTGTTGAACAAGCCTA
ACAAAGATGCTAGCCAGGTGGTAAATACTCTACAGGCCCTCTATGAGATTGTTATTCGAGATTTTTTCAG
GGACAAGAGGAGCATTGAACAGTTAAAGGAGGAGGGTTTGGCTCCTCAAAATCTGGCTTCTACAGCAGGG
CTTCTGTTTGAGAATTCTGTTCAGTTTCCGGATCCTGATGATGAAGCCTTCTATCGGCAGGTGCGGCGCC
TGCACACCATACTCACTTCTCGGGATTCAATGCACAATATCCCGGTTAACCTTGAGGCAAGACGCAGAAT
TGCTTTCTTTAGTAACTCCCTCTTTATGAACATGCCCCATGCTCCCCAAGTTGAGAAAATGATGGCTTTC
AGTGTTCTGACCCCTTACTACAGCGAAGAAGTTTTGTACAACAAAGAGCAACTCAGGACTGAGAATGAGG
ATGGGATTTCGACCCTGTATTATTTGCAGACAATTTATAATGATGAGTGGAAAAATTTTATGGAGAGGAT
GCGCCGAGAAGGAATAGTAGATGATAAGGAAATATGGACAACGAAGTTGAGAGATCTCAGGCTCTGGGCA
TCGTACAGAGGCCAAACACTTTCACGAACTGTGAGGGGAATGATGTATTACTATCGAGCTCTTAAGATGC
TGGCATTTCTAGACTCTGCTTCAGAGATGGACATTCGTGAAGGATCACGGGAGCTTGGTTCAATGAGGCG
AGACATCAGCCTGGACGGTTTCAACTCGGAGAGGTCTCCTTCATCTAAGAGTTTAAGTAGAACAAACAGT
TCGGTGAGTTTGTTATTCAAAGGCCATGAGTATGGGACTGCTTTAATGAAGTTTACATATGTGGTAGCGT
GCCAGATATATGGAACGCAGAAGGCAAAAAAAGACCCGCATGCTGAGGAAATTTTGTATCTAATGAAAAC
CAATGAGGCTCTTCGAGTTGCCTATGTTGATGAGGTGTCAACTGGCAGAGACGAGAAAGATTATTACTCT
GTTCTGGTGAAGTATGATCAGAAGTTAGATAAGGAAGTGGAAATCTACCGTGTAAAGTTGCCTGGTCCGT
TGAAGCTCGGAGAGGGAAAACCAGAGAATCAAAATCACGCCATTATCTTCACTCGTGGTGACGCAGTTCA
GACAATTGATATGAACCAGGATAACTACTTCGAGGAGGCCCTGAAAATGAGGAATCTATTGGAAGAATAC
AGGCGCTACTATGGTGTCCGAAAACCTACAATCTTGGGAGTCAGAGAACATGTTTTTACTGGTTCTGTTT
CATCACTTGCTTGGTTTATGTCGGCGCAAGAAACTAGCTTTGTCACCTTGGGTCAGCGTGTTTTGGCGAA
CCCTCTAAAAGTTCGAATGCATTATGGCCATCCTGATGTGTTTGACAGGTTTTGGTTCTTCACTCGAGGT
GGATTTAGTAAAGCTTCCAGGGTGATTAACATTAGTGAAGACATTTTTGCTGGCTTTAACTGCACGCTGC
GTGGAGGCAATGTTACTCACCATGAATATATTCAAGTTGGAAAGGGAAGAGATGTAGGGTTGAACCAAAT
TTCCATGTTTGAAGCCAAGGTTGCAAGTGGAAATGGGGAGCAAGTACTGAGCAGAGATGTCTACAGGCTG
GGTCATAGGCTGGATTTCTTTAGAATGCTGTCATTCTTTTACACTACAGTGGGATTCTTTTTAAACACAA
TGATGGTTATTCTGACTGTATATGCATTCTTGTGGGGCCGTCTGTATCTGGCTCTTAGTGGTATCGAGGG
TTCTGCTTTGTCGAACGATAGCAATAAAGCACTTAGCACAATCTTGAATCAGCAGTTCATCATCCAACTT
GGTCTCTTCACTGCCCTTCCAATGATAGTGGAGAACTCTCTTGAGCATGGTTTCCTGCAGGCTGTTTGGG
ATTTCTTGACAATGCAGCTCCAGCTTTCTTCTGTTTTCTACACATTCTCGATGGGAACTCGTACCCACTT
CTTTGGCCGAACTATTCTTCATGGTGGTGCAAAGTATCGTGCCACTGGACGTGGCTTTGTTGTTCAGCAC
AAGAGTTTTGCAGAAAATTATAGACTCTACGCTCGTAGTCATTTCATAAAAGCTATTGAACTTGGGCTGA
TACTTATAGTTTATGCATCGCATAGTGCTGTAGCCAAGGACACGTTTGTATACATAGCCTTGACCATCTC
TAGTTGGTTTCTTGTTGCCTCATGGATCATGGCTCCCTTTGTCTTTAATCCTTCCGGTTTCGATTGGTTG
AAGACTGTAGATGATTTTGATGACTTCATGAACTGGATTTGGTTTCGTGGTAGTGTGTTTGCGAAAGCTG
AGCAGAGCTGGGAAAGATGGTGGTATGAGGAGCAGGATCATTTGAGAACCACTGGCTTATGGGGAAAGTT
GCTGGAGGTAATTTTGGATCTCCGCTTTTTCTTTTTCCAATATGGTATTGTGTACCAGCTTGATATTGCA
TCTGGAAATAAAAGCATCATTGTATATTTGTTGTCTTGGATCTATGTCCTGGTGGCTTTTGGAATATATG
TGGTGATAGCATATGCTCGGGACAGATATGCAGCAAAAGAGCACATATACTACCGTCTGGTCCAGTTTCT
TGTCATTGTACTCGGGATACTTGTGATTATTGCATTGCTGAAGTTCACAAACTTCAATTTCATGGACATT
TTCACTAGTCTATTGCCATTTATCCCCACTGGGTGGGGCATGATATTGATTTGCCAAGTACTTCGCCCGT
TTCTGCAGTCTACGATACTTTGGGAATTGGTCGTCTCTGTGGCTCGATTGTACGATATAGTATTTGGTGT
GATTATCTTGGTTCCTGTGGCTCTTCTATCATGGCTTCCTGGGTTTCAGTCTATGCAGACTAGGATCCTG
TTCAATGAAGCATTCAGCAGGGGCCTCCGGATATTCCAGATTGTTACGGGCAAAAAATCTAAGGTTGACA
TGTAA
>XM_006376431.2_*Populus_trichocarpa*

ATGTCTCTCCGTCGCCCGCCCCCTCCTAATCCGGCCCGAACCGAATCCCAACCCTACAACATAATCCCCA
TCCAGAACCTGCTTGCCGACCACCCTTCCCTCCGCTACCCCGAAGTTCGAGCAGCCGCCGCCGCCTTACG
CACCGTCGGCAACCTCCGTAAACCACCCTACGCTCAATGGCACCCTTCAATGGACCTCCTCGACTGGCTT
GCTCTGCTCTTCGGCTTCCAAAAGGACAACGTTCGCAACCAGCGGGAGCACCTAGTCCTTCACCTTGCAA
ACGCTCAGATGCGGCTGACTCCTCCGCCGGACAACATTGATACCCTAGACGCCGGTGTACTCCGTCGGTT
CAGGCGGAAACTGCTGAAGAATTATACAAATTGGTGTGATTACTTGAATAAGAAGTCGAATATCTGGATC
TCTGACCGGTCGACGGATCTGAGAAGGGAGTTACTGTATGTCTCGTTGTATTTGTTAATTTGGGGGGAAT
CGGCGAATTTACGGTTTATGCCGGAGTGTATTTGCTTTATATTTCATAATATGTGTTTTGAATTGAATAG
AGTTTTAGAGGATTATATTGATGAGAATACAGGGCAGCCGGTGATGCCGTCAATTTCAGGGGAGAATGCG
TTTTTGAATGGTGTTGTGAAGCCAATTTATGAGACGGTGAGAAGGGAAGTGGATAGGAGCTTTAACGGGG
CTGCGCCACATAGTGCTTGGCGGAATTATGATGATTTGAATGAGTATTTTTGGAGTAAGAGGTGTTTTGA
GAGGTTGAAATGGCCGATTGATTTAGGGAGTAATTTTTTTGTGACTTCGGGATCGAGGAAGAAGGTGGGG
AAGACGGGGTTTGTGGAGCAACGGTCGTTTTGGAATATTGTAAGGAGTTTTGATAGGTTGTGGGTGATGT
TGATTTTGTTTTTGCAAGCGGGGATTATTGTTGCGTGGGAGGAGAAGGAGTATCCGTGGAAAGCGTTGAA
GAGCAGGGATGTGCAGGTTAGGGTGTTGACTGTTTTTTTTACTTGGAGTGGGTTGAGGTTCTTGCAGTCA
TTGCTTGATGTTGGGACACAGTATAATTTGGTTTCGAGGGAGACTTTGGGGCTTGGGGTTAGGATGATTT
TGAAAAGTGTGGTTGCAGTAGGGTGGATTATTGTGTTTGGTGCATTTTATGGGAGGATTTGGAGTCAGAG
GAACAGTGATCTGAGGAGGTCGCCCAGGGATCTGAGTTGGTCATCGGAGGCTGATAGGAAGGTAGTGACT
TTTCTTGAGGTTGCTTTGGTGTTTGTGGCGCCAGAGATATTGGCATTGGCTCTGTTTATTCTTCCTTGGA
TTAGGAATTTTCTTGAGAATACGGATTGGAGGATATTTCGGATGATGACATGGTGGTTTCAGAGTAGTAG
TTTTATTGGTAGAGGGTTGAGGGAGGGGCTTGTGGATAATATTAAATATACTTTGTTTTGGGCTATGGTT
TTAGCTACCAAATTTGCTTTCAGTTACTTTATGCAGATTAAACCCATGGTTAAACCATCAAAACAGATGC
TGAAGCTGAAGGATGTGAATTATGAATGGCACGAGTTTTTTGACCACAGCAATAGGTTTTCAGTTGGGTT
GCTGTGGCTTCCTGTGGTGTTGATTTACTTGATGGACTTGCAGATTTGGTATGCCATTTATTCCTCTTTT
GTTGGAGCAGGAGTAGGGTTGTTTCAACATTTGGGTGAGATTCGAAACATCCAGCAATTAAGGTTGAGAT
TTCAGTTCTTTGCAAGTGCAATTCAGTTTAATCTGATGCCAGAGGAGCAGCTGTTGAATGCAAGGGGTAC
GATCAAGAGCAAGTTCAAAGATGCCATTCACAGGTTGAAGCTTAGGTATGGGTTTGGCCACCCTTACAAG
AAGCTGGAGTCTAACCAGGTTGAGGCGAACAAGTTTGCTTTGATATGGAATGAGATCATAATAATTTTCA
GGGAGGAGGATATTATCTCTGACAAGGAGCTTGAGTTGATGGAGTTGCCACAGAATTCTTGGAATGTGAG
GGTGATTCGCTGGCCAAGTTTTCTCCTGTGCAATGAGCTGCTGCTTGCTCTTAGCCAGGCCAAAGAGTTG
GTAGATGCTCCTGATAAGTGGCTCTGGTACAAGATATGCAAGAACGAGTATAGGCGCTGTGCGGTCATGG
AAGCTTATGATAGTGTCAAGCACCTGTTACTTGAAATCATCAAGACCAACACAGAAGAGCACTCAATTAT
CACGGTTTTGTTTCAAGAAATTGATCACTCTCTGCAGATTGAGAAATTCACCAAGACTTTCAAGATGACA
GCTCTGCCTAATTTCCATGCCAAGCTGATAAAGCTTCTTGAGCTGTTGAACAAGCCTAAGCGGNNATCTG
AACCAGTGGTAGATACTCTACAGGCTCTATATGAGATTGCTGTACGAGAATTTTTCAGAGAAAAGAGGAG
CACTGAACAGTTGATGGAGGGACGGTTGGCTCCCTCCGTGAACCCAGCTGGCCATGGGCTTGGCCTTCTT
TTTNNNNNNNNNNNNNNNNNNNNNNNNNCGTAAACCATCATCTCTTTGTTAACTCATTAAGTAGTCTTCATA
GGGATTCGATGAACACTATCCCAGAAAATCTAGAGGCCAGGCGCAGAATTGCATTTTTCAGCAATTCCCT
GTTCATGAGCATGCCCCACGCTCCCCAGGTTGAGAAAATGATGGCTTTTAGTGTGCTGACCCCTTATTAC
AATGAGGAGGTGCTATACAGCAGAGAACAGCTTCGAACTGAAAATGAAGATGGGGTTTCCACACTGTACT
ACCTGCAAACTATTTATGCTGATGAGTGGAAAAATTTCATGCAGAGGATGCGCCGTGAAGGAATGGAAAA
GGATGGTGAGATATGGACAACCAAGTTGAGAGATCTTAGGCTCTGGGCATCTTATAGAGGCCAGACGCTT
GGCCGTACTGTGAGGGGAATGATGTATTATTACCGTGCTCTTAAGATGCTGGCTTTTCTTGATTCTGCCT
CGGAGATGGACATTAAAGAGGGTTCACGAGAACTTGGCTCGATGAGGCGAGACAATGGTTTGGATAGCTT
TGACTCAGAAAGTTCTCCTTCTAAGAGCTTGAGCAGAAATAGCAGTTCAGTGAATTTGTTGTTTAAAGGT
CATGAGTATGGGACTGCTTTGATGAAATACACATATGTGGTTGCCTGCCAGATATACGGGGCACAAAAGG
CAAAGAAGGATCCCCATGCTGAGGAAATCTTGTATCTGATGAAAAATAATGAGGCCCTTCGCGTTGCCTA
TGTTGATGAAGTAAACACTGGGAGGGATGAGATGGAATATTATTCTGTACTTGTGAAGTATGATCAGCAG
TTGGACAAGGAAGTGGAAATCTACAGGGTGAAGTTGCCGGGTCCCTTGAAACTCGGTGAGGGAAAACCAG
AGAATCAAAATCATGCCCTAATCTTCACTCGTGGGGATGCAGTGCAGACTATTGATATGAACCAGGATAA
CTATTTTGAAGAGGCTCTCAAAATGCGGAATCTTTTGGAAGAATACAGGCACTACTATGGAGCTCGTAAA
CCTACTATCTTGGGAGTCAGGGAACACATTTTTACTGGTTCTGTATCATCTCTTGCATGGTTTATGTCTG
CTCAGGAAACTAGTTTTGTCACCCTGGGTCAGCGTGTTTTGGCAAACCCTTTGAAAATTCGAATGCATTA
TGGCCATCCAGATGTCTTTGACAGGTTTTGGTTCATGACTAGAGGTGGAATCAGCAAGGCTTCCAGAGTG

ATTAACATTAGTGAGGACATATTTGCTGGCTTTAATTGCACCTTGAGAGGAGGCAATATTACTCACCACG
AATACATCCAAGTTGGAAAAGGAAGGGATGTTGGGTTGAATCAAATATCCATGTTTGAAGCAAAAGTTGC
CAGTGGAAATGGCGAGCAAACTCTTAGCAGAGATGTCTATAGATTGGGCCATAGGCTGGACTTCTTCCGT
ATGCTGTCATTCTTTTATACTACGGTGGGATTTTTTTTGAACACTATGATGGTCATTCTGACTGTGTATG
CATTTCTGTGGGGCCGTCTTTATCTGGCTCTTAGCGGTGTTGAGGGTTCTGCTTTGGCCGACAACAGCAG
TAACAATAAGGCACTTGGTGCTATTTTGAATCAGCAATTCATCATCCAACTTGGCCTCTTCACTGCCCTT
CCGATGATAGTGGAGAACTCTCTTGAACACGGGTTTCTCGAAGCTATCTGGGATTTCTTGACAATGCAGC
TCCAGCTCTCATCTGTTTTCTACACATTCTCTATGGGAACTCGGACGCACTACTTTGGCCGTACCATCCT
CCATGGTGGCGCAAAATATCGGGCAACTGGGCGTGGTTTTGTTGTGCAGCACAAGAGTTTTGCAGAGAAT
TACAGGCTCTATGCTCGTAGCCATTTTGTGAAGGCAATTGAGCTTGGACTGATACTTGTAGTTTATGCAG
CATACAGCCCTGTAGCTAAGGACACATTTGTTTATATAGCAATGACCATCTCTAGTTGGTTCCTGGTTGT
GTCGTGGATAATGGCCCCGTTTGTCTTCAATCCATCTGGCTTTGATTGGTTGAAGACAGTATACGACTTT
GATGATTTTATGAACTGGATTTGGTACCAAGGTGGTGTGTTTGCAAAATCTGAACAGAGCTGGGAAAGAT
GGTGGTATGAGGAGCAGGATCATCTGAGGACAACTGGGCTTTGGGGAAAGTTACTGGATGTAATATTGGA
CCTTCGCTTCTTCTTTTTTTCAATATGGGATCGTATACCAACTAGGTATTGCTGCTGGGAGTACTAGCATT
GCTGTTTACTTGCTTTCTTGGATTTATGTAGTTGTCGCCTTTGGGTTTTTTTTGATGGTAGCATATGCCC
GGAACAAGTATGCTGCAAAAGAACACATCTACTATCGGATGGTCCAGTTTCTGATCATTGTGCTTGGCAT
CTTTGTGATTATAGCCCTGCTTCAGTTCACATCTTTCAAATTTACTGATGTTTTCACAAGTTTGTTGGCT
TTTATCCCCACTGGATGGGGCATTTTATTGATTGCCCAAGTACTTCGCCCCTTCCTGCCCGCTATACTTT
GGGAAGCAGTGGTTTCTGTGGCTCGGCTATATGATATATTGTTTGGGGTGATAGTTATGGTCCCTGTGGC
ATTTTTGTCATGGATGCCTGGGTTCCAATCAATGCAGACTAGGATCCTCTTCAACGAGGCATTCAGCAGG
GGCCTCCGGATCTTCCAGCTTTTCACGGAAAAAAATCGTAG
>XM_024190232.1_*Citrus_clementina*
ATGAGTAATTTGCGTCACCGAGCGGGTGCGGGCCAGTCCAGGCCGGACCGCCTTCCCGAAGAAGAAGAGG
AGCCCTACAACATTATCCCAGTTCACAACCTCTTAGCCGACCACCCTTCTCTCCGCTACCCAGAAGTCCG
CGCCGCCGCCGCCGCCTTACGCACTGTTGGAAACCTCCGCAAGCCGCCGTATGTCCAATGGTTGCCCCAC
ATGGATCTCCTTGATTGGCTTCAGCTCTTTTTCGGTTTTCAACTTGACAACGTTCGGAACGAACGGGAGC
ATCTCGTTCTTCATTTGGCTAACGCCCAGATGCGGCTCACGCCGCCGCCGGATAACATTGACACCCTCGA
CGCTGGTGTTTTGAGGAGGTTTAGGCGGAAGTTGTTGAAGAATTATACACTCTGGTGTTCCTATTTGGGG
AAGAAATCGAACATTTGGCTTTCGGATCGGAGCTCCGACCAGCGCCGTGAACTGCTGTATGTTTCTCTGT
ATCTTTTAATTTGGGGCGAGGCGGCGAATCTCCGGTTCATGCCGGAATGTTTGTGCTACATTTTCCATAA
CATGGCCATGGAGTTGAATAAGATTTTAGAAGATTACATTGATGAGAACACGGGGCAGCCAGTGATGCCT
TCGATTTCGGGCGAAAACGCTTTTCTGAACTGCGTCGTGAAGCCCATTTACGAAACGGTGAAAGCTGAGG
TGGAGAGTAGTAAGAATGGATCGGCGCCGCATTATGCGTGGAGGAACTACGATGATATTAACGAGTACTT
TTGGAGTAAGAGGTGTTTTCAGAAGCTGAAATGGCCGATTGATGTTGGGAGCAATTTCTTTGTGTTGTCG
GGTAAGACTAAGCACGTGGGGAAAACTGGGTTTGTCGAGCAACGATCGTTTTGGAACTTGTTTAGGAGTT
TTGATAGGTTGTGGGTGATGTTGATATTGTTCATTCAAGCTGCAGTTATCGTGGCCTGGGAGGAGAGAGA
GTATCCGTGGCAAGCATTGGAGGAGAGAGATGTTCAGGTTCGTGCTTTGACTGTGGTTCTTACTTGGAGT
GGATTAAGGTTTTTGCAAGCTTTGTTGGATTTCGCAATGCAACGTAGACTTGTCTCTAGGGAAACAAAGT
TACTTGGTATGAGGATGGTGTTGAAGGGTGTTGTGTCGGCCATATGGATAACTGTTTTTGGAGTGCTTTA
TGCTAGGATTTGGATGCAGAGAAATAGTGATCGCAGATGGTCTAATGAGGCGAATAACCGTTTGGTGGTT
TTTCTTCGGGCAGTGTTTGTGTTTGTCTTGCCTGAATTATTGGCTATAGCTTTGTTTATAATTCCTTGGA
TTAGGAATTTTCTCGAGAATACAAATTGGAAGATCTTTTATGCATTGACATGGTGGTTTCAAAGTAGAAG
TTTTGTTGGTCGTGGATTGAGAGAAGGGCTTGTTGACAATTTGAAGTATAGTTTGTTCTGGGTGCTTGTT
TTGGCTACAAAATTTGTATTCAGTTACTTCTTGCAGATTAAACCCATGATTGCCCCAACCAAACAATTGT
TAAAACTTAAAAATGTGGAGTATGAGTGGTATCAAGTGTTCGGTCATGGAAACAGATTGGCTGTTGGGTT
ACTGTGGGTCCCCGTGGTTTTGATTTATTTGATGGATTTGCAGCTCTTTTACTCTATTTATTCATCTTTG
GTGGGTGCAGCAGTGGGGTTGTTCCAGCACTTGGGTGAGATCCGAAATATGCAGCAGCTGAGGCTTAGAT
TTCAGTTCTTTGCGAGTGCGATGCAGTTTAATCTAATGCCTGAGGAGCAGCTGTTGGATGCGAGGGGAAC
ACTTAAGAGTAAATTTAGAGATGCCATTCATCGGCTGAAACTGAGATATGGGCTAGGAAGACCCTATAAG
AAGCTTGAATCTAACCAGGTTGAGGCAAACAGGTTTGCTCTGATATGGAATGAGATAATTGCCACTTTCA
GGGAAGAGGATATCATATCTGATAAGGAGGTGAGCTGTGAGCTGCGGCAGATACTGATGTAAGGGTATCG
TTGGCCTNNGTTTCTGCTATGCAATGAGTGCTCTAGCTCTCAGCAAGCCAAGGAGCTGGTGGATGCTNCT
GATAAGTGGCTTTGGTATNAGATCTGCAAGAATGAGTATCGGCGTTGCGCTGTGATTGAAGCTTATGATA

GCATAAAGCACTTGATTCTCCATATCATCAAAGTTAACACCGAAGAGCATTCAATTATTACGGTCCTGTT
TCAAGAAATTGATCATTCGCTTCAGATTGAGAAGTTTACCAGAACATTCAAGATGACCGTGCTGCCCAGG
ATTCATACCCAGTTGATAAAACTAGTTGATCTGTTGAACAAGCCCAAGAAAGATCTTAACAAGGTGGTAA
ACACCCTACAGGCCCTTTATGAGACTGCTATTCGAGATTTTTTCTCAGAGAAGAGAAGCTCTGAGCAGCT
TGTGGAGGATGGTTTGGCCCCTCGTAATCCGGCTGCCATGGCAGGTTTGCTGTTTGAGACTGCTGTTGAG
TTACCTGATCCCAGTAACGAGAATTTCTATCGTCAGGTCAGGCGCTTAAACACAATTCTAACATCCAGGG
ATTCAATGAATAACATTCCTGTTAATCTTGAAGCAAGGCGCAGAATAGCCTTTTTTAGTAACTCTCTCTT
CATGATATTGCCACGAGTTCGCCAAGTTGAGAAGATGATGTCCTTCAGCGTTCTCACCCCCTACTACAAT
GAAGAAGTTGTTTATAGTAAAGAGCAACTTCGAACTGAAAATGAGGATGGGGTATCCATCCTGTATTATT
TGCAAACAATTTATGCTGATGAGTGGAAGAATTTCCTGGAGAGGATGCACCGAGAAGGAATGGTGAATGA
TAAAGAAATTTGGACAGAAAAGTTAAAAGATCTCAGGCTTTGGGCATCCTACAGAGGCCAGACACTTTCC
CGCACTGTTAGGGGAATGATGTATTATTATCGGGCTCTTAAGATGCTTGCTTTTCTTGATTCTGCTTCTG
AGATGGACATTAGGGAAGGGGCCAGGGAACTTGGTTCAATGAGGCAAGATGCCAGTTTGGATCGTATCAC
CTCAGAAAGGTCACCATCTTCCATGAGTTTAAGTAGAAATGGTAGTTCAGTGAGCATGCTGTTCAAGGGC
CATGAATATGGGACTGCTCTGATGAAATTCACATATGTTGTTGCCTGCCAGATATATGGCCAGCAAAAGG
ACAAGAAAGACCCCCATGCTGAGGAAATCCTGTATCTGATGAAGAACAATGAAGCCCTTCGAGTTGCCTA
TGTTGATGAAGTCTCTACAGGGAGGGATGAGAAGGATTATTTCTCTGTTCTTGTGAAATATGATAAGCAG
TTAGAGAAGGAAGTAGAAATCTACAGGGTCAAGTTGCCTGGTCCGTTGAAGCTTGGGGAGGGTAAACCGG
AGAACCAAAATCATGCTTTTATCTTCACCCGAGGGGATGCAGTCCAGACAATTGATATGAACCAAGACAA
CTACTTTGAGGAGGCACTTAAAATGCGGAATTTGTTGGAAGAATACAGACACTACTATGGCATACGGAAA
CCTACCATCCTTGGTGTCAGGGAGCACATTTTTACTGGTTCAGTGTCATCACTAGCTGGCTTTATGTCGG
CACAGGAAACCAGTTTTGTTACCTTGGGTCAGCGTGTTTTGGCTAACCCTTTGAAAATCCGAATGCATTA
TGGCCATCCTGATGTCTTTGACAGGTTTTGGTTCTTGACTCGTGGAGGCCTCAGTAAAGCTTCCAGAGTG
ATTAACATCAGTGAAGACATTTTTGCTGGCTTTAACTGCACACTGCGAGGAGGCAATGTCACACACCACG
AATACATCCAAGTTGGCAAGGGAAGGGATGTTGGATTGAATCAAATATCCATGTTTGAAGCCAAGGTTGC
TAGTGGAAATGGTGAGCAAGTTCTCAGTAGAGATGTCTACAGGTTGGGTCATAGACTGGACTTCTTTCGG
ATGTTATCATTCTTTTACACTACTGTGGGATTCTTTTTCAACACTATGGTGATCATTTTGACTGTATATG
CATTCTTATGGGGTCGATTTTATCTGGCTCTTAGTGGTATAGAGGATGCTGTGGCAAGCAACAGTAACAA
CAATAAAGCACTTGGTACTATCTTAAATCAGCAGTTCATTATCCAACTTGGTTTGTTCACGGCCCTACCT
ATGATTGTGGAGAATTCTCTTGAGCATGGATTTCTTCAAGCTATATGGGATTTCCTGACTATGCTGCTTC
AGCTTTCATCTGTTTTCTACACATTCTCCATGGGAACTCGCAGTCACTACTTTGGCCGGACTATCCTTCA
CGGTGGTGCTAAGTATCGGGCAACTGGCCGTGGTTTTGTTGTGCAGCACAAGAGCTTTGCAGAAAATTAT
AGGCTTTATGCACGTAGCCACTTTATAAAGGCCATTGAACTTGGATTGATTCTTACAATTTATGCATCGC
ATAGCGCTATAACTAAGGGCACATTTGTTTACATAGCCATGACCATTTCTAGTTGGTTTCTGGTTATGTC
ATGGATAATGGCTCCATTTGCTTTCAATCCTTCTGGCTTTGATTGGTTGAAGACTGTATATGACTTTGAG
GATTTCATGAACTGGATCTGGTTCCGTGGTAGTGTGTTTGCAAAAGCTGAACAGAGCTGGGAAAAATGGT
GGTATGAGGAGCAGGATCATCTAAAGACAACTGGCATTTTGGGGAAGATAATGGAAATAATTCTAGACCT
CCGCTTCTTCATTTTTCAGTATGGGATAGTATACCAGCTAGGTATTTCAGCTGGAAGTACTAGTATTGTC
GTTTACTTGCTTTCTTGGATCTATGTTGTTATGGCTTTTGGGATTTATGCAATTGTATCATATGCTCGGG
ACAAATATGCAGCCATAGAACACATCTACTACCGTCTGGTTCAGTTCCTCATTGTTATTTTTATGATACT
TGTGATAGTTGCCCTTCTGGAGTTCACAAAATTCAGGTTGATGGATCTTTTAACTAGTCTGATGGCTTTT
ATCCCTACAGGGTGGGGTCTGATATTGATTGCCCAAGTATTCCGGCCCTTTTTGCAGTCCACTAGGCTTT
GGCAGCCCGTGGTCTCTGTGGCTAGATTGTATGATATAATGTTTGGAGTGATTGTCTTGACCCCCGTGGC
ATTCTTATCTTGGATGCCTGGATTCCAGTCTATGCAAACAAGGATACTGTTCAATGAAGCATTCAGCAGA
GGCCTCCGAATATTCCAGATTGTTACTGGGAAAAAAGCTAAGGGCGACATGTAA
>XM_035060727.1_*Populus_alba*
ATGTCTCTCCGTCGCCCGCCCCCTCCTAACCCGAACCGACCCGAATCCCAACCCTACAACATAATCCCCA
TCCAGAACCTCCTTGCCGACCACCCTTCCCTCCGCTACCCCGAAGTTCGAGCAGCCGCCGCCGCCTTACG
CACCGTCGGAAACCTCCGTAAACCACCCTACGCTCAATGGCACCCTTCAATGGACCTCCTCGACTGGCTT
GCTCTGCTCTTCGGCTTCCAAAAGGACAACGTTCGCAACCAGCGGGAGCACCTAGTCCTTCACCTTGCAA
ACGCTCAGATGCGGCTGACTCCTCCGCCGGACAACATTGATACCCTAGACGCCGGTGTACTCCGTCGGTT
CAGGCGGAAACTGCTGAAGAATTATACAAATTGGTGTGATTACTTGAATAAGAAGTCGAATATCTGGATC
TCTGACCGGTCGACGGATCTGAGAAGGGAGTTACTGTATGTCTCGTTGTATTTGTTAATTTGGGGGGAAT
CGGCGAATTTACGGTTTATGCCGGAGTGTATTTGCTTTATATTTCATAATATGTGTTTTGAATTGAATAG

AGTATTAGAGGATTATATTGATGAGAATACAGGGCAGCCGGTGATGCCGTCAATTTCAGGGGAGAATGCG
TTTTTGAATGGTGTTGTGAAGCCAATTTATGAGACGGTGAGGAGGGAAGTGGACAGGAGCTTTAACGGGG
CTGCGCCACATAGTGCTTGGCGGAATTATGATGATTTGAATGAGTATTTTTGGAGTAGGAGGTGTTTTGA
GAGGTTGAAATGGCCGATTGATTTAGGGAGTAATTTTTTTGTGACTTCGGGATCGAGGAAGAAGGTGGGG
AAGACGGGGTTTGTGGAGCAACGGTCGTTTTGGAATATCGTAAGGAGTTTTGATAGGCTGTGGGTGATGT
TGATTTTGTTTTTGCAAGCGGGGATTATTGTTGCGTGGGAGGAGAAGGAGTATCCGTGGAAAGCGTTGAA
GAGCAGGGATGTGCAGGTTAGGGTGTTGACTGTTTTTTTTACTTGGAGTGGGTTGAGGTTCTTGCAGTCA
TTGCTTGATGTCGGGACACAGTATAATTTGGTTTCGAGGGAGACTTTGGGGCTTGGGGTTAGGATGATTT
TGAAAAGTGTGGTTGCAGTAGGGTGGATTATTGTGTTTGGTGCATTTTATGGGAGGATTTGGAGTCAGAG
GAACAGTGATCTGAGGAGGTCGCCCAGGGATCTGAGTTGGTCATCGGAGGCTGATAAGAAGGTAGTGACT
TTTCTTGAGGTTGCTTTGGTGTTTGTGGCGCCAGAGATACTGGCATTGGCTCTGTTTATTCTTCCTTGGA
TTAGGAATTTTCTTGAGAATACGGATTGGAGGATATTTCGGATGTTGACATGGTGGTTTCAGAGTAGTAG
TTTTATTGGTAGAGGGTTGAGGGAGGGGCTTGTGGATAATATTAAATATACTTTGTTTTGGGCTATGGTT
TTAGCTACCAAATTTGCTTTCAGCTACTTTATGCAGATTAAACCCATGGTTAAACCATCAAAACAGATGC
TGAAGCTGAAGGATGTGAATTATGAATGGCACGAGTTTTTTGACCACAGCAATAGGTTTTCAGTTGGGTT
GCTGTGGCTTCCTGTGGTGTTGATTTACTTGATGGACTTGCAGATTTGGTATGCCATTTATTCCTCTTTT
GTTGGAGCAGGAGTGGGGTTGTTTCAACATTTGGGTGAGATTCGAAACATCCAGCAATTAAGGTTGAGAT
TTCAGTTCTTTGCAAGTGCAATTCAGTTTAATCTGATGCCAGAGGAGCAGCTGTTGAATGCAAGGGGGAC
GTTCAAGAGCAAGTTCAGAGATGCCATTCACAGGTTAAAGCTTAGGTATGGGTTTGGCCACCCTTACAAG
AAGCTGGAGTCTAACCAGGTCGAGGCAAACAAGTTTGCTTTGATATGGAATGAGATCATAATAATTTTCA
GGGAGGAGGATATTATCTCTGACAAGGAGCTTGAGTTGATGGAGTTGCCACAGAATTCTTGGAACGTGAG
GGTGATTCGCTGGCCAAGTTTTCTCCTGTGCAATGAGCTGCTGCTTGCTCTTAGCCAGGCCAAAGAGTTG
GTAGATGCTCCTGATAAGTGGCTCTGGTACAAGATATGCAAGAACGAGTATAGGCGCTGTGCAGTCATAG
AAGCTTATGATAGTGTCAAGCACCTGTTACTTGAAATCATCAAGACCAACACAGAAGAGCACTCAATTAT
CACGGTTTTGTTTCAAGAAATTGATCACTCTGTGCAGATTGAGAAATTCACCAAGACTTTCAAGATGACA
GCTTTGCCTAATTTCCATGCCAAGTTGATAAAGCTTCTTGAGCTGTTGAACAAGCCTAAGCGGGATCTGA
ACCAGGTGGTAGATACTCTACAGGCTCTATATGAGATTGCTGTACGAGAATTTTTCAGAGAGAAGAGGAG
CACTGAAGAGTTGATGGAGGACGGGTTGGCTCCTCGTGACCCAGCTGCCATGGCTGGGCTTCTTTTTGGG
AATGCAGTTCAGTTGCCTGATGCTAGCAATGAGACCTTTTATAGGCAGGCACGGCGTTTGCACATGATTC
TTACCTCTAGGGATTCGATGAACGCTATCCCAGAAAATCTAGAGGCCAGGCGCAGAATTGCATTTTTCAG
CAATTCCCTGTTCATGAACATGCCCCACGCTCCCCAGGTTGAGAAAATGATGGCTTTCAGTGTGCTGACC
CCTTATTACAATGAGGAGGTGCTATACAGCAGAGAACAGCTTCGAACTGAAAATGAAGATGGGGTTTCCA
CACTGTACTACCTGCAAACTATTTATGCTGATGAGTGGAAAAACTTCATGCAGAGGATGCGCCGTGAAGG
AATGGAAAAGGATGGTGAGATATGGACAACCAAGTTGAGAGATCTTAGGCTCTGGGCATCTTATAGAGGC
CAGACGCTTGGCCGTACTGTGAGGGGAATGATGTATTATTACCGTGCTCTTAAGATGCTGGCTTTTCTTG
ATTCTGCCTCGGAGATGGACATTAAAGAGGGTTCACGAGAACTTGGCTCAATGAGGCGAGACAATGGTTT
GGATAGCTTTGACTCAGAAAGTTCTCCTTCTCAGAGCTTGAGCAGAAATAGCAGTTCAGTGAATTTGTTG
TTTAAAGGTCATGAGTATGGGACTGCTTTGATGAAATACACGTATGTGGTTGCCTGCCAGATATACGGGG
CACAAAAGGCAAAGAAGGATCCCCATGCTGAGGAAATCTTGTATCTGATGAAAAATAATGAGGCCCTTCG
AGTTGCCTATGTTGATGAAGTAAACACTGGGAGGGATGAGATGGAATATTATTCTGTACTTGTGAAGTAT
GATCAGCAGCTGGACAAGGAAGTGGAAATCTACAGGGTGAAGTTGCCGGGTCCCTTGAAACTCGGTGAGG
GAAAACCAGAGAATCAAAATCATGCCCTAATCTTCACTCGTGGGGATGCAGTGCAGACTATTGATATGAA
CCAGGATAACTATTTTGAAGAGGCTCTCAAAATACGGAATCTTTTGGAAGAATACAGGCACTACTATGGA
GCTCGTAAACCTACTATCTTGGGAGTCAGGGAACACATTTTTACTGGTTCTGTCTCATCTCTTGCATGGT
TTATGTCTGCTCAGGAAACTAGTTTTGTCACCCTGGGTCAGCGTGTTTTGGCAAACCCTTTGAAAATTCG
AATGCATTATGGCCATCCAGATGTCTTTGACAGGTTTTGGTTCATGACTAGAGGTGGAATCAGCAAGGCT
TCCAGAGTGATTAACATTAGTGAGGACATATTTGCTGGCTTTAATTGCACCTTGAGAGGAGGCAATATTA
CTCACCACGAATACATCCAAGTTGGAAAAGGAAGGGATGTTGGGTTGAATCAAATATCCATGTTTGAAGC
AAAAGTTGCCAGTGGAAATGGCGAGCAAATTCTTAGCAGAGATGTCTATAGATTGGGCCATAGGCTGGAC
TTCTTCCGTATGCTGTCATTCTTTTATACTACGGTGGGATTTTTTTTGAACACTATGATGGTCATTCTGA
CTGTGTATGCATTTCTGTGGGGCCGTCTTTATCTGGCTCTTAGCGGTGTTGAGGGTTCTGCTTTGGCCGA
CAACAGCAGTAACAATAAGGCACTTGGTGCTATTTTGAATCAGCAATTCATCATCCAACTTGGCCTCTTC
ACTGCCCTTCCGATGATAGTGGAGAACTCTCTTGAGCATGGGTTTCTTGAAGCTATCTGGGATTTCTTGA
CGATGCAGCTCCAGCTCTCATCTGTTTTCTACACATTCTCTATGGGAACTCGGACGCACTACTTTGGCCG

TACCATCCTCCATGGTGGCGCAAAATATCGGGCAACTGGGCGTGGTTTTGTTGTGCAGCACAAGAGTTTT
GCAGAGAATTACAGGCTCTATGCTCGTAGCCATTTTGTGAAGGCAATTGAGCTTGGACTGATACTTGTAG
TTTATGCAGCATACAGCCCTGTAGCTAAGGACACATTTGTTTATATAGCAATGACCATCTCTAGTTGGTT
CCTGGTTGTGTCGTGGATAATGGCCCCGTTTGTCTTCAATCCATCTGGCTTTGATTGGTTGAAGACAGTA
TACGACTTTGATGATTTTATGAACTGGATTTGGTACCAAGGTGGTGTGTTTGCAAAATCTGAACAGAGCT
GGGAAAGATGGTGGAATGAGGAGCAGGATCATCTGAGGACAACTGGGCTTTGGGGAAAGTTACTGGATGT
AATATTGGACCTTCGCTTCTTCTTTTTTCAATATGGGATCGTATACCAACTAGGTATTGCTGCTGGAAGT
ACTAGCATTGCTGTTTACATGCTTTCTTGGATTTATGTAGTTGTCGCCTTTGGGTTTTTTTTGATGGTAG
CATATGCCCGGAACAAGTATGCTGCAAAAGAACACATCTACTATCGGATGGTCCAGTTTCTGATCATTGT
GCTTGGCATCTTTGTGATTATAGCCCTGCTTCAGTTTACATCTTTCAAATTTACTGATGTTTTCACAAGT
TTGTTGGCTTTTATCCCCACTGGATGGGGCATTTTATTGATTGCCCAAGTACTCCGCCCCTTCCTGCCCG
CTATACTTTGGGAAGCAGTGGTTTCTGTGGCTCGGCTATATGATATATTGTTTGGGGTGATAGTTATGGT
CCCTGTGGCATTTTTGTCATGGATGCCTGGGTTCCAATCAATGCAGACTAGGATCCTCTTCAACGAGGCA
TTCAGCAGGGGCCTCCGGATCTTCCAGCTTGTCACGGGAAAAAAATCGTAG
>XM_004243304.4_*Solanum_lycopersicum*
ATGAGCCTCCGGCAACGTTCAACGCCGGCGGCGAGACAAGTTTCTATAGATGAAGAACCATATAACATCA
TTCCGATTCATAATCTTCTAGCTGACCATCCTTCTCTACGTTTCCCTGAGGTACGCGCTGCGGCGGCGGC
TTTACGCTCTGTAGGTGACCTAAGGAGACCTCCATTTGCACCGTGGAAACCTCACTATGACCTGCTTGAC
TGGCTGGCGCTGTTCTTCGGGTTTCAGGATTCTAGTGTTCGTAACCAACGGGAACATATCGTGCTTCATC
TTGCTAATGCTCAGATGCGTTTATCTCCGCCGCCGGACAATATTGACTCTCTTGACCCTGCTGTTCTCCG
TCGGTTCCGACGTCAGCTTCTGAAGAATTACTCGTCGTGGTGCTCTTTTCTCGGTCTCAAATCTAATGTT
TGGCTTTCCGACCGGCATAACTCATCTGACCACCGCCGTGAGTTGCTTTATGTCTCGCTTTACCTTCTTA
TATGGGGTGAGTCAGCGAATCTACGTTTTGTTCCTGAATGTTTATGCTTTATTTTTCATAATATGGCTAT
GGAATTGAATAAGATTTTGGAGGATTACATTGATGAGAATACTGGTAGGCCATTTTTGCCATCGATATCT
GGTGAAAATGCTTTTCTGAATCGGATCGTAACGCCAATTTACCAAACAATCCGAGCTGAGGCTGATAATA
GTCGGAATGGTACTGCCCCACACTCTGCGTGGCGGAATTACGATGACATCAATGAGTATTTCTGGACTAA
AAGGTGTTTTGATAAGTTGAAGTGGCCTATTGATATTGGGAGTACATTTTTTGTGACCACTAACAAGGGA
AAGAAGGTTGGAAAGACAGGGTTTGTGGAGCAGAGATCATTTTTGAATTTGTATAGGAGTTTTGATAAGC
TATGGATCATGCTGGCGTTGTTTTTGCAGGCTGCAATTATTGTAGCTTGGGAAGGGAAGCCTTATCCGTG
GCAGGCTTTGGAGAGTAGGGAGGTTCAGGTGAGGGTGTTAACTATCTTCTTCACCTGGAGCAGTATGAGA
TTTCTGCAGTCGTTACTTGATGCAGGAATGCAATATCGTATCATCTCTAGGGAGACCCCGTGGCATGGGG
TGAGAATGGTGTTGAAGAGTGTGGTTGCAGCTGCGTGGATTGTGGTCTTTGGTGCATTCTATGGGAGGAT
TTGGATCCAGAGGAATAGGGATGGGAAATGGAGCAGTGCTGCTAACAGGAGGGTAGTGAATTTTCTTGAG
GTTGCTCTTGTTTTCATTGCTCCAGAACTGTTAGCCCTGGCACTCTTTGTTCTGCCATGGGTCAGGAATT
TTCTCGAGAACACGAACTGGAGGATATTTTACCTGTTGTCCTGGTGGTTCCAGAGTCGAACGTTTGTGGG
TCGTGGACTCAGGGAAGGCCTTGTTGATAACATTAAGTATTCCCTCTTTTGGGTAGTAGTGCTCGCGACC
AAGTTTTCCTTCAGTTACTTCCTACAGATCAAACCTATGATCGTTCCAACAAGAGCACTGTTGCGCCTCA
GGGATGTGAAGTACGAATGGCATGAATTCTTTAACCATAGCAACAGGTTCTCAGTAGGATTGCTTTGGCT
TCCTGTTGTACTGATTTATCTCATGGATATTCAGATATGGTACTCAATCTACTCTTCTTTTGTTGGGGCA
GCGGTTGGATTATTTGATCACTTGGGAGAGATTCGAAACATGCCGCAGTTAAGGTTGAGATTTCAATTTT
TTGCAAGTGCAATGCAGTTTAATCTGATGCCAGAAGAGCAGTTGTTGAATGCTCAAGGAACACTAAAAAG
CAAGTTCAAGGACGCCATCCTCCGTTTGAAACTCAGATATGGGTTTGGTCGACCATTCAAAAAGCTTGAA
TCAAACCAGGTAGAGGCGAACAAATTTGCCTTGATTTGGAATGAGATAATTACAACTTTCAGAGAAGAAG
ATATTCTGAATGACCGTGAGGTTGAGTTGTTGGAGCTGCCCCAGAACACATGGAATGTTAGAGTGATTCG
TTGGCCATGTTTGCTCCTCTGCAACGAGGTGCTGCTTGGTCTCAGCCAGGCGAAGGAGCTGGTGGATGCT
CCTGATAAGTGGCTCTGGCATAAGATCAGCAAGTATGAGTACAGACGATGTGCTGTTATTGAGGCTTATG
ACAGTACAAGGCATTTGCTGCTGGAAATTGTGAAATTGAACAGCGAGGAGCATTCCATCATAACAACCTT
TTTTCAGCAGATTGATCAGTGGATTCAGCTGGAGAAATTCACAAAATACTATAATCTGACTGCTCTGCCC
CAGATCCGTGGAAAGTTGATTGCTCTTCTGGATCTATTACTTAAGCCAAAAAAGGATGTTGACAAGATTG
TGAATGTTCTCCAGGCCTTATATGAGGTTGCCACTCGGGATTTTCTGAAAGAGAAGATGACTGGAGATCA
GCTGAGAGAGGAAGGTCTGGCTCTTCAGGCATCTGCAACTAGATTGCTTTTTGAGAATGTAGTTTCATTG
CCTGATCCAGAGAATGAGACATTTTATCGGCAAGCTCGCCGCTTGAACACTATTCTTACATCTCGGGACT
CTATGAGTAATATCCCAAGAAATCTTGAGGCGAGACGTCGACTTGCCTTCTTTAGCAATTCTCTATTTAT
GAATATGCCACATGCGCCCCAAGTTGAGAAGATGATGGCTTTCAGTGTTTTGACACCTTACTACAATGAA

GATGTACTGTACAACAAGGAACAACTCAGAACTGAGAATGAAGATGGGATTTCTACATTATATTACTTGC
AGACTATTTATGCTGATGAGTGGGAAAATTTCTTGCAGCGAATGCGTAGAGAAGGAATGGTTGATGAGAA
GAAAGAGTTATGGACTACAAAGCTAAGGGATCTTCGTCTTTGGGCATCATACAGAGGGCAGACTCTTACT
CGCACGGTTAGGGGGATGATGTACTACTATCGAGCTCTCAAAATGCTGGCCTTTCTGGATTCTGCTTGTG
AGATGGATATCAGAGAAGGATCAGTGGAACTTGGTTCTATGAGGCATGATGATAGCATTGGTGGTTTAAG
TTCAGAAAGATCTCAGTCTTCGAGGAGGTTGAGTAGAGCTGACAGTTCAGTGAGTATGTTGTTTAAAGGC
CACGAGTATGGGACTGCTTTAATGAAATTCACATATGTGGTAGCTTGTCAGATATATGGGGCTCAGAAGG
CCAAAAAAGATCCACATGCAGAGGAAATTTTGTATCTGATGAAAAATAATGAAGCTCTTCGTGTAGCTTA
TGTTGATGAGGTTCCCACAGGAAGGGATGAGAAGGATTATTATTCTGTGCTTGTGAAGTATGATCAAAAA
CTTGAAAGGGAAGTTGAGATCTATCGAGTTAAGTTGCCTGGTCCTTTGAAGCTTGGGGAGGGGAAACCAG
AAAATCAAAATCATGCCTTTATCTTTACCCGTGGTGATGCAGTTCAGACTATTGACATGAACCAAGATAA
TTACTTTGAGGAGGCACTGAAAATGAGGAACTTGTTGGAAGAATTCAAACTCTACTATGGTATTCGCAAA
CCTACGATTCTTGGAGTTCGAGAACATATATTTACTGGTTCCGTGTCATCCCTTGCTTGGTTCATGTCAG
CTCAGGAAATGAGTTTTGTAACCCTAGGACAGCGTGTATTAGCCAACCCCCTGAAAATCCGAATGCATTA
TGGACATCCAGATGTATTTGACAGGTTTTGGTTTCTAACTAGGGGAGGAATAAGCAAGGCATCTAAAGTG
ATCAACATCAGCGAGGACATTTTTGCTGGCTTCAACTGTACATTACGAGGTGGCAATGTCACTCACCATG
AGTATATACAAGTTGGCAAAGGAAGGGATGTTGGGTTGAATCAGATATCTATGTTTGAAGCCAAGGTTGC
CAGTGGCAATGGAGAACAAGTTCTTAGCAGAGATGTCTATAGGTTGGGTCATAGGCTGGATTTCTTCAGA
ATGCTTTCTTTCTTTTATACAACTGTAGGATTCTTCTTCAATACAATGATGATTGTCCTCACTGTATATG
CATTCTTATGGGGACGACTTTACCTGGCACTTAGTGGGGTTGAGGGCTCTGTTGCTGCAGATACCACCGA
CAACAACAGAGCACTTGGTGCCATACTGAACCAGCAATTTATCATCCAGCTGGGCCTTTTCACCGCATTA
CCAATGATTGTGGAGAACTCTCTCGAGCATGGTTTTCTTACATCTATCTGGGAATTTCTTACAATGATGC
TCCAACTTTCATCTGTATTTTACACATTCTCAATGGGAACTCGTGCTCATTACTTTGGTCGTACCATTCT
CCATGGTGGTGCAAAATACCGGGCAACTGGGAGAGGTTTTGTCGTGCAGCACAAGTGTTTTGCTGAGAAT
TATCGGTTATATGCTCGTAGCCATTTTGTCAAGGCAATTGAACTTGGTTTGATACTTACAGTGTATGCTG
CATACAGCCCTGTTGCTAAAGGAACTTTTACATATATAGCACTGACTATATCAAGTTGGTTCCTGGTGGT
GTCATGGATCTTGGGGCCCTTTGTGTTTAATCCTTCTGGGTTTGATTGGCTAAAGACAGTGTATGATTTT
GATGACTTCATGAACTGGATTTGGTACCGTGGTAGTGTTTTTGCGAAGTCAGACCAGAGCTGGGAGAAAT
GGTGGGAGGAGGAACAGGATCATTTAAGAACGACAGGTCTGTGGGGAAAGATACTGGAAATTATCCTAGA
CCTCCGCTTCTTCTTTTTCCAGTATGGCATTGTATATCATCTGGGTATTGCTGCTGGAAGCAAAAGCATT
GCTGTTTACTTGCTTTCATGGATTTATGTGGTGGTGGCTCTTGGCTTTTTTAATATTACAGCTTATGCTC
GGGAAAAATATGCTGCACGGGAGCACATATACTTTCGCCTTGTGCAGCTCCTTGCTGTACTCTTTTTCAT
AGTTGTAATTGTTGCTTTACTGCAGTTCACAGCATTTAAATTTGGTGATCTCTTTGTCAGCCTGTTGGCT
TTTGTTCCTACTGGTTGGGGCTTCATTTCAATCGCCCAAGTGTTACGTCCCTTTTTGCAGAAGAGTATGA
TATGGGGAACTGTTGTGTCTGTGGCGCGACTATATGAGATAATGTTTGGGATTATTGTCATGGTACCTGT
TGCAGTACTGTCTTGGTTGCCTGGTTTCCAACCAATGCAGACAAGGATCCTATTCAATGAAGCATTTAGT
AGAGGTCTGCGGATATTCCAGATTGTGACAGGAAAAAAGCCTAAGAGTGACGTGTGA
>XM_031397563.1_*Pistacia_vera*
ATGAGTAATCTCCGCCACCGTGCCGGTTCATCCCGGCCCAGACACGACCACCAACCCGAGGAAGAGGAGC
CTTACAATGTAATTCCTGTCCACAACCTCCTTGCTGACCACCCTTCCCTCCGCTTCCCCGAGGTACGAGC
CGCCGCCGCCGCTCTCCGCACCGTCGGAAGTCTCCGCAAGCCTCCATACACCCAATGGCTTCCCCACATG
GACCTTCTCGATTGGCTCGCACTGTTCTTCGGATTTCAAAAGGACAACGTTCGAAACCAGCGTGAACACT
TGGTTCTCCATTTGGCCAATGCTCAGATGCGTTTAACGCCACCTCCGGACAACATCGACACGCTGGACGG
CAGCGTTTTGAGGAAATTCCGGCGCAAATTGTTAAAGAATTACACCAACTGGTGTTCGTATTTGGGGAAG
AAATCGAACATCTGGATCTCGGATCGGAACTCCGACCAACGTCGTGAACTCTTGTATGTATCGTTGTATC
TTTTGATTTGGGGCGAAGCAGCGAATCTGAGGTTCATGCCAGAGTGTATTTGTTATATTTTTCACAACAT
GGCGATGGAATTGAACAAGATTTTAGAAGATTATATTGACGAGAACACGGGTCAGCCCGTGATGCCTTCC
GTATCGGGTGAGAACGCGTTTTTGAACTTTGTGGTGAAGCCCATTTACGAGACGGTGAAGGCGGAAATGG
AGAGTAGTAAGAACGGAACAGCGCCACATAGTGCGTGGAGGAACTATGATGATATTAATGAGTATTTTTG
GAGTAAGAGGTGTTTTCAGAAGCTGAAATGGCCAATCGATGTTGGGAGTAATTTTTTTGTGCTGTCAAGT
AGGACTAAACACGTCGGGAAAACTGGGTTTGTGGAGCAACGATCGTTTTGGAACTTGTATAGGAGCTTTG
ACAGGTTGTGGGTGATGTTGATATTGTTTATTCAAGCAGCGATTATTGTGGCTTGGAAGGGGAAGGAGTA
TCCGTGGCAATCGTTGGAGGAAAGAGAAGTGCAGGTTCGTGTTTTAACTGTGTTTATTACATGGAGTGGA
TTGAGATTTTTGCAGTCTTTGTTAGATATTGGAACGCAATATAGACTTGTTTCGAGAGAGACAATGGGGC

TTGGTGTGAGGATGTTGTTAAAGAGTGTTTTTGCGGCTGTGTGGATTTTGGTTTTTGGAGTGTTTTATGC
GCGGATTTGGGTGCAGAAGAATCGGGATAATAATTGGTCTGCTCAGGTAGATAGGCGTATAGTAAATTTT
CTTCAGGCGGCATTTGTGTTTATTTTGCCTGAGTTGTTGGCAGTAGCTTTGTTTATTCTTCCTTGGGTGA
GGAACTTTCTGGAAAGAACGAATTGGAAGATCTTTTATTTGTTAACATGGTGGTTTCAGAGTAAAAGTTT
TGTTGGTCGTGGATTGAGAGAAGGGCTTGTGGATAATCTCAAGTATAGTTTATTCTGGATAGTAGTGTTA
GCTACTAAATTTTCGTTCAGTTACTTCCTGCAGATCAAACCCTTGGTTGACCCAACGAAACGATTGTTAC
AACTTAATGATGTGAAGTATGAGTGGTATCAACTCTTCCCTGACAGCAACAGGTTGGCTGTTGGGCTACT
GTGGCTGCCTGTAATTTTGATATATTTGATGGATATTCAGCTTTGGTACGCAATTTATTCATCTCTTGTG
GGTGCAACAGTGGGCTTGTTCCAGCATTTGGGTGAGATTCGAAATATACAGCAGTTGAGGCTAAGATTTC
AGTTCTTTGCAAGTGCTATGCAGTTTAATCTAATGCCTGAGGAGCAGCTGTTGAATGTGAGGGGAACGCT
GAGGAATAAGTTCAACGATGCCATTCATCGGTTGAAACTGAGATATGGCCTTGGGAGACCCTATAGGAAG
CTTGAATCGAACCAGGTTGAGGCAAACAAATTTGCCTTGATCTGGAATGAGATAATTTCCATTTTTAGGG
AAGAGGATATCATCTCTGATACGGAGGTTGAACTGTTGGAGCTGCCGCAGAATTCTTGGAATGTATCGGT
TATTCGTTGGCCTTGTTTCTTGCTGTGCAATGAGCTGCTTCTAGCTCTTAGCCAGGCTAAGGAGTTGGTG
GATGCTCCTGACAAGTGGCTTTGGTATAAGATTTCTAAGAATGAGTATAGGCGTTGTGTTGTGATAGAAA
CTTATGATAGCCTGAAGCACTTGATTCTTAATATCATCAGAGTTAACACTCAAGAGCATTCCATTATGAC
GGTCGTGTTTCAAGAAATTGATCACTCCATTCAGATTGAGAGGTTCACTAAAACATTCAAGATGTCTGTT
TTGCCCAAAATTCATGCCCAGTTGATCAAACTTGTTGATTTGTTGAACAAGCCCAATAAGGACATTAACA
AGGTGGTGAATGCCCTACAGGCCCTTTATGAGATCGTTATTCGAGATTTTTTTTTGGAGAAAAGGGGCAC
TGACCAGCTTGTTGAGGATGGCTTGGCTCCTAGTAATCCAGCCTTGCTGTTCGAGAATGCTGTTCAGTTG
CCTGACTCTGATAACGAGAACTTCTATCGTCAGGTTAGGCGCTTACACACAATTCTAACATCTAGGGATT
CAATGAATAATATTCCTGTTAATCTTGAAGCAAGGCGCAGAATAGCTTTTTTTAGCAACTCACTCTTCAT
GAATATGCCCCATGCTCCCCAAGTTGAGAAGATGATGTCCTTCAGTGTTCTCACCCCTTACTACAATGAA
GAAGTTCTCTACAGTAAAGAGCAACTACGAACTGAGAATGAGGATGGCATATCCATCCTATACTATTTGC
AGACAATTTATCCTGATGACTGGAAGAATTTCATGGAAAGGATGCGCCAAGAAGGAATGGTGAAGGATAA
TGAAATCTGGACGGAGAAGTTGAAGGATCTGAGGCTTTGGGCATCATACAGAGGCCAGACACTTGCTCGC
ACTGTGAGGGGAATGATGTATTATTATCGGGCTCTTAAGTTGCTGGCTTTTCTTGATTCTGCATCTGAGA
TGGACATTAAGGAAGGGACCCGAGAACTTGGTTCAATGAGGCGGGATGGCAGCTTGGATCGTTTCGGCTC
AGCAAGGTCATCGTCTGTGAAAAGTTTAAGTAGAAATGGTAGTTCAGTTAGCATGCTATTCAAGGGCCAT
GAGTATGGAACCGCTTTGATGAAATACACATATGTTATTGCCTGCCAGATATATGGGCAACAAAAGGAGA
AGAAAGACCCCCATGCTGAAGAAATTTTGTATCTGATGAAGAACAATGAAGCCCTTCGAGTTGCATATGT
TGATGAGGTTTCCACAGGGAGGGATGATAAGGAGTATTACTCTGTTCTTGTGAAATATGATCAGCAGTTA
CAGAGGGAAGTGGAAATCTACAGGGTCAAATTGCCTGGTCCTTTGAAGCTTGGCGAGGGAAAACCGGAGA
ACCAAAACCATGCCCTTATCTTCACCAGAGGAGATGCAGTTCAGACTATTGATATGAATCAGGACAATTA
CTTTGAGGAGGCACTTAAAATGCGGAATTTGTTGGAAGAATACAGGCACTACCATGGTATCAGGAAACCT
ACCATCTTGGGCGTTAGGGAGCACATTTTTACTGGCTCAGTGTCGTCACTTGCTTGGTTTATGTCGGCTC
AGGAAACCAGTTTTGTCACCTTGGGTCAGCGTGTCTTGGCAGACCCTTTGAAAATCCGAATGCATTATGG
CCATCCTGATGTCTTTGACAGGTTTTGGTTCTTAACTCGAGGAGGCCTCAGCAAAGCTTCTAGAGTGATT
AACATCAGTGAGGACATTTTTGCTGGCTTTAACTGCACATTACGAGGAGGCAGTGTTACACACCATGAAT
ACATCCAAGTTGGCAAGGGAAGGGATGTTGGATTGAATCAGATATCTATGTTTGAAGCCAAGGTTGCTAG
TGGAAATGGTGAGCAAGTTCTCAGCAGAGATGTGTATAGGTTGGGTCATAGACTGGACTTCTTCCGTATG
CTATCATTTTTTTATACTACTGTTGGATTCTTTTTCAACACAATGTTGGTTGTGTGGACTGTGTATGCAT
TCTTATGGGGCCGACTTTATCTGGCTCTTAGTGGTGTTGAGGATTCTGTTGCAAACGACAGTGAAACCAA
TAAAGCACTTGGTGCCATCTTGAACCAACAATTCATCATCCAACTTGGTTTATTCACAGCCCTTCCGATG
ATTGTGGAGAATTCTCTTGAGCATGGATTTCTTCAAGCTATTTGGGATTTCCTGACTATGTTGCTCCAGC
TTTCATCAGTTTTCTACACATTCTCCATGGGAACTCGCACTCACTTCTTTGGCCGGACTATCCTTCATGG
TGGTGCAAAGTATCGAGCTACTGGACGTGGTTTTGTTGTGCAGCACAAGGGCTTTGCAGAAAATTATCGG
CTTTATGCACGTAGCCACTTTATAAAAGCCATTGAGCTTGGATTGATTCTTATAATTTATGCATCACACA
GCCCTATAGCTAAGGACACATTTGTTTACATAGCCATGAGCATCTCAAGTTGGTTCATGGTTATTTCGTG
GATAATGGCCCCGTTTGTCTTCAATCCTTCTGGTTTTGATTGGTTGAAGACTGTATATGACTTTGATGAA
TTCATGAACTGGATTTGGTTCCGTGGTGGTGTGTTTGCAAAAGCAGAACAGAGCTGGGAAGAATGGTGGA
GTGAGGAACAGGATCATCTAAAGACAACTGGTATTTGGGGAAAGATACTGGAGATAATTTTAGACCTCCG
CTTCTTCTTTTTTCAGTATGCCCTAGTATACCAGCTAGGTATTGCTGCTGGAAGTACTAGTATTGCTGTT
TACTTGCTTTCTTGGATCTATGTTGTTGTGGCGTTTGGGATTTATGCAGTAATATCCTATGCTCGTGACA

AATATGCGGCAAAAGAACACATCTACTACCGACTGGTTCAGTTCCTTATTGTCATATTTGCGATACTTGT
GATAATTGCCCTAATGGAATTTACAGAATTAAAGTTTATAGATCTTTTCACCAGTCTGTTGGCTTTTATC
CCTACTGGGTGGGGCCTGATATTAATTGCCCAAGTATTCCGGCCCTTTTTGCGGCCCACAGGGGTTTGGC
AGGCTGTGGTTTCTGTTGCTAGATTGTACGATATAATGTTTGGAGTGATTGTCATGACCCCTGTGGCATT
TCTATCATGGATGCCTGGATTCCAGTCTATGCAGACAAGGATACTATTCAATGAAGCATTCAGCAGAGGC
CTTAGGATATTCCAGATTTTTACCGGAAAACAAGCTAAGGGCGACATATAA
>XM_031235888.1_*Ipomoea_triloba*
ATGAGTAACCGCCAACGCCCCCCGCCGCCGGCGCAGCAAAGACAGCAGCGACCTCATCCTCTCGGAGAGG
ATGAGCAGCCCTACAATATCATTCCGATCCACAATCTCTTGGCGGACCACCCGTCTCTCCGCTTCCCGGA
GGTGCGAGCCGCGGCGGCGGCTCTGCGCTCAGTCGGCGAGCTGCGGAAGCCGCCGTTCTGTCCGTGGAGG
CCGGAGTATGATCTCCTCGACTGGCTCGCGCTCTTCTTCGGCTTCCAGGCTTCCAGTGTTCGAAACCAGA
GGGAGCATCTCGTTCTGCACCTCGCTAATGCTCAGATGCGGCTCACTCCGCCCCCCGACAACATCGATTG
TCTCGACCCTGGGGTTCTGCGCCGCTTCCGCCGGCAGCTCCTCAGGAACTATACCTCCTGGTGCTCTTTC
CTCGGACTTAAATCTAACGTTTGGCTCTCCGAACGACACAATTCCGCCTCCGATAACCGCCGCGAGCTCC
TTTATGTGTCGCTCTACCTACTTATCTGGGGTGAGTCCGCTAATATCCGGTTTGTTCCTGAGTGTATTAG
CTATATTTTTCATAACATGGCCATGGAATTGAATAAAATTTTGGAGGATTACATCGATGAGAACACCGGC
AGTCCGTTTTTGCCCTCGATTTCGGGTGAGAATGCGTATCTGAATCGAATTGTGAAGCCGATTTATGAGA
CAATACGAGCTGAGGTTGAGAATAGTAGAAATGGCACTGCTCCACACTCTGCTTGGAGGAACTATGATGA
TATAAATGAGTATTTCTGGACTAAGAGGTGCTTTGAGAAGTTGAAGTGGCCGATTGATATTGGTAGCACC
TTTTTTGTGACCACAGATAAGGGGAAGAAGGTAGGGAAGACAGGGTTTGTGGAGCAGAGATCCTTTTGGA
ATTTGTTTAGGAGCTTTGATAAGTTGTGGATCATGCTTGCTCTGTTCCTCCAGGCTGCAATTATTGTGGC
TTGGGAGGGAAAGGAGTATCCATGGCAAGCTTTGAAGAGTAGAGAGGTGCAAGTGAAGGTTCTGACTGTG
TTCTTCACATGGAGTGGGATGAGGTTCTTGCAGTCATTGCTTGATATGGGGATGCAGTATAGACTGGTTT
CAAGAGAGACACCATGGCATGGTGTGAGAATGGTGTTGAAGGCCATCGTTTCAGCTGGATGGATTGTGGT
TTTTGGTGTGTTCTATGGCAGGATCTGGACCCAGCGGAATAATGATCGGGGTTGGTCAGGCGAGGCTAAC
AGGAGGGTGGTGAATTTCCTTGAGGTCTCCTTGGTTTTCCTTGCCCCTGAGATTTTGGCTTTAGCCTTCT
TTATTCTGCCCTGGGTTCGGAATTTTCTTGAGAACACTAACTGGAAGATATTTCACTTGCTGCTGTGGTG
GTTCCAGAGCCGGACATTTGTGGGTCGTGGACTTAGGGAAGGGCTTGTTGATAATATAAAGTATAGCCTG
TTCTGGGTGCTTGTGCTTGTTACTAAGTTTACTTTCAGTTACTTTTTACAGATTAAACCAATGATTGCTC
CAACAAAGACACTGTTACGTCTTAAGATCGATACATATGAATGGCATCAATTTTTTGGCAACAGCAATAG
GTTTGCTGTAGGGTTGCTTTGGCTTCCTGTAGTTCTGATCTACCTTATGGATATACAGATATGGTATGCT
ATCTACTCTTCCTTTACTGGTGCGGCAGTTGGGTTATTTGACCACTTGGGTGAGATTCGGAATATGCAAC
AGCTGAGGTTGAGGTTCCAGTTCTTTGCAAGTGCCATTCAGTTCAATCTGATGCCAGAGGAGCAGTTACT
GAATACTCATGGAACATTTAAGAGCAGGATCAAGGATGCCATTCACCGCTTGAAACTCAGATACGGATTT
GGTCGGCCATTTAAAAAGCTTGAGTCCAGCCAGGTAGAGGCTAACAAATTTGCATTGATATGGAATGAGA
TAATTTTGACATTTAGAGAAGAAGATATCATCAGTGATCATGAGGTTGAGCTGTTGGAGCTGCCCCAGAA
CACCTGGGATGTTAGAGTGATACGTTGGCCATGTTTACTGCTTTGCAATGAGCTTTTGCTTGCTCTTAGT
CAGGCAAAAGAGTTGGTAGATGCTCCTGACCATTGGCTCTGGTCTAAGATTAGCAAGAGCGAATACAGGC
GGTGCGCAGTTATTGAAGCTTATGAATGTACTAGACACTTGTTACTGGAGATTGTCACATTAAATAGTGA
GGAACACTCCATTCTCAGCACTTTCTTCCAGCAAATTGATGAATGGGTTAAACTGGAAAAGTTCACAAAA
TACTACAACCTGACTGCACTTCCCAAAATATGTGACAAATTGACCATTCTTCTCAATCTAAGTCTCAATC
CTAAAAGGGACATTGACAAAGTGGTGAATGTTCTACAGGCCCTGTATGAGATTGCAACCCGGGATTTTCT
CAAGGAGAAGATGACTGCAGATCAGTTACGAGAGGATGGTCTTGCACCTCGGACTTCTGGGGATAAATTG
CTTTTTCAGAATGCAGTTGTGTTTCCTGATCCTAACAATGAGATCTTTTATCGGCAAGCTCGGCGCTTAC
ACACTATTCTTACTTCTCGTGACTCTATGAGTAACATTCCAAGAAATCTTGAGGCAAGACGTCGACTTGC
CTTCTTTAGCAACTCTCTTTTTATGAACATGCCACATGCTCCTCATGTTGAGAAAATGAGAGCTTTCAGT
GTTTTGACCCCATACTACAATGAAGAAGTGTTGTACAGTAAGGAACAACTTCGAACTGAAAACGAAGATG
GTATTTCCATACTCTATTACTTGCAGACGATCTATGCTGATGAGTGGGAGAATTTCTTGGAGCGAATGCG
ACGAGAAGGAATGACTAATGAGATAAGAGAGTTGTGGACAGAAAGGCTTAAAGATCTTAGGCTTTGGGCA
TCATACAGAGGCCAGACTCTTGCACGAACAGTGAGAGGAATGATGTATTATTACCGAGCTCTTAACATGC
TGGCTTTTTTGGATTCAGCTGCTGAGGTGGACATAAAGGAAGGATCACGCGAGCTTGCTTCTATGAGGCG
TAGTGAGGGACCTGATGGTTTGAGCTCAGAAAGGGCACCATCATCTAGAAGTTTGAGCAGAGCTGATAGT
TCTGTGAGCTTGTTGTTTAAAGGTCATGAATATGGGACTTCTTTAATGAAATTCACTTATGTGGTTGCCT
GTCAGATTTATGGTACTCAGAAGGCCAAAAGGGATCCCCACGCTGAAGACATCTTGAAGCTGATGGAAAA

CAATGAAGCTCTTCGTGTTGCTTATGTTGATGAGGTTGTAAGAGGAAGGGATGAGAAAGAGTATTATTCT
GTGCTAGTGAAGTACGACCAAAAACTGAAGAAGGAAGTGGAGATCTATCGAGTCAAGTTGCCTGGTCCAT
TGAAGCTTGGGGAGGGAAAGCCTGAAAATCAGAATCATGCTCTTATTTTCACTCGGGGAGATGCAGTCCA
GACAATTGATATGAACCAAGATAATTACTTTGAGGAGGCACTGAAAATGAGGAATCTGTTGGAGGAATTC
AAGCATTACTATGGAATCCGCAAGCCAAAAATTTTGGGAGTTAGGGAACATATTTTTACTGGTTCAGTCT
CATCCCTCGCTTGGTTCATGTCAGCTCAAGAAATGAGTTTTGTCACTCTGGGTCAACGTGTCTTAGCTAA
CCCTCTGAAAATCCGAATGCACTATGGGCACCCAGATGTGTTTGACAGGTTTTGGTTTTTGACTCGGGGA
GGGATTAGCAAAGCATCTAGAGTGATCAACATTAGTGAAGATATTTTTGCCGGCTTTAACTGCACATTGC
GAGGTGGGAATGTTACCCACCATGAATACATCCAAGTTGGCAAGGGAAGGGATGTTGGACTGAATCAAAT
AGCTATGTTTGAAGCCAAGGTTGCCAGTGGGAATGGCGAGCAAGTTCTGAGTAGGGATGTCTATAGGTTG
GGCCATAGGCTTGACTTCTTCAGAATGCTCTCATTTTTTTATACAACTGTTGGGTTTTATTTCAACACAA
TGATGATTGTTCTTACAGTTTTTGCATTCTTATGGGGAAGACTCTACCTGGCTCTTAGCGGGCTTGAGGA
CTCTATTGCTGACAGTGGTACCAATGACAACAAAGCACTCGGTACAATTTTGAACCAACAGTTCATCATT
CAGCTGGGTCTATTCACTGCTTTACCAATGATTGTGGAGAATTCTCTTGAGCATGGTTTTCTTACTTCTA
TTTGGGAATTTATAACAATGCAACTCCAACTTTCTTCTGTATTTTACACTTTCTCAATGGGAACGCGTGC
CCACTATTTTGGCCGAACTATTCTTCATGGTGGTGCCAAGTACCGGGCAACTGGACGTGGCTTTGTGGTA
CAACACAAGGGTTTTGCTGAAAATTATAGGCTTTATGCTCGTAGCCACTTTACCAAGGCAATTGAACTTG
GGCTCATACTTACAATATATGCCTCATACAGCCCTGTAGCTACCAGAACTTTTACATACATAGCATTGAC
CATCTCAAGTTGGTTCTTGGTGGTGTCATGGATCTTGGCGCCCTTTGTGTTTAATCCTTCTGGATTTGAT
TGGTTGAAGACAGTGTATGATTTTGATGAGTTTATGAGCTGGATATGGTACCGGGGTGGTGTTTTTGCAA
AAGCTGAACAAAGTTGGGAAAAATGGTGGGATGAAGAACAGGATCATTTAAGGACAACTGGGCTTTGGGG
GAAGATACTGGAAATAATTTTAGACCTCCGTTTCTTCTTTTTCCAGTATGGAATTGTTTATCAGCTAGGC
ATTGCTGCTGACAGCAAGAGCATTGCAGTTTACTTGCTTTCCTGGATTTATGTGGTGGTAGCTCTTGGGA
TTTACACCATTATAGCTTATGCTCGGGATAAGTATGCTGCAAAGGAGCACATATATTATCGCCTGGTTCA
GTTCCTAGTTATAATTCTTTTCATAATTCTGATAATTGCACTGCTCCAGTTCACAGATTTCAAATTTATC
GATCTATTCACTAGCCTGCTGGCTTTTGTTCCCACTGGTTGGGGTCTCCTCTCATTTGCGCAAGTGCTAC
GTCCCCTTTTGCAAAACACTTTTATCTGGGGGACAGTTGTTGCTGTGGCTCGACTGTATGAAATCATGAT
TGGAGTGATTGTCTTGATACCCGTGGCACTATTATCCTGGTTGCCTGGATTCCAACCCATGCAGACAAGG
ATCCTATTCAATGATGCTTTCAGTAGAGGCCTGCGGATATTCCAGATTGTGACCGGGGCAAAAAAAACAA
AGCGTGATGTCTGA
>XM_031076142.1_*Quercus_lobata*
ATGAGCTACCACCGCCAACGCCCACCTCCGAGCCCGAACCCGAGACAAACCCGGCCCAGTCCAGCCCCAC
CGCCATTAGAATCCGATCCGTACAACATAATCCCCATCCACAACCTCCTCGCGGAGCACCCGTCGCTCAG
ATTCCCGGAGGTCCGAGCCATTACCACCGCGCTCCGAGCCGTCGGCGACCTCAGGAAGCCGCCGCACAAC
CAATGGGCTCCGCACATGGACCTCCTGGACTGGCTCGCCCTCTTCTTCGGCTTCCAGAACGACAACGTTC
GCAACCAGCGCGAGCACCTCGTCCTCCACCTCGCCAACGCTCAGATGCGCCTCTCTCCTCCGCCGGACAA
CATCGACGCCCTGGACGCCGCCGTTTTGCGCAAGTTTCGGAGGAAGCTCCTCAAGAACTACACCAACTGG
TGCTCCTATCTCGGCAAGAAATCCAATATCTGGATCTCCGATCGCCGCGAGGCCGCCGCCGATCACCGCC
GGGAGCTGCTCTACGTGTCGCTCTTCCTTCTGATTTGGGGTGAGTCTGCTAACCTCCGCTTTGTTCCCGA
GTGCATTTGCTTTATATTTCATAACATGGCCATGGAGTTGAACAAGATCTTAGAGGATTACATAGATGAG
AACACCGGCCAACCTGTGCAGCCCTCGGTCTCCGGCGAAAACGCGTTTTTGAACTGCGTTGTGAAGCCGA
TTTACGAGACGATTAGGGCTGAGGTGGAGAGTAGTAAGAATGGCACTGCGCCGCACAGTGTGTGGCGCAA
TTACGACGACATTAACGAGTACTTTTGGAGTAAACGGTGTTTTCAGAAGCTCAGATGGCCGATTGATTTG
GGAAGTAATTTCTTTGTGACGGGTGAGGGTGGCGCGAGAAGAAAACATGTAGGGAAGACTGGTTTTGTGG
AGCAGAGATCGTTTTGGAACTTGTTGAGGAGCTTTGACAGGCTTTGGGTGATGCTTGTGTTGTTTTTACA
GGCGGCGATTATTGTGGCGTGGGAAGAGAGGAGATATCCATGGGAAGCTCTGCAGTCTAGGGATGTCCAA
GCGAAAGTTTTGACAGTGTTTTTCACTTGGAGTGGGATGAGGTTTTTGCAGTCTCTGCTGGATGCAGGGA
TGCAATACAGTTTGGTTTCGAGGGAGACAATGGGGCTTGGCGTGAGGATGGTGATGAAGAGTGTGGTTGC
AGCCATGTGGATCTTGGTTTTTGGTGTGTTTTATGGGCGGATATGGGCGCAGAGGAATCGCGATAGGAGG
TGGTCTGCAGAGGCTAATAGGAGGTTGGTTACTTTTCTTGAGGTGGCATTGGTTTTCATTTTGCCGGAGC
TTCTGGCTGTGGCGCTGTTTGTGATTCCGTGGATCAGGAATTTCCTCGAGGAGACGAATTTGAAGATCTT
TTATATGTTGACTTGGTGGTTTCAAGGGAGGACCTTTGTGGGTCGTGGGTTGAGGGAAGGTCTTGTGGAT
AATATCAAGTACAGTTTGTTTTGGATTGTGGTGCTTGCTACCAAATTTTTGTTCAGTTACTTCTTGCAGG
TTAAACCGATGATTGGCCCAACGAAAGCATTGTTGGATTTGAAGGATGTGCAGTATCAATGGTATCAGCT

TTTTAACAATAGCAACAGATTGGCAGTGGGCTTACTGTGGATTCCTGTTATTCTGATTTACCTCATGGAT
ATTCAGATTTGGTATTCAATCTACTCGTCTTTTGTTGGGGCGGGTGTGGGATTGTTTCAGCACTTGGGTG
AGATTCGAAATATTGAACAGTTGAGGTTGAGGTTCCAATTCTTTGCAAGTGCTATTCAGTTTAATCTCAT
GCCGGAGGAGCAGCTGTTAAATGCAAGGGGGATGAGGAACAGGTTTAAGGATGCCTTTTATCGATTGAAG
CTGAGATATGGGCTTGGTCGGCCCTATAGGAAGCTTGAATCTAGTCAGGTTGAGGCGAAGAGGTTTGCTT
TGATATGGAATGAGATAATTTCGATTTTCAGGGAAGAAGACATCATCTCTGACCGTGAGCAGGAGCTGTT
GGAGCTGCCCCAGAATTCTTGGAATGTCAGGGTCATTCGATGGCCTTGTTTCCTCCTCTGTAATGAGCTT
CTGCTTGCGCTCAGTCAGGCCAAAGAGTTGGTAGATGCTCCTGACAAGTGGCTCTGGTATAAGATATGCA
AGAATGAGTACAGGCGTTGCGCTGTGATTGAAGCTTATGATTGTATCAAACACTTGCTGCTTGAGATTAT
CAAACGCAACACAGAAGAGCATTCCATTATGACCGTATTTTTTCAAGAGATTGATCACTCCATTGAGATT
GAGAGGTTCACTAAAACATTTAACATGACTGTTCTGCCCCAGCTTCATACCCAGTTAATCAAACTTGTTC
AGCTATTGAACAAGCCAAAGAAAGATCCTACCCAGGTAGTGAACACTCTGCAAGCCATTTATGAGATTGC
TAAACGGGACCTTTTCAAAGACAAGAGGAGCATTGACCAGCTGAGGGAGGATGGTCTGGCTCCTCGTAGT
CCAGCTTCCACACAGGGGCTGCTTTTTGAGAATGCTGTTGAATTGCCTAATTCAGATAATGAGACATTCT
ATAGGCAGGTTCGGCGGTTGCACACAATTCTTACCTCTCGGGACTCAATGCACGATATCCCAGTAAATCT
TGAGGCAAGACGCCGAATTGCCTTCTTCAGTAATTCACTTTTCATGAACATGCCTCATGCCCCTCAGGTT
GAGAAAATGATTTCCTTCAGTGTTCTGACCCCTTATTACAGTGAAGAAGTAATCTATAGCAAAGAACAAC
TCAAATCTGAGAATGAAGATGGTATTTCGATCCTGTACTATTTGCAGACAATTTATGCCGACGAGTGGAA
AAATTTCTTGGAGAGAATGCGCCGAGAAGGGATGGTGAGGGATACCGAGATATGGAATACTAAGCTGAGA
GATCTCAGGCTTTGGGCATCATACAGGGGTCAGACACTCTCCCGAACTGTTAGGGGAATGATGTATTACT
ATCGGGCTCTTAAGATGCTGGCATTTCTGGATTCTGCATCGGAGATGGACATTCGGGAAGGATCACAAGA
ACTTGGTTCGATGAGGCGAGACAGCGGTTTTGATGGTTACTCCTCGGAAAGGTCACCATCTTCTAGGAGA
TTAAGCAGAGCAAGCAGTTCAATGGACTTGTTACTCAAAGGCCACGAGTATGGTACTGCATTGATGAAAT
TTACATATGTGGTTGCCTGCCAGATATATGGAACTCAGAAGGCAAAGAAAGATCCACATGCCGAGGAAAT
CTTGTATCTAATGAAAAACAATGAAGCCCTTCGAGTTGCCTACGTTGATGAAGTTTCCACTGGGAGGGAT
GAGAAGGAATTTTATTCTGTTCTTGTGAAGTATGATCACCAATTGGAGAGGGAAGTGGAGATCTACAGGG
TAAAGTTGCCTGGTCCCTTGAAGCTTGGTGAGGGAAAACCGGAGAATCAAAATCATGCCATCATTTTCAC
TCGTGGTGATGCTGTCCAGACTATTGATATGAACCAAGACAATTATTTTGAAGAGGCACTCAAAATGCGC
AATCTGCTGGAAGAATTCAGGCGTTATTATGGTATCCGGAAGCCTACTATCTTGGGAGTTAGGGAACACA
TATTTACAGGTTCTGTTTCATCACTTGCTTGGTTTATGTCGGCTCAGGAAACGAGTTTTGTCACCTTGGG
GCAGCGTGTTTTGGCGAACCCTTTAAAAGTTCGAATGCATTATGGCCATCCAGATGTGTTTGACAGGTTT
TGGGTCATGACTCGTGGTGGATCAGTAAAGCTTCCAGAGTGATTAATATCAGTGAGGACATTTTTGCTG
GCTTTAACTGCACATTGCGTGGAGGGAATGTCACTCACCATGAATACATCCAGGTCGGCAAGGGAAGGGA
TGTTGGGTTGAATCAAGTGTCCATGTTTGAGGCCAAGGTGGCTAGTGGAAATGGTGAGCAAGTTCTTAGC
AGAGATATCTACAGGTTGGGTCATAGGTTGGACTTCTTCCGAATGCTGTCATTCTTTTACACTACTGTGG
GATTCTTTTTCAACACAATGGTGGTGATTCTGACTGTATATGCATTTCTGTGGGGCCGACTCTATCTGGC
TCTTAGTGGTGTTGAGGCTTCTTCTGAGGCTAGTACTAATAACAATGCAGCACTTGGTGCAATCTTGAAT
CAGCAGTTCATCATCCAGCTTGGTCTGTTCACTGCCCTTCCGATGATAGTGGAAAACTCTCTTGAGCATG
GGTTCCTTCAAGCTATCTGGGATTTTCTGACAATGCAGCTCCAGCTTTCATCCGTATTCTACACGTTCTC
AATGGGAACTCGTGCCCACTACTTTGGCCGTACTATTCTTCATGGTGGTGCAAAATATCGGGCTACCGGG
CGTGGTTTTGTTGTGGAGCACAAGGGTTTTGCTGAGAATTATAGACTCTATGCTCGTAGCCATTTTGTGA
AGGCAATTGAACTTGGGTTAATACTTGTAGTTTATGCGTCACACAGTCCTATAGCTGGCAACACATTTGT
TTACATAGCCTTGACCATCACTAGTTGGTTCCTGGTTGTGTCATGGATTATGGCCCCCTTTGTGTTCAAT
CCTTCTGGATTTGATTGGTTGAAGACAGTGTACGACTTTGATGACTTTATGAACTGGATTTGGTTCCGTG
GCAGTGTTTTTGCAAAAGCTGAACAGAGTTGGGAAAGATGGTGGTATGAGGAGCAAGATCATCTCAGGAA
CACTGGCATTTGGGGTAAAATAATGGAAATAATCTTAGACCTCCGATTCTTCTTCTTCCAGTATGGGATA
GTATATCAACTAGATATTGCAGCTGGAAGTACCAGCATTGCTGTTTACTTGTTGTCTTGGATCTATGTAT
TTGTGGCTTTTGCGATTTATGCAGTAATAGCATTTGCTCGGGATAAATATGCGGCAAAAGAGCACATATA
CTATCGTCTAGTCCAATTCCTTGTGATTATACTTGCAATACTTGTGACAATTGCTCTGCTGCAATTTACA
GCTTTCACATTTATTGATATTTTCACTAGCATGTTGGCATTCATCCCCACTGGGTGGGCCCTGATATTGA
TTGCCCAAGTACTCCGGCCCTTTCTGCCTACTCTTGTTTGGGAAAGTGTTGTTTCTGTGGCCCGACTATA
TGATATAGTGTTCGGAGTAATTGTCATGGCTCCTGTGGCATTACTGTCATGGTTGCCTGGGTTTCAGTCA
ATGCAGACAAGGATCCTTTTCAATGAAGCATTTAGTAGGGGCCTCCGCATTAACCAGCTTGTTGCAGGAA
AAAAATCCAATGACTTATAA

>XM_023781713.1_*Capsella_rubella*
ATGAGCCTCCGCCACCGCACCGTCCCGCCGCAAACCGGACGGCCGTTGGCGGCGGATGCTGCCGGAATCG
AAGAGGAGCCTTATAATATCATTCCCGTTAACAATCTCCTCGCCGACCATCCTTCCTTACGGTTTCCCGA
GGTTCGTGCCGCCGCCGCTGCTCTTAAAACCGTAGGGGACCTTCGTCGTCCGCCGTATGTTCAATGGCGC
TCTCACTACGATCTCCTCGACTGGCTCGCCTTGTTCTTTGGTTTCCAGAAGGATAACGTTCGTAACCAGC
GTGAACATATGGTGCTTCATCTCGCGAATGCTCAGATGCGTCTCTCTCCGCCGCCGGATAATATTGATTC
TCTCGATTCCGCTGTTGTTCGTCGGTTTCGTCGGAAACTTCTTGGTAACTACTCTAGCTGGTGTTCGTAT
TTGGGGAAAAAATCAAATATCTGGATCTCAGATCGGAACCCTGATTCGAGACGTGAGCTTCTCTATGTTG
GACTCTATCTTCTCATATGGGGAGAGGCCGCGAATCTTAGGTTTATGCCTGAATGTATTTGTTACATCTT
CCATAACATGGCCTCGGAGCTCAACAAAATCTTAGAGGACTGCCTCGATGAGAACACCGGTCAACCTTTT
TTGCCTTCTATCTCAGGCGAAAACGCTTTCTTAATCGGGGTCGTTAAACCTATTTACGATACAATCCAAG
CTGAGATTGATGAGAGCAAGAACGGTACAGTTGCGCATTGTAAGTGGAGAAACTACGACGATATCAATGA
GTACTTCTGGACGGATCGATGTTTCAGCAAATTGAAATGGCCGCTTGATTTGGGAAGCAATTTTTTCAAG
AGTAGAGGCAAAAGTGTAGGGAAAACTGGTTTTGTGGAGCGCAGGACATTCTTCTACCTATACAGGAGTT
TTGATCGGCTTTGGGTGATGCTAGCTTTGTTTCTTCAAGCCGCCATTATTGTAGCTTGGGAGGAAAAACC
TGAGGATAAGTCGGTGACAGTGCAGCTATGGAATGCTTTGAAGGCAAAAGATGTTCAGGTGAGACTTTTG
ACCGTGTTCTTGACCTGGAGTGGTATGAGACTCTTGCAGGCTGTGCTGGACGCGGCTTCACAATTTCCCC
TCATTTCGAGAGAGACCAAACGGCACTTTTTCAGAATGCTGATGAAGGTTATAGCTGCCACAGTTTGGAT
CATAGCTTTTACAGTACTTTACATCAACATCTGGAAGCAGAAGAGGCAAGACAGACAGTGGTCCAATGCC
GCAACGACTAAGATCTACCAATTCCTTTACGCTGTGGTGGCCTTCTTGGTGCCCGAAATTCTGGCTTTGG
CTTTGTTTATAATCCCATGGATGAGAAACTTTCTGGAAGAGACCAATTGGAAAATATTCTTTGCTCTAAC
TTGGTGGTTTCAGGGCAAAAGCTTTGTAGGTCGAGGTTTGAGAGAGGGTTTAGTGGATAACATTAAGTAC
TCGACTTTCTGGATTTTTGTCCTAGCTACAAAGTTCACATTTAGCTACTTCCTCCAGGTTAAGCCAATGA
TTAAACCCTCAAAGCTGCTATGGAACTTAAAGGACTTAGACTATGAGTGGCATCAATTTTTTGGTGACAG
CAATAGGTTTTCTGTCGCATTGTTATGGTTGCCCGTTGTGTTGATATATCTGATGGATATTCAAATTTGG
TATGCAATATATTCTTCAATTGTTGGTGCTGTTGTTGGGCTGTTTGATCATCTGGGGGAGATCAGGGACA
TGGGACAGCTTAGGTTGAGGTTTCAATTCTTTGCTAGCGCTATTCAGTTCAACCTAATGCCTGAGGAACA
ACTCCTGAATGCTAGAGGCTTTGGTAATAAGTTCAAGGACGGCATTCATAGATTGAAGCTAAGGTATGGA
TTTGGGCGGCCGTTTAAAAAACTCGAGTCGAATCAGGTTGAGGCCAACAAGTTTGCGTTGATCTGGAATG
AAATCATCTTAGCTTTCAGAGAAGAAGATATAGTGTCTGATCGAGAAGTAGAGCTGCTGGAGCTGCCAAA
AAATTCCTGGAATGTGACGGTTATTCGCTGGCCATGTTTCTTGTTGTGCAATGAGCTTTTGCTTGCACTG
AGCCAGGCCAGAGAACTGGTAGACGCTCCTGATAAATGGATGTGGCACAAAATATGCAAGAATGAATACA
GGCGTTGTGCTGTAGTTGAGGCATATGACAGCATCAAACATCTTTTGCTCTCGATCATCAAAACTGACAC
TGAAGAACATTCGATAATTACGGTCTTCTTTCAGATGATTGATCTTTCCATTCAGTCAGAGAATTTCGTC
AAGACCTTTAGAGTAGACCTGCTGCCAAAGATTTACGAAACACTGCAGAAGTTGGTTGGGCTGCTGAATG
ATGAGAAAAAGGATGGTGGTCGGGTGGTGAATGTTCTGCAGTCTCTTTATGAGATCGCAACTCGACAGTT
CTTTACAGAGAAGAAGACAACTGAACAGCTGTCTAATGAAGGGTTGACTCCTCGTGACCCAGCCTCAAAG
TTACTGTTTCAGAATGCTATTAGGCTTCCTGATGCAAGCAATGAAGACTTCTACCGGCAGGTTAGGCGCT
TACACACGATTCTCACCTCTAGAGACTCTATGCACAGTGTTCCCGTGAATTTAGAGGCGAGACGGCGGAT
TGCTTTCTTCAGTAATTCGCTTTTCATGAACATGCCTCATGCACCTCAGGTTGAGAAGATGATGGCGTTC
AGTGTTCTGACTCCATATTACAGTGAGGAAGTTGTATACAGCAAAGAACAGCTCCGAAATGAGACTGAAG
ATGGGATTTCAACCCTATACTACCTGCAGACAATTTATGCTGATGAATGGAGAAATTTCAAGGAACGGAT
GCATAGGGAGGGAATAAAGACAGATAGCGAGTTGTGGACAACCAAGCTGAGAGAGCTCAGGCTTTGGGCT
TCCTACAGAGGTCAGACATTGGCACGTACAGTTCGGGGGATGATGTACTACTACCGAGCTCTTAAGATGC
TCGCTTTTCTTGACTCTGCATCTGAAATGGACATTCGGGAGGGTGCTCAGGAGCTTGGTTCTGTGAGGAG
TTTGCAGGGAGAACTGGGTGATCGATCTGATGGGGTTGTCTCTGAAAACGACCGATCGTCCTTGAGCAGA
GCAAGTAGTTCTGTGAGTACGCTGTATAAAGGCCATGAGTATGGGACTGCATTGATGAAATTCACATATG
TTGTGGCATGCCAGATCTATGGGTCTCAAAAAGCAAAGAAGGAGCCTCAGGCAGAGGAAATTCTGTATCT
GATGAAACAGAACGAAGCTCTCCGTATTGCATATGTGGATGAGGTGCCTGCTGGAAGAGAAGAGACTGAG
TATTACTCTGTTCTGGTGAAATACGATCACCAGTTGGAGAAGGAAGTGGAGATATTCCGAGTGAAGCTAC
CTGGTCCGGTGAAGCTGGGCGAGGGAAAGCCGGAGAACCAGAATCATGCAATGATTTTTACCCGTGGTGA
TGCTGTTCAGACCATTGATATGAACCAAGACAGCTATTTTGAGGAAGCTCTCAAGATGAGAAATTTGCTC
CAGGAGTACAAACATTATCATGGGATCAGAAAACCAACTATTCTTGGTGTCCGGGAGCATATCTTCACAG
GCTCAGTCTCGTCGCTGGCGTGGTTCATGTCTGCTCAGGAGACAAGTTTTGTCACTCTTGGTCAGCGTGT

TCTTGCAAACCCGCTGAAGGTTAGAATGCATTATGGCCACCCTGATGTATTTGACAGATTCTGGTTCTTG
AGTCGAGGCGGCATCAGCAAGGCTTCCAGAGTCATAAATATCAGTGAGGACATCTTTGCAGGGTTTAATT
GCACATTGAGGGGTGGAAACGTCACCCACCACGAGTATATTCAGGTTGGGAAGGGTCGGGATGTTGGATT
GAATCAGATATCCATGTTTGAGGCTAAGGTAGCCAGTGGGAATGGAGAGCAGGTTCTTAGCCGAGATGTG
TACCGGCTCGGTCACAGGCTCGATTTCTTCAGAATGTTGTCCTTTTTCTACACAACGGTCGGGTTTTTCT
TCAACACGATGATGGTCATTCTTACTGTTTACGCTTTCCTCTGGGGCCGGGTTTATCTGGCTCTCAGCGG
GGTTGAGAAGTCTGCTCTAGCGGACAGTACAGACACCAACGCGGCGCTTGGGGTGATCTTGAACCAGCAG
TTCATCGTTCAGCTTGGTCTGTTCACTGCCCTGCCAATGATTGTTGAATGGTCTCTCGAGGAGGGTTTCC
TTCTGGCGATATGGAATTTCATTCGAATGCAGATTCAGCTTTCATCTGTCTTTTACACATTCTCAATGGG
GACCAGAGCTCACTATTTCGGTCGAACTATTCTCCATGGTGGGGCGAAGTATAGAGCTACTGGACGTGGA
TTTGTTGTCGAGCACAAGGGATTTACTGAGAACTACCGATTGTATGCACGCAGTCACTTTGTGAAGGCCA
TTGAGCTTGGGCTGATACTCATAGTCTACGCAACGCACAGTCCGGTCGCCAAAGACTCGTTGATTTACAT
AGCCATGACTCTCACCAGCTGGTTCCTTGTGATTTCATGGATAATGGCCCCATTTGTGTTCAACCCATCA
GGATTTGACTGGCTTAAGACAGTCTACGACTTTGAAGACTTTATGAACTGGATCTGGTACCAAGGCAGAA
TCTCAACGAAATCTGAACAAAGCTGGGAAAAATGGTGGTATGAGGAACAGGACCACCTGAGAAACACCGG
GATGGCAGGAAAAATTGTGGAGATCATCTTGGACCTCAGGTTTTTCTTCTTCCAGTATGGAATTGTTTAC
CAGCTCAAAATTGCAAGTGGATCTACCAGTCTTTTGGTCTACATGTTCTCATGGATATACATCTTTGCTA
TATTTGTGCTCTTCCTAGTAATCCAATATGCCCGTGACAAGTATTCTGCAAAAGCTCACATACGGTACAG
GCTAGTCCAATTCCTCATGATCGTGCTTGCTATACTGGTGATTGTTGCTTTGCTAGAGTTCACGCATTTC
AGCTTCATTGATATCTTCACGAGTCTTCTTGCATTCATCCCAACCGGCTGGGGAATTCTGCTGATCGCAC
AGACTCAAAGGGGTTGGCTGACAAAGTACAGGGTTTTCTGGAACGCTGTGGTCTCTGTTGCGCGCATGTA
TGACATATTGTTCGGGATACTCATAATGGTTCCAGTAGCGTTCTTGTCGTGGATGCCTGGATTCCAATCA
ATGCAAACGAGGATATTATTCAATGAAGCTTTTAGCAGAGGGCTTCGCATCATGCAGATTGTCACTGGGA
AGAAATCAAAAGGCGATGTCTAA
>XM_018600257.1_*Raphanus_sativus*
ATGAGCCTCCGCCACCGCACCGTCCCATCTCAAACCGGACGGCCGTCGGCGGGGGGAACCGAGGAGGAGC
CCTACAACATCATCCCCGTCAACAACCTCCTCGCCGACCACCCTTCCCTCCGCTACCCCGAGGTCCGCGC
CGCCGCCGCCGCTCTCAAAACCGTCGGAGACCTCCGCCGTCCTCCCTTCGTCCAATGGCGCCCTCACTAC
GACCTCCTCGACTGGCTCGCCCTCTTCTTCGGCTTCCAGAAGGACAACGTCCGCAACCAGCGCGAGCACG
TCGTCCTCCACCTCGCCAACGCCCAGATGCGCCTCTCCCCTCCCCCCGACAACATCGACTCCCTCGATCC
CGCCGTCGTCCGCCGCTTCCGCCGCAAGCTCCTCGGGAACTACTCCAGCTGGTGCTCCTACCTCGGGAGG
AAATCGAACATCTGGATCTCGGATCGGAGCCCCGATTCGAGGCGGGAGCTTCTCTACGTCGGTCTCTACC
TCCTCGTGTGGGGCGAGGCCGCGAATCTTAGGTTTATGCCCGAGTGCGTCTGTTACATCTTCCACAATAT
GGCCTCGGAGCTTAACAAGGTGTTGGAGGATTGCCTCGATGAGAACACGGGGCAGCCTTATACGCCTACT
CTCTCTGGGGAGAACGCTTTCCTAAACGGCGTCGTTAAACCTATTTACGAGACGATCCGAGCTGAGATTA
GTGAGAGTAAGAACGGGACGGAGCCGCATTGTAAGTGGAGGAACTATGATGATATTAATGAGTACTTCTG
GACGGATAGGTGTTTCAGTAAGTTGAAATGGCCGCTTGATTTGGGGAGCAGTTTCTTCAAGAATAGTAGA
GGGAAGAGCGTTGGGAAGACTGGTTTTGTGGAGCGGAGGACGTTTTTTTATCTCTACAGGAGCTTTGATA
GGCTTTGGGTGATGCTCGCTTTGTTCCTTCAAGCCGCCATTATAGTTGCTTGGGAGGAGAAGCCGGATAG
AGGGTCGGTGGCAAAGCAGATGTGGAATGCCTTGAAGTCGAGAGATGTTCAGGTGAGGCTTTTGACAGTT
TTCTTGACGTGGAGTGGGATGAGACTGCTGCAGGCTGTGCTGGACGCTGGTTCGCAACGGTCCCTTATTT
CTAGAGAGACCAAGCGGCTGTTTTTCAGGATGTTGATGAAGGTTGTGGCTGCCACGGTTTGGATTATAGC
CTTTATTGTTCTCTACACGAACATCTGGAAGCAGAGGAAGCAAGACAGACAGTGGTCCAGAGCCGCGAAT
GACAAGATCTACCAATTCCTTTACGCCGTGGTGGCATTCTTGGTCCCTGAGATCCTGGCTCTGGCTCTGT
TTATAATCCCGTGGATAAGGAACTTTCTGGAAGAGAGCAATTGGAAAATATTCTTTGCTTTAACTTGGTG
GTTCCAGGGTAAAAGCTTTGTGGGTCGAGGTTTGAGAGAGGGTTTGGTGGACAACATCAAGTACTCGGCG
TTCTGGATCTTTGTCCTAGCAACGAAGTTCACTTTCAGCTACTTCCTGCAGGTTAAACCAATGATTAAAC
CCTCGAAGCTGCTATGGAACTTAAAGGAGGTAGATTATGAGTGGCATCAGTTCTTTGGCGAGAGCAATAG
GTTTTCTGTCTTGTTATTATGGCTGCCAGTGGTGTTGATATACCTGATGGATATCCAAATTTGGTACGCA
ATCTATTCGTCGATTGTTGGTGCTAGTGTTGGGTTGTTTGATCATCTGGGGGAGATCAGGGACATGGGAC
AGCTTAGGCTGAGGTTTCAGTTCTTTGCTAGCGCTATTCAGTTCAACCTAATGCCTGAGGAACAACTCCT
GAATGCTAGAGGCTTTGGTAACAAGCTTAAGGACGCCATTCATAGATTGAAGCTGAGGTATGGATTTGGG
CGGCCGTTTAAGAAACTCGAGTCTAATCAGGTTGAAGCCAACAAGTTTGCGTTGATCTGGAATGAGATAA
TCTTAGCTTTCAGAGAAGAGGATATAGTCTCTGATCGAGAAGTAGAGCTACTGGAGCTGCCAAAGAATTC

GTGGAATGTGTCAGTTATCCGCTGGCCGTGTTTCCTGCTGTGCAACGAGCTTTTACTTGCACTGAGCCAG
GCGAAAGAGCTGGTTGATGCTCCTGATAAATGGCTGTGGCACAAGATATGCAAGAATGAGTACAGGCGTT
GTGCTGTGGTTGAGGCATATGACAGCATCAAACATCTGTTGCTCTCAATCATCAAGATTGACACCGAAGA
ACATAAAATCATTACGATCTTCTTCCAGATGATTGAGGTCTCTATTCAGGGTGAGCAGTTCACCAAGACC
TTTAAAGTGGACCTTCTGCCAAAGATGTACGAGACACTTCAGAAGTTGGTGGGGCTACTGAATGCTGAGA
AGCCAGATATTGAGCGAGTGGTGAATGTTTTGCAGTCGATTTATGAGATTGCAACACGACAGTTCTTCAT
AGAGAAGAAGACAACTGAACAGCTATCTAATGAGGGGTTGACTCCTCATGATCCAGCCTCCAAGTTACTG
TTTCAGAACGCTGTTAGGCTTCCCGATGCAAGCAATGAAGACTTCTTTCGGCAGGTTAGGCGGTTACACA
CAATTCTCACTTCTAGGGACTCTATGCACAGCGTCCCTGTGAATCTAGAGGCGAGACGGCGGATTGCCTT
CTTCAGCAATTCGCTCTTCATGAACTTGCCTCATGCACCTCAGGTGGAGAAAATGTTGGCGTTCAGTGTT
CTGACTCCATACTACAGCGAGGAAGTTGTATACAGCAAAGAACAGCTCAAAAACGAGACTGAGGATGGGA
TTTCAACCTTGTATTACCTGCAGACGATTTATGCTGACGAATGGAAAAATTTTAAGGAACGGATGCGTAG
GGAAGGTATAAAGACAGATGTTGAGTTGTGGACAACCAAGCTGAGAGAGCTCAGGCTTTGGGCTTCCTAC
AGAGGTCAGACTTTGGCACGTACAGTTCGAGGAATGATGTACTATTACAGGGCTCTTAAGATGCTTGCTT
TTCTTGACTCTGCGTCTGAAATGGACATTCGGGAGGATGCTCAGGAGCTTGGTTCAATGAGGAGTTCGCA
GGGAAATGGTCGACTGGATGGGGTTGACGATGTAAATGGTGGATCTTCTCTAAGCAGAGCAACTAGCTCC
GTGAGCATGCTGTATAAAGGCCATGAGCATGGGACTGCATTGATGAAATTCACATATGTCGTGGCGTGCC
AGATCTATGGGTCTCAAAAAGCAAAGAAGGAGCCTCAGGCAGAGGAAATTCTGTATCTTATGAAGCAAAA
TGAAGCCCTCCGTATTGCGTATGTGGATGAGGTGCATGCGGGCAGGGGAGAGACAGAGTATTACTCAGTT
CTGGTGAAATACGATCACACGTTGGAGAAGGAAGTGGAGATATTCCGTGTGAAGCTGCCTGGTCCGTTGA
AGCTGGGTGAGGGAAAGCCAGAGAACCAGAATCATGCAATGATCTTTACCCGTGGTGATGCTGTTCAGAC
CATAGATATGAACCAGGATAATTATTTTGAGGAGGCTCTCAAGATGAGAAATTTGCTCCAGGAGTTTAGA
AAATCTCATGGGATCAGAAAGCCAACTATTCTCGGTGTCCGGGAGCACATCTTCACGGGCTCTGTCTCGT
CTCTGGCGTGGTTCATGTCTGCTCAGGAGACTAGTTTTGTCACTCTTGGTCAGCGTGTTCTTGCCAACCC
GCTTAAGGTCAGAATGCATTATGGTCACCCCGATGTTTTTGACAGATTCTGGTTCTTGAGTCGAGGTGGT
ATCAGCAAAGCTTCCAGAGTCATAAATATCAGTGAAGACATCTTCGCCGGGTTTAATTGCACATTGCGAG
GCGGTAACGTCACCCACCACGAGTATATTCAGGTTGGGAAGGGCCGGGATGTTGGATTGAATCAAATATC
AATGTTTGAGGCTAAGGTAGCCAGTGGGAATGGAGAGCAGGTTCTTAGCCGAGATGTGTACAGGCTGGGT
CATAGGCTCGATTTCTTCAGAATGTTATCATTTTTCTACACAACGGTCGGGTTTTTCTTCAACACGATGA
TGGTCATTCTTACGGTTTACGCTTTCCTCTGGGGCCGGGTTTATCTCGCGCTGAGCGGTGTTGAGAAGTC
CGCTCTAGCAGACAGTACAGACACCAACGCAGCGCTTGCTGTGATACTGAACCAGCAGTTCATCGTTCAG
CTTGGTCTGTTCACTGCCCTGCCAATGATTGTGGAATGGTCTCTCGAGGAGGGTTTCCTTCTAGCTATAT
GGAATTTCATTCGGATGCAGATTCAGCTTTCCTCTGTCTTCTACACATTCTCAATGGGGACCAGAGCTCA
CTATTTTGGTCGAACCATTCTCCATGGTGGAGCAAAGTACAGAGCCACTGGACGTGGATTTGTTGTCGAG
CACAAGAGTTTCACCGAGAACTACCGACTCTATGCACGCAGTCACTTTGTGAAGGCCATCGAGCTTGGGC
TGATCCTCATAGTCTATGCTACGCACAGTCCCATCGCCAAAGACTCATTGATCTACATAGCCATGACTCT
CACCAGCTGGTTCCTCGTGATATCATGGATACTGGCCCCATTTGTGTTCAACCCGTCAGGATTCGACTGG
CTTAAGACGGTCTATGACTTCGAAGGCTTCATGAACTGGATCTGGTATCAAGGCAGAATCTCAACGAAAT
CGGAACAGAGCTGGGAGATATGGTGGTATGAGGAACAGGACCACCTGAGAACCACCGGTATACCAGGAAG
AGTTGTGGAGATAATCTTGGACCTTCGGTTTTTCTTCTTCCAGTACGGGATTGTTTACCAGCTCAAAATC
GCAAAGGGATCAACCAGCATTCTCGTCTACTTGTTGTCATGGATATACATCTTTGCAGTGTTTGTGTTCT
TCCTGATAATCCAGTACGCCCGTGACAAGTACTCTGCGAGAAACCACATACGGTACAGGCTCGTTCAGTT
CCTCCTGATCGTGTTTGGTATTCTGGTGATTGTTGCTTTGCTAGAGTTCACGCATTTCAGCTTCGTGGAT
ATCTTCACGAGTCTTCTTGCGTTCGTCCCAACCGGATGGGGAATCTTGCTGATCGCACAGGCTTTAAGGC
CAGCGCTGACGAAGTTTAGGCTTATCTGGAACGCGGTTGTCTCCCTTGCTAGGGTATATGACATACTGTT
CGGGGTACTCATCATGGTTCCCGTAGCGTTCATGTCGTGGATGCCTGGGTTTCAATCAATGCAAACAAGG
ATCTTATTCAATGAAGCCTTTAGCAGAGGGCTTCGTATCATGCAGATTGTCACTGGGAAGAAATCAAAAG
GCGATGTCTAA
>XM_017391380.1_Daucus_carota_subsp._sativus
ATGAATGTCCGACGACCTGGTCAGACCCGGCCCGACCCGAACCCGCCCCAACCCTACAACATCATCCCCA
TCCACGACCTACTAGCTGACCACCCCTCTCTCCGCTTCCCCGAAGTACGCGCCGCCGCCGCCGCCCTACG
CGCCGTCGGAGACCTCCGCAAACCTCCCTTTGCGCCATGGCTCCCCCACATGGACCTCCTCGACTGGCTC
GGCGCTTTTTTCGGCTTCCAAAACGACAACGTTCGCAACCAGCGCGAGCACATAGTCCTTCACTTGGCCA
ACGCTCAGATGCGCCTGACCCCACCCCCCGACAATATCGATTCACTGGACCCCACTGTGCTCCGACGTTT

TCGCCGGAAATTGCTCAAGAACTACTCTGATTGGTGCTCGTTTCTTGGGCGGAAGCCAAACATCTGGATC
TCCGATAGCTCCCGTGGGGCCCACTCCGATCAAAGGCGCGAGCTTTTGTATGTATCTCTCTACTTGCTTA
TTTGGGGCGAGTCTGCGAATTTGAGATTTATGCCAGAATGTATTTGTTTTATTTTTCATAATTTGAGTAT
GGAGCTTAATAAGATTCTTGAGGATTACATTGATGAGAATACGGGGAGGCCGATTTTGCCTAGTGTGTCG
GGGGAGAATGCGTTCTTGATAAAGATTGTTACGCCTATTTATGATACGATTAAGGCTGAGGTAGAGAATA
GTAGGAATGGGACTGCCCCTCATTCGAATTGGAGGAATTATGATGATATCAATGAGTACTTTTGGAGTAG
GAGGTGTTTCGATAAGTTGAATTGGCCACTTGATCGGGGGAGTAATTTTTTTGTGGCAGATGTTAGGGGT
GGGAAGAGGGTAGGGAAGACGGGGTTTGTGGAGCAGAGGTCATTTTGGAATTTGTTTAGGAGTTTCGATA
AGCTTTGGATTATGTTGATTTTGTTTCTTCAGGCTGCGATTATTGTGGCGTGGGAGGAGAGGGAGTATCC
GTGGCAGGCATTGGAGGAGAGGAGTGTTCAGGTTACGCTTTTGACTGTGTTTATTACGTGGAGTGGGTT G
AGGTTCTTGCAGTCAATTCTTGATGCTGGAATGCAGTATAGTTTAGTTACGAGGGAGACTATGGGGCTTG
GTGTGAGGATGGTGTTGAAGAGTGTTGTTGCTGCTATATGGATTCTAATCTTTGGTGTTTTCTATGGGAG
GATAGTGAAGGAGAAGAATAAATATGGGAAATGGGAAAAACATGAGGTGAACAATCAGGTGGTTACTTTT
CTTGAGATTGCATTAGTATATGTCATCCCGGAACTTCTGGCACTTTCGCTTTTTATAGTACCGTGGATCA
GAAATTTCATCGAGAATCGAAATTGGAGGATCTTTTACGTGTTGTCATGGTGGTTTCAGAGCCGGTCATT
TGTGGGGAGAGGCCTTAGAGAAGGACTTATGGACAATGTGAAGTATACTTTGTTCTGGATTGTTGTCCTT
GCCACAAAATTTTGCTTCAGTTACTTTTTGCAGATCAAACCGATGGTTAACCCGACACAGGCCTTACTGG
ATATGAAAGATGTGACTTATGAGTGGCACCAGTTCTTTGGGAAAAGCAACAGATTTGCTGTTGGAATATT
GTGGCTTCCAGTGGTGTTAATATACTTAATGGATATACAGATTTGGTACTCCATATACTCGTCATTTGTT
GGTGCAGGAGTTGGGTTGTTCCAGCACTTGGGCGAGATCCGAAACATGCAGCAGTTGAGATTAAGATTCC
AATTCTTTGCTAGTGCGATTCAGTTCAATCTCATGCCTGAGGAACAACTATTGAATGCCAGGGGAAGTCT
CAAGAGCAAGTTTCGGGATGCAATTAACAGGTTGAAGCTAAGGTATGGCCTTGGCCGACCCTTTAAGAAG
CTTGAATCGAGCCAGGTAGAAGCAAACAAATTTGCATTGATATGGAACGAGATAATCTTGACATTTAGGG
AAGAAGATATCTTAAGTGATAAGGAGGTGGAGTTGTTGGAGTTGCCACAGGATACCTGGAATGTTAGGGT
CATTCGATGGCCATGTTTGCTTCTCTGCAATGAGCTGCTTCTCGCTCTAAGCCAAGCCAAGGAGTTAGTA
GATGCTCCTGATAAGTGGCTCTGGTACAAAATTTGCAAGTATGAGTACAGGCGTTGTGCTGTCATTGAAA
CTTATGACTGTGTTAAGCACTTGTTGCTGCATATTATTAAATATGACGTGGAGGAACATTCTATTGTTAC
GGTCTTATTTCAAGAAATTGATCACTCGTTGCAGAATGAAAAGTTTACAAAAACATTCAATCTGAAGGCA
CTTCCCCGGATTCATGCAAAGCTGATCATCCTCCTCGACCTGATAATCAAGCCTACTAAAGATGTCAACA
AGATTGTGAACATTCTGCAGGCCCTGTATGAGACTGCTATTCGAGATTTTTTTAAAGAAAAAAGGAATGC
AGATCAGCTGAGGGAGGATGGTTTGGCTCCAAGAAGGGCAGTTTCGGGTGACAGGTTGCTATTTGAGAAT
GCTATTGAATTGCCAGATACAGATCATGAAACTTTTTACCGGCAAGCTCGACGCCTTCACACTATTCTGA
CATCTCGGGATTCGATGAATAACGTCCCGAAAAATCTAGAGGCTAGACGTCGTATTGCCTTTTTCAGTAA
CTCATTGTTTATGAACATGCCTCATGCTCCCCAAGTCGAGAAAATGATGGCTTTCAGTGTTTTGACCCCG
TATTACAATGAAGAAGTGCTATATAGTAAGGAAAACCTTCGAACTGAAAATGAAGATGGTATTTCCACCT
TGTATTACTTGCAGACAATTTATGCTGATGAATGGGAGAATTTCTTGGAGAGAATGCGACGAGAAGGTTT
GGCTAAAAATGGTGAGATATGGACTGACAAGCTCAGAGACCTGCGCCTTTGGGCGTCATACCGAGGCCAG
ACACTGGCCCGTACTGTGAGGGGAATGATGTATTACTATCGTGCTCTCAAAATGCTCGCTTTTCTGGATT
CTGCTTCAGAGATGGATATCAGGGAAGGGTCGCGGGAACTTGCTTCGATGGGGCGTAGCCGTAGCTTAGA
TAGTTTCAACTCAGAAAGGTCGCAATCTGCAAGAAGCCTGAGTAGGGCAGACAGTACAGTTAACTTGTTG
TTTAAAGGCCATGAGTATGGTACTGCTCTGATGAAATACACCTATGTGGTTGCCTGCCAGATATATGGGA
CCCAAAAGGCGAAGAAGGACCCCCATGCTGAAGATATATTGTATCTGATGAAAAACAACGAGGCTCTTCG
GGTTGCTTATGTCGACGTGGTTCCCTCAGGGAGGCCTGATCCGGATTATTACTCTGTTTTGGTAAAATAT
GACCAACAGTTGGAGAAGGAAGTGGAGATATATAGAGTTAAGTTACCTGGTCCATTAAAGCTTGGGGAGG
GAAAGCCAGAGAATCAAAACCATGCTCTTATCTTTACTAGAGGAGATGCAGTTCAGACCATTGATATGAA
CCAGGACAGCTATTTCGAGGAGGCACTTAAAATGCGAAATCTTCTGGAGGAGTACAGGCACTATTATGGT
TTGAGAAAGCCGACTATTTTGGGAGTCCGGGAACACATATTTACTGGTTCTGTCTCCTCGCTTGCTTGGT
TTATGTCAGCCCAGGAGACTAGTTTTGTCACATTGGGACAACGTGTTTTGGCGAACCCCCTTAAAATACG
CATGCACTATGGTCATCCGGATGTATTTGATAGATTCTGGTTCTTAACTAGGGGAGGAATAAGCAAAGCG
TCAAGGGTGATCAATATCAGTGAGGACATATTTGCAGGCTTCAGCTGCACATTGCGAGGTGGGAATGTTA
CTCATCATGAATACGTGCAAGTTGGCAAGGGGAGGGATGTTGGACTGAATCAAATTGCGATGTTTGAAGC
CAAAGTTGCTAGTGGAAATGGGGAGCAGGTTCTTAGCAGAGAGGTCTATAGGTTGGGACATAGGCTGGAT
TTTTTCAGAATGCTATCATTTTTCTACACTACTGTAGGGTTTTTCCTGAGTACAACAATGATCATCCTCA
CTGTTTATGCATTCCTGTGGGGTCGGCTTTATCTTGCTTTAAGTGGGATTGAGAGTTCTAATGCAGCAGA

TGATGCTGCGGACAATAAAGCACTTGGTGCTATATTAAACCAACAGTTCATTCTCCAACTTGGCCTGTTC
ACTGCGTTGCCAATGATAGTAGAAAATTCTTTGGAGCATGGTTTTCTTTCAGCAATTTGGGATTTTATAA
CTATGCAACTTCAGCTGTCATCTGTATTTTTCACATTTTCCATGGGAACTCGGGCTCACTATTTTGGCCG
AACTATTCTTCATGGTGGTGCAAAGTACAGGGCAACTGGTCGTGGTTTCGTGGTGGAGCACAAAAGCTTT
GCTGAAAACTATAGACTTTATGCGCGTAGCCACTTTGTAAAGGCTATTGAGCTTGGGCTGATACTTGTAG
TGTATGCCTCTTACAGTGCTGTAGCTAAAGGAACTTTTGTTTACATTGCGTTGACCATCTCAAGTTGGTT
CCTAGTGATTTCATGGATGATGGCCCCTTTCTTGTTCAATCCATCTGGTTTTGATTGGTTGAAAACGGTG
TATGACTTTGATGATTTCATGAACTGGATATGGTTCCGCGGTGGTGTGTTTGCTAAAGCTGATCAGGGCT
GGGAAAGATGGTGGTATGAGGAGCAGGATCACTTGAGAACAACAGGTCTTTGGGGAAAATTTCTGGAAAT
TATTTTGGACCTGCGGTTTTTCTTTTTTCAGTATGGGATTGTATACCAACTTGGTATTTCTGATGGGAGC
AACAGCATATTTGTTTACTTGCTTTCGTGGATCTATGTGGGCATGGCACTTGCAGTTTACTCTACTGTAG
CTTATGCTCGGGATAAATATGCTGCAAGAGAACATATCTATTATCGATTGGTTCAGTTTCTCCTTATAAT
CATTTTTGTGCTTGTGATCATTGCCTTGCTCCAGTTCACCGAGTTCAAATTTGTGGATATTTTCACTAGT
CTGTTGGCATTTTTACCCACAGGTTGGGGTTTTATTTCGATAGCTCAAGTGTTGCGACCCTTTTTGGAGA
ATACTATGATTTGGGAAACTGTTGTTTCTGTTGCTCGCTTGTATGATATATTGTTTGGAGTGATTGTTAT
GACCCCTGTGGCGGTGCTATCATGGTTGCCTGGATTCCAGTCTATGCAAACAAGGATTTTATTTAATGAA
GCATTTAGTAGGGGATTGCAGATAACCAAGATCGTGACAGGAAAAAAATCTTCTGATATGTGA
>XM_010325437.3_*Solanum_lycopersicum*
ATGAGCCTCCGGCAACGTTCAACGCCGGCGGCGAGACAAGTTTCTATAGATGAAGAACCATATAACATCA
TTCCGATTCATAATCTTCTAGCTGACCATCCTTCTCTACGTTTCCCTGAGGTACGCGCTGCGGCGGCGGC
TTTACGCTCTGTAGGTGACCTAAGGAGACCTCCATTTGCACCGTGGAAACCTCACTATGACCTGCTTGAC
TGGCTGGCGCTGTTCTTCGGGTTTCAGGATTCTAGTGTTCGTAACCAACGGGAACATATCGTGCTTCATC
TTGCTAATGCTCAGATGCGTTTATCTCCGCCGCCGGACAATATTGACTCTCTTGACCCTGCTGTTCTCCG
TCGGTTCCGACGTCAGCTTCTGAAGAATTACTCGTCGTGGTGCTCTTTTCTCGGTCTCAAATCTAATGTT
TGGCTTTCCGACCGGCATAACTCATCTGACCACCGCCGTGAGTTGCTTTATGTCTCGCTTTACCTTCTTA
TATGGGGTGAGTCAGCGAATCTACGTTTTGTTCCTGAATGTTTATGCTTTATTTTTCATAATATGGCTAT
GGAATTGAATAAGATTTTGGAGGATTACATTGATGAGAATACTGGTAGGCCATTTTTGCCATCGATATCT
GGTGAAAATGCTTTTCTGAATCGGATCGTAACGCCAATTTACCAAACAATCCGAGCTGAGGCTGATAATA
GTCGGAATGGTACTGCCCCACACTCTGCGTGGCGGAATTACGATGACATCAATGAGTATTTCTGGACTAA
AAGGTGTTTTGATAAGTTGAAGTGGCCTATTGATATTGGGAGTACATTTTTTGTGACCACTAACAAGGGA
AAGAAGGTTGGAAAGACAGGGTTTGTGGAGCAGAGATCATTTTTGAATTTGTATAGGAGTTTTGATAAGC
TATGGATCATGCTGGCGTTGTTTTTGCAGGCTGCAATTATTGTAGCTTGGGAAGGGAAGCCTTATCCGTG
GCAGGCTTTGGAGAGTAGGGAGGTTCAGGTGAGGGTGTTAACTATCTTCTTCACCTGGAGCAGTATGAGA
TTTCTGCAGTCGTTACTTGATGCAGGAATGCAATATCGTATCATCTCTAGGGAGACCCCGTGGCATGGGG
TGAGAATGGTGTTGAAGAGTGTGGTTGCAGCTGCGTGGATTGTGGTCTTTGGTGCATTCTATGGGAGGAT
TTGGATCCAGAGGAATAGGGATGGGAAATGGAGCAGTGCTGCTAACAGGAGGGTAGTGAATTTTCTTGAG
GTTGCTCTTGTTTTCATTGCTCCAGAACTGTTAGCCCTGGCACTCTTTGTTCTGCCATGGGTCAGGAATT
TTCTCGAGAACACGAACTGGAGGATATTTTACCTGTTGTCCTGGTGGTTCCAGAGTCGAACGTTTGTGGG
TCGTGGACTCAGGGAAGGCCTTGTTGATAACATTAAGTATTCCCTCTTTTGGGTAGTAGTGCTCGCGACC
AAGTTTTCCTTCAGTTACTTCCTACAGATCAAACCTATGATCGTTCCAACAAGAGCACTGTTGCGCCTCA
GGGATGTGAAGTACGAATGGCATGAATTCTTTAACCATAGCAACAGGTTCTCAGTAGGATTGCTTTGGCT
TCCTGTTGTACTGATTTATCTCATGGATATTCAGATATGGTACTCAATCTACTCTTCTTTTGTTGGGGCA
GCGGTTGGATTATTTGATCACTTGGGAGAGATTCGAAACATGCCGCAGTTAAGGTTGAGATTTCAATTTT
TTGCAAGTGCAATGCAGTTTAATCTGATGCCAGAAGAGCAGTTGTTGAATGCTCAAGGAACACTAAAAAG
CAAGTTCAAGGACGCCATCCTCCGTTTGAAACTCAGATATGGGTTTGGTCGACCATTCAAAAAGCTTGAA
TCAAACCAGGTAGAGGCGAACAAATTTGCCTTGATTTGGAATGAGATAATTACAACTTTCAGAGAAGAAG
ATATTCTGAATGACCGTGAGGTTGAGTTGTTGGAGCTGCCCCAGAACACATGGAATGTTAGAGTGATTCG
TTGGCCATGTTTGCTCCTCTGCAACGAGGTGCTGCTTGGTCTCAGCCAGGCGAAGGAGCTGGTGGATGCT
CCTGATAAGTGGCTCTGGCATAAGATCAGCAAGTATGAGTACAGACGATGTGCTGTTATTGAGGCTTATG
ACAGTACAAGGCATTTGCTGCTGGAAATTGTGAAATTGAACAGCGAGGAGCATTCCATCATAACAACCTT
TTTTCAGCAGATTGATCAGTGGATTCAGCTGGAGAAATTCACAAAATACTATAATCTGACTGCTCTGCCC
CAGATCCGTGGAAAGTTGATTGCTCTTCTGGATCTATTACTTAAGCCAAAAAAGGATGTTGACAAGATTG
TGAATGTTCTCCAGGCCTTATATGAGGTTGCCACTCGGGATTTTCTGAAAGAGAAGATGACTGGAGATCA
GCTGAGAGAGGAAGGTCTGGCTCTTCAGGCATCTGCAACTAGATTGCTTTTTGAGAATGTAGTTTCATTG

CCTGATCCAGAGAATGAGACATTTTATCGGCAAGCTCGCCGCTTGAACACTATTCTTACATCTCGGGACT
CTATGAGTAATATCCCAAGAAATCTTGAGGCGAGACGTCGACTTGCCTTCTTTAGCAATTCTCTATTTAT
GAATATGCCACATGCGCCCCAAGTTGAGAAGATGATGGCTTTCAGTGTTTTGACACCTTACTACAATGAA
GATGTACTGTACAACAAGGAACAACTCAGAACTGAGAATGAAGATGGGATTTCTACATTATATTACTTGC
AGACTATTTATGCTGATGAGTGGGAAAATTTCTTGCAGCGAATGCGTAGAGAAGGAATGGTTGATGAGAA
GAAAGAGTTATGGACTACAAAGCTAAGGGATCTTCGTCTTTGGGCATCATACAGAGGGCAGACTCTTACT
CGCACGGTTAGGGGGATGATGTACTACTATCGAGCTCTCAAAATGCTGGCCTTTCTGGATTCTGCTTGTG
AGATGGATATCAGAGAAGGATCAGTGGAACTTGGTTCTATGAGGCATGATGATAGCATTGGTGGTTTAAG
TTCAGAAAGATCTCAGTCTTCGAGGAGGTTGAGTAGAGCTGACAGTTCAGTGAGTATGTTGTTTAAAGGC
CACGAGTATGGGACTGCTTTAATGAAATTCACATATGTGGTAGCTTGTCAGATATATGGGGCTCAGAAGG
CCAAAAAAGATCCACATGCAGAGGAAATTTTGTATCTGATGAAAAATAATGAAGCTCTTCGTGTAGCTTA
TGTTGATGAGGTTCCCACAGGAAGGGATGAGAAGGATTATTATTCTGTGCTTGTGAAGTATGATCAAAAA
CTTGAAAGGGAAGTTGAGATCTATCGAGTTAAGTTGCCTGGTCCTTTGAAGCTTGGGGAGGGAAACCAG
AAAATCAAAATCATGCCTTTATCTTTACCCGTGGTGATGCAGTTCAGACTATTGACATGAACCAAGATAA
TTACTTTGAGGAGGCACTGAAAATGAGGAACTTGTTGGAAGAATTCAAACTCTACTATGGTATTCGCAAA
CCTACGATTCTTGGAGTTCGAGAACATATATTTACTGGTTCCGTGTCATCCCTTGCTTGGTTCATGTCAG
CTCAGGAAATGAGTTTTGTAACCCTAGGACAGCGTGTATTAGCCAACCCCCTGAAAATCCGAATGCATTA
TGGACATCCAGATGTATTTGACAGGTTTTGGTTTCTAACTAGGGGAGGAATAAGCAAGGCATCTAAAGTG
ATCAACATCAGCGAGGACATTTTTGCTGGCTTCAACTGTACATTACGAGGTGGCAATGTCACTCACCATG
AGTATATACAAGTTGGCAAAGGAAGGGATGTTGGGTTGAATCAGATATCTATGTTTGAAGCCAAGGTTGC
CAGTGGCAATGGAGAACAAGTTCTTAGCAGAGATGTCTATAGGTTGGGTCATAGGCTGGATTTCTTCAGA
ATGCTTTCTTTCTTTTATACAACTGTAGGATTCTTCTTCAATACAATGATGATTGTCCTCACTGTATATG
CATTCTTATGGGGACGACTTTACCTGGCACTTAGTGGGGTTGAGGGCTCTGTTGCTGCAGATACCACCGA
CAACAACAGAGCACTTGGTGCCATACTGAACCAGCAATTTATCATCCAGCTGGGCCTTTTCACCGCATTA
CCAATGATTGTGGAGAACTCTCTCGAGCATGGTTTTCTTACATCTATCTGGGAATTTCTTACAATGATGC
TCCAACTTTCATCTGTATTTTACACATTCTCAATGGGAACTCGTGCTCATTACTTTGGTCGTACCATTCT
CCATGGTGGTGCAAAATACCGGGCAACTGGGAGAGGTTTTGTCGTGCAGCACAAGTGTTTTGCTGAGAAT
TATCGGTTATATGCTCGTAGCCATTTTGTCAAGGCAATTGAACTTGGTTTGATACTTACAGTGTATGCTG
CATACAGCCCTGTTGCTAAAGGAACTTTTACATATATAGCACTGACTATATCAAGTTGGTTCCTGGTGGT
GTCATGGATCTTGGGGCCCTTTGTGTTTAATCCTTCTGGGTTTGATTGGCTAAAGACAGTGTATGATTTT
GATGACTTCATGAACTGGATTTGGTACCGTGGTAGTGTTTTTGCGAAGTCAGACCAGAGCTGGGAGAAAT
GGTGGGAGGAGGAACAGGATCATTTAAGAACGACAGGTCTGTGGGGAAAGATACTGGAAATTATCCTAGA
CCTCCGCTTCTTCTTTTTCCAGTATGGCATTGTATATCATCTGGGTATTGCTGCTGGAAGCAAAAGCATT
GCTGTTTACTTGCTTTCATGGATTTATGTGGTGGTGGCTCTTGGCTTTTTTAATATTACAGCTTATGCTC
GGGAAAAATATGCTGCACGGGAGCACATATACTTTCGCCTTGTGCAGCTCCTTGCTGTACTCTTTTTCAT
AGTTGTAATTGTTGCTTTACTGCAGTTCACAGCATTTAAATTTGGTGATCTCTTTGTCAGCCTGTTGGCT
TTTGTTCCTACTGGTTGGGGCTTCATTTCAATCGCCCAAGTGTTACGTCCCTTTTTGCAGAAGAGTATGA
TATGGGGAACTGTTGTGTCTGTGGCGCGACTATATGAGATAATGTTTGGGATTATTGTCATGGTACCTGT
TGCAGTACTGTCTTGGTTGCCTGGTTTCCAACCAATGCAGACAAGGATCCTATTCAATGAAGCATTTAGT
AGAGGTCTGCGGATATTCCAGATTGTGACAGGAAAAAAGCCTAAGAGTGACGTGTGA
>XM_010658666.2_*Vitis_vinifera*
ATGAGCCTCAGACAGCGCCCCCCGGCGGCTGCCGGTTCGCAGTACGGGGCGAACCGGTCGTCTCAGCCAC
CGAACCCGGAAGAAGAAGCGTACAATATCATTCCCATTCACAATCTCATCGCCGACCACCCCTCACTCCG
CTACCCGGAGGTACGAGCCGCGGCGTATGCCCTACGCGCTGTCGGGAGTCTCCGAAAGCCGCCGTTCGGA
GCGTGGCACGAGCACATGGACCTCCTCGACTGGCTCGGCCTCTTCTTCGGCTTCCAGAGCGACAACGTTC
GGAACCAGCGAGAGCACCTAGTGCTCCACCTCGCTAACGCTCAGATGCGGCTCCAGCCACCGCCGGACAA
TATCGACACTCTCGACCCCGGCGTGCTCCGGCGATTCCGACGGAAGCTGCTAAGCAACTACTCGGCATGG
TGCTCCTTCCTCGGCCGGAAATCGAACGTTTGGATCCGAGATTCTGCGCCCGATCCGCGTCGTGAATTGC
TCTACACCGGCCTCTATCTGCTCATCTGGGGTGAGTCTGCTAATCTTAGGTTCATGCCTGAATGTATTTC
CTACATATTTCATCACATGGCTATGGAACTTAATCGAATTCTTGAGGATTACATTGATGAAAACACGGGT
CAGCCTGTACTGCCGTCAATTTCGGGTGAGAACGCGTACCTGGCCCGTGTGGTTAAGCCCATTTACGAGA
CTGTGCATAATGAGGTTGAGCGAAGCAAAAATGGGACTGCCCCACATAGTGCTTGGAGGAATTACGATGA
TATAAATGAGTATTTTTGGAGCCCTCGGTGTTTTCAGAAGCTCAAGTGGCCAATGGATTTGGGGAGTAAT
TTTTTTGCTCTGTCCAGTAAGAGTAAGCATGTGGGTAAGACTGGTTTTGTGGAGCAAAGGTCATTTTGGA

ACTTGTTTAGGAGCTTTGATCGGCTTTGGGTCATGTTGATTTTGTTTCTTCAAGCTGCCATTATTGTTGC
TTGGGAGGGTAAGGAGTATCCATGGCAGGCATTGGAGAGTCGTTATGTTCAGGTCCGGGTTCTCACTGTG
TTTTTCACTTGGAGTGCTTTGCGGCTTCTCCAGTCTCTACTTGATGCTGGGATGCAATATAGCTTGATTT
CTAGGGAGACTTTGTGGCTTGGGGTGAGGATGGTGATGAAGACTGTAGTTGCTGCAGGGTGGATTATTGT
GTTTGCGGTATTTTATGCGAGAATTTGGACGCAGGAAAACAATGATGGTGGGTGGACAAGCAAAGGGAAT
GCACGGGTGGTGAATTTTCTTGAGGTTGCATTGGTTTTCATCCTCCCGGAGCTTTTGGCATTGGCTCTGT
TTATTGTTCCATGGATAAGGAATTTTTTGGAGGAGAAAAATTGGAGGATCTTTTACTTATTATCATGGTG
GTTTCAGAGTAGAATTTTTGTGGGCCGTGGGTTGAGGGAAGGTCTTGTGGATAATATAAAGTATTCTTCA
TTCTGGATTTTGGTGCTTGCTACAAAATTTTCATTCAGCTACTTCCTGCAGATTAAACCTATGGTTGCCC
CTTCCAAAGCCCTTTTGAGAATTAAGAATTTAGAATACGAGTGGCATGAGTTCTTTGATAACAGCAATAG
ATTAGCAGTGGGGCTATTGTGGCTTCCTGTGGTTTTGATGTACCTCATGGATTTGAATATTTGGTACTCC
ATTTACTCCTCTTTCTATGGGGCAGTAGTTGGGCTGTTCTCACATTTGGGTGAAATTCGAAACATTCAGC
AACTGAGACTGAGATTCCAGTTCTTTGCAAGTGCAATCAAGTTCAATCTTATGCCAGAGGAGCAACTGTT
ACATGGAAGGAACATGAGGAACCGATTCAATGATGCTATCCACAGATTGAAGCTACGGTATGGGCTGGGC
CGGCCCTACAAGAAGCTGGAATCTAATCAGGTCGAAGCGACCAAGTTTGCTTTGATATGGAATGAGATAA
TTTCAATTTTCAGGGAAGAAGATATTATCAATGACCATGAGGTCGAGCTGTTGGAACTTCCTCATAACTC
TTGGAACGTTAGGGTTATTCGCTGGCCATGTTTGCTCCTTTGCAATGAGCTGCTGCTAGCTCTCAGCCAG
GCAAAAGAATTGGTAGATGCTCCTGATAAGTGGCTTTGGTATAAGATTTGCAAGAATGAGTATAGACGCT
GTGTTGTCATTGAGGCCTATGATAGTATCAAGCACTTACTGCTTCAGATCATCAAGTTTGATACTGAAGA
GCACTCCATTATCAAGGTCTTGTTTCAAGAAATTGATCACTCTCTTGGTATTGAGAAGTTTACTAAAACA
TTCAAAATGACTGCTCTGCCCCAGATCCATCTCAAGTTGATATCTCTTCTTAAGCTATTGAATGAGCCTA
AGAAAGATCCTAACAAGGTGGTGAACATTTTACAGGCCCTTTATGAGATTGTAATTCGGAATTTTTTCAA
GGATCAAAGGACCAGTGATCAGCTGAGGGAGGATGGTCTGGCTCCTCGTAATCTGTCTTCCAGCACGGGC
CTCCTCTTTGAAAATGCTGTGGAGTTGCCTGATGCCAATAATGGGACCTTCTATCGGCAGGTACGACGCT
TGCACACAATTCTTACTTCTCGTGACTCAATGAACAATATCCCAAAAAATCTGGAGGCAAGGCGCCGGAT
TGCCTTCTTTAGCAATTCCCTGTTCATGAACATGCCCCATGCTCCTCAAGTTGAGAAGATGATGGCTTTC
AGTGTGCTGACCCCTTATTACAATGAAGAAGTACTATACAGTAAAGAACAACTTCGAACTGAGAATGAAG
ATGGGATTTCCATCCTATATTATTTACAAACAATTTATGATGATGAGTGGAATAATTTCTTGGAACGGAT
GAAGCGAGAAGGAATGAAGGATAAAAATGACTTATGGATAACAAAGCTGAGAGATCTCCGTCTATGGGCC
TCTTTTAGAGGCCAGACGCTCACCCGCACTGTAAGGGGAATGATGTATTATTATCGAGCTCTCAAGATGC
TGGCCTATCTCGATTCTGCATCAGAAAAGGACATTGAAGAAGGATCACACGAACTTGGCTCAGTGAGGCG
AAACAACAGTATTGATGGTTTTAATTCTGAAAGATCACCTTCTTCAAGGAGCTTAAGTAGAGCAAGTAGT
TCAGTGAGTTTGTTATTCAAAGGCCATGAGTATGGGACTGCTTTAATGAAATACACATATGTAGTTGCTT
GCCAGATATATGGGTCTCAGAAGGCAAAAAAAGATCCCCATGCTGAAGAAATTTTGTATTTAATGGAACA
CAATGAAGCCCTTCGAGTTGCCTATGTTGATGAAGTTTTGAAAGGGAGGGATGAGAAAGAATATTACTCT
GTTCTTGTGAAGTATGATCAGCAGCTACAGAAGGAAGTGGAGATCTATCGGGTCAAGTTGCCTGGTCCCT
TGAAGCTAGGAGAGGGAAAACCAGAGAATCAGAACCATGCCCTTATCTTCACTCGTGGGGATGCAGTCCA
GACGATTGATATGAACCAAGACAACTATTTTGAAGAAGCACTCAAAATGCGGAATCTATTGGAAGAATAC
AGGACCTACTATGGTATTAGGAAGCCTACTATTCTAGGAGTCCGGGAACACATTTTTACTGGTTCTGTAT
CATCACTTGCTTGGTTTATGTCTGCCCAAGAAACAAGTTTTGTTACATTGGGACAGCGTGTTTTGGCGAA
TCCTTTGAAGATTCGGATGCATTATGGTCACCCAGATGTGTTTGATAGGTTTTGGTTTCTGACCAGGGGA
GGGATTAGCAAAGCTTCCAGAGTGATTAATATTAGTGAGGACATTTTTGCTGGCTTCAACTGCACATTGC
GAGGTGGAAATGTTACACACCATGAATACATCCAAGTTGGAAAGGGAAGGGATGTTGGGTTGAATCAAAT
ATCCATGTTTGAAGCAAAGGTTGCTAGCGGAAATGGTGAGCAAGTTCTCAGCAGAGATGTCTATAGGTTG
GGTCACAGGTTGGATTTTTTACGTATGCTGTCATTCTTCTACACTACAGTGGGATTCTTTTTCAACACGA
TGCTGGTGGTTCTGACTGTTTATGCTTTTTTATGGGGCCGGCTGTATCTGGCTCTTAGTGGGGTTGAGGG
TTCTGCTCTGGCGGATAAAAGCAGCAACAATAAGGCCCTTGGTACAATCTTGAATCAGCAGTTCATAATC
CAGCTTGGTCTGTTTACTGCACTTCCGATGATTGTGGAAAATTCTCTTGAGCATGGGTTCCTTGCAGCCA
TCTGGGATTTCATAACAATGCTGCTCCAGCTTTCTTCTGTCTTCTACACGTTCTCCATGGGAACTCGCAC
TCACTTTTTTGGCCGGACTATTCTTCATGGTGGTGCAAAATACCGAGCAACTGGACGTGGTTTTGTCGTG
CAGCACAAGAGTTTTGCTGAGAACTATAGACTCTATGCTCGTAGCCATTTTGTAAAGGCAATTGAGCTTG
GCTTGATCCTTACAGTTTACGCTGCATACAGTGTCATAGCTACTGACACTTTTGTCTATATAGCCATGAC
CATCACGAGTTGGTTCCTGGTAGTGTCATGGATCATGGCCCCCTTTGTATTTAATCCTTCTGGGTTTGAT
TGGTTGAAGACAGTGGACGACTTCGATGATTTTATGAACTGGATATGGTACCGTGGAGGTGTGTTTGCAA

AGGCTGAGCAAAGTTGGGAAAAAATGGTGGAATGAGGAGCAGGATCATCTAAGGACAACTGGTCTTTGGGG
AAAGTTACTGGAGATAATCTTAGATCTTCGTTTCTTCTTCTTTCAGTATGGAATTGTATACCAGCTAGGT
ATTGCTGCTAATAGCACCAGTATTGCTGTTTACTTGCTTTCCTGGATCTATGTGGTTGTTGCTGTGGCCA
TTTCTTTAACAATAGCATATGCTCGGGACAAGTATGCTGCAAAAGACCACATCTACTATCGGCTGGTCCA
ATTCCTTGTTATTTTGCTTGTAATAATTGTCATTGTTGCATTGCTGGAGTTCACCCACTTTAAATTTGTT
GATCTCTTCACCAGTCTGCTGGCATTTGTTCCCACTGGGTGGGGCCTGATTTTAATTGCCCAGGTATTCA
GGCCCTTTCTGCGGCGCACTTCAGCATGGGAGGCCATTATTTCTTTGGCTCGGCTATATGATATTATGTT
TGGTGTGATCGTCATGGCCCCTGTGGCACTACTATCATGGTTGCCTGGGTTCCAATCGATGCAGACAAGG
ATTCTATTCAATGAAGCATTTAGTAGGGGCCTCCACATATCTCAGATTGTGACAGGGAAAAAATCCAAGG
TCTAA
>XM_010921260.3_*Elaeis_guineensis*
ATGAGCAGCCTCCGGCAGCGCCCGAACCGGGGCGGCCGCCCGGCCGCCCTTGAGACTGCGGACGACGGAG
CCGGGGAGGAGGCCTACAACATCATCCCCATCCACAATCTCCTGGCCGAGCACCCGTCGCTCCGGTTTCC
GGAGGTCCGCGCCGCTATGGCGGCCCTCCGCACCGTGGGCGAGCTCCGCAAGCCCCCCTTCGTCCGCTGG
CACGACGGACTCGACCTCCTCGATTGGCTCGGCGCCTTCTTTGGCTTCCAGCGCGACAACGTGCGCAACC
AGCGGGAGCACCTCGTCCTCCTCCTCGCCAACGCCCAGATGCGCATCCAGCCCCCGCCTGACAACATCGA
CGCCCTCGACCCCGCCATCGTCCGCCGCATCCGCCGCAAGCTCCTCCACAACTACACCGCCTGGTGCGCC
TACCTCGGCCGGAAGTCTAACGTCTGGATCTCCGACTCCGCCGCCCGCCGCTCACCCGACCCCCGCCGCG
ACCTCCTCTACGCCTCCCTCTACCTCCTCATCTGGGGCGAGGCCGCCAACCTCCGCTTCGTCCCCGAATG
CCTCTGCTACATCTTCCACCACATGGCCATGGACCTCAACCGCATCCTCGAGGGCTACATCGACGAGTCC
ACTGGCCGCCCCGCCCTCCCAGCCATCTCCGGCGAGAACGCCTTCCTCGCCCGCGTCGTCACCCCTCTGT
ACACCACCATCAAGAAAGAGGTCGACGCCAGCCGCAACGGCACCGCACCCCACTCCGCCTGGCGTAACTA
CGACGACATCAACGAGTACTTCTGGAGCCGCCACTGCTTCGACCGCCTCCGCTGGCCCCTCGACAACTCC
CGCAACTTCTTCGCTGCCCCACCCGACAAGAACCGCGTCGGAAAGACCGGCTTTGTCGAGCAGCGCTCCT
TCTGGAACATCTTCCGCAGCTTTGACCGCCTCTGGATCATGCTCATCCTCTTCCTCCAGGCCGCCACCAT
CGTCGCCTGGGAGGGCAAGACGTATCCCTGGCAGAACCTTCGGAGCCGCGACGTCCAGGTCCGCGTCCTC
ACCATCTTCATCACCTGGGCCGGCCTTCGCTTCCTCCAGTCCATCCTCGACGCCGGCACCCAGTACAGCC
GCGTCTCACGGGAGACCCTCTGGCTCGGTGTCCGGATGGTGCTCAAGAGTATCGTTGCCATTGCCTGGAC
TATCGCCTTCGGTGTCCTCTATGCCCGGATCTGGACCCAGAAGAACCATGATCGCCGGTGGTCCTATGAT
GCCAACCAGAGGATAATTACGTTCCTCGAGGTGGCCGGCGTGTTCGTCCTCCCAGAGGTGCTTGCTCTCC
TGCTCTTCATCCTTCCATGGGTCCGCAACTTCCTCGAGAAGACCAACTGGAAGATCTTCTACATCCTCAC
TTGGTGGTTCCAGAGCCGCACATTCGTCGGCCGGGGCCTGCGGGAAGGTTTGGTGGACAATGTGAAATAC
GCCCTCTTCTGGGTCGTCCTCCTTGCCGCGAAATTCAGCTTCAGCTACTTCCTCCAGATCAAGCCCATGG
TTGCCACGAGCAAGGCCATATACAATCTTGATAATATCAAGTACCAGTGGCATGAATTCTTCACCCATAC
AAACCGGTTTGCTGTGGTTCTCTTGTGGATCCCTGTTGTCCTGATCTATCTGATGGACATCCAGATATGG
TACTCTATCTTCTCCTCGCTTGTGGGAGCTCTGGTGGGGCTGTTCTCACACCTGGGTGAGATTCGAAATG
TGCAGCAGCTGAGGCTGAGGTTCCAGTTCTTTGCAAGTGCCATGCAGTTCAATTTGATGCCGGAGGAGCA
GCTGTTCAAGGACCGGGGTTCACTTCGAAGCAAGTTCAGTGATGCGGTCAACCGGCTCAAGCTGAGGTAT
GGGTTGGGGCATCCCTACAAGAAGATTGAATCAAGCCAGGTGCAGGCCAGCAGGTTTGCTCTGATATGGA
ACGAGATCATTGCAACGTTCAGGGAGGAGGATATCATCAGTGACTGTGAGGTGGAGCTTCTTGAGCTGCC
ACCCAACGCATGGAATATCCGGGTAATCAGATGGCCATGCGTGTTACTCTGCAATGAGCTGCTGCTTGCT
CTTGGCCAGGCAAAGGAACTAGAGGCGTCGGACCGGAGTCACTGGAGAAAGATATGCAAGAATGAGTACA
GGCGGTGTGCAGTCATTGAGGCCTATGACAGTGTCAGGTTCCTCTTACTGGAGATCATCAGAGAGGAGAC
TGAAGAGCATGCCATTGTTAACCAGCTGTTTCTCGGGTTTGATGATTCGATTCGGGTGGAGAAGTTTACA
GTGGAGTATAAGATGGCTGTTTTACAAAAGATCCATACCCAACTGATAGTTCTCTTGGACACACTGATCA
AGCCGAACAAAGATTTAAACAAAATGGTCAATACATTGCAGACCCTTTATGACATTGTCATCCGTGACTT
TCCCACTAATAAGAAGAGCACAGATGAGCTGAAGCAGATGGGGTTGGCACCAATGAGGCCGCGACTACTT
TTTGAGAATGCTATCGAACTACCGAGTACAGATAATGCGAACTTCTATAGGCAGGTGAGAAGGCTGCATA
CGGTTCTCTCATCTAGGGATTCCATGAACAATGTACCAAAGAACTTGGAGGCTCGGCGGCGCATTGCTTT
CTTCAGCAACTCCTTGTTCATGAACATGCCCCGAGCTCCTCAAGTTGAAAAGATGATGGCCTTTAGTGTT
CTGACGCCATATTACAATGAGGAGGTTTTGTATAGCAAGGAGCAGCTTCGTACCGATAATGAGGATGGCA
TCTCCATCATATTCTATCTGCAGAAGATTTATGATGATGAGTGGGAAAACTTTTTGGAACGTATGCGGAA
AGGGGGTATGGTTGATGAGAAGGAACTGTGGGGTGTGAGGTTGAGGGATCTCCGTCTTTGGGCCTCATAC
AGGGGCCAGACACTAGCACGTACTGTAAGGGGAATGATGTACTACTACAAGGCTCTCAAGATGCTCACAT

TTCTCGACACTGCTTCTGAGATTGACATAAGGGAAGGGTCGAGGGAACTCGCTTCAGTTGGTTCCTCGAT
GAGGCGGGACAGTGATGAAGATGGTCTGGAGAATGCTGGTAAATCTCCATCATCACGAGTTTTGAGCAGA
GAAAGCAGTGGTGTGAGCTTATTGTTCAAAGGCCATGAGCGTGGGACTGCTCTGATGAAATACACTTATG
TGGTTGCCTGCCAGATATATGGGAACCAAAAGGCTAAGAAGGACCCTCATGCTGAAGATATTCTGTATCT
AATGAAGAACAATGAAGCTCTTCGAGTTGCCTATGTTGATGAAGTCCACACAGGGAGGGATGAAGTAGAG
TACTACTCCGTTCTTGTTAAGTATGATCAGCAATTGGAGCGAGAGGTGGAGATTTACCGGGTCAGATTGC
CTGGGCCTTTGAAGCTTGGAGAGGGCAAGCCAGAGAACCAGAACCATGCTCTCATCTTCACAAGGGGTGA
TGCAGTGCAGACTATAGATATGAATCAAGACAATTACTTTGAGGAGGCCCTCAAGATGCGGAACCTGTTG
GAAGAGTACAATCATAACTATGGTGCCCGAAAACCAACAATCTTGGGAGTTCGAGAACATGTTTTTACTG
GTTCTGTTTCTTCTCTTGCTTGGTTCATGTCTGCGCAGGAGACAAGCTTTGTCACCCTTGGACAGCGAGT
TCTGGCAAACCCTCTGAAGGTGCGAATGCATTATGGCCATCCTGATGTTTTTGACCGTCTCTGGTTTTTG
AGTCGTGGTGGTATCAGTAAGGCATCCAGGGTGATCAACATTAGTGAGGACATATTTGCAGGCTTTAACT
GTACTCTGCGTGGCGGCAATGTTACCCACCATGAGTATATCGAGGTAGGTAAGGGACGGGATGTAGGGCT
GAATCAGATTTCTATGTTTGAAGCCAAGGTTGCTAGTGGCAATGGCGAACAGACTTTAAGCCGAGATGTT
TATAGATTGGGTCACAGGTTGGACTTCTTCCGGATGCTTTCCTTCTTCTACACAACTGTGGGGTTCTACT
TTAACACTATGCTGGTGGTGCTGACTGTCTATGCATTTGTGTGGGGGCGCCTATATCTGGCTCTCAGTGG
ACTTGAGGATTCAATCAAGAATAATGCTGACTCTACCAATAATGCAGCCCTGGGTACTGTTCTTAATCAG
CAGTTCATCATTCAGCTCGGCCTTTTCACTGCATTGCCTATGATTATAGAAAACTCACTTGAGCATGGAT
TCCTTCCTGCGATATGGGATTTCTTCACAATGCAGCTCCAGCTTGCATCTGTGTTCTACACGTTCTCCAT
GGGAACTAAAACTCATTACTATGGGCGAACAATCCTCCATGGAGGTGCAAAGTATCGGGCTACAGGACGT
GGTTTTGTTGTGCAGCATAAGAGCTTTGCTGAGAACTATAGGCTCTATGCTCGTAGCCATTTCATAAAAG
CAATAGAGCTTGGGGTGATATTAACAGTTTATGCAGCTTACAGTGCTCTTTCTAAGAACACCTTTGTCTA
CATTGTCATGACAATTTCGTGCTGGTTCCTGGTGGTATCATGGATAATGGCTCCTTTTGCATTTAATCCA
TCTGGGTTTGATTGGTTGAAAACTGTTTATGACTTTGATGATTTTATGGACTGGATTTGGTACCGTGGCA
GTGTCTTCACAAAATCTGATCAGAGTTGGGAGGTCTGGTGGTATGAGGAGCAGGATCATCTTCGGACAAC
TGGTCTTTGGGGAAAGCTATTGGAAATAATATTAGATCTCCGGTTTTTCTTTTTCCAATATGGGATTGTG
TACCAGCTAAAAATTGCCAATGGGAGCACAAGCATTGCAGTTTATCTGCTTTCTTGGATATATGTGGTTG
TGGCTGTTGGAATTTTTGTGCTAATTGCTTATGCTCGGGACAAGTATGCTGCAAAGGATCACATTTATTA
CCGGGCTGTCCAATCCTTCATCATCATTCTGGTGATCCTAGTGATTGTCATATTGCTCAAGTTCACTAAA
TTTGAGATTGTCGACCTTTTTACGAGCCTTTTGGCATTTATTCCTACTGGGTGGGGCCTTATTTTAATTG
CACAAGTGATCAGACCATTTATTGAGTCAACCTTGGTTTGGGATACTGTGGTTGCTGTGGCACGGCTATA
TGACATAATGTTTGGAGTCGTTGTAATGGCTCCTGTGGCATTATTGTCCTGGTTGCCTGGATTCCAGTCG
ATGCAAACAAGGATTCTCTTCAATGAAGCATTCAGCCGAGGACTCCAGATATCGCGTATTATAACTGGGA
AAAAGCCTAATGTGATTTGA
>XM_015761145.2_*Oryza_sativa_Japonica*_Group
ATGAGCTTGCTGCGGAACCGCCGCGCCGCGGCGGCGGCGGGTTCGGGGGAGCAGACGGTGGTGCAGGCGG
CGTACAACATCATCCCGATCCAGGACGTGGTGATGCACGGGGACCACCCGTCGCTGCAGGTGCCGGAAGT
GCGGGCGGCGGTGGAGGCGCTGTCGCACGCGTCGGACTTCCCGGCGCCGCCGCTGGCCCGCGTCTGGGAC
CCGCACCGCGCCGACATCTTCGACTGGCTCGGCGCCACCTTCGGGTTCCAGGCGGACAACGTCCGGAACC
AGCGGGAGCACCTGGTGCTCCTCCTCGCCAACGCGCAGCTCCGCGCCGCGCCGGGATTCCCCAAGGACCA
CCCCATCGACGTCCTCCACCTCACCGTCGCCCGCGGCATCCGCAGGAAGCTCCTCAAGAACTACACCTCC
TGGTGCGCCTACCTCGGCCAGAAGCGCCACTTCCGCGTGCCCAGCGGCGGCGGCGGCGGCCGCCGCACCG
GCGCCGCCACCGGCAACGACGTCCGCATGGACCTCCTCTACACGGCGCTGTACCTGCTCATCTGGGGGGA
GGCCGCCAACCTGAGGTTCATGCCGGAGTGCCTCTGCTACATCTTCCACTACATGGCCCTCGACCTCCAC
CACGTCGTCGAGCAGTCCATCGACATCGAGACAGGGCGGCCTGCGATGCCCGCCGTGTGCGGCGAGGACG
CCTTCCTCATCCGCGTGGTGACTCCGATCTACAACGTCCTCAAGAACGAGGTGGAGGCCAGCCGGAACGG
GACCAAGCCGCACTCGGCGTGGAGGAATTACGACGATGTCAACGAGTACTTCTGGAGCCGACGAGTTTTC
AAGCGGCTCCGGTGGCCGCTGGATCCATCGAGGAGCTTCTTTGTGGAGCCTGGAAAGACTGGGCGCATTG
GCAAGACTGGCTTTGTTGAGCAGCGGTCATTCTGGAATGTCTACCGAAGTTTCGATAGGGTATGGGTGAT
GCATATTCTGTTCTTTCAAGCTGCGATGATTGTTGCATGGGATGGTAAAACCCCATGGGTTAGCCTCAGA
TTCCGGGACATCCAGGTCCGGGTGCTGTCTGTGTTCATCACTTGGGGTGGACTGCGCTTTGTTCAGGCAA
TGCTTGATGCGGGTACTCAGTACAGTCTCGTGTCGAGGGAGACAAAAACAGTGGCTGTTCGGATGGTGCT
CAAGGTGCTTGTTGCTGCAGGGTGGACAATCACATTCAGTGTGCTCTATAAGCGAATGTGGGATCAGCGG
TGGCGAGATCGCAGGTGGTCCTTTGCTGCCAATACCAGAGTGCTGAATTACCTTGAGGCAGCTGCTGTGT

TTGTCATCCCGCAAGTGCTTGCGATTGTGCTCTTCATCATCCCTTGGATTCGAAATTTTCTGGAGAAAAC
CAATTGGAAAATTCTATATGTGCTGACCTGGTGGTTCCAAACACGTACATTTGTTGGCCGTGGTCTGAGG
GAAGGTCTCATTGATAACATCAAGTACTCCATATTCTGGGTGTGCCTCCTTGTTTCTAAGTTTAGCTTCA
GCTACTTTCTCCAGATTAAGCCGATGGTGGGGCCAACAAAGGTAATCTTCAAACTCCATGACATCAAGCG
TAACTGGTTTGAGTTCATGCCTCACACAGAGCGCCTTGCAGTAATCATCCTGTGGCTCCCAGTTATTATC
ATTTACCTCATGGATATCCAGATATGGTATGCAGTCTTCTCATCACTAACAGGGGCGCTTATCGGCCTTT
TCTCGCATCTGGGGGAGATTCGCAGTGTTGAGCAGCTGCGCTTGAGGTTCCAGTTCTTTGCAAGTGCAAT
GCAGTTCAATTTAATGCCAGAGGAGCACCTGGATACCGTTCATGGTGGCATCCGCAGCAAGTTCTATGAT
GCAATCAATCGGCTGAAGCTGAGATATGGGTTTGGCCGTCCATATAGGAAGATTGAAGCAAACGAGGTGG
AAGCTAAGAGGTTTGCACTGGTTTGGAATGAGATCATTCAAACATTTAGGGAAGAGGACATCATTAGTGA
TAAAGAGCTTGGGCTTCTTGAGCTGCCGGCAGTTGTGTGGAGAATTCGTGTGGTACGGTGGCCTTGTTTG
CTGCTAAAAAATGAGCTTCTTCTTGCTCTCAGTCAGGCAGCAGAGCTGGTGGCTGATGACAGGACACATT
GGAACAAGATATGTAACAACGAGTACAGGCGATGTGCAGTAATTGAAGCTTATGACAGCATACGTCACTT
GCTGCTGGAAATAATCAAGGAGAGAACTAATGAACACATTATTGTCAATCAGCTATTCCTTGCATTTGAT
GGTGCAATGGAATATGGAAAGTTCACTGAAGAATATAGGCTAACCTTATTACCTCAAATTCATAAGTATG
TAATCTCCTTAGTGGAGCAGCTTCTATTGAAGGACAAGGATCAGATCAAGATAGTTCGTACCTTGCAGGA
TCTGTATGATCTTGCAGTGCATGATTTTCCAAAAATCAAGAAGGACTTTGAACAGCTGAGGCGAGAGGGG
CTAGCACTATCAAGACCAACCGAATCTCAGCTGCTTTTTCAGGATGCCATAAAATGCCCAGATGACAATG
ATGTTAGCTTCTATAAGCAGGTGAGAAGACTCCATACGATTCTCACATCCAGGGATTCTATGGACGATGT
TCCAAAGAACCCCGAGGCTCGTCGGCGCATAACTTTCTTCAGCAACTCCCTGTTCATGAACATGCCTCGT
GCTCCCACCGTTCAGAGGATGATGGCATTCAGTGTTTTAACCCCATATTACAATGAAGATGTTCTGTACA
ACAAGGACCAGCTTCGTCGTGAGAATGAAGATGGCATCTCAATCTTGTTTTATCTTCAAAAGATTTATGA
AGATGATTGGAAAAACTTTTTAGAGCGCATGCAAAGGGAAGGAATGGCTAGTGATGATGGCATTTGGGCT
GGAAAATTCCAGGATCTCCGACTTTGGGCCTCGTACAGGGGGCAGACCTTAGCACGGACTGTGAGGGGAA
TGATGTACTACTACAGGGCTCTCAAGATGCTTGCTTTTCTTGATAATGCATCTGAAGTAGAAATTACAGA
GGGAACAAAACAGCTGGCTTCTTTTGGTTCAGTTCAGTATGAGAATGATGTATATCCCATGAACGGGGGT
TTATCACAACGACCTCGAAGGAGGTTAGAGAGAGGAACAAGTACTGTAAGTCAATTGTTTAAAGGCCAGG
AAGATGGTGCTGCTATCATGAAGTACACCTATGTGGTCGCTTGCCAAATATATGGAAACCAGAAAAAGGC
GAAAGATCAACGTGCTGAAGATATCCTAACTCTTATGAAGAAAAATGATGCCCTCCGTGTTGCTTATGTC
GATGAGGTCCATCCTGAGATTGGTGATACGCAATACTATTCTGTCCTTGTTAAATTTGATCCTGTCTTAC
AGAGAGAGGTTGAGATATACCGTATTAGGTTGCCAGGTCAACTGAAACTTGGGGAGGGAAAACCTGAAAA
TCAGAACCATGCCATCATATTCACACGAGGTGATGCTGTGCAAACGATTGATATGAACCAGGATAATTAT
TTTGAGGAGGCCCTCAAGATGCGTAACCTACTAGAACAGTACGACTATTATCATGGAAGCCAGAAACCAA
CGCTTTTGGGCGTCCGGGAGCATGTTTTTACTGGATCGGTGTCTTCACTTGCTTGGTTCATGTCTGCACA
AGAAACAAGCTTTGTTACCCTTGGACAGCGAGTTCTAGCTAATCCGTTGAAGGTTCGGATGCATTATGGG
CATCCTGATGTATTTGATCGCCTTTGGTTTTTAACCCGAGGTGGTATAAGCAAGGCATCCAGAGTAATCA
ATATCAGCGAGGACATATTTGCAGGTTTCAACTGTACCCTACGCGGTGGCAATGTTAGCCACCATGAGTA
TATCCAGGTTGGTAAGGGACGTGATGTTGGGCTCAATCAGATATCGATGTTTGAAGCAAAGGTTTCTAGT
GGCAATGGTGAACAGACCCTAAGTAGGGATATCTACAGACTTGGTCATAGGCTGGATTTCTTTAGGAGTC
TTTCTGTGTTCTATACAACAGTAGGATTCTACTTCAACACAATGATGGTGGTCTTGACAGTTTATACATT
TGTTTGGGGACGCCTCTATCTAGCTCTGAGTGGTCTTGAGGCTGGAATTCAGGGTAGCGCTAATGCTACC
AACAATAAAGCCTTGGGTGCCGTTCTGAATCAGCAATTTGTCATACAGCTTGGCCTCTTCACTGCCTTGC
CAATGATTATAGAGAACTCTCTTGAACAGGGTTTTCTGCCTGCTGTCTGGGATTTTTTCACTATGCAAAT
GATGTTTTCGTCCGTGTTCTACACATTTTCCATGGGGACGAAAAGCCATTACTATGGCCGGACAATTCTT
CATGGCGGTGCTAAGTATCGTGCTACTGGCCGTGGTTTTGTTGTGCAGCATAAGAGTTTTGCTGAAAATT
ACAGGCTGTATGCTAGGAGCCACTTCATAAAGGCAATAGAACTTGGAATAGTATTGACTGTGTATGCAGC
TCACAGTGTGATTGCAAGGGATACACTTGTTTACATAATCATGATGATATCTAGCTGGTTTCTAGTGGTA
TCATGGATCATGGCTCCATTTGCTTTTAATCCATCTGGCTTTGATTGGTTGAAAACAGTGTATGACTTTG
ATGATTTCATGAACTGGATCTGGTACCCTGGTAGTATCTTTTCTAAGGCTGAACACAGCTGGGAAGTTTG
GTGGTTTGAGGAGCAGGATCATCTACGAACAACTGGTCTTTGGGGAAAGATTTTAGAAATATTGTTAGAT
CTCAGATACTTCTTTTTTCAGTATGGGGTTGTATACCAGTTGAAGATCGCAAATGAAAGCAGAAGTATTG
CTGTTTATCTGCTTTCCTGGATTTGTGTGGCTGTGATTTTCGGCATTTTTGTCCTAATGTCATATGCTCG
AGACAAGTATGCTGCAAAACAACACCTTTACTACCGGGTCATCCAATCTGGTGTCATTATTCTTGCAGTG
CTAGTGCTGATAATATTTCTGAAATTTACTAAATTTCAAATCATTGACATCTTCACAAGCCTTTTGGCAT

TCATTCCTACTGGTTGGGGCTTGATTTCCATTGCTCAAGTGATTAGACCATTCATTGAGTCTACTGTGGT
CTGGGCCAGTGTTGTTTCTGTGGCACGTTTGTATGAGATACTGCTTGGGGTGTTTGTTATGGCGCCAGTT
GCATTCTTTTCCTGGTTGCCTGGATTTCAGGAAATGCAGACGAGGGTACTTTTCAATGAAGCTTTCAGCA
GAGGCCTCCAGATATCCCGCATTCTTGCTGGCAAGAAAACAATTGCAGTTTGA
>XM_015761139.2_*Oryza_sativa_Japonica*_Group
ATGAGCTTGCTGCGGAACCGCCGCGCCGCGGCGGCGGCGGGTTCGGGGGAGCAGACGGTGGTGCAGGCGG
CGTACAACATCATCCCGATCCAGGACGTGGTGATGCACGGGGACCACCCGTCGCTGCAGGTGCCGGAAGT
GCGGGCGGCGGTGGAGGCGCTGTCGCACGCGTCGGACTTCCCGGCGCCGCCGCTGGCCCGCGTCTGGGAC
CCGCACCGCGCCGACATCTTCGACTGGCTCGGCGCCACCTTCGGGTTCCAGGCGGACAACGTCCGGAACC
AGCGGGAGCACCTGGTGCTCCTCCTCGCCAACGCGCAGCTCCGCGCCGCGCCGGGATTCCCCAAGGACCA
CCCCATCGACGTCCTCCACCTCACCGTCGCCCGCGGCATCCGCAGGAAGCTCCTCAAGAACTACACCTCC
TGGTGCGCCTACCTCGGCCAGAAGCGCCACTTCCGCGTGCCCAGCGGCGGCGGCGGCGGCCGCCGCACCG
GCGCCGCCACCGGCAACGACGTCCGCATGGACCTCCTCTACACGGCGCTGTACCTGCTCATCTGGGGGGA
GGCCGCCAACCTGAGGTTCATGCCGGAGTGCCTCTGCTACATCTTCCACTACATGGCCCTCGACCTCCAC
CACGTCGTCGAGCAGTCCATCGACATCGAGACAGGGCGGCCTGCGATGCCCGCCGTGTGCGGCGAGGACG
CCTTCCTCATCCGCGTGGTGACTCCGATCTACAACGTCCTCAAGAACGAGGTGGAGGCCAGCCGGAACGG
GACCAAGCCGCACTCGGCGTGGAGGAATTACGACGATGTCAACGAGTACTTCTGGAGCCGACGAGTTTTC
AAGCGGCTCCGGTGGCCGCTGGATCCATCGAGGAGCTTCTTTGTGGAGCCTGGAAAGACTGGGCGCATTG
GCAAGACTGGCTTTGTTGAGCAGCGGTCATTCTGGAATGTCTACCGAAGTTTCGATAGGGTATGGGTGAT
GCATATTCTGTTCTTTCAAGCTGCGATGATTGTTGCATGGGATGGTAAAACCCCATGGGTTAGCCTCAGA
TTCCGGGACATCCAGGTCCGGGTGCTGTCTGTGTTCATCACTTGGGGTGGACTGCGCTTTGTTCAGGCAA
TGCTTGATGCGGGTACTCAGTACAGTCTCGTGTCGAGGGAGACAAAAACAGTGGCTGTTCGGATGGTGCT
CAAGGTGCTTGTTGCTGCAGGGTGGACAATCACATTCAGTGTGCTCTATAAGCGAATGTGGGATCAGCGG
TGGCGAGATCGCAGGTGGTCCTTTGCTGCCAATACCAGAGTGCTGAATTACCTTGAGGCAGCTGCTGTGT
TTGTCATCCCGCAAGTGCTTGCGATTGTGCTCTTCATCATCCCTTGGATTCGAAATTTTCTGGAGAAAAC
CAATTGGAAAATTCTATATGTGCTGACCTGGTGGTTCCAAACACGTACATTTGTTGGCCGTGGTCTGAGG
GAAGGTCTCATTGATAACATCAAGTACTCCATATTCTGGGTGTGCCTCCTTGTTTCTAAGTTTAGCTTCA
GCTACTTTCTCCAGATTAAGCCGATGGTGGGGCCAACAAAGGTAATCTTCAAACTCCATGACATCAAGCG
TAACTGGTTTGAGTTCATGCCTCACACAGAGCGCCTTGCAGTAATCATCCTGTGGCTCCCAGTTATTATC
ATTTACCTCATGGATATCCAGATATGGTATGCAGTCTTCTCATCACTAACAGGGGCGCTTATCGGCCTTT
TCTCGCATCTGGGGGAGATTCGCAGTGTTGAGCAGCTGCGCTTGAGGTTCCAGTTCTTTGCAAGTGCAAT
GCAGTTCAATTTAATGCCAGAGGAGCACCTGGATACCGTTCATGGTGGCATCCGCAGCAAGTTCTATGAT
GCAATCAATCGGCTGAAGCTGAGATATGGGTTTGGCCGTCCATATAGGAAGATTGAAGCAAACGAGGTGG
AAGCTAAGAGGTTTGCACTGGTTTGGAATGAGATCATTCAAACATTTAGGGAAGAGGACATCATTAGTGA
TAAAGAGCTTGGGCTTCTTGAGCTGCCGGCAGTTGTGTGGAGAATTCGTGTGGTACGGTGGCCTTGTTTG
CTGCTAAAAAATGAGCTTCTTCTTGCTCTCAGTCAGGCAGCAGAGCTGGTGGCTGATGACAGGACACATT
GGAACAAGATATGTAACAACGAGTACAGGCGATGTGCAGTAATTGAAGCTTATGACAGCATACGTCACTT
GCTGCTGGAAATAATCAAGGAGAGAACTAATGAACACATTATTGTCAATCAGCTATTCCTTGCATTTGAT
GGTGCAATGGAATATGGAAAGTTCACTGAAGAATATAGGCTAACCTTATTACCTCAAATTCATAAGTATG
TAATCTCCTTAGTGGAGCAGCTTCTATTGAAGGACAAGGATCAGATCAAGATAGTTCGTACCTTGCAGGA
TCTGTATGATCTTGCAGTGCATGATTTTCCAAAAATCAAGAAGGACTTTGAACAGCTGAGGCGAGAGGGG
CTAGCACTATCAAGACCAACCGAATCTCAGCTGCTTTTTCAGGATGCCATAAAATGCCCAGATGACAATG
ATGTTAGCTTCTATAAGCAGGTGAGAAGACTCCATACGATTCTCACATCCAGGGATTCTATGGACGATGT
TCCAAAGAACCCCGAGGCTCGTCGGCGCATAACTTTCTTCAGCAACTCCCTGTTCATGAACATGCCTCGT
GCTCCCACCGTTCAGAGGATGATGGCATTCAGTGTTTTAACCCCATATTACAATGAAGATGTTCTGTACA
ACAAGGACCAGCTTCGTCGTGAGAATGAAGATGGCATCTCAATCTTGTTTTATCTTCAAAAGATTTATGA
AGATGATTGGAAAAACTTTTTAGAGCGCATGCAAAGGGAAGGAATGGCTAGTGATGATGGCATTTGGGCT
GGAAAATTCCAGGATCTCCGACTTTGGGCCTCGTACAGGGGGCAGACCTTAGCACGGACTGTGAGGGGAA
TGATGTACTACTACAGGGCTCTCAAGATGCTTGCTTTTCTTGATAATGCATCTGAAGTAGAAATTACAGA
GGGAACAAAACAGCTGGCTTCTTTTGGTTCAGTTCAGTATGAGAATGATGTATATCCCATGAACGGGGGT
TTATCACAACGACCTCGAAGGAGGTTAGAGAGAGGAACAAGTACTGTAAGTCAATTGTTTAAAGGCCAGG
AAGATGGTGCTGCTATCATGAAGTACACCTATGTGGTCGCTTGCCAAATATATGGAAACCAGAAAAAGGC
GAAAGATCAACGTGCTGAAGATATCCTAACTCTTATGAAGAAAAATGATGCCCTCCGTGTTGCTTATGTC
GATGAGGTCCATCCTGAGATTGGTGATACGCAATACTATTCTGTCCTTGTTAAATTTGATCCTGTCTTAC

AGAGAGAGGTTGAGATATACCGTATTAGGTTGCCAGGTCAACTGAAACTTGGGGAGGGAAAACCTGAAAA
TCAGAACCATGCCATCATATTCACACGAGGTGATGCTGTGCAAACGATTGATATGAACCAGGATAATTAT
TTTGAGGAGGCCCTCAAGATGCGTAACCTACTAGAACAGTACGACTATTATCATGGAAGCCAGAAACCAA
CGCTTTTGGGCGTCCGGGAGCATGTTTTTACTGGATCGGTGTCTTCACTTGCTTGGTTCATGTCTGCACA
AGAAACAAGCTTTGTTACCCTTGGACAGCGAGTTCTAGCTAATCCGTTGAAGGTTCGGATGCATTATGGG
CATCCTGATGTATTTGATCGCCTTTGGTTTTTAACCCGAGGTGGTATAAGCAAGGCATCCAGAGTAATCA
ATATCAGCGAGGACATATTTGCAGGTTTCAACTGTACCCTACGCGGTGGCAATGTTAGCCACCATGAGTA
TATCCAGGTTGGTAAGGGACGTGATGTTGGGCTCAATCAGATATCGATGTTTGAAGCAAAGGTTTCTAGT
GGCAATGGTGAACAGACCCTAAGTAGGGATATCTACAGACTTGGTCATAGGCTGGATTTCTTTAGGAGTC
TTTCTGTGTTCTATACAACAGTAGGATTCTACTTCAACACAATGATGGTGGTCTTGACAGTTTATACATT
TGTTTGGGGACGCCTCTATCTAGCTCTGAGTGGTCTTGAGGCTGGAATTCAGGGTAGCGCTAATGCTACC
AACAATAAAGCCTTGGGTGCCGTTCTGAATCAGCAATTTGTCATACAGCTTGGCCTCTTCACTGCCTTGC
CAATGATTATAGAGAACTCTCTTGAACAGGGTTTTCTGCCTGCTGTCTGGGATTTTTTCACTATGCAAAT
GATGTTTTCGTCCGTGTTCTACACATTTTCCATGGGGACGAAAAGCCATTACTATGGCCGGACAATTCTT
CATGGCGGTGCTAAGTATCGTGCTACTGGCCGTGGTTTTGTTGTGCAGCATAAGAGTTTTGCTGAAAATT
ACAGGCTGTATGCTAGGAGCCACTTCATAAAGGCAATAGAACTTGGAATAGTATTGACTGTGTATGCAGC
TCACAGTGTGATTGCAAGGGATACACTTGTTTACATAATCATGATGATATCTAGCTGGTTTCTAGTGGTA
TCATGGATCATGGCTCCATTTGCTTTTAATCCATCTGGCTTTGATTGGTTGAAAACAGTGTATGACTTTG
ATGATTTCATGAACTGGATCTGGTACCCTGGTAGTATCTTTTCTAAGGCTGAACACAGCTGGGAAGTTTG
GTGGTTTGAGGAGCAGGATCATCTACGAACAACTGGTCTTTGGGGAAAGATTTTAGAAATATTGTTAGAT
CTCAGATACTTCTTTTTTCAGTATGGGGTTGTATACCAGTTGAAGATCGCAAATGAAAGCAGAAGTATTG
CTGTTTATCTGCTTTCCTGGATTTGTGTGGCTGTGATTTTCGGCATTTTTGTCCTAATGTCATATGCTCG
AGACAAGTATGCTGCAAAACAACACCTTTACTACCGGGTCATCCAATCTGGTGTCATTATTCTTGCAGTG
CTAGTGCTGATAATATTTCTGAAATTTACTAAATTTCAAATCATTGACATCTTCACAAGCCTTTTGGCAT
TCATTCCTACTGGTTGGGGCTTGATTTCCATTGCTCAAGTGATTAGACCATTCATTGAGTCTACTGTGGT
CTGGGCCAGTGTTGTTTCTGTGGCACGTTTGTATGAGATACTGCTTGGGGTGTTTGTTATGGCGCCAGTT
GCATTCTTTTCCTGGTTGCCTGGATTTCAGGAAATGCAGACGAGGGTACTTTTCAATGAAGCTTTCAGCA
GAGGCCTCCAGATATCCCGCATTCTTGCTGGCAAGAAAACAATTGCAGTTTGA
>XM_015761151.2_*Oryza_sativa_Japonica*_Group
ATGAGCTTGCTGCGGAACCGCCGCGCCGCGGCGGCGGCGGGTTCGGGGGAGCAGACGGTGGTGCAGGCGG
CGTACAACATCATCCCGATCCAGGACGTGGTGATGCACGGGGACCACCCGTCGCTGCAGGTGCCGGAAGT
GCGGGCGGCGGTGGAGGCGCTGTCGCACGCGTCGGACTTCCCGGCGCCGCCGCTGGCCCGCGTCTGGGAC
CCGCACCGCGCCGACATCTTCGACTGGCTCGGCGCCACCTTCGGGTTCCAGGCGGACAACGTCCGGAACC
AGCGGGAGCACCTGGTGCTCCTCCTCGCCAACGCGCAGCTCCGCGCCGCGCCGGGATTCCCCAAGGACCA
CCCCATCGACGTCCTCCACCTCACCGTCGCCCGCGGCATCCGCAGGAAGCTCCTCAAGAACTACACCTCC
TGGTGCGCCTACCTCGGCCAGAAGCGCCACTTCCGCGTGCCCAGCGGCGGCGGCGGCGGCCGCCGCACCG
GCGCCGCCACCGGCAACGACGTCCGCATGGACCTCCTCTACACGGCGCTGTACCTGCTCATCTGGGGGGA
GGCCGCCAACCTGAGGTTCATGCCGGAGTGCCTCTGCTACATCTTCCACTACATGGCCCTCGACCTCCAC
CACGTCGTCGAGCAGTCCATCGACATCGAGACAGGGCGGCCTGCGATGCCCGCCGTGTGCGGCGAGGACG
CCTTCCTCATCCGCGTGGTGACTCCGATCTACAACGTCCTCAAGAACGAGGTGGAGGCCAGCCGGAACGG
GACCAAGCCGCACTCGGCGTGGAGGAATTACGACGATGTCAACGAGTACTTCTGGAGCCGACGAGTTTTC
AAGCGGCTCCGGTGGCCGCTGGATCCATCGAGGAGCTTCTTTGTGGAGCCTGGAAAGACTGGGCGCATTG
GCAAGACTGGCTTTGTTGAGCAGCGGTCATTCTGGAATGTCTACCGAAGTTTCGATAGGGTATGGGTGAT
GCATATTCTGTTCTTTCAAGCTGCGATGATTGTTGCATGGGATGGTAAAACCCCATGGGTTAGCCTCAGA
TTCCGGGACATCCAGGTCCGGGTGCTGTCTGTGTTCATCACTTGGGGTGGACTGCGCTTTGTTCAGGCAA
TGCTTGATGCGGGTACTCAGTACAGTCTCGTGTCGAGGGAGACAAAAACAGTGGCTGTTCGGATGGTGCT
CAAGGTGCTTGTTGCTGCAGGGTGGACAATCACATTCAGTGTGCTCTATAAGCGAATGTGGGATCAGCGG
TGGCGAGATCGCAGGTGGTCCTTTGCTGCCAATACCAGAGTGCTGAATTACCTTGAGGCAGCTGCTGTGT
TTGTCATCCCGCAAGTGCTTGCGATTGTGCTCTTCATCATCCCTTGGATTCGAAATTTTCTGGAGAAAAC
CAATTGGAAAATTCTATATGTGCTGACCTGGTGGTTCCAAACACGTACATTTGTTGGCCGTGGTCTGAGG
GAAGGTCTCATTGATAACATCAAGTACTCCATATTCTGGGTGTGCCTCCTTGTTTCTAAGTTTAGCTTCA
GCTACTTTCTCCAGATTAAGCCGATGGTGGGGCCAACAAAGGTAATCTTCAAACTCCATGACATCAAGCG
TAACTGGTTTGAGTTCATGCCTCACACAGAGCGCCTTGCAGTAATCATCCTGTGGCTCCCAGTTATTATC
ATTTACCTCATGGATATCCAGATATGGTATGCAGTCTTCTCATCACTAACAGGGGCGCTTATCGGCCTTT

TCTCGCATCTGGGGGAGATTCGCAGTGTTGAGCAGCTGCGCTTGAGGTTCCAGTTCTTTGCAAGTGCAAT
GCAGTTCAATTTAATGCCAGAGGAGCACCTGGATACCGTTCATGGTGGCATCCGCAGCAAGTTCTATGAT
GCAATCAATCGGCTGAAGCTGAGATATGGGTTTGGCCGTCCATATAGGAAGATTGAAGCAAACGAGGTGG
AAGCTAAGAGGTTTGCACTGGTTTGGAATGAGATCATTCAAACATTTAGGGAAGAGGACATCATTAGTGA
TAAAGAGCTTGGGCTTCTTGAGCTGCCGGCAGTTGTGTGGAGAATTCGTGTGGTACGGTGGCCTTGTTTG
CTGCTAAAAAATGAGCTTCTTCTTGCTCTCAGTCAGGCAGCAGAGCTGGTGGCTGATGACAGGACACATT
GGAACAAGATATGTAACAACGAGTACAGGCGATGTGCAGTAATTGAAGCTTATGACAGCATACGTCACTT
GCTGCTGGAAATAATCAAGGAGAGAACTAATGAACACATTATTGTCAATCAGCTATTCCTTGCATTTGAT
GGTGCAATGGAATATGGAAAGTTCACTGAAGAATATAGGCTAACCTTATTACCTCAAATTCATAAGTATG
TAATCTCCTTAGTGGAGCAGCTTCTATTGAAGGACAAGGATCAGATCAAGATAGTTCGTACCTTGCAGGA
TCTGTATGATCTTGCAGTGCATGATTTTCCAAAAATCAAGAAGGACTTTGAACAGCTGAGGCGAGAGGGG
CTAGCACTATCAAGACCAACCGAATCTCAGCTGCTTTTTCAGGATGCCATAAAATGCCCAGATGACAATG
ATGTTAGCTTCTATAAGCAGGTGAGAAGACTCCATACGATTCTCACATCCAGGGATTCTATGGACGATGT
TCCAAAGAACCCCGAGGCTCGTCGGCGCATAACTTTCTTCAGCAACTCCCTGTTCATGAACATGCCTCGT
GCTCCCACCGTTCAGAGGATGATGGCATTCAGTGTTTTAACCCCATATTACAATGAAGATGTTCTGTACA
ACAAGGACCAGCTTCGTCGTGAGAATGAAGATGGCATCTCAATCTTGTTTTATCTTCAAAAGATTTATGA
AGATGATTGGAAAAACTTTTTAGAGCGCATGCAAAGGGAAGGAATGGCTAGTGATGATGGCATTTGGGCT
GGAAAATTCCAGGATCTCCGACTTTGGGCCTCGTACAGGGGGCAGACCTTAGCACGGACTGTGAGGGGAA
TGATGTACTACTACAGGGCTCTCAAGATGCTTGCTTTTCTTGATAATGCATCTGAAGTAGAAATTACAGA
GGGAACAAAACAGCTGGCTTCTTTTGGTTCAGTTCAGTATGAGAATGATGTATATCCCATGAACGGGGGT
TTATCACAACGACCTCGAAGGAGGTTAGAGAGAGGAACAAGTACTGTAAGTCAATTGTTTAAAGGCCAGG
AAGATGGTGCTGCTATCATGAAGTACACCTATGTGGTCGCTTGCCAAATATATGGAAACCAGAAAAAGGC
GAAAGATCAACGTGCTGAAGATATCCTAACTCTTATGAAGAAAAATGATGCCCTCCGTGTTGCTTATGTC
GATGAGGTCCATCCTGAGATTGGTGATACGCAATACTATTCTGTCCTTGTTAAATTTGATCCTGTCTTAC
AGAGAGAGGTTGAGATATACCGTATTAGGTTGCCAGGTCAACTGAAACTTGGGGAGGGAAAACCTGAAAA
TCAGAACCATGCCATCATATTCACACGAGGTGATGCTGTGCAAACGATTGATATGAACCAGGATAATTAT
TTTGAGGAGGCCCTCAAGATGCGTAACCTACTAGAACAGTACGACTATTATCATGGAAGCCAGAAACCAA
CGCTTTTGGGCGTCCGGGAGCATGTTTTTACTGGATCGGTGTCTTCACTTGCTTGGTTCATGTCTGCACA
AGAAACAAGCTTTGTTACCCTTGGACAGCGAGTTCTAGCTAATCCGTTGAAGGTTCGGATGCATTATGGG
CATCCTGATGTATTTGATCGCCTTTGGTTTTTAACCCGAGGTGGTATAAGCAAGGCATCCAGAGTAATCA
ATATCAGCGAGGACATATTTGCAGGTTTCAACTGTACCCTACGCGGTGGCAATGTTAGCCACCATGAGTA
TATCCAGGTTGGTAAGGGACGTGATGTTGGGCTCAATCAGATATCGATGTTTGAAGCAAAGGTTTCTAGT
GGCAATGGTGAACAGACCCTAAGTAGGGATATCTACAGACTTGGTCATAGGCTGGATTTCTTTAGGAGTC
TTTCTGTGTTCTATACAACAGTAGGATTCTACTTCAACACAATGATGGTGGTCTTGACAGTTTATACATT
TGTTTGGGGACGCCTCTATCTAGCTCTGAGTGGTCTTGAGGCTGGAATTCAGGGTAGCGCTAATGCTACC
AACAATAAAGCCTTGGGTGCCGTTCTGAATCAGCAATTTGTCATACAGCTTGGCCTCTTCACTGCCTTGC
CAATGATTATAGAGAACTCTCTTGAACAGGGTTTTCTGCCTGCTGTCTGGGATTTTTTCACTATGCAAAT
GATGTTTTCGTCCGTGTTCTACACATTTTCCATGGGGACGAAAAGCCATTACTATGGCCGGACAATTCTT
CATGGCGGTGCTAAGTATCGTGCTACTGGCCGTGGTTTTGTTGTGCAGCATAAGAGTTTTGCTGAAAATT
ACAGGCTGTATGCTAGGAGCCACTTCATAAAGGCAATAGAACTTGGAATAGTATTGACTGTGTATGCAGC
TCACAGTGTGATTGCAAGGGATACACTTGTTTACATAATCATGATGATATCTAGCTGGTTTCTAGTGGTA
TCATGGATCATGGCTCCATTTGCTTTTAATCCATCTGGCTTTGATTGGTTGAAAACAGTGTATGACTTTG
ATGATTTCATGAACTGGATCTGGTACCCTGGTAGTATCTTTTCTAAGGCTGAACACAGCTGGGAAGTTTG
GTGGTTTGAGGAGCAGGATCATCTACGAACAACTGGTCTTTGGGGAAAGATTTTAGAAATATTGTTAGAT
CTCAGATACTTCTTTTTTCAGTATGGGGTTGTATACCAGTTGAAGATCGCAAATGAAAGCAGAAGTATTG
CTGTTTATCTGCTTTCCTGGATTTGTGTGGCTGTGATTTTCGGCATTTTTGTCCTAATGTCATATGCTCG
AGACAAGTATGCTGCAAAACAACACCTTTACTACCGGGTCATCCAATCTGGTGTCATTATTCTTGCAGTG
CTAGTGCTGATAATATTTCTGAAATTTACTAAATTTCAAATCATTGACATCTTCACAAGCCTTTTGGCAT
TCATTCCTACTGGTTGGGGCTTGATTTCCATTGCTCAAGTGATTAGACCATTCATTGAGTCTACTGTGGT
CTGGGCCAGTGTTGTTTCTGTGGCACGTTTGTATGAGATACTGCTTGGGGTGTTTGTTATGGCGCCAGTT
GCATTCTTTTCCTGGTTGCCTGGATTTCAGGAAATGCAGACGAGGGTACTTTTCAATGAAGCTTTCAGCA
GAGGCCTCCAGATATCCCGCATTCTTGCTGGCAAGAAAACAATTGCAGTTTGA
>PMR4_scaffold00300_*Castanea_mollissima*

ATGAGCTACCACAGGCAACGCCCACCTCCGAGCCCGAACCCGAGCCAAACCCGGCCCAGTCCGGCCACACCGC
CATTAGAATCCGACCCATACAACATAATCCCAATCCACAACCTCCTCGCGGAGCACCCGTCGCTCAGGTTCCC
GGAGGTCCGAGCCATAACCACCGCGCTCCGAGCCGTGGGAGACCTCCGGAAGCCGCCGCACAACCAATGGGCA
CCGCACATGGACCTCCTGGACTGGCTGGCCCTCTTCTTCGGCTTCCAAAGCGACAACGTTCGCAACCAGCGCG
AGCACCTCGTTCTCCACCTCGCCAACGCGCAGATGCGCCTCTCTCCTCCGCCGGACAACATCGACGCCCTGGA
CGCCGCCGTTTTGCGCAAGTTTCGGAAGAAGCTCCTCAGGAACTACACCAGCTGGTGCTCCTATCTCGGCAAG
AAGTCCAATATCTGGATCTCCGATCGCCGCGAGGCCGTCGCCGACCACCGGCGGGAGCTGCTGTACGTGTCGC
TCTTCCTTCTGATTTGGGGTGAGTCTGCTAATCTCCGCTTTGTTCCTGAGTGCATTTGCTTTATATTTCATAA
CATGGCCATGGAGTTGAACAAGATCTTGGAGGATTACATAGACGAGAACACCGGTCAACCTGTGCAGCCCTCA
ATCTCCGGCGAAAACGCGTTTTTGAACTGCGTTGTGAAGCCGATTTACGAGACGATTAGGGCTGAGGTGGAGA
GTAGTAAGAACGGCTCTGCGCCGCACAGCGTGTGGCGCAATTACGACGACATTAACGAGTACTTTTGGAGCAA
GCGGTGCTTTCAGAAGCTCAGATGGCCGATTGATTTAGGAAGTAATTTCTTTGTGACGAGCGAGGGTGGTGCG
AGAAGAAAACACGTAGGGAAGACTGGTTTTGTGGAGCAGAGATCGTTTTGGAACTTGTTGAGGAGCTTTGACA
GGCTTTGGGTGATGCTTGTGTTGTTTTTACAAGCGGCGATTATAGTGGCGTGGGAAGAGAGGAAATATCCATG
GGATGCTCTGCAGACTAGGGATGTCCAAGCGAAAGTTTTGACAGTGTTTTTCACTTGGAGTGGGATGAGGTTT
TTGCAGTCTGTGTTGGATGCAGGGATGCAATACAGTTTGGTTTCGAGGGAGACGTTGGGGCTTGGCGTGAGGA
TGGTGATGAAGAGTGTGGTTGCAGCCGTGTGGATCTTGGTTTTTGGTGTGTTTTATGGGCGGATATGGGCGCA
GAAGAATCGCGATAGGAGGTGGTCTCCAGAGGCTGATAGGAGGTTGGTTACTTTTCTTGAGGTGGCATTGGTT
TTCATTTTGCCGGAGCTTCTGGCCGTGGTGCTGTTTGTGATTCCGTGGATCAGGAATTTCCTCGAGGAGACCA
ATTTGAAGATCTTTTATATGTTGACTTGGTGGTTTCAAGGGAGGACCTTTGTGGGTCGTGGGTTGAGGGAAGG
TCTTTTGGATAATATCAAGTACACTTTGTTTTGGATTCTGGTGCTTGCTACCAAATTTTTGTTCAGTTACTTC
TTGCAGGTTAAACCGATGATTGCCCCAACTAAAGCATTGTTGGATTTGAAGAATGTGGAGTATCAATGGTATC
AGCTTTTTAACAATAGCAACAGATTGGCAGTGGGCTTACTGTGGCTTCCTGTTGTTCTGATTTACCTCATGGA
TATTCAGATTTGGTATTCAATCTACTCGTCTTTTGTTGGGGCGGGTGTGGGATTGTTTCAGCACTTGGGTGAG
ATTCGAAATATTGAACAGTTGAGGTTGAGGTTCCAATTCTTTGCAAGTGCTATTCAGTTTAATCTCATGCCGG
AGGAGCAGCTGCTAAATCCAAGGGGGATGAGGAACAGGTTTAAGGATGCCATTCATCGATTGAAGCTGAGATA
TGGGCTTGGTCGGCCCTATAGGAAGCTTGAATCGAGTCAGGTTGAGGCGAAGAGGTTTGCTTTGATATGGAAT
GAGATAGTTTTGATTTTCAGGGAAGAAGACATCATCTCTGACCGTGAGCAGGAGCTGTTGGAGCTGCCCCAGA
ATTCTTGGAATGTCAGGGTCATTCGATGGCCTTGTTTCCTCCTCTGTAATGAGCTTCTGCTTGCGCTCAGTCA
GGCCAAAGAGTTGTTAGATGCTCCGGACAAGTGGCTCTGGTATAAGATATGCAAGAATGAGTACAGGCGTTGC
GCTGTGATTGAAGCTTATGATTGTATCAAACACTTGCTGCTTGAGATTATCAAAAGCAACACAGAAGAGCATT
CCATTATGAACATGTTTTTTAAAGAGATTGATCACTCCATTGAGATTGAGAAGTTCACTAAAACATTTAACAT
GGCTGTTCTGCCCCAGCTTCATACCCAGTTAATCAAACTTGTTCAGCTATTGAACAAGCCAAAGAAAGATCCT
ACCCAGGTAGTGAACGCTCTGCAAGCCATTTATGAGATTGCTAAACGGGACCTTTTCAAAGACAAGAGGAGCA
TTGACCAGTTGAGGGAGGATGGTCTGGCTCCTCGTAGTCCAGCTTCCACACAGGGGCTGCTTTTTGAGAATGC
TGTTGAATTGCCTAATTCAGATAATGAGACATTCTATAGGCAGGTTCGGCGGTTGCACACAATTCTTACCTCT
CGGGACTCAATGCACAATATCCCAGTAAATCTTGAGGCAAGGCGCCGAATTGCCTTCTTCAGTAATTCACTTT
TCATGAACATGCCTCATGCCCCTCAGGTTGAGAAAATGATGGCCTTCAGTGTTCTGACCCCTTATTACAGTGA
AGAAGTAATCTATAGCAAAGAACAACTCAAATCTGAGAATGAAGACGGTATTTCGATCCTGTACTATTTGCAG
ACGATTTATGCCGATGAGTGGAAAAATTTCATGGAGAGAATGCGCCGAGAAGGGATGGTGAAGGATACCGAGA
TATGGAATACTAAGCTGAGAGATCTCAGGCTTTGGGCATCATACAGGGGTCAGACACTCTCCCGAACTGTTAG
GGGAATGATGTATTACTATCGGGCTCTTAAGATGCTGGCATTTCTGGATTCTGCATCAGAGATGGACATTCGG
GAAGGATCACAAGAACTTGGTTCGATGAGGCGAGACAGCGGTTTGGATGGTTACTCCTCGGAAAGGTCACCAT
CTTCTAGGAGTTTAAGCAGAGGAAGCAGTTCAATGGGCTTGTTACTCAAAGGCCACGAGTATGGGACTGCATT
GATGAAATTTACATATGTGGTTGCCTGCCAGATATATGGAACTCAGAAGGCAAAGAAAGATCCACACGCTGAG
GAAATATTGTATCTAATGAAAAACAATGAAGCCCTTCGAGTTGCCTACGTTGATGAAGTTTCCACTGGGAGGG
ATGGGAAGGAGTTTTATTCTGTTCTTGTGAAGTATGATCACCAATTGGAGAGGGAAGTGGAGATCTATCGGGT
AAAGTTGCCTGGTCCCTTGAAGCTTGGTGAGGGAAAACCGGAGAATCAGAATCATGCCATCATCTTCACTCGC
GGTGATGCTGTCCAGACAATTGATATGAACCAAGACAACTATTTTGAAGAGGCACTCAAAATGCGCAATCTGT
TGGAAGAATTCAGGCGTTATTATGGTATCCGGAAGCCTACTATCTTGGGAGTAAGGGAACACATATTTACAGG
TTCTGTTTCATCACTTGCTTGGTTTATGTCGGCTCAGGAAACGAGTTTTGTCACCTTGGGGCAGCGTGTTTTG
GCGAACCCTTTAAAAGTTCGAATGCATTATGGCCATCCAGATGTGTTTGACAGGTTTTGGTTCATGACTCGTG
GTGGGATCAGTAAAGCTTCCAGAGTGATTAATATCAGTGAGGACATTTTTGCTGGCTTTAACTGCACATTGCG
TGGAGGGAATGTCACCCACCATGAATACATCCAGGTCGGCAAGGGAAGGGATGTTGGGTTGAATCAAGTATCC

ATGTTTGAGGCCAAGGTGGCTAGTGGAAATGGTGAGCAAGTTCTTAGCAGAGATATATACAGGTTGGGTCATA
GGTTGGACTTCTTCCGAATGCTGTCATTCTTTTACACTACCGTGGGATTCTTTTTCAACACAATGGTGGTGAT
TCTGACTGTATATGCATTTCTGTGGGGCCGACTCTATCTGGCTCTTAGTGGTGTTGAGGATTCTTCTAAGGCA
AGTAGTACTAATAACAATGCGGCACTTGGTGCAATCTTGAATCAGCAGTTCATCATCCAGCTTGGTCTGTTCA
CTGCCCTTCCGATGATAGTGGAAAACTCTCTTGAGCATGGGTTCCTTCAAGCTATCTGGGATTTTCTGACAAT
GCAGCTCCAGCTTTCATCTGTATTCTACACATTCTCAATGGGAACTCGTGCCCACTACTTTGGCCGTACTATT
CTTCATGGTGGTGCAAAATATCGGGCTACCGGGCGTGGTTTTGTCGTGGAGCACAAGGGTTTTGCTGAGAATT
ATAGACTCTATGCTCGTAGCCATTTTGTGAAGGCAATTGAACTTGGGTTAATACTTGTAGTTTATGCGTCACA
CAGCCCTATAGCTGGCAACACATTTGTTTACATAGCCTTGACCATCACTAGTTGGTTCCTGGTTGTGTCATGG
ATTATGGCCCCCTTTGTGTTCAATCCTTCTGGATTTGATTGGTTGAAGACGGTGTACGACTTTGATGACTTTA
TGAACTGGATTTGGTTCCGTGGCAGTGTTTTTGCAAAAGCTGAACAGAGTTGGGAAAGATGGTGGTATGAGGA
GCAGGATCATCTCAGGAACACTGGCATTTGGGGTAAAATAATGGAAATAATCTTAGACCTCCGATTCTTCTTC
TTCCAGTATGGGATAGTATATCAACTAAATATTGCAGCTGGAAGTACCAGCATTGCTGTTTACTTGTTGTCTT
GGATCTATGTATTTGTGGCTTTTGCAATTTATGCGGTAATAGCATTTGCTCGGGTTAAATATGCGGCGAAAGA
GCACATATACTATCGTCTAGTCCAATTCCTCGTGATTATACTTGCAATACTTGTGATAATTGCTCTGCTGCAA
TTTACAAAATTCACATTTATTGATATTTTCACTAGCCTGTTAGCATTCATCCCCACTGGTTGGGGCCTGATAT
TGATTGCCCAAGTACTCCGGCCCTTTCTGCGGTCTACTCTTGTTTGGGAAAGTGTTGTTTCTGTGGCCCGACT
ATATGATATAATGTTCGGAGTAATTGTCATGGTTCCTGTGGCATTACTGTCATGGTTGCCTGGGTTTCAGTCA
ATGCAGACGAGGATCCTTTTCAATGAAGCATTTAGCAGGGGCCTCCGCATTAACCAGCTTGTTGCAGGAAAAA
AATCTAATGACTTATAA
