# Supplementary material for: Identification of Susceptibility Genes in Castanea sativa and Their Transcription Dynamics following Pathogen Infection
Source: Plants (Basel). 2021 May 2;10(5):913. doi: 10.3390/plants10050913 (PMC8147476; doi:10.3390/plants10050913)
Supplement: Supplementary file 1 [file plants-10-00913-s001.zip › S5 File.pdf]

Gene	Primers sequence
MLO1_F	GAGGGGTTTCTGGTTCTTCC
MLO1_R	GTGTGCACTGTGATTGTAGCC
DND1_F	AACACCGGTAAGCCCACTC
DND1_R	ACCAGCGACAGTGACAACAG
PMR4_F	TCTTCATCACCATCCTCACG
PMR4_R	GGGATGAGGTTTTTGCAGTC
DMR6_F	GATATCGGTGGCTTCTTTGC
DMR6_R	CCCTTGGATTCTCTTCGTTG
CHI3_F	GCCATCAAGCCACAATGTCATC
CHI3_R	ATCCCACATTCGAGACCACC
GLUB_F	ACAACCTCCAATGAACCGCC
GLUB_R	TCCACGCGACATTTCTCTTCC
YPT_F	TAGCTGCTAGTGATCGTGCG
YPT_R	GTCCTTCGCGGTCCTATTCG
MF1_F	TCATCCTGGAACACTTCGGC
MF1_R	TGGTGGTGGTTGAGTCATCG
ACTIN7_F	CCAAGGCCAACAGGGAAAA
ACTIN7_R	CGGCCTGGATAGCAACATA
