# Supplementary figures and images for: Identification of Susceptibility Genes in Castanea sativa and Their Transcription Dynamics following Pathogen Infection
Source: Plants (Basel). 2021 May 2;10(5):913. doi: 10.3390/plants10050913 (PMC8147476; doi:10.3390/plants10050913)

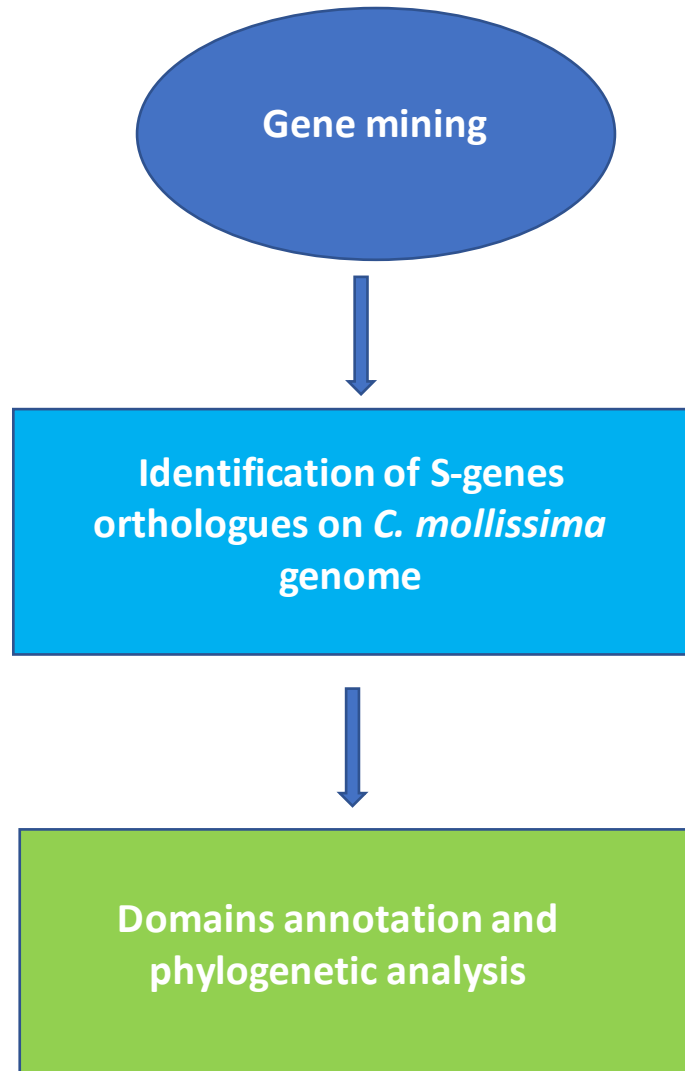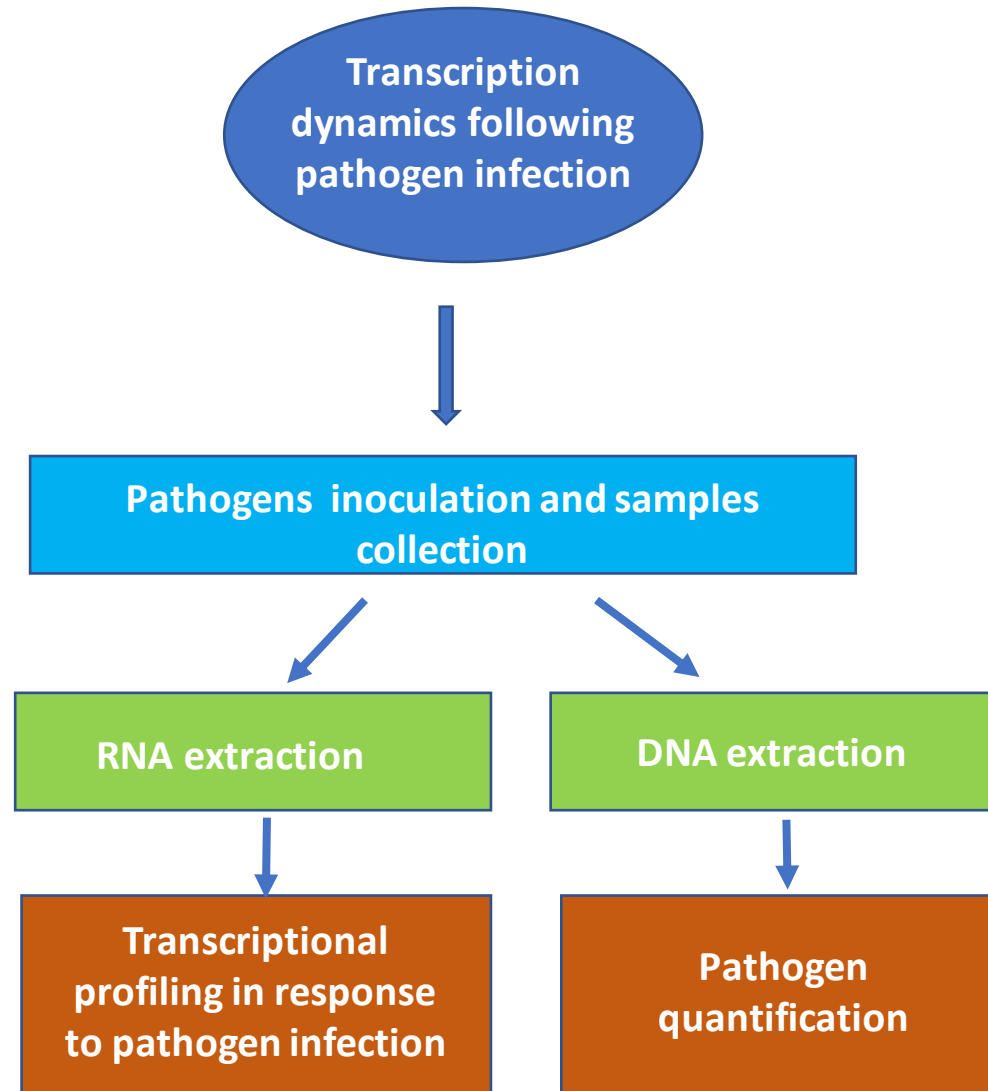

Supplement: Supplementary file 1 [file plants-10-00913-s001.zip › S1 File.pdf]

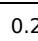

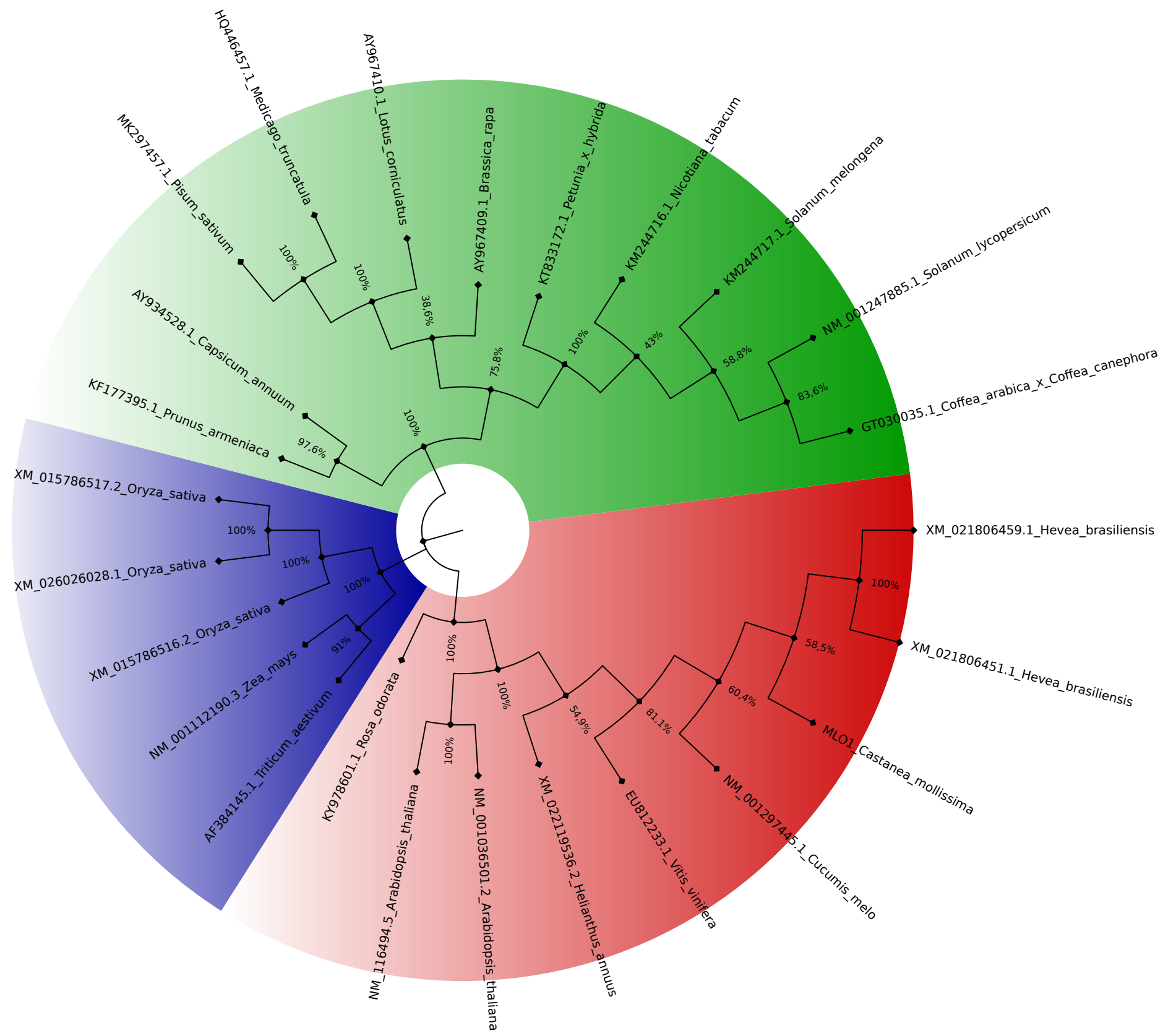

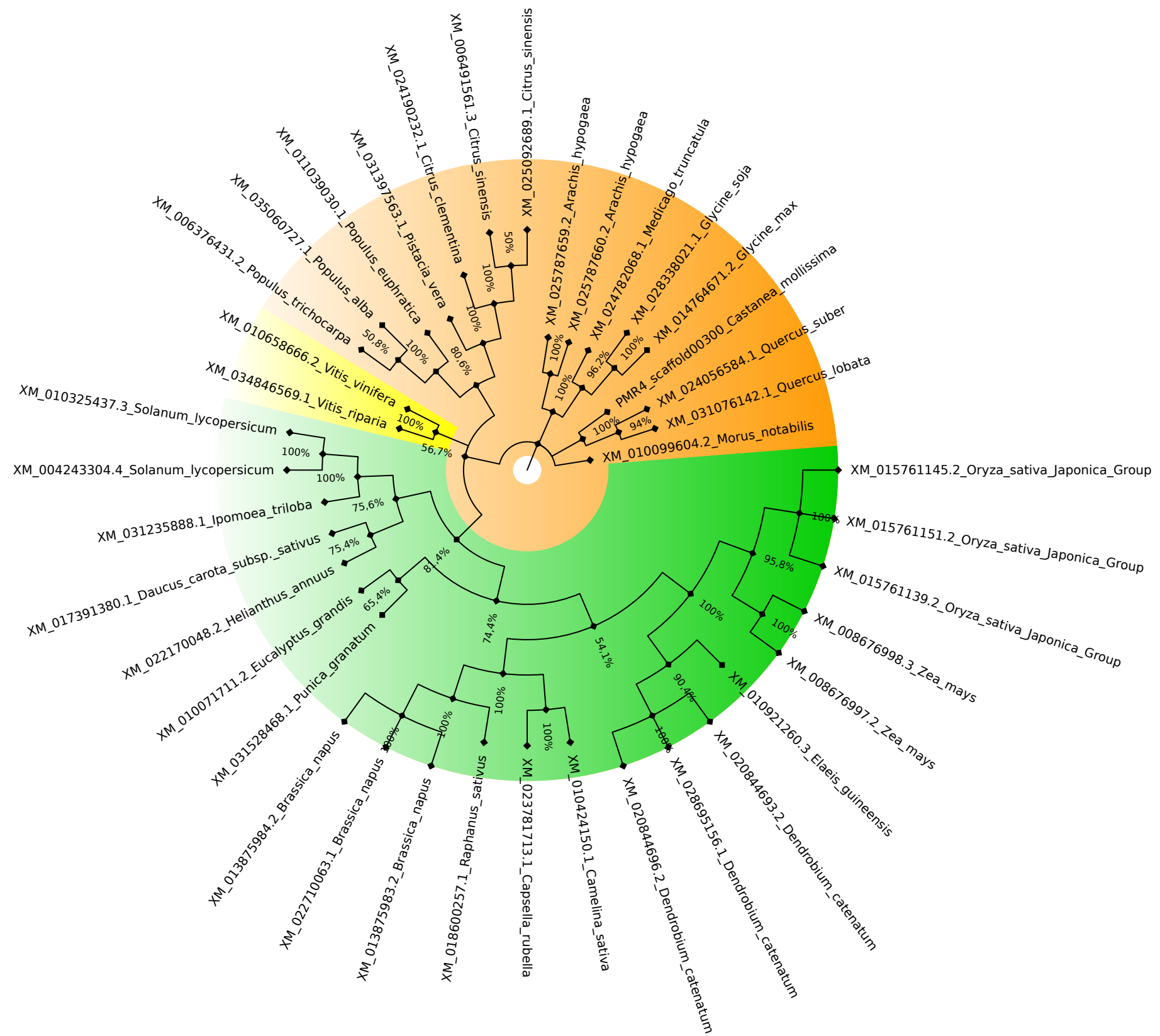

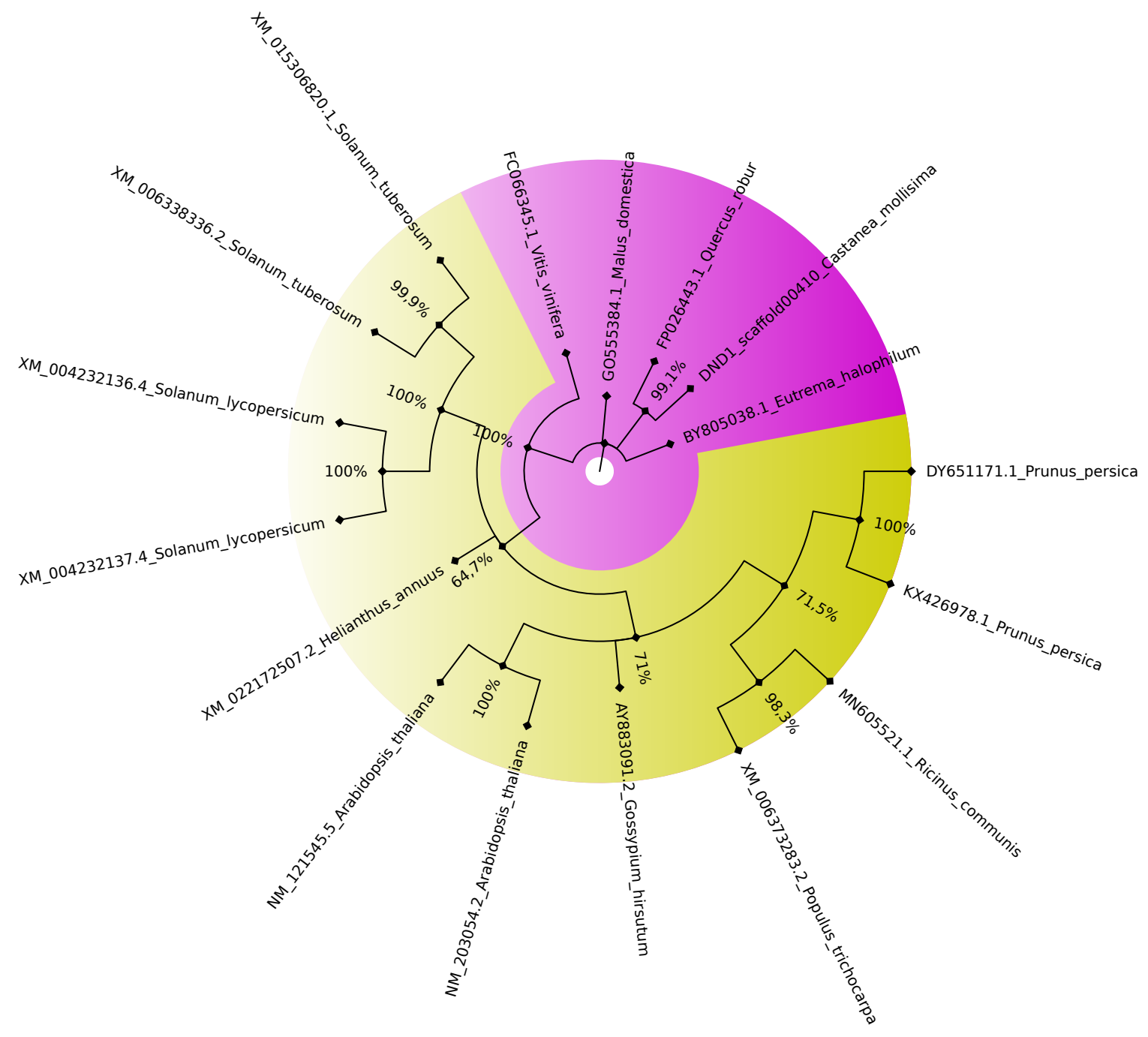

Supplement: Supplementary file 1 [file plants-10-00913-s001.zip › S4 File.pdf]
